# Supplementary material for: A new lineage of Ranavirus micropterus1 infects ornamental wrasses (Macropharyngodon choati) from the Great Barrier Reef and causes severe disease in captivity
Source: Front Vet Sci. 2026 May 18;13:1829414. doi: 10.3389/fvets.2026.1829414 (PMC13224474; doi:10.3389/fvets.2026.1829414)
Supplement: Supplementary file 1 [file Table_1.PDF]

Table S1. Viral contigs, their abundance, and closest matches on NCBI/GenBank (BLAST).

Contig	Library	Length	Top Hit	Percentage	BitScore	E.value	Abundance (reads)
k141_104076_flag1_multi4009.0000_len7540	W1-1	7540	QJE49076.1 putative myristylated membrane protein [Largemouth bass virus]	99.8	503	0	156397
k141_11782_flag0_multi1729.6004_len3499	W1-1	3499	WXI69454.1 hypothetical protein [Largemouth bass virus]	99.3	563	0	22738
k141_123371_flag1_multi1217.6985_len5215	W1-1	5215	UUY86269.1 hypothetical protein [Largemouth bass virus]	99.1	949	0	22857
k141_14017_flag0_multi2385.0592_len5308	W1-1	5308	WEI28960.1 putative D5 family NTPase/ATPase [Largemouth bass virus]	99.7	955	0	44446
k141_24902_flag0_multi7767.7579_len6672	W1-1	6672	UUY86232.1 hypothetical protein [Largemouth bass virus]	99.4	492	0	136039.9
k141_3154_flag1_multi1309.0000_len10308	W1-1	10308	QJE49122.1 putative DNA-dependent RNA polymerase b subunit [Largemouth bass virus]	99.6	1094	0	36559
k141_350_flag0_multi2739.6577_len11441	W1-1	11441	QJE49137.1 hypothetical protein LMBV_074 [Largemouth bass virus]	99.1	1162	0	108950
k141_56498_flag1_multi3565.8636_len15921	W1-1	15921	QJE49219.1 hypothetical protein LMBV_070 [Largemouth bass virus]	99.8	879	0	145873.3
k141_66726_flag0_multi4450.6891_len3776	W1-1	3776	QJE49145.1 putative proliferating cell nuclear antigen [Largemouth bass virus]	98.7	594	0	60026
k141_77380_flag0_multi4707.0000_len3152	W1-1	3152	UUY86258.1 hypothetical protein [Largemouth bass virus]	99.6	566	0	66684
k141_30232_flag0_multi1159.3025_len3625	W1-1	3625	AYV88170.1 putative DNA polymerase [Mandarin fish ranavirus]	99.5	1004	0	17020
k141_70260_flag0_multi1586.0000_len7683	W1-1	7683	AYV88134.2 putative tyrosine kinase [Mandarin fish ranavirus]	99.6	957	0	35334
k141_131099_flag1_multi3574.6533_len11182	W1-1	11182	XRL22828.1 DNA-directed RNA polymerase subunit beta [Siniperca chuatsi ranavirus]	98.7	1369	0	119903.48
k141_11817_flag1_multi65.5505_len3783	W1-2	3783	UUY86258.1 hypothetical protein [Largemouth bass virus]	99.6	559	0	1348
k141_118671_flag1_multi18.4460_len5155	W1-2	5155	WXI69454.1 hypothetical protein [Largemouth bass virus]	99.3	563	0	342
k141_126933_flag1_multi148.0000_len2271	W1-2	2271	QJE49132.1 hypothetical protein LMBV_069 [Largemouth bass virus]	99.5	597	0	1190
k141_14299_flag1_multi38.9297_len3670	W1-2	3670	QJE49145.1 putative proliferating cell nuclear antigen [Largemouth bass virus]	98.7	594	0	502
k141_19382_flag1_multi152.0000_len1759	W1-2	1759	QJE49076.1 putative myristylated membrane protein [Largemouth bass virus]	99.8	503	0	961
k141_24832_flag1_multi23.0000_len3956	W1-2	3956	QJE49137.1 hypothetical protein LMBV_074 [Largemouth bass virus]	99.1	1158	0	334
k141_33718_flag1_multi67.0000_len3844	W1-2	3844	UUY86232.1 hypothetical protein [Largemouth bass virus]	99.4	492	0	912
k141_81627_flag1_multi33.0000_len1752	W1-2	1752	UUY86267.1 ribonucleotide reductase alpha subunit [Largemouth bass virus]	100	562	0	197
k141_98543_flag1_multi15.0000_len3769	W1-2	3769	UUY86269.1 hypothetical protein [Largemouth bass virus]	99.1	949	0	197
k141_14764_flag1_multi21.0000_len3088	W1-2	3088	AYV88170.1 putative DNA polymerase [Mandarin fish ranavirus]	99.5	1004	0	217
k141_49324_flag1_multi20.0000_len3405	W1-2	3405	AYV88134.2 putative tyrosine kinase [Mandarin fish ranavirus]	99.6	957	0	243
k141_107887_flag0_multi25.6789_len3290	W1-2	3290	XRL22821.1 hypothetical protein [Siniperca chuatsi ranavirus]	99.7	952	0	286
k141_7433_flag1_multi52.9715_len5611	W1-2	5611	XRL22828.1 DNA-directed RNA polymerase subunit beta [Siniperca chuatsi ranavirus]	98.6	1311	0	1057
k141_130924_flag1_multi27.0000_len1712	W1-3	1712	UUY86261.1 hypothetical protein [Largemouth bass virus]	99.8	503	0	154
k141_213587_flag1_multi23.0000_len1897	W1-3	1897	QJE49145.1 putative proliferating cell nuclear antigen [Largemouth bass virus]	98.7	594	0	168
k141_240836_flag1_multi10.0000_len2352	W1-3	2352	WXI69454.1 hypothetical protein [Largemouth bass virus]	99.3	561	0	78
k141_250055_flag1_multi8.0000_len2156	W1-3	2156	WXI69547.1 DNA-dependent RNA polymerase II alpha subunit [Largemouth bass virus]	99	718	0	57
k141_72834_flag1_multi11.9295_len1843	W1-3	1843	UUY86267.1 ribonucleotide reductase alpha subunit [Largemouth bass virus]	99.8	547	0	70
k141_7620_flag0_multi19.0000_len2224	W1-3	2224	UUY86258.1 hypothetical protein [Largemouth bass virus]	99.6	566	0	151

k141_78202_flag1_multi62.0000_len2151	W1-3	2151	UUY86232.1 hypothetical protein [Largemouth bass virus]	99.4	492	0	459
k141_25368_flag1_multi416.0000_len1486	W1-3	1486	UVF58785.1 MAG: major capsid protein [Halichoeres melanurus ranavirus]	98.3	459	0	2210
k141_94625_flag1_multi66.0000_len1634	W1-3	1634	UVF58790.1 MAG: myristylated membrane protein, partial [Halichoeres melanurus ranavirus]	100	496	0	395
k141_1107_flag1_multi10.0000_len1982	W1-3	1982	AYV88134.2 putative tyrosine kinase [Mandarin fish ranavirus]	99.4	642	0	72
k141_149749_flag1_multi13.0000_len2499	W1-3	2499	AYV88199.1 hypothetical protein [Mandarin fish ranavirus]	99.5	746	0	126
k141_164954_flag1_multi52.0000_len2484	W1-3	2484	WHA35678.1 hypothetical protein SCRaV_86R [Siniperca chuatsi ranavirus]	95.8	660	0	493
k141_108285_flag1_multi597.5245_len5340	W2-1	5340	WXI69454.1 hypothetical protein [Largemouth bass virus]	99.3	556	0	10782
k141_26739_flag1_multi926.6753_len4872	W2-1	4872	QJE49122.1 putative DNA-dependent RNA polymerase b subunit [Largemouth bass virus]	99.6	1094	0	15422
k141_335_flag0_multi1664.4011_len3701	W2-1	3701	QJE49145.1 putative proliferating cell nuclear antigen [Largemouth bass virus]	98.7	594	0	22407
k141_35379_flag1_multi2963.4315_len4275	W2-1	4275	UUY86232.1 hypothetical protein [Largemouth bass virus]	99.4	492	0	43093
k141_57335_flag1_multi3436.5611_len5297	W2-1	5297	QJE49219.1 hypothetical protein LMBV_070 [Largemouth bass virus]	99.8	879	0	119745.15
k141_85599_flag1_multi571.0000_len7666	W2-1	7666	QJE49137.1 hypothetical protein LMBV_074 [Largemouth bass virus]	99.1	1162	0	15584
k141_94752_flag1_multi442.7351_len7846	W2-1	7846	UUY86269.1 hypothetical protein [Largemouth bass virus]	99.1	949	0	11857
k141_9957_flag1_multi870.0000_len7268	W2-1	7268	UUY86258.1 hypothetical protein [Largemouth bass virus]	99.6	566	0	20795.45
k141_50607_flag1_multi13727.0000_len4186	W2-1	4186	UVF58785.1 MAG: major capsid protein [Halichoeres melanurus ranavirus]	99.8	462	0	218588.45
k141_108732_flag1_multi513.0632_len12644	W2-1	12644	AYV88170.1 putative DNA polymerase [Mandarin fish ranavirus]	99.5	1004	0	20583.24
k141_82684_flag1_multi479.0000_len8192	W2-1	8192	AYV88134.2 putative tyrosine kinase [Mandarin fish ranavirus]	99.6	957	0	13572
k141_110649_flag0_multi1198.8755_len3184	W2-1	3184	XRL22821.1 hypothetical protein [Siniperca chuatsi ranavirus]	99.7	933	0	11131
k141_50351_flag1_multi1577.9690_len7565	W2-1	7565	XRL22828.1 DNA-directed RNA polymerase subunit beta [Siniperca chuatsi ranavirus]	98.7	1369	0	41964.37
k141_108957_flag1_multi161.8953_len3236	W2-2	3236	WEI28960.1 putative D5 family NTPase/ATPase [Largemouth bass virus]	99.7	955	0	1893
k141_14040_flag0_multi447.0000_len9258	W2-2	9258	QJE49076.1 putative myristylated membrane protein [Largemouth bass virus]	99.8	503	0	12373
k141_1415_flag1_multi277.6054_len3813	W2-2	3813	UUY86258.1 hypothetical protein [Largemouth bass virus]	99.6	566	0	5014.41
k141_38373_flag1_multi107.0000_len6712	W2-2	6712	QJE49122.1 putative DNA-dependent RNA polymerase b subunit [Largemouth bass virus]	99.6	1094	0	2507
k141_65096_flag1_multi284.6073_len4847	W2-2	4847	QJE49219.1 hypothetical protein LMBV_070 [Largemouth bass virus]	99.8	879	0	4896
k141_71280_flag0_multi193.7902_len3669	W2-2	3669	QJE49145.1 putative proliferating cell nuclear antigen [Largemouth bass virus]	98.7	594	0	3902
k141_88771_flag1_multi425.0000_len9603	W2-2	9603	UUY86232.1 hypothetical protein [Largemouth bass virus]	99.4	492	0	14483
k141_97130_flag1_multi111.0000_len12670	W2-2	12670	QJE49137.1 hypothetical protein LMBV_074 [Largemouth bass virus]	99.1	1162	0	4869
k141_137359_flag1_multi175.0991_len6398	W2-2	6398	AYV88170.1 putative DNA polymerase [Mandarin fish ranavirus]	99.5	1004	0	4310
k141_7301_flag1_multi101.7366_len15158	W2-2	15158	AYV88134.2 putative tyrosine kinase [Mandarin fish ranavirus]	99.6	957	0	5111
k141_32707_flag1_multi244.0000_len5956	W2-2	5956	XRL22828.1 DNA-directed RNA polymerase subunit beta [Siniperca chuatsi ranavirus]	98.7	1369	0	5175
k141_127614_flag1_multi1038.1650_len3760	W2-3	3760	UUY86232.1 hypothetical protein [Largemouth bass virus]	99.4	492	0	12938
k141_132806_flag1_multi177.6741_len6659	W2-3	6659	QJE49137.1 hypothetical protein LMBV_074 [Largemouth bass virus]	99.1	1162	0	3873.86
k141_144911_flag0_multi268.0201_len6158	W2-3	6158	QJE49122.1 putative DNA-dependent RNA polymerase b subunit [Largemouth bass virus]	99.6	1094	0	5524
k141_25177_flag1_multi261.0000_len4927	W2-3	4927	WEI28960.1 putative D5 family NTPase/ATPase [Largemouth bass virus]	99.7	955	0	4209

k141_51782_flag1_multi267.0000_len5316	W2-3	5316	WXI69454.1 hypothetical protein [Largemouth bass virus]	99.3	563	0	4611
k141_73830_flag1_multi296.0000_len6590	W2-3	6590	QJE49145.1 putative proliferating cell nuclear antigen [Largemouth bass virus]	98.7	594	0	6777
k141_77026_flag1_multi396.0000_len4992	W2-3	4992	QJE49219.1 hypothetical protein LMBV_070 [Largemouth bass virus]	99.8	879	0	6319
k141_88335_flag1_multi509.6523_len3517	W2-3	3517	UUY86258.1 hypothetical protein [Largemouth bass virus]	99.6	566	0	4305.52
k141_96175_flag1_multi55.0000_len6081	W2-3	6081	UUY86269.1 hypothetical protein [Largemouth bass virus]	99.1	949	0	1147.19
k141_169500_flag0_multi784.0000_len4173	W2-3	4173	UVF58785.1 MAG: major capsid protein [Halichoeres melanurus ranavirus]	100	463	0	19404
k141_143344_flag0_multi325.7912_len6344	W2-3	6344	AYV88170.1 putative DNA polymerase [Mandarin fish ranavirus]	99.5	1004	0	6555.45
k141_25074_flag1_multi163.4244_len6371	W2-3	6371	AYV88134.2 putative tyrosine kinase [Mandarin fish ranavirus]	99.6	957	0	2273
k141_11980_flag0_multi528.6306_len5062	W2-3	5062	XRL22828.1 DNA-directed RNA polymerase subunit beta [Siniperca chuatsi ranavirus]	98.5	1138	0	12228
k141_11371_flag1_multi19.0000_len1717	W3-1	1717	UUY86258.1 hypothetical protein [Largemouth bass virus]	99.6	566	0	120
k141_19214_flag1_multi7.0000_len1715	W3-1	1715	UUY86267.1 ribonucleotide reductase alpha subunit [Largemouth bass virus]	100	561	0	41
k141_22867_flag1_multi71.0000_len2052	W3-1	2052	UUY86232.1 hypothetical protein [Largemouth bass virus]	99.4	492	0	470
k141_32023_flag1_multi7.0000_len2105	W3-1	2105	QJE49086.1 hypothetical protein LMBV_023 [Largemouth bass virus]	99.4	701	0	42
k141_3496_flag1_multi24.0000_len1712	W3-1	1712	QJE49145.1 putative proliferating cell nuclear antigen [Largemouth bass virus]	98.6	560	0	133
k141_3752_flag1_multi52.0000_len2304	W3-1	2304	QJE49132.1 hypothetical protein LMBV_069 [Largemouth bass virus]	99.7	606	0	392
k141_979_flag1_multi61.8659_len5042	W3-1	5042	UVF58790.1 MAG: myristylated membrane protein, partial [Halichoeres melanurus ranavirus]	100	494	0	1084.76
k141_22052_flag1_multi9.0000_len2072	W3-1	2072	AYV88199.1 hypothetical protein [Mandarin fish ranavirus]	98.4	548	0	56
k141_4092_flag1_multi8.0000_len2986	W3-1	2986	XRL22821.1 hypothetical protein [Siniperca chuatsi ranavirus]	99.6	919	0	76
k141_122255_flag1_multi32.0000_len3843	W3-2	3843	UUY86232.1 hypothetical protein [Largemouth bass virus]	99.4	492	0	420
k141_47706_flag1_multi24.0000_len3823	W3-2	3823	WXI69454.1 hypothetical protein [Largemouth bass virus]	99.3	556	0	302
k141_47902_flag1_multi35.0000_len1827	W3-2	1827	QJE49145.1 putative proliferating cell nuclear antigen [Largemouth bass virus]	98.7	594	0	223
k141_64244_flag1_multi42.0000_len2204	W3-2	2204	QJE49132.1 hypothetical protein LMBV_069 [Largemouth bass virus]	99.7	581	0	331
k141_65531_flag1_multi33.0000_len1715	W3-2	1715	UUY86258.1 hypothetical protein [Largemouth bass virus]	99.6	563	0	195
k141_74124_flag1_multi4.0000_len1638	W3-2	1638	UUY86269.1 hypothetical protein [Largemouth bass virus]	99.2	525	0	22
k141_79031_flag1_multi9.0000_len2553	W3-2	2553	QJE49122.1 putative DNA-dependent RNA polymerase b subunit [Largemouth bass virus]	99.5	846	0	79
k141_80253_flag1_multi26.8755_len2439	W3-2	2439	UUY86267.1 ribonucleotide reductase alpha subunit [Largemouth bass virus]	100	562	0	215
k141_9285_flag1_multi54.0000_len4722	W3-2	4722	QJE49076.1 putative myristylated membrane protein [Largemouth bass virus]	99.8	503	0	875
k141_114091_flag1_multi124.0000_len3305	W3-2	3305	UVF58785.1 MAG: major capsid protein [Halichoeres melanurus ranavirus]	100	463	0	1534
k141_101487_flag1_multi30.0000_len4884	W3-2	4884	AYV88134.2 putative tyrosine kinase [Mandarin fish ranavirus]	99.6	957	0	535
k141_122507_flag0_multi28.8746_len5052	W3-2	5052	AYV88170.1 putative DNA polymerase [Mandarin fish ranavirus]	99.5	1004	0	421
k141_95189_flag1_multi13.0000_len2500	W3-2	2500	AYV88199.1 hypothetical protein [Mandarin fish ranavirus]	99.5	777	0	117
k141_109742_flag1_multi28.0000_len7125	W3-2	7125	XRL22828.1 DNA-directed RNA polymerase subunit beta [Siniperca chuatsi ranavirus]	98.6	1261	0	715
k141_89037_flag1_multi20.8536_len3284	W3-2	3284	XRL22821.1 hypothetical protein [Siniperca chuatsi ranavirus]	99.7	953	0	228
k141_136_flag0_multi9804.0000_len6482	W4-1	6482	QJE49219.1 hypothetical protein LMBV_070 [Largemouth bass virus]	99.8	879	0	261945.95
k141_13896_flag1_multi1558.9071_len4491	W4-1	4491	UUY86269.1 hypothetical protein [Largemouth bass virus]	99.1	949	0	24781
k141_58595_flag1_multi1483.0000_len2910	W4-1	2910	UUY86267.1 ribonucleotide reductase alpha subunit [Largemouth bass virus]	100	562	0	16310.08

k141_70699_flag1_multi9037.1091_len9998	W4-1	9998	QJE49076.1 putative myristylated membrane protein [Largemouth bass virus]	99.8	503	0	433969.74
k141_71500_flag1_multi4763.2646_len3418	W4-1	3418	UUY86258.1 hypothetical protein [Largemouth bass virus]	99.6	566	0	60669.35
k141_88838_flag1_multi1052.9493_len7818	W4-1	7818	WXI69454.1 hypothetical protein [Largemouth bass virus]	99.3	535	0	26432
k141_94885_flag1_multi1935.0016_len9106	W4-1	9106	QJE49122.1 putative DNA-dependent RNA polymerase b subunit [Largemouth bass virus]	99.6	1094	0	66440.79
k141_96684_flag1_multi7454.3768_len5813	W4-1	5813	QJE49145.1 putative proliferating cell nuclear antigen [Largemouth bass virus]	98.7	594	0	123701.87
k141_101816_flag1_multi1894.0000_len12206	W4-1	12206	AYV88134.2 putative tyrosine kinase [Mandarin fish ranavirus]	99.6	957	0	81964
k141_40315_flag0_multi1856.0000_len7957	W4-1	7957	AYV88170.1 putative DNA polymerase [Mandarin fish ranavirus]	99.5	994	0	43990
k141_67569_flag0_multi1303.0000_len2724	W4-1	2724	AYV88199.1 hypothetical protein [Mandarin fish ranavirus]	99.4	466	0	22449.04
k141_31830_flag1_multi7502.0000_len10097	W4-1	10097	XRL22828.1 DNA-directed RNA polymerase subunit beta [Siniperca chuatsi ranavirus]	98.7	1369	0	267531
k141_99002_flag1_multi3285.0000_len4973	W4-1	4973	XRL22821.1 hypothetical protein [Siniperca chuatsi ranavirus]	99.7	952	0	57863
k141_114409_flag1_multi94.5383_len4824	W4-2	4824	QJE49137.1 hypothetical protein LMBV_074 [Largemouth bass virus]	99.1	1162	0	1604
k141_116824_flag1_multi201.9411_len2075	W4-2	2075	UUY86267.1 ribonucleotide reductase alpha subunit [Largemouth bass virus]	100	557	0	1402
k141_34734_flag1_multi91.5859_len5195	W4-2	5195	WXI69454.1 hypothetical protein [Largemouth bass virus]	99.3	556	0	1613
k141_512_flag1_multi332.0000_len4007	W4-2	4007	UUY86232.1 hypothetical protein [Largemouth bass virus]	99.4	492	0	4515
k141_79837_flag1_multi286.3146_len5983	W4-2	5983	QJE49219.1 hypothetical protein LMBV_070 [Largemouth bass virus]	99.8	879	0	6104
k141_91778_flag0_multi773.0000_len5473	W4-2	5473	QJE49145.1 putative proliferating cell nuclear antigen [Largemouth bass virus]	98.7	594	0	15692
k141_94638_flag0_multi304.0843_len9363	W4-2	9363	QJE49076.1 putative myristylated membrane protein [Largemouth bass virus]	99.8	503	0	8574
k141_95585_flag1_multi309.9457_len3880	W4-2	3880	UUY86258.1 hypothetical protein [Largemouth bass virus]	99.6	566	0	4457
k141_97921_flag1_multi47.0000_len3776	W4-2	3776	UUY86269.1 hypothetical protein [Largemouth bass virus]	99	947	0	601
k141_101067_flag0_multi114.9077_len2525	W4-2	2525	AYV88179.2 putative DNA dependent RNA polymerase II second largest subunit [Mandarin fish ranavirus]	99.7	792	0	479
k141_19186_flag1_multi158.9555_len5621	W4-2	5621	AYV88170.1 putative DNA polymerase [Mandarin fish ranavirus]	99.5	1004	0	2997
k141_97174_flag1_multi64.0000_len6146	W4-2	6146	AYV88134.2 putative tyrosine kinase [Mandarin fish ranavirus]	98.7	954	0	1392
k141_11277_flag0_multi193.6347_len3267	W4-2	3267	XRL22821.1 hypothetical protein [Siniperca chuatsi ranavirus]	99.7	951	0	2131
k141_85139_flag0_multi232.2412_len5970	W4-2	5970	XRL22828.1 DNA-directed RNA polymerase subunit beta [Siniperca chuatsi ranavirus]	98.7	1369	0	4856
k141_123303_flag1_multi161.0000_len1826	W4-3	1826	UUY86258.1 hypothetical protein [Largemouth bass virus]	99.6	566	0	1057
k141_136013_flag1_multi179.0000_len9582	W4-3	9582	QJE49219.1 hypothetical protein LMBV_070 [Largemouth bass virus]	99.8	879	0	6044
k141_187617_flag1_multi67.1190_len3973	W4-3	3973	QJE49137.1 hypothetical protein LMBV_074 [Largemouth bass virus]	99	1153	0	974
k141_206346_flag1_multi311.0000_len3621	W4-3	3621	UUY86232.1 hypothetical protein [Largemouth bass virus]	99.4	492	0	3957
k141_212115_flag0_multi73.2792_len2369	W4-3	2369	UUY86267.1 ribonucleotide reductase alpha subunit [Largemouth bass virus]	100	562	0	575
k141_220412_flag0_multi103.4052_len3951	W4-3	3951	QJE49145.1 putative proliferating cell nuclear antigen [Largemouth bass virus]	98.6	583	0	1431
k141_66255_flag1_multi66.0000_len4687	W4-3	4687	QJE49122.1 putative DNA-dependent RNA polymerase b subunit [Largemouth bass virus]	99.6	1090	0	1070
k141_81724_flag1_multi44.0000_len3681	W4-3	3681	UUY86269.1 hypothetical protein [Largemouth bass virus]	99.1	948	0	567
k141_90633_flag1_multi52.0000_len5041	W4-3	5041	WXI69454.1 hypothetical protein [Largemouth bass virus]	99.3	563	0	890
k141_183438_flag0_multi1328.0000_len5783	W4-3	5783	AVJ54673.1 major capsid protein [Smallmouth bass virus]	98.5	462	0	17888

k141_10150_flag1_multi450.0000_len1700	W4-3	1700	UVF58790.1 MAG: myristylated membrane protein, partial [Halichoeres melanurus ranavirus]	99.8	483	0	2725
k141_122155_flag1_multi42.0000_len3032	W4-3	3032	AYV88170.1 putative DNA polymerase [Mandarin fish ranavirus]	99.5	1003	0	405
k141_51354_flag1_multi70.0000_len3395	W4-3	3395	AYV88134.2 putative tyrosine kinase [Mandarin fish ranavirus]	99.6	954	0	839
k141_8642_flag1_multi23.0000_len1393	W4-3	1393	AYV88130.2 putative ATPase-dependent protease [Mandarin fish ranavirus]	99.1	463	0	113
k141_149432_flag1_multi52.0000_len4448	W4-3	4448	XRL22828.1 DNA-directed RNA polymerase subunit beta [Siniperca chuatsi ranavirus]	98.5	1303	0	716
k141_65910_flag1_multi66.0000_len3105	W4-3	3105	XRL22821.1 hypothetical protein [Siniperca chuatsi ranavirus]	99.7	953	0	704
k141_151962_flag1_multi2476.0000_len15670	W5-1	15670	QJE49122.1 putative DNA-dependent RNA polymerase b subunit [Largemouth bass virus]	99.6	1094	0	106891
k141_16814_flag1_multi7296.9623_len31929	W5-1	31929	QJE49137.1 hypothetical protein LMBV_074 [Largemouth bass virus]	99.1	1162	0	909850.07
k141_72672_flag0_multi3360.0000_len9773	W5-1	9773	WEI28960.1 putative D5 family NTPase/ATPase [Largemouth bass virus]	99.7	955	0	142045.34
k141_95291_flag1_multi13356.0000_len10588	W5-1	10588	QJE49076.1 putative myristylated membrane protein [Largemouth bass virus]	99.8	503	0	512209.23
k141_115668_flag0_multi13643.0000_len4942	W5-1	4942	UVF58785.1 MAG: major capsid protein [Halichoeres melanurus ranavirus]	99.6	461	0	948394.8
k141_136027_flag0_multi4045.1620_len12243	W5-1	12243	AYV88134.2 putative tyrosine kinase [Mandarin fish ranavirus]	99.6	957	0	144945.87
k141_150520_flag1_multi5953.1972_len22349	W5-1	22349	AYV88170.1 putative DNA polymerase [Mandarin fish ranavirus]	99.5	1004	0	354285.82
k141_6970_flag0_multi8017.0000_len13241	W5-1	13241	XRL22828.1 DNA-directed RNA polymerase subunit beta [Siniperca chuatsi ranavirus]	98.7	1369	0	527250.93
k141_10305_flag1_multi9.0000_len1469	W5-2	1469	QJE49122.1 putative DNA-dependent RNA polymerase b subunit [Largemouth bass virus]	99.8	489	0	45
k141_108124_flag0_multi15.0000_len2780	W5-2	2780	WXI69454.1 hypothetical protein [Largemouth bass virus]	99.3	563	0	146
k141_12015_flag1_multi48.0000_len2001	W5-2	2001	UUY86232.1 hypothetical protein [Largemouth bass virus]	99.4	492	0	296
k141_132087_flag1_multi7.0000_len2466	W5-2	2466	UUY86269.1 hypothetical protein [Largemouth bass virus]	98.9	821	0	63
k141_150044_flag1_multi7.0000_len2068	W5-2	2068	WXI69547.1 DNA-dependent RNA polymerase II alpha subunit [Largemouth bass virus]	99	689	0	47
k141_158116_flag1_multi34.8608_len4781	W5-2	4781	QJE49219.1 hypothetical protein LMBV_070 [Largemouth bass virus]	99.8	879	0	492
k141_162810_flag1_multi9.0000_len1986	W5-2	1986	QJE49137.1 hypothetical protein LMBV_074 [Largemouth bass virus]	99.6	470	0	58
k141_20688_flag1_multi13.0000_len1769	W5-2	1769	UUY86267.1 ribonucleotide reductase alpha subunit [Largemouth bass virus]	100	562	0	79
k141_48388_flag1_multi6.0000_len2011	W5-2	2011	QJE49137.1 hypothetical protein LMBV_074 [Largemouth bass virus]	98.7	670	0	37
k141_54394_flag1_multi11.0000_len3143	W5-2	3143	WEI28960.1 putative D5 family NTPase/ATPase [Largemouth bass virus]	99.7	955	0	109
k141_61317_flag1_multi28.0000_len1670	W5-2	1670	UUY86258.1 hypothetical protein [Largemouth bass virus]	99.6	556	0	153
k141_92401_flag1_multi8.0000_len1405	W5-2	1405	QJE49086.1 hypothetical protein LMBV_023 [Largemouth bass virus]	99.4	468	0	40
k141_15294_flag1_multi237.4203_len1616	W5-2	1616	UVF58785.1 MAG: major capsid protein [Halichoeres melanurus ranavirus]	99.8	455	0	1481
k141_59161_flag1_multi68.0000_len2196	W5-2	2196	UVF58790.1 MAG: myristylated membrane protein, partial [Halichoeres melanurus ranavirus]	99.4	494	0	511
k141_62061_flag1_multi10.0000_len2511	W5-2	2511	AYV88170.1 putative DNA polymerase [Mandarin fish ranavirus]	99.5	837	0	80
k141_136644_flag1_multi11.9617_len1865	W5-3	1865	UUY86267.1 ribonucleotide reductase alpha subunit [Largemouth bass virus]	100	562	0	64
k141_196909_flag1_multi137.0000_len3174	W5-3	3174	UUY86232.1 hypothetical protein [Largemouth bass virus]	99.4	492	0	525.04
k141_216948_flag1_multi24.8417_len2637	W5-3	2637	UUY86258.1 hypothetical protein [Largemouth bass virus]	99.6	566	0	353
k141_220256_flag1_multi20.9726_len1672	W5-3	1672	WXI69454.1 hypothetical protein [Largemouth bass virus]	99.2	517	0	111

k141_246267_flag1_multi15.0000_len1645	W5-3	1645	QJE49132.1 hypothetical protein LMBV_069 [Largemouth bass virus]	99.5	547	0	77
k141_257081_flag1_multi34.0000_len1774	W5-3	1774	UUY86261.1 hypothetical protein [Largemouth bass virus]	99.8	503	0	222
k141_7685_flag0_multi16.8382_len2317	W5-3	2317	QJE49145.1 putative proliferating cell nuclear antigen [Largemouth bass virus]	98.7	594	0	114
k141_267055_flag1_multi8.0000_len1355	W5-3	1355	UVF58793.1 MAG: DNA polymerase [Halichoeres melanurus ranavirus]	99.8	451	0	32
k141_171238_flag1_multi7.0000_len1401	W5-3	1401	AYV88199.1 hypothetical protein [Mandarin fish ranavirus]	98.7	464	0	31
k141_24543_flag1_multi6.0000_len1809	W5-3	1809	WAK75092.1 putative D5 family NTPase/ATPase [Mandarin fish ranavirus]	99.5	584	0	39
k141_246234_flag1_multi6.0000_len1528	W5-3	1528	AYV88134.2 putative tyrosine kinase [Mandarin fish ranavirus]	99.6	503	0	27
k141_19344_flag0_multi429.1133_len5155	W6-1	5155	UUY86258.1 hypothetical protein [Largemouth bass virus]	99.6	566	0	7840.26
k141_20527_flag1_multi107.6398_len1965	W6-1	1965	UUY86267.1 ribonucleotide reductase alpha subunit [Largemouth bass virus]	100	555	0	696
k141_22548_flag1_multi176.0000_len4596	W6-1	4596	QJE49137.1 hypothetical protein LMBV_074 [Largemouth bass virus]	99.1	1162	0	2760
k141_25837_flag1_multi1932.1295_len7925	W6-1	7925	QJE49145.1 putative proliferating cell nuclear antigen [Largemouth bass virus]	98.7	594	0	49374
k141_2784_flag1_multi106.0000_len6083	W6-1	6083	WXI69454.1 hypothetical protein [Largemouth bass virus]	99.3	551	0	2001
k141_66286_flag0_multi491.0000_len9199	W6-1	9199	QJE49219.1 hypothetical protein LMBV_070 [Largemouth bass virus]	99.8	879	0	16678
k141_80119_flag0_multi138.0000_len6844	W6-1	6844	QJE49122.1 putative DNA-dependent RNA polymerase b subunit [Largemouth bass virus]	99.6	1094	0	3268
k141_84444_flag1_multi95.4697_len3837	W6-1	3837	UUY86269.1 hypothetical protein [Largemouth bass virus]	98.9	947	0	1231
k141_87520_flag1_multi484.9576_len3752	W6-1	3752	UUY86232.1 hypothetical protein [Largemouth bass virus]	99.4	492	0	6321
k141_29519_flag1_multi100.0523_len3719	W6-1	3719	AYV88134.2 putative tyrosine kinase [Mandarin fish ranavirus]	99.6	957	0	6752.69
k141_30779_flag0_multi175.0000_len3472	W6-1	3472	AYV88170.1 putative DNA polymerase [Mandarin fish ranavirus]	99.5	1004	0	1327
k141_5301_flag1_multi40.0000_len2732	W6-1	2732	AYV88130.2 putative ATPase-dependent protease [Mandarin fish ranavirus]	99.1	453	0	330
k141_22578_flag0_multi147.7496_len2489	W6-1	2489	XRL22821.1 hypothetical protein [Siniperca chuatsi ranavirus]	100	704	0	1285
k141_23266_flag1_multi354.4227_len5797	W6-1	5797	XRL22828.1 DNA-directed RNA polymerase subunit beta [Siniperca chuatsi ranavirus]	98.7	1369	0	7134
k141_130447_flag1_multi48.6197_len2208	W6-2	2208	QJE49145.1 putative proliferating cell nuclear antigen [Largemouth bass virus]	98.6	560	0	396
k141_131598_flag1_multi30.9528_len3322	W6-2	3322	WEI28960.1 putative D5 family NTPase/ATPase [Largemouth bass virus]	99.5	955	0	359
k141_154690_flag1_multi35.0000_len4785	W6-2	4785	QJE49137.1 hypothetical protein LMBV_074 [Largemouth bass virus]	99.1	1162	0	432
k141_16749_flag1_multi15.0000_len3615	W6-2	3615	UUY86269.1 hypothetical protein [Largemouth bass virus]	99.1	949	0	193
k141_19388_flag1_multi48.0000_len1753	W6-2	1753	UUY86267.1 ribonucleotide reductase alpha subunit [Largemouth bass virus]	100	562	0	288
k141_21673_flag1_multi141.0000_len3505	W6-2	3505	QJE49076.1 putative myristylated membrane protein [Largemouth bass virus]	99.8	503	0	1765
k141_37376_flag1_multi92.0000_len3641	W6-2	3641	UUY86232.1 hypothetical protein [Largemouth bass virus]	99.4	492	0	1149
k141_5715_flag1_multi34.0000_len4706	W6-2	4706	QJE49122.1 putative DNA-dependent RNA polymerase b subunit [Largemouth bass virus]	99.6	1093	0	554
k141_73980_flag0_multi1659.0000_len3644	W6-2	3644	WXI69454.1 hypothetical protein [Largemouth bass virus]	99.3	563	0	529
k141_90611_flag0_multi67.4518_len2830	W6-2	2830	UUY86258.1 hypothetical protein [Largemouth bass virus]	99.6	566	0	657
k141_99825_flag1_multi131.0000_len4738	W6-2	4738	QJE49219.1 hypothetical protein LMBV_070 [Largemouth bass virus]	99.8	879	0	1996
k141_154175_flag1_multi14.9977_len6257	W6-2	6257	AYV88134.2 putative tyrosine kinase [Mandarin fish ranavirus]	99.6	957	0	331
k141_44950_flag0_multi22.8314_len3143	W6-2	3143	AYV88170.1 putative DNA polymerase [Mandarin fish ranavirus]	99.5	999	0	237
k141_767_flag1_multi13.0000_len1469	W6-2	1469	WAK75099.1 putative DNA dependent RNA polymerase II largest subunit [Mandarin fish ranavirus]	100	448	0	67

k141_89951_flag1_multi78.0000_len4367	W6-2	4367	XRL22828.1 DNA-directed RNA polymerase subunit beta [Siniperca chuatsi ranavirus]	98.2	926	0	1209
k141_53133_flag1_multi30.0000_len1411	W6-3	1411	UVF58785.1 MAG: major capsid protein [Halichoeres melanurus ranavirus]	100	462	0	145
k141_88354_flag1_multi7.0000_len1491	W6-3	1491	UVF58790.1 MAG: myristylated membrane protein, partial [Halichoeres melanurus ranavirus]	100	492	0	38
k141_228895_flag0_multi131.9718_len2343	W5-3	2343	YP_009506759.1 major capsid protein [Santee-Cooper ranavirus]	96.5	455	1.05E-305	1152.07
k141_8776_flag1_multi14.8167_len2585	W3-2	2585	UUY86261.1 hypothetical protein [Largemouth bass virus]	99.8	503	1.68E-305	131
k141_153794_flag1_multi3.7727_len1254	W6-3	1254	WEI29006.1 putative 2-cysteine adaptor domain protein [Largemouth bass virus]	99.8	417	6.42E-305	17
k141_138302_flag1_multi5.0000_len1279	W5-2	1279	WHA35533.1 putative DNA dependent RNA polymerase A subunit [Micropterus salmoides ranavirus]	100	423	8.96E-305	21
k141_75056_flag0_multi3077.2812_len2573	W4-1	2573	QJE49137.1 hypothetical protein LMBV_074 [Largemouth bass virus]	98.7	458	8.37E-304	18491
k141_87621_flag1_multi58.0000_len2238	W6-2	2238	WEI29033.1 hypothetical protein [Largemouth bass virus]	98	608	3.90E-303	525
k141_149008_flag1_multi159.0000_len2228	W4-3	2228	WEI29033.1 hypothetical protein [Largemouth bass virus]	98	608	4.95E-303	1476
k141_31245_flag0_multi472.0641_len11236	W2-2	11236	UVF58785.1 MAG: major capsid protein [Halichoeres melanurus ranavirus]	100	463	1.83E-302	19462
k141_70752_flag1_multi1921.2657_len2418	W2-1	2418	WEI29033.1 hypothetical protein [Largemouth bass virus]	98	608	5.00E-302	20174
k141_15453_flag1_multi21.0000_len2614	W3-1	2614	UUY86261.1 hypothetical protein [Largemouth bass virus]	98.4	498	1.02E-300	173
k141_7870_flag1_multi24.0000_len2179	W3-1	2179	WEI29033.1 hypothetical protein [Largemouth bass virus]	98	604	2.98E-300	194
k141_22204_flag1_multi53.9771_len2457	W1-2	2457	UUY86261.1 hypothetical protein [Largemouth bass virus]	99.8	487	2.87E-299	435
k141_229213_flag1_multi13.0000_len1643	W5-3	1643	UVF58788.1 MAG: helicase, partial [Halichoeres melanurus ranavirus]	99.8	403	6.82E-296	70
k141_70101_flag1_multi46.0000_len1236	W1-2	1236	QJE49149.1 putative immediate early protein ICP-46 [Largemouth bass virus]	100	382	7.74E-296	193
k141_134802_flag1_multi67.0000_len1244	W6-2	1244	QJE49149.1 putative immediate early protein ICP-46 [Largemouth bass virus]	100	382	8.74E-296	272
k141_151400_flag1_multi17.9052_len1755	W5-2	1755	UVF58788.1 MAG: helicase, partial [Halichoeres melanurus ranavirus]	99.8	403	2.85E-295	99
k141_51796_flag1_multi8.0000_len1275	W1-3	1275	UUY86269.1 hypothetical protein [Largemouth bass virus]	99.1	424	4.15E-295	34
k141_2247_flag1_multi165.8378_len1362	W4-2	1362	QJE49149.1 putative immediate early protein ICP-46 [Largemouth bass virus]	100	382	5.93E-295	732
k141_6757_flag1_multi27.0000_len1204	W3-1	1204	QJE49149.1 putative immediate early protein ICP-46 [Largemouth bass virus]	99.7	382	1.73E-294	106
k141_71610_flag1_multi17.9846_len1312	W5-2	1312	QJE49149.1 putative immediate early protein ICP-46 [Largemouth bass virus]	100	380	4.26E-293	72
k141_69739_flag1_multi84.0000_len4471	W1-2	4471	WEI29033.1 hypothetical protein [Largemouth bass virus]	98	608	2.70E-292	1450
k141_203133_flag1_multi18.0000_len1246	W5-3	1246	QJE49149.1 putative immediate early protein ICP-46 [Largemouth bass virus]	100	378	1.69E-291	65
k141_94858_flag0_multi126.8455_len2561	W2-3	2561	UVF58788.1 MAG: helicase, partial [Halichoeres melanurus ranavirus]	99.8	403	5.97E-291	1021
k141_112871_flag1_multi10.0000_len1658	W5-2	1658	QJE49110.1 hypothetical protein LMBV_047 [Largemouth bass virus]	99.5	405	5.47E-290	59
k141_37166_flag1_multi148.0000_len1250	W4-2	1250	AYV88173.2 putative ribonucleotide reductase beta subunit [Mandarin fish ranavirus]	100	387	5.84E-289	622
k141_194666_flag1_multi27.0000_len1254	W4-3	1254	AYV88173.2 putative ribonucleotide reductase beta subunit [Mandarin fish ranavirus]	100	387	6.08E-289	121
k141_67495_flag1_multi21.0000_len1285	W1-2	1285	AYV88173.2 putative ribonucleotide reductase beta subunit [Mandarin fish ranavirus]	100	387	9.45E-289	96
k141_144846_flag1_multi156.0000_len1294	W2-3	1294	AYV88173.2 putative ribonucleotide reductase beta subunit [Mandarin fish ranavirus]	100	387	1.07E-288	642
k141_64406_flag1_multi9.0000_len1307	W5-3	1307	AYV88173.2 putative ribonucleotide reductase beta subunit [Mandarin fish ranavirus]	100	387	1.78E-288	44
k141_212268_flag1_multi11.0000_len2321	W5-3	2321	QJE49110.1 hypothetical protein LMBV_047 [Largemouth bass virus]	99.5	405	2.16E-286	87
k141_52448_flag0_multi784.0000_len6091	W2-3	6091	WEI29033.1 hypothetical protein [Largemouth bass virus]	98	608	2.67E-286	8383
k141_86222_flag1_multi13.0000_len1218	W5-2	1218	AYV88173.2 putative ribonucleotide reductase beta subunit [Mandarin fish ranavirus]	100	384	3.07E-286	54
k141_128460_flag1_multi29.0000_len2453	W6-2	2453	QJE49110.1 hypothetical protein LMBV_047 [Largemouth bass virus]	99.5	405	1.05E-285	258
k141_103485_flag1_multi281.0000_len6336	W4-2	6336	WEI29033.1 hypothetical protein [Largemouth bass virus]	98	608	2.40E-285	6612

k141_31460_flag1_multi9.0000_len3848	W3-1	3848	UVF58788.1 MAG: helicase, partial [Halichoeres melanurus ranavirus]	99.8	403	8.75E-285	107
k141_88400_flag1_multi196.4808_len2614	W6-1	2614	QJE49110.1 hypothetical protein LMBV_047 [Largemouth bass virus]	99.5	405	1.43E-284	1812
k141_10177_flag1_multi3019.0000_len1951	W4-1	1951	QJE49149.1 putative immediate early protein ICP-46 [Largemouth bass virus]	100	374	1.75E-284	20970
k141_70439_flag1_multi44.0000_len2051	W6-2	2051	AYV88173.2 putative ribonucleotide reductase beta subunit [Mandarin fish ranavirus]	100	387	1.79E-284	310
k141_140188_flag1_multi9.0000_len1205	W1-3	1205	UVF58793.1 MAG: DNA polymerase [Halichoeres melanurus ranavirus]	99.8	401	3.68E-284	33
k141_31296_flag1_multi6.0000_len1179	W5-2	1179	UUY86211.1 hypothetical protein [Largemouth bass virus]	99	392	5.28E-284	25
k141_106283_flag1_multi17.0000_len1123	W1-3	1123	QJE49149.1 putative immediate early protein ICP-46 [Largemouth bass virus]	100	365	7.48E-284	62
k141_45892_flag1_multi23.5601_len2180	W3-2	2180	AYV88173.2 putative ribonucleotide reductase beta subunit [Mandarin fish ranavirus]	100	387	8.75E-284	161
k141_26717_flag1_multi10.0000_len1532	W3-1	1532	QJE49110.1 hypothetical protein LMBV_047 [Largemouth bass virus]	99.2	398	3.09E-283	53
k141_99163_flag0_multi85.1957_len2466	W6-1	2466	AYV88173.2 putative ribonucleotide reductase beta subunit [Mandarin fish ranavirus]	100	387	1.55E-281	604
k141_71351_flag1_multi26.0000_len3471	W1-2	3471	QJE49110.1 hypothetical protein LMBV_047 [Largemouth bass virus]	99.5	405	7.27E-281	325
k141_1143_flag0_multi758.3783_len2639	W1-1	2639	AYV88173.2 putative ribonucleotide reductase beta subunit [Mandarin fish ranavirus]	100	387	1.20E-280	6385
k141_140063_flag1_multi59.0000_len2210	W1-3	2210	UUY86194.1 hypothetical protein [Largemouth bass virus]	100	400	1.52E-279	501
k141_95404_flag1_multi7.0000_len2414	W1-2	2414	UUY86211.1 hypothetical protein [Largemouth bass virus]	99	395	1.79E-279	60
k141_188960_flag1_multi10.0000_len1116	W1-3	1116	XRL22821.1 hypothetical protein [Siniperca chuatsi ranavirus]	99.2	372	2.75E-279	38
k141_91115_flag1_multi34.9156_len9001	W3-2	9001	WEI29033.1 hypothetical protein [Largemouth bass virus]	98	608	4.81E-279	1036
k141_22886_flag0_multi41.8327_len2621	W3-1	2621	UUY86194.1 hypothetical protein [Largemouth bass virus]	99.8	401	1.41E-277	350
k141_103735_flag1_multi87.0000_len4425	W3-2	4425	QJE49110.1 hypothetical protein LMBV_047 [Largemouth bass virus]	99.5	405	1.01E-276	1364
k141_162545_flag1_multi21.0000_len1767	W5-2	1767	QJE49113.1 hypothetical protein LMBV_050 [Largemouth bass virus]	99.7	371	1.74E-275	115
k141_3646_flag1_multi214.4098_len5580	W4-3	5580	QJE49149.1 putative immediate early protein ICP-46 [Largemouth bass virus]	100	382	2.07E-275	4362
k141_214585_flag1_multi64.0000_len4768	W4-3	4768	QJE49110.1 hypothetical protein LMBV_047 [Largemouth bass virus]	99.5	405	2.42E-275	771
k141_101172_flag1_multi149.0000_len1306	W6-1	1306	AYV88121.1 hypothetical protein [Mandarin fish ranavirus]	98.7	374	2.80E-275	657
k141_119466_flag1_multi29.0000_len1207	W5-2	1207	QJE49145.1 putative proliferating cell nuclear antigen [Largemouth bass virus]	100	387	3.37E-275	118
k141_6081_flag1_multi17.0000_len1763	W5-3	1763	QJE49113.1 hypothetical protein LMBV_050 [Largemouth bass virus]	99.7	371	3.38E-275	90
k141_122171_flag1_multi245.3165_len2258	W2-2	2258	AYV88173.2 putative ribonucleotide reductase beta subunit [Mandarin fish ranavirus]	99.7	379	5.15E-275	1237
k141_113085_flag1_multi25.5077_len1309	W6-2	1309	AYV88121.1 hypothetical protein [Mandarin fish ranavirus]	98.7	374	1.18E-274	106
k141_133060_flag1_multi5.0000_len1217	W5-3	1217	QJE49102.1 putative DNA dependent RNA polymerase a subunit [Largemouth bass virus]	98.3	405	5.53E-273	19
k141_52929_flag1_multi51.0000_len2435	W6-2	2435	QJE49113.1 hypothetical protein LMBV_050 [Largemouth bass virus]	99.7	371	8.85E-272	422
k141_109800_flag1_multi444.0000_len5408	W2-1	5408	QJE49110.1 hypothetical protein LMBV_047 [Largemouth bass virus]	99.5	405	9.16E-272	8543
k141_126922_flag1_multi56.0000_len2454	W4-3	2454	QJE49113.1 hypothetical protein LMBV_050 [Largemouth bass virus]	99.7	371	1.09E-271	485
k141_59622_flag1_multi171.0000_len5790	W2-3	5790	QJE49110.1 hypothetical protein LMBV_047 [Largemouth bass virus]	99.5	405	1.25E-271	3447
k141_118046_flag1_multi515.1995_len5896	W4-2	5896	QJE49110.1 hypothetical protein LMBV_047 [Largemouth bass virus]	99.5	405	2.74E-271	10763
k141_72709_flag0_multi9814.7139_len5965	W4-1	5965	QJE49110.1 hypothetical protein LMBV_047 [Largemouth bass virus]	99.5	405	4.72E-271	160115
k141_148402_flag1_multi30.0000_len2593	W1-2	2593	QJE49113.1 hypothetical protein LMBV_050 [Largemouth bass virus]	99.7	371	5.48E-271	280
k141_85491_flag1_multi145.0000_len4316	W6-1	4316	QJE49149.1 putative immediate early protein ICP-46 [Largemouth bass virus]	100	368	3.46E-270	1936
k141_221601_flag1_multi12.0000_len2294	W5-3	2294	AYV88121.1 hypothetical protein [Mandarin fish ranavirus]	98.7	374	7.10E-270	83
k141_70364_flag1_multi38.0000_len2651	W1-2	2651	QJE49122.1 putative DNA-dependent RNA polymerase b subunit [Largemouth bass virus]	100	379	1.55E-269	361
k141_99425_flag1_multi131.4283_len3349	W6-2	3349	UUY86194.1 hypothetical protein [Largemouth bass virus]	100	394	2.32E-269	1305

k141_191652_flag1_multi10.0000_len1328	W5-3	1328	XRL22821.1 hypothetical protein [Siniperca chuatsi ranavirus]	100	366	1.61E-268	39
k141_99763_flag1_multi24.0000_len2262	W1-2	2262	AYV88121.1 hypothetical protein [Mandarin fish ranavirus]	98.7	372	2.24E-268	196
k141_75207_flag0_multi172.1696_len3184	W6-1	3184	QJE49113.1 hypothetical protein LMBV_050 [Largemouth bass virus]	99.7	371	4.22E-268	2263.37
k141_242733_flag1_multi5.0000_len1107	W6-3	1107	WEI28972.1 CTD-phosphotransferase [Largemouth bass virus]	99.2	368	1.13E-267	13
k141_21420_flag1_multi144.0000_len2159	W4-2	2159	UUY86210.1 putative RNAseIII [Largemouth bass virus]	99.7	385	1.15E-267	1031
k141_52312_flag1_multi14.0000_len1529	W1-3	1529	XRL22828.1 DNA-directed RNA polymerase subunit beta [Siniperca chuatsi ranavirus]	99.2	385	7.50E-267	69
k141_83252_flag1_multi1833.0000_len9377	W1-1	9377	QJE49149.1 putative immediate early protein ICP-46 [Largemouth bass virus]	100	382	2.98E-266	49513.48
k141_58798_flag1_multi32.7929_len5970	W5-3	5970	UUY86194.1 hypothetical protein [Largemouth bass virus]	100	404	3.64E-266	691
k141_57058_flag0_multi12.7320_len1126	W1-3	1126	XRL22821.1 hypothetical protein [Siniperca chuatsi ranavirus]	100	374	6.71E-266	32
k141_150395_flag1_multi31.9936_len6057	W5-2	6057	UUY86194.1 hypothetical protein [Largemouth bass virus]	100	404	6.88E-266	679
k141_110311_flag1_multi48.0000_len1105	W4-3	1105	AYV88121.1 hypothetical protein [Mandarin fish ranavirus]	98.9	361	1.26E-265	190
k141_105077_flag0_multi681.0766_len3341	W4-2	3341	AYV88121.1 hypothetical protein [Mandarin fish ranavirus]	98.7	374	9.27E-265	7376
k141_24471_flag1_multi224.7477_len2757	W2-3	2757	UUY86210.1 putative RNAseIII [Largemouth bass virus]	99.7	385	1.25E-264	2033
k141_78623_flag0_multi3166.1765_len2878	W1-1	2878	UUY86210.1 putative RNAseIII [Largemouth bass virus]	99.7	385	3.49E-264	21543
k141_119494_flag1_multi68.0000_len1066	W4-3	1066	UVF58786.1 MAG: 3-beta-hydroxy-delta-5-C27 steroid oxidoreductase, partial [Halichoeres melanurus ranavirus]	99.7	353	6.70E-263	248
k141_49418_flag1_multi1928.9751_len7203	W2-1	7203	UUY86194.1 hypothetical protein [Largemouth bass virus]	100	404	1.23E-262	50402
k141_829_flag1_multi118.5173_len1357	W6-1	1357	AYV88122.1 putative 3-beta-hydroxy-delta-5-C27 steroid oxidoreductase-like protein [Mandarin fish ranavirus]	100	354	1.62E-262	689
k141_85950_flag1_multi35.0000_len1456	W6-2	1456	AYV88122.1 putative 3-beta-hydroxy-delta-5-C27 steroid oxidoreductase-like protein [Mandarin fish ranavirus]	100	354	2.06E-262	167
k141_223599_flag1_multi15.0000_len1708	W1-3	1708	QJE49113.1 hypothetical protein LMBV_050 [Largemouth bass virus]	99.7	353	4.73E-261	93
k141_109392_flag1_multi18.0000_len1230	W5-2	1230	AYV88122.1 putative 3-beta-hydroxy-delta-5-C27 steroid oxidoreductase-like protein [Mandarin fish ranavirus]	100	349	1.34E-259	68
k141_89383_flag1_multi18.0000_len1050	W1-3	1050	AYV88122.1 putative 3-beta-hydroxy-delta-5-C27 steroid oxidoreductase-like protein [Mandarin fish ranavirus]	99.7	349	1.40E-259	58
k141_34166_flag1_multi26.0000_len1953	W1-2	1953	UUY86210.1 putative RNAseIII [Largemouth bass virus]	99.5	373	2.44E-259	178
k141_22634_flag1_multi7.0000_len1076	W3-1	1076	UUY86223.1 hypothetical protein [Largemouth bass virus]	99.4	358	2.46E-259	31
k141_89704_flag0_multi4129.2364_len4641	W1-1	4641	AYV88121.1 hypothetical protein [Mandarin fish ranavirus]	98.7	374	2.72E-259	83528
k141_159369_flag1_multi55.0000_len1949	W4-3	1949	UUY86210.1 putative RNAseIII [Largemouth bass virus]	99.7	372	4.74E-259	369
k141_82687_flag1_multi128.2945_len4932	W6-1	4932	UUY86210.1 putative RNAseIII [Largemouth bass virus]	99.7	385	2.46E-255	2540.65
k141_91552_flag1_multi4.0000_len1491	W3-2	1491	UUY86269.1 hypothetical protein [Largemouth bass virus]	98.8	409	6.12E-255	24
k141_27183_flag1_multi6.0000_len1069	W3-1	1069	UVF58793.1 MAG: DNA polymerase [Halichoeres melanurus ranavirus]	100	350	6.71E-254	19
k141_35943_flag0_multi82.8514_len9469	W2-1	9469	AYV88173.2 putative ribonucleotide reductase beta subunit [Mandarin fish ranavirus]	100	379	1.24E-252	6172.86
k141_10750_flag1_multi11.0000_len1023	W3-1	1023	AYV88122.1 putative 3-beta-hydroxy-delta-5-C27 steroid oxidoreductase-like protein [Mandarin fish ranavirus]	100	340	2.87E-252	33
k141_35002_flag0_multi153.8863_len13594	W6-1	13594	QYU76034.1 putative myristylated membrane protein, partial [Koi ranavirus]	100	400	4.19E-252	7264
k141_257811_flag1_multi3.0000_len1043	W6-3	1043	AYV88121.1 hypothetical protein [Mandarin fish ranavirus]	98.8	342	1.34E-250	12
k141_12802_flag1_multi43.0000_len1971	W4-2	1971	QJE49123.1 putative DNA repair protein RAD2 [Largemouth bass virus]	99.4	353	6.35E-249	211

k141_31135_flag0_multi41.0000_len1827	W3-1	1827	QJE49113.1 hypothetical protein LMBV_050 [Largemouth bass virus]	99.1	341	6.31E-248	71
k141_258307_flag1_multi14.0000_len1491	W5-3	1491	QJE49137.1 hypothetical protein LMBV_074 [Largemouth bass virus]	99.4	354	7.65E-248	61
k141_99651_flag0_multi908.7785_len1202	W6-2	1202	UVF58785.1 MAG: major capsid protein [Halichoeres melanurus ranavirus]	100	350	2.92E-246	3387
k141_106459_flag0_multi124.8789_len2594	W4-2	2594	QJE49122.1 putative DNA-dependent RNA polymerase b subunit [Largemouth bass virus]	100	349	9.06E-246	1587
k141_66854_flag1_multi9.0000_len1005	W5-2	1005	QJE49109.1 hypothetical protein LMBV_046 [Largemouth bass virus]	100	324	3.05E-245	25
k141_184146_flag1_multi33.0000_len1059	W4-3	1059	QJE49109.1 hypothetical protein LMBV_046 [Largemouth bass virus]	100	324	3.26E-245	122
k141_53546_flag1_multi44.0000_len1024	W6-2	1024	QJE49109.1 hypothetical protein LMBV_046 [Largemouth bass virus]	100	324	3.90E-245	168
k141_166544_flag1_multi59.0000_len3192	W2-3	3192	QJE49123.1 putative DNA repair protein RAD2 [Largemouth bass virus]	99.4	352	3.51E-242	601
k141_56684_flag0_multi5668.0000_len1740	W2-1	1740	QJE49109.1 hypothetical protein LMBV_046 [Largemouth bass virus]	100	324	2.10E-241	44358
k141_122318_flag0_multi6207.6862_len1846	W1-1	1846	QJE49109.1 hypothetical protein LMBV_046 [Largemouth bass virus]	100	324	1.54E-240	101503
k141_168238_flag0_multi583.2094_len3542	W2-3	3542	QYU76034.1 putative myristylated membrane protein, partial [Koi ranavirus]	100	357	3.47E-240	8445
k141_154539_flag1_multi84.0000_len1785	W5-3	1785	QJE49109.1 hypothetical protein LMBV_046 [Largemouth bass virus]	100	318	4.44E-235	512
k141_112307_flag1_multi436.1117_len6451	W1-1	6451	QJE49123.1 putative DNA repair protein RAD2 [Largemouth bass virus]	99.2	354	6.16E-232	8880
k141_102627_flag1_multi274.4999_len5620	W2-1	5620	AYV88122.1 putative 3-beta-hydroxy-delta-5-C27 steroid oxidoreductase-like protein [Mandarin fish ranavirus]	100	336	1.40E-230	5970
k141_19190_flag1_multi4.0000_len976	W3-1	976	QJE49102.1 putative DNA dependent RNA polymerase a subunit [Largemouth bass virus]	99.4	325	6.40E-228	15
k141_137607_flag1_multi7.0000_len995	W5-2	995	AYV88134.2 putative tyrosine kinase [Mandarin fish ranavirus]	100	312	5.55E-227	23
k141_141923_flag1_multi7.0000_len1407	W5-3	1407	QJE49086.1 hypothetical protein LMBV_023 [Largemouth bass virus]	99.7	313	3.86E-226	26
k141_78591_flag1_multi11.0000_len1428	W1-3	1428	QJE49110.1 hypothetical protein LMBV_047 [Largemouth bass virus]	99.4	320	1.29E-222	55
k141_23331_flag1_multi6.0000_len1020	W3-1	1020	AYV88179.2 putative DNA dependent RNA polymerase II second largest subunit [Mandarin fish ranavirus]	99.4	317	3.31E-222	20
k141_33607_flag0_multi302.2347_len5957	W2-1	5957	QJE49123.1 putative DNA repair protein RAD2 [Largemouth bass virus]	99.4	336	4.48E-220	2233
k141_121424_flag1_multi9.6115_len1114	W6-2	1114	QJE49123.1 putative DNA repair protein RAD2 [Largemouth bass virus]	97.5	314	5.34E-220	39
k141_109491_flag0_multi4415.5043_len1180	W2-1	1180	AYV88121.1 hypothetical protein [Mandarin fish ranavirus]	99	303	8.31E-220	2087
k141_89885_flag1_multi13.0000_len899	W1-3	899	QJE49109.1 hypothetical protein LMBV_046 [Largemouth bass virus]	100	294	8.66E-220	41
k141_20370_flag1_multi5.0000_len930	W5-3	930	QJE49122.1 putative DNA-dependent RNA polymerase b subunit [Largemouth bass virus]	100	309	1.00E-219	14
k141_85822_flag1_multi7.0000_len951	W1-2	951	AYV88179.2 putative DNA dependent RNA polymerase II second largest subunit [Mandarin fish ranavirus]	99.7	312	1.33E-219	18
k141_41740_flag1_multi23.0000_len1866	W1-3	1866	WEI29033.1 hypothetical protein [Largemouth bass virus]	97.6	495	2.29E-216	169
k141_100681_flag1_multi17.0000_len1071	W1-3	1071	UVF58788.1 MAG: helicase, partial [Halichoeres melanurus ranavirus]	100	305	4.50E-215	69
k141_21218_flag0_multi148.0000_len1025	W6-1	1025	WAK75092.1 putative D5 family NTPase/ATPase [Mandarin fish ranavirus]	98.3	297	4.87E-212	291
k141_124415_flag1_multi41.0000_len1865	W1-2	1865	UUY86193.1 hypothetical protein [Largemouth bass virus]	100	290	1.69E-211	253
k141_73519_flag1_multi3.0000_len971	W5-3	971	AYV88179.2 putative DNA dependent RNA polymerase II second largest subunit [Mandarin fish ranavirus]	100	299	2.38E-208	10
k141_89944_flag1_multi211.0000_len1999	W4-2	1999	WHA35577.1 putative ABC-ATPase [Micropterus salmoides ranavirus]	97.7	300	3.24E-207	1471
k141_4641_flag1_multi17.0000_len1805	W3-1	1805	UUY86193.1 hypothetical protein [Largemouth bass virus]	100	281	5.67E-205	92
k141_54207_flag1_multi7.0000_len852	W1-2	852	QJE49123.1 putative DNA repair protein RAD2 [Largemouth bass virus]	99.6	283	4.25E-203	19

k141_66094_flag1_multi74.0000_len1732	W4-3	1732	QJE49097.1 hypothetical protein LMBV_034 [Largemouth bass virus]	100	297	1.95E-201	457
k141_73874_flag1_multi34.0000_len1718	W6-2	1718	QJE49097.1 hypothetical protein LMBV_034 [Largemouth bass virus]	100	297	2.31E-201	200
k141_107520_flag1_multi116.0000_len1731	W2-2	1731	QJE49097.1 hypothetical protein LMBV_034 [Largemouth bass virus]	100	297	2.67E-201	692
k141_148463_flag1_multi43.0000_len1971	W1-2	1971	UUY86263.1 hypothetical protein [Largemouth bass virus]	97.6	292	4.79E-201	296
k141_22343_flag1_multi25.0000_len1968	W1-3	1968	UUY86263.1 hypothetical protein [Largemouth bass virus]	97.6	292	4.79E-201	187
k141_93444_flag1_multi3915.0000_len4338	W4-1	4338	UUY86193.1 hypothetical protein [Largemouth bass virus]	100	290	6.17E-201	58875.14
k141_212791_flag1_multi15.0000_len1545	W1-3	1545	UUY86192.1 putative myristylated membrane protein [Largemouth bass virus]	99.6	269	9.77E-201	84
k141_152258_flag1_multi38.1159_len1728	W6-2	1728	UUY86192.1 putative myristylated membrane protein [Largemouth bass virus]	99.6	270	1.07E-200	243
k141_29360_flag1_multi15.0000_len1923	W3-1	1923	UUY86263.1 hypothetical protein [Largemouth bass virus]	97.6	291	1.63E-200	95
k141_29722_flag1_multi210.0507_len1898	W6-1	1898	QJE49097.1 hypothetical protein LMBV_034 [Largemouth bass virus]	100	297	1.95E-200	1400.17
k141_111157_flag1_multi96.8236_len1955	W4-2	1955	QJE49097.1 hypothetical protein LMBV_034 [Largemouth bass virus]	100	297	3.80E-200	661
k141_150271_flag1_multi54.9155_len2200	W6-2	2200	UUY86263.1 hypothetical protein [Largemouth bass virus]	97.6	292	6.65E-200	438
k141_28312_flag1_multi10.0000_len1608	W3-1	1608	QJE49097.1 hypothetical protein LMBV_034 [Largemouth bass virus]	100	295	8.39E-200	48
k141_39200_flag1_multi5.0000_len1254	W5-2	1254	AYV88131.1 putative eIF-2 alpha-like protein [Mandarin fish ranavirus]	99.6	270	1.95E-199	16
k141_15001_flag1_multi16.0000_len1667	W5-3	1667	QJE49097.1 hypothetical protein LMBV_034 [Largemouth bass virus]	100	294	2.75E-198	88
k141_27532_flag0_multi69.0000_len1484	W5-3	1484	QYU76034.1 putative myristylated membrane protein, partial [Koi ranavirus]	100	273	1.08E-196	331
k141_11746_flag1_multi16.0000_len1738	W1-2	1738	QJE49097.1 hypothetical protein LMBV_034 [Largemouth bass virus]	100	291	1.47E-196	106
k141_250457_flag1_multi20.5879_len4446	W5-3	4446	WHA35577.1 putative ABC-ATPase [Micropterus salmoides ranavirus]	97.7	299	1.80E-196	295
k141_77103_flag0_multi662.3691_len2747	W2-1	2747	QJE49097.1 hypothetical protein LMBV_034 [Largemouth bass virus]	100	297	2.14E-196	6638
k141_108012_flag1_multi7.0000_len1181	W5-3	1181	QJE49141.1 putative NTPase [Largemouth bass virus]	98.9	279	8.47E-195	23
k141_101067_flag1_multi29.0000_len1951	W5-2	1951	UUY86263.1 hypothetical protein [Largemouth bass virus]	97.5	283	1.30E-194	198
k141_122121_flag1_multi139.0000_len1944	W4-3	1944	UUY86263.1 hypothetical protein [Largemouth bass virus]	97.5	282	4.89E-194	982
k141_135793_flag0_multi310.8017_len2294	W2-3	2294	UUY86263.1 hypothetical protein [Largemouth bass virus]	97.2	283	9.74E-192	2466
k141_32698_flag1_multi18.0000_len1930	W3-2	1930	AYV88194.1 hypothetical protein [Mandarin fish ranavirus]	99.6	281	9.99E-192	127
k141_116161_flag1_multi296.4162_len2217	W6-1	2217	UUY86263.1 hypothetical protein [Largemouth bass virus]	97.2	281	3.35E-191	2271
k141_158938_flag0_multi495.7268_len983	W2-3	983	WHA35533.1 putative DNA dependent RNA polymerase A subunit [Micropterus salmoides ranavirus]	100	277	9.34E-190	94
k141_184467_flag1_multi4.0000_len1197	W6-3	1197	WAK75070.1 putative neurofilament triplet H1-like protein [Mandarin fish ranavirus]	100	398	1.43E-185	14
k141_258402_flag1_multi16.0000_len1160	W5-3	1160	XRL22828.1 DNA-directed RNA polymerase subunit beta [Siniperca chuatsi ranavirus]	98.9	281	1.89E-185	57
k141_223620_flag1_multi6.0000_len820	W5-3	820	QJE49123.1 putative DNA repair protein RAD2 [Largemouth bass virus]	99.6	253	2.73E-180	14
k141_27788_flag1_multi15.0000_len1296	W5-2	1296	QJE49098.1 hypothetical protein LMBV_035 [Largemouth bass virus]	99.2	250	3.12E-179	67
k141_162408_flag1_multi5.0000_len750	W5-2	750	QJE49123.1 putative DNA repair protein RAD2 [Largemouth bass virus]	99.6	249	1.70E-177	7
k141_22533_flag1_multi19.0000_len1287	W3-1	1287	AYV88176.1 putative tumor necrosis factor receptor [Mandarin fish ranavirus]	100	237	4.21E-177	80
k141_3833_flag1_multi4.0000_len751	W3-1	751	XPZ21295.1 putative RNAseIII [Mandarin fish ranavirus]	100	249	3.26E-176	9
k141_63892_flag1_multi26.0000_len1460	W5-2	1460	AYV88176.1 putative tumor necrosis factor receptor [Mandarin fish ranavirus]	100	237	5.09E-176	161
k141_8377_flag1_multi28.0000_len1441	W1-3	1441	AYV88176.1 putative tumor necrosis factor receptor [Mandarin fish ranavirus]	100	237	8.24E-176	142
k141_113380_flag1_multi39.9125_len1478	W3-2	1478	AYV88176.1 putative tumor necrosis factor receptor [Mandarin fish ranavirus]	100	237	8.98E-176	205
k141_12772_flag1_multi30.4192_len2004	W6-2	2004	UUY86210.1 putative RNAseIII [Largemouth bass virus]	100	258	1.40E-174	204
k141_213726_flag1_multi29.0000_len1377	W5-3	1377	AYV88176.1 putative tumor necrosis factor receptor [Mandarin fish ranavirus]	100	234	1.73E-174	132

k141_12973_flag0_multi9.8718_len1685	W3-1	1685	QJE49141.1 putative NTPase [Largemouth bass virus]	98.8	253	2.03E-172	58
k141_81191_flag0_multi16.0000_len1208	W5-3	1208	WAK75136.1 hypothetical protein [Mandarin fish ranavirus]	100	243	2.89E-172	60
k141_90160_flag1_multi9.0000_len1170	W1-3	1170	WAK75136.1 hypothetical protein [Mandarin fish ranavirus]	100	243	7.15E-172	30
k141_32708_flag1_multi43.0000_len1290	W6-2	1290	WAK75136.1 hypothetical protein [Mandarin fish ranavirus]	100	243	7.97E-172	186
k141_157714_flag1_multi139.0000_len896	W5-2	896	QJE49073.1 putative p31K protein [Largemouth bass virus]	99.2	262	8.24E-171	387
k141_35782_flag1_multi10.7099_len1675	W3-1	1675	WAK75136.1 hypothetical protein [Mandarin fish ranavirus]	100	243	8.16E-170	65
k141_140860_flag1_multi7.0000_len742	W5-2	742	AYV88179.2 putative DNA dependent RNA polymerase II second largest subunit [Mandarin fish ranavirus]	99.2	247	3.09E-169	16
k141_8215_flag1_multi208.0368_len1038	W3-2	1038	XRB52768.1 P31K protein, partial [Largemouth bass virus]	98	246	4.49E-169	801
k141_213102_flag1_multi98.0000_len881	W5-3	881	QJE49073.1 putative p31K protein [Largemouth bass virus]	99.2	242	6.46E-169	269
k141_253_flag1_multi11.0000_len1204	W1-3	1204	QJE49098.1 hypothetical protein LMBV_035 [Largemouth bass virus]	98.7	235	2.51E-168	42
k141_88962_flag0_multi69.0000_len1056	W5-3	1056	UUY86204.1 hypothetical protein [Largemouth bass virus]	100	274	2.94E-167	167
k141_154919_flag1_multi18.3374_len2278	W5-2	2278	WAK75136.1 hypothetical protein [Mandarin fish ranavirus]	100	243	6.37E-167	150
k141_127817_flag1_multi321.0000_len988	W6-2	988	XRB52768.1 P31K protein, partial [Largemouth bass virus]	96.3	246	9.00E-167	1159
k141_75134_flag1_multi51.0000_len869	W4-3	869	UUY86216.1 hypothetical protein [Largemouth bass virus]	99.6	248	9.07E-167	158
k141_8920_flag0_multi142.8769_len1701	W1-2	1701	QJE49073.1 putative p31K protein [Largemouth bass virus]	99.2	262	1.25E-166	879.17
k141_56578_flag1_multi28.6446_len1016	W5-2	1016	UUY86216.1 hypothetical protein [Largemouth bass virus]	99.6	248	6.20E-166	105
k141_127394_flag1_multi622.5455_len977	W4-3	977	AYV88120.1 putative p31K protein [Mandarin fish ranavirus]	97.9	241	6.99E-165	2402
k141_107697_flag1_multi102.6397_len1007	W6-2	1007	UUY86216.1 hypothetical protein [Largemouth bass virus]	99.2	248	1.84E-164	330
k141_15534_flag1_multi39.0000_len1009	W3-1	1009	UUY86216.1 hypothetical protein [Largemouth bass virus]	99.2	248	1.91E-164	41
k141_53718_flag1_multi2.0000_len719	W6-3	719	XPZ21261.1 hypothetical protein MRVORF002 [Mandarin fish ranavirus]	99.6	239	2.77E-164	3
k141_129963_flag0_multi1505.4252_len1411	W1-1	1411	UUY86216.1 hypothetical protein [Largemouth bass virus]	99.6	248	2.94E-164	12239
k141_32404_flag1_multi15.0000_len1296	W4-2	1296	ADB77862.1 DNA methyltransferase [Largemouth bass ulcerative syndrome virus]	99.5	220	3.17E-164	63
k141_104114_flag0_multi322.1731_len1198	W6-1	1198	UUY86216.1 hypothetical protein [Largemouth bass virus]	99.6	247	1.01E-163	1759
k141_35669_flag1_multi6.0000_len726	W3-1	726	AYV88179.2 putative DNA dependent RNA polymerase II second largest subunit [Mandarin fish ranavirus]	100	241	8.83E-163	12
k141_71093_flag1_multi588.2269_len1697	W2-3	1697	UUY86216.1 hypothetical protein [Largemouth bass virus]	99.6	248	1.75E-162	3781.33
k141_43277_flag1_multi6.0000_len800	W3-2	800	QJE49083.1 hypothetical protein LMBV_020 [Largemouth bass virus]	99.2	244	1.80E-162	15
k141_13407_flag1_multi39.0000_len1361	W1-2	1361	UUY86216.1 hypothetical protein [Largemouth bass virus]	99.2	246	4.44E-162	220
k141_34592_flag1_multi428.9965_len2407	W3-1	2407	XRB52768.1 P31K protein, partial [Largemouth bass virus]	98.4	245	5.77E-162	587
k141_91010_flag1_multi98.0000_len835	W1-3	835	QJE49073.1 putative p31K protein [Largemouth bass virus]	99.6	250	2.02E-161	227
k141_103027_flag1_multi10.0000_len659	W5-2	659	UUY86238.1 hypothetical protein [Largemouth bass virus]	99.5	219	4.15E-161	22
k141_97128_flag1_multi16258.1688_len3305	W4-1	3305	UUY86197.1 hypothetical protein [Largemouth bass virus]	100	331	4.18E-161	181883.17
k141_107474_flag1_multi1004.3072_len1902	W2-1	1902	UUY86216.1 hypothetical protein [Largemouth bass virus]	99.6	248	5.09E-161	6841
k141_45526_flag1_multi85.0000_len1952	W4-3	1952	ADB77862.1 DNA methyltransferase [Largemouth bass ulcerative syndrome virus]	99.5	220	6.22E-161	564
k141_70128_flag1_multi21.2250_len6302	W3-2	6302	QJE49123.1 putative DNA repair protein RAD2 [Largemouth bass virus]	92.7	273	1.61E-160	301
k141_12543_flag1_multi12.0000_len1371	W3-1	1371	AYV88210.2 putative proliferating cell nuclear antigen [Mandarin fish ranavirus]	100	240	5.34E-160	53
k141_168285_flag1_multi16.0000_len1347	W5-3	1347	AYV88210.2 putative proliferating cell nuclear antigen [Mandarin fish ranavirus]	100	241	8.03E-160	68
k141_15188_flag1_multi20.0000_len1359	W1-3	1359	AYV88210.2 putative proliferating cell nuclear antigen [Mandarin fish ranavirus]	100	240	9.29E-160	98
k141_115771_flag1_multi66.0000_len1361	W4-3	1361	AYV88210.2 putative proliferating cell nuclear antigen [Mandarin fish ranavirus]	100	240	9.64E-160	319

k141_79051_flag1_multi24.0000_len1360	W5-2	1360	AYV88210.2 putative proliferating cell nuclear antigen [Mandarin fish ranavirus]	100	240	9.64E-160	117
k141_70625_flag1_multi980.0000_len1517	W2-1	1517	AYV88210.2 putative proliferating cell nuclear antigen [Mandarin fish ranavirus]	100	242	2.17E-159	5265
k141_95025_flag1_multi473.4332_len1600	W2-3	1600	AYV88210.2 putative proliferating cell nuclear antigen [Mandarin fish ranavirus]	100	242	5.79E-159	2535
k141_7594_flag1_multi6.0000_len1229	W3-1	1229	WHA35533.1 putative DNA dependent RNA polymerase A subunit [Micropterus salmoides ranavirus]	93.5	260	7.33E-159	17
k141_166887_flag1_multi3.0000_len706	W5-3	706	WHA35533.1 putative DNA dependent RNA polymerase A subunit [Micropterus salmoides ranavirus]	100	234	7.71E-158	7
k141_85479_flag1_multi30.0000_len1400	W3-2	1400	AYV88210.2 putative proliferating cell nuclear antigen [Mandarin fish ranavirus]	100	239	1.03E-157	141
k141_80469_flag1_multi243.0000_len2992	W6-1	2992	ADB77862.1 DNA methyltransferase [Largemouth bass ulcerative syndrome virus]	99.5	220	1.63E-156	2032
k141_4095_flag1_multi10.0000_len705	W1-3	705	QJE49083.1 hypothetical protein LMBV_020 [Largemouth bass virus]	99.6	234	3.40E-156	22
k141_4696_flag1_multi6078.0000_len5033	W1-1	5033	UUY86197.1 hypothetical protein [Largemouth bass virus]	100	331	3.55E-156	93127.92
k141_64825_flag1_multi233.2356_len1703	W2-2	1703	AYV88210.2 putative proliferating cell nuclear antigen [Mandarin fish ranavirus]	100	237	4.03E-155	1548
k141_29827_flag1_multi1573.1261_len2402	W1-1	2402	AYV88210.2 putative proliferating cell nuclear antigen [Mandarin fish ranavirus]	99.6	242	6.15E-155	14045.63
k141_50685_flag1_multi98.0000_len1209	W1-3	1209	WXI69548.1 hypothetical protein [Largemouth bass virus]	99.6	224	1.47E-154	459
k141_199569_flag1_multi510.6626_len1217	W4-3	1217	WXI69548.1 hypothetical protein [Largemouth bass virus]	99.6	224	1.59E-154	2358
k141_40951_flag1_multi369.1714_len3151	W4-2	3151	UUY86216.1 hypothetical protein [Largemouth bass virus]	99.6	246	2.72E-154	1354
k141_254628_flag1_multi9.0000_len1028	W6-3	1028	WXI69548.1 hypothetical protein [Largemouth bass virus]	99.5	222	4.93E-154	31
k141_37707_flag1_multi7825.0000_len4325	W2-1	4325	QJE49073.1 putative p31K protein [Largemouth bass virus]	99.2	259	5.47E-154	120377
k141_133264_flag1_multi14.0000_len629	W1-3	629	AYV88168.1 hypothetical protein [Mandarin fish ranavirus]	99	207	1.04E-153	31
k141_19935_flag1_multi81.1913_len1343	W3-1	1343	WXI69548.1 hypothetical protein [Largemouth bass virus]	99.6	223	4.24E-153	359
k141_89183_flag0_multi12.6292_len853	W5-2	853	AYV88168.1 hypothetical protein [Mandarin fish ranavirus]	99	207	2.90E-152	28
k141_32475_flag1_multi50.1649_len2142	W5-2	2142	WXI69548.1 hypothetical protein [Largemouth bass virus]	99.6	226	2.92E-152	370
k141_63690_flag1_multi373.9203_len1183	W6-2	1183	AYV88159.1 hypothetical protein [Mandarin fish ranavirus]	100	214	5.49E-152	1643
k141_104197_flag1_multi10.0000_len1328	W5-3	1328	AYV88142.2 hypothetical protein [Mandarin fish ranavirus]	100	223	6.04E-151	39
k141_208011_flag1_multi70.0000_len1858	W1-3	1858	AYV88159.1 hypothetical protein [Mandarin fish ranavirus]	100	216	3.08E-150	467
k141_143040_flag1_multi43.0000_len1446	W1-2	1446	AYV88210.2 putative proliferating cell nuclear antigen [Mandarin fish ranavirus]	99.6	229	4.83E-150	217
k141_23750_flag1_multi181.0000_len1968	W1-2	1968	AYV88159.1 hypothetical protein [Mandarin fish ranavirus]	100	216	6.92E-150	1318
k141_40919_flag1_multi398.0000_len1957	W4-3	1957	AYV88159.1 hypothetical protein [Mandarin fish ranavirus]	100	216	8.90E-150	2813
k141_122964_flag1_multi9.9857_len772	W6-3	772	AYV88159.1 hypothetical protein [Mandarin fish ranavirus]	100	208	2.19E-149	28
k141_97747_flag0_multi289.7093_len1135	W6-1	1135	WEI29033.1 hypothetical protein [Largemouth bass virus]	100	304	2.70E-149	859
k141_44525_flag1_multi20.0000_len627	W5-3	627	AYV88168.1 hypothetical protein [Mandarin fish ranavirus]	99	202	2.76E-149	42
k141_3861_flag1_multi11.0000_len616	W3-1	616	AYV88168.1 hypothetical protein [Mandarin fish ranavirus]	99	200	1.59E-147	20
k141_102487_flag0_multi80.2700_len2552	W5-2	2552	AYV88159.1 hypothetical protein [Mandarin fish ranavirus]	100	216	3.26E-147	696
k141_144146_flag1_multi8.0000_len949	W6-3	949	AYV88176.1 putative tumor necrosis factor receptor [Mandarin fish ranavirus]	100	192	7.79E-147	23
k141_31734_flag1_multi7.9872_len688	W3-1	688	UUY86269.1 hypothetical protein [Largemouth bass virus]	98.7	229	1.40E-145	15
k141_114355_flag1_multi107.1101_len1295	W5-3	1295	QJE49100.1 hypothetical protein LMBV_037 [Largemouth bass virus]	99.5	212	1.74E-145	475
k141_107414_flag0_multi50.0000_len1230	W3-2	1230	AYV88168.1 hypothetical protein [Mandarin fish ranavirus]	99	201	7.33E-145	198
k141_57966_flag1_multi6.0000_len628	W6-3	628	QJE49149.1 putative immediate early protein ICP-46 [Largemouth bass virus]	100	200	8.52E-145	11
k141_50210_flag1_multi18.0000_len703	W1-3	703	AYV88121.1 hypothetical protein [Mandarin fish ranavirus]	98.6	208	5.30E-144	47
k141_176343_flag1_multi4.0000_len653	W5-3	653	UVF58793.1 MAG: DNA polymerase [Halichoeres melanurus ranavirus]	100	217	6.63E-144	6

k141_23025_flag1_multi5.0000_len668	W1-3	668	QJE49137.1 hypothetical protein LMBV_074 [Largemouth bass virus]	98.2	222	1.81E-143	13
k141_5299_flag1_multi33.0000_len615	W6-2	615	QJE49080.1 hypothetical protein LMBV_017 [Largemouth bass virus]	98.5	196	1.07E-142	75
k141_123234_flag1_multi10.0000_len607	W5-2	607	QJE49080.1 hypothetical protein LMBV_017 [Largemouth bass virus]	98.5	196	1.40E-142	21
k141_230486_flag1_multi6.0000_len592	W5-3	592	QJE49080.1 hypothetical protein LMBV_017 [Largemouth bass virus]	98.5	195	2.30E-142	11
k141_219393_flag1_multi12.0000_len589	W1-3	589	UUY86238.1 hypothetical protein [Largemouth bass virus]	100	196	2.40E-142	24
k141_120901_flag1_multi19.0000_len610	W1-2	610	QJE49080.1 hypothetical protein LMBV_017 [Largemouth bass virus]	98.5	195	2.94E-142	37
k141_78985_flag1_multi61.0000_len702	W4-2	702	QJE49080.1 hypothetical protein LMBV_017 [Largemouth bass virus]	98.5	196	4.90E-142	142
k141_15583_flag1_multi6.0000_len624	W3-1	624	QJE49080.1 hypothetical protein LMBV_017 [Largemouth bass virus]	98.5	196	5.11E-142	13
k141_21554_flag1_multi37.0000_len611	W3-2	611	QJE49080.1 hypothetical protein LMBV_017 [Largemouth bass virus]	98.5	195	5.92E-142	82
k141_193202_flag1_multi26.0000_len645	W4-3	645	QJE49080.1 hypothetical protein LMBV_017 [Largemouth bass virus]	98.5	196	6.79E-142	60
k141_174785_flag1_multi771.0000_len4210	W2-3	4210	AYV88159.1 hypothetical protein [Mandarin fish ranavirus]	100	216	9.51E-142	12132
k141_215866_flag1_multi12.0000_len586	W1-3	586	QJE49080.1 hypothetical protein LMBV_017 [Largemouth bass virus]	98.4	193	2.47E-141	25
k141_102353_flag1_multi7.0000_len582	W3-2	582	UUY86238.1 hypothetical protein [Largemouth bass virus]	100	193	4.06E-140	12
k141_34231_flag1_multi7.0000_len588	W6-3	588	QJE49080.1 hypothetical protein LMBV_017 [Largemouth bass virus]	98.4	191	1.18E-139	13
k141_225593_flag1_multi10.0000_len623	W5-3	623	UUY86210.1 putative RNAseIII [Largemouth bass virus]	100	200	3.83E-139	22
k141_21315_flag1_multi6.8669_len1178	W3-1	1178	AYV88134.2 putative tyrosine kinase [Mandarin fish ranavirus]	100	201	1.99E-138	22
k141_33597_flag1_multi15.0000_len621	W1-3	621	UUY86210.1 putative RNAseIII [Largemouth bass virus]	99.5	200	4.27E-138	27
k141_60077_flag1_multi32.0000_len3887	W6-2	3887	AYV88210.2 putative proliferating cell nuclear antigen [Mandarin fish ranavirus]	99.6	224	8.95E-138	388
k141_112616_flag1_multi453.8178_len849	W2-1	849	QJE49080.1 hypothetical protein LMBV_017 [Largemouth bass virus]	97.4	189	2.53E-134	1809
k141_237792_flag1_multi6.0000_len803	W6-3	803	UUY86261.1 hypothetical protein [Largemouth bass virus]	100	212	1.12E-133	13
k141_146178_flag1_multi18.0000_len1418	W5-2	1418	UUY86210.1 putative RNAseIII [Largemouth bass virus]	99.5	200	1.36E-133	87
k141_98541_flag1_multi6.0000_len647	W6-3	647	WAK75136.1 hypothetical protein [Mandarin fish ranavirus]	100	189	7.22E-133	11
k141_44182_flag1_multi5.0000_len1168	W5-3	1168	QJE49083.1 hypothetical protein LMBV_020 [Largemouth bass virus]	99.5	205	1.13E-132	21
k141_186552_flag1_multi4.0000_len603	W1-3	603	AYV88179.2 putative DNA dependent RNA polymerase II second largest subunit [Mandarin fish ranavirus]	99	200	2.32E-132	7
k141_94270_flag1_multi6.0000_len910	W1-2	910	WHA35507.1 hypothetical protein MSRaV_19R [Micropterus salmoides ranavirus]	98.9	187	1.77E-131	18
k141_23824_flag1_multi50.0000_len629	W1-3	629	AYV88125.2 hypothetical protein [Mandarin fish ranavirus]	97.9	190	2.98E-131	106
k141_131990_flag1_multi6.0000_len1104	W1-3	1104	QJE49140.1 putative ribonucleotide reductase alpha subunit [Largemouth bass virus]	99.5	194	4.06E-131	18
k141_129900_flag1_multi118.0000_len788	W1-2	788	AYV88125.2 hypothetical protein [Mandarin fish ranavirus]	97.4	191	4.24E-131	280
k141_15468_flag1_multi40.0000_len857	W3-1	857	AYV88125.2 hypothetical protein [Mandarin fish ranavirus]	97.4	191	2.09E-130	100
k141_224937_flag1_multi12.0000_len850	W6-3	850	WXI69518.1 hypothetical protein [Largemouth bass virus]	91.8	267	6.80E-130	39
k141_30454_flag1_multi99.6214_len1245	W3-2	1245	WEI28998.1 hypothetical protein [Largemouth bass virus]	93	284	3.65E-129	537
k141_30825_flag1_multi54.0000_len1271	W1-3	1271	WEI28998.1 hypothetical protein [Largemouth bass virus]	93	284	4.85E-129	289
k141_76549_flag1_multi9.0000_len646	W6-3	646	XRL22790.1 hypothetical protein [Siniperca chuatsi ranavirus]	99	192	5.46E-129	19
k141_25742_flag1_multi3.0000_len619	W6-3	619	UUY86236.1 hypothetical protein [Largemouth bass virus]	100	181	8.18E-129	4
k141_61635_flag1_multi30.0000_len1379	W5-3	1379	WEI28998.1 hypothetical protein [Largemouth bass virus]	93	284	2.42E-128	161
k141_62480_flag1_multi84.0000_len1393	W1-2	1393	WEI28998.1 hypothetical protein [Largemouth bass virus]	93	284	4.07E-128	467
k141_105001_flag0_multi345.1931_len1467	W4-2	1467	WEI28998.1 hypothetical protein [Largemouth bass virus]	93	284	4.59E-128	2018
k141_154408_flag1_multi59.3321_len1478	W6-2	1478	WEI28998.1 hypothetical protein [Largemouth bass virus]	93	284	5.25E-128	667
k141_150661_flag1_multi4.0000_len548	W5-3	548	AYV88131.1 putative eIF-2 alpha-like protein [Mandarin fish ranavirus]	99.5	182	3.68E-127	5

k141_19597_flag1_multi2.0000_len545	W6-3	545	UUY86263.1 hypothetical protein [Largemouth bass virus]	100	181	3.91E-127	4
k141_84233_flag1_multi456.0000_len1783	W6-1	1783	WEI28998.1 hypothetical protein [Largemouth bass virus]	93	284	1.40E-126	3488
k141_99491_flag0_multi13.0000_len803	W1-3	803	XRL22821.1 hypothetical protein [Siniperca chuatsi ranavirus]	100	186	1.42E-126	45
k141_232727_flag1_multi7.0000_len547	W4-3	547	AYV88131.1 putative eIF-2 alpha-like protein [Mandarin fish ranavirus]	100	181	1.43E-126	12
k141_116885_flag1_multi30.0000_len588	W1-3	588	UUY86216.1 hypothetical protein [Largemouth bass virus]	99.5	191	4.07E-125	65
k141_32673_flag1_multi5.0000_len610	W3-1	610	QJE49137.1 hypothetical protein LMBV_074 [Largemouth bass virus]	98.5	202	1.06E-124	9
k141_257294_flag1_multi3.0000_len572	W6-3	572	UUY86194.1 hypothetical protein [Largemouth bass virus]	100	190	1.40E-124	4
k141_56393_flag1_multi3.0000_len577	W6-3	577	UUY86258.1 hypothetical protein [Largemouth bass virus]	100	189	1.85E-124	4
k141_1780_flag1_multi760.0696_len658	W3-1	658	AYV88121.1 hypothetical protein [Mandarin fish ranavirus]	98.4	183	3.36E-124	7
k141_243336_flag1_multi18.0000_len542	W4-3	542	QJE49123.1 putative DNA repair protein RAD2 [Largemouth bass virus]	100	180	3.58E-123	34
k141_125706_flag1_multi13.0000_len589	W5-2	589	AYV88121.1 hypothetical protein [Mandarin fish ranavirus]	95.2	188	4.22E-123	22
k141_159_flag1_multi2974.6154_len3066	W4-1	3066	UUY86198.1 hypothetical protein [Largemouth bass virus]	100	213	2.41E-122	34542
k141_113272_flag1_multi14.0000_len562	W1-2	562	AYV88179.2 putative DNA dependent RNA polymerase II second largest subunit [Mandarin fish ranavirus]	98.9	186	1.47E-121	35
k141_43035_flag1_multi5.0000_len546	W5-3	546	AYV88179.2 putative DNA dependent RNA polymerase II second largest subunit [Mandarin fish ranavirus]	100	181	1.71E-120	9
k141_86911_flag1_multi28.0698_len1616	W5-2	1616	WEI28998.1 hypothetical protein [Largemouth bass virus]	92.8	276	4.27E-120	189
k141_50511_flag1_multi4.0000_len544	W6-3	544	UUY86267.1 ribonucleotide reductase alpha subunit [Largemouth bass virus]	100	180	7.14E-120	5
k141_189512_flag1_multi7.0000_len668	W1-3	668	AYV88133.1 putative DNA-directed RNA polymerase II subunit [Mandarin fish ranavirus]	100	159	1.47E-118	19
k141_79222_flag1_multi6.0000_len724	W5-2	724	AYV88133.1 putative DNA-directed RNA polymerase II subunit [Mandarin fish ranavirus]	100	159	3.11E-118	10
k141_162819_flag0_multi1.0000_len558	W5-1	558	AIG51690.1 major capsid protein, partial [Koi ranavirus]	94.6	186	3.49E-117	79.17
k141_134434_flag1_multi5.0000_len525	W1-3	525	QJE49123.1 putative DNA repair protein RAD2 [Largemouth bass virus]	100	174	1.36E-116	7
k141_31913_flag1_multi4.0000_len594	W3-1	594	QJE49083.1 hypothetical protein LMBV_020 [Largemouth bass virus]	99.4	178	1.96E-115	7
k141_230771_flag1_multi4.0000_len520	W6-3	520	QJE49145.1 putative proliferating cell nuclear antigen [Largemouth bass virus]	100	173	3.06E-115	7
k141_13295_flag1_multi7.0000_len501	W3-1	501	UUY86238.1 hypothetical protein [Largemouth bass virus]	98.2	167	3.36E-115	9
k141_46866_flag1_multi4.0000_len485	W3-3	485	QJE49149.1 putative immediate early protein ICP-46 [Largemouth bass virus]	100	161	6.22E-115	5
k141_53869_flag1_multi5.0000_len584	W6-3	584	AYV88168.1 hypothetical protein [Mandarin fish ranavirus]	98.1	159	7.05E-114	10
k141_147870_flag1_multi4.0000_len548	W5-3	548	QJE49137.1 hypothetical protein LMBV_074 [Largemouth bass virus]	98.4	182	2.05E-113	8
k141_81444_flag1_multi19.0000_len558	W6-2	558	AYV88136.2 hypothetical protein [Mandarin fish ranavirus]	98.7	159	6.20E-113	35
k141_188315_flag1_multi19.0000_len513	W4-3	513	AYV88136.2 hypothetical protein [Mandarin fish ranavirus]	98.7	159	6.54E-113	34
k141_9206_flag1_multi23.0301_len1006	W3-1	1006	WEI28998.1 hypothetical protein [Largemouth bass virus]	92.3	261	3.28E-112	94
k141_9694_flag1_multi4.0000_len486	W3-1	486	UVF58787.1 MAG: DNA-dependent RNA polymerase largest subunit, partial [Halichoeres melanurus ranavirus]	100	161	3.40E-112	5
k141_37889_flag1_multi56.5406_len707	W4-2	707	AYV88136.2 hypothetical protein [Mandarin fish ranavirus]	98.7	159	4.34E-112	140.69
k141_146417_flag1_multi8.0000_len520	W1-2	520	AYV88179.2 putative DNA dependent RNA polymerase II second largest subunit [Mandarin fish ranavirus]	99.4	172	1.37E-111	12
k141_6351_flag1_multi13.0000_len479	W1-2	479	AYV88136.2 hypothetical protein [Mandarin fish ranavirus]	98.7	157	1.40E-111	20
k141_67506_flag1_multi29.4862_len795	W6-1	795	AYV88136.2 hypothetical protein [Mandarin fish ranavirus]	98.7	159	2.68E-111	75

k141_31106_flag1_multi18.0000_len537	W3-2	537	AYV88136.2 hypothetical protein [Mandarin fish ranavirus]	98.7	151	6.38E-111	31
k141_36093_flag0_multi14513.5106_len1316	W1-1	1316	UVF58785.1 MAG: major capsid protein [Halichoeres melanurus ranavirus]	100	166	8.23E-111	204167
k141_217865_flag1_multi6.8142_len464	W5-3	464	QYU76035.1 DNA polymerase, partial [Koi ranavirus]	100	154	1.11E-110	9
k141_26331_flag1_multi36.0000_len967	W3-1	967	UUY86256.1 putative orf58-like protein [Largemouth bass virus]	99.1	225	1.55E-110	161
k141_121205_flag1_multi6.0000_len996	W5-3	996	AYV88136.2 hypothetical protein [Mandarin fish ranavirus]	98.7	159	2.37E-110	19
k141_100791_flag1_multi5.0000_len496	W1-3	496	QJE49122.1 putative DNA-dependent RNA polymerase b subunit [Largemouth bass virus]	100	164	5.54E-110	7
k141_151609_flag1_multi9.0000_len1924	W5-2	1924	UUY86218.1 hypothetical protein [Largemouth bass virus]	98.2	171	1.34E-109	46
k141_32021_flag1_multi12.0000_len892	W1-2	892	XRB52829.1 P8.141C-like protein, partial [Largemouth bass virus]	98.8	160	1.59E-109	33
k141_66652_flag0_multi358.0000_len2814	W2-3	2814	UUY86204.1 hypothetical protein [Largemouth bass virus]	100	193	9.61E-109	1413
k141_14272_flag1_multi4.0000_len481	W3-1	481	UUY86211.1 hypothetical protein [Largemouth bass virus]	98.7	159	3.09E-108	6
k141_79644_flag0_multi1662.1574_len2263	W1-1	2263	UUY86218.1 hypothetical protein [Largemouth bass virus]	98.8	170	5.41E-108	8476
k141_4619_flag0_multi372.9712_len905	W6-2	905	UVF58785.1 MAG: major capsid protein [Halichoeres melanurus ranavirus]	100	159	1.20E-107	872
k141_27804_flag1_multi61.1749_len1010	W5-3	1010	UUY86256.1 putative orf58-like protein [Largemouth bass virus]	92.1	242	1.43E-107	260
k141_116449_flag1_multi6.0000_len853	W5-2	853	QJE49140.1 putative ribonucleotide reductase alpha subunit [Largemouth bass virus]	100	160	1.56E-107	15
k141_115774_flag1_multi7.0000_len652	W1-3	652	UUY86221.1 hypothetical protein [Largemouth bass virus]	98.9	177	2.90E-107	15
k141_61173_flag1_multi90.0000_len1678	W6-2	1678	WXI69513.1 hypothetical protein [Largemouth bass virus]	97.9	187	3.12E-107	588
k141_91943_flag1_multi4.0000_len463	W3-2	463	AYV88130.2 putative ATPase-dependent protease [Mandarin fish ranavirus]	98.7	153	7.85E-107	6
k141_23771_flag1_multi7.0000_len468	W5-3	468	AYV88130.2 putative ATPase-dependent protease [Mandarin fish ranavirus]	98.7	153	8.53E-107	9
k141_64226_flag1_multi190.2107_len1793	W1-1	1793	AYV88136.2 hypothetical protein [Mandarin fish ranavirus]	98.7	159	9.44E-107	3238.19
k141_148965_flag0_multi44.2305_len1399	W1-3	1399	UUY86256.1 putative orf58-like protein [Largemouth bass virus]	97.8	223	1.06E-106	231
k141_11586_flag1_multi2.0000_len479	W3-1	479	AYV88173.2 putative ribonucleotide reductase beta subunit [Mandarin fish ranavirus]	100	159	2.12E-106	3
k141_2498_flag0_multi60.0922_len1595	W3-2	1595	UUY86256.1 putative orf58-like protein [Largemouth bass virus]	98.2	225	3.04E-106	411
k141_22544_flag0_multi248.3778_len1131	W4-3	1131	UUY86256.1 putative orf58-like protein [Largemouth bass virus]	100	160	4.81E-106	1835.85
k141_114775_flag1_multi36.6247_len1244	W5-2	1244	UUY86256.1 putative orf58-like protein [Largemouth bass virus]	100	160	1.78E-105	222
k141_4674_flag1_multi3.0000_len459	W3-1	459	AYV88121.1 hypothetical protein [Mandarin fish ranavirus]	98.7	151	1.79E-105	3
k141_202261_flag1_multi3.0000_len503	W3-3	503	UVF58785.1 MAG: major capsid protein [Halichoeres melanurus ranavirus]	100	154	3.11E-105	6
k141_13925_flag1_multi5.0000_len463	W3-1	463	AYV88131.1 putative eIF-2 alpha-like protein [Mandarin fish ranavirus]	100	153	3.83E-105	6
k141_125040_flag1_multi15.0000_len511	W6-3	511	UUY86216.1 hypothetical protein [Largemouth bass virus]	99.4	165	7.31E-105	25
k141_20534_flag1_multi9.0000_len1076	W3-1	1076	UUY86221.1 hypothetical protein [Largemouth bass virus]	98.9	177	8.17E-105	29
k141_9376_flag1_multi13.2474_len521	W3-1	521	AYV88126.1 putative immediate early protein ICP-18 [Mandarin fish ranavirus]	99.3	147	1.19E-104	17
k141_175103_flag1_multi400.2270_len1709	W2-3	1709	UUY86256.1 putative orf58-like protein [Largemouth bass virus]	92.1	242	3.05E-104	5642.75
k141_31432_flag1_multi6243.2500_len1741	W1-1	1741	UUY86256.1 putative orf58-like protein [Largemouth bass virus]	92.1	242	4.09E-104	62061.35
k141_127675_flag1_multi7.0000_len593	W3-2	593	UUY86218.1 hypothetical protein [Largemouth bass virus]	98.7	155	7.66E-104	11
k141_59753_flag1_multi20495.0000_len1350	W4-1	1350	UVF58785.1 MAG: major capsid protein [Halichoeres melanurus ranavirus]	100	156	1.38E-103	171223.32
k141_29370_flag1_multi31.0000_len798	W3-2	798	AYV88126.1 putative immediate early protein ICP-18 [Mandarin fish ranavirus]	99.3	147	3.17E-103	81
k141_246427_flag1_multi7.0000_len561	W1-3	561	UUY86269.1 hypothetical protein [Largemouth bass virus]	99.4	164	4.50E-103	12
k141_268124_flag1_multi17.9250_len861	W5-3	861	AYV88126.1 putative immediate early protein ICP-18 [Mandarin fish ranavirus]	99.3	147	9.51E-103	41
k141_157199_flag1_multi5.0000_len509	W5-2	509	UVF58793.1 MAG: DNA polymerase [Halichoeres melanurus ranavirus]	100	162	1.02E-102	8
k141_7178_flag0_multi80.6557_len1233	W5-3	1233	WEI29006.1 putative 2-cysteine adaptor domain protein [Largemouth bass virus]	100	152	1.66E-102	345

k141_21216_flag1_multi9.0000_len1908	W3-2	1908	UUY86221.1 hypothetical protein [Largemouth bass virus]	98.9	177	2.92E-102	53
k141_44197_flag1_multi7.0000_len879	W5-3	879	UUY86218.1 hypothetical protein [Largemouth bass virus]	98.7	155	2.92E-102	16
k141_225011_flag1_multi13.0000_len446	W6-3	446	AYV88120.1 putative p31K protein [Mandarin fish ranavirus]	98.6	148	1.47E-101	19
k141_135881_flag1_multi10759.0000_len2218	W5-1	2218	UUY86256.1 putative orf58-like protein [Largemouth bass virus]	100	160	2.39E-101	103510.49
k141_2743_flag1_multi5.0000_len480	W3-1	480	QJE49137.1 hypothetical protein LMBV_074 [Largemouth bass virus]	98.1	159	4.28E-101	6
k141_118642_flag1_multi4.0000_len432	W6-2	432	XRB52806.1 hypothetical protein LMBV_49 [Largemouth bass virus]	99.3	141	4.28E-101	5
k141_7287_flag1_multi16.0000_len1219	W3-1	1219	UUY86218.1 hypothetical protein [Largemouth bass virus]	98.7	155	5.65E-101	52
k141_88665_flag1_multi16.0000_len792	W1-3	792	AYV88126.1 putative immediate early protein ICP-18 [Mandarin fish ranavirus]	99.3	145	8.01E-101	40
k141_40633_flag0_multi3.3469_len1000	W5-1	1000	UUY86192.1 putative myristylated membrane protein [Largemouth bass virus]	92.5	159	3.69E-100	8
k141_125032_flag1_multi17.6904_len1349	W6-2	1349	UUY86218.1 hypothetical protein [Largemouth bass virus]	98.7	155	6.60E-100	72
k141_233065_flag1_multi368.0000_len3611	W4-3	3611	XRB52829.1 P8.141C-like protein, partial [Largemouth bass virus]	98.8	160	7.34E-100	4187
k141_17999_flag1_multi5.0000_len501	W6-1	501	UUY86233.1 hypothetical protein [Largemouth bass virus]	95.9	148	4.61E-99	5
k141_11670_flag1_multi2.0000_len535	W3-1	535	UVF58793.1 MAG: DNA polymerase [Halichoeres melanurus ranavirus]	100	155	9.02E-99	4
k141_7534_flag1_multi7.9699_len673	W3-2	673	QJE49085.1 hypothetical protein LMBV_022 [Largemouth bass virus]	97.8	139	9.71E-99	11
k141_33388_flag1_multi2.0000_len425	W3-3	425	AYV88159.1 hypothetical protein [Mandarin fish ranavirus]	100	141	3.07E-98	2
k141_18940_flag1_multi10.0000_len709	W6-3	709	UUY86256.1 putative orf58-like protein [Largemouth bass virus]	100	147	3.88E-98	22
k141_80021_flag1_multi2793.0000_len3928	W4-1	3928	UUY86256.1 putative orf58-like protein [Largemouth bass virus]	100	160	5.90E-97	37904
k141_24529_flag1_multi5.0000_len573	W1-2	573	QJE49128.1 hypothetical protein LMBV_065 [Largemouth bass virus]	98.5	133	1.00E-96	10
k141_52856_flag1_multi8.0000_len685	W4-3	685	QJE49128.1 hypothetical protein LMBV_065 [Largemouth bass virus]	98.5	133	4.24E-96	20
k141_205111_flag1_multi2.0000_len454	W3-3	454	AYV88211.1 hypothetical protein [Mandarin fish ranavirus]	100	150	6.82E-95	3
k141_109364_flag0_multi359.6471_len549	W2-1	549	AYV88126.1 putative immediate early protein ICP-18 [Mandarin fish ranavirus]	99.3	136	6.88E-95	1806
k141_110039_flag1_multi3.0000_len414	W6-3	414	QJE49097.1 hypothetical protein LMBV_034 [Largemouth bass virus]	100	137	7.58E-95	2
k141_7711_flag1_multi6.0000_len406	W3-1	406	QJE49109.1 hypothetical protein LMBV_046 [Largemouth bass virus]	100	135	1.18E-94	7
k141_80951_flag1_multi3.0000_len446	W6-3	446	WHA35533.1 putative DNA dependent RNA polymerase A subunit [Micropterus salmoides ranavirus]	100	148	2.68E-94	4
k141_11533_flag1_multi129.4239_len785	W2-1	785	AYV88185.1 hypothetical protein [Mandarin fish ranavirus]	97	132	2.84E-93	356
k141_47257_flag1_multi2.0000_len386	W5-3	386	QJE49128.1 hypothetical protein LMBV_065 [Largemouth bass virus]	98.4	128	5.91E-93	2
k141_11025_flag0_multi1142.8511_len732	W2-1	732	QJE49125.1 hypothetical protein LMBV_062 [Largemouth bass virus]	98.1	214	6.89E-93	8211
k141_43345_flag1_multi10.0000_len381	W6-3	381	QJE49149.1 putative immediate early protein ICP-46 [Largemouth bass virus]	100	126	4.72E-92	11
k141_225160_flag1_multi26.8158_len874	W5-3	874	QJE49119.1 hypothetical protein LMBV_056 [Largemouth bass virus]	100	140	1.17E-91	78
k141_179303_flag1_multi15.0000_len766	W1-3	766	QJE49119.1 hypothetical protein LMBV_056 [Largemouth bass virus]	100	139	1.22E-91	38
k141_14737_flag1_multi22.0000_len788	W3-1	788	QJE49119.1 hypothetical protein LMBV_056 [Largemouth bass virus]	100	139	1.64E-91	48
k141_104345_flag1_multi3.0000_len427	W5-2	427	AYV88179.2 putative DNA dependent RNA polymerase II second largest subunit [Mandarin fish ranavirus]	100	142	1.89E-91	2
k141_134652_flag3_multi10546.0000_len536	W1-1	536	AYV88191.1 hypothetical protein [Mandarin fish ranavirus]	99.2	130	2.41E-91	52361.96
k141_43035_flag1_multi1076.0280_len999	W2-1	999	QJE49119.1 hypothetical protein LMBV_056 [Largemouth bass virus]	99.3	141	3.53E-91	3736
k141_21166_flag1_multi26.0000_len763	W5-2	763	QJE49119.1 hypothetical protein LMBV_056 [Largemouth bass virus]	100	138	4.78E-91	65
k141_37090_flag1_multi7.0000_len429	W1-3	429	AYV88179.2 putative DNA dependent RNA polymerase II second largest subunit [Mandarin fish ranavirus]	100	142	2.64E-90	8
k141_144087_flag1_multi62.0000_len968	W4-3	968	QJE49119.1 hypothetical protein LMBV_056 [Largemouth bass virus]	100	138	4.08E-90	220

k141_119432_flag1_multi163.0000_len1405	W2-3	1405	QJE49119.1 hypothetical protein LMBV_056 [Largemouth bass virus]	100	141	7.36E-90	727
k141_19261_flag1_multi3.0000_len423	W3-1	423	AYV88179.2 putative DNA dependent RNA polymerase II second largest subunit [Mandarin fish ranavirus]	100	140	1.26E-89	3
k141_21163_flag0_multi441.9775_len585	W2-2	585	QJE49073.1 putative p31K protein [Largemouth bass virus]	100	138	2.46E-89	1309
k141_188002_flag1_multi3.0000_len482	W5-3	482	UUY86269.1 hypothetical protein [Largemouth bass virus]	99.3	147	3.12E-89	4
k141_188904_flag1_multi3.8041_len432	W5-3	432	XPZ21295.1 putative RNAseIII [Mandarin fish ranavirus]	99.3	143	4.98E-89	5
k141_90995_flag1_multi13.4669_len700	W6-1	700	AYV88208.1 hypothetical protein [Mandarin fish ranavirus]	100	129	1.84E-88	30
k141_80618_flag1_multi234.9130_len1348	W6-1	1348	QJE49119.1 hypothetical protein LMBV_056 [Largemouth bass virus]	100	139	2.58E-88	6294.41
k141_256006_flag1_multi3.0000_len425	W6-3	425	AYV88211.1 hypothetical protein [Mandarin fish ranavirus]	100	141	4.91E-88	4
k141_61389_flag1_multi12.0000_len2326	W6-1	2326	AYV88185.1 hypothetical protein [Mandarin fish ranavirus]	97.7	132	9.93E-88	94
k141_76455_flag0_multi410.9509_len548	W6-1	548	AYV88120.1 putative p31K protein [Mandarin fish ranavirus]	98.5	137	1.99E-87	1059
k141_240804_flag1_multi4.0000_len421	W6-3	421	UUY86258.1 hypothetical protein [Largemouth bass virus]	99.3	140	3.70E-87	4
k141_104768_flag0_multi585.0000_len966	W6-1	966	QJE49215.1 putative orf58-like protein [Largemouth bass virus]	100	132	7.26E-87	2014
k141_112996_flag1_multi9.0000_len420	W1-3	420	UVF58793.1 MAG: DNA polymerase [Halichoeres melanurus ranavirus]	100	140	1.24E-86	11
k141_273023_flag1_multi4.0000_len619	W5-3	619	AYV88204.1 putative NIF/NLI interacting factor [Mandarin fish ranavirus]	99.3	134	1.29E-86	7
k141_2470_flag1_multi25.0000_len779	W3-1	779	XRL22828.1 DNA-directed RNA polymerase subunit beta [Siniperca chuatsi ranavirus]	98.7	152	1.62E-86	62
k141_224120_flag1_multi5.0000_len433	W5-3	433	WHA35533.1 putative DNA dependent RNA polymerase A subunit [Micropterus salmoides ranavirus]	100	144	3.12E-86	5
k141_42247_flag1_multi5.0000_len433	W5-3	433	UUY86269.1 hypothetical protein [Largemouth bass virus]	98.6	144	1.32E-85	6
k141_211606_flag1_multi4.0000_len583	W6-3	583	XRB52842.1 transcription factor TFIIB cyclin-like domain-containing protein, partial [Largemouth bass virus]	100	125	2.23E-85	6
k141_87762_flag1_multi239.3994_len3008	W1-1	3008	QJE49119.1 hypothetical protein LMBV_056 [Largemouth bass virus]	100	141	2.34E-84	5543
k141_98614_flag1_multi4.0000_len353	W1-3	353	ADB77862.1 DNA methyltransferase [Largemouth bass ulcerative syndrome virus]	100	117	3.83E-84	3
k141_229916_flag1_multi4.0000_len449	W6-3	449	AYV88155.1 hypothetical protein [Mandarin fish ranavirus]	97.7	129	1.39E-83	5
k141_24074_flag1_multi4.0000_len463	W5-2	463	AYV88179.2 putative DNA dependent RNA polymerase II second largest subunit [Mandarin fish ranavirus]	100	135	1.54E-83	4
k141_75357_flag1_multi4.0000_len536	W5-2	536	QJE49128.1 hypothetical protein LMBV_065 [Largemouth bass virus]	98.3	118	2.20E-83	8
k141_7478_flag1_multi5.0000_len376	W5-2	376	AYV88136.2 hypothetical protein [Mandarin fish ranavirus]	98.4	123	3.25E-83	5
k141_142161_flag1_multi6.0000_len369	W1-3	369	AYV88173.2 putative ribonucleotide reductase beta subunit [Mandarin fish ranavirus]	100	123	5.84E-83	9
k141_25586_flag0_multi90.2459_len1231	W6-1	1231	UUY86204.1 hypothetical protein [Largemouth bass virus]	100	149	3.05E-82	827
k141_34464_flag1_multi4.0000_len410	W3-1	410	ABA41591.1 DNA-dependent DNA polymerase, partial [Largemouth bass virus]	98.5	136	1.28E-81	4
k141_120855_flag1_multi1.0000_len663	W5-1	663	UUY86258.1 hypothetical protein [Largemouth bass virus]	91.9	149	1.06E-80	1
k141_111147_flag1_multi5.0000_len352	W4-2	352	AYV88208.1 hypothetical protein [Mandarin fish ranavirus]	100	117	2.14E-80	3
k141_39172_flag1_multi3.0000_len375	W6-3	375	XPZ21261.1 hypothetical protein MRVORF002 [Mandarin fish ranavirus]	99.2	125	1.59E-79	3
k141_145314_flag1_multi1.0000_len366	W5-1	366	UVF58785.1 MAG: major capsid protein [Halichoeres melanurus ranavirus]	95.9	122	3.48E-79	0
k141_34524_flag1_multi20.0000_len1146	W3-1	1146	UUY86234.1 hypothetical protein [Largemouth bass virus]	100	123	9.12E-79	78
k141_45500_flag1_multi1.0000_len629	W2-1	629	UUY86256.1 putative orf58-like protein [Largemouth bass virus]	84.4	147	1.02E-78	2340.16
k141_153608_flag1_multi53.0000_len1171	W6-2	1171	UUY86234.1 hypothetical protein [Largemouth bass virus]	100	123	1.21E-78	224
k141_245910_flag1_multi16.0000_len790	W1-3	790	WAK75073.1 hypothetical protein [Mandarin fish ranavirus]	100	119	1.33E-78	33

k141_84366_flag1_multi17.1461_len1236	W5-3	1236	UUY86234.1 hypothetical protein [Largemouth bass virus]	100	123	2.33E-78	76
k141_32018_flag1_multi1.0000_len428	W5-1	428	QYU76034.1 putative myristylated membrane protein, partial [Koi ranavirus]	95.8	142	3.65E-78	0
k141_48751_flag1_multi10.0000_len348	W6-3	348	QJE49109.1 hypothetical protein LMBV_046 [Largemouth bass virus]	100	115	2.05E-77	12
k141_7920_flag0_multi7384.8387_len1468	W2-1	1468	UUY86234.1 hypothetical protein [Largemouth bass virus]	100	123	2.21E-77	7696
k141_267941_flag1_multi4.0000_len387	W5-3	387	UUY86269.1 hypothetical protein [Largemouth bass virus]	99.2	129	2.21E-77	4
k141_102429_flag1_multi33.8577_len1097	W5-2	1097	WAK75073.1 hypothetical protein [Mandarin fish ranavirus]	100	119	3.12E-77	132
k141_91875_flag1_multi153.1910_len1073	W6-2	1073	UUY86251.1 hypothetical protein [Largemouth bass virus]	99.2	120	1.10E-76	718
k141_73340_flag1_multi32.0000_len1335	W1-2	1335	WAK75073.1 hypothetical protein [Mandarin fish ranavirus]	100	119	3.51E-76	207
k141_100_flag1_multi2.0000_len353	W6-3	353	UUY86238.1 hypothetical protein [Largemouth bass virus]	99.1	117	6.11E-76	0
k141_78704_flag0_multi25.0028_len1194	W5-3	1194	WAK75073.1 hypothetical protein [Mandarin fish ranavirus]	99.2	118	9.75E-76	92
k141_128726_flag1_multi1.0000_len340	W1-1	340	WEI29006.1 putative 2-cysteine adaptor domain protein [Largemouth bass virus]	99.1	112	1.61E-75	0
k141_160409_flag1_multi3.0000_len422	W5-3	422	ADB77862.1 DNA methyltransferase [Largemouth bass ulcerative syndrome virus]	100	105	3.28E-75	2
k141_75522_flag1_multi1.0000_len359	W4-1	359	AYV88120.1 putative p31K protein [Mandarin fish ranavirus]	95.8	119	3.68E-75	0
k141_165032_flag1_multi3.0000_len362	W1-3	362	AYV88179.2 putative DNA dependent RNA polymerase II second largest subunit [Mandarin fish ranavirus]	100	120	4.04E-75	4
k141_15852_flag1_multi364.0703_len1692	W6-1	1692	WAK75073.1 hypothetical protein [Mandarin fish ranavirus]	100	119	8.64E-75	1609
k141_9408_flag0_multi20.0000_len515	W3-2	515	UUY86257.1 hypothetical protein [Largemouth bass virus]	92.7	123	1.22E-74	50
k141_33957_flag1_multi28.0000_len1709	W3-1	1709	WAK75073.1 hypothetical protein [Mandarin fish ranavirus]	99.2	119	3.95E-74	148
k141_48689_flag0_multi322.0000_len498	W4-3	498	UUY86257.1 hypothetical protein [Largemouth bass virus]	92.6	122	5.56E-74	146.45
k141_86554_flag1_multi6.0000_len446	W3-2	446	AYV88183.1 hypothetical protein [Mandarin fish ranavirus]	99.1	113	7.64E-74	7
k141_87806_flag0_multi302.2527_len513	W2-1	513	AYV88121.1 hypothetical protein [Mandarin fish ranavirus]	98.3	117	1.09E-73	682
k141_55135_flag1_multi9437.0000_len1687	W4-1	1687	UVF58785.1 MAG: major capsid protein [Halichoeres melanurus ranavirus]	100	127	1.45E-73	88052.16
k141_16508_flag1_multi3.0000_len353	W3-2	353	AYV88179.2 putative DNA dependent RNA polymerase II second largest subunit [Mandarin fish ranavirus]	100	117	1.70E-73	2
k141_41165_flag1_multi3.0000_len359	W5-3	359	WHA35533.1 putative DNA dependent RNA polymerase A subunit [Micropterus salmoides ranavirus]	100	119	3.86E-73	3
k141_121765_flag1_multi30.5923_len872	W5-2	872	UUY86257.1 hypothetical protein [Largemouth bass virus]	92.7	123	9.74E-73	218
k141_18640_flag1_multi2.0000_len331	W6-3	331	UUY86267.1 ribonucleotide reductase alpha subunit [Largemouth bass virus]	100	109	2.22E-72	1
k141_32584_flag1_multi88.0000_len2644	W4-2	2644	WAK75073.1 hypothetical protein [Mandarin fish ranavirus]	100	119	3.57E-72	739
k141_30403_flag0_multi15.0000_len446	W5-2	446	UUY86221.1 hypothetical protein [Largemouth bass virus]	100	122	6.99E-72	9
k141_214089_flag1_multi7.0000_len316	W1-3	316	ACP19247.1 DNA polymerase, partial [Doctor fish virus]	100	104	7.80E-72	8
k141_123470_flag1_multi31.3813_len1038	W1-2	1038	QJE49119.1 hypothetical protein LMBV_056 [Largemouth bass virus]	100	114	9.76E-72	113
k141_131987_flag1_multi1.0000_len533	W1-1	533	AIG51690.1 major capsid protein, partial [Koi ranavirus]	93.1	131	2.62E-71	3
k141_101207_flag1_multi1.0000_len373	W5-1	373	AYV88120.1 putative p31K protein [Mandarin fish ranavirus]	94.3	123	4.02E-71	0
k141_19304_flag1_multi5.0000_len382	W3-1	382	QJE49137.1 hypothetical protein LMBV_074 [Largemouth bass virus]	99.2	127	1.58E-70	5
k141_139620_flag1_multi42.6660_len1147	W6-2	1147	WAK75073.1 hypothetical protein [Mandarin fish ranavirus]	100	110	3.32E-70	155
k141_30533_flag1_multi5.4376_len630	W1-3	630	UUY86251.1 hypothetical protein [Largemouth bass virus]	100	106	4.00E-70	10
k141_34768_flag1_multi24.0000_len369	W5-3	369	UUY86216.1 hypothetical protein [Largemouth bass virus]	99.1	111	5.72E-70	57
k141_99928_flag1_multi8.0000_len333	W3-3	333	XRB52768.1 P31K protein, partial [Largemouth bass virus]	98.2	110	7.39E-70	9
k141_9753_flag1_multi4.0000_len446	W6-3	446	UUY86269.1 hypothetical protein [Largemouth bass virus]	100	108	1.23E-69	4

k141_18034_flag1_multi6.0000_len341	W3-1	341	UVF58793.1 MAG: DNA polymerase [Halichoeres melanurus ranavirus]	100	113	3.13E-69	5
k141_128539_flag1_multi49.0000_len350	W1-2	350	QJE49138.1 hypothetical protein LMBV_075 [Largemouth bass virus]	98.1	106	6.93E-69	60
k141_70112_flag1_multi3.0000_len361	W3-2	361	UUY86207.1 hypothetical protein [Largemouth bass virus]	96.3	107	1.02E-68	2
k141_133485_flag1_multi5.0000_len591	W6-2	591	XPZ21295.1 putative RNAseIII [Mandarin fish ranavirus]	99.2	120	1.84E-68	8
k141_201648_flag1_multi3.0000_len328	W6-3	328	QJE49095.1 hypothetical protein LMBV_032 [Largemouth bass virus]	100	109	3.87E-68	1
k141_144322_flag1_multi1.0000_len506	W5-1	506	AYV88159.1 hypothetical protein [Mandarin fish ranavirus]	87.2	125	8.59E-68	2331.15
k141_56646_flag1_multi13.0000_len619	W5-2	619	UUY86251.1 hypothetical protein [Largemouth bass virus]	100	103	2.77E-67	22
k141_51626_flag1_multi6.0000_len349	W3-2	349	XPZ21295.1 putative RNAseIII [Mandarin fish ranavirus]	98.3	116	5.46E-67	6
k141_108857_flag1_multi13.0000_len796	W5-3	796	QJE49138.1 hypothetical protein LMBV_075 [Largemouth bass virus]	98.1	106	6.79E-67	40
k141_104129_flag1_multi12.9310_len663	W1-3	663	QJE49138.1 hypothetical protein LMBV_075 [Largemouth bass virus]	98.1	105	1.12E-66	33
k141_23560_flag1_multi10.0000_len338	W3-1	338	QJE49138.1 hypothetical protein LMBV_075 [Largemouth bass virus]	98.1	104	1.14E-66	10
k141_36962_flag0_multi1.0000_len494	W5-1	494	AIG51690.1 major capsid protein, partial [Koi ranavirus]	94.1	118	1.48E-66	29.74
k141_74_flag1_multi1.0000_len322	W5-1	322	QIZ30887.1 major capsid protein, partial [Largemouth bass virus]	92.5	107	1.72E-66	0
k141_150034_flag0_multi1.0000_len433	W5-1	433	AIG51690.1 major capsid protein, partial [Koi ranavirus]	96.4	111	1.90E-66	7.72
k141_106064_flag1_multi5.0000_len346	W5-2	346	QJE49097.1 hypothetical protein LMBV_034 [Largemouth bass virus]	99.1	114	2.01E-66	3
k141_82359_flag1_multi1.0000_len312	W5-1	312	UVF58785.1 MAG: major capsid protein [Halichoeres melanurus ranavirus]	97.1	104	2.62E-66	0
k141_166270_flag1_multi7.0000_len324	W1-3	324	AYV88134.2 putative tyrosine kinase [Mandarin fish ranavirus]	100	107	5.85E-66	10
k141_17386_flag0_multi2.8553_len445	W5-3	445	UUY86198.1 hypothetical protein [Largemouth bass virus]	100	103	6.50E-66	4
k141_168731_flag1_multi1.0000_len360	W5-1	360	QJE49137.1 hypothetical protein LMBV_074 [Largemouth bass virus]	95	119	1.37E-65	0
k141_7626_flag1_multi3.0000_len328	W6-3	328	UUY86258.1 hypothetical protein [Largemouth bass virus]	100	108	1.62E-65	3
k141_42104_flag1_multi9.1679_len671	W5-2	671	QJE49138.1 hypothetical protein LMBV_075 [Largemouth bass virus]	99	104	2.05E-65	26
k141_150090_flag1_multi1.0000_len328	W5-1	328	XRL22790.1 hypothetical protein [Siniperca chuatsi ranavirus]	96.3	108	2.83E-65	1
k141_181039_flag1_multi5.0000_len340	W1-3	340	WEI28968.1 putative DNA dependent RNA polymerase a subunit [Largemouth bass virus]	100	112	3.12E-65	5
k141_20214_flag1_multi1.0000_len349	W5-1	349	QYU76034.1 putative myristylated membrane protein, partial [Koi ranavirus]	91.3	115	4.24E-65	0
k141_40012_flag1_multi1.0000_len310	W1-1	310	UVF58785.1 MAG: major capsid protein [Halichoeres melanurus ranavirus]	98.1	103	5.57E-65	0
k141_105146_flag1_multi1.0000_len357	W5-1	357	UUY86256.1 putative orf58-like protein [Largemouth bass virus]	93.8	112	7.53E-65	130.93
k141_57538_flag1_multi5.0000_len321	W5-3	321	QJE49137.1 hypothetical protein LMBV_074 [Largemouth bass virus]	100	106	1.36E-64	1
k141_174145_flag1_multi3.0000_len311	W3-3	311	AYV88149.2 hypothetical protein [Mandarin fish ranavirus]	98	100	3.10E-64	2
k141_10703_flag0_multi26.6576_len544	W1-1	544	AYV88191.1 hypothetical protein [Mandarin fish ranavirus]	93.4	106	3.48E-64	16366.04
k141_31211_flag1_multi3.0000_len393	W3-1	393	QJE49085.1 hypothetical protein LMBV_022 [Largemouth bass virus]	98.9	91	9.12E-64	3
k141_199634_flag1_multi3.0000_len316	W1-3	316	UVF58793.1 MAG: DNA polymerase [Halichoeres melanurus ranavirus]	100	105	9.56E-64	2
k141_5600_flag1_multi7.0000_len302	W3-1	302	QJE49123.1 putative DNA repair protein RAD2 [Largemouth bass virus]	100	100	1.59E-63	7
k141_155744_flag1_multi1.0000_len451	W5-1	451	AYV88191.1 hypothetical protein [Mandarin fish ranavirus]	86.4	118	1.73E-63	1
k141_38143_flag1_multi3.0000_len348	W1-2	348	AYV88208.1 hypothetical protein [Mandarin fish ranavirus]	100	93	2.46E-63	2
k141_95083_flag1_multi1.0000_len332	W4-1	332	AYV88120.1 putative p31K protein [Mandarin fish ranavirus]	91.8	110	7.11E-63	1
k141_44813_flag0_multi1.0000_len528	W4-1	528	UZV46047.1 major capsid protein, partial [Largemouth bass virus]	89.6	115	8.24E-63	29079
k141_2544_flag1_multi5.0000_len305	W3-3	305	AIG51690.1 major capsid protein, partial [Koi ranavirus]	100	101	1.08E-62	4
k141_18935_flag1_multi1.0000_len337	W5-1	337	UUY86261.1 hypothetical protein [Largemouth bass virus]	98.2	112	4.91E-62	0
k141_38492_flag0_multi1.0000_len282	W5-1	282	AYV88159.1 hypothetical protein [Mandarin fish ranavirus]	98.9	93	1.65E-61	899.06

k141_82181_flag1_multi1.0000_len324	W5-1	324	UUY86241.1 hypothetical protein [Largemouth bass virus]	96.5	86	2.11E-61	1
k141_68259_flag1_multi1.0000_len371	W5-1	371	AIG51690.1 major capsid protein, partial [Koi ranavirus]	94.5	110	2.13E-61	1
k141_120880_flag1_multi1.0000_len327	W5-1	327	QJE49215.1 putative orf58-like protein [Largemouth bass virus]	96.9	96	2.86E-61	0
k141_26109_flag1_multi4.0000_len309	W3-1	309	WHA35533.1 putative DNA dependent RNA polymerase A subunit [Micropterus salmoides ranavirus]	100	102	3.22E-61	3
k141_201663_flag1_multi16.0000_len591	W5-3	591	UUY86251.1 hypothetical protein [Largemouth bass virus]	100	94	3.37E-61	24
k141_122015_flag1_multi1.0000_len295	W5-1	295	UVF58785.1 MAG: major capsid protein [Halichoeres melanurus ranavirus]	96.9	98	1.37E-60	0
k141_70526_flag1_multi1.0000_len281	W1-1	281	AYV88159.1 hypothetical protein [Mandarin fish ranavirus]	100	93	2.72E-60	0
k141_43473_flag0_multi1.0000_len282	W5-1	282	AYV88159.1 hypothetical protein [Mandarin fish ranavirus]	98.9	93	5.48E-60	149.14
k141_50251_flag0_multi1.0000_len312	W5-1	312	AIG51690.1 major capsid protein, partial [Koi ranavirus]	95.1	103	8.46E-60	121.23
k141_41688_flag1_multi1.0000_len341	W5-1	341	UUY86258.1 hypothetical protein [Largemouth bass virus]	94.7	113	1.10E-59	3
k141_134553_flag1_multi1.0000_len286	W5-1	286	UVF58785.1 MAG: major capsid protein [Halichoeres melanurus ranavirus]	97.9	94	1.33E-59	0
k141_45372_flag0_multi1.0000_len329	W1-1	329	XRL22790.1 hypothetical protein [Siniperca chuatsi ranavirus]	91.7	108	1.65E-59	3995.08
k141_74891_flag1_multi1.0000_len342	W5-1	342	QJE49153.1 hypothetical protein LMBV_004 [Largemouth bass virus]	96.1	103	2.03E-59	0
k141_201037_flag1_multi2.0000_len305	W6-3	305	AYV88134.2 putative tyrosine kinase [Mandarin fish ranavirus]	100	101	4.45E-59	1
k141_126897_flag0_multi1.7538_len535	W5-2	535	XPZ21295.1 putative RNAseIII [Mandarin fish ranavirus]	82.4	136	7.48E-59	1
k141_48031_flag1_multi1.0000_len586	W4-1	586	XRL22790.1 hypothetical protein [Siniperca chuatsi ranavirus]	93.4	106	8.25E-59	0
k141_86763_flag1_multi30.2031_len1165	W4-1	1165	AYV88120.1 putative p31K protein [Mandarin fish ranavirus]	94.5	110	1.55E-58	133.26
k141_61328_flag1_multi5.5287_len385	W1-3	385	WAK75094.1 hypothetical protein [Mandarin fish ranavirus]	100	82	1.64E-58	5
k141_42162_flag1_multi49.6296_len465	W5-2	465	WAK75112.1 putative LITAF PIG7 possible membrane associated motif in LPS-induced tumor necrosis factor alpha factor [Mandarin fish ranavirus]	100	85	2.69E-58	69
k141_47692_flag0_multi332.0000_len297	W6-1	297	UUY86197.1 hypothetical protein [Largemouth bass virus]	100	99	2.82E-58	120
k141_199098_flag1_multi3.0000_len356	W4-3	356	QJE49085.1 hypothetical protein LMBV_022 [Largemouth bass virus]	97.6	84	3.20E-58	3
k141_20649_flag1_multi57.0000_len386	W3-1	386	WAK75112.1 putative LITAF PIG7 possible membrane associated motif in LPS-induced tumor necrosis factor alpha factor [Mandarin fish ranavirus]	98.8	85	3.94E-58	72
k141_103636_flag1_multi2.0000_len505	W5-2	505	AYV88134.2 putative tyrosine kinase [Mandarin fish ranavirus]	100	94	4.79E-58	2
k141_100332_flag1_multi1.0000_len281	W5-1	281	QJE49066.1 hypothetical protein LMBV_003 [Largemouth bass virus]	100	93	5.33E-58	0
k141_26627_flag1_multi1.0000_len293	W4-1	293	AYV88159.1 hypothetical protein [Mandarin fish ranavirus]	96.9	97	8.67E-58	0
k141_141614_flag1_multi1.0000_len360	W5-1	360	QJE49132.1 hypothetical protein LMBV_069 [Largemouth bass virus]	90.8	119	1.06E-57	1
k141_81646_flag1_multi3.0593_len731	W4-1	731	XRL22821.1 hypothetical protein [Siniperca chuatsi ranavirus]	87.9	116	1.53E-57	6
k141_39687_flag1_multi1.0000_len309	W4-1	309	AIG51690.1 major capsid protein, partial [Koi ranavirus]	96.1	102	2.06E-57	0
k141_109815_flag1_multi1.0000_len326	W2-1	326	UUY86227.1 hypothetical protein [Largemouth bass virus]	82.4	108	5.62E-57	0
k141_50065_flag0_multi1.0000_len282	W5-1	282	UVF58785.1 MAG: major capsid protein [Halichoeres melanurus ranavirus]	98.9	93	5.87E-57	958.11
k141_89647_flag1_multi224.5107_len795	W4-2	795	WAK75112.1 putative LITAF PIG7 possible membrane associated motif in LPS-induced tumor necrosis factor alpha factor [Mandarin fish ranavirus]	100	85	6.96E-57	543
k141_65082_flag1_multi4.0000_len311	W6-3	311	UUY86269.1 hypothetical protein [Largemouth bass virus]	99	103	7.72E-57	4
k141_139920_flag1_multi1.0000_len574	W5-1	574	XRB52768.1 P31K protein, partial [Largemouth bass virus]	92.1	101	1.15E-56	2
k141_75887_flag1_multi3.0000_len345	W1-1	345	UVF58785.1 MAG: major capsid protein [Halichoeres melanurus ranavirus]	82.5	114	3.91E-56	0
k141_118680_flag1_multi1.0000_len455	W1-1	455	AIG51690.1 major capsid protein, partial [Koi ranavirus]	75.4	142	1.17E-55	2
k141_108844_flag0_multi1.0000_len278	W1-1	278	AIG51690.1 major capsid protein, partial [Koi ranavirus]	98.9	92	1.21E-55	24726.77

k141_11082_flag0_multi1.0000_len304	W1-1	304	AIG51690.1 major capsid protein, partial [Koi ranavirus]	96	101	1.76E-55	5992.98
k141_62093_flag1_multi1.0000_len281	W5-1	281	AIG51690.1 major capsid protein, partial [Koi ranavirus]	98.9	93	2.53E-55	2.98
k141_21637_flag1_multi4.8115_len629	W3-1	629	UUY86257.1 hypothetical protein [Largemouth bass virus]	91.8	97	3.50E-55	7
k141_81600_flag0_multi1.0000_len289	W4-1	289	AIG51690.1 major capsid protein, partial [Koi ranavirus]	97.9	96	4.04E-55	6407.23
k141_85502_flag1_multi1.0000_len402	W5-1	402	QJE49215.1 putative orf58-like protein [Largemouth bass virus]	92.5	93	6.59E-55	2.1
k141_131786_flag1_multi2.0000_len649	W2-2	649	UUY86227.1 hypothetical protein [Largemouth bass virus]	83.5	109	9.86E-55	4
k141_108962_flag1_multi4.2099_len970	W1-1	970	WEI28972.1 CTD-phosphotransferase [Largemouth bass virus]	76.6	124	1.11E-54	11.1
k141_13338_flag1_multi3.5259_len681	W5-1	681	XRL22821.1 hypothetical protein [Siniperca chuatsi ranavirus]	94.9	99	1.26E-54	9.09
k141_120741_flag1_multi4.9222_len1272	W6-3	1272	AYV88191.1 hypothetical protein [Mandarin fish ranavirus]	98.9	89	1.71E-54	30
k141_78661_flag1_multi1.0000_len361	W4-1	361	AYV88176.1 putative tumor necrosis factor receptor [Mandarin fish ranavirus]	98.8	80	2.18E-54	0
k141_9129_flag1_multi1.0000_len318	W1-1	318	QYU76034.1 putative myristylated membrane protein, partial [Koi ranavirus]	100	89	4.60E-54	0
k141_243691_flag1_multi2.0000_len317	W6-3	317	UUY86234.1 hypothetical protein [Largemouth bass virus]	100	86	4.71E-54	2
k141_87414_flag1_multi1.0000_len418	W5-1	418	XRL22790.1 hypothetical protein [Siniperca chuatsi ranavirus]	92.3	104	5.27E-54	0
k141_249205_flag1_multi4.0000_len302	W6-3	302	AYV88211.1 hypothetical protein [Mandarin fish ranavirus]	100	90	4.10E-53	4
k141_42308_flag1_multi2.6056_len785	W5-1	785	AYV88159.1 hypothetical protein [Mandarin fish ranavirus]	96.6	88	2.09E-52	32.02
k141_45566_flag0_multi1.0000_len458	W2-1	458	UUY86263.1 hypothetical protein [Largemouth bass virus]	97.8	90	2.30E-52	0
k141_74968_flag1_multi1.0000_len281	W1-1	281	AIG51690.1 major capsid protein, partial [Koi ranavirus]	98.9	93	2.52E-52	0
k141_124057_flag1_multi1.0000_len281	W5-1	281	XRL22790.1 hypothetical protein [Siniperca chuatsi ranavirus]	97.8	93	3.20E-52	0
k141_59372_flag1_multi28.0000_len319	W4-3	319	WXI69503.1 hypothetical protein [Largemouth bass virus]	95.7	92	4.77E-52	30
k141_94414_flag0_multi1.0000_len282	W1-1	282	AIG51690.1 major capsid protein, partial [Koi ranavirus]	98.9	94	5.23E-52	1538.56
k141_19751_flag1_multi1.0000_len304	W5-1	304	XRL22790.1 hypothetical protein [Siniperca chuatsi ranavirus]	94.7	94	6.06E-52	0
k141_141310_flag1_multi1.0000_len320	W5-1	320	QJE49066.1 hypothetical protein LMBV_003 [Largemouth bass virus]	87.7	106	6.19E-52	2
k141_73662_flag1_multi1.0000_len604	W4-1	604	WAK75073.1 hypothetical protein [Mandarin fish ranavirus]	91.5	94	6.42E-52	27
k141_98280_flag1_multi34.9753_len627	W5-1	627	UUY86227.1 hypothetical protein [Largemouth bass virus]	94.1	85	8.01E-52	320
k141_159970_flag1_multi1.0000_len281	W5-1	281	AYV88149.2 hypothetical protein [Mandarin fish ranavirus]	97.6	84	8.85E-52	0.82
k141_82689_flag1_multi1.0000_len477	W2-1	477	AYV88133.1 putative DNA-directed RNA polymerase II subunit [Mandarin fish ranavirus]	82.7	98	1.39E-51	2
k141_96382_flag1_multi4.0000_len317	W1-3	317	WEI28969.1 hypothetical protein [Largemouth bass virus]	100	83	2.33E-51	3
k141_104300_flag1_multi1.0000_len281	W4-1	281	QJE49132.1 hypothetical protein LMBV_069 [Largemouth bass virus]	98.9	93	2.44E-51	0
k141_19968_flag1_multi2.6058_len826	W5-1	826	QJE49149.1 putative immediate early protein ICP-46 [Largemouth bass virus]	95.5	88	3.98E-51	7
k141_121029_flag0_multi1.0000_len311	W5-1	311	XRB52831.1 hypothetical protein LMBV_74 [Largemouth bass virus]	96.1	102	8.40E-51	239.86
k141_51064_flag1_multi1.0000_len590	W2-1	590	WAK75073.1 hypothetical protein [Mandarin fish ranavirus]	95.5	88	9.12E-51	6
k141_135603_flag1_multi1.0000_len373	W5-1	373	UUY86235.1 hypothetical protein [Largemouth bass virus]	93.3	89	1.24E-50	0
k141_63726_flag1_multi1.0000_len380	W5-1	380	WHU98721.1 MCP, partial [Hybrid snakehead ranavirus]	95.5	88	2.84E-50	0
k141_132639_flag1_multi1.0000_len281	W5-1	281	QJE49105.1 hypothetical protein LMBV_042 [Largemouth bass virus]	98.8	86	3.48E-50	0
k141_56714_flag1_multi1.0000_len322	W1-1	322	AYV88120.1 putative p31K protein [Mandarin fish ranavirus]	80.4	107	4.68E-50	0
k141_162859_flag1_multi1.0000_len373	W5-1	373	QJE49066.1 hypothetical protein LMBV_003 [Largemouth bass virus]	79	124	5.60E-50	0
k141_3762_flag0_multi1.0000_len364	W4-1	364	AIG51690.1 major capsid protein, partial [Koi ranavirus]	94.4	89	9.81E-50	11616.05
k141_23684_flag1_multi4.0000_len715	W5-1	715	AYV88211.1 hypothetical protein [Mandarin fish ranavirus]	92.9	98	1.06E-49	11
k141_57398_flag1_multi1.0000_len324	W5-1	324	QJE49077.1 hypothetical protein LMBV_014 [Largemouth bass virus]	93.6	78	1.95E-49	0

k141_102859_flag1_multi1.0000_len281	W4-1	281	QJE49132.1 hypothetical protein LMBV_069 [Largemouth bass virus]	97.8	93	2.88E-49	0
k141_146377_flag1_multi5.8708_len1031	W5-1	1031	AYV88191.1 hypothetical protein [Mandarin fish ranavirus]	93.3	89	5.41E-49	27
k141_10492_flag1_multi126.7056_len389	W6-1	389	AYV88191.1 hypothetical protein [Mandarin fish ranavirus]	100	78	8.88E-49	161
k141_26280_flag0_multi385.0289_len971	W6-1	971	XRB52836.1 SAP domain-containing protein [Largemouth bass virus]	70.9	213	1.35E-48	1519
k141_84172_flag1_multi1.0000_len318	W1-1	318	QJE49101.1 hypothetical protein LMBV_038 [Largemouth bass virus]	91.4	93	2.22E-48	0
k141_44802_flag0_multi3.1024_len688	W4-1	688	AIG51690.1 major capsid protein, partial [Koi ranavirus]	97.6	83	2.71E-48	3438.12
k141_32218_flag1_multi3.0000_len337	W3-2	337	AYV88198.1 hypothetical protein [Mandarin fish ranavirus]	98.9	87	6.60E-48	2
k141_120202_flag1_multi5.0000_len310	W3-3	310	QJE49215.1 putative orf58-like protein [Largemouth bass virus]	100	72	4.70E-47	3
k141_9048_flag1_multi1.7041_len577	W3-1	577	AYV88167.1 hypothetical protein [Mandarin fish ranavirus]	98.6	73	9.91E-47	56
k141_77612_flag0_multi3.7700_len428	W6-2	428	AYV88167.1 hypothetical protein [Mandarin fish ranavirus]	100	71	1.84E-46	5
k141_130366_flag1_multi1.7172_len629	W5-1	629	XRB52797.1 Bcl-2 Bcl-2 homology region 1-3 domain-containing protein [Largemouth bass virus]	89.1	92	3.65E-46	3
k141_117052_flag0_multi2058.8519_len357	W1-1	357	AIG51690.1 major capsid protein, partial [Koi ranavirus]	100	76	1.32E-45	1391.23
k141_54552_flag0_multi1.0000_len371	W1-1	371	QJE49080.1 hypothetical protein LMBV_017 [Largemouth bass virus]	84	94	1.33E-45	1
k141_27638_flag1_multi4.5422_len792	W4-1	792	XRL22790.1 hypothetical protein [Siniperca chuatsi ranavirus]	95.5	88	1.36E-45	14
k141_25458_flag1_multi73.2836_len3096	W4-2	3096	AYV88125.2 hypothetical protein [Mandarin fish ranavirus]	94	84	1.63E-45	337
k141_61600_flag1_multi17.5367_len985	W1-1	985	AIG51690.1 major capsid protein, partial [Koi ranavirus]	97.6	85	1.78E-45	11048.04
k141_153895_flag1_multi89.0000_len425	W6-2	425	WAK75112.1 putative LITAF PIG7 possible membrane associated motif in LPS-induced tumor necrosis factor alpha factor [Mandarin fish ranavirus]	98.6	70	2.92E-45	83
k141_94323_flag0_multi1.0000_len386	W4-1	386	AIG51690.1 major capsid protein, partial [Koi ranavirus]	98.8	82	5.47E-45	3
k141_99952_flag1_multi5.0000_len394	W1-3	394	QJE49132.1 hypothetical protein LMBV_069 [Largemouth bass virus]	100	130	6.62E-45	6
k141_73746_flag1_multi1.0000_len636	W5-1	636	UUY86258.1 hypothetical protein [Largemouth bass virus]	90.3	93	1.23E-44	2
k141_25181_flag1_multi8.5014_len2315	W5-1	2315	AYV88191.1 hypothetical protein [Mandarin fish ranavirus]	97.5	80	2.12E-44	71.22
k141_24428_flag1_multi1.0000_len528	W1-1	528	AIG51690.1 major capsid protein, partial [Koi ranavirus]	91	89	3.24E-44	65982.9
k141_101459_flag1_multi20.6667_len357	W4-3	357	AYV88167.1 hypothetical protein [Mandarin fish ranavirus]	100	66	1.19E-43	30
k141_91087_flag1_multi6.0000_len608	W1-2	608	AYV88179.2 putative DNA dependent RNA polymerase II second largest subunit [Mandarin fish ranavirus]	100	81	1.22E-43	12
k141_99444_flag1_multi1.0000_len306	W5-1	306	QJE49066.1 hypothetical protein LMBV_003 [Largemouth bass virus]	98.7	79	1.59E-43	1
k141_92601_flag0_multi1612.9560_len1686	W1-1	1686	UVF58785.1 MAG: major capsid protein [Halichoeres melanurus ranavirus]	100	86	4.60E-43	30212.01
k141_116726_flag1_multi1.0000_len379	W5-1	379	QJE49132.1 hypothetical protein LMBV_069 [Largemouth bass virus]	81.1	111	6.32E-43	0
k141_19373_flag1_multi1.0000_len599	W1-1	599	QJE49101.1 hypothetical protein LMBV_038 [Largemouth bass virus]	94.3	87	9.61E-43	1
k141_87091_flag1_multi1.0000_len406	W4-1	406	QYU76034.1 putative myristylated membrane protein, partial [Koi ranavirus]	91	89	1.37E-42	13
k141_38297_flag1_multi1.0000_len353	W4-2	353	QJE49077.1 hypothetical protein LMBV_014 [Largemouth bass virus]	98.5	67	1.38E-42	36
k141_49952_flag1_multi9.9514_len4399	W1-1	4399	AYV88120.1 putative p31K protein [Mandarin fish ranavirus]	96.4	83	5.43E-42	174
k141_116467_flag0_multi7.5291_len468	W5-3	468	WAK75112.1 putative LITAF PIG7 possible membrane associated motif in LPS-induced tumor necrosis factor alpha factor [Mandarin fish ranavirus]	98.5	66	5.46E-42	28
k141_67968_flag0_multi1.0000_len787	W5-1	787	QJE49137.1 hypothetical protein LMBV_074 [Largemouth bass virus]	96.4	84	3.85E-41	20
k141_54883_flag0_multi590.5915_len939	W4-1	939	UVF58785.1 MAG: major capsid protein [Halichoeres melanurus ranavirus]	96.4	84	1.24E-40	3192
k141_13741_flag1_multi1.0000_len411	W5-1	411	XRB52842.1 transcription factor TFIIB cyclin-like domain-containing protein, partial [Largemouth bass virus]	94.2	69	2.67E-40	0

k141_104554_flag0_multi1.0000_len603	W5-1	603	AYV88120.1 putative p31K protein [Mandarin fish ranavirus]	85.9	85	4.33E-40	11654.04
k141_13975_flag1_multi1.0000_len358	W5-1	358	WEI28972.1 CTD-phosphotransferase [Largemouth bass virus]	94.8	77	4.61E-40	0
k141_30727_flag1_multi4.0000_len398	W6-3	398	WAK75097.1 hypothetical protein [Mandarin fish ranavirus]	100	68	8.47E-39	3
k141_31522_flag1_multi1.0000_len519	W2-1	519	AYV88159.1 hypothetical protein [Mandarin fish ranavirus]	82.4	85	1.22E-38	5
k141_156459_flag0_multi1.0000_len352	W5-1	352	AYV88191.1 hypothetical protein [Mandarin fish ranavirus]	97	67	1.65E-38	31.23
k141_97207_flag1_multi7.0000_len885	W5-1	885	WEI28998.1 hypothetical protein [Largemouth bass virus]	92.3	78	2.01E-38	19
k141_58582_flag1_multi1.0000_len465	W5-1	465	AFD96401.1 major capsid protein, partial [Largemouth bass virus]	88.5	78	3.13E-38	81.8
k141_162810_flag0_multi3.7326_len399	W5-1	399	AFD96401.1 major capsid protein, partial [Largemouth bass virus]	94.3	70	1.16E-37	7.33
k141_53982_flag0_multi59.9830_len729	W1-1	729	AIG51690.1 major capsid protein, partial [Koi ranavirus]	100	70	2.57E-37	6774.18
k141_126628_flag1_multi3.8399_len572	W1-1	572	WEI28972.1 CTD-phosphotransferase [Largemouth bass virus]	98.6	71	3.50E-37	4
k141_97923_flag1_multi5.6947_len1189	W2-1	1189	WEI28972.1 CTD-phosphotransferase [Largemouth bass virus]	84.3	89	4.55E-37	25
k141_6833_flag1_multi13.0000_len377	W3-1	377	WAK75121.1 hypothetical protein [Mandarin fish ranavirus]	96.8	63	5.79E-37	12
k141_8180_flag1_multi33.0998_len1423	W5-1	1423	QYU76034.1 putative myristylated membrane protein, partial [Koi ranavirus]	98.6	73	5.85E-37	352.02
k141_167168_flag0_multi1.0000_len430	W5-1	430	QIZ30887.1 major capsid protein, partial [Largemouth bass virus]	93.9	66	2.45E-36	9398.67
k141_16189_flag1_multi7.0000_len350	W6-2	350	QJE49203.1 hypothetical protein LMBV_054 [Largemouth bass virus]	96.3	82	2.59E-36	7
k141_33623_flag1_multi1.0000_len318	W5-1	318	AYV88159.1 hypothetical protein [Mandarin fish ranavirus]	93.9	66	3.97E-36	0
k141_101063_flag1_multi1.9922_len655	W5-1	655	QJE49137.1 hypothetical protein LMBV_074 [Largemouth bass virus]	98.6	71	5.15E-36	4
k141_160288_flag1_multi1.0000_len462	W5-1	462	AYV88120.1 putative p31K protein [Mandarin fish ranavirus]	97	66	9.95E-36	23
k141_75471_flag1_multi7.8802_len1485	W4-1	1485	AYV88121.1 hypothetical protein [Mandarin fish ranavirus]	94.6	74	1.00E-35	37
k141_128897_flag1_multi2.0000_len669	W5-1	669	QJE49077.1 hypothetical protein LMBV_014 [Largemouth bass virus]	95.2	63	1.16E-35	7
k141_105112_flag0_multi395.0079_len394	W4-1	394	QJE49203.1 hypothetical protein LMBV_054 [Largemouth bass virus]	98.7	78	2.63E-35	402
k141_85762_flag1_multi1.0000_len344	W4-1	344	AIG51690.1 major capsid protein, partial [Koi ranavirus]	84.9	86	3.81E-35	0
k141_3370_flag0_multi7.8845_len1474	W5-1	1474	AYP19467.1 hypothetical protein [Frog virus 3]	70.4	108	4.45E-35	23
k141_124085_flag1_multi1.0000_len336	W5-1	336	QJE49215.1 putative orf58-like protein [Largemouth bass virus]	82.1	78	6.74E-35	2
k141_150904_flag1_multi23.0000_len354	W1-3	354	WAK75072.1 hypothetical protein [Mandarin fish ranavirus]	100	62	8.32E-35	26
k141_53238_flag0_multi23.2126_len602	W4-1	602	WEI28972.1 CTD-phosphotransferase [Largemouth bass virus]	97.2	71	9.87E-35	33
k141_43468_flag0_multi1.0000_len330	W5-1	330	UUY86235.1 hypothetical protein [Largemouth bass virus]	96.7	61	1.06E-34	1
k141_81031_flag1_multi14.0000_len889	W4-2	889	WHA35556.1 putative LPXTG-anchored collagen-like adhesin Scl2/SclB [Micropterus salmoides ranavirus]	100	208	1.15E-34	37
k141_159434_flag0_multi1.0000_len426	W5-1	426	AIG51690.1 major capsid protein, partial [Koi ranavirus]	85.7	77	2.18E-34	16.01
k141_73703_flag1_multi18.0000_len2083	W5-1	2083	UUY86263.1 hypothetical protein [Largemouth bass virus]	95.8	72	3.43E-34	131
k141_38104_flag1_multi1.0000_len329	W4-1	329	UUY86267.1 ribonucleotide reductase alpha subunit [Largemouth bass virus]	80	80	5.30E-34	0
k141_106679_flag0_multi1.0000_len360	W2-1	360	WEI28972.1 CTD-phosphotransferase [Largemouth bass virus]	95.3	64	1.52E-33	35.7
k141_176071_flag0_multi2.3686_len670	W2-3	670	UUY86235.1 hypothetical protein [Largemouth bass virus]	95.5	66	2.89E-33	6
k141_164191_flag0_multi51.0000_len4401	W5-1	4401	AYV88211.1 hypothetical protein [Mandarin fish ranavirus]	93.9	82	2.95E-33	750
k141_56464_flag1_multi12.0000_len721	W4-3	721	WHA35556.1 putative LPXTG-anchored collagen-like adhesin Scl2/SclB [Micropterus salmoides ranavirus]	100	205	3.80E-33	30
k141_74344_flag1_multi19.0000_len2132	W1-1	2132	AYV88159.1 hypothetical protein [Mandarin fish ranavirus]	91.8	73	4.02E-33	141
k141_95987_flag1_multi14.0000_len1458	W2-2	1458	WHA35556.1 putative LPXTG-anchored collagen-like adhesin Scl2/SclB [Micropterus salmoides ranavirus]	100	208	6.08E-33	67

k141_145631_flag1_multi1.0000_len368	W5-1	368	AYV88191.1 hypothetical protein [Mandarin fish ranavirus]	96.4	55	6.09E-33	1
k141_5749_flag1_multi1.0000_len436	W5-1	436	AYV88191.1 hypothetical protein [Mandarin fish ranavirus]	90.8	65	6.85E-33	2
k141_41202_flag0_multi1.0000_len434	W5-1	434	AIG51690.1 major capsid protein, partial [Koi ranavirus]	92.6	68	6.93E-33	0
k141_41639_flag1_multi1.0000_len319	W1-1	319	AYV88191.1 hypothetical protein [Mandarin fish ranavirus]	96.6	59	9.02E-33	3466.7
k141_64096_flag1_multi33.0000_len1597	W6-1	1597	WHA35556.1 putative LPXTG-anchored collagen-like adhesin Scl2/SclB [Micropterus salmoides ranavirus]	100	208	1.15E-32	118
k141_141198_flag1_multi1.0000_len302	W5-1	302	QJE49067.1 hypothetical protein LMBV_004 [Largemouth bass virus]	81.8	77	1.22E-32	0
k141_91204_flag0_multi1.0000_len321	W1-1	321	UUY86266.1 hypothetical protein [Largemouth bass virus]	96.7	61	1.52E-32	3.03
k141_156332_flag1_multi12.6856_len1019	W5-1	1019	AYV88191.1 hypothetical protein [Mandarin fish ranavirus]	95.2	62	4.62E-32	59.09
k141_112637_flag1_multi3.8150_len514	W2-1	514	UUY86235.1 hypothetical protein [Largemouth bass virus]	64.1	103	4.69E-32	6
k141_34164_flag1_multi1.0000_len391	W5-1	391	XRL22790.1 hypothetical protein [Siniperca chuatsi ranavirus]	93.9	66	7.45E-32	180.41
k141_91965_flag0_multi1.0000_len238	W2-1	238	WXI69513.1 hypothetical protein [Largemouth bass virus]	86.1	79	8.09E-32	72
k141_60285_flag1_multi1.0000_len281	W5-1	281	XRB52842.1 transcription factor TFIIB cyclin-like domain-containing protein, partial [Largemouth bass virus]	98.4	63	1.04E-31	0
k141_118169_flag1_multi1.0000_len571	W5-1	571	AIG51690.1 major capsid protein, partial [Koi ranavirus]	94.2	69	1.29E-31	2
k141_105568_flag1_multi1.6689_len443	W5-1	443	XRL22821.1 hypothetical protein [Siniperca chuatsi ranavirus]	96.8	62	3.33E-31	2.57
k141_46899_flag1_multi114.0000_len791	W6-2	791	WAK75072.1 hypothetical protein [Mandarin fish ranavirus]	100	60	4.34E-31	142
k141_109619_flag1_multi14.2732_len1682	W5-1	1682	UVF58785.1 MAG: major capsid protein [Halichoeres melanurus ranavirus]	97.2	71	6.43E-31	86
k141_152057_flag1_multi1.0000_len446	W5-3	446	CBW45581.1 hypothetical protein, partial [Doctor fish virus]	87	69	1.03E-30	13
k141_73402_flag1_multi1.0000_len345	W5-1	345	AIG51690.1 major capsid protein, partial [Koi ranavirus]	94.1	68	1.92E-30	0
k141_22725_flag1_multi3.8165_len517	W3-1	517	UUY86269.1 hypothetical protein [Largemouth bass virus]	98.4	63	2.08E-30	5
k141_105093_flag1_multi5.3500_len861	W6-1	861	AIG51690.1 major capsid protein, partial [Koi ranavirus]	98.5	65	4.54E-30	3
k141_117063_flag0_multi1.0000_len383	W5-1	383	WAK75136.1 hypothetical protein [Mandarin fish ranavirus]	100	57	1.09E-29	776.74
k141_52924_flag1_multi4.9574_len704	W1-1	704	AYV88120.1 putative p31K protein [Mandarin fish ranavirus]	96.8	63	1.18E-29	11
k141_861_flag1_multi1.0000_len302	W4-1	302	UUY86195.1 hypothetical protein [Largemouth bass virus]	94.9	59	1.21E-29	0
k141_44142_flag1_multi1.0000_len320	W4-1	320	AIG51690.1 major capsid protein, partial [Koi ranavirus]	94	67	1.48E-29	0
k141_72438_flag1_multi1.0000_len476	W4-1	476	AYV88191.1 hypothetical protein [Mandarin fish ranavirus]	77.6	76	1.69E-29	181
k141_77731_flag1_multi5.8750_len557	W4-1	557	AYV88211.1 hypothetical protein [Mandarin fish ranavirus]	96.9	65	1.74E-29	17
k141_58569_flag1_multi1.9084_len818	W5-1	818	AFD96401.1 major capsid protein, partial [Largemouth bass virus]	96.6	59	2.10E-29	4.83
k141_129193_flag1_multi2.8957_len582	W5-1	582	WAK75094.1 hypothetical protein [Mandarin fish ranavirus]	98	50	2.36E-29	1
k141_113044_flag1_multi1.0000_len353	W5-1	353	AYV88120.1 putative p31K protein [Mandarin fish ranavirus]	85.9	64	3.96E-29	0
k141_106742_flag1_multi1.0000_len295	W6-1	295	UUY86241.1 hypothetical protein [Largemouth bass virus]	100	48	6.07E-29	0
k141_38160_flag1_multi3.0000_len1022	W5-1	1022	UUY86193.1 hypothetical protein [Largemouth bass virus]	89.2	65	6.34E-29	9
k141_57293_flag1_multi1.0000_len414	W4-1	414	QJE49149.1 putative immediate early protein ICP-46 [Largemouth bass virus]	100	54	7.58E-29	135
k141_82995_flag1_multi1.0000_len280	W5-1	280	AYV88191.1 hypothetical protein [Mandarin fish ranavirus]	83.1	59	9.99E-29	0
k141_66706_flag0_multi1.0000_len381	W5-1	381	UUY86261.1 hypothetical protein [Largemouth bass virus]	98.4	63	2.04E-28	3
k141_53661_flag1_multi133.1899_len2010	W2-1	2010	WHA35556.1 putative LPXTG-anchored collagen-like adhesin Scl2/SclB [Micropterus salmoides ranavirus]	100	202	2.48E-28	648
k141_162761_flag1_multi1.0000_len329	W5-1	329	WAK75068.1 hypothetical protein [Mandarin fish ranavirus]	94.4	54	3.45E-28	2814.39
k141_87350_flag1_multi6.6878_len583	W4-1	583	AYV88120.1 putative p31K protein [Mandarin fish ranavirus]	66.7	96	5.78E-28	43

k141_8888_flag1_multi1.0000_len281	W5-1	281	AYV88191.1 hypothetical protein [Mandarin fish ranavirus]	96.2	53	8.12E-28	0
k141_43805_flag1_multi1.0000_len481	W5-1	481	UUY86235.1 hypothetical protein [Largemouth bass virus]	84.4	64	1.01E-27	11.48
k141_23283_flag1_multi3.0135_len584	W2-1	584	WEI29006.1 putative 2-cysteine adaptor domain protein [Largemouth bass virus]	98	50	1.51E-27	8
k141_127062_flag0_multi7.2396_len454	W1-1	454	AIG51690.1 major capsid protein, partial [Koi ranavirus]	98.2	57	1.56E-27	0
k141_72180_flag1_multi1.0000_len399	W4-1	399	UUY86192.1 putative myristylated membrane protein [Largemouth bass virus]	87.7	57	2.05E-27	9
k141_11300_flag1_multi1.0000_len520	W4-1	520	AYV88194.1 hypothetical protein [Mandarin fish ranavirus]	85.5	69	2.40E-27	3
k141_59471_flag1_multi1.0000_len377	W1-1	377	QYU76034.1 putative myristylated membrane protein, partial [Koi ranavirus]	79.1	67	2.57E-27	0
k141_33544_flag1_multi6.0000_len353	W3-1	353	WAK75077.1 hypothetical protein [Mandarin fish ranavirus]	100	52	2.90E-27	3
k141_108486_flag1_multi3.7745_len447	W5-1	447	UUY86241.1 hypothetical protein [Largemouth bass virus]	97.9	47	3.12E-27	2
k141_101007_flag0_multi1.0000_len480	W5-1	480	UUY86241.1 hypothetical protein [Largemouth bass virus]	97.9	47	3.34E-27	7
k141_116248_flag1_multi1.0000_len504	W1-1	504	UVF58787.1 MAG: DNA-dependent RNA polymerase largest subunit, partial [Halichoeres melanurus ranavirus]	94.5	55	3.78E-27	1
k141_72042_flag1_multi1.0000_len385	W4-1	385	UUY86267.1 ribonucleotide reductase alpha subunit [Largemouth bass virus]	85.7	63	5.76E-27	1
k141_66748_flag0_multi1.0000_len348	W5-1	348	QJE49215.1 putative orf58-like protein [Largemouth bass virus]	87.1	62	6.11E-27	3
k141_22_flag0_multi7.7600_len941	W5-1	941	UUY86269.1 hypothetical protein [Largemouth bass virus]	92.4	66	7.72E-27	27.98
k141_104027_flag0_multi475.0000_len1602	W1-1	1602	AIG51690.1 major capsid protein, partial [Koi ranavirus]	100	58	8.98E-27	1212.04
k141_168715_flag1_multi1.0000_len588	W5-1	588	UUY86241.1 hypothetical protein [Largemouth bass virus]	61.8	89	1.07E-26	2
k141_78715_flag1_multi1.7372_len415	W4-1	415	UUY86229.1 putative DNA dependent RNA polymerase a subunit [Largemouth bass virus]	95.2	63	1.26E-26	1
k141_121153_flag0_multi13643.0000_len2220	W5-1	2220	CBW45581.1 hypothetical protein, partial [Doctor fish virus]	87	69	1.26E-26	1345
k141_2249_flag1_multi16.8258_len2254	W5-1	2254	UUY86227.1 hypothetical protein [Largemouth bass virus]	94.9	59	1.43E-26	131
k141_67942_flag1_multi2.9064_len739	W1-1	739	UUY86241.1 hypothetical protein [Largemouth bass virus]	79.4	63	1.62E-26	12
k141_179472_flag1_multi9.0000_len911	W1-3	911	QJE49090.1 hypothetical protein LMBV_027 [Largemouth bass virus]	96.7	60	1.91E-26	27
k141_6913_flag0_multi1.0000_len279	W5-1	279	WAK75112.1 putative LITAF PIG7 possible membrane associated motif in LPS-induced tumor necrosis factor alpha factor [Mandarin fish ranavirus]	100	46	2.03E-26	0
k141_86911_flag1_multi1.0000_len373	W6-1	373	AIG51690.1 major capsid protein, partial [Koi ranavirus]	74.7	79	2.41E-26	15
k141_128489_flag1_multi36.1614_len3723	W5-1	3723	WAK75073.1 hypothetical protein [Mandarin fish ranavirus]	94.7	57	2.50E-26	554
k141_1389_flag1_multi1.0000_len319	W1-1	319	AIG51690.1 major capsid protein, partial [Koi ranavirus]	86.6	67	3.32E-26	1146
k141_137807_flag1_multi1.0000_len310	W5-1	310	AFD96401.1 major capsid protein, partial [Largemouth bass virus]	85	60	3.38E-26	6
k141_97577_flag1_multi3.7738_len446	W4-1	446	WHU98721.1 MCP, partial [Hybrid snakehead ranavirus]	100	54	3.71E-26	948
k141_85098_flag1_multi1.0000_len311	W5-1	311	AAC79876.1 viral core protein, partial [Labroides dimidatus ranavirus]	95.9	49	4.20E-26	0
k141_117340_flag1_multi3.0000_len313	W6-3	313	WAK75103.1 hypothetical protein [Mandarin fish ranavirus]	100	52	4.50E-26	3
k141_33936_flag1_multi12.5453_len748	W1-1	748	AYV88191.1 hypothetical protein [Mandarin fish ranavirus]	97.9	47	4.68E-26	22
k141_76735_flag0_multi1.0000_len270	W2-1	270	AAC79876.1 viral core protein, partial [Labroides dimidatus ranavirus]	100	46	4.91E-26	0
k141_73262_flag1_multi1.0000_len280	W2-2	280	WAK75112.1 putative LITAF PIG7 possible membrane associated motif in LPS-induced tumor necrosis factor alpha factor [Mandarin fish ranavirus]	97.9	47	5.80E-26	0
k141_65614_flag0_multi99.3625_len301	W2-1	301	UUY86250.1 hypothetical protein [Largemouth bass virus]	100	57	7.75E-26	2
k141_65032_flag0_multi71.5825_len335	W5-1	335	AAC79876.1 viral core protein, partial [Labroides dimidatus ranavirus]	92.2	51	8.07E-26	0
k141_91258_flag1_multi1.0000_len522	W1-1	522	WAK75072.1 hypothetical protein [Mandarin fish ranavirus]	100	55	9.53E-26	210
k141_39458_flag1_multi1.0000_len272	W1-1	272	AYV88191.1 hypothetical protein [Mandarin fish ranavirus]	100	46	9.57E-26	0

k141_44430_flag1_multi1.8674_len488	W5-1	488	WEI29006.1 putative 2-cysteine adaptor domain protein [Largemouth bass virus]	97.9	47	9.86E-26	4
k141_82305_flag1_multi1.0000_len324	W5-1	324	WAK75112.1 putative LITAF PIG7 possible membrane associated motif in LPS-induced tumor necrosis factor alpha factor [Mandarin fish ranavirus]	100	46	9.95E-26	0
k141_124236_flag1_multi1.9428_len543	W2-2	543	UUY86241.1 hypothetical protein [Largemouth bass virus]	97.8	46	1.05E-25	4
k141_153587_flag1_multi1.0000_len281	W5-1	281	AYV88191.1 hypothetical protein [Mandarin fish ranavirus]	100	46	1.08E-25	0
k141_115864_flag1_multi2.7263_len521	W5-1	521	UUY86241.1 hypothetical protein [Largemouth bass virus]	95.7	47	1.15E-25	5
k141_104940_flag1_multi1.0000_len320	W5-1	320	QJE49112.1 hypothetical protein LMBV_049 [Largemouth bass virus]	97.9	47	1.17E-25	0
k141_29471_flag0_multi1.0000_len278	W5-1	278	WAK75107.1 hypothetical protein [Mandarin fish ranavirus]	100	45	1.41E-25	0
k141_37838_flag1_multi1.0000_len278	W5-1	278	WAK75112.1 putative LITAF PIG7 possible membrane associated motif in LPS-induced tumor necrosis factor alpha factor [Mandarin fish ranavirus]	100	46	1.60E-25	0
k141_122486_flag1_multi1.0000_len279	W1-1	279	QJE49077.1 hypothetical protein LMBV_014 [Largemouth bass virus]	91.8	49	1.79E-25	0
k141_9480_flag0_multi1.0000_len292	W5-1	292	AIG51690.1 major capsid protein, partial [Koi ranavirus]	92.7	55	2.42E-25	2.07
k141_64324_flag1_multi3.7893_len554	W4-1	554	WAK75112.1 putative LITAF PIG7 possible membrane associated motif in LPS-induced tumor necrosis factor alpha factor [Mandarin fish ranavirus]	98	49	2.45E-25	10
k141_96141_flag1_multi4.3359_len531	W1-1	531	UUY86241.1 hypothetical protein [Largemouth bass virus]	97.8	46	2.52E-25	9
k141_46707_flag1_multi6.6280_len512	W4-1	512	AFD96401.1 major capsid protein, partial [Largemouth bass virus]	98	50	2.58E-25	6
k141_26148_flag1_multi1.0000_len278	W4-1	278	CBW45583.1 ICP 46 homolog, partial [Doctor fish virus]	100	46	2.61E-25	0
k141_34813_flag1_multi2.8827_len533	W5-1	533	AAC79876.1 viral core protein, partial [Labroides dimidatus ranavirus]	100	46	2.77E-25	4
k141_167045_flag1_multi2.8663_len470	W5-1	470	UUY86199.1 hypothetical protein [Largemouth bass virus]	100	46	2.98E-25	6
k141_49335_flag1_multi3.8139_len1092	W5-1	1092	UUY86241.1 hypothetical protein [Largemouth bass virus]	92.5	53	3.06E-25	15
k141_178855_flag0_multi58.0000_len1301	W2-3	1301	WHA35556.1 putative LPXTG-anchored collagen-like adhesin Scl2/SclB [Micropterus salmoides ranavirus]	99	199	3.14E-25	78
k141_6879_flag0_multi15.4642_len783	W1-1	783	QJE49077.1 hypothetical protein LMBV_014 [Largemouth bass virus]	96	50	3.22E-25	22
k141_153370_flag1_multi2.9322_len539	W5-1	539	WEI29006.1 putative 2-cysteine adaptor domain protein [Largemouth bass virus]	97.9	47	3.27E-25	4
k141_109588_flag1_multi28.9688_len783	W5-1	783	WAK75112.1 putative LITAF PIG7 possible membrane associated motif in LPS-induced tumor necrosis factor alpha factor [Mandarin fish ranavirus]	100	48	3.69E-25	40
k141_66330_flag1_multi1.0000_len280	W5-1	280	WAK75107.1 hypothetical protein [Mandarin fish ranavirus]	100	45	4.03E-25	1
k141_14274_flag0_multi1.0000_len280	W5-1	280	AYV88191.1 hypothetical protein [Mandarin fish ranavirus]	100	46	4.35E-25	0
k141_142487_flag1_multi4.7923_len584	W5-1	584	UUY86241.1 hypothetical protein [Largemouth bass virus]	97.8	46	4.43E-25	4
k141_120826_flag1_multi3.9244_len657	W1-1	657	AAC79876.1 viral core protein, partial [Labroides dimidatus ranavirus]	90.4	52	4.71E-25	21
k141_91212_flag1_multi4.7333_len486	W5-1	486	UUY86199.1 hypothetical protein [Largemouth bass virus]	100	46	4.97E-25	4
k141_129534_flag1_multi1.0000_len588	W1-1	588	WAK75112.1 putative LITAF PIG7 possible membrane associated motif in LPS-induced tumor necrosis factor alpha factor [Mandarin fish ranavirus]	100	47	5.00E-25	0
k141_122367_flag1_multi2.6803_len654	W1-1	654	AYV88191.1 hypothetical protein [Mandarin fish ranavirus]	88.7	53	6.22E-25	7
k141_87611_flag1_multi1.0000_len419	W5-1	419	WAK75112.1 putative LITAF PIG7 possible membrane associated motif in LPS-induced tumor necrosis factor alpha factor [Mandarin fish ranavirus]	97.9	47	6.40E-25	1
k141_14833_flag1_multi1.0000_len464	W1-1	464	AYV88191.1 hypothetical protein [Mandarin fish ranavirus]	97.9	47	7.02E-25	17.09
k141_1040_flag1_multi5.1812_len279	W1-1	279	WAK75107.1 hypothetical protein [Mandarin fish ranavirus]	100	45	8.13E-25	0
k141_27467_flag1_multi4.8185_len648	W1-1	648	AAC79876.1 viral core protein, partial [Labroides dimidatus ranavirus]	100	46	8.64E-25	8
k141_111406_flag1_multi7.7755_len858	W2-1	858	AYV88191.1 hypothetical protein [Mandarin fish ranavirus]	100	46	9.22E-25	13

k141_76753_flag1_multi2.8909_len581	W5-1	581	UUY86199.1 hypothetical protein [Largemouth bass virus]	100	46	9.62E-25	3
k141_67427_flag1_multi1.5756_len617	W5-1	617	QJE49097.1 hypothetical protein LMBV_034 [Largemouth bass virus]	89.8	59	1.07E-24	4
k141_36770_flag0_multi62.6134_len1957	W5-1	1957	QJE49137.1 hypothetical protein LMBV_074 [Largemouth bass virus]	94.9	59	1.31E-24	309
k141_86746_flag0_multi30.5790_len2065	W4-1	2065	AAF64582.1 capsid protein, partial [Guppyfish iridovirus]	93.3	60	1.41E-24	158
k141_40140_flag1_multi1.9450_len559	W1-1	559	AAC79876.1 viral core protein, partial [Labroides dimidatus ranavirus]	95.8	48	1.43E-24	3
k141_39552_flag0_multi1.0000_len281	W1-1	281	QIZ30887.1 major capsid protein, partial [Largemouth bass virus]	100	46	1.47E-24	0
k141_159064_flag1_multi2.2204_len631	W5-1	631	QJE49078.1 putative DNA methyltransferase [Largemouth bass virus]	100	46	1.84E-24	4
k141_131166_flag1_multi2.6945_len691	W5-1	691	UUY86266.1 hypothetical protein [Largemouth bass virus]	88.1	59	2.01E-24	14.4
k141_151418_flag1_multi1.0000_len269	W5-1	269	WAK75094.1 hypothetical protein [Mandarin fish ranavirus]	100	41	2.01E-24	0
k141_69568_flag1_multi4.8067_len617	W5-1	617	CBW45583.1 ICP 46 homolog, partial [Doctor fish virus]	97.9	48	2.12E-24	4
k141_32209_flag1_multi2.8514_len437	W5-1	437	AYV88120.1 putative p31K protein [Mandarin fish ranavirus]	70.5	78	2.25E-24	4
k141_108613_flag1_multi39.2249_len590	W5-1	590	AYV88191.1 hypothetical protein [Mandarin fish ranavirus]	100	46	2.67E-24	82
k141_131353_flag0_multi1248.4265_len685	W1-1	685	UUY86227.1 hypothetical protein [Largemouth bass virus]	87.8	49	2.79E-24	4
k141_125453_flag1_multi5.6858_len507	W5-1	507	XRB52842.1 transcription factor TFIIB cyclin-like domain-containing protein, partial [Largemouth bass virus]	100	46	2.94E-24	2
k141_5070_flag1_multi4.7588_len1368	W5-1	1368	UUY86197.1 hypothetical protein [Largemouth bass virus]	95.3	64	3.84E-24	32
k141_74904_flag1_multi1.0000_len499	W1-1	499	WAK75073.1 hypothetical protein [Mandarin fish ranavirus]	100	46	4.16E-24	4
k141_40092_flag1_multi4.5556_len357	W5-1	357	AYV88191.1 hypothetical protein [Mandarin fish ranavirus]	100	46	4.69E-24	0
k141_113299_flag0_multi1.0000_len279	W5-1	279	WAK75107.1 hypothetical protein [Mandarin fish ranavirus]	100	44	4.89E-24	0
k141_68654_flag0_multi12.4751_len644	W4-2	644	AYV88172.2 hypothetical protein [Mandarin fish ranavirus]	92.2	167	4.91E-24	13
k141_73067_flag1_multi1.5298_len560	W5-1	560	AYV88176.1 putative tumor necrosis factor receptor [Mandarin fish ranavirus]	100	46	5.17E-24	3
k141_41922_flag1_multi1.0000_len279	W1-1	279	QJE49146.1 putative thiol oxidoreductase [Largemouth bass virus]	87	54	5.21E-24	0
k141_72440_flag1_multi1.0000_len308	W5-1	308	UUY86235.1 hypothetical protein [Largemouth bass virus]	100	46	5.42E-24	0
k141_34557_flag1_multi1.0000_len405	W5-1	405	AYV88191.1 hypothetical protein [Mandarin fish ranavirus]	100	46	5.68E-24	2
k141_3091_flag1_multi4.8168_len1495	W4-1	1495	XPZ21295.1 putative RNAseIII [Mandarin fish ranavirus]	69.3	88	5.70E-24	21
k141_121564_flag1_multi15.6748_len922	W2-3	922	UUY86241.1 hypothetical protein [Largemouth bass virus]	100	46	5.75E-24	49
k141_131264_flag1_multi1.0000_len269	W5-1	269	UUY86241.1 hypothetical protein [Largemouth bass virus]	100	39	5.75E-24	0
k141_12672_flag1_multi7.8169_len982	W5-1	982	AYV88191.1 hypothetical protein [Mandarin fish ranavirus]	100	46	6.01E-24	19
k141_94879_flag0_multi17.5057_len841	W1-1	841	XRB52785.1 hypothetical protein LMBV_28 [Largemouth bass virus]	100	46	6.34E-24	21
k141_143126_flag1_multi3.8985_len791	W5-1	791	UUY86199.1 hypothetical protein [Largemouth bass virus]	100	46	6.34E-24	9
k141_58772_flag1_multi8.8692_len569	W1-1	569	UUY86199.1 hypothetical protein [Largemouth bass virus]	100	46	6.82E-24	14
k141_29715_flag1_multi7.0421_len1163	W6-1	1163	WAK75094.1 hypothetical protein [Mandarin fish ranavirus]	100	46	7.22E-24	20
k141_53177_flag1_multi2.4590_len446	W5-1	446	UUY86235.1 hypothetical protein [Largemouth bass virus]	83.1	59	7.24E-24	2
k141_197906_flag1_multi7.9472_len3192	W4-3	3192	AYV88172.2 hypothetical protein [Mandarin fish ranavirus]	92.9	170	8.09E-24	85
k141_164336_flag0_multi1.0000_len334	W5-1	334	AFD96401.1 major capsid protein, partial [Largemouth bass virus]	100	46	8.13E-24	1
k141_102173_flag1_multi1.9010_len434	W5-1	434	UUY86235.1 hypothetical protein [Largemouth bass virus]	100	47	8.89E-24	2
k141_42653_flag1_multi1.0000_len277	W5-1	277	UUY86192.1 putative myristylated membrane protein [Largemouth bass virus]	100	46	9.89E-24	0
k141_135165_flag1_multi1.0000_len363	W1-1	363	UUY86235.1 hypothetical protein [Largemouth bass virus]	78.5	65	1.06E-23	0
k141_133299_flag0_multi1.6756_len440	W1-1	440	WAK75094.1 hypothetical protein [Mandarin fish ranavirus]	80	55	1.14E-23	1
k141_35662_flag1_multi15.5312_len877	W2-1	877	UUY86199.1 hypothetical protein [Largemouth bass virus]	100	46	1.22E-23	50

k141_16131_flag1_multi2.8701_len495	W4-1	495	AYV88120.1 putative p31K protein [Mandarin fish ranavirus]	100	48	1.23E-23	15
k141_154686_flag0_multi19.3444_len1764	W5-1	1764	AFD96401.1 major capsid protein, partial [Largemouth bass virus]	100	51	1.29E-23	97.96
k141_52324_flag1_multi1.0000_len280	W5-1	280	UUY86263.1 hypothetical protein [Largemouth bass virus]	100	46	1.30E-23	0
k141_184_flag1_multi2.6613_len389	W5-1	389	AYV88191.1 hypothetical protein [Mandarin fish ranavirus]	100	44	1.35E-23	1
k141_82495_flag0_multi1.0000_len277	W5-1	277	QYU76034.1 putative myristylated membrane protein, partial [Koi ranavirus]	89.5	57	1.38E-23	1
k141_18245_flag1_multi5.0000_len517	W6-1	517	AYV88120.1 putative p31K protein [Mandarin fish ranavirus]	89.3	56	1.49E-23	9
k141_81563_flag0_multi1.0000_len282	W1-1	282	AIG51690.1 major capsid protein, partial [Koi ranavirus]	95.9	49	1.56E-23	2
k141_98926_flag1_multi6.5577_len453	W4-1	453	AYV88155.1 hypothetical protein [Mandarin fish ranavirus]	100	46	1.58E-23	0
k141_96640_flag1_multi1.0000_len280	W4-1	280	WEI29006.1 putative 2-cysteine adaptor domain protein [Largemouth bass virus]	78.3	69	1.75E-23	0
k141_91948_flag1_multi2.9247_len566	W6-1	566	WEI29006.1 putative 2-cysteine adaptor domain protein [Largemouth bass virus]	100	44	1.79E-23	3
k141_50773_flag1_multi2.8969_len626	W1-1	626	WEI28972.1 CTD-phosphotransferase [Largemouth bass virus]	100	46	1.81E-23	8
k141_5029_flag1_multi3.7125_len381	W5-1	381	QJE49215.1 putative orf58-like protein [Largemouth bass virus]	100	44	1.95E-23	4
k141_94017_flag0_multi1.0000_len276	W2-1	276	UUY86235.1 hypothetical protein [Largemouth bass virus]	97.9	47	1.98E-23	0
k141_35845_flag1_multi1.0000_len506	W3-2	506	UUY86235.1 hypothetical protein [Largemouth bass virus]	100	46	1.98E-23	0
k141_15533_flag1_multi1.0000_len372	W1-1	372	WAK75106.1 hypothetical protein [Mandarin fish ranavirus]	75.7	70	2.11E-23	0
k141_36558_flag1_multi1.0000_len316	W5-1	316	AYV88191.1 hypothetical protein [Mandarin fish ranavirus]	97.7	43	2.17E-23	0
k141_82698_flag0_multi7.7828_len1591	W5-1	1591	WAK75112.1 putative LITAF PIG7 possible membrane associated motif in LPS-induced tumor necrosis factor alpha factor [Mandarin fish ranavirus]	95.8	48	2.35E-23	53
k141_9380_flag1_multi1.0000_len432	W5-1	432	QJE49128.1 hypothetical protein LMBV_065 [Largemouth bass virus]	100	46	2.50E-23	0
k141_152259_flag1_multi1.9716_len1021	W5-1	1021	WEI29006.1 putative 2-cysteine adaptor domain protein [Largemouth bass virus]	100	46	2.56E-23	18
k141_75588_flag0_multi15.5977_len1993	W5-1	1993	UUY86241.1 hypothetical protein [Largemouth bass virus]	100	46	2.70E-23	127.71
k141_107154_flag0_multi1.0000_len208	W1-1	208	UUY86197.1 hypothetical protein [Largemouth bass virus]	91.9	74	2.71E-23	3
k141_12140_flag1_multi9.8087_len1223	W1-1	1223	QJE49149.1 putative immediate early protein ICP-46 [Largemouth bass virus]	100	46	2.74E-23	36
k141_51991_flag0_multi60.3407_len913	W1-1	913	AYV88191.1 hypothetical protein [Mandarin fish ranavirus]	76.6	64	2.86E-23	76
k141_163272_flag0_multi1.0000_len413	W5-1	413	QJE49077.1 hypothetical protein LMBV_014 [Largemouth bass virus]	100	43	3.03E-23	97
k141_50367_flag1_multi21.4896_len717	W5-1	717	WEI29006.1 putative 2-cysteine adaptor domain protein [Largemouth bass virus]	94	50	3.22E-23	10
k141_126924_flag0_multi2.8945_len615	W1-1	615	AFD96401.1 major capsid protein, partial [Largemouth bass virus]	100	47	3.24E-23	7
k141_29759_flag1_multi22.0000_len1645	W5-1	1645	UUY86241.1 hypothetical protein [Largemouth bass virus]	95.9	49	3.24E-23	121
k141_123083_flag1_multi3.4458_len870	W5-1	870	QJE49065.1 hypothetical protein LMBV_002 [Largemouth bass virus]	100	47	3.31E-23	6
k141_135314_flag1_multi2.4572_len679	W5-1	679	QJE49080.1 hypothetical protein LMBV_017 [Largemouth bass virus]	58.2	91	3.46E-23	6
k141_99572_flag1_multi1.0000_len370	W4-1	370	QJE49215.1 putative orf58-like protein [Largemouth bass virus]	100	46	3.52E-23	8
k141_159951_flag0_multi1.0000_len264	W5-1	264	AYV88134.2 putative tyrosine kinase [Mandarin fish ranavirus]	86.8	53	3.52E-23	0
k141_96993_flag1_multi3.4721_len535	W5-1	535	AYV88155.1 hypothetical protein [Mandarin fish ranavirus]	76.6	64	3.79E-23	6
k141_8800_flag1_multi2.6078_len373	W1-1	373	AFD96401.1 major capsid protein, partial [Largemouth bass virus]	93.9	49	3.85E-23	11
k141_14688_flag1_multi1.0000_len518	W1-1	518	UUY86241.1 hypothetical protein [Largemouth bass virus]	97.7	43	3.89E-23	7
k141_26442_flag1_multi1.0000_len428	W5-1	428	AYV88211.1 hypothetical protein [Mandarin fish ranavirus]	94.5	55	3.92E-23	1
k141_35247_flag1_multi1.0000_len390	W4-1	390	AYV88120.1 putative p31K protein [Mandarin fish ranavirus]	100	47	4.01E-23	354.92
k141_168940_flag0_multi1.0000_len279	W5-1	279	QJE49215.1 putative orf58-like protein [Largemouth bass virus]	97.9	48	4.44E-23	0
k141_68888_flag1_multi1.6978_len280	W5-1	280	QJE49096.1 putative D5 family NTPase/ATPase [Largemouth bass virus]	86	57	4.49E-23	0
k141_59301_flag0_multi18.0705_len297	W1-1	297	QYU76034.1 putative myristylated membrane protein, partial [Koi ranavirus]	100	46	4.64E-23	0

k141_74124_flag0_multi1.0000_len347	W4-1	347	UUY86235.1 hypothetical protein [Largemouth bass virus]	92.3	52	4.94E-23	21
k141_43565_flag0_multi5.4843_len810	W5-1	810	AYV88191.1 hypothetical protein [Mandarin fish ranavirus]	100	44	5.16E-23	16
k141_12341_flag0_multi1.0000_len278	W4-1	278	AYV88120.1 putative p31K protein [Mandarin fish ranavirus]	95.7	47	5.32E-23	4
k141_43967_flag0_multi1.0000_len336	W5-1	336	WAK75068.1 hypothetical protein [Mandarin fish ranavirus]	97.8	46	5.58E-23	0
k141_87618_flag0_multi1.0000_len774	W5-1	774	WAK75112.1 putative LITAF PIG7 possible membrane associated motif in LPS-induced tumor necrosis factor alpha factor [Mandarin fish ranavirus]	97.9	47	5.82E-23	17
k141_54522_flag1_multi1.0000_len280	W5-1	280	UUY86235.1 hypothetical protein [Largemouth bass virus]	95.9	49	6.02E-23	0
k141_132256_flag1_multi2.8827_len499	W1-1	499	QJE49149.1 putative immediate early protein ICP-46 [Largemouth bass virus]	100	46	6.36E-23	5
k141_24308_flag0_multi1.0000_len305	W4-1	305	UUY86199.1 hypothetical protein [Largemouth bass virus]	93.2	44	6.41E-23	0
k141_66722_flag1_multi10.5330_len899	W5-1	899	QJE49077.1 hypothetical protein LMBV_014 [Largemouth bass virus]	100	46	6.43E-23	26
k141_36581_flag1_multi3.8889_len762	W1-1	762	AYV88191.1 hypothetical protein [Mandarin fish ranavirus]	97.8	46	6.96E-23	9
k141_159139_flag1_multi1.0000_len269	W5-1	269	UUY86235.1 hypothetical protein [Largemouth bass virus]	100	46	7.27E-23	0
k141_133371_flag1_multi1.0000_len280	W5-1	280	WAK75136.1 hypothetical protein [Mandarin fish ranavirus]	100	46	7.64E-23	0
k141_47002_flag1_multi6.6167_len501	W4-1	501	AYV88134.2 putative tyrosine kinase [Mandarin fish ranavirus]	100	47	7.96E-23	7
k141_1855_flag1_multi1.0000_len380	W1-1	380	AAF64582.1 capsid protein, partial [Guppyfish iridovirus]	90.6	53	8.06E-23	1
k141_101369_flag0_multi30.9148_len493	W5-1	493	QJE49119.1 hypothetical protein LMBV_056 [Largemouth bass virus]	100	46	8.14E-23	11
k141_91018_flag1_multi3.6174_len1223	W1-1	1223	QJE49114.1 hypothetical protein LMBV_051 [Largemouth bass virus]	100	46	8.50E-23	15
k141_66567_flag1_multi4.8375_len707	W1-1	707	AYV88191.1 hypothetical protein [Mandarin fish ranavirus]	95.9	49	8.76E-23	11
k141_17675_flag1_multi1.0000_len280	W2-1	280	AYV88126.1 putative immediate early protein ICP-18 [Mandarin fish ranavirus]	100	46	8.80E-23	0
k141_104896_flag0_multi1.0000_len290	W4-1	290	AIG51690.1 major capsid protein, partial [Koi ranavirus]	97.9	47	9.21E-23	60
k141_158123_flag0_multi2.9674_len1369	W5-1	1369	UUY86199.1 hypothetical protein [Largemouth bass virus]	88.7	53	1.02E-22	14
k141_18336_flag0_multi1.0000_len405	W5-1	405	AYV88183.1 hypothetical protein [Mandarin fish ranavirus]	100	46	1.04E-22	0
k141_154454_flag1_multi1.0000_len280	W5-1	280	QJE49066.1 hypothetical protein LMBV_003 [Largemouth bass virus]	100	46	1.08E-22	0
k141_50478_flag0_multi1.0000_len374	W1-1	374	AYV88134.2 putative tyrosine kinase [Mandarin fish ranavirus]	100	46	1.10E-22	13.33
k141_108852_flag0_multi1.0000_len376	W1-1	376	AFD96401.1 major capsid protein, partial [Largemouth bass virus]	79.7	64	1.13E-22	1
k141_75449_flag0_multi1.0000_len278	W1-1	278	UUY86235.1 hypothetical protein [Largemouth bass virus]	100	46	1.15E-22	1
k141_139321_flag0_multi1.8659_len305	W5-1	305	AYV88159.1 hypothetical protein [Mandarin fish ranavirus]	100	46	1.16E-22	0
k141_108045_flag1_multi1.0000_len292	W1-1	292	QYU76034.1 putative myristylated membrane protein, partial [Koi ranavirus]	100	48	1.19E-22	0
k141_65065_flag1_multi1.0000_len504	W5-1	504	AYV88176.1 putative tumor necrosis factor receptor [Mandarin fish ranavirus]	100	43	1.20E-22	2
k141_95380_flag1_multi1.0000_len280	W6-1	280	QJE49104.1 hypothetical protein LMBV_041 [Largemouth bass virus]	100	46	1.26E-22	0
k141_4717_flag1_multi1.0000_len387	W5-1	387	AYV88134.2 putative tyrosine kinase [Mandarin fish ranavirus]	94.1	51	1.33E-22	0
k141_90620_flag1_multi1.0000_len387	W6-2	387	UUY86257.1 hypothetical protein [Largemouth bass virus]	87.5	56	1.33E-22	24
k141_16270_flag1_multi9.0000_len801	W4-1	801	WAK75112.1 putative LITAF PIG7 possible membrane associated motif in LPS-induced tumor necrosis factor alpha factor [Mandarin fish ranavirus]	100	44	1.39E-22	139
k141_80610_flag1_multi2.8950_len579	W5-1	579	AYV88191.1 hypothetical protein [Mandarin fish ranavirus]	97.9	47	1.48E-22	2
k141_2348_flag1_multi2.4727_len856	W5-1	856	QJE49215.1 putative orf58-like protein [Largemouth bass virus]	100	46	1.60E-22	6
k141_96195_flag1_multi1.0000_len708	W5-1	708	UUY86251.1 hypothetical protein [Largemouth bass virus]	100	46	1.62E-22	7
k141_96648_flag1_multi4.0864_len662	W4-1	662	AYV88134.2 putative tyrosine kinase [Mandarin fish ranavirus]	83.1	65	1.70E-22	9
k141_127361_flag1_multi2.8982_len593	W5-1	593	AYV88191.1 hypothetical protein [Mandarin fish ranavirus]	87	54	1.72E-22	9
k141_160390_flag1_multi1.8978_len366	W5-1	366	UUY86235.1 hypothetical protein [Largemouth bass virus]	100	46	1.73E-22	1

k141_79483_flag1_multi2.8783_len519	W6-1	519	QYU76034.1 putative myristylated membrane protein, partial [Koi ranavirus]	100	46	1.76E-22	4
k141_125302_flag1_multi2.8655_len483	W5-1	483	AIG51690.1 major capsid protein, partial [Koi ranavirus]	95.9	49	1.77E-22	3
k141_142502_flag0_multi1.0000_len330	W5-1	330	AFD96401.1 major capsid protein, partial [Largemouth bass virus]	71.6	67	1.78E-22	0
k141_23666_flag1_multi15.7169_len579	W4-1	579	AFD96401.1 major capsid protein, partial [Largemouth bass virus]	97.9	48	1.79E-22	11
k141_25661_flag0_multi1.0000_len374	W5-1	374	AIG51690.1 major capsid protein, partial [Koi ranavirus]	100	47	1.90E-22	0
k141_45677_flag1_multi1.0000_len311	W5-1	311	AYV88173.2 putative ribonucleotide reductase beta subunit [Mandarin fish ranavirus]	85.5	55	1.94E-22	0
k141_22109_flag0_multi9.4314_len345	W4-1	345	AYV88159.1 hypothetical protein [Mandarin fish ranavirus]	100	47	1.96E-22	3
k141_168257_flag1_multi1.0000_len276	W5-1	276	WEI28972.1 CTD-phosphotransferase [Largemouth bass virus]	100	47	1.98E-22	0.95
k141_91079_flag1_multi14.7007_len1217	W5-1	1217	AYV88191.1 hypothetical protein [Mandarin fish ranavirus]	97.9	47	2.12E-22	62
k141_65644_flag1_multi3.8493_len599	W4-1	599	AFD96401.1 major capsid protein, partial [Largemouth bass virus]	100	46	2.20E-22	6
k141_101527_flag0_multi1.0000_len279	W4-1	279	AIG51690.1 major capsid protein, partial [Koi ranavirus]	100	47	2.21E-22	1
k141_101527_flag0_multi1.0000_len279	W4-1	279	AIG51690.1 major capsid protein, partial [Koi ranavirus]	100	47	2.21E-22	0
k141_161467_flag1_multi1.0000_len279	W5-1	279	AYV88191.1 hypothetical protein [Mandarin fish ranavirus]	97.8	46	2.22E-22	0
k141_100350_flag0_multi1.0000_len279	W5-1	279	UUY86235.1 hypothetical protein [Largemouth bass virus]	100	46	2.30E-22	0
k141_20890_flag0_multi1.0000_len279	W5-1	279	UUY86235.1 hypothetical protein [Largemouth bass virus]	100	46	2.30E-22	0
k141_146336_flag1_multi2.9103_len1033	W5-1	1033	AYV88191.1 hypothetical protein [Mandarin fish ranavirus]	97.8	46	2.32E-22	11
k141_93020_flag0_multi1.9499_len580	W5-1	580	UUY86199.1 hypothetical protein [Largemouth bass virus]	60.5	86	2.38E-22	3
k141_116367_flag1_multi4.7996_len650	W5-1	650	QIZ30887.1 major capsid protein, partial [Largemouth bass virus]	100	46	2.45E-22	7
k141_138126_flag0_multi1.0000_len682	W5-1	682	XPZ21295.1 putative RNAseIII [Mandarin fish ranavirus]	76.6	64	2.48E-22	2
k141_14590_flag0_multi1.0000_len401	W1-1	401	UUY86235.1 hypothetical protein [Largemouth bass virus]	97.9	47	2.65E-22	0
k141_19812_flag0_multi33.5131_len1903	W5-1	1903	AYV88191.1 hypothetical protein [Mandarin fish ranavirus]	74.2	66	2.68E-22	211
k141_35856_flag1_multi1.0000_len662	W5-1	662	QYU76034.1 putative myristylated membrane protein, partial [Koi ranavirus]	100	46	2.85E-22	10
k141_109485_flag1_multi10.7486_len1223	W2-2	1223	WAK75112.1 putative LITAF PIG7 possible membrane associated motif in LPS-induced tumor necrosis factor alpha factor [Mandarin fish ranavirus]	71.6	67	2.90E-22	45
k141_108930_flag0_multi172.0268_len290	W5-1	290	QYU76034.1 putative myristylated membrane protein, partial [Koi ranavirus]	100	46	3.03E-22	1
k141_3174_flag1_multi6.7915_len803	W2-1	803	UUY86235.1 hypothetical protein [Largemouth bass virus]	100	48	3.11E-22	16
k141_83135_flag1_multi2.9257_len760	W1-1	760	QIZ30887.1 major capsid protein, partial [Largemouth bass virus]	100	46	3.18E-22	9
k141_105610_flag1_multi5.8477_len896	W2-3	896	QJE49125.1 hypothetical protein LMBV_062 [Largemouth bass virus]	98.1	54	3.27E-22	18
k141_21169_flag1_multi5.6361_len457	W2-1	457	AYV88191.1 hypothetical protein [Mandarin fish ranavirus]	97.8	46	3.37E-22	4
k141_125300_flag1_multi3.4303_len729	W5-1	729	AYV88183.1 hypothetical protein [Mandarin fish ranavirus]	98	49	3.38E-22	5
k141_10698_flag1_multi1.8515_len1097	W4-1	1097	UUY86195.1 hypothetical protein [Largemouth bass virus]	92.3	52	3.46E-22	16
k141_94551_flag0_multi1.0000_len340	W5-1	340	UUY86235.1 hypothetical protein [Largemouth bass virus]	100	46	3.48E-22	2
k141_9915_flag1_multi1.0000_len531	W5-1	531	WEI29006.1 putative 2-cysteine adaptor domain protein [Largemouth bass virus]	95.7	47	3.61E-22	19
k141_28189_flag0_multi1.0000_len279	W5-1	279	WEI28972.1 CTD-phosphotransferase [Largemouth bass virus]	92.2	51	3.75E-22	0
k141_104014_flag1_multi2.9184_len705	W6-1	705	UUY86235.1 hypothetical protein [Largemouth bass virus]	66.2	77	3.78E-22	4
k141_106483_flag0_multi3.6786_len561	W5-1	561	UUY86235.1 hypothetical protein [Largemouth bass virus]	97.9	47	3.81E-22	1
k141_54376_flag1_multi1.9404_len527	W4-1	527	QYU76035.1 DNA polymerase, partial [Koi ranavirus]	100	46	3.87E-22	2
k141_36748_flag1_multi1.0000_len275	W4-1	275	AYV88120.1 putative p31K protein [Mandarin fish ranavirus]	93.9	49	3.96E-22	139
k141_3551_flag1_multi3.3323_len797	W4-1	797	UUY86199.1 hypothetical protein [Largemouth bass virus]	100	44	3.99E-22	18
k141_29367_flag1_multi1.6429_len589	W6-1	589	AFD96401.1 major capsid protein, partial [Largemouth bass virus]	87	54	4.00E-22	3

k141_95609_flag1_multi1.0000_len279	W6-1	279	AYV88120.1 putative p31K protein [Mandarin fish ranavirus]	90.2	51	4.11E-22	0
k141_55465_flag1_multi15.9300_len484	W6-1	484	QJE49119.1 hypothetical protein LMBV_056 [Largemouth bass virus]	65.9	82	4.17E-22	26
k141_3808_flag1_multi1.0000_len338	W5-1	338	WEI29006.1 putative 2-cysteine adaptor domain protein [Largemouth bass virus]	95.7	47	4.44E-22	3
k141_94187_flag1_multi3.1073_len495	W4-1	495	WEI29006.1 putative 2-cysteine adaptor domain protein [Largemouth bass virus]	100	46	4.49E-22	2
k141_72495_flag1_multi1.9510_len610	W5-1	610	QYU76034.1 putative myristylated membrane protein, partial [Koi ranavirus]	97.9	47	4.58E-22	6
k141_143602_flag1_multi2.8516_len451	W5-1	451	AIG51690.1 major capsid protein, partial [Koi ranavirus]	95.9	49	4.74E-22	1
k141_38550_flag1_multi1.0000_len280	W4-1	280	QYU76034.1 putative myristylated membrane protein, partial [Koi ranavirus]	100	46	4.97E-22	4.51
k141_97022_flag0_multi1.0000_len278	W5-1	278	QYU76034.1 putative myristylated membrane protein, partial [Koi ranavirus]	100	46	4.97E-22	9
k141_34161_flag0_multi1.0000_len432	W5-1	432	AYV88120.1 putative p31K protein [Mandarin fish ranavirus]	100	47	5.03E-22	15.01
k141_99760_flag1_multi1.0000_len276	W4-1	276	QJE49104.1 hypothetical protein LMBV_041 [Largemouth bass virus]	97.9	47	5.10E-22	3
k141_9506_flag1_multi6.0848_len471	W1-1	471	UUY86241.1 hypothetical protein [Largemouth bass virus]	100	40	5.15E-22	17
k141_49681_flag0_multi3.7850_len448	W5-1	448	AFD96401.1 major capsid protein, partial [Largemouth bass virus]	100	45	5.17E-22	3
k141_10120_flag1_multi6.5701_len462	W2-1	462	AIG51690.1 major capsid protein, partial [Koi ranavirus]	100	48	5.42E-22	10
k141_3368_flag1_multi1.0000_len353	W5-1	353	AIG51690.1 major capsid protein, partial [Koi ranavirus]	95.8	48	5.51E-22	0
k141_61072_flag1_multi1.9644_len590	W5-1	590	WEI28974.1 hypothetical protein [Largemouth bass virus]	93.8	48	5.72E-22	3
k141_49065_flag1_multi1.0000_len444	W6-1	444	AYV88120.1 putative p31K protein [Mandarin fish ranavirus]	97.9	47	5.76E-22	0
k141_122591_flag1_multi1.0000_len757	W5-1	757	QJE49215.1 putative orf58-like protein [Largemouth bass virus]	97.9	47	5.78E-22	11
k141_19474_flag0_multi1.9027_len398	W5-1	398	AFD96401.1 major capsid protein, partial [Largemouth bass virus]	97.8	46	5.78E-22	2
k141_239378_flag1_multi6.8475_len967	W4-3	967	UUY86199.1 hypothetical protein [Largemouth bass virus]	100	45	6.33E-22	23
k141_92333_flag1_multi174.2957_len1629	W5-1	1629	WAK75079.1 hypothetical protein [Mandarin fish ranavirus]	92	50	6.59E-22	700.68
k141_138522_flag1_multi8.8630_len1484	W5-1	1484	AYV88191.1 hypothetical protein [Mandarin fish ranavirus]	97.9	48	6.94E-22	42
k141_57199_flag1_multi1.4310_len373	W5-1	373	QJE49066.1 hypothetical protein LMBV_003 [Largemouth bass virus]	85.2	54	6.98E-22	1
k141_94390_flag0_multi9.4000_len1146	W5-1	1146	QJE49077.1 hypothetical protein LMBV_014 [Largemouth bass virus]	90.6	53	7.41E-22	33
k141_161788_flag0_multi3.4981_len408	W5-1	408	AYV88191.1 hypothetical protein [Mandarin fish ranavirus]	100	46	7.87E-22	4
k141_92757_flag0_multi16.8085_len329	W5-1	329	AYV88120.1 putative p31K protein [Mandarin fish ranavirus]	100	46	7.88E-22	0
k141_48182_flag1_multi2.8844_len539	W4-1	539	QYU76034.1 putative myristylated membrane protein, partial [Koi ranavirus]	100	46	8.03E-22	5
k141_88255_flag1_multi5.6788_len499	W4-1	499	AIG51690.1 major capsid protein, partial [Koi ranavirus]	100	46	8.06E-22	7
k141_79804_flag1_multi4.9240_len944	W4-1	944	QIZ30887.1 major capsid protein, partial [Largemouth bass virus]	100	46	8.32E-22	18
k141_101922_flag1_multi3.7957_len988	W5-1	988	QYU76036.1 helicase-like protein, partial [Koi ranavirus]	98.2	55	8.93E-22	17
k141_5985_flag1_multi1.0000_len422	W5-1	422	WEI29006.1 putative 2-cysteine adaptor domain protein [Largemouth bass virus]	100	46	9.17E-22	3
k141_107147_flag0_multi89.3862_len677	W1-1	677	XRB52842.1 transcription factor TFIIB cyclin-like domain-containing protein, partial [Largemouth bass virus]	97.9	47	9.33E-22	213
k141_6148_flag0_multi14.6412_len2106	W1-1	2106	AIG51690.1 major capsid protein, partial [Koi ranavirus]	88.9	63	9.40E-22	1374.02
k141_145088_flag0_multi1.0000_len280	W5-1	280	UUY86235.1 hypothetical protein [Largemouth bass virus]	100	46	9.46E-22	0
k141_151461_flag1_multi1.0000_len279	W5-1	279	QYU76034.1 putative myristylated membrane protein, partial [Koi ranavirus]	100	46	9.51E-22	0
k141_67381_flag1_multi2.7469_len623	W1-1	623	QJE49146.1 putative thiol oxidoreductase [Largemouth bass virus]	100	46	9.78E-22	9
k141_5505_flag0_multi1.0000_len582	W1-1	582	AIG51690.1 major capsid protein, partial [Koi ranavirus]	97.9	48	1.00E-21	32
k141_23153_flag1_multi1.0000_len655	W2-3	655	AYV88169.1 hypothetical protein [Mandarin fish ranavirus]	97.9	48	1.01E-21	8
k141_46425_flag1_multi6.3658_len562	W5-1	562	AYV88120.1 putative p31K protein [Mandarin fish ranavirus]	88.7	53	1.02E-21	15
k141_115107_flag1_multi9.4732_len589	W5-1	589	AIG51690.1 major capsid protein, partial [Koi ranavirus]	82	61	1.03E-21	27

k141_137877_flag1_multi10.2075_len406	W5-1	406	WHU98721.1 MCP, partial [Hybrid snakehead ranavirus]	100	46	1.06E-21	1
k141_77283_flag1_multi1.0000_len269	W5-1	269	AXB27397.1 major capsid protein, partial [Ranavirus sp.]	95.7	46	1.09E-21	0
k141_82820_flag0_multi1.0000_len279	W5-1	279	QJE49153.1 hypothetical protein LMBV_004 [Largemouth bass virus]	97.9	47	1.13E-21	0
k141_108358_flag1_multi2.8757_len511	W5-1	511	QJE49097.1 hypothetical protein LMBV_034 [Largemouth bass virus]	93.9	49	1.16E-21	6
k141_89629_flag1_multi6.8416_len482	W1-1	482	UUY86192.1 putative myristylated membrane protein [Largemouth bass virus]	100	42	1.24E-21	7
k141_68650_flag1_multi4.8680_len838	W5-1	838	QYU76034.1 putative myristylated membrane protein, partial [Koi ranavirus]	100	46	1.27E-21	16
k141_112452_flag1_multi8.8875_len1563	W2-1	1563	QYU76035.1 DNA polymerase, partial [Koi ranavirus]	98	50	1.31E-21	55
k141_14419_flag1_multi9.4570_len2476	W5-1	2476	QJE49077.1 hypothetical protein LMBV_014 [Largemouth bass virus]	97.8	46	1.37E-21	74
k141_95499_flag1_multi1.0000_len280	W1-1	280	XRB52768.1 P31K protein, partial [Largemouth bass virus]	97.8	46	1.45E-21	0
k141_33355_flag1_multi1.0000_len434	W4-1	434	AYV88191.1 hypothetical protein [Mandarin fish ranavirus]	97.8	46	1.46E-21	15.1
k141_15503_flag0_multi1.0000_len363	W5-1	363	WEI29006.1 putative 2-cysteine adaptor domain protein [Largemouth bass virus]	100	45	1.53E-21	1
k141_76344_flag1_multi27.6336_len755	W5-1	755	AYV88120.1 putative p31K protein [Mandarin fish ranavirus]	87.3	55	1.53E-21	121
k141_53812_flag1_multi2.7074_len640	W2-1	640	XRL22821.1 hypothetical protein [Siniperca chuatsi ranavirus]	89.3	56	1.56E-21	3
k141_66974_flag1_multi7.9047_len1831	W5-1	1831	XRB52842.1 transcription factor TFIIB cyclin-like domain-containing protein, partial [Largemouth bass virus]	97.9	47	1.57E-21	41
k141_102732_flag1_multi1.0000_len278	W4-1	278	AYV88120.1 putative p31K protein [Mandarin fish ranavirus]	93.9	49	1.61E-21	11
k141_23617_flag1_multi2.5211_len734	W5-1	734	QJE49137.1 hypothetical protein LMBV_074 [Largemouth bass virus]	95.8	48	1.62E-21	5
k141_100770_flag1_multi2.0000_len572	W1-1	572	UUY86192.1 putative myristylated membrane protein [Largemouth bass virus]	95.7	47	1.65E-21	3
k141_45220_flag0_multi2.7606_len517	W5-1	517	WXI69525.1 caspase recruitment domain protein [Largemouth bass virus]	95.8	48	1.67E-21	5
k141_129068_flag1_multi1.0000_len352	W5-1	352	QJE49104.1 hypothetical protein LMBV_041 [Largemouth bass virus]	97.9	47	1.85E-21	5
k141_109798_flag1_multi3.4627_len932	W5-1	932	QYU76034.1 putative myristylated membrane protein, partial [Koi ranavirus]	100	46	1.87E-21	10
k141_82460_flag1_multi3.7621_len431	W4-1	431	WEI29006.1 putative 2-cysteine adaptor domain protein [Largemouth bass virus]	100	46	1.90E-21	7
k141_66672_flag1_multi3.0841_len652	W5-1	652	UUY86221.1 hypothetical protein [Largemouth bass virus]	65.1	83	2.00E-21	5
k141_36147_flag0_multi41.8846_len297	W5-1	297	QJE49153.1 hypothetical protein LMBV_004 [Largemouth bass virus]	92.2	51	2.01E-21	41
k141_18887_flag1_multi4.7942_len588	W4-1	588	WEI28972.1 CTD-phosphotransferase [Largemouth bass virus]	100	47	2.05E-21	8
k141_39131_flag0_multi1.0000_len451	W2-1	451	AFD96401.1 major capsid protein, partial [Largemouth bass virus]	88.2	51	2.13E-21	2
k141_19588_flag1_multi1.0000_len513	W5-1	513	UUY86258.1 hypothetical protein [Largemouth bass virus]	88.9	54	2.20E-21	1
k141_149209_flag1_multi1.0000_len307	W2-3	307	AIG51690.1 major capsid protein, partial [Koi ranavirus]	100	46	2.27E-21	1
k141_86101_flag1_multi7.7905_len1678	W5-1	1678	WAK75112.1 putative LITAF PIG7 possible membrane associated motif in LPS-induced tumor necrosis factor alpha factor [Mandarin fish ranavirus]	100	46	2.28E-21	43
k141_8349_flag1_multi1.0000_len323	W1-1	323	WEI29006.1 putative 2-cysteine adaptor domain protein [Largemouth bass virus]	100	46	2.36E-21	0
k141_6958_flag1_multi7.6873_len1014	W4-1	1014	WAK75114.1 hypothetical protein [Mandarin fish ranavirus]	100	46	2.40E-21	23
k141_42120_flag0_multi352.3462_len817	W2-2	817	AYV88120.1 putative p31K protein [Mandarin fish ranavirus]	85.5	55	2.46E-21	1578
k141_154855_flag0_multi1.0000_len325	W5-1	325	WEI28972.1 CTD-phosphotransferase [Largemouth bass virus]	100	46	2.49E-21	2
k141_57224_flag0_multi1.0000_len303	W4-1	303	WEI29006.1 putative 2-cysteine adaptor domain protein [Largemouth bass virus]	100	46	2.55E-21	0
k141_55157_flag1_multi1.0000_len329	W5-1	329	UVF58793.1 MAG: DNA polymerase [Halichoeres melanurus ranavirus]	97.9	47	2.58E-21	1
k141_33337_flag1_multi1.0000_len269	W1-1	269	QJE49104.1 hypothetical protein LMBV_041 [Largemouth bass virus]	97.9	47	2.60E-21	0
k141_110518_flag1_multi2.8921_len660	W4-2	660	UUY86235.1 hypothetical protein [Largemouth bass virus]	100	46	2.71E-21	5
k141_147399_flag1_multi6.0571_len631	W5-1	631	UUY86223.1 hypothetical protein [Largemouth bass virus]	76.6	64	2.74E-21	8
k141_28339_flag1_multi18.6487_len2242	W5-1	2242	UUY86199.1 hypothetical protein [Largemouth bass virus]	95.9	49	2.74E-21	148

k141_161137_flag1_multi1.0000_len379	W5-1	379	XPZ21295.1 putative RNAseIII [Mandarin fish ranavirus]	100	44	2.74E-21	1
k141_133033_flag0_multi1.0000_len270	W5-1	270	AIG51690.1 major capsid protein, partial [Koi ranavirus]	74.6	71	2.76E-21	0
k141_67565_flag1_multi20.3669_len975	W4-1	975	AYV88120.1 putative p31K protein [Mandarin fish ranavirus]	98	49	2.80E-21	81
k141_45940_flag0_multi44.2096_len618	W1-1	618	WAK75139.1 putative myristylated membrane protein [Mandarin fish ranavirus]	80.8	125	2.89E-21	457
k141_123398_flag0_multi16.8640_len560	W5-1	560	UUY86218.1 hypothetical protein [Largemouth bass virus]	85.2	54	2.94E-21	69
k141_43553_flag0_multi1.0000_len332	W4-1	332	AIG51690.1 major capsid protein, partial [Koi ranavirus]	95.9	49	3.08E-21	0
k141_42395_flag1_multi1.0000_len379	W5-1	379	UUY86235.1 hypothetical protein [Largemouth bass virus]	77.6	67	3.11E-21	0
k141_44649_flag0_multi1.0000_len279	W5-1	279	AIG51690.1 major capsid protein, partial [Koi ranavirus]	100	47	3.11E-21	1
k141_43602_flag1_multi28.7494_len568	W4-1	568	AIG51690.1 major capsid protein, partial [Koi ranavirus]	100	46	3.19E-21	60
k141_20708_flag1_multi1.9368_len505	W5-1	505	WHU98721.1 MCP, partial [Hybrid snakehead ranavirus]	100	46	3.25E-21	80
k141_27209_flag1_multi1.0000_len277	W5-1	277	WEI29006.1 putative 2-cysteine adaptor domain protein [Largemouth bass virus]	100	45	3.33E-21	3
k141_21224_flag1_multi1.0000_len279	W6-1	279	XRL22821.1 hypothetical protein [Siniperca chuatsi ranavirus]	92.2	51	3.36E-21	0
k141_8097_flag1_multi7.5695_len515	W4-1	515	AYV88120.1 putative p31K protein [Mandarin fish ranavirus]	92.3	52	3.37E-21	9
k141_74101_flag1_multi7.4958_len1458	W5-1	1458	QJE49081.1 hypothetical protein LMBV_018 [Largemouth bass virus]	96	50	3.49E-21	35
k141_154346_flag0_multi1.0000_len316	W5-1	316	AIG51690.1 major capsid protein, partial [Koi ranavirus]	100	46	3.54E-21	1
k141_94539_flag1_multi7.6454_len595	W4-1	595	QYU76034.1 putative myristylated membrane protein, partial [Koi ranavirus]	95.8	48	3.67E-21	8
k141_26105_flag1_multi7.7925_len917	W4-1	917	WEI29006.1 putative 2-cysteine adaptor domain protein [Largemouth bass virus]	90.7	54	3.68E-21	18
k141_31172_flag0_multi1.0000_len441	W5-1	441	AYV88176.1 putative tumor necrosis factor receptor [Mandarin fish ranavirus]	100	41	3.73E-21	5
k141_163799_flag1_multi2.4991_len684	W5-1	684	AFD96401.1 major capsid protein, partial [Largemouth bass virus]	100	45	3.79E-21	16
k141_77124_flag1_multi1.0000_len269	W5-1	269	AYV88120.1 putative p31K protein [Mandarin fish ranavirus]	100	43	3.97E-21	0
k141_77671_flag1_multi3.8421_len578	W4-1	578	WHA35533.1 putative DNA dependent RNA polymerase A subunit [Micropterus salmoides ranavirus]	81.7	60	4.09E-21	7
k141_114607_flag1_multi5.5023_len362	W5-1	362	QJE49104.1 hypothetical protein LMBV_041 [Largemouth bass virus]	100	46	4.12E-21	3
k141_131553_flag1_multi5.7935_len698	W5-1	698	UUY86195.1 hypothetical protein [Largemouth bass virus]	97.8	46	4.30E-21	17
k141_155402_flag0_multi1.0000_len281	W5-1	281	QJE49215.1 putative orf58-like protein [Largemouth bass virus]	100	40	4.30E-21	0
k141_80853_flag1_multi16.6089_len1082	W4-1	1082	XRB52842.1 transcription factor TFIIB cyclin-like domain-containing protein, partial [Largemouth bass virus]	100	46	4.34E-21	66
k141_214261_flag1_multi13.3944_len1353	W5-3	1353	WAK75072.1 hypothetical protein [Mandarin fish ranavirus]	100	49	4.43E-21	35
k141_62374_flag1_multi3.0116_len571	W5-1	571	AIG51690.1 major capsid protein, partial [Koi ranavirus]	100	46	4.57E-21	4
k141_86674_flag1_multi3.3052_len508	W5-1	508	AYV88191.1 hypothetical protein [Mandarin fish ranavirus]	88.5	52	4.63E-21	5
k141_104006_flag0_multi1.3692_len401	W1-1	401	AYV88179.2 putative DNA dependent RNA polymerase II second largest subunit [Mandarin fish ranavirus]	100	46	4.71E-21	0
k141_55127_flag1_multi3.5462_len1179	W4-1	1179	AYV88191.1 hypothetical protein [Mandarin fish ranavirus]	97.8	46	4.76E-21	12
k141_95510_flag0_multi5.1036_len498	W1-1	498	XRB52768.1 P31K protein, partial [Largemouth bass virus]	91.8	49	4.79E-21	6
k141_23010_flag1_multi1.0000_len351	W2-1	351	XRL22790.1 hypothetical protein [Siniperca chuatsi ranavirus]	100	46	4.81E-21	0
k141_26476_flag0_multi1.0000_len282	W4-1	282	AXB27397.1 major capsid protein, partial [Ranavirus sp.]	95.7	46	5.11E-21	0
k141_93117_flag1_multi5.6788_len499	W4-1	499	QJE49153.1 hypothetical protein LMBV_004 [Largemouth bass virus]	100	46	5.16E-21	12
k141_29543_flag1_multi19.8303_len2698	W5-1	2698	QJE49128.1 hypothetical protein LMBV_065 [Largemouth bass virus]	97.8	46	5.31E-21	147
k141_92989_flag0_multi1.0000_len306	W5-1	306	XRL22790.1 hypothetical protein [Siniperca chuatsi ranavirus]	100	46	5.43E-21	1
k141_56200_flag1_multi3.8825_len728	W5-1	728	UUY86238.1 hypothetical protein [Largemouth bass virus]	97.9	48	5.46E-21	12

k141_69996_flag1_multi16.3458_len702	W1-1	702	AYV88179.2 putative DNA dependent RNA polymerase II second largest subunit [Mandarin fish ranavirus]	94	50	5.60E-21	34
k141_96149_flag0_multi6.0000_len278	W5-1	278	UUY86258.1 hypothetical protein [Largemouth bass virus]	100	46	5.74E-21	0
k141_119322_flag1_multi1.0000_len387	W5-1	387	QJE49112.1 hypothetical protein LMBV_049 [Largemouth bass virus]	83	53	5.76E-21	1
k141_28559_flag1_multi1.0000_len402	W5-1	402	UUY86258.1 hypothetical protein [Largemouth bass virus]	63.9	83	5.78E-21	0
k141_32572_flag1_multi1.0000_len383	W5-1	383	AYV88120.1 putative p31K protein [Mandarin fish ranavirus]	100	46	5.91E-21	1
k141_109281_flag1_multi2.8386_len426	W5-1	426	UUY86227.1 hypothetical protein [Largemouth bass virus]	97.7	44	6.18E-21	7
k141_157482_flag1_multi173.9635_len4001	W5-1	4001	AYV88133.1 putative DNA-directed RNA polymerase II subunit [Mandarin fish ranavirus]	80.3	61	6.22E-21	466
k141_148639_flag1_multi8.6614_len1157	W5-1	1157	WEI28974.1 hypothetical protein [Largemouth bass virus]	97.9	48	6.31E-21	30
k141_147440_flag1_multi1.0000_len685	W5-1	685	QYU76034.1 putative myristylated membrane protein, partial [Koi ranavirus]	52.6	116	6.40E-21	7
k141_117852_flag1_multi3.8000_len486	W5-1	486	QJE49101.1 hypothetical protein LMBV_038 [Largemouth bass virus]	98	49	6.63E-21	6
k141_139037_flag1_multi2.7594_len889	W5-1	889	XPZ21295.1 putative RNAseIII [Mandarin fish ranavirus]	100	46	6.75E-21	7.01
k141_4361_flag1_multi1.6483_len559	W5-1	559	QYU76034.1 putative myristylated membrane protein, partial [Koi ranavirus]	100	46	6.92E-21	0
k141_4653_flag0_multi6.2380_len952	W5-1	952	WAK75107.1 hypothetical protein [Mandarin fish ranavirus]	93.6	47	6.97E-21	80
k141_43930_flag1_multi5.9103_len999	W5-1	999	WAK75073.1 hypothetical protein [Mandarin fish ranavirus]	97.9	47	7.20E-21	18
k141_56288_flag0_multi49.2251_len674	W5-1	674	UUY86263.1 hypothetical protein [Largemouth bass virus]	100	46	7.34E-21	92
k141_128876_flag1_multi1.9406_len528	W5-1	528	AYV88120.1 putative p31K protein [Mandarin fish ranavirus]	93.9	49	7.50E-21	44
k141_111755_flag1_multi12.8269_len1735	W5-1	1735	WAK75112.1 putative LITAF PIG7 possible membrane associated motif in LPS-induced tumor necrosis factor alpha factor [Mandarin fish ranavirus]	100	46	8.55E-21	131
k141_73943_flag1_multi4.7306_len334	W4-1	334	AIG51690.1 major capsid protein, partial [Koi ranavirus]	81.8	55	8.58E-21	4
k141_119057_flag0_multi1.0000_len333	W5-1	333	QJE49153.1 hypothetical protein LMBV_004 [Largemouth bass virus]	83	53	8.61E-21	2
k141_82572_flag1_multi1.0000_len590	W5-1	590	WAK75072.1 hypothetical protein [Mandarin fish ranavirus]	97.9	47	8.65E-21	4
k141_31599_flag1_multi19.6827_len412	W6-1	412	QJE49125.1 hypothetical protein LMBV_062 [Largemouth bass virus]	100	46	8.70E-21	1
k141_57745_flag1_multi3.9086_len896	W5-1	896	AYV88155.1 hypothetical protein [Mandarin fish ranavirus]	100	46	8.74E-21	8
k141_59037_flag0_multi1.0000_len523	W5-1	523	WEI28972.1 CTD-phosphotransferase [Largemouth bass virus]	100	46	9.23E-21	4
k141_99436_flag1_multi3.8357_len561	W6-2	561	QJE49153.1 hypothetical protein LMBV_004 [Largemouth bass virus]	87.3	55	9.49E-21	4
k141_41669_flag1_multi6.5197_len928	W5-1	928	AYV88191.1 hypothetical protein [Mandarin fish ranavirus]	100	45	9.58E-21	14
k141_96281_flag0_multi1.0000_len377	W1-1	377	AIG51690.1 major capsid protein, partial [Koi ranavirus]	100	46	1.03E-20	1
k141_33055_flag1_multi117.7338_len1005	W2-1	1005	AYV88191.1 hypothetical protein [Mandarin fish ranavirus]	85.2	54	1.06E-20	696
k141_90309_flag1_multi12.9055_len829	W1-1	829	WAK75079.1 hypothetical protein [Mandarin fish ranavirus]	89.8	49	1.08E-20	28
k141_53927_flag1_multi6.8400_len985	W5-1	985	WEI29006.1 putative 2-cysteine adaptor domain protein [Largemouth bass virus]	75	68	1.08E-20	22
k141_46035_flag1_multi1.0000_len376	W5-1	376	QJE49073.1 putative p31K protein [Largemouth bass virus]	94.3	53	1.11E-20	3
k141_151002_flag0_multi5.0688_len388	W5-1	388	AIG51690.1 major capsid protein, partial [Koi ranavirus]	100	47	1.19E-20	5
k141_99971_flag1_multi3.9418_len1327	W5-1	1327	QIZ30887.1 major capsid protein, partial [Largemouth bass virus]	100	46	1.26E-20	24
k141_2596_flag1_multi2.9168_len694	W5-1	694	XPZ21295.1 putative RNAseIII [Mandarin fish ranavirus]	97.9	48	1.35E-20	17
k141_156269_flag1_multi1.0000_len775	W5-1	775	QJE49125.1 hypothetical protein LMBV_062 [Largemouth bass virus]	100	46	1.39E-20	15
k141_132570_flag1_multi3.9605_len748	W5-1	748	WEI29006.1 putative 2-cysteine adaptor domain protein [Largemouth bass virus]	68.4	76	1.41E-20	5
k141_142490_flag1_multi3.5877_len745	W5-1	745	XRL22821.1 hypothetical protein [Siniperca chuatsi ranavirus]	88.7	53	1.44E-20	13
k141_30513_flag1_multi2.8547_len733	W5-1	733	WAK75072.1 hypothetical protein [Mandarin fish ranavirus]	88.7	53	1.45E-20	5

k141_58995_flag0_multi1.0000_len279	W1-1	279	XRL22790.1 hypothetical protein [Siniperca chuatsi ranavirus]	95.8	48	1.50E-20	0
k141_99155_flag1_multi1.0000_len418	W1-1	418	UUY86197.1 hypothetical protein [Largemouth bass virus]	100	47	1.51E-20	7
k141_110732_flag1_multi1.0000_len273	W5-1	273	QJE49128.1 hypothetical protein LMBV_065 [Largemouth bass virus]	97.7	43	1.54E-20	0
k141_136363_flag1_multi5.8358_len476	W5-1	476	AFD96401.1 major capsid protein, partial [Largemouth bass virus]	100	43	1.55E-20	5
k141_70833_flag0_multi1.0000_len278	W5-1	278	AIG51690.1 major capsid protein, partial [Koi ranavirus]	100	46	1.55E-20	0
k141_6194_flag1_multi3.8720_len680	W1-1	680	UUY86241.1 hypothetical protein [Largemouth bass virus]	88.9	45	1.57E-20	7
k141_62346_flag1_multi18.7124_len1518	W4-1	1518	AFD96401.1 major capsid protein, partial [Largemouth bass virus]	100	47	1.64E-20	74
k141_147424_flag1_multi3.7753_len715	W5-1	715	UUY86192.1 putative myristylated membrane protein [Largemouth bass virus]	100	46	1.64E-20	14
k141_118114_flag1_multi17.9084_len4411	W5-1	4411	QIZ30887.1 major capsid protein, partial [Largemouth bass virus]	94	50	1.65E-20	261
k141_39029_flag1_multi13.7760_len632	W6-1	632	WEI29006.1 putative 2-cysteine adaptor domain protein [Largemouth bass virus]	100	46	1.66E-20	28
k141_111999_flag1_multi11.0000_len2684	W2-3	2684	WAK75102.1 hypothetical protein [Mandarin fish ranavirus]	95.9	49	1.69E-20	109
k141_67036_flag1_multi3.8222_len529	W1-1	529	UUY86267.1 ribonucleotide reductase alpha subunit [Largemouth bass virus]	94	50	1.72E-20	3
k141_105676_flag1_multi14.5340_len832	W2-1	832	XRB52842.1 transcription factor TFIIB cyclin-like domain-containing protein, partial [Largemouth bass virus]	100	46	1.76E-20	27
k141_29002_flag1_multi3.4061_len993	W5-1	993	QJE49077.1 hypothetical protein LMBV_014 [Largemouth bass virus]	97.7	43	1.76E-20	8
k141_96093_flag1_multi1.0000_len367	W2-1	367	AIG51690.1 major capsid protein, partial [Koi ranavirus]	100	46	1.78E-20	3.55
k141_32100_flag1_multi1.9076_len390	W6-1	390	QJE49080.1 hypothetical protein LMBV_017 [Largemouth bass virus]	97.8	46	1.80E-20	0
k141_68596_flag1_multi3.8969_len810	W1-1	810	AYV88159.1 hypothetical protein [Mandarin fish ranavirus]	100	46	1.82E-20	12
k141_52126_flag1_multi3.6632_len622	W5-1	622	WEI28972.1 CTD-phosphotransferase [Largemouth bass virus]	97.9	47	1.83E-20	5
k141_66197_flag0_multi2.8079_len370	W5-1	370	AIG51690.1 major capsid protein, partial [Koi ranavirus]	85	60	1.85E-20	2
k141_51217_flag1_multi3.9590_len751	W5-1	751	WXI69525.1 caspase recruitment domain protein [Largemouth bass virus]	100	46	1.90E-20	10
k141_155458_flag1_multi1.8610_len472	W5-1	472	UUY86227.1 hypothetical protein [Largemouth bass virus]	95.7	46	2.02E-20	0
k141_57874_flag1_multi9.4887_len896	W2-1	896	WEI28972.1 CTD-phosphotransferase [Largemouth bass virus]	97.9	47	2.05E-20	31
k141_132215_flag0_multi14.0512_len395	W1-1	395	WEI28972.1 CTD-phosphotransferase [Largemouth bass virus]	100	46	2.07E-20	1
k141_114623_flag1_multi4.8533_len741	W5-1	741	QJE49104.1 hypothetical protein LMBV_041 [Largemouth bass virus]	100	46	2.17E-20	9
k141_72770_flag0_multi1.7993_len684	W5-1	684	ALR73092.1 major capsid protein, partial [Box turtle ranavirus]	93.8	48	2.19E-20	9
k141_72610_flag1_multi18.0000_len784	W5-1	784	WAK75102.1 hypothetical protein [Mandarin fish ranavirus]	90.2	51	2.22E-20	99
k141_19950_flag1_multi4.9588_len1014	W5-1	1014	UUY86199.1 hypothetical protein [Largemouth bass virus]	83	53	2.35E-20	16
k141_168185_flag1_multi5.5361_len764	W5-1	764	AYV88120.1 putative p31K protein [Mandarin fish ranavirus]	97.8	46	2.37E-20	18
k141_111838_flag1_multi1.8891_len745	W1-1	745	QJE49065.1 hypothetical protein LMBV_002 [Largemouth bass virus]	100	43	2.50E-20	6
k141_46050_flag1_multi17.0907_len1685	W1-1	1685	QIZ30887.1 major capsid protein, partial [Largemouth bass virus]	100	46	2.55E-20	115
k141_66267_flag1_multi4.8850_len1080	W5-1	1080	WXI69541.1 hypothetical protein [Largemouth bass virus]	96	50	2.56E-20	26
k141_19826_flag1_multi16.3984_len1233	W6-1	1233	AIZ70320.1 major capsid protein, partial [Chinese giant salamander virus]	78.3	60	2.57E-20	33
k141_64511_flag0_multi2.7427_len518	W4-1	518	WXI69548.1 hypothetical protein [Largemouth bass virus]	100	45	2.59E-20	8
k141_61248_flag1_multi2.5803_len851	W5-1	851	AYV88134.2 putative tyrosine kinase [Mandarin fish ranavirus]	95.9	49	2.64E-20	27
k141_127332_flag1_multi3.0000_len661	W3-2	661	AYV88172.2 hypothetical protein [Mandarin fish ranavirus]	91.5	164	2.69E-20	4
k141_104333_flag0_multi1.8889_len546	W1-1	546	AYV88159.1 hypothetical protein [Mandarin fish ranavirus]	100	46	2.83E-20	11
k141_47138_flag0_multi1.0000_len332	W4-1	332	AYV88191.1 hypothetical protein [Mandarin fish ranavirus]	83.7	49	2.85E-20	0
k141_47459_flag1_multi2.8817_len530	W5-1	530	QJE49137.1 hypothetical protein LMBV_074 [Largemouth bass virus]	92.2	51	2.86E-20	5
k141_41892_flag0_multi1.0000_len329	W5-1	329	AIG51690.1 major capsid protein, partial [Koi ranavirus]	93.6	47	2.97E-20	0

k141_16284_flag1_multi1.5706_len665	W5-1	665	UUY86235.1 hypothetical protein [Largemouth bass virus]	100	46	3.05E-20	4
k141_53993_flag1_multi1.0000_len376	W5-1	376	AYV88134.2 putative tyrosine kinase [Mandarin fish ranavirus]	100	46	3.05E-20	1
k141_85277_flag1_multi3.6469_len444	W1-1	444	AIG51690.1 major capsid protein, partial [Koi ranavirus]	83.6	61	3.07E-20	1
k141_163861_flag0_multi1.0886_len299	W5-1	299	QJE49215.1 putative orf58-like protein [Largemouth bass virus]	100	46	3.08E-20	0
k141_38384_flag1_multi4.5783_len767	W5-1	767	WEI29006.1 putative 2-cysteine adaptor domain protein [Largemouth bass virus]	100	47	3.12E-20	6
k141_6224_flag1_multi4.5440_len1106	W4-1	1106	WHA35503.1 putative myristylated membrane protein [Micropterus salmoides ranavirus]	100	44	3.26E-20	24
k141_27324_flag1_multi2.8847_len540	W5-1	540	QJE49137.1 hypothetical protein LMBV_074 [Largemouth bass virus]	94	50	3.26E-20	4
k141_145378_flag1_multi6.0000_len669	W5-1	669	AYV88167.1 hypothetical protein [Mandarin fish ranavirus]	92.9	42	3.34E-20	11
k141_153164_flag0_multi28.6273_len1869	W5-1	1869	WAK75073.1 hypothetical protein [Mandarin fish ranavirus]	100	46	3.48E-20	119
k141_21227_flag0_multi1.0000_len256	W4-1	256	QJE49077.1 hypothetical protein LMBV_014 [Largemouth bass virus]	95	40	3.49E-20	0
k141_146856_flag1_multi1.8767_len514	W5-1	514	AYV88120.1 putative p31K protein [Mandarin fish ranavirus]	95.7	46	3.58E-20	1
k141_28109_flag1_multi3.8990_len824	W5-1	824	AYV88191.1 hypothetical protein [Mandarin fish ranavirus]	97.9	47	3.59E-20	8
k141_97011_flag1_multi1.6868_len406	W1-1	406	AIG51690.1 major capsid protein, partial [Koi ranavirus]	90.6	53	3.79E-20	1
k141_146799_flag1_multi3.6465_len1049	W2-3	1049	AYV88136.2 hypothetical protein [Mandarin fish ranavirus]	97.7	43	3.92E-20	14
k141_112715_flag1_multi18.7039_len749	W1-1	749	QYU76034.1 putative myristylated membrane protein, partial [Koi ranavirus]	100	46	3.96E-20	9
k141_84634_flag0_multi171.7544_len1912	W4-1	1912	UUY86235.1 hypothetical protein [Largemouth bass virus]	95.9	49	3.98E-20	1679
k141_85586_flag1_multi11.9167_len561	W4-1	561	AIG51690.1 major capsid protein, partial [Koi ranavirus]	92.2	51	3.99E-20	15
k141_68589_flag1_multi1.9696_len503	W5-1	503	UUY86199.1 hypothetical protein [Largemouth bass virus]	100	41	4.05E-20	3
k141_22074_flag1_multi2.8509_len530	W4-1	530	AIG51690.1 major capsid protein, partial [Koi ranavirus]	94.2	52	4.08E-20	4
k141_145656_flag1_multi1.0000_len423	W5-1	423	AYV88191.1 hypothetical protein [Mandarin fish ranavirus]	100	44	4.08E-20	0
k141_72834_flag0_multi19.2727_len471	W5-1	471	AYV88142.2 hypothetical protein [Mandarin fish ranavirus]	100	47	4.20E-20	0
k141_3212_flag0_multi5.0947_len648	W5-1	648	QYU76034.1 putative myristylated membrane protein, partial [Koi ranavirus]	89.6	48	4.33E-20	4
k141_77542_flag1_multi1.0000_len568	W1-1	568	WEI28968.1 putative DNA dependent RNA polymerase a subunit [Largemouth bass virus]	97.8	45	4.38E-20	8
k141_6840_flag1_multi1.0000_len524	W5-1	524	UUY86235.1 hypothetical protein [Largemouth bass virus]	100	46	4.38E-20	2
k141_26388_flag0_multi4.9425_len1672	W3-2	1672	QJE49112.1 hypothetical protein LMBV_049 [Largemouth bass virus]	100	46	4.72E-20	26
k141_14187_flag0_multi1.0000_len278	W4-1	278	AYV88120.1 putative p31K protein [Mandarin fish ranavirus]	100	46	4.82E-20	0
k141_8124_flag0_multi3.6359_len1278	W5-1	1278	AYV88191.1 hypothetical protein [Mandarin fish ranavirus]	100	45	4.84E-20	29
k141_32091_flag1_multi10.4548_len561	W4-1	561	XRB52768.1 P31K protein, partial [Largemouth bass virus]	97.8	46	4.92E-20	15
k141_149795_flag0_multi1.0000_len322	W5-1	322	AIG51690.1 major capsid protein, partial [Koi ranavirus]	98	50	5.31E-20	3.62
k141_61217_flag1_multi1.0000_len280	W5-1	280	QJE49066.1 hypothetical protein LMBV_003 [Largemouth bass virus]	100	46	5.58E-20	3
k141_61217_flag1_multi1.0000_len280	W5-1	280	QJE49066.1 hypothetical protein LMBV_003 [Largemouth bass virus]	100	46	5.58E-20	0
k141_77977_flag0_multi1.0000_len281	W5-1	281	UVF58793.1 MAG: DNA polymerase [Halichoeres melanurus ranavirus]	100	46	5.75E-20	0
k141_88604_flag1_multi2.9624_len460	W5-1	460	UUY86241.1 hypothetical protein [Largemouth bass virus]	100	38	5.77E-20	7
k141_12550_flag1_multi1.0000_len537	W3-1	537	QJE49144.1 hypothetical protein LMBV_081 [Largemouth bass virus]	100	46	6.05E-20	2
k141_4045_flag1_multi4.8870_len495	W4-1	495	AYV88191.1 hypothetical protein [Mandarin fish ranavirus]	95.2	42	6.27E-20	5
k141_22321_flag0_multi84.6432_len1049	W1-1	1049	AYV88125.2 hypothetical protein [Mandarin fish ranavirus]	93.5	62	6.33E-20	283
k141_101051_flag1_multi1.0000_len331	W4-1	331	WEI28972.1 CTD-phosphotransferase [Largemouth bass virus]	100	47	6.46E-20	0
k141_153239_flag1_multi7.2500_len477	W5-1	477	UUY86241.1 hypothetical protein [Largemouth bass virus]	97.4	38	6.77E-20	11

k141_109571_flag1_multi3.0000_len342	W3-2	342	UUY86238.1 hypothetical protein [Largemouth bass virus]	97.7	43	7.08E-20	2
k141_114721_flag1_multi7.8000_len946	W5-1	946	WEI28972.1 CTD-phosphotransferase [Largemouth bass virus]	100	46	7.21E-20	21
k141_95128_flag1_multi4.6421_len711	W1-1	711	QJE49153.1 hypothetical protein LMBV_004 [Largemouth bass virus]	73.4	64	7.22E-20	10
k141_72402_flag1_multi1.6253_len560	W4-1	560	AYV88179.2 putative DNA dependent RNA polymerase II second largest subunit [Mandarin fish ranavirus]	93.8	48	7.25E-20	3
k141_121872_flag1_multi4.0775_len683	W1-1	683	XRB52842.1 transcription factor TFIIB cyclin-like domain-containing protein, partial [Largemouth bass virus]	100	41	7.70E-20	9
k141_72571_flag1_multi3.8874_len754	W4-1	754	WAK75107.1 hypothetical protein [Mandarin fish ranavirus]	100	41	7.86E-20	8
k141_82548_flag1_multi1.0000_len361	W5-1	361	UUY86235.1 hypothetical protein [Largemouth bass virus]	100	46	8.02E-20	11
k141_46066_flag1_multi2.9345_len843	W5-1	843	AYV88191.1 hypothetical protein [Mandarin fish ranavirus]	97.8	46	8.15E-20	5
k141_87014_flag1_multi2.1534_len519	W2-3	519	WEI28972.1 CTD-phosphotransferase [Largemouth bass virus]	92.2	51	8.19E-20	4
k141_97628_flag1_multi2.9017_len609	W5-1	609	WEI29006.1 putative 2-cysteine adaptor domain protein [Largemouth bass virus]	100	46	8.30E-20	8
k141_11997_flag0_multi3.6662_len797	W5-1	797	WEI28972.1 CTD-phosphotransferase [Largemouth bass virus]	100	47	8.60E-20	5
k141_90772_flag1_multi2.9218_len729	W5-1	729	QYU76034.1 putative myristylated membrane protein, partial [Koi ranavirus]	86	57	8.62E-20	6
k141_57634_flag1_multi4.8938_len970	W5-1	970	WHU98721.1 MCP, partial [Hybrid snakehead ranavirus]	92	50	8.88E-20	860
k141_99174_flag1_multi3.8028_len430	W5-1	430	AYV88159.1 hypothetical protein [Mandarin fish ranavirus]	100	44	8.96E-20	2
k141_125873_flag1_multi1.0000_len269	W1-1	269	WAK75103.1 hypothetical protein [Mandarin fish ranavirus]	100	43	8.98E-20	0
k141_166669_flag1_multi17.8368_len2059	W5-1	2059	UUY86192.1 putative myristylated membrane protein [Largemouth bass virus]	100	47	9.30E-20	123
k141_133567_flag1_multi9.6956_len821	W5-1	821	QYU76034.1 putative myristylated membrane protein, partial [Koi ranavirus]	100	46	9.61E-20	24
k141_94028_flag0_multi1.0000_len376	W5-1	376	WAK75136.1 hypothetical protein [Mandarin fish ranavirus]	100	45	9.66E-20	5
k141_43808_flag0_multi2.8537_len729	W4-1	729	AIG51690.1 major capsid protein, partial [Koi ranavirus]	97.9	48	9.73E-20	2
k141_23456_flag1_multi10.1534_len956	W4-1	956	UUY86192.1 putative myristylated membrane protein [Largemouth bass virus]	100	46	9.86E-20	34
k141_98852_flag1_multi11.6598_len2031	W5-1	2031	QJE49068.1 hypothetical protein LMBV_005 [Largemouth bass virus]	97.9	47	9.88E-20	114.79
k141_69565_flag0_multi7.9790_len1473	W5-1	1473	AFD96401.1 major capsid protein, partial [Largemouth bass virus]	84.2	57	9.98E-20	52
k141_26840_flag0_multi2.8693_len493	W1-1	493	AIG51690.1 major capsid protein, partial [Koi ranavirus]	100	46	1.00E-19	6
k141_34169_flag1_multi15.7945_len1820	W1-1	1820	QJE49112.1 hypothetical protein LMBV_049 [Largemouth bass virus]	100	44	1.04E-19	78
k141_57070_flag1_multi1.9395_len521	W1-1	521	AYV88211.1 hypothetical protein [Mandarin fish ranavirus]	94	50	1.06E-19	1
k141_76851_flag1_multi4.2332_len917	W5-1	917	XRB52797.1 Bcl-2 Bcl-2 homology region 1-3 domain-containing protein [Largemouth bass virus]	97.9	47	1.12E-19	13
k141_20970_flag1_multi1.8833_len501	W1-1	501	UUY86192.1 putative myristylated membrane protein [Largemouth bass virus]	65.8	73	1.14E-19	7
k141_109331_flag0_multi5.3570_len844	W1-3	844	XRL22821.1 hypothetical protein [Siniperca chuatsi ranavirus]	100	46	1.14E-19	27
k141_102609_flag1_multi2.9006_len604	W4-1	604	AIG51690.1 major capsid protein, partial [Koi ranavirus]	100	46	1.15E-19	6
k141_122495_flag1_multi1.0000_len280	W5-1	280	UUY86192.1 putative myristylated membrane protein [Largemouth bass virus]	100	42	1.15E-19	0
k141_147265_flag1_multi3.8565_len580	W5-1	580	QJE49104.1 hypothetical protein LMBV_041 [Largemouth bass virus]	94	50	1.16E-19	5
k141_164061_flag1_multi1.0000_len362	W5-1	362	AIG51690.1 major capsid protein, partial [Koi ranavirus]	100	45	1.19E-19	6
k141_94398_flag1_multi2.0000_len433	W5-1	433	UUY86241.1 hypothetical protein [Largemouth bass virus]	92.7	41	1.20E-19	3
k141_141168_flag1_multi18.8806_len3600	W5-1	3600	WEI28974.1 hypothetical protein [Largemouth bass virus]	100	46	1.21E-19	244
k141_106369_flag0_multi83.0000_len659	W5-3	659	XRB52842.1 transcription factor TFIIB cyclin-like domain-containing protein, partial [Largemouth bass virus]	76.8	56	1.22E-19	213
k141_6278_flag1_multi6.6231_len937	W5-1	937	QJE49077.1 hypothetical protein LMBV_014 [Largemouth bass virus]	91.3	46	1.26E-19	32

k141_26540_flag1_multi2.9548_len539	W5-1	539	AYV88120.1 putative p31K protein [Mandarin fish ranavirus]	97.9	47	1.26E-19	5
k141_60266_flag0_multi1.0000_len313	W1-1	313	AIG51690.1 major capsid protein, partial [Koi ranavirus]	100	46	1.27E-19	90.65
k141_105001_flag0_multi1.8284_len1586	W5-1	1586	WAK75136.1 hypothetical protein [Mandarin fish ranavirus]	71.4	70	1.29E-19	40
k141_99433_flag1_multi1.0000_len278	W4-1	278	AYV88211.1 hypothetical protein [Mandarin fish ranavirus]	93.9	49	1.35E-19	0
k141_95371_flag0_multi2.8845_len470	W1-1	470	AYV88159.1 hypothetical protein [Mandarin fish ranavirus]	100	44	1.37E-19	0
k141_69694_flag1_multi1.0000_len524	W5-1	524	UUY86258.1 hypothetical protein [Largemouth bass virus]	100	47	1.44E-19	22
k141_25061_flag1_multi3.8964_len807	W5-1	807	XRB52797.1 Bcl-2 Bcl-2 homology region 1-3 domain-containing protein [Largemouth bass virus]	100	46	1.50E-19	8
k141_141979_flag1_multi4.4437_len434	W5-1	434	QJE49104.1 hypothetical protein LMBV_041 [Largemouth bass virus]	100	44	1.51E-19	26.63
k141_74965_flag1_multi22.7678_len632	W4-1	632	QJE49137.1 hypothetical protein LMBV_074 [Largemouth bass virus]	100	46	1.52E-19	65
k141_88153_flag1_multi5.7709_len643	W4-1	643	XRB52768.1 P31K protein, partial [Largemouth bass virus]	88.2	51	1.52E-19	8
k141_60637_flag0_multi1.8593_len603	W5-1	603	AYV88120.1 putative p31K protein [Mandarin fish ranavirus]	97.8	46	1.65E-19	3
k141_31688_flag1_multi10.2433_len1004	W4-1	1004	AYV88120.1 putative p31K protein [Mandarin fish ranavirus]	100	46	1.66E-19	34
k141_89573_flag1_multi1.0000_len338	W5-1	338	AIG51690.1 major capsid protein, partial [Koi ranavirus]	75.4	65	1.71E-19	0
k141_49187_flag1_multi12.7811_len1402	W1-1	1402	AYV88191.1 hypothetical protein [Mandarin fish ranavirus]	97.8	46	1.79E-19	50
k141_53648_flag1_multi2.4714_len665	W1-1	665	QJE49215.1 putative orf58-like protein [Largemouth bass virus]	92	50	1.80E-19	5
k141_24489_flag1_multi12.4671_len567	W4-1	567	WEI28972.1 CTD-phosphotransferase [Largemouth bass virus]	97.9	47	1.84E-19	29
k141_62140_flag1_multi1.0000_len291	W4-1	291	AIG51690.1 major capsid protein, partial [Koi ranavirus]	100	46	1.87E-19	61
k141_39213_flag1_multi1.0000_len706	W5-1	706	UUY86221.1 hypothetical protein [Largemouth bass virus]	100	46	1.87E-19	14
k141_149313_flag1_multi2.5503_len817	W5-1	817	AYV88120.1 putative p31K protein [Mandarin fish ranavirus]	97.8	46	1.87E-19	5
k141_58220_flag1_multi2.8277_len408	W5-1	408	QJE49066.1 hypothetical protein LMBV_003 [Largemouth bass virus]	84.5	58	1.89E-19	2
k141_34050_flag1_multi3.3835_len1356	W5-1	1356	QYU76034.1 putative myristylated membrane protein, partial [Koi ranavirus]	94.1	51	1.89E-19	11
k141_37590_flag1_multi1.9347_len493	W5-1	493	AIG51690.1 major capsid protein, partial [Koi ranavirus]	97.9	47	1.98E-19	2
k141_79157_flag1_multi9.8745_len1790	W1-1	1790	AIZ70320.1 major capsid protein, partial [Chinese giant salamander virus]	88.5	52	2.01E-19	68
k141_91430_flag1_multi4.4578_len1172	W5-1	1172	WEI29002.1 hypothetical protein [Largemouth bass virus]	100	45	2.07E-19	11
k141_28965_flag1_multi4.4006_len463	W4-1	463	AYV88134.2 putative tyrosine kinase [Mandarin fish ranavirus]	100	44	2.10E-19	3
k141_54160_flag0_multi3.2873_len503	W1-1	503	AIG51690.1 major capsid protein, partial [Koi ranavirus]	92.2	51	2.18E-19	98.35
k141_165279_flag1_multi1.0000_len545	W5-1	545	QJE49065.1 hypothetical protein LMBV_002 [Largemouth bass virus]	100	42	2.19E-19	6
k141_101867_flag1_multi1.0000_len922	W5-1	922	WAK75114.1 hypothetical protein [Mandarin fish ranavirus]	95.6	45	2.19E-19	31
k141_82389_flag1_multi3.8597_len1445	W5-1	1445	QIZ30887.1 major capsid protein, partial [Largemouth bass virus]	64.6	82	2.20E-19	25
k141_93369_flag1_multi4.7500_len349	W4-1	349	AYV88120.1 putative p31K protein [Mandarin fish ranavirus]	97.7	43	2.25E-19	5
k141_150118_flag1_multi4.6893_len553	W5-1	553	UUY86194.1 hypothetical protein [Largemouth bass virus]	97.9	47	2.53E-19	7
k141_63554_flag1_multi6.3196_len773	W5-1	773	XPZ21261.1 hypothetical protein MRVORF002 [Mandarin fish ranavirus]	97.8	46	2.63E-19	13
k141_153331_flag0_multi9.8537_len2909	W5-1	2909	QJE49109.1 hypothetical protein LMBV_046 [Largemouth bass virus]	100	46	2.66E-19	86
k141_14507_flag1_multi1.0000_len277	W2-1	277	QJE49073.1 putative p31K protein [Largemouth bass virus]	95.9	49	2.67E-19	4
k141_159630_flag1_multi2.9374_len876	W5-1	876	AYV88179.2 putative DNA dependent RNA polymerase II second largest subunit [Mandarin fish ranavirus]	100	46	2.73E-19	5
k141_77097_flag0_multi1.0000_len270	W4-1	270	AIG51690.1 major capsid protein, partial [Koi ranavirus]	100	43	2.75E-19	2
k141_34373_flag1_multi1.9404_len527	W5-1	527	AIG51690.1 major capsid protein, partial [Koi ranavirus]	100	47	2.80E-19	1
k141_2511_flag1_multi3.8240_len533	W5-1	533	XRL22790.1 hypothetical protein [Siniperca chuatsi ranavirus]	100	46	2.80E-19	5

k141_21512_flag1_multi5.2164_len1033	W4-1	1033	QJE49090.1 hypothetical protein LMBV_027 [Largemouth bass virus]	100	46	2.88E-19	15
k141_13430_flag1_multi3.8282_len892	W6-1	892	QJE49114.1 hypothetical protein LMBV_051 [Largemouth bass virus]	80.4	51	2.89E-19	27
k141_89907_flag1_multi1.7493_len907	W5-1	907	XRL22790.1 hypothetical protein [Siniperca chuatsi ranavirus]	83.9	56	2.96E-19	10
k141_25877_flag1_multi1.0000_len293	W6-1	293	AYV88118.1 hypothetical protein [Mandarin fish ranavirus]	100	37	3.01E-19	0
k141_52117_flag1_multi3.8288_len544	W4-1	544	XRL22790.1 hypothetical protein [Siniperca chuatsi ranavirus]	100	46	3.06E-19	9
k141_2085_flag1_multi5.2237_len1035	W5-1	1035	AIG51690.1 major capsid protein, partial [Koi ranavirus]	100	46	3.08E-19	20
k141_109322_flag1_multi1.0000_len415	W5-1	415	AIG51690.1 major capsid protein, partial [Koi ranavirus]	97.9	48	3.10E-19	5
k141_13981_flag0_multi11.7067_len6141	W1-1	6141	XRL22821.1 hypothetical protein [Siniperca chuatsi ranavirus]	90.4	52	3.14E-19	186
k141_98498_flag1_multi1.0000_len573	W5-1	573	AIG51690.1 major capsid protein, partial [Koi ranavirus]	100	45	3.17E-19	8
k141_103397_flag1_multi5.8766_len1073	W1-1	1073	WAK75102.1 hypothetical protein [Mandarin fish ranavirus]	93.8	48	3.31E-19	22
k141_71435_flag1_multi15.8900_len3277	W5-1	3277	AYV88191.1 hypothetical protein [Mandarin fish ranavirus]	90.4	52	3.41E-19	202
k141_43786_flag1_multi1.0000_len280	W1-1	280	UUY86197.1 hypothetical protein [Largemouth bass virus]	94	50	3.56E-19	0
k141_120635_flag0_multi1.0000_len864	W1-1	864	AYV88120.1 putative p31K protein [Mandarin fish ranavirus]	97.8	46	3.57E-19	23.88
k141_35420_flag1_multi5.7876_len447	W4-1	447	AYV88120.1 putative p31K protein [Mandarin fish ranavirus]	97.7	43	3.57E-19	11
k141_155697_flag1_multi8.3645_len1864	W5-1	1864	QJE49125.1 hypothetical protein LMBV_062 [Largemouth bass virus]	97.9	48	3.68E-19	45
k141_92482_flag1_multi2.8340_len659	W5-1	659	AIG51690.1 major capsid protein, partial [Koi ranavirus]	100	46	3.74E-19	4
k141_96019_flag1_multi2.5806_len780	W5-1	780	QJE49066.1 hypothetical protein LMBV_003 [Largemouth bass virus]	88.7	53	3.76E-19	9
k141_58425_flag1_multi2.8540_len456	W5-1	456	AAF64582.1 capsid protein, partial [Guppyfish iridovirus]	95.7	46	3.78E-19	0
k141_16766_flag1_multi11.6241_len814	W1-1	814	QJE49149.1 putative immediate early protein ICP-46 [Largemouth bass virus]	100	46	3.91E-19	32
k141_132021_flag1_multi2.8919_len363	W1-1	363	AYV88120.1 putative p31K protein [Mandarin fish ranavirus]	97.7	43	3.91E-19	4
k141_158552_flag1_multi15.5898_len982	W5-1	982	WEI29006.1 putative 2-cysteine adaptor domain protein [Largemouth bass virus]	100	47	3.95E-19	43
k141_29073_flag1_multi2.9394_len405	W5-1	405	XRB52780.1 hypothetical protein LMBV_23 [Largemouth bass virus]	100	41	3.97E-19	6
k141_147941_flag1_multi2.8241_len357	W5-1	357	AIG51690.1 major capsid protein, partial [Koi ranavirus]	100	45	4.10E-19	3
k141_127257_flag1_multi1.0000_len279	W1-1	279	AIG51690.1 major capsid protein, partial [Koi ranavirus]	92	50	4.13E-19	0
k141_76451_flag1_multi2.5671_len931	W4-1	931	WEI28972.1 CTD-phosphotransferase [Largemouth bass virus]	100	46	4.23E-19	19
k141_34581_flag1_multi1.0000_len469	W4-1	469	QJE49104.1 hypothetical protein LMBV_041 [Largemouth bass virus]	100	45	4.30E-19	96
k141_92918_flag1_multi2.8198_len474	W5-1	474	UUY86221.1 hypothetical protein [Largemouth bass virus]	100	46	4.56E-19	4
k141_16493_flag1_multi4.0345_len721	W5-1	721	WXI69525.1 caspase recruitment domain protein [Largemouth bass virus]	91.8	49	4.57E-19	19
k141_70828_flag1_multi3.9006_len835	W4-1	835	XRL22821.1 hypothetical protein [Siniperca chuatsi ranavirus]	100	46	4.72E-19	12
k141_72897_flag0_multi1.0000_len516	W5-1	516	AIG51690.1 major capsid protein, partial [Koi ranavirus]	100	44	4.89E-19	2
k141_103229_flag1_multi1.9170_len418	W5-1	418	AAF64580.1 capsid protein, partial [Frog virus 3]	95.7	46	5.09E-19	3
k141_59837_flag1_multi1.0000_len742	W5-1	742	QJE49103.1 hypothetical protein LMBV_040 [Largemouth bass virus]	100	46	5.20E-19	11
k141_138996_flag1_multi1.5255_len455	W5-1	455	WAK75112.1 putative LITAF PIG7 possible membrane associated motif in LPS-induced tumor necrosis factor alpha factor [Mandarin fish ranavirus]	100	39	5.37E-19	6
k141_134716_flag1_multi1.0000_len294	W5-1	294	XRL22790.1 hypothetical protein [Siniperca chuatsi ranavirus]	79.4	63	5.46E-19	0
k141_143795_flag1_multi1.0000_len432	W5-1	432	XRB52831.1 hypothetical protein LMBV_74 [Largemouth bass virus]	97.8	46	5.84E-19	1
k141_124765_flag0_multi2.9173_len673	W5-1	673	AIG51690.1 major capsid protein, partial [Koi ranavirus]	100	46	5.89E-19	11
k141_132619_flag0_multi2.6436_len719	W5-1	719	QJE49153.1 hypothetical protein LMBV_004 [Largemouth bass virus]	97.8	46	5.93E-19	166.38
k141_170_flag1_multi1.0000_len648	W5-1	648	UUY86269.1 hypothetical protein [Largemouth bass virus]	92	50	5.99E-19	0
k141_25141_flag0_multi1.0000_len435	W5-1	435	WAK75139.1 putative myristylated membrane protein [Mandarin fish ranavirus]	100	76	5.99E-19	486.05

k141_97362_flag1_multi3.5245_len488	W2-1	488	UUY86241.1 hypothetical protein [Largemouth bass virus]	92.5	40	6.01E-19	5
k141_9809_flag1_multi3.1190_len620	W5-1	620	UUY86261.1 hypothetical protein [Largemouth bass virus]	97.9	48	6.05E-19	5
k141_133587_flag0_multi3.7122_len610	W5-2	610	UUY86221.1 hypothetical protein [Largemouth bass virus]	100	46	6.30E-19	6
k141_9346_flag1_multi1.9207_len431	W1-1	431	UVF58785.1 MAG: major capsid protein [Halichoeres melanurus ranavirus]	95.9	49	6.69E-19	9
k141_164548_flag1_multi3.7335_len670	W5-1	670	UUY86235.1 hypothetical protein [Largemouth bass virus]	100	46	6.69E-19	12
k141_33639_flag1_multi4.2129_len822	W1-1	822	WEI28972.1 CTD-phosphotransferase [Largemouth bass virus]	100	45	6.82E-19	13
k141_92034_flag1_multi7.6798_len1081	W4-1	1081	AYV88159.1 hypothetical protein [Mandarin fish ranavirus]	100	46	6.83E-19	45
k141_53237_flag1_multi4.9085_len1147	W1-1	1147	QYU76034.1 putative myristylated membrane protein, partial [Koi ranavirus]	100	46	6.90E-19	20
k141_121144_flag1_multi7.3063_len820	W5-1	820	AAC79508.1 viral core protein, partial [Ictalurus melas ranavirus]	85.1	47	6.95E-19	20
k141_179467_flag1_multi1.0000_len369	W2-3	369	UUY86256.1 putative orf58-like protein [Largemouth bass virus]	100	46	7.05E-19	0
k141_114528_flag1_multi2.8545_len622	W5-1	622	UUY86258.1 hypothetical protein [Largemouth bass virus]	97.9	47	7.21E-19	4
k141_142218_flag1_multi1.0000_len306	W5-1	306	QJE49096.1 putative D5 family NTPase/ATPase [Largemouth bass virus]	73.1	67	7.24E-19	1
k141_24931_flag1_multi1.0000_len634	W1-1	634	WAK75112.1 putative LITAF PIG7 possible membrane associated motif in LPS-induced tumor necrosis factor alpha factor [Mandarin fish ranavirus]	100	41	7.32E-19	10
k141_116507_flag0_multi6.4962_len403	W1-1	403	XRB52842.1 transcription factor TFIIB cyclin-like domain-containing protein, partial [Largemouth bass virus]	49.1	108	7.49E-19	6
k141_86011_flag1_multi5.4914_len662	W1-1	662	AYV88120.1 putative p31K protein [Mandarin fish ranavirus]	90	50	7.73E-19	10
k141_72844_flag1_multi12.0000_len1555	W5-1	1555	AIG51690.1 major capsid protein, partial [Koi ranavirus]	100	49	8.02E-19	54
k141_117925_flag1_multi6.9296_len1376	W5-1	1376	QJE49137.1 hypothetical protein LMBV_074 [Largemouth bass virus]	90.4	52	8.19E-19	32
k141_3124_flag1_multi8.8630_len1484	W1-1	1484	XRL22821.1 hypothetical protein [Siniperca chuatsi ranavirus]	67.9	81	8.25E-19	52
k141_83680_flag1_multi3.9585_len1298	W2-1	1298	UVF58787.1 MAG: DNA-dependent RNA polymerase largest subunit, partial [Halichoeres melanurus ranavirus]	93.6	47	8.46E-19	24
k141_104085_flag1_multi1.0000_len724	W1-1	724	AFD96401.1 major capsid protein, partial [Largemouth bass virus]	88.2	51	8.50E-19	26
k141_96314_flag1_multi6.7441_len563	W4-1	563	AYV88120.1 putative p31K protein [Mandarin fish ranavirus]	97.7	44	8.52E-19	10
k141_50895_flag1_multi5.4087_len1636	W1-1	1636	AFD96401.1 major capsid protein, partial [Largemouth bass virus]	97.8	46	8.56E-19	30
k141_4855_flag1_multi1.0000_len433	W1-1	433	QJE49153.1 hypothetical protein LMBV_004 [Largemouth bass virus]	95.5	44	8.61E-19	1
k141_149589_flag1_multi2.7455_len471	W5-1	471	UUY86261.1 hypothetical protein [Largemouth bass virus]	81.4	59	8.94E-19	4
k141_75314_flag1_multi1.8549_len458	W1-1	458	UVF58785.1 MAG: major capsid protein [Halichoeres melanurus ranavirus]	100	47	9.05E-19	0
k141_81887_flag1_multi1.0000_len435	W5-1	435	AYV88211.1 hypothetical protein [Mandarin fish ranavirus]	100	45	9.11E-19	1
k141_25007_flag0_multi14.6680_len647	W4-1	647	AYV88120.1 putative p31K protein [Mandarin fish ranavirus]	95.7	47	9.47E-19	19
k141_48992_flag1_multi52.1545_len510	W4-1	510	QJE49149.1 putative immediate early protein ICP-46 [Largemouth bass virus]	75.9	58	9.49E-19	87
k141_137480_flag1_multi3.7738_len446	W4-3	446	QJE49215.1 putative orf58-like protein [Largemouth bass virus]	100	46	9.70E-19	4
k141_90326_flag1_multi3.3163_len767	W5-1	767	UUY86211.1 hypothetical protein [Largemouth bass virus]	100	46	9.79E-19	6
k141_62551_flag1_multi4.8009_len784	W5-1	784	AIG51690.1 major capsid protein, partial [Koi ranavirus]	76.9	65	1.03E-18	14
k141_69653_flag1_multi4.5915_len682	W5-1	682	WEI29006.1 putative 2-cysteine adaptor domain protein [Largemouth bass virus]	97.8	46	1.04E-18	11
k141_74140_flag1_multi6.2659_len991	W6-1	991	QYU76034.1 putative myristylated membrane protein, partial [Koi ranavirus]	100	46	1.04E-18	19
k141_39031_flag0_multi1.7386_len229	W5-1	229	QJE49105.1 hypothetical protein LMBV_042 [Largemouth bass virus]	97.9	47	1.05E-18	0
k141_45773_flag1_multi1.0000_len426	W5-1	426	QJE49095.1 hypothetical protein LMBV_032 [Largemouth bass virus]	97.7	43	1.06E-18	2
k141_133428_flag1_multi1.9567_len672	W5-1	672	AIG51690.1 major capsid protein, partial [Koi ranavirus]	94.1	51	1.09E-18	2
k141_58964_flag1_multi18.2499_len2810	W5-1	2810	XRL22821.1 hypothetical protein [Siniperca chuatsi ranavirus]	90.6	53	1.10E-18	160

k141_66218_flag1_multi1.0000_len346	W4-1	346	AYV88211.1 hypothetical protein [Mandarin fish ranavirus]	97.8	46	1.13E-18	0
k141_27221_flag1_multi1.0000_len316	W5-1	316	QJE49066.1 hypothetical protein LMBV_003 [Largemouth bass virus]	86.3	51	1.14E-18	0
k141_68868_flag1_multi13.6729_len1055	W5-1	1055	QJE49104.1 hypothetical protein LMBV_041 [Largemouth bass virus]	100	46	1.14E-18	35
k141_144065_flag1_multi23.9241_len7114	W5-1	7114	UUY86235.1 hypothetical protein [Largemouth bass virus]	97.9	47	1.16E-18	585
k141_17202_flag1_multi2.5227_len405	W2-1	405	UUY86236.1 hypothetical protein [Largemouth bass virus]	93.6	47	1.17E-18	2
k141_36062_flag1_multi20.1139_len1177	W4-1	1177	AIG51690.1 major capsid protein, partial [Koi ranavirus]	88.9	54	1.17E-18	77
k141_103740_flag1_multi4.8897_len975	W5-1	975	AYV88122.1 putative 3-beta-hydroxy-delta-5-C27 steroid oxidoreductase-like protein [Mandarin fish ranavirus]	100	46	1.17E-18	8
k141_115784_flag1_multi10.8708_len1921	W5-1	1921	AYV88191.1 hypothetical protein [Mandarin fish ranavirus]	97.8	46	1.20E-18	67
k141_106082_flag1_multi7.9140_len1199	W5-1	1199	UUY86192.1 putative myristylated membrane protein [Largemouth bass virus]	100	43	1.21E-18	30
k141_143735_flag1_multi4.8435_len729	W5-1	729	AAF64582.1 capsid protein, partial [Guppyfish iridovirus]	84.9	53	1.22E-18	32
k141_124142_flag1_multi2.8700_len464	W1-1	464	UUY86199.1 hypothetical protein [Largemouth bass virus]	100	39	1.23E-18	5
k141_6340_flag0_multi12.2043_len1120	W4-1	1120	AIG51690.1 major capsid protein, partial [Koi ranavirus]	97.9	47	1.23E-18	81
k141_147360_flag0_multi43.1424_len1293	W5-1	1293	XRL22790.1 hypothetical protein [Siniperca chuatsi ranavirus]	91.8	49	1.29E-18	180
k141_61364_flag1_multi1.0000_len332	W5-1	332	AYV88191.1 hypothetical protein [Mandarin fish ranavirus]	95	40	1.30E-18	1
k141_77390_flag1_multi6.2881_len443	W5-1	443	QJE49066.1 hypothetical protein LMBV_003 [Largemouth bass virus]	100	46	1.43E-18	5
k141_38997_flag1_multi7.7811_len712	W4-1	712	AYV88134.2 putative tyrosine kinase [Mandarin fish ranavirus]	95.8	48	1.45E-18	20
k141_79395_flag1_multi1.0000_len820	W5-1	820	WEI28998.1 hypothetical protein [Largemouth bass virus]	100	47	1.49E-18	8
k141_60048_flag1_multi1.0000_len355	W6-2	355	AIG51690.1 major capsid protein, partial [Koi ranavirus]	88	50	1.51E-18	5
k141_108648_flag1_multi178.2810_len1027	W5-1	1027	QJE49101.1 hypothetical protein LMBV_038 [Largemouth bass virus]	78.7	61	1.52E-18	13
k141_26757_flag1_multi2.3990_len557	W2-1	557	QJE49144.1 hypothetical protein LMBV_081 [Largemouth bass virus]	100	43	1.55E-18	7
k141_99003_flag1_multi1.9463_len569	W5-1	569	AIG51690.1 major capsid protein, partial [Koi ranavirus]	94	50	1.56E-18	1
k141_130810_flag0_multi1.0000_len688	W5-1	688	UUY86197.1 hypothetical protein [Largemouth bass virus]	100	48	1.57E-18	993.14
k141_60288_flag1_multi2.9494_len1130	W5-1	1130	UUY86235.1 hypothetical protein [Largemouth bass virus]	100	46	1.62E-18	36
k141_15243_flag1_multi7.6239_len1872	W5-1	1872	AYV88120.1 putative p31K protein [Mandarin fish ranavirus]	100	46	1.63E-18	50
k141_57560_flag1_multi1.0000_len351	W1-1	351	XRB52785.1 hypothetical protein LMBV_28 [Largemouth bass virus]	97.4	39	1.71E-18	0
k141_97330_flag1_multi43.9239_len3044	W1-1	3044	QJE49149.1 putative immediate early protein ICP-46 [Largemouth bass virus]	100	46	1.71E-18	429
k141_51689_flag1_multi5.6327_len386	W2-2	386	UUY86223.1 hypothetical protein [Largemouth bass virus]	100	45	1.72E-18	6
k141_44542_flag1_multi1.0000_len425	W5-1	425	AIG51690.1 major capsid protein, partial [Koi ranavirus]	100	45	1.76E-18	3
k141_89673_flag1_multi2.9331_len829	W5-1	829	UUY86227.1 hypothetical protein [Largemouth bass virus]	93.5	46	1.78E-18	9
k141_152840_flag1_multi3.0000_len542	W5-1	542	QJE49077.1 hypothetical protein LMBV_014 [Largemouth bass virus]	90.7	43	1.79E-18	11
k141_82606_flag1_multi1.0000_len341	W1-1	341	UUY86235.1 hypothetical protein [Largemouth bass virus]	95.2	42	1.91E-18	0
k141_95175_flag1_multi10.7442_len1040	W1-1	1040	AYV88134.2 putative tyrosine kinase [Mandarin fish ranavirus]	100	46	2.00E-18	30
k141_44527_flag1_multi1.5561_len711	W5-1	711	QJE49066.1 hypothetical protein LMBV_003 [Largemouth bass virus]	51.9	108	2.00E-18	5
k141_211057_flag1_multi3.0000_len306	W6-3	306	YP_073558.1 RNA-dependent DNA polymerase [lymphocystis disease virus-China]	52.3	86	2.01E-18	2
k141_40141_flag1_multi6.7108_len390	W4-1	390	UUY86229.1 putative DNA dependent RNA polymerase a subunit [Largemouth bass virus]	95.6	45	2.04E-18	8
k141_31785_flag1_multi1.6401_len505	W5-1	505	AYV88169.1 hypothetical protein [Mandarin fish ranavirus]	81.8	55	2.07E-18	4
k141_37295_flag1_multi3.9992_len1411	W5-1	1411	AYV88159.1 hypothetical protein [Mandarin fish ranavirus]	100	46	2.08E-18	29
k141_90518_flag1_multi8.6290_len637	W5-1	637	AYV88211.1 hypothetical protein [Mandarin fish ranavirus]	95.9	49	2.11E-18	16

k141_44836_flag1_multi1.0000_len497	W2-1	497	AYV88176.1 putative tumor necrosis factor receptor [Mandarin fish ranavirus]	100	38	2.14E-18	0
k141_18062_flag1_multi11.7731_len1256	W1-1	1256	AIG51690.1 major capsid protein, partial [Koi ranavirus]	100	47	2.16E-18	29
k141_95761_flag1_multi11.8192_len993	W5-1	993	UUY86218.1 hypothetical protein [Largemouth bass virus]	72.6	62	2.19E-18	53
k141_32285_flag1_multi2.0000_len346	W5-1	346	AYV88191.1 hypothetical protein [Mandarin fish ranavirus]	97.4	39	2.20E-18	1
k141_117699_flag1_multi10.4369_len917	W5-1	917	UUY86221.1 hypothetical protein [Largemouth bass virus]	75.8	62	2.22E-18	28
k141_24372_flag0_multi76.6733_len594	W4-1	594	UUY86261.1 hypothetical protein [Largemouth bass virus]	100	47	2.23E-18	214
k141_139896_flag1_multi16.3333_len345	W5-1	345	AYV88120.1 putative p31K protein [Mandarin fish ranavirus]	97.6	42	2.31E-18	4
k141_141506_flag1_multi4.6654_len930	W5-1	930	AAF64582.1 capsid protein, partial [Guppyfish iridovirus]	95.7	46	2.34E-18	16
k141_40358_flag1_multi1.7080_len391	W5-1	391	AYV88211.1 hypothetical protein [Mandarin fish ranavirus]	89.4	47	2.65E-18	0
k141_99842_flag1_multi2.9024_len510	W1-1	510	AYV88120.1 putative p31K protein [Mandarin fish ranavirus]	95.5	44	2.67E-18	5
k141_16627_flag0_multi1.0000_len249	W4-1	249	AYV88191.1 hypothetical protein [Mandarin fish ranavirus]	100	37	2.67E-18	0.61
k141_1237_flag1_multi7.8530_len1046	W4-1	1046	AYV88120.1 putative p31K protein [Mandarin fish ranavirus]	97.8	45	2.76E-18	25
k141_180778_flag1_multi2.2715_len782	W2-3	782	WHA35533.1 putative DNA dependent RNA polymerase A subunit [Micropterus salmoides ranavirus]	95.9	49	2.77E-18	15
k141_24684_flag1_multi17.5278_len2765	W5-1	2765	AYV88173.2 putative ribonucleotide reductase beta subunit [Mandarin fish ranavirus]	100	46	2.87E-18	153
k141_150415_flag1_multi13.1874_len1235	W5-1	1235	XRB52768.1 P31K protein, partial [Largemouth bass virus]	97.8	46	2.88E-18	66
k141_33261_flag1_multi4.0000_len470	W2-1	470	WEI28972.1 CTD-phosphotransferase [Largemouth bass virus]	66.2	68	2.95E-18	3
k141_40603_flag0_multi1.0000_len334	W2-1	334	WEI28972.1 CTD-phosphotransferase [Largemouth bass virus]	97.7	44	2.99E-18	4.87
k141_46772_flag1_multi20.3106_len508	W5-1	508	UUY86261.1 hypothetical protein [Largemouth bass virus]	97.9	48	3.36E-18	24
k141_29676_flag1_multi5.8031_len725	W5-1	725	AYV88211.1 hypothetical protein [Mandarin fish ranavirus]	97.9	47	3.44E-18	12
k141_124028_flag1_multi4.7322_len1082	W5-1	1082	WEI28972.1 CTD-phosphotransferase [Largemouth bass virus]	100	46	3.45E-18	14
k141_62907_flag1_multi10.8653_len1744	W5-1	1744	UUY86235.1 hypothetical protein [Largemouth bass virus]	100	43	3.47E-18	87
k141_94855_flag1_multi13.0000_len369	W2-1	369	QIZ30887.1 major capsid protein, partial [Largemouth bass virus]	100	39	3.57E-18	11
k141_142375_flag1_multi3.2768_len701	W5-1	701	QJE49215.1 putative orf58-like protein [Largemouth bass virus]	100	41	3.68E-18	11
k141_34289_flag1_multi3.9053_len870	W4-1	870	AYV88120.1 putative p31K protein [Mandarin fish ranavirus]	97.8	46	3.69E-18	11
k141_37964_flag1_multi1.0000_len269	W5-1	269	QYU76034.1 putative myristylated membrane protein, partial [Koi ranavirus]	95.9	49	3.72E-18	0
k141_6474_flag1_multi8.8323_len1238	W5-1	1238	AIG51690.1 major capsid protein, partial [Koi ranavirus]	100	46	3.74E-18	51
k141_13785_flag0_multi14.1896_len1333	W1-1	1333	AIG51690.1 major capsid protein, partial [Koi ranavirus]	92.2	51	3.82E-18	78
k141_5481_flag1_multi1.7681_len624	W1-1	624	WAK75102.1 hypothetical protein [Mandarin fish ranavirus]	97.7	44	4.09E-18	3
k141_22894_flag1_multi5.8341_len834	W4-1	834	AYV88120.1 putative p31K protein [Mandarin fish ranavirus]	97.8	46	4.12E-18	17
k141_148391_flag1_multi9.0888_len614	W5-1	614	AYV88175.2 putative interleukin-1 beta convertase precursor [Mandarin fish ranavirus]	100	44	4.12E-18	21
k141_139912_flag1_multi79.1437_len2354	W5-1	2354	AYV88120.1 putative p31K protein [Mandarin fish ranavirus]	97.8	46	4.16E-18	609
k141_136222_flag1_multi2.8987_len595	W1-1	595	WAK75112.1 putative LITAF PIG7 possible membrane associated motif in LPS-induced tumor necrosis factor alpha factor [Mandarin fish ranavirus]	97.4	39	4.21E-18	2
k141_131438_flag0_multi9.7315_len357	W5-1	357	QYU76034.1 putative myristylated membrane protein, partial [Koi ranavirus]	90	50	4.23E-18	7
k141_126317_flag1_multi2.9147_len1619	W5-1	1619	QJE49125.1 hypothetical protein LMBV_062 [Largemouth bass virus]	100	46	4.36E-18	12
k141_57616_flag1_multi21.6398_len1482	W5-1	1482	AAC79508.1 viral core protein, partial [Ictalurus melas ranavirus]	89.1	46	4.36E-18	78
k141_150497_flag1_multi2.7284_len686	W5-1	686	AYV88169.1 hypothetical protein [Mandarin fish ranavirus]	93.8	48	4.38E-18	10
k141_61989_flag1_multi9.0000_len447	W5-1	447	UUY86241.1 hypothetical protein [Largemouth bass virus]	100	35	4.39E-18	7

k141_127794_flag1_multi10.1663_len1151	W1-1	1151	AYV88120.1 putative p31K protein [Mandarin fish ranavirus]	97.8	45	4.43E-18	67
k141_102706_flag1_multi2.4347_len723	W4-1	723	AIG51690.1 major capsid protein, partial [Koi ranavirus]	97.9	47	4.52E-18	2
k141_103088_flag1_multi1.0000_len519	W5-1	519	AYV88134.2 putative tyrosine kinase [Mandarin fish ranavirus]	100	41	4.66E-18	11
k141_143201_flag1_multi5.0000_len512	W5-1	512	AYV88191.1 hypothetical protein [Mandarin fish ranavirus]	100	39	4.70E-18	7
k141_46760_flag1_multi3.5974_len372	W5-1	372	UUY86261.1 hypothetical protein [Largemouth bass virus]	100	46	4.74E-18	3
k141_139442_flag0_multi12.9149_len6347	W5-1	6347	AYV88159.1 hypothetical protein [Mandarin fish ranavirus]	100	46	4.75E-18	240
k141_162201_flag0_multi16.9544_len1304	W5-1	1304	QJE49066.1 hypothetical protein LMBV_003 [Largemouth bass virus]	78.5	65	4.76E-18	151
k141_47848_flag1_multi1.0000_len279	W1-1	279	UVF58785.1 MAG: major capsid protein [Halichoeres melanurus ranavirus]	97.9	47	4.95E-18	0
k141_90310_flag1_multi4.5094_len622	W4-1	622	WAK75117.1 hypothetical protein [Mandarin fish ranavirus]	90.5	42	5.14E-18	8
k141_94001_flag1_multi1.0000_len330	W5-1	330	WAK75097.1 hypothetical protein [Mandarin fish ranavirus]	100	44	5.17E-18	2
k141_165022_flag0_multi10.3906_len781	W5-1	781	AYV88120.1 putative p31K protein [Mandarin fish ranavirus]	97.7	44	5.32E-18	12
k141_86545_flag1_multi2.5207_len358	W5-1	358	WEI28972.1 CTD-phosphotransferase [Largemouth bass virus]	100	44	5.49E-18	0
k141_139615_flag0_multi1.0000_len392	W5-1	392	XRB52831.1 hypothetical protein LMBV_74 [Largemouth bass virus]	91.7	60	5.51E-18	1177.81
k141_57817_flag0_multi1.0000_len302	W5-1	302	UUY86258.1 hypothetical protein [Largemouth bass virus]	100	46	5.60E-18	3.35
k141_154340_flag1_multi6.9395_len1033	W5-1	1033	AFD96401.1 major capsid protein, partial [Largemouth bass virus]	97.7	43	5.76E-18	20
k141_62718_flag1_multi47.8887_len4373	W1-1	4373	AIG51690.1 major capsid protein, partial [Koi ranavirus]	80.6	62	5.93E-18	535
k141_97305_flag1_multi5.9015_len801	W1-1	801	AIG51690.1 major capsid protein, partial [Koi ranavirus]	100	44	5.97E-18	16
k141_72208_flag1_multi2.9214_len421	W5-1	421	AIG51690.1 major capsid protein, partial [Koi ranavirus]	100	43	6.04E-18	1
k141_17063_flag1_multi1.0000_len428	W5-1	428	AYV88120.1 putative p31K protein [Mandarin fish ranavirus]	97.6	42	6.09E-18	4.16
k141_83280_flag1_multi4.8943_len898	W2-1	898	AIG51690.1 major capsid protein, partial [Koi ranavirus]	95.8	48	6.11E-18	17
k141_131931_flag0_multi9.7783_len1968	W1-1	1968	UUY86204.1 hypothetical protein [Largemouth bass virus]	100	47	6.36E-18	137
k141_128886_flag1_multi18.9667_len1223	W5-1	1223	QJE49077.1 hypothetical protein LMBV_014 [Largemouth bass virus]	100	39	6.43E-18	61
k141_81064_flag0_multi1.0000_len281	W4-1	281	UVF58785.1 MAG: major capsid protein [Halichoeres melanurus ranavirus]	100	46	6.79E-18	0
k141_158485_flag1_multi10.5150_len4904	W5-1	4904	AYV88179.2 putative DNA dependent RNA polymerase II second largest subunit [Mandarin fish ranavirus]	78.8	66	6.83E-18	183
k141_46668_flag1_multi53.7912_len4423	W4-1	4423	WAK75068.1 hypothetical protein [Mandarin fish ranavirus]	90	50	6.90E-18	181
k141_31549_flag1_multi4.0000_len687	W1-1	687	QYU76034.1 putative myristylated membrane protein, partial [Koi ranavirus]	89.8	49	7.01E-18	5
k141_84657_flag1_multi7.7402_len599	W2-1	599	QJE49065.1 hypothetical protein LMBV_002 [Largemouth bass virus]	97.5	40	7.32E-18	13
k141_113969_flag1_multi4.9725_len1051	W5-1	1051	QJE49146.1 putative thiol oxidoreductase [Largemouth bass virus]	100	42	7.68E-18	10
k141_112534_flag1_multi1.0000_len444	W5-1	444	AAC79876.1 viral core protein, partial [Labroides dimidatus ranavirus]	100	39	7.71E-18	5.01
k141_32600_flag1_multi1.9055_len416	W4-2	416	AAF64582.1 capsid protein, partial [Guppyfish iridovirus]	97.6	42	7.76E-18	0
k141_38993_flag0_multi1.0000_len279	W1-1	279	AIG51690.1 major capsid protein, partial [Koi ranavirus]	97.9	47	7.80E-18	6
k141_25207_flag1_multi3.6233_len1264	W5-1	1264	UUY86195.1 hypothetical protein [Largemouth bass virus]	97.8	46	7.85E-18	10
k141_9421_flag1_multi13.0000_len1334	W4-1	1334	QIZ30887.1 major capsid protein, partial [Largemouth bass virus]	52.6	95	7.91E-18	47
k141_20686_flag1_multi1.0000_len572	W5-1	572	AIG51690.1 major capsid protein, partial [Koi ranavirus]	100	46	8.10E-18	4
k141_13992_flag1_multi11.1116_len2158	W5-1	2158	XRB52797.1 Bcl-2 Bcl-2 homology region 1-3 domain-containing protein [Largemouth bass virus]	97.8	46	8.16E-18	80
k141_130371_flag1_multi8.6349_len897	W1-1	897	AIG51690.1 major capsid protein, partial [Koi ranavirus]	97.8	46	8.22E-18	22
k141_39772_flag1_multi10.7624_len1067	W4-1	1067	AIG51690.1 major capsid protein, partial [Koi ranavirus]	97.9	47	8.42E-18	95.29
k141_73391_flag0_multi8.5429_len526	W4-1	526	WAK75070.1 putative neurofilament triplet H1-like protein [Mandarin fish ranavirus]	77.2	57	8.66E-18	10

k141_12611_flag0_multi28.3574_len6283	W5-1	6283	UUY86235.1 hypothetical protein [Largemouth bass virus]	100	46	8.68E-18	615
k141_109160_flag1_multi8.9106_len2200	W1-2	2200	WEI29006.1 putative 2-cysteine adaptor domain protein [Largemouth bass virus]	95.8	48	8.73E-18	143
k141_65526_flag1_multi4.7254_len476	W5-1	476	UUY86258.1 hypothetical protein [Largemouth bass virus]	100	46	9.15E-18	8
k141_21644_flag1_multi2.1626_len547	W5-1	547	UUY86192.1 putative myristylated membrane protein [Largemouth bass virus]	77.1	48	9.22E-18	6
k141_164582_flag1_multi8.7875_len781	W5-1	781	QIZ30887.1 major capsid protein, partial [Largemouth bass virus]	95.2	42	9.80E-18	23
k141_86031_flag0_multi9.1831_len354	W4-1	354	AYV88198.1 hypothetical protein [Mandarin fish ranavirus]	97.7	43	9.93E-18	5
k141_53926_flag1_multi3.3996_len609	W5-1	609	QJE49153.1 hypothetical protein LMBV_004 [Largemouth bass virus]	97.8	46	1.01E-17	5
k141_20801_flag1_multi4.3269_len1019	W6-1	1019	QJE49077.1 hypothetical protein LMBV_014 [Largemouth bass virus]	97.6	41	1.03E-17	90.04
k141_132287_flag1_multi3.8763_len796	W5-1	796	AYV88121.1 hypothetical protein [Mandarin fish ranavirus]	97.8	46	1.08E-17	4
k141_35806_flag1_multi7.8591_len1284	W5-1	1284	WEI29006.1 putative 2-cysteine adaptor domain protein [Largemouth bass virus]	93.9	49	1.14E-17	42
k141_6300_flag1_multi42.0000_len1711	W5-1	1711	AYV88159.1 hypothetical protein [Mandarin fish ranavirus]	100	44	1.15E-17	294
k141_145305_flag1_multi12.9370_len760	W6-2	760	UUY86192.1 putative myristylated membrane protein [Largemouth bass virus]	100	38	1.15E-17	24
k141_14675_flag1_multi2.3534_len605	W2-3	605	AYV88179.2 putative DNA dependent RNA polymerase II second largest subunit [Mandarin fish ranavirus]	100	43	1.16E-17	6
k141_82705_flag1_multi8.9520_len3976	W5-1	3976	AAF64582.1 capsid protein, partial [Guppyfish iridovirus]	93.8	48	1.16E-17	102
k141_152249_flag1_multi4.9223_len450	W5-1	450	QIZ30887.1 major capsid protein, partial [Largemouth bass virus]	92.9	42	1.19E-17	0
k141_131316_flag1_multi2.9216_len728	W5-1	728	UVF58785.1 MAG: major capsid protein [Halichoeres melanurus ranavirus]	85.5	55	1.19E-17	6
k141_72809_flag0_multi1.0000_len289	W1-1	289	AIG51690.1 major capsid protein, partial [Koi ranavirus]	100	45	1.21E-17	0
k141_125819_flag1_multi4.0000_len455	W5-1	455	QIZ30887.1 major capsid protein, partial [Largemouth bass virus]	100	39	1.27E-17	4
k141_92906_flag1_multi4.0000_len802	W4-3	802	WEI29006.1 putative 2-cysteine adaptor domain protein [Largemouth bass virus]	100	39	1.35E-17	10
k141_96520_flag0_multi39.8919_len3804	W2-3	3804	AYV88120.1 putative p31K protein [Mandarin fish ranavirus]	97.8	46	1.38E-17	569.21
k141_60042_flag1_multi1.0000_len395	W2-1	395	QJE49101.1 hypothetical protein LMBV_038 [Largemouth bass virus]	93.3	45	1.48E-17	3
k141_19136_flag1_multi38.9071_len9545	W1-1	9545	AYV88120.1 putative p31K protein [Mandarin fish ranavirus]	97.8	46	1.54E-17	1062
k141_22565_flag1_multi9.9204_len2743	W1-1	2743	XRB52768.1 P31K protein, partial [Largemouth bass virus]	95.7	47	1.57E-17	99
k141_10215_flag1_multi5.3256_len1342	W6-1	1342	UVF58793.1 MAG: DNA polymerase [Halichoeres melanurus ranavirus]	100	45	1.59E-17	20
k141_71523_flag1_multi6.8311_len958	W4-1	958	AIG51690.1 major capsid protein, partial [Koi ranavirus]	100	46	1.62E-17	18
k141_158086_flag1_multi1.0000_len415	W5-1	415	WAK75112.1 putative LITAF PIG7 possible membrane associated motif in LPS-induced tumor necrosis factor alpha factor [Mandarin fish ranavirus]	100	38	1.62E-17	1
k141_138710_flag1_multi1.0000_len312	W5-1	312	AIG51690.1 major capsid protein, partial [Koi ranavirus]	100	41	1.64E-17	0
k141_46274_flag1_multi2.9117_len526	W4-1	526	UVF58785.1 MAG: major capsid protein [Halichoeres melanurus ranavirus]	100	46	1.66E-17	5
k141_11826_flag1_multi5.9123_len369	W4-1	369	AYV88120.1 putative p31K protein [Mandarin fish ranavirus]	91.1	45	1.66E-17	0
k141_165579_flag0_multi29.1505_len513	W5-1	513	UUY86261.1 hypothetical protein [Largemouth bass virus]	100	46	1.66E-17	124
k141_102827_flag1_multi4.8655_len825	W5-1	825	XRL22790.1 hypothetical protein [Siniperca chuatsi ranavirus]	97.9	48	1.73E-17	12
k141_25020_flag1_multi13.0000_len342	W4-1	342	AYV88120.1 putative p31K protein [Mandarin fish ranavirus]	100	39	1.75E-17	8
k141_33871_flag1_multi4.8874_len958	W1-1	958	QJE49088.1 hypothetical protein LMBV_025 [Largemouth bass virus]	100	46	1.80E-17	9
k141_159368_flag1_multi5.0633_len899	W5-1	899	WAK75112.1 putative LITAF PIG7 possible membrane associated motif in LPS-induced tumor necrosis factor alpha factor [Mandarin fish ranavirus]	95	40	1.85E-17	12
k141_57164_flag0_multi8.4210_len761	W5-1	761	QJE49137.1 hypothetical protein LMBV_074 [Largemouth bass virus]	97.8	46	1.92E-17	16
k141_102591_flag1_multi15.9692_len2313	W1-1	2313	WEI28972.1 CTD-phosphotransferase [Largemouth bass virus]	95.8	48	1.93E-17	106
k141_28809_flag1_multi3.7669_len437	W5-1	437	AIG51690.1 major capsid protein, partial [Koi ranavirus]	69.4	62	1.95E-17	5

k141_32212_flag1_multi1.0000_len269	W5-1	269	UVF58785.1 MAG: major capsid protein [Halichoeres melanurus ranavirus]	100	42	2.07E-17	0
k141_21777_flag0_multi7.6455_len1340	W4-1	1340	AYV88134.2 putative tyrosine kinase [Mandarin fish ranavirus]	97.7	44	2.10E-17	30
k141_120678_flag1_multi2.0000_len640	W5-1	640	AYV88118.1 hypothetical protein [Mandarin fish ranavirus]	100	39	2.15E-17	6.1
k141_30342_flag0_multi6.1799_len1992	W2-1	1992	UUY86241.1 hypothetical protein [Largemouth bass virus]	84.1	44	2.18E-17	82.1
k141_64205_flag1_multi5.3921_len544	W5-1	544	QJE49137.1 hypothetical protein LMBV_074 [Largemouth bass virus]	100	41	2.18E-17	11
k141_133439_flag1_multi1.0000_len511	W5-1	511	QIZ30887.1 major capsid protein, partial [Largemouth bass virus]	97.4	39	2.19E-17	37
k141_63973_flag0_multi1.0000_len281	W5-1	281	QJE49073.1 putative p31K protein [Largemouth bass virus]	100	46	2.25E-17	0
k141_130104_flag1_multi3.0000_len721	W5-1	721	AAC79876.1 viral core protein, partial [Labroides dimidatus ranavirus]	100	39	2.28E-17	9
k141_40111_flag1_multi1.0000_len313	W1-1	313	AYV88120.1 putative p31K protein [Mandarin fish ranavirus]	97.6	42	2.30E-17	2
k141_32063_flag1_multi3.2065_len417	W4-1	417	QJE49137.1 hypothetical protein LMBV_074 [Largemouth bass virus]	79.2	53	2.45E-17	3
k141_79270_flag1_multi1.0000_len842	W5-1	842	UUY86235.1 hypothetical protein [Largemouth bass virus]	100	46	2.47E-17	6
k141_166906_flag1_multi1.0000_len354	W5-1	354	QJE49110.1 hypothetical protein LMBV_047 [Largemouth bass virus]	95.3	43	2.53E-17	4
k141_125442_flag1_multi1.0000_len387	W1-1	387	UUY86197.1 hypothetical protein [Largemouth bass virus]	100	41	2.56E-17	2
k141_48830_flag1_multi5.9643_len2382	W5-1	2382	UUY86266.1 hypothetical protein [Largemouth bass virus]	97.7	44	2.56E-17	54
k141_33634_flag0_multi1.0000_len425	W5-1	425	WEI29006.1 putative 2-cysteine adaptor domain protein [Largemouth bass virus]	100	40	2.63E-17	25
k141_27192_flag1_multi1.0000_len790	W5-1	790	AIG51690.1 major capsid protein, partial [Koi ranavirus]	97.6	42	2.69E-17	21
k141_80145_flag1_multi9.7650_len1022	W5-1	1022	AYV88211.1 hypothetical protein [Mandarin fish ranavirus]	100	46	2.74E-17	28
k141_146674_flag1_multi3.9359_len1218	W5-1	1218	WEI29006.1 putative 2-cysteine adaptor domain protein [Largemouth bass virus]	97.8	46	2.75E-17	14
k141_135011_flag0_multi1.0000_len309	W5-1	309	UUY86235.1 hypothetical protein [Largemouth bass virus]	100	43	2.77E-17	3
k141_41495_flag1_multi1.0000_len599	W5-1	599	UUY86229.1 putative DNA dependent RNA polymerase a subunit [Largemouth bass virus]	97.7	44	2.82E-17	6
k141_66947_flag1_multi5.1094_len461	W4-1	461	XRL22821.1 hypothetical protein [Siniperca chuatsi ranavirus]	100	40	2.96E-17	3
k141_5690_flag1_multi5.0000_len662	W5-1	662	AFD96401.1 major capsid protein, partial [Largemouth bass virus]	100	39	2.99E-17	6
k141_137750_flag1_multi24.2398_len5128	W1-1	5128	QJE49100.1 hypothetical protein LMBV_037 [Largemouth bass virus]	97.9	47	3.07E-17	266
k141_145654_flag1_multi5.8594_len781	W2-3	781	QJE49100.1 hypothetical protein LMBV_037 [Largemouth bass virus]	100	45	3.07E-17	14
k141_115049_flag1_multi5.7444_len591	W5-1	591	UUY86197.1 hypothetical protein [Largemouth bass virus]	100	46	3.10E-17	3
k141_43274_flag1_multi32.8463_len798	W5-1	798	WEI28972.1 CTD-phosphotransferase [Largemouth bass virus]	100	46	3.16E-17	91
k141_128496_flag1_multi3.8428_len580	W1-1	580	UUY86261.1 hypothetical protein [Largemouth bass virus]	100	46	3.30E-17	7
k141_99631_flag1_multi17.4635_len868	W4-1	868	UUY86211.1 hypothetical protein [Largemouth bass virus]	67.1	73	3.31E-17	35
k141_103457_flag1_multi7.0000_len588	W5-1	588	QIZ30887.1 major capsid protein, partial [Largemouth bass virus]	97.5	40	3.31E-17	12
k141_163008_flag1_multi7.9148_len2689	W5-1	2689	UUY86238.1 hypothetical protein [Largemouth bass virus]	100	47	3.44E-17	94
k141_54192_flag1_multi1.9558_len594	W5-1	594	AYV88134.2 putative tyrosine kinase [Mandarin fish ranavirus]	100	43	3.45E-17	3
k141_28118_flag1_multi7.0000_len658	W5-1	658	AYV88191.1 hypothetical protein [Mandarin fish ranavirus]	97.4	39	3.49E-17	19
k141_146266_flag1_multi13.7506_len2270	W5-1	2270	QJE49101.1 hypothetical protein LMBV_038 [Largemouth bass virus]	97.8	46	3.52E-17	107
k141_113158_flag1_multi1.0000_len628	W1-1	628	AIG51690.1 major capsid protein, partial [Koi ranavirus]	100	42	3.60E-17	20
k141_94472_flag0_multi1.0000_len350	W4-1	350	AYV88191.1 hypothetical protein [Mandarin fish ranavirus]	100	37	3.65E-17	7
k141_32956_flag1_multi6.5382_len821	W1-1	821	WEI28972.1 CTD-phosphotransferase [Largemouth bass virus]	70	60	3.69E-17	23
k141_28448_flag1_multi9.9081_len2393	W5-1	2393	AIG51690.1 major capsid protein, partial [Koi ranavirus]	100	46	3.74E-17	91
k141_139266_flag1_multi1.0000_len352	W2-3	352	AYV88136.2 hypothetical protein [Mandarin fish ranavirus]	83.7	43	3.86E-17	4
k141_67328_flag1_multi3.3333_len1215	W1-1	1215	WXI69503.1 hypothetical protein [Largemouth bass virus]	90	50	4.09E-17	48

k141_90250_flag1_multi17.8899_len1222	W1-1	1222	XRL22821.1 hypothetical protein [Siniperca chuatsi ranavirus]	97.7	43	4.13E-17	76
k141_26547_flag1_multi1.0000_len483	W5-1	483	AYV88120.1 putative p31K protein [Mandarin fish ranavirus]	78.3	60	4.15E-17	6.93
k141_6950_flag1_multi6.0000_len361	W4-1	361	AYV88191.1 hypothetical protein [Mandarin fish ranavirus]	100	35	4.20E-17	6
k141_69043_flag1_multi68.0000_len756	W1-1	756	UUY86199.1 hypothetical protein [Largemouth bass virus]	97.4	39	4.27E-17	162
k141_3251_flag1_multi27.0000_len3550	W5-1	3550	QJE49114.1 hypothetical protein LMBV_051 [Largemouth bass virus]	100	39	4.27E-17	254
k141_90351_flag1_multi4.0312_len494	W2-1	494	AYV88191.1 hypothetical protein [Mandarin fish ranavirus]	92.5	40	4.36E-17	5
k141_112600_flag1_multi0.9596_len364	W5-1	364	AIG51690.1 major capsid protein, partial [Koi ranavirus]	89.8	49	4.36E-17	1
k141_29195_flag0_multi1.0000_len336	W5-1	336	UVF58793.1 MAG: DNA polymerase [Halichoeres melanurus ranavirus]	75.9	58	4.43E-17	2
k141_107110_flag1_multi2.2789_len539	W1-1	539	UUY86267.1 ribonucleotide reductase alpha subunit [Largemouth bass virus]	100	40	4.46E-17	2
k141_107627_flag1_multi8.3654_len1917	W5-1	1917	UUY86267.1 ribonucleotide reductase alpha subunit [Largemouth bass virus]	100	42	4.46E-17	56
k141_44620_flag1_multi7.9055_len2290	W5-1	2290	WEI28972.1 CTD-phosphotransferase [Largemouth bass virus]	100	46	4.48E-17	51
k141_57867_flag0_multi16.8760_len270	W5-1	270	XRL22821.1 hypothetical protein [Siniperca chuatsi ranavirus]	100	39	4.61E-17	0
k141_44665_flag1_multi5.0000_len821	W5-1	821	QIZ30887.1 major capsid protein, partial [Largemouth bass virus]	85.4	48	4.87E-17	7
k141_48159_flag0_multi56.2530_len1157	W5-1	1157	WEI29006.1 putative 2-cysteine adaptor domain protein [Largemouth bass virus]	92.2	51	5.02E-17	223
k141_130472_flag1_multi6.2425_len607	W5-1	607	UVF58785.1 MAG: major capsid protein [Halichoeres melanurus ranavirus]	100	44	5.13E-17	108
k141_87328_flag1_multi14.7718_len1552	W2-1	1552	AIG51690.1 major capsid protein, partial [Koi ranavirus]	100	46	5.29E-17	69
k141_129593_flag1_multi1.0000_len327	W1-1	327	AIG51690.1 major capsid protein, partial [Koi ranavirus]	70.6	68	5.43E-17	3
k141_29442_flag1_multi3.0000_len374	W4-1	374	AFD96401.1 major capsid protein, partial [Largemouth bass virus]	97.4	39	5.53E-17	1
k141_80451_flag1_multi2.8762_len545	W2-1	545	AYV88120.1 putative p31K protein [Mandarin fish ranavirus]	100	46	5.55E-17	13
k141_114671_flag1_multi18.0000_len867	W5-1	867	UUY86235.1 hypothetical protein [Largemouth bass virus]	45	131	5.56E-17	57
k141_94858_flag1_multi8.9366_len1276	W4-1	1276	AYV88191.1 hypothetical protein [Mandarin fish ranavirus]	97.6	42	5.68E-17	34
k141_29210_flag1_multi11.9377_len4024	W5-1	4024	UUY86199.1 hypothetical protein [Largemouth bass virus]	97.4	39	5.71E-17	146
k141_17083_flag1_multi2.9369_len870	W5-1	870	UUY86269.1 hypothetical protein [Largemouth bass virus]	100	46	5.74E-17	8
k141_29947_flag0_multi19.0692_len488	W5-1	488	QJE49066.1 hypothetical protein LMBV_003 [Largemouth bass virus]	81.1	53	5.78E-17	10
k141_49804_flag1_multi7.0000_len540	W4-1	540	QIZ30887.1 major capsid protein, partial [Largemouth bass virus]	100	38	5.82E-17	9
k141_80896_flag1_multi2.0000_len499	W5-1	499	UUY86235.1 hypothetical protein [Largemouth bass virus]	93	43	6.03E-17	2
k141_1454_flag0_multi12.7577_len595	W4-1	595	AYV88199.1 hypothetical protein [Mandarin fish ranavirus]	80.4	51	6.55E-17	8
k141_158190_flag1_multi1.0000_len701	W5-1	701	AIG51690.1 major capsid protein, partial [Koi ranavirus]	100	47	6.64E-17	5
k141_21114_flag1_multi1.0000_len400	W1-1	400	QJE49081.1 hypothetical protein LMBV_018 [Largemouth bass virus]	100	39	7.02E-17	0
k141_146365_flag1_multi18.1417_len2413	W5-1	2413	QJE49112.1 hypothetical protein LMBV_049 [Largemouth bass virus]	100	39	7.07E-17	140
k141_142252_flag1_multi3.1546_len665	W5-1	665	UUY86261.1 hypothetical protein [Largemouth bass virus]	95.8	48	7.08E-17	7
k141_1511_flag1_multi6.9478_len486	W1-1	486	UVF58793.1 MAG: DNA polymerase [Halichoeres melanurus ranavirus]	95.5	44	7.17E-17	19
k141_31219_flag1_multi49.6569_len3426	W4-1	3426	CBW45581.1 hypothetical protein, partial [Doctor fish virus]	95.7	46	7.18E-17	234
k141_108759_flag1_multi2.6060_len872	W5-1	872	UUY86251.1 hypothetical protein [Largemouth bass virus]	100	40	7.22E-17	13
k141_72955_flag0_multi1.0000_len281	W2-3	281	ABA41591.1 DNA-dependent DNA polymerase, partial [Largemouth bass virus]	95.7	46	7.31E-17	0
k141_145588_flag0_multi7.8519_len7752	W5-1	7752	XRL22821.1 hypothetical protein [Siniperca chuatsi ranavirus]	100	46	7.35E-17	224
k141_4266_flag1_multi5.0000_len847	W5-1	847	WAK75112.1 putative LITAF PIG7 possible membrane associated motif in LPS-induced tumor necrosis factor alpha factor [Mandarin fish ranavirus]	94.7	38	7.39E-17	34
k141_46904_flag1_multi3.7675_len799	W1-2	799	WAK75112.1 putative LITAF PIG7 possible membrane associated motif in LPS-induced tumor necrosis factor alpha factor [Mandarin fish ranavirus]	100	36	7.72E-17	11

k141_104030_flag1_multi4.0000_len1097	W5-1	1097	AYV88168.1 hypothetical protein [Mandarin fish ranavirus]	79.6	54	7.83E-17	16
k141_141326_flag1_multi1.0000_len679	W5-1	679	UUY86235.1 hypothetical protein [Largemouth bass virus]	100	44	7.90E-17	23.86
k141_98638_flag1_multi4.3280_len955	W5-1	955	QJE49137.1 hypothetical protein LMBV_074 [Largemouth bass virus]	95.9	49	7.98E-17	14
k141_20173_flag1_multi5.8591_len957	W4-1	957	AYV88211.1 hypothetical protein [Mandarin fish ranavirus]	100	46	8.30E-17	17
k141_105310_flag1_multi8.8218_len2565	W5-1	2565	WXI69548.1 hypothetical protein [Largemouth bass virus]	97.8	45	8.48E-17	66
k141_75013_flag1_multi1.6799_len419	W5-1	419	UUY86192.1 putative myristylated membrane protein [Largemouth bass virus]	57.3	82	8.74E-17	1
k141_33788_flag0_multi1.0000_len380	W1-1	380	QJE49100.1 hypothetical protein LMBV_037 [Largemouth bass virus]	100	42	8.79E-17	0
k141_39572_flag0_multi1.0000_len328	W1-1	328	QYU76036.1 helicase-like protein, partial [Koi ranavirus]	97.7	44	8.86E-17	0
k141_11156_flag1_multi4.9327_len587	W5-1	587	AIG51690.1 major capsid protein, partial [Koi ranavirus]	89.6	48	8.91E-17	10
k141_32255_flag0_multi1.0000_len259	W1-1	259	WEI28972.1 CTD-phosphotransferase [Largemouth bass virus]	100	38	9.10E-17	0
k141_75320_flag1_multi9.7875_len861	W1-1	861	QJE49101.1 hypothetical protein LMBV_038 [Largemouth bass virus]	100	46	9.28E-17	18
k141_145719_flag1_multi3.8642_len627	W5-1	627	UUY86266.1 hypothetical protein [Largemouth bass virus]	100	40	9.53E-17	4
k141_110897_flag1_multi2.0000_len636	W5-1	636	QIZ30887.1 major capsid protein, partial [Largemouth bass virus]	100	39	9.85E-17	4
k141_15468_flag1_multi1.0000_len498	W5-1	498	UUY86258.1 hypothetical protein [Largemouth bass virus]	100	46	1.00E-16	11
k141_2569_flag1_multi1.0000_len459	W1-1	459	XRB52768.1 P31K protein, partial [Largemouth bass virus]	92.9	42	1.05E-16	0
k141_21189_flag0_multi1.0000_len423	W1-1	423	XRL22790.1 hypothetical protein [Siniperca chuatsi ranavirus]	97.7	43	1.06E-16	1
k141_52319_flag1_multi6.4279_len1882	W5-1	1882	WHA35537.1 hypothetical protein MSRaV_49L [Micropterus salmoides ranavirus]	100	41	1.09E-16	52
k141_16876_flag1_multi8.8906_len1457	W2-3	1457	WHA35533.1 putative DNA dependent RNA polymerase A subunit [Micropterus salmoides ranavirus]	100	45	1.12E-16	40
k141_79075_flag1_multi9.9304_len9327	W5-1	9327	UUY86267.1 ribonucleotide reductase alpha subunit [Largemouth bass virus]	100	46	1.12E-16	299
k141_85909_flag1_multi1.0000_len506	W5-1	506	UUY86227.1 hypothetical protein [Largemouth bass virus]	97.6	41	1.13E-16	17.96
k141_100184_flag1_multi13.0000_len1264	W1-1	1264	QJE49215.1 putative orf58-like protein [Largemouth bass virus]	100	39	1.16E-16	62
k141_166990_flag1_multi4.8209_len610	W5-1	610	QJE49119.1 hypothetical protein LMBV_056 [Largemouth bass virus]	100	40	1.19E-16	10
k141_41563_flag1_multi1.0000_len767	W5-1	767	UUY86261.1 hypothetical protein [Largemouth bass virus]	97.9	47	1.19E-16	9
k141_16896_flag0_multi1.0000_len317	W4-1	317	UUY86235.1 hypothetical protein [Largemouth bass virus]	90.9	44	1.21E-16	2
k141_37862_flag1_multi4.9411_len1703	W4-1	1703	WEI29006.1 putative 2-cysteine adaptor domain protein [Largemouth bass virus]	100	46	1.21E-16	51.45
k141_65113_flag0_multi1.0000_len282	W5-1	282	UUY86218.1 hypothetical protein [Largemouth bass virus]	97.3	37	1.25E-16	0
k141_149610_flag0_multi59.1296_len8706	W5-1	8706	UUY86199.1 hypothetical protein [Largemouth bass virus]	86.7	45	1.26E-16	1636
k141_101341_flag1_multi2.0000_len708	W6-1	708	UUY86241.1 hypothetical protein [Largemouth bass virus]	100	35	1.28E-16	5
k141_28902_flag1_multi3.1193_len560	W4-2	560	AAC79876.1 viral core protein, partial [Labroides dimidatus ranavirus]	97.4	38	1.32E-16	5
k141_55374_flag1_multi3.4992_len1415	W5-1	1415	AIG51690.1 major capsid protein, partial [Koi ranavirus]	100	46	1.33E-16	27
k141_28670_flag1_multi8.6878_len756	W5-1	756	AYV88191.1 hypothetical protein [Mandarin fish ranavirus]	97.5	40	1.41E-16	15
k141_21924_flag1_multi2.9193_len364	W5-1	364	UUY86258.1 hypothetical protein [Largemouth bass virus]	95.2	42	1.43E-16	0
k141_89977_flag1_multi3.8313_len390	W5-1	390	UUY86258.1 hypothetical protein [Largemouth bass virus]	100	43	1.44E-16	3
k141_105718_flag0_multi1.0000_len464	W5-1	464	WAK75112.1 putative LITAF PIG7 possible membrane associated motif in LPS-induced tumor necrosis factor alpha factor [Mandarin fish ranavirus]	97.2	36	1.52E-16	1
k141_125792_flag1_multi3.0000_len620	W5-1	620	AYV88120.1 putative p31K protein [Mandarin fish ranavirus]	92.9	42	1.53E-16	5
k141_80241_flag1_multi20.5864_len2235	W5-1	2235	QJE49066.1 hypothetical protein LMBV_003 [Largemouth bass virus]	100	46	1.54E-16	96
k141_40241_flag1_multi13.8005_len1314	W4-1	1314	QJE49095.1 hypothetical protein LMBV_032 [Largemouth bass virus]	100	45	1.57E-16	58

k141_42207_flag1_multi10.6974_len802	W4-1	802	AYV88179.2 putative DNA dependent RNA polymerase II second largest subunit [Mandarin fish ranavirus]	92	50	1.60E-16	27
k141_43914_flag1_multi1.0000_len314	W5-1	314	QYU76034.1 putative myristylated membrane protein, partial [Koi ranavirus]	100	44	1.61E-16	0
k141_116521_flag1_multi1.5312_len1374	W5-1	1374	AIG51690.1 major capsid protein, partial [Koi ranavirus]	100	44	1.62E-16	19
k141_198576_flag1_multi1.0000_len335	W5-3	335	WEI28972.1 CTD-phosphotransferase [Largemouth bass virus]	89.1	46	1.79E-16	0
k141_83004_flag1_multi3.0000_len458	W5-1	458	WEI29006.1 putative 2-cysteine adaptor domain protein [Largemouth bass virus]	90.9	44	1.80E-16	3
k141_6841_flag1_multi38.5964_len1355	W5-1	1355	QJE49137.1 hypothetical protein LMBV_074 [Largemouth bass virus]	92	50	1.82E-16	165
k141_4697_flag1_multi18.8475_len3800	W5-1	3800	QYU76034.1 putative myristylated membrane protein, partial [Koi ranavirus]	100	46	1.82E-16	226
k141_117638_flag0_multi1.0000_len230	W5-1	230	UUY86218.1 hypothetical protein [Largemouth bass virus]	97.3	37	1.83E-16	0
k141_160227_flag1_multi1.0000_len420	W5-1	420	XPZ21261.1 hypothetical protein MRVORF002 [Mandarin fish ranavirus]	95.3	43	1.89E-16	2
k141_133_flag0_multi52.6393_len385	W5-1	385	UVF58793.1 MAG: DNA polymerase [Halichoeres melanurus ranavirus]	97.6	42	2.02E-16	0
k141_42937_flag1_multi1.0000_len517	W5-1	517	WEI28972.1 CTD-phosphotransferase [Largemouth bass virus]	97.5	40	2.03E-16	0
k141_12442_flag0_multi20.6203_len457	W5-1	457	UUY86235.1 hypothetical protein [Largemouth bass virus]	100	41	2.14E-16	1
k141_166405_flag1_multi2.1808_len600	W5-1	600	UUY86235.1 hypothetical protein [Largemouth bass virus]	95.6	45	2.14E-16	7
k141_109320_flag1_multi2.6777_len563	W1-1	563	UVF58785.1 MAG: major capsid protein [Halichoeres melanurus ranavirus]	100	46	2.21E-16	6
k141_25017_flag1_multi5.0229_len926	W5-1	926	UVF58785.1 MAG: major capsid protein [Halichoeres melanurus ranavirus]	97.9	48	2.24E-16	16
k141_30241_flag1_multi5.7862_len562	W4-1	562	WHA35533.1 putative DNA dependent RNA polymerase A subunit [Micropterus salmoides ranavirus]	100	45	2.28E-16	12
k141_108736_flag1_multi6.6396_len990	W5-1	990	QJE49121.1 hypothetical protein LMBV_058 [Largemouth bass virus]	81.5	54	2.44E-16	11
k141_65110_flag0_multi1.0000_len282	W5-1	282	UUY86218.1 hypothetical protein [Largemouth bass virus]	90	40	2.50E-16	0
k141_85666_flag1_multi7.0861_len2953	W5-1	2953	AAC79876.1 viral core protein, partial [Labroides dimidatus ranavirus]	93	43	2.53E-16	68
k141_22524_flag0_multi58.0999_len1623	W6-1	1623	XRL22821.1 hypothetical protein [Siniperca chuatsi ranavirus]	100	46	2.69E-16	320
k141_58615_flag1_multi5.5101_len488	W5-3	488	UUY86197.1 hypothetical protein [Largemouth bass virus]	91.5	47	2.72E-16	27
k141_47628_flag1_multi1.0000_len1001	W5-1	1001	AYV88120.1 putative p31K protein [Mandarin fish ranavirus]	100	46	2.75E-16	84
k141_153913_flag1_multi1.0000_len387	W5-1	387	WHA35533.1 putative DNA dependent RNA polymerase A subunit [Micropterus salmoides ranavirus]	95.7	46	2.81E-16	1
k141_78210_flag1_multi5.7970_len683	W5-1	683	AIG51690.1 major capsid protein, partial [Koi ranavirus]	100	43	2.83E-16	11
k141_167057_flag1_multi0.9750_len341	W5-1	341	QJE49096.1 putative D5 family NTPase/ATPase [Largemouth bass virus]	89.1	46	2.97E-16	1
k141_18651_flag1_multi5.8541_len614	W2-1	614	AIG51690.1 major capsid protein, partial [Koi ranavirus]	58.8	85	2.98E-16	12
k141_62962_flag1_multi6.8494_len1004	W5-1	1004	UUY86261.1 hypothetical protein [Largemouth bass virus]	100	46	2.99E-16	31
k141_100469_flag1_multi10.9806_len8382	W5-1	8382	UUY86258.1 hypothetical protein [Largemouth bass virus]	100	44	3.11E-16	314
k141_106834_flag1_multi1.0000_len453	W5-1	453	WEI29006.1 putative 2-cysteine adaptor domain protein [Largemouth bass virus]	88.6	44	3.12E-16	0
k141_158706_flag0_multi9.7062_len1424	W5-1	1424	QJE49125.1 hypothetical protein LMBV_062 [Largemouth bass virus]	100	43	3.18E-16	35
k141_154795_flag1_multi4.6090_len788	W5-1	788	QJE49101.1 hypothetical protein LMBV_038 [Largemouth bass virus]	93.3	45	3.21E-16	6
k141_94107_flag1_multi123.0177_len649	W2-1	649	UUY86263.1 hypothetical protein [Largemouth bass virus]	97.6	41	3.22E-16	372
k141_46797_flag1_multi4.8793_len870	W5-1	870	UUY86261.1 hypothetical protein [Largemouth bass virus]	100	46	3.32E-16	9
k141_86678_flag1_multi1.0000_len343	W1-1	343	WHU98721.1 MCP, partial [Hybrid snakehead ranavirus]	100	39	3.39E-16	0
k141_69031_flag1_multi11.6427_len849	W4-1	849	UVF58785.1 MAG: major capsid protein [Halichoeres melanurus ranavirus]	100	46	3.42E-16	30
k141_77870_flag1_multi2.5625_len781	W1-1	781	QYU76034.1 putative myristylated membrane protein, partial [Koi ranavirus]	100	46	3.44E-16	5
k141_64044_flag1_multi6.5225_len430	W4-1	430	QJE49073.1 putative p31K protein [Largemouth bass virus]	75.8	62	3.59E-16	4

k141_87942_flag1_multi4.1714_len1314	W1-1	1314	WAK75094.1 hypothetical protein [Mandarin fish ranavirus]	100	38	3.62E-16	22
k141_68702_flag1_multi5.2853_len1126	W5-1	1126	QJE49141.1 putative NTPase [Largemouth bass virus]	94	50	3.66E-16	19
k141_8868_flag0_multi18.2157_len1504	W1-1	1504	UUY86267.1 ribonucleotide reductase alpha subunit [Largemouth bass virus]	93.5	46	3.81E-16	67
k141_44496_flag1_multi2.9300_len798	W5-1	798	AYV88176.1 putative tumor necrosis factor receptor [Mandarin fish ranavirus]	100	46	3.90E-16	7
k141_43210_flag1_multi1.0000_len998	W5-1	998	UUY86197.1 hypothetical protein [Largemouth bass virus]	100	46	4.11E-16	27.94
k141_106802_flag1_multi2.9847_len926	W2-1	926	AYV88131.1 putative eIF-2 alpha-like protein [Mandarin fish ranavirus]	100	41	4.14E-16	13
k141_39063_flag0_multi52.4673_len860	W2-1	860	UUY86261.1 hypothetical protein [Largemouth bass virus]	100	46	4.24E-16	99
k141_75661_flag1_multi9.3790_len579	W4-1	579	UUY86258.1 hypothetical protein [Largemouth bass virus]	90.2	51	4.30E-16	14
k141_87876_flag1_multi4.0000_len616	W1-1	616	AFD96401.1 major capsid protein, partial [Largemouth bass virus]	97.4	39	4.35E-16	7
k141_38201_flag1_multi2.9198_len490	W5-1	490	UUY86258.1 hypothetical protein [Largemouth bass virus]	97.7	44	4.43E-16	3
k141_40314_flag1_multi5.9178_len1540	W5-1	1540	QJE49114.1 hypothetical protein LMBV_051 [Largemouth bass virus]	100	37	4.45E-16	25
k141_4680_flag1_multi3.9355_len1210	W6-1	1210	AIG51690.1 major capsid protein, partial [Koi ranavirus]	100	46	4.55E-16	14
k141_53383_flag0_multi1.0000_len403	W4-1	403	AIG51690.1 major capsid protein, partial [Koi ranavirus]	97.8	45	4.61E-16	6.09
k141_136490_flag0_multi1.0000_len548	W1-1	548	QJE49088.1 hypothetical protein LMBV_025 [Largemouth bass virus]	97.9	47	4.75E-16	3.06
k141_36075_flag0_multi87.6538_len2507	W5-1	2507	UUY86256.1 putative orf58-like protein [Largemouth bass virus]	97.9	47	4.88E-16	745
k141_161425_flag1_multi1.0000_len640	W5-1	640	AYV88120.1 putative p31K protein [Mandarin fish ranavirus]	100	39	4.95E-16	14.78
k141_104994_flag1_multi23.7164_len10848	W2-3	10848	QJE49112.1 hypothetical protein LMBV_049 [Largemouth bass virus]	97.5	40	5.03E-16	768
k141_38255_flag1_multi2.0000_len458	W6-1	458	AYV88118.1 hypothetical protein [Mandarin fish ranavirus]	94.4	36	5.15E-16	3
k141_75772_flag1_multi5.0000_len557	W4-1	557	WHA35533.1 putative DNA dependent RNA polymerase A subunit [Micropterus salmoides ranavirus]	72.2	54	5.40E-16	13
k141_24784_flag1_multi2.6675_len544	W5-1	544	UUY86193.1 hypothetical protein [Largemouth bass virus]	100	40	6.11E-16	2
k141_81114_flag0_multi2.7500_len365	W2-1	365	UUY86199.1 hypothetical protein [Largemouth bass virus]	100	34	6.17E-16	4.85
k141_66120_flag1_multi14.0000_len734	W1-1	734	QJE49149.1 putative immediate early protein ICP-46 [Largemouth bass virus]	100	39	6.18E-16	34
k141_81051_flag1_multi3.0000_len514	W6-1	514	QJE49112.1 hypothetical protein LMBV_049 [Largemouth bass virus]	88.1	42	6.18E-16	9
k141_164556_flag1_multi23.9760_len1183	W5-1	1183	UUY86269.1 hypothetical protein [Largemouth bass virus]	88.2	51	6.33E-16	338.56
k141_1926_flag1_multi4.0000_len589	W6-1	589	QYU76034.1 putative myristylated membrane protein, partial [Koi ranavirus]	93	43	6.41E-16	6
k141_23457_flag0_multi1.6759_len610	W1-1	610	QJE49097.1 hypothetical protein LMBV_034 [Largemouth bass virus]	100	37	6.51E-16	5
k141_74976_flag0_multi1.0000_len361	W5-1	361	QJE49103.1 hypothetical protein LMBV_040 [Largemouth bass virus]	87	46	6.59E-16	0
k141_66465_flag1_multi13.7521_len1347	W1-1	1347	XPZ21295.1 putative RNAseIII [Mandarin fish ranavirus]	100	47	6.60E-16	50
k141_168652_flag1_multi11.9914_len1426	W5-1	1426	WAK75107.1 hypothetical protein [Mandarin fish ranavirus]	100	38	7.66E-16	53
k141_34419_flag1_multi1.0000_len495	W5-1	495	AYV88154.1 putative helicase-like protein [Mandarin fish ranavirus]	88.6	44	7.77E-16	3
k141_143761_flag0_multi1.0000_len563	W5-1	563	UUY86197.1 hypothetical protein [Largemouth bass virus]	73.2	71	7.83E-16	6
k141_127950_flag1_multi1.8535_len414	W5-1	414	UVF58785.1 MAG: major capsid protein [Halichoeres melanurus ranavirus]	100	39	7.86E-16	1
k141_7341_flag1_multi4.0109_len508	W5-1	508	UUY86271.1 hypothetical protein [Largemouth bass virus]	100	37	7.98E-16	6
k141_13310_flag0_multi3.4399_len907	W4-1	907	QIZ30887.1 major capsid protein, partial [Largemouth bass virus]	95.1	41	8.26E-16	14
k141_100232_flag1_multi4.9078_len835	W5-1	835	QJE49101.1 hypothetical protein LMBV_038 [Largemouth bass virus]	97.7	44	8.26E-16	11
k141_57170_flag1_multi5.8940_len424	W4-1	424	UUY86229.1 putative DNA dependent RNA polymerase a subunit [Largemouth bass virus]	92.9	42	8.31E-16	5
k141_127803_flag1_multi3.9280_len683	W5-1	683	UUY86235.1 hypothetical protein [Largemouth bass virus]	97.7	44	8.37E-16	6
k141_24209_flag1_multi11.4000_len561	W4-2	561	UUY86266.1 hypothetical protein [Largemouth bass virus]	83.3	48	8.42E-16	30

k141_5784_flag0_multi3.0584_len603	W1-1	603	QJE49066.1 hypothetical protein LMBV_003 [Largemouth bass virus]	97.6	41	8.56E-16	6
k141_55999_flag1_multi3.0926_len670	W5-1	670	AYV88120.1 putative p31K protein [Mandarin fish ranavirus]	100	46	8.59E-16	6
k141_140227_flag1_multi1.0000_len330	W6-2	330	UUY86256.1 putative orf58-like protein [Largemouth bass virus]	100	41	8.90E-16	4
k141_46372_flag1_multi4.1533_len715	W5-1	715	QJE49096.1 putative D5 family NTPase/ATPase [Largemouth bass virus]	100	40	8.97E-16	6
k141_126455_flag0_multi1.0000_len281	W1-1	281	AYV88120.1 putative p31K protein [Mandarin fish ranavirus]	95	40	9.01E-16	0
k141_144289_flag1_multi1.0000_len455	W5-1	455	UUY86211.1 hypothetical protein [Largemouth bass virus]	90.7	43	9.11E-16	1
k141_75585_flag1_multi1.0000_len450	W5-1	450	UUY86197.1 hypothetical protein [Largemouth bass virus]	91.3	46	9.24E-16	1
k141_108207_flag1_multi5.8535_len926	W5-1	926	AYV88120.1 putative p31K protein [Mandarin fish ranavirus]	100	46	9.36E-16	12
k141_52472_flag1_multi2.0000_len488	W5-1	488	AYV88168.1 hypothetical protein [Mandarin fish ranavirus]	100	40	9.61E-16	2
k141_127690_flag0_multi4.1494_len837	W5-1	837	AYV88179.2 putative DNA dependent RNA polymerase II second largest subunit [Mandarin fish ranavirus]	83.3	54	9.73E-16	11
k141_125822_flag1_multi8.2604_len859	W5-1	859	AIG51690.1 major capsid protein, partial [Koi ranavirus]	97.9	47	9.91E-16	25
k141_115298_flag1_multi2.7186_len1136	W5-1	1136	UUY86261.1 hypothetical protein [Largemouth bass virus]	100	46	1.02E-15	14
k141_50361_flag1_multi20.4472_len2413	W1-1	2413	AYV88191.1 hypothetical protein [Mandarin fish ranavirus]	95.1	41	1.04E-15	147
k141_6834_flag1_multi2.6751_len1015	W6-2	1015	AYV88120.1 putative p31K protein [Mandarin fish ranavirus]	100	42	1.06E-15	7
k141_105571_flag1_multi8.1720_len1560	W5-1	1560	UUY86261.1 hypothetical protein [Largemouth bass virus]	100	46	1.10E-15	50
k141_6942_flag1_multi6.8635_len1767	W5-1	1767	UVF58790.1 MAG: myristylated membrane protein, partial [Halichoeres melanurus ranavirus]	88.9	54	1.12E-15	34
k141_122035_flag1_multi2.9197_len1212	W1-1	1212	AIG51690.1 major capsid protein, partial [Koi ranavirus]	100	46	1.13E-15	19
k141_33782_flag0_multi1.0000_len281	W1-1	281	QJE49100.1 hypothetical protein LMBV_037 [Largemouth bass virus]	100	39	1.14E-15	0
k141_54679_flag1_multi7.3305_len1300	W1-1	1300	UVF58785.1 MAG: major capsid protein [Halichoeres melanurus ranavirus]	100	46	1.17E-15	39
k141_66702_flag1_multi2.5219_len689	W2-3	689	UUY86241.1 hypothetical protein [Largemouth bass virus]	94.6	37	1.17E-15	6.09
k141_110257_flag1_multi4.0000_len497	W5-1	497	QJE49114.1 hypothetical protein LMBV_051 [Largemouth bass virus]	100	33	1.21E-15	11
k141_112428_flag1_multi10.0000_len495	W6-2	495	QJE49116.1 hypothetical protein LMBV_053 [Largemouth bass virus]	100	40	1.27E-15	9
k141_112_flag1_multi2.6594_len552	W5-1	552	WXI69548.1 hypothetical protein [Largemouth bass virus]	100	39	1.30E-15	4
k141_129064_flag0_multi15.5000_len207	W5-1	207	QYU76034.1 putative myristylated membrane protein, partial [Koi ranavirus]	100	41	1.30E-15	0
k141_17027_flag1_multi1.7265_len598	W1-1	598	UUY86238.1 hypothetical protein [Largemouth bass virus]	97.6	42	1.32E-15	3
k141_78995_flag1_multi4.0000_len1248	W5-1	1248	QJE49081.1 hypothetical protein LMBV_018 [Largemouth bass virus]	100	39	1.38E-15	13
k141_155921_flag1_multi3.0000_len442	W5-1	442	UUY86229.1 putative DNA dependent RNA polymerase a subunit [Largemouth bass virus]	90.9	44	1.39E-15	2
k141_41577_flag1_multi7.6785_len1012	W5-1	1012	AYV88120.1 putative p31K protein [Mandarin fish ranavirus]	100	46	1.42E-15	23
k141_138108_flag0_multi8.0000_len672	W5-1	672	WHA35678.1 hypothetical protein SCRaV_86R [Siniperca chuatsi ranavirus]	100	39	1.50E-15	6
k141_110057_flag0_multi26.0000_len393	W5-1	393	AYV88155.1 hypothetical protein [Mandarin fish ranavirus]	97.4	39	1.53E-15	12
k141_45913_flag1_multi1.9315_len477	W5-1	477	UUY86211.1 hypothetical protein [Largemouth bass virus]	100	46	1.56E-15	2
k141_180603_flag1_multi1.0000_len324	W2-3	324	QJE49090.1 hypothetical protein LMBV_027 [Largemouth bass virus]	81.1	53	1.60E-15	2
k141_55983_flag1_multi11.9138_len524	W4-1	524	CBW45581.1 hypothetical protein, partial [Doctor fish virus]	92.7	41	1.60E-15	30
k141_84771_flag1_multi5.0432_len604	W4-1	604	WAK75110.1 hypothetical protein [Mandarin fish ranavirus]	87.8	49	1.68E-15	36
k141_78658_flag1_multi11.6786_len897	W6-2	897	UUY86235.1 hypothetical protein [Largemouth bass virus]	100	43	1.76E-15	37
k141_48145_flag1_multi7.9842_len1220	W2-1	1220	UUY86199.1 hypothetical protein [Largemouth bass virus]	87.2	39	1.78E-15	41
k141_118315_flag0_multi147.0000_len313	W1-1	313	AYV88134.2 putative tyrosine kinase [Mandarin fish ranavirus]	67.3	52	1.79E-15	2

k141_157848_flag1_multi8.0000_len937	W2-3	937	AFD96401.1 major capsid protein, partial [Largemouth bass virus]	88.9	45	1.82E-15	24
k141_90551_flag0_multi31.0000_len591	W1-1	591	UVF58785.1 MAG: major capsid protein [Halichoeres melanurus ranavirus]	81.6	49	1.85E-15	22.14
k141_103137_flag1_multi8.0000_len2471	W5-1	2471	UUY86235.1 hypothetical protein [Largemouth bass virus]	100	39	1.87E-15	74
k141_99524_flag1_multi1.0000_len411	W5-1	411	AIG51690.1 major capsid protein, partial [Koi ranavirus]	100	39	1.91E-15	10
k141_92253_flag1_multi11.9085_len2906	W1-1	2906	UUY86269.1 hypothetical protein [Largemouth bass virus]	92.2	51	1.92E-15	117
k141_152238_flag1_multi3.4344_len850	W5-1	850	AYV88179.2 putative DNA dependent RNA polymerase II second largest subunit [Mandarin fish ranavirus]	97.6	42	1.93E-15	12
k141_130182_flag1_multi8.0000_len986	W1-1	986	UUY86211.1 hypothetical protein [Largemouth bass virus]	79.2	53	1.95E-15	17
k141_54460_flag1_multi2.9692_len660	W5-1	660	WEI28972.1 CTD-phosphotransferase [Largemouth bass virus]	100	39	1.96E-15	1
k141_136219_flag1_multi3.3630_len1097	W5-1	1097	WHA35534.1 hypothetical protein MSRaV_46R [Micropterus salmoides ranavirus]	78.7	47	2.04E-15	10
k141_22702_flag0_multi39.2678_len1403	W4-2	1403	UVF58785.1 MAG: major capsid protein [Halichoeres melanurus ranavirus]	97.9	47	2.06E-15	69
k141_163633_flag1_multi1.6573_len462	W5-1	462	UUY86267.1 ribonucleotide reductase alpha subunit [Largemouth bass virus]	92.5	40	2.07E-15	1
k141_70747_flag1_multi1.0000_len561	W5-1	561	WAK75112.1 putative LITAF PIG7 possible membrane associated motif in LPS-induced tumor necrosis factor alpha factor [Mandarin fish ranavirus]	97	33	2.11E-15	5
k141_129652_flag0_multi35.5711_len549	W1-1	549	WEI29006.1 putative 2-cysteine adaptor domain protein [Largemouth bass virus]	90.5	42	2.16E-15	16
k141_44485_flag1_multi1.0000_len359	W5-1	359	WAK75102.1 hypothetical protein [Mandarin fish ranavirus]	97.4	38	2.30E-15	1
k141_32197_flag1_multi8.5111_len910	W5-1	910	QJE49066.1 hypothetical protein LMBV_003 [Largemouth bass virus]	100	42	2.31E-15	22
k141_167803_flag1_multi18.7474_len1780	W5-1	1780	UUY86197.1 hypothetical protein [Largemouth bass virus]	100	46	2.36E-15	113
k141_10591_flag1_multi3.0000_len756	W5-1	756	WAK75112.1 putative LITAF PIG7 possible membrane associated motif in LPS-induced tumor necrosis factor alpha factor [Mandarin fish ranavirus]	97.2	36	2.36E-15	8
k141_146639_flag0_multi26.8824_len5004	W5-1	5004	WAK75075.1 hypothetical protein [Mandarin fish ranavirus]	97.3	37	2.37E-15	368
k141_43233_flag1_multi2.9547_len980	W5-1	980	UUY86197.1 hypothetical protein [Largemouth bass virus]	100	45	2.38E-15	10
k141_155617_flag1_multi17.8582_len4936	W5-1	4936	XRL22821.1 hypothetical protein [Siniperca chuatsi ranavirus]	100	46	2.45E-15	232
k141_181733_flag0_multi31.5222_len434	W2-3	434	QJE49116.1 hypothetical protein LMBV_053 [Largemouth bass virus]	100	39	2.63E-15	12
k141_89786_flag0_multi4.0331_len322	W5-1	322	QJE49153.1 hypothetical protein LMBV_004 [Largemouth bass virus]	92.3	39	2.66E-15	2
k141_146826_flag0_multi19.7231_len2709	W5-1	2709	UVF58785.1 MAG: major capsid protein [Halichoeres melanurus ranavirus]	100	46	2.67E-15	184
k141_84230_flag1_multi8.7401_len849	W4-1	849	XRL22790.1 hypothetical protein [Siniperca chuatsi ranavirus]	100	46	2.69E-15	20
k141_103216_flag1_multi3.6951_len1102	W5-1	1102	QJE49105.1 hypothetical protein LMBV_042 [Largemouth bass virus]	97.8	46	2.77E-15	19
k141_10876_flag1_multi12.5832_len760	W5-1	760	UUY86235.1 hypothetical protein [Largemouth bass virus]	97.6	42	2.86E-15	35
k141_32418_flag0_multi196.8737_len1028	W6-1	1028	QJE49101.1 hypothetical protein LMBV_038 [Largemouth bass virus]	86	50	2.91E-15	444
k141_5640_flag1_multi14.6767_len639	W1-1	639	UUY86261.1 hypothetical protein [Largemouth bass virus]	100	44	3.11E-15	52
k141_113678_flag1_multi27.7892_len3087	W1-1	3087	UUY86261.1 hypothetical protein [Largemouth bass virus]	100	47	3.18E-15	328
k141_28076_flag1_multi3.4578_len556	W1-1	556	WEI29006.1 putative 2-cysteine adaptor domain protein [Largemouth bass virus]	100	38	3.21E-15	3
k141_63035_flag0_multi21.1678_len576	W4-1	576	AAF64582.1 capsid protein, partial [Guppyfish iridovirus]	95.2	42	3.27E-15	17
k141_40059_flag1_multi11.7704_len1866	W5-1	1866	UUY86235.1 hypothetical protein [Largemouth bass virus]	97.8	45	3.28E-15	68
k141_9340_flag1_multi1.0000_len343	W5-1	343	AFD96401.1 major capsid protein, partial [Largemouth bass virus]	94.6	37	3.33E-15	0
k141_42036_flag1_multi3.7948_len1340	W1-1	1340	QIZ30887.1 major capsid protein, partial [Largemouth bass virus]	100	39	3.34E-15	14
k141_9435_flag0_multi1.0000_len378	W2-1	378	AIG51690.1 major capsid protein, partial [Koi ranavirus]	100	40	3.35E-15	0
k141_41886_flag1_multi2.3196_len554	W5-1	554	AYV88142.2 hypothetical protein [Mandarin fish ranavirus]	97.4	39	3.36E-15	2
k141_55668_flag1_multi3.0672_len1093	W5-1	1093	WAK75129.1 hypothetical protein [Mandarin fish ranavirus]	97.2	36	3.43E-15	11

k141_73199_flag1_multi24.3708_len586	W5-1	586	UUY86256.1 putative orf58-like protein [Largemouth bass virus]	72.9	59	3.45E-15	43
k141_86354_flag0_multi124.3179_len767	W5-1	767	UUY86261.1 hypothetical protein [Largemouth bass virus]	100	46	3.46E-15	225
k141_81185_flag1_multi2.8456_len536	W4-1	536	WEI29006.1 putative 2-cysteine adaptor domain protein [Largemouth bass virus]	100	39	3.55E-15	104.68
k141_114572_flag1_multi3.9189_len770	W5-1	770	AIG51690.1 major capsid protein, partial [Koi ranavirus]	100	44	3.66E-15	12
k141_166916_flag0_multi1.0000_len283	W5-1	283	UUY86256.1 putative orf58-like protein [Largemouth bass virus]	88.4	43	3.68E-15	1
k141_121140_flag1_multi13.6921_len1112	W5-1	1112	UUY86221.1 hypothetical protein [Largemouth bass virus]	95.9	49	3.71E-15	61
k141_156811_flag1_multi4.7617_len527	W5-1	527	UVF58790.1 MAG: myristylated membrane protein, partial [Halichoeres melanurus ranavirus]	100	46	3.80E-15	8
k141_116379_flag1_multi2.0000_len585	W5-1	585	UUY86235.1 hypothetical protein [Largemouth bass virus]	97.5	40	3.85E-15	3
k141_142285_flag0_multi2.2871_len545	W5-1	545	WEI29006.1 putative 2-cysteine adaptor domain protein [Largemouth bass virus]	97.5	40	3.88E-15	2
k141_102651_flag1_multi2.8614_len473	W4-1	473	AYV88120.1 putative p31K protein [Mandarin fish ranavirus]	95.7	46	4.07E-15	2
k141_114357_flag1_multi3.0000_len735	W1-1	735	UUY86192.1 putative myristylated membrane protein [Largemouth bass virus]	97.5	40	4.15E-15	5
k141_1439_flag1_multi4.9229_len1335	W5-1	1335	UVF58785.1 MAG: major capsid protein [Halichoeres melanurus ranavirus]	85.5	55	4.16E-15	20
k141_90880_flag1_multi1.8394_len527	W6-1	527	QJE49153.1 hypothetical protein LMBV_004 [Largemouth bass virus]	97.5	40	4.28E-15	0
k141_28945_flag1_multi69.4956_len825	W5-1	825	WAK75107.1 hypothetical protein [Mandarin fish ranavirus]	66.7	60	4.45E-15	168
k141_91450_flag1_multi2.0000_len683	W6-1	683	QJE49119.1 hypothetical protein LMBV_056 [Largemouth bass virus]	92.9	42	4.47E-15	3
k141_23621_flag1_multi4.0000_len534	W3-1	534	XRB52788.1 collagen-like protein [Largemouth bass virus]	100	119	4.68E-15	4
k141_69211_flag1_multi3.8623_len446	W4-1	446	QJE49137.1 hypothetical protein LMBV_074 [Largemouth bass virus]	100	45	4.91E-15	3
k141_94357_flag1_multi1.0000_len658	W5-1	658	UUY86261.1 hypothetical protein [Largemouth bass virus]	100	42	4.93E-15	4
k141_87463_flag1_multi20.0000_len5807	W5-1	5807	AYV88191.1 hypothetical protein [Mandarin fish ranavirus]	78.2	55	4.99E-15	382
k141_10514_flag1_multi27.3640_len2553	W1-1	2553	QJE49105.1 hypothetical protein LMBV_042 [Largemouth bass virus]	93.9	49	5.20E-15	193
k141_131396_flag1_multi2.3881_len543	W5-1	543	XRL22821.1 hypothetical protein [Siniperca chuatsi ranavirus]	97.3	37	5.26E-15	6
k141_28661_flag1_multi1.9482_len585	W1-1	585	AYV88211.1 hypothetical protein [Mandarin fish ranavirus]	76.9	52	5.28E-15	8
k141_6958_flag1_multi11.9226_len3696	W5-1	3696	UUY86235.1 hypothetical protein [Largemouth bass virus]	65.3	72	5.52E-15	135
k141_85767_flag1_multi25.2186_len992	W5-1	992	XRL22790.1 hypothetical protein [Siniperca chuatsi ranavirus]	97.6	41	5.64E-15	123
k141_122811_flag1_multi14.8862_len598	W5-1	598	WAK75112.1 putative LITAF PIG7 possible membrane associated motif in LPS-induced tumor necrosis factor alpha factor [Mandarin fish ranavirus]	100	36	5.68E-15	14
k141_7130_flag1_multi21.9464_len6019	W1-1	6019	QYU76034.1 putative myristylated membrane protein, partial [Koi ranavirus]	100	43	5.80E-15	421
k141_38405_flag1_multi4.7728_len546	W2-1	546	AYV88120.1 putative p31K protein [Mandarin fish ranavirus]	97.8	46	5.85E-15	4
k141_111796_flag1_multi10.8919_len955	W2-1	955	QJE49114.1 hypothetical protein LMBV_051 [Largemouth bass virus]	89.7	39	5.89E-15	31
k141_96171_flag1_multi7.9081_len1892	W1-1	1892	UVF58785.1 MAG: major capsid protein [Halichoeres melanurus ranavirus]	100	46	5.99E-15	42
k141_145880_flag1_multi2.0000_len456	W5-1	456	UUY86211.1 hypothetical protein [Largemouth bass virus]	94.9	39	6.17E-15	3
k141_46505_flag1_multi10.7105_len521	W1-1	521	UVF58785.1 MAG: major capsid protein [Halichoeres melanurus ranavirus]	100	43	6.26E-15	94.01
k141_5503_flag1_multi1.0000_len546	W5-1	546	AYV88125.2 hypothetical protein [Mandarin fish ranavirus]	74	50	6.52E-15	1
k141_62572_flag1_multi4.0000_len535	W5-1	535	AIG51690.1 major capsid protein, partial [Koi ranavirus]	100	39	6.56E-15	10
k141_148344_flag0_multi7.0000_len356	W5-1	356	AIG51690.1 major capsid protein, partial [Koi ranavirus]	95	40	6.90E-15	2
k141_148208_flag1_multi3.4785_len559	W5-1	559	QJE49105.1 hypothetical protein LMBV_042 [Largemouth bass virus]	97.8	46	7.07E-15	2
k141_30055_flag1_multi18.7668_len990	W5-1	990	AYV88191.1 hypothetical protein [Mandarin fish ranavirus]	100	35	7.32E-15	59
k141_80901_flag1_multi1.0000_len723	W5-1	723	WAK75138.1 hypothetical protein [Mandarin fish ranavirus]	79.6	49	7.46E-15	7
k141_101851_flag1_multi13.0000_len1861	W5-1	1861	QIZ30887.1 major capsid protein, partial [Largemouth bass virus]	100	38	7.57E-15	121

k141_126365_flag1_multi10.0000_len1303	W1-1	1303	QJE49107.1 hypothetical protein LMBV_044 [Largemouth bass virus]	95.1	41	7.67E-15	32
k141_102759_flag1_multi19.2896_len2544	W4-1	2544	UUY86197.1 hypothetical protein [Largemouth bass virus]	100	46	7.72E-15	195
k141_87333_flag1_multi8.7875_len1195	W4-1	1195	UUY86228.1 hypothetical protein [Largemouth bass virus]	95.7	46	7.76E-15	30
k141_35098_flag1_multi16.3749_len1240	W4-1	1240	AYV88120.1 putative p31K protein [Mandarin fish ranavirus]	100	45	8.32E-15	43
k141_126806_flag1_multi1.0000_len508	W5-1	508	AYV88191.1 hypothetical protein [Mandarin fish ranavirus]	97.1	35	8.39E-15	2
k141_62760_flag0_multi4.7784_len714	W2-1	714	QJE49077.1 hypothetical protein LMBV_014 [Largemouth bass virus]	97.3	37	9.01E-15	11
k141_161689_flag1_multi4.0000_len576	W5-1	576	UUY86258.1 hypothetical protein [Largemouth bass virus]	100	39	9.08E-15	5
k141_134357_flag1_multi2.5690_len800	W5-1	800	AYV88211.1 hypothetical protein [Mandarin fish ranavirus]	78	59	9.09E-15	7
k141_17857_flag1_multi9.7160_len870	W5-1	870	UVF58790.1 MAG: myristylated membrane protein, partial [Halichoeres melanurus ranavirus]	95.8	48	9.36E-15	22
k141_98350_flag1_multi1.0000_len328	W5-1	328	WAK75079.1 hypothetical protein [Mandarin fish ranavirus]	88.9	36	9.36E-15	1
k141_31954_flag1_multi16.0952_len939	W4-1	939	XPZ21261.1 hypothetical protein MRVORF002 [Mandarin fish ranavirus]	95.3	43	9.57E-15	87
k141_58459_flag0_multi1.0000_len372	W2-1	372	AYV88120.1 putative p31K protein [Mandarin fish ranavirus]	86	43	1.01E-14	3
k141_90533_flag0_multi24.0000_len542	W5-1	542	UUY86235.1 hypothetical protein [Largemouth bass virus]	100	39	1.01E-14	12
k141_43351_flag0_multi5.6113_len424	W2-1	424	AYV88191.1 hypothetical protein [Mandarin fish ranavirus]	94.7	38	1.02E-14	0
k141_95490_flag0_multi8.8854_len1467	W5-1	1467	UVF58785.1 MAG: major capsid protein [Halichoeres melanurus ranavirus]	86.3	51	1.02E-14	36.03
k141_28993_flag1_multi3.9434_len1465	W1-1	1465	QJE49090.1 hypothetical protein LMBV_027 [Largemouth bass virus]	86.3	51	1.04E-14	25
k141_3830_flag0_multi25.9719_len1491	W1-1	1491	WHA35503.1 putative myristylated membrane protein [Micropterus salmoides ranavirus]	100	38	1.15E-14	116
k141_130576_flag1_multi23.0000_len4857	W5-1	4857	QIZ30887.1 major capsid protein, partial [Largemouth bass virus]	93.2	44	1.15E-14	441
k141_166100_flag1_multi0.9947_len516	W5-1	516	AYV88120.1 putative p31K protein [Mandarin fish ranavirus]	100	38	1.19E-14	0
k141_122520_flag1_multi7.0647_len3943	W1-1	3943	AYV88143.1 hypothetical protein [Mandarin fish ranavirus]	97.6	42	1.23E-14	430.45
k141_56647_flag0_multi1.0000_len447	W5-1	447	WEI28966.1 hypothetical protein [Largemouth bass virus]	97.2	36	1.23E-14	9.37
k141_6468_flag1_multi7.9744_len960	W4-1	960	QJE49137.1 hypothetical protein LMBV_074 [Largemouth bass virus]	100	39	1.31E-14	19
k141_79280_flag0_multi1.0000_len258	W2-1	258	UUY86257.1 hypothetical protein [Largemouth bass virus]	84.4	45	1.33E-14	1088.07
k141_19841_flag1_multi3.0000_len575	W5-1	575	WAK75079.1 hypothetical protein [Mandarin fish ranavirus]	88.9	36	1.37E-14	3
k141_135122_flag0_multi69.6768_len1357	W1-1	1357	UVF58785.1 MAG: major capsid protein [Halichoeres melanurus ranavirus]	100	46	1.43E-14	327
k141_564_flag0_multi12.7120_len1273	W4-1	1273	AAF64582.1 capsid protein, partial [Guppyfish iridovirus]	74.6	59	1.46E-14	44
k141_7074_flag1_multi4.4321_len583	W1-1	583	AYV88130.2 putative ATPase-dependent protease [Mandarin fish ranavirus]	67.8	59	1.51E-14	6
k141_56386_flag0_multi1.0000_len307	W5-1	307	WEI29006.1 putative 2-cysteine adaptor domain protein [Largemouth bass virus]	100	37	1.51E-14	0
k141_91240_flag0_multi1.0000_len366	W2-1	366	AYV88191.1 hypothetical protein [Mandarin fish ranavirus]	97.3	37	1.55E-14	5
k141_78051_flag1_multi11.5831_len1242	W5-1	1242	AYV88120.1 putative p31K protein [Mandarin fish ranavirus]	95.1	41	1.55E-14	50
k141_31994_flag1_multi3.0024_len550	W5-1	550	XRB52763.1 hypothetical protein LMBV_6 [Largemouth bass virus]	55.6	72	1.65E-14	1
k141_105740_flag1_multi3.9326_len809	W1-1	809	AYV88120.1 putative p31K protein [Mandarin fish ranavirus]	100	44	1.66E-14	12
k141_30777_flag1_multi3.0000_len722	W5-1	722	AIG51690.1 major capsid protein, partial [Koi ranavirus]	100	39	1.71E-14	3
k141_96676_flag1_multi1.0000_len562	W5-1	562	QIZ30887.1 major capsid protein, partial [Largemouth bass virus]	100	36	1.73E-14	5
k141_139420_flag1_multi3.5446_len477	W5-1	477	QYU76034.1 putative myristylated membrane protein, partial [Koi ranavirus]	94.9	39	1.74E-14	8
k141_114329_flag1_multi7.2585_len965	W2-3	965	XRB52768.1 P31K protein, partial [Largemouth bass virus]	84.4	45	1.76E-14	19
k141_48765_flag1_multi7.4516_len2105	W5-1	2105	QJE49137.1 hypothetical protein LMBV_074 [Largemouth bass virus]	95.3	43	1.79E-14	53
k141_104557_flag1_multi3.0000_len473	W1-1	473	AIG51690.1 major capsid protein, partial [Koi ranavirus]	100	38	1.80E-14	3

k141_7929_flag1_multi1.0000_len619	W5-1	619	WAK75112.1 putative LITAF PIG7 possible membrane associated motif in LPS-induced tumor necrosis factor alpha factor [Mandarin fish ranavirus]	100	34	1.87E-14	13
k141_125133_flag1_multi1.0000_len365	W1-1	365	AIG51690.1 major capsid protein, partial [Koi ranavirus]	90	40	2.01E-14	0
k141_84644_flag1_multi7.5400_len1328	W2-1	1328	QJE49081.1 hypothetical protein LMBV_018 [Largemouth bass virus]	97.4	38	2.05E-14	31
k141_139099_flag1_multi89.8629_len15001	W5-1	15001	XRL22821.1 hypothetical protein [Siniperca chuatsi ranavirus]	100	46	2.05E-14	4634.99
k141_73739_flag0_multi1.0000_len391	W2-1	391	QJE49149.1 putative immediate early protein ICP-46 [Largemouth bass virus]	88.6	44	2.07E-14	0
k141_161790_flag0_multi20.3767_len510	W5-1	510	UUY86192.1 putative myristylated membrane protein [Largemouth bass virus]	82.9	41	2.09E-14	5
k141_93213_flag1_multi1.0000_len447	W5-1	447	QJE49153.1 hypothetical protein LMBV_004 [Largemouth bass virus]	97.4	38	2.12E-14	16
k141_48155_flag1_multi2.9276_len362	W4-3	362	XRL22821.1 hypothetical protein [Siniperca chuatsi ranavirus]	100	35	2.18E-14	2
k141_95393_flag1_multi3.8473_len593	W2-3	593	UUY86235.1 hypothetical protein [Largemouth bass virus]	100	38	2.21E-14	6
k141_117862_flag1_multi9.9451_len1617	W1-1	1617	WXI69525.1 caspase recruitment domain protein [Largemouth bass virus]	97.6	42	2.22E-14	58
k141_76232_flag1_multi2.8472_len704	W1-1	704	AYV88134.2 putative tyrosine kinase [Mandarin fish ranavirus]	80.4	46	2.30E-14	4
k141_65261_flag1_multi1.0000_len385	W2-1	385	QJE49125.1 hypothetical protein LMBV_062 [Largemouth bass virus]	94.7	38	2.39E-14	0
k141_151889_flag1_multi2.8060_len543	W5-1	543	WEI29006.1 putative 2-cysteine adaptor domain protein [Largemouth bass virus]	97.3	37	2.39E-14	16
k141_91173_flag0_multi1.0000_len453	W5-1	453	WAK75112.1 putative LITAF PIG7 possible membrane associated motif in LPS-induced tumor necrosis factor alpha factor [Mandarin fish ranavirus]	94.1	34	2.43E-14	0
k141_138955_flag1_multi5.0000_len653	W5-1	653	AYV88211.1 hypothetical protein [Mandarin fish ranavirus]	92.9	42	2.44E-14	9
k141_66705_flag1_multi4.0000_len606	W5-1	606	UUY86235.1 hypothetical protein [Largemouth bass virus]	90.5	42	2.45E-14	8
k141_63476_flag0_multi4.0000_len524	W1-1	524	AYV88120.1 putative p31K protein [Mandarin fish ranavirus]	97.4	39	2.52E-14	7
k141_75765_flag1_multi8.3839_len925	W5-1	925	WAK75093.1 hypothetical protein [Mandarin fish ranavirus]	83	47	2.69E-14	16
k141_2597_flag1_multi4.8595_len568	W4-1	568	AYV88120.1 putative p31K protein [Mandarin fish ranavirus]	94.7	38	2.73E-14	7
k141_61609_flag1_multi10.9160_len2283	W5-1	2283	QJE49068.1 hypothetical protein LMBV_005 [Largemouth bass virus]	69.8	63	2.74E-14	66
k141_77574_flag1_multi3.3590_len829	W1-1	829	QJE49149.1 putative immediate early protein ICP-46 [Largemouth bass virus]	97.4	38	2.76E-14	11
k141_46851_flag1_multi2.1571_len644	W5-1	644	WEI28972.1 CTD-phosphotransferase [Largemouth bass virus]	76.4	55	2.77E-14	9
k141_165999_flag1_multi1.0000_len323	W5-1	323	XRB52831.1 hypothetical protein LMBV_74 [Largemouth bass virus]	97.4	38	2.81E-14	5
k141_88214_flag1_multi4.0000_len699	W5-1	699	WHU98721.1 MCP, partial [Hybrid snakehead ranavirus]	100	38	2.82E-14	4
k141_34378_flag0_multi2.1779_len304	W1-1	304	ACT85833.1 major capsid protein, partial [Frog virus 3]	82.1	39	2.84E-14	0
k141_166452_flag1_multi2.7788_len349	W5-1	349	QJE49114.1 hypothetical protein LMBV_051 [Largemouth bass virus]	100	31	2.93E-14	3
k141_146467_flag1_multi1.0000_len557	W5-1	557	XRB52842.1 transcription factor TFIIB cyclin-like domain-containing protein, partial [Largemouth bass virus]	80	50	3.03E-14	0
k141_120443_flag1_multi2.5516_len538	W1-1	538	UUY86236.1 hypothetical protein [Largemouth bass virus]	94.6	37	3.11E-14	7
k141_21034_flag1_multi10.0000_len846	W4-1	846	WAK75102.1 hypothetical protein [Mandarin fish ranavirus]	95	40	3.15E-14	25
k141_72275_flag1_multi5.0000_len826	W1-1	826	QJE49105.1 hypothetical protein LMBV_042 [Largemouth bass virus]	100	39	3.17E-14	10
k141_37438_flag1_multi9.0000_len808	W4-1	808	AYV88211.1 hypothetical protein [Mandarin fish ranavirus]	92.5	40	3.17E-14	25
k141_76227_flag1_multi1.0000_len483	W1-1	483	UUY86241.1 hypothetical protein [Largemouth bass virus]	97	33	3.21E-14	1
k141_109368_flag1_multi5.2391_len601	W5-1	601	XRL22790.1 hypothetical protein [Siniperca chuatsi ranavirus]	97.7	43	3.21E-14	14
k141_771_flag0_multi1.0000_len518	W5-1	518	UUY86267.1 ribonucleotide reductase alpha subunit [Largemouth bass virus]	75	52	3.23E-14	0
k141_92596_flag1_multi3.5210_len617	W4-1	617	UUY86258.1 hypothetical protein [Largemouth bass virus]	92.3	39	3.24E-14	9
k141_10319_flag1_multi3.0000_len638	W5-1	638	WAK75072.1 hypothetical protein [Mandarin fish ranavirus]	90.7	43	3.36E-14	4
k141_21598_flag1_multi25.6235_len1602	W4-1	1602	AYV88176.1 putative tumor necrosis factor receptor [Mandarin fish ranavirus]	97.4	38	3.39E-14	131

k141_154422_flag0_multi1.0000_len458	W5-1	458	QJE49086.1 hypothetical protein LMBV_023 [Largemouth bass virus]	87.2	39	3.52E-14	3
k141_15707_flag1_multi2.9742_len1927	W6-1	1927	WAK75138.1 hypothetical protein [Mandarin fish ranavirus]	80.9	47	3.92E-14	52
k141_29951_flag0_multi3.4543_len480	W5-1	480	QJE49098.1 hypothetical protein LMBV_035 [Largemouth bass virus]	91.7	36	3.94E-14	9
k141_38722_flag1_multi16.6624_len4454	W5-1	4454	WAK75112.1 putative LITAF PIG7 possible membrane associated motif in LPS-induced tumor necrosis factor alpha factor [Mandarin fish ranavirus]	64.1	64	3.98E-14	286
k141_22876_flag1_multi9.0000_len2232	W5-1	2232	WAK75107.1 hypothetical protein [Mandarin fish ranavirus]	100	34	4.00E-14	60
k141_33978_flag1_multi6.9684_len711	W1-1	711	ALR73092.1 major capsid protein, partial [Box turtle ranavirus]	86	43	4.07E-14	13
k141_31057_flag0_multi67.6227_len520	W1-1	520	UUY86228.1 hypothetical protein [Largemouth bass virus]	100	39	4.19E-14	250
k141_113931_flag1_multi1.0000_len346	W5-1	346	UUY86258.1 hypothetical protein [Largemouth bass virus]	94.6	37	4.40E-14	0
k141_52130_flag1_multi4.0000_len488	W5-1	488	QJE49104.1 hypothetical protein LMBV_041 [Largemouth bass virus]	100	35	4.55E-14	10
k141_124547_flag1_multi1.0000_len829	W5-1	829	AYV88118.1 hypothetical protein [Mandarin fish ranavirus]	94.3	35	4.68E-14	39
k141_146850_flag1_multi16.8719_len4639	W5-1	4639	QIZ30887.1 major capsid protein, partial [Largemouth bass virus]	100	38	5.15E-14	328
k141_4542_flag1_multi2.9316_len1749	W5-1	1749	XRL22790.1 hypothetical protein [Siniperca chuatsi ranavirus]	97.8	46	5.24E-14	43.21
k141_40804_flag1_multi1.0000_len365	W5-1	365	AYV88210.2 putative proliferating cell nuclear antigen [Mandarin fish ranavirus]	88.4	43	5.32E-14	0
k141_71268_flag0_multi1.0000_len319	W5-1	319	UVF58785.1 MAG: major capsid protein [Halichoeres melanurus ranavirus]	97.6	42	5.34E-14	0
k141_64333_flag0_multi3.0000_len463	W5-1	463	UUY86256.1 putative orf58-like protein [Largemouth bass virus]	100	37	5.45E-14	1
k141_30423_flag1_multi7.0000_len729	W2-1	729	UVF58787.1 MAG: DNA-dependent RNA polymerase largest subunit, partial [Halichoeres melanurus ranavirus]	100	38	5.51E-14	15
k141_145426_flag1_multi1.9125_len404	W5-1	404	QYU76036.1 helicase-like protein, partial [Koi ranavirus]	94.6	37	5.56E-14	3
k141_6088_flag1_multi5.2128_len827	W4-1	827	QJE49080.1 hypothetical protein LMBV_017 [Largemouth bass virus]	91.9	37	5.59E-14	8
k141_153919_flag1_multi4.0000_len1095	W5-1	1095	QJE49203.1 hypothetical protein LMBV_054 [Largemouth bass virus]	97.4	38	5.69E-14	11
k141_119077_flag1_multi1.0000_len318	W5-1	318	AYV88211.1 hypothetical protein [Mandarin fish ranavirus]	100	39	5.73E-14	0
k141_148074_flag1_multi4.9582_len811	W5-1	811	AYV88191.1 hypothetical protein [Mandarin fish ranavirus]	79.6	49	5.94E-14	9
k141_116884_flag1_multi4.0000_len633	W1-1	633	QJE49102.1 putative DNA dependent RNA polymerase a subunit [Largemouth bass virus]	100	39	5.95E-14	2
k141_22012_flag1_multi24.2107_len1133	W1-1	1133	WHA35533.1 putative DNA dependent RNA polymerase A subunit [Micropterus salmoides ranavirus]	100	44	5.98E-14	85
k141_37158_flag1_multi2.9707_len550	W6-3	550	QJE49129.1 putative orf58-like protein [Largemouth bass virus]	70.9	182	6.05E-14	13
k141_1559_flag1_multi4.0000_len485	W5-1	485	UUY86235.1 hypothetical protein [Largemouth bass virus]	100	38	6.22E-14	7
k141_84241_flag1_multi2.0000_len494	W5-1	494	UUY86258.1 hypothetical protein [Largemouth bass virus]	100	36	6.43E-14	1
k141_155551_flag1_multi4.0000_len439	W5-1	439	QJE49105.1 hypothetical protein LMBV_042 [Largemouth bass virus]	95.2	42	6.46E-14	4
k141_88175_flag1_multi3.9704_len445	W4-1	445	UVF58785.1 MAG: major capsid protein [Halichoeres melanurus ranavirus]	74.1	58	6.62E-14	1
k141_60655_flag1_multi22.0000_len723	W5-1	723	AYV88191.1 hypothetical protein [Mandarin fish ranavirus]	95	40	6.78E-14	62
k141_56292_flag1_multi7.9757_len800	W5-1	800	QJE49137.1 hypothetical protein LMBV_074 [Largemouth bass virus]	100	38	6.80E-14	11
k141_85368_flag1_multi11.9602_len694	W4-1	694	QJE49149.1 putative immediate early protein ICP-46 [Largemouth bass virus]	64.3	70	6.95E-14	19
k141_127507_flag1_multi1.8324_len493	W5-1	493	AYV88186.1 hypothetical protein [Mandarin fish ranavirus]	100	35	7.48E-14	6
k141_10945_flag0_multi97.4330_len238	W5-1	238	QJE49065.1 hypothetical protein LMBV_002 [Largemouth bass virus]	100	33	7.72E-14	1
k141_19030_flag1_multi1.0000_len404	W5-1	404	UUY86241.1 hypothetical protein [Largemouth bass virus]	97	33	7.73E-14	6
k141_103611_flag1_multi17.8112_len8604	W4-1	8604	UUY86192.1 putative myristylated membrane protein [Largemouth bass virus]	97.4	38	8.09E-14	537

k141_168708_flag1_multi1.6146_len634	W5-1	634	WHA35533.1 putative DNA dependent RNA polymerase A subunit [Micropterus salmoides ranavirus]	86	43	8.30E-14	3
k141_54480_flag1_multi1.4397_len605	W5-1	605	WAK75107.1 hypothetical protein [Mandarin fish ranavirus]	100	32	8.42E-14	3
k141_25225_flag1_multi1.0000_len596	W2-2	596	UUY86235.1 hypothetical protein [Largemouth bass virus]	57.1	84	8.62E-14	2
k141_136392_flag1_multi2.3985_len550	W5-1	550	UUY86269.1 hypothetical protein [Largemouth bass virus]	97.4	39	9.16E-14	5
k141_132493_flag1_multi3.0000_len428	W5-3	428	AYV88122.1 putative 3-beta-hydroxy-delta-5-C27 steroid oxidoreductase-like protein [Mandarin fish ranavirus]	97.1	34	9.36E-14	4
k141_32213_flag1_multi2.3896_len390	W1-1	390	AYV88183.1 hypothetical protein [Mandarin fish ranavirus]	100	33	9.62E-14	1
k141_51468_flag1_multi4.0000_len655	W4-1	655	QJE49114.1 hypothetical protein LMBV_051 [Largemouth bass virus]	52.5	80	9.68E-14	17
k141_65254_flag1_multi7.5257_len1056	W2-1	1056	AYV88179.2 putative DNA dependent RNA polymerase II second largest subunit [Mandarin fish ranavirus]	87.2	47	9.70E-14	30
k141_105890_flag0_multi7.9299_len5734	W5-1	5734	QYU76034.1 putative myristylated membrane protein, partial [Koi ranavirus]	88.9	45	9.75E-14	144
k141_144546_flag1_multi5.0000_len2837	W5-1	2837	QYU76031.1 neurofilament triplet H1-like protein, partial [Koi ranavirus]	100	39	9.92E-14	41
k141_142796_flag1_multi1.0000_len327	W5-1	327	WAK75112.1 putative LITAF PIG7 possible membrane associated motif in LPS-induced tumor necrosis factor alpha factor [Mandarin fish ranavirus]	100	31	1.02E-13	3
k141_256390_flag1_multi1.0000_len669	W5-3	669	AIG51690.1 major capsid protein, partial [Koi ranavirus]	100	38	1.03E-13	8
k141_181161_flag1_multi4.0000_len713	W2-3	713	AIG51690.1 major capsid protein, partial [Koi ranavirus]	65.2	66	1.06E-13	9
k141_99680_flag1_multi2.0000_len645	W5-1	645	AYV88118.1 hypothetical protein [Mandarin fish ranavirus]	97.1	35	1.13E-13	3
k141_37910_flag1_multi3.0000_len894	W5-1	894	WEI29006.1 putative 2-cysteine adaptor domain protein [Largemouth bass virus]	100	37	1.15E-13	10
k141_38857_flag1_multi7.1707_len639	W4-1	639	UVF58785.1 MAG: major capsid protein [Halichoeres melanurus ranavirus]	100	39	1.16E-13	18
k141_26982_flag1_multi21.5900_len1319	W1-1	1319	AYV88120.1 putative p31K protein [Mandarin fish ranavirus]	95.7	46	1.17E-13	82
k141_15110_flag1_multi7.2468_len1405	W5-1	1405	UVF58785.1 MAG: major capsid protein [Halichoeres melanurus ranavirus]	97.8	46	1.21E-13	34
k141_20921_flag1_multi8.5950_len583	W5-1	583	QJE49109.1 hypothetical protein LMBV_046 [Largemouth bass virus]	89.2	37	1.26E-13	19
k141_79780_flag1_multi7.5296_len902	W5-1	902	UUY86261.1 hypothetical protein [Largemouth bass virus]	95.8	48	1.26E-13	29
k141_123331_flag1_multi4.2519_len788	W5-1	788	UUY86235.1 hypothetical protein [Largemouth bass virus]	100	40	1.27E-13	9
k141_96756_flag0_multi15.7823_len1657	W5-1	1657	QJE49104.1 hypothetical protein LMBV_041 [Largemouth bass virus]	100	39	1.45E-13	40
k141_37699_flag1_multi4.8442_len603	W1-1	603	UUY86229.1 putative DNA dependent RNA polymerase a subunit [Largemouth bass virus]	100	38	1.55E-13	13
k141_86939_flag1_multi13.7693_len995	W5-1	995	AIG51690.1 major capsid protein, partial [Koi ranavirus]	100	42	1.59E-13	48
k141_47503_flag1_multi5.0000_len1031	W5-1	1031	AYV88191.1 hypothetical protein [Mandarin fish ranavirus]	97.2	36	1.59E-13	13
k141_42311_flag1_multi1.0000_len382	W5-1	382	AYV88120.1 putative p31K protein [Mandarin fish ranavirus]	79.6	49	1.61E-13	0
k141_65913_flag1_multi4.9501_len1003	W5-1	1003	QJE49079.1 hypothetical protein LMBV_016 [Largemouth bass virus]	97.3	37	1.64E-13	28
k141_63350_flag1_multi10.4384_len871	W1-1	871	QJE49146.1 putative thiol oxidoreductase [Largemouth bass virus]	75.5	49	1.67E-13	10
k141_74649_flag1_multi6.1006_len1622	W5-1	1622	UUY86256.1 putative orf58-like protein [Largemouth bass virus]	100	39	1.74E-13	49
k141_978_flag0_multi3.9216_len830	W5-1	830	QJE49149.1 putative immediate early protein ICP-46 [Largemouth bass virus]	88.6	44	1.78E-13	7
k141_153818_flag1_multi1.0000_len467	W2-3	467	AAK54493.1 RSIV DNA polymerase-like protein, partial [Regina ranavirus]	61.4	57	1.83E-13	3
k141_35512_flag1_multi9.9201_len704	W5-1	704	UUY86269.1 hypothetical protein [Largemouth bass virus]	97.5	40	1.93E-13	12
k141_166952_flag1_multi1.0000_len775	W5-1	775	WHA35533.1 putative DNA dependent RNA polymerase A subunit [Micropterus salmoides ranavirus]	97.6	42	1.93E-13	14
k141_141808_flag1_multi1.0000_len914	W5-1	914	AIG51690.1 major capsid protein, partial [Koi ranavirus]	100	38	1.98E-13	12

k141_163940_flag0_multi4.8870_len318	W5-1	318	AYV88211.1 hypothetical protein [Mandarin fish ranavirus]	83	47	1.99E-13	68
k141_25768_flag1_multi1.0000_len462	W5-1	462	AYV88120.1 putative p31K protein [Mandarin fish ranavirus]	53.5	86	2.00E-13	3
k141_17680_flag1_multi8.0000_len632	W4-1	632	UUY86229.1 putative DNA dependent RNA polymerase a subunit [Largemouth bass virus]	100	39	2.04E-13	18
k141_97999_flag1_multi1.0000_len374	W4-1	374	QJE49155.1 hypothetical protein LMBV_006 [Largemouth bass virus]	97.4	39	2.12E-13	3
k141_63419_flag1_multi4.0000_len564	W1-1	564	AIG51690.1 major capsid protein, partial [Koi ranavirus]	97.4	38	2.14E-13	2
k141_180133_flag1_multi4.0000_len853	W2-3	853	QJE49112.1 hypothetical protein LMBV_049 [Largemouth bass virus]	87.5	40	2.19E-13	13
k141_31333_flag0_multi1.4741_len411	W5-1	411	AYV88120.1 putative p31K protein [Mandarin fish ranavirus]	85.4	48	2.23E-13	1
k141_77917_flag1_multi4.8535_len769	W5-1	769	AYV88149.2 hypothetical protein [Mandarin fish ranavirus]	100	35	2.35E-13	8
k141_161261_flag1_multi3.0000_len595	W5-1	595	AAC79876.1 viral core protein, partial [Labroides dimidatus ranavirus]	100	33	2.38E-13	2
k141_108628_flag1_multi8.7738_len698	W5-1	698	QJE49215.1 putative orf58-like protein [Largemouth bass virus]	97.4	38	2.49E-13	18
k141_35417_flag1_multi3.0000_len406	W5-1	406	UUY86192.1 putative myristylated membrane protein [Largemouth bass virus]	100	35	2.55E-13	5
k141_112524_flag0_multi7.6980_len651	W6-2	651	QJE49092.1 hypothetical protein LMBV_029 [Largemouth bass virus]	100	37	2.59E-13	8
k141_15078_flag0_multi1.8668_len877	W5-1	877	WEI28966.1 hypothetical protein [Largemouth bass virus]	97.2	36	2.64E-13	10
k141_145801_flag1_multi7.0000_len435	W5-3	435	QJE49092.1 hypothetical protein LMBV_029 [Largemouth bass virus]	100	36	2.71E-13	7
k141_102879_flag1_multi52.8629_len1279	W4-1	1279	AYV88120.1 putative p31K protein [Mandarin fish ranavirus]	97.4	39	2.72E-13	188
k141_128321_flag1_multi1.0000_len311	W5-1	311	WAK75072.1 hypothetical protein [Mandarin fish ranavirus]	90	40	2.72E-13	3
k141_138617_flag0_multi76.4762_len477	W5-1	477	XPZ21261.1 hypothetical protein MRVORF002 [Mandarin fish ranavirus]	100	40	2.72E-13	95
k141_121774_flag0_multi29.9036_len1687	W5-1	1687	AYV88211.1 hypothetical protein [Mandarin fish ranavirus]	86.3	51	3.04E-13	83
k141_16848_flag1_multi3.4282_len858	W5-1	858	UVF58790.1 MAG: myristylated membrane protein, partial [Halichoeres melanurus ranavirus]	97.9	47	3.25E-13	15
k141_73771_flag1_multi8.0000_len644	W5-1	644	QJE49100.1 hypothetical protein LMBV_037 [Largemouth bass virus]	90.5	42	3.30E-13	14
k141_71206_flag1_multi21.0000_len553	W2-3	553	QJE49090.1 hypothetical protein LMBV_027 [Largemouth bass virus]	92.5	40	3.69E-13	23
k141_17430_flag0_multi3.6190_len582	W5-1	582	UUY86197.1 hypothetical protein [Largemouth bass virus]	83.9	56	3.73E-13	11
k141_38925_flag1_multi14.0000_len415	W5-1	415	UUY86199.1 hypothetical protein [Largemouth bass virus]	100	31	3.78E-13	8
k141_54706_flag1_multi2.0000_len311	W5-1	311	UUY86235.1 hypothetical protein [Largemouth bass virus]	97.1	35	3.91E-13	2
k141_129561_flag1_multi2.9529_len1117	W5-1	1117	XRL22790.1 hypothetical protein [Siniperca chuatsi ranavirus]	97.4	39	4.01E-13	114
k141_91272_flag1_multi2.0000_len480	W5-1	480	XRB52842.1 transcription factor TFIIB cyclin-like domain-containing protein, partial [Largemouth bass virus]	97.3	37	4.02E-13	3
k141_42204_flag1_multi11.0000_len1899	W5-1	1899	AIG51690.1 major capsid protein, partial [Koi ranavirus]	100	39	4.15E-13	77
k141_40670_flag1_multi5.7067_len516	W2-1	516	QYU76034.1 putative myristylated membrane protein, partial [Koi ranavirus]	88.1	42	4.42E-13	0
k141_15043_flag1_multi2.0000_len394	W5-1	394	WHU98721.1 MCP, partial [Hybrid snakehead ranavirus]	100	37	4.53E-13	0
k141_30522_flag0_multi1.0000_len260	W2-3	260	AYV88122.1 putative 3-beta-hydroxy-delta-5-C27 steroid oxidoreductase-like protein [Mandarin fish ranavirus]	100	32	4.55E-13	1
k141_64292_flag1_multi2.5641_len788	W6-1	788	WAK75072.1 hypothetical protein [Mandarin fish ranavirus]	100	40	4.72E-13	6
k141_60220_flag1_multi1.0000_len334	W1-2	334	AYV88167.1 hypothetical protein [Mandarin fish ranavirus]	100	31	4.73E-13	0
k141_68243_flag1_multi2.0000_len510	W5-1	510	QJE49145.1 putative proliferating cell nuclear antigen [Largemouth bass virus]	100	36	4.81E-13	2
k141_40584_flag1_multi5.9201_len454	W5-1	454	QJE49076.1 putative myristylated membrane protein [Largemouth bass virus]	89.4	47	4.93E-13	2
k141_50746_flag1_multi1.0000_len416	W5-1	416	QJE49097.1 hypothetical protein LMBV_034 [Largemouth bass virus]	64.4	59	5.00E-13	0
k141_87159_flag0_multi1.0000_len581	W4-1	581	QJE49145.1 putative proliferating cell nuclear antigen [Largemouth bass virus]	78.4	51	5.13E-13	124.3

k141_67084_flag1_multi6.0000_len344	W2-3	344	WEI29006.1 putative 2-cysteine adaptor domain protein [Largemouth bass virus]	87.5	40	5.28E-13	4
k141_62978_flag1_multi5.3865_len630	W4-1	630	UUY86261.1 hypothetical protein [Largemouth bass virus]	85.7	49	5.35E-13	14
k141_167168_flag0_multi1.0000_len392	W2-3	392	QJE49097.1 hypothetical protein LMBV_034 [Largemouth bass virus]	94.3	35	5.37E-13	0
k141_163803_flag1_multi7.0000_len1038	W5-1	1038	UUY86251.1 hypothetical protein [Largemouth bass virus]	97.2	36	5.50E-13	23
k141_49160_flag1_multi4.1834_len719	W1-1	719	QJE49149.1 putative immediate early protein ICP-46 [Largemouth bass virus]	100	35	5.53E-13	14
k141_85625_flag1_multi4.0000_len324	W1-1	324	WAK75112.1 putative LITAF PIG7 possible membrane associated motif in LPS-induced tumor necrosis factor alpha factor [Mandarin fish ranavirus]	100	30	5.62E-13	5
k141_122634_flag1_multi10.0000_len2009	W2-3	2009	AYV88191.1 hypothetical protein [Mandarin fish ranavirus]	100	35	5.66E-13	55
k141_68503_flag1_multi1.0000_len348	W4-1	348	AYV88120.1 putative p31K protein [Mandarin fish ranavirus]	91.3	46	5.85E-13	40.17
k141_66186_flag1_multi3.0000_len700	W4-1	700	AYV88133.1 putative DNA-directed RNA polymerase II subunit [Mandarin fish ranavirus]	92.5	40	6.10E-13	5
k141_89166_flag1_multi2.6031_len398	W4-1	398	AYV88149.2 hypothetical protein [Mandarin fish ranavirus]	89.5	38	6.29E-13	1
k141_62711_flag0_multi15.9257_len5995	W5-1	5995	AYV88198.1 hypothetical protein [Mandarin fish ranavirus]	100	41	6.81E-13	377
k141_49567_flag1_multi18.8005_len6255	W5-1	6255	QJE49105.1 hypothetical protein LMBV_042 [Largemouth bass virus]	97.8	45	6.88E-13	407
k141_72194_flag1_multi1.9522_len810	W5-1	810	AIG51690.1 major capsid protein, partial [Koi ranavirus]	100	37	6.98E-13	15
k141_6850_flag1_multi1.0000_len373	W5-1	373	QJE49077.1 hypothetical protein LMBV_014 [Largemouth bass virus]	100	32	7.00E-13	1
k141_51870_flag1_multi6.0000_len584	W5-1	584	UUY86192.1 putative myristylated membrane protein [Largemouth bass virus]	97.2	36	7.40E-13	11
k141_43726_flag1_multi3.0000_len682	W5-1	682	UUY86269.1 hypothetical protein [Largemouth bass virus]	93.2	44	7.43E-13	12
k141_10505_flag1_multi6.0000_len1057	W5-1	1057	QIZ30887.1 major capsid protein, partial [Largemouth bass virus]	94.6	37	7.46E-13	23
k141_139723_flag1_multi9.0000_len1032	W5-1	1032	UUY86235.1 hypothetical protein [Largemouth bass virus]	76	50	7.72E-13	38.19
k141_83263_flag1_multi6.8997_len500	W4-1	500	AIG51690.1 major capsid protein, partial [Koi ranavirus]	97.6	41	7.78E-13	5
k141_77376_flag0_multi31.6562_len522	W5-1	522	WAK75072.1 hypothetical protein [Mandarin fish ranavirus]	96.9	32	8.01E-13	3.12
k141_76562_flag1_multi4.0000_len691	W1-3	691	UVF58785.1 MAG: major capsid protein [Halichoeres melanurus ranavirus]	97.4	38	8.25E-13	6
k141_35245_flag1_multi3.0000_len496	W5-1	496	UUY86199.1 hypothetical protein [Largemouth bass virus]	96.9	32	8.48E-13	2
k141_105726_flag3_multi293.7876_len513	W4-1	513	QJE49116.1 hypothetical protein LMBV_053 [Largemouth bass virus]	100	37	8.56E-13	209
k141_53393_flag1_multi9.0000_len1249	W5-1	1249	AYV88120.1 putative p31K protein [Mandarin fish ranavirus]	94.9	39	8.66E-13	19
k141_150796_flag1_multi1.9578_len544	W5-1	544	AYV88167.1 hypothetical protein [Mandarin fish ranavirus]	100	31	8.98E-13	2
k141_30215_flag0_multi2.4795_len579	W5-1	579	XRB52842.1 transcription factor TFIIB cyclin-like domain-containing protein, partial [Largemouth bass virus]	100	41	1.01E-12	3
k141_36372_flag1_multi1.8686_len491	W5-1	491	QJE49104.1 hypothetical protein LMBV_041 [Largemouth bass virus]	92.3	39	1.02E-12	1
k141_11222_flag1_multi7.0543_len785	W4-1	785	AYV88118.1 hypothetical protein [Mandarin fish ranavirus]	100	33	1.04E-12	21
k141_143749_flag0_multi3.8136_len495	W5-1	495	UUY86238.1 hypothetical protein [Largemouth bass virus]	91.7	36	1.05E-12	4
k141_84355_flag1_multi6.0000_len605	W5-1	605	AIG51690.1 major capsid protein, partial [Koi ranavirus]	100	37	1.06E-12	13
k141_120724_flag1_multi1.7979_len616	W5-1	616	AAC79876.1 viral core protein, partial [Labroides dimidatus ranavirus]	100	32	1.09E-12	3
k141_92277_flag0_multi32.0000_len757	W1-1	757	UUY86250.1 hypothetical protein [Largemouth bass virus]	97.5	40	1.12E-12	63
k141_52274_flag1_multi8.0000_len787	W5-1	787	UUY86211.1 hypothetical protein [Largemouth bass virus]	97.4	39	1.19E-12	22
k141_47593_flag1_multi6.0000_len848	W2-1	848	UUY86235.1 hypothetical protein [Largemouth bass virus]	100	39	1.25E-12	10
k141_76948_flag1_multi5.0000_len875	W6-1	875	QYU76034.1 putative myristylated membrane protein, partial [Koi ranavirus]	100	33	1.34E-12	12
k141_155255_flag1_multi6.8432_len983	W5-1	983	QJE49146.1 putative thiol oxidoreductase [Largemouth bass virus]	73.5	49	1.39E-12	25
k141_68069_flag1_multi5.6149_len1055	W4-1	1055	UVF58785.1 MAG: major capsid protein [Halichoeres melanurus ranavirus]	100	40	1.43E-12	11

k141_25167_flag1_multi2.8663_len485	W2-3	485	UUY86229.1 putative DNA dependent RNA polymerase a subunit [Largemouth bass virus]	62.5	64	1.45E-12	4
k141_119606_flag0_multi43.4816_len494	W1-1	494	WEI29006.1 putative 2-cysteine adaptor domain protein [Largemouth bass virus]	64.8	54	1.50E-12	30
k141_7246_flag1_multi8.5152_len504	W5-1	504	QJE49105.1 hypothetical protein LMBV_042 [Largemouth bass virus]	100	40	1.51E-12	11
k141_38910_flag1_multi3.0000_len405	W5-1	405	AYV88120.1 putative p31K protein [Mandarin fish ranavirus]	91.7	36	1.53E-12	5
k141_88323_flag1_multi7.0000_len1699	W2-3	1699	XRB52785.1 hypothetical protein LMBV_28 [Largemouth bass virus]	91.7	36	1.64E-12	41
k141_79260_flag1_multi12.9155_len851	W4-1	851	UUY86227.1 hypothetical protein [Largemouth bass virus]	97.2	36	1.64E-12	25
k141_94911_flag1_multi2.0000_len536	W5-1	536	WEI29006.1 putative 2-cysteine adaptor domain protein [Largemouth bass virus]	82.9	41	1.67E-12	2
k141_131124_flag0_multi2.3139_len552	W5-1	552	UVF58785.1 MAG: major capsid protein [Halichoeres melanurus ranavirus]	92.3	39	1.68E-12	1
k141_41888_flag0_multi25.2162_len548	W2-2	548	QJE49095.1 hypothetical protein LMBV_032 [Largemouth bass virus]	100	34	1.71E-12	6
k141_764_flag1_multi1.6734_len634	W5-1	634	AIG51690.1 major capsid protein, partial [Koi ranavirus]	100	39	1.80E-12	12
k141_18059_flag1_multi2.9034_len1487	W5-1	1487	UUY86235.1 hypothetical protein [Largemouth bass virus]	93	43	1.96E-12	11
k141_39887_flag1_multi17.9836_len810	W5-1	810	ACT85833.1 major capsid protein, partial [Frog virus 3]	82.9	41	1.96E-12	6
k141_61475_flag1_multi1.0000_len545	W4-1	545	AYV88191.1 hypothetical protein [Mandarin fish ranavirus]	56.3	71	1.97E-12	34
k141_161011_flag1_multi1.0000_len845	W5-1	845	UUY86258.1 hypothetical protein [Largemouth bass virus]	97.5	40	1.98E-12	36.42
k141_108819_flag1_multi4.9739_len447	W1-1	447	XRB52768.1 P31K protein, partial [Largemouth bass virus]	97.1	34	1.99E-12	9
k141_20653_flag1_multi1.0000_len355	W5-1	355	UVF58790.1 MAG: myristylated membrane protein, partial [Halichoeres melanurus ranavirus]	100	41	1.99E-12	0
k141_135433_flag0_multi380.8529_len515	W5-1	515	AYV88122.1 putative 3-beta-hydroxy-delta-5-C27 steroid oxidoreductase-like protein [Mandarin fish ranavirus]	97.1	35	2.08E-12	90
k141_39445_flag1_multi1.7154_len394	W2-1	394	QYU76034.1 putative myristylated membrane protein, partial [Koi ranavirus]	58.5	65	2.10E-12	1
k141_103996_flag0_multi81.1204_len789	W4-1	789	UVF58789.1 MAG: NIF/NLI interacting factor [Halichoeres melanurus ranavirus]	91.9	37	2.16E-12	49
k141_35161_flag1_multi1.0000_len334	W2-1	334	QJE49128.1 hypothetical protein LMBV_065 [Largemouth bass virus]	96.9	32	2.20E-12	0
k141_72857_flag1_multi6.9318_len2340	W5-1	2340	QJE49105.1 hypothetical protein LMBV_042 [Largemouth bass virus]	81.1	53	2.30E-12	57
k141_85489_flag1_multi3.0000_len851	W4-1	851	UVF58785.1 MAG: major capsid protein [Halichoeres melanurus ranavirus]	100	39	2.36E-12	9
k141_14530_flag1_multi3.0000_len708	W5-1	708	WEI28972.1 CTD-phosphotransferase [Largemouth bass virus]	91.7	36	2.42E-12	12
k141_162108_flag1_multi2.5621_len785	W5-1	785	XPZ21261.1 hypothetical protein MRVORF002 [Mandarin fish ranavirus]	100	35	2.42E-12	8
k141_99627_flag1_multi10.0000_len483	W1-1	483	AYV88173.2 putative ribonucleotide reductase beta subunit [Mandarin fish ranavirus]	100	34	2.45E-12	14
k141_124114_flag1_multi2.0000_len1406	W5-1	1406	UUY86192.1 putative myristylated membrane protein [Largemouth bass virus]	90.5	42	2.46E-12	11
k141_45509_flag1_multi10.0000_len2348	W5-1	2348	AIG51690.1 major capsid protein, partial [Koi ranavirus]	100	39	2.49E-12	60
k141_75964_flag1_multi6.0000_len1407	W5-1	1407	WHA35528.1 putative replicating factor [Micropterus salmoides ranavirus]	100	38	2.52E-12	30
k141_92236_flag1_multi6.9858_len565	W4-1	565	UVF58785.1 MAG: major capsid protein [Halichoeres melanurus ranavirus]	100	39	2.54E-12	14
k141_36377_flag1_multi1.8534_len523	W5-1	523	UUY86241.1 hypothetical protein [Largemouth bass virus]	96.8	31	2.66E-12	3
k141_137080_flag0_multi3.6413_len325	W1-1	325	XRL22821.1 hypothetical protein [Siniperca chuatsi ranavirus]	100	33	2.67E-12	0
k141_100188_flag0_multi6.8935_len1380	W5-1	1380	QJE49066.1 hypothetical protein LMBV_003 [Largemouth bass virus]	94.7	38	2.77E-12	34
k141_27649_flag1_multi4.0000_len620	W5-1	620	UUY86235.1 hypothetical protein [Largemouth bass virus]	97.1	34	2.88E-12	9
k141_104359_flag0_multi7.4439_len943	W4-1	943	AYV88120.1 putative p31K protein [Mandarin fish ranavirus]	93.2	44	2.95E-12	24
k141_73487_flag0_multi17.4096_len639	W5-1	639	QJE49066.1 hypothetical protein LMBV_003 [Largemouth bass virus]	97.4	38	3.01E-12	34
k141_86208_flag1_multi1.9245_len1664	W1-1	1664	UUY86269.1 hypothetical protein [Largemouth bass virus]	84.6	52	3.04E-12	40
k141_70720_flag1_multi14.9261_len2739	W5-1	2739	AYV88120.1 putative p31K protein [Mandarin fish ranavirus]	72.3	65	3.04E-12	127

k141_24906_flag1_multi9.0000_len632	W2-1	632	AYV88179.2 putative DNA dependent RNA polymerase II second largest subunit [Mandarin fish ranavirus]	94.3	35	3.09E-12	7
k141_75658_flag1_multi5.0000_len501	W2-1	501	UUY86229.1 putative DNA dependent RNA polymerase a subunit [Largemouth bass virus]	97.2	36	3.13E-12	6
k141_77885_flag1_multi1.7580_len608	W1-1	608	QJE49066.1 hypothetical protein LMBV_003 [Largemouth bass virus]	97.1	35	3.15E-12	10
k141_48327_flag1_multi1.4895_len521	W1-1	521	AYV88191.1 hypothetical protein [Mandarin fish ranavirus]	100	32	3.17E-12	6
k141_14028_flag1_multi1.7742_len606	W1-1	606	AYV88191.1 hypothetical protein [Mandarin fish ranavirus]	80.9	47	3.19E-12	3
k141_32228_flag1_multi6.0000_len408	W4-1	408	XPZ21261.1 hypothetical protein MRVORF002 [Mandarin fish ranavirus]	94.7	38	3.20E-12	6
k141_94683_flag1_multi2.0000_len335	W2-2	335	QJE49128.1 hypothetical protein LMBV_065 [Largemouth bass virus]	96.9	32	3.22E-12	2
k141_97829_flag1_multi27.7137_len864	W4-1	864	AIG51690.1 major capsid protein, partial [Koi ranavirus]	100	39	3.32E-12	113
k141_163602_flag1_multi1.0000_len421	W5-1	421	QJE49077.1 hypothetical protein LMBV_014 [Largemouth bass virus]	93.9	33	3.32E-12	7.05
k141_38480_flag1_multi1.9419_len468	W5-1	468	WHU98721.1 MCP, partial [Hybrid snakehead ranavirus]	100	36	3.37E-12	3
k141_128874_flag1_multi11.0853_len3376	W5-1	3376	WAK75107.1 hypothetical protein [Mandarin fish ranavirus]	91.4	35	3.50E-12	127
k141_133930_flag1_multi7.0000_len401	W1-1	401	WEI29006.1 putative 2-cysteine adaptor domain protein [Largemouth bass virus]	100	33	3.55E-12	9
k141_84344_flag1_multi5.1917_len981	W1-1	981	AYV88125.2 hypothetical protein [Mandarin fish ranavirus]	100	46	3.63E-12	19
k141_77511_flag1_multi5.0000_len1281	W2-1	1281	UUY86223.1 hypothetical protein [Largemouth bass virus]	100	39	3.64E-12	21
k141_78668_flag1_multi3.0000_len789	W4-1	789	QJE49101.1 hypothetical protein LMBV_038 [Largemouth bass virus]	100	39	3.74E-12	2
k141_166_flag1_multi8.0000_len414	W5-1	414	WEI28972.1 CTD-phosphotransferase [Largemouth bass virus]	92.7	41	3.75E-12	12
k141_143127_flag1_multi2.0000_len569	W5-1	569	UUY86193.1 hypothetical protein [Largemouth bass virus]	94.4	36	3.81E-12	5
k141_37689_flag1_multi2.6447_len535	W2-3	535	UUY86208.1 hypothetical protein [Largemouth bass virus]	76	50	3.83E-12	5
k141_1947_flag1_multi4.8312_len686	W5-1	686	UUY86258.1 hypothetical protein [Largemouth bass virus]	94.9	39	4.09E-12	15
k141_66643_flag1_multi16.9819_len4549	W4-1	4549	AYV88120.1 putative p31K protein [Mandarin fish ranavirus]	97.4	38	4.11E-12	237
k141_10451_flag1_multi2.3469_len657	W6-1	657	AIG51690.1 major capsid protein, partial [Koi ranavirus]	50	96	4.14E-12	3
k141_3900_flag1_multi1.9485_len588	W2-1	588	AYV88120.1 putative p31K protein [Mandarin fish ranavirus]	92.1	38	4.36E-12	1
k141_40629_flag0_multi5.8358_len811	W2-3	811	WEI29006.1 putative 2-cysteine adaptor domain protein [Largemouth bass virus]	100	32	4.44E-12	6
k141_39644_flag1_multi1.0000_len398	W5-1	398	UUY86267.1 ribonucleotide reductase alpha subunit [Largemouth bass virus]	100	35	4.48E-12	2
k141_37303_flag1_multi2.5531_len772	W5-1	772	QJE49101.1 hypothetical protein LMBV_038 [Largemouth bass virus]	97.1	35	4.74E-12	4
k141_39526_flag1_multi11.0000_len1002	W1-1	1002	AYV88194.1 hypothetical protein [Mandarin fish ranavirus]	92.9	42	4.86E-12	32
k141_27006_flag1_multi4.5598_len350	W6-1	350	UVF58793.1 MAG: DNA polymerase [Halichoeres melanurus ranavirus]	100	33	5.07E-12	3
k141_127194_flag1_multi2.8985_len594	W5-1	594	WAK75112.1 putative LITAF PIG7 possible membrane associated motif in LPS-induced tumor necrosis factor alpha factor [Mandarin fish ranavirus]	96.7	30	5.11E-12	6
k141_12555_flag1_multi4.0000_len979	W5-1	979	AYV88134.2 putative tyrosine kinase [Mandarin fish ranavirus]	100	38	5.18E-12	10
k141_13041_flag1_multi3.0000_len390	W5-1	390	UVF58785.1 MAG: major capsid protein [Halichoeres melanurus ranavirus]	100	38	5.25E-12	0
k141_94970_flag1_multi10.5455_len1263	W5-1	1263	QJE49137.1 hypothetical protein LMBV_074 [Largemouth bass virus]	97.4	38	5.47E-12	40
k141_144060_flag1_multi9.9412_len3200	W5-1	3200	UUY86229.1 putative DNA dependent RNA polymerase a subunit [Largemouth bass virus]	97.5	40	5.54E-12	107
k141_52114_flag1_multi8.5040_len512	W1-1	512	AYV88125.2 hypothetical protein [Mandarin fish ranavirus]	100	46	5.57E-12	1
k141_29945_flag1_multi2.0000_len338	W6-2	338	AYV88172.2 hypothetical protein [Mandarin fish ranavirus]	75	44	6.00E-12	2
k141_145012_flag1_multi13.0000_len1087	W5-1	1087	QYU76034.1 putative myristylated membrane protein, partial [Koi ranavirus]	100	36	6.33E-12	62
k141_104202_flag0_multi17.9454_len8855	W5-1	8855	UUY86235.1 hypothetical protein [Largemouth bass virus]	100	41	6.38E-12	569

k141_58375_flag1_multi1.0000_len400	W2-1	400	AIG51690.1 major capsid protein, partial [Koi ranavirus]	82.6	46	6.57E-12	0
k141_73151_flag1_multi8.6850_len395	W5-1	395	WHU98721.1 MCP, partial [Hybrid snakehead ranavirus]	100	35	7.54E-12	10
k141_101456_flag1_multi1.0000_len566	W4-1	566	AYV88134.2 putative tyrosine kinase [Mandarin fish ranavirus]	100	33	7.76E-12	12
k141_99131_flag0_multi26.8247_len586	W5-1	586	UUY86261.1 hypothetical protein [Largemouth bass virus]	100	40	8.04E-12	37
k141_63767_flag0_multi34.8992_len7562	W1-1	7562	UUY86267.1 ribonucleotide reductase alpha subunit [Largemouth bass virus]	90.5	42	8.16E-12	963
k141_101736_flag1_multi3.0000_len492	W4-1	492	XRB52768.1 P31K protein, partial [Largemouth bass virus]	72.2	54	8.20E-12	5
k141_143975_flag0_multi6.0000_len340	W5-1	340	UUY86235.1 hypothetical protein [Largemouth bass virus]	100	32	8.23E-12	2
k141_43248_flag1_multi25.0000_len1452	W2-3	1452	WEI28972.1 CTD-phosphotransferase [Largemouth bass virus]	76.6	47	8.38E-12	98
k141_47800_flag1_multi6.3241_len1508	W5-1	1508	UUY86193.1 hypothetical protein [Largemouth bass virus]	95	40	9.06E-12	29
k141_40667_flag1_multi5.0000_len511	W1-2	511	AYV88172.2 hypothetical protein [Mandarin fish ranavirus]	97.7	44	9.08E-12	6
k141_6559_flag1_multi4.0000_len1087	W5-1	1087	WHU98721.1 MCP, partial [Hybrid snakehead ranavirus]	100	37	9.08E-12	26
k141_101194_flag1_multi20.4903_len1789	W4-1	1789	AIG51690.1 major capsid protein, partial [Koi ranavirus]	89.1	46	9.24E-12	143
k141_95037_flag1_multi2.5216_len605	W5-1	605	QYU76036.1 helicase-like protein, partial [Koi ranavirus]	100	39	9.30E-12	7
k141_77171_flag1_multi2.5385_len687	W6-1	687	AIG51690.1 major capsid protein, partial [Koi ranavirus]	92.5	40	9.40E-12	5
k141_124087_flag1_multi4.0000_len831	W5-1	831	UVF58785.1 MAG: major capsid protein [Halichoeres melanurus ranavirus]	100	39	9.43E-12	8
k141_78598_flag1_multi3.0000_len406	W4-1	406	QJE49141.1 putative NTPase [Largemouth bass virus]	75	48	9.46E-12	2
k141_83139_flag1_multi10.0000_len967	W5-1	967	XPZ21295.1 putative RNAseIII [Mandarin fish ranavirus]	94.9	39	9.58E-12	14
k141_24940_flag1_multi3.0000_len650	W4-1	650	AYV88176.1 putative tumor necrosis factor receptor [Mandarin fish ranavirus]	100	31	9.73E-12	9
k141_64207_flag1_multi3.0000_len736	W5-1	736	WHA35533.1 putative DNA dependent RNA polymerase A subunit [Micropterus salmoides ranavirus]	86.7	45	9.87E-12	8
k141_53066_flag1_multi2.0000_len494	W5-1	494	QYU76031.1 neurofilament triplet H1-like protein, partial [Koi ranavirus]	100	33	9.99E-12	2
k141_108665_flag1_multi1.5772_len562	W1-1	562	QJE49104.1 hypothetical protein LMBV_041 [Largemouth bass virus]	97.4	38	1.03E-11	1
k141_11552_flag1_multi12.0000_len334	W5-1	334	UUY86235.1 hypothetical protein [Largemouth bass virus]	81.4	43	1.08E-11	0
k141_148763_flag1_multi4.4625_len900	W5-1	900	UUY86197.1 hypothetical protein [Largemouth bass virus]	100	46	1.10E-11	11
k141_92485_flag1_multi20.1911_len680	W5-1	680	WHA35533.1 putative DNA dependent RNA polymerase A subunit [Micropterus salmoides ranavirus]	92.1	38	1.17E-11	62
k141_18056_flag1_multi8.7480_len522	W5-1	522	UUY86267.1 ribonucleotide reductase alpha subunit [Largemouth bass virus]	100	36	1.19E-11	9
k141_10455_flag0_multi1.0000_len347	W5-1	347	UUY86229.1 putative DNA dependent RNA polymerase a subunit [Largemouth bass virus]	97.1	35	1.27E-11	0
k141_76614_flag1_multi2.2732_len668	W5-1	668	WAK75072.1 hypothetical protein [Mandarin fish ranavirus]	92.3	39	1.32E-11	6
k141_148538_flag0_multi12.5550_len950	W5-1	950	UUY86252.1 hypothetical protein [Largemouth bass virus]	98.1	52	1.43E-11	379.56
k141_24984_flag1_multi8.6213_len2972	W5-1	2972	AYV88120.1 putative p31K protein [Mandarin fish ranavirus]	100	37	1.51E-11	81
k141_111035_flag1_multi2.5054_len606	W5-1	606	AYV88179.2 putative DNA dependent RNA polymerase II second largest subunit [Mandarin fish ranavirus]	86	43	1.52E-11	7
k141_87367_flag1_multi2.0000_len446	W5-1	446	QYU76036.1 helicase-like protein, partial [Koi ranavirus]	93.9	33	1.53E-11	0
k141_55671_flag1_multi35.0000_len1678	W4-1	1678	WEI28972.1 CTD-phosphotransferase [Largemouth bass virus]	100	39	1.58E-11	181
k141_114731_flag1_multi5.9419_len1174	W5-1	1174	QJE49149.1 putative immediate early protein ICP-46 [Largemouth bass virus]	100	33	1.58E-11	21
k141_80555_flag1_multi11.9086_len1946	W5-1	1946	UUY86228.1 hypothetical protein [Largemouth bass virus]	92.7	41	1.71E-11	94
k141_81263_flag1_multi13.7152_len1191	W5-1	1191	AYV88183.1 hypothetical protein [Mandarin fish ranavirus]	100	34	1.71E-11	55
k141_107592_flag1_multi5.0000_len676	W5-1	676	QJE49119.1 hypothetical protein LMBV_056 [Largemouth bass virus]	100	32	1.85E-11	13

k141_164124_flag1_multi1.0000_len423	W5-1	423	UUY86263.1 hypothetical protein [Largemouth bass virus]	97.1	34	1.85E-11	3
k141_121533_flag1_multi9.0000_len513	W1-1	513	WAK75107.1 hypothetical protein [Mandarin fish ranavirus]	100	28	1.90E-11	14
k141_126568_flag1_multi1.9630_len546	W1-1	546	UUY86195.1 hypothetical protein [Largemouth bass virus]	97.1	34	1.97E-11	8
k141_32581_flag1_multi1.0000_len481	W4-1	481	UUY86227.1 hypothetical protein [Largemouth bass virus]	97	33	1.97E-11	1
k141_158737_flag1_multi1.8139_len501	W5-1	501	QJE49066.1 hypothetical protein LMBV_003 [Largemouth bass virus]	100	33	2.02E-11	0
k141_78596_flag1_multi3.4959_len385	W4-1	385	AIG51690.1 major capsid protein, partial [Koi ranavirus]	100	35	2.05E-11	6
k141_55390_flag1_multi3.0000_len754	W1-1	754	UUY86258.1 hypothetical protein [Largemouth bass virus]	88.4	43	2.24E-11	8
k141_24470_flag1_multi4.0000_len860	W5-1	860	WHA35533.1 putative DNA dependent RNA polymerase A subunit [Micropterus salmoides ranavirus]	100	34	2.25E-11	10
k141_29839_flag1_multi9.0000_len817	W5-1	817	AYV88191.1 hypothetical protein [Mandarin fish ranavirus]	100	32	2.32E-11	14
k141_49239_flag1_multi4.0000_len831	W6-1	831	AYV88203.1 putative insulin-like growth factor [Mandarin fish ranavirus]	97.4	38	2.52E-11	10
k141_88907_flag1_multi1.6529_len383	W1-1	383	WEI29006.1 putative 2-cysteine adaptor domain protein [Largemouth bass virus]	96.9	32	2.54E-11	0
k141_168239_flag1_multi4.0000_len323	W5-1	323	WAK75112.1 putative LITAF PIG7 possible membrane associated motif in LPS-induced tumor necrosis factor alpha factor [Mandarin fish ranavirus]	93.3	30	2.59E-11	2
k141_57194_flag1_multi19.0000_len1506	W5-1	1506	QJE49066.1 hypothetical protein LMBV_003 [Largemouth bass virus]	92.1	38	2.61E-11	93
k141_101469_flag1_multi3.4428_len543	W5-1	543	QJE49145.1 putative proliferating cell nuclear antigen [Largemouth bass virus]	100	35	2.64E-11	3
k141_116568_flag1_multi6.9662_len496	W5-1	496	ABA41591.1 DNA-dependent DNA polymerase, partial [Largemouth bass virus]	70.2	47	2.65E-11	13
k141_3665_flag1_multi5.0852_len458	W5-1	458	QYU76034.1 putative myristylated membrane protein, partial [Koi ranavirus]	96.8	31	2.65E-11	5
k141_92921_flag1_multi1.0000_len500	W2-1	500	ALR73092.1 major capsid protein, partial [Box turtle ranavirus]	85.3	34	2.66E-11	16
k141_103475_flag1_multi7.6598_len1314	W4-1	1314	WHA35533.1 putative DNA dependent RNA polymerase A subunit [Micropterus salmoides ranavirus]	100	40	2.71E-11	25
k141_135449_flag1_multi11.8443_len1695	W1-1	1695	UUY86238.1 hypothetical protein [Largemouth bass virus]	85	40	2.75E-11	68
k141_78530_flag1_multi9.2727_len493	W5-1	493	ACT85834.1 major capsid protein, partial [Frog virus 3]	79.5	39	2.78E-11	21
k141_26913_flag1_multi2.0000_len372	W2-1	372	UUY86211.1 hypothetical protein [Largemouth bass virus]	100	32	2.79E-11	1
k141_25579_flag1_multi4.5509_len524	W2-1	524	WXI69541.1 hypothetical protein [Largemouth bass virus]	91.4	35	2.81E-11	6
k141_67825_flag1_multi4.0000_len530	W4-1	530	QYU76034.1 putative myristylated membrane protein, partial [Koi ranavirus]	100	33	2.81E-11	9
k141_18703_flag1_multi1.0000_len459	W5-1	459	ABB92283.1 hypothetical protein [Tiger frog virus]	80	45	2.84E-11	1
k141_76944_flag1_multi4.0000_len572	W5-1	572	QJE49097.1 hypothetical protein LMBV_034 [Largemouth bass virus]	100	33	2.89E-11	5
k141_116432_flag1_multi5.0000_len664	W5-1	664	QJE49149.1 putative immediate early protein ICP-46 [Largemouth bass virus]	97.4	38	2.90E-11	10
k141_138995_flag1_multi5.0000_len1102	W5-1	1102	WAK75106.1 hypothetical protein [Mandarin fish ranavirus]	100	31	3.04E-11	12
k141_8381_flag1_multi7.0000_len940	W5-1	940	WEI29006.1 putative 2-cysteine adaptor domain protein [Largemouth bass virus]	53.2	94	3.15E-11	19
k141_39359_flag1_multi1.0000_len596	W4-1	596	WEI29006.1 putative 2-cysteine adaptor domain protein [Largemouth bass virus]	100	32	3.35E-11	16.07
k141_154672_flag1_multi10.5958_len710	W5-1	710	AYV88125.2 hypothetical protein [Mandarin fish ranavirus]	100	46	3.44E-11	26
k141_60892_flag1_multi1.0000_len523	W4-1	523	UUY86266.1 hypothetical protein [Largemouth bass virus]	91.7	36	3.46E-11	0
k141_79049_flag1_multi2.0000_len800	W2-3	800	WHA35533.1 putative DNA dependent RNA polymerase A subunit [Micropterus salmoides ranavirus]	100	35	3.77E-11	13
k141_91165_flag1_multi2.8933_len572	W5-1	572	QJE49137.1 hypothetical protein LMBV_074 [Largemouth bass virus]	100	35	3.89E-11	2
k141_47747_flag1_multi8.1945_len794	W4-1	794	AYV88211.1 hypothetical protein [Mandarin fish ranavirus]	82.2	45	3.98E-11	14
k141_107348_flag1_multi4.0000_len825	W1-1	825	AIG51690.1 major capsid protein, partial [Koi ranavirus]	81.4	43	4.22E-11	14
k141_63664_flag1_multi1.0000_len471	W5-1	471	UUY86261.1 hypothetical protein [Largemouth bass virus]	94.7	38	4.37E-11	4

k141_54510_flag1_multi3.9623_len936	W1-1	936	XRB52771.1 hypothetical protein LMBV_14 [Largemouth bass virus]	100	31	4.81E-11	11
k141_24992_flag1_multi18.8254_len2512	W5-1	2512	AIG51690.1 major capsid protein, partial [Koi ranavirus]	100	39	4.99E-11	128
k141_86417_flag1_multi4.9093_len659	W5-1	659	UUY86258.1 hypothetical protein [Largemouth bass virus]	97.3	37	5.11E-11	12
k141_82722_flag1_multi7.0000_len608	W5-1	608	QYU76034.1 putative myristylated membrane protein, partial [Koi ranavirus]	96.8	31	5.46E-11	13
k141_36831_flag1_multi8.6829_len532	W5-1	532	UUY86211.1 hypothetical protein [Largemouth bass virus]	85.4	41	5.63E-11	15
k141_43402_flag1_multi3.2061_len568	W6-1	568	UUY86267.1 ribonucleotide reductase alpha subunit [Largemouth bass virus]	94.4	36	6.11E-11	8
k141_106100_flag0_multi1.6141_len566	W5-1	566	WEI29006.1 putative 2-cysteine adaptor domain protein [Largemouth bass virus]	97.1	35	6.44E-11	16
k141_104464_flag1_multi12.9739_len600	W4-1	600	AFD96401.1 major capsid protein, partial [Largemouth bass virus]	100	31	7.09E-11	24
k141_70993_flag0_multi14.9085_len447	W5-1	447	QJE49101.1 hypothetical protein LMBV_038 [Largemouth bass virus]	97	33	7.21E-11	7
k141_19260_flag1_multi17.7864_len3366	W4-2	3366	AYV88191.1 hypothetical protein [Mandarin fish ranavirus]	94.1	34	7.63E-11	222
k141_81508_flag1_multi8.0000_len1212	W5-1	1212	WHA35533.1 putative DNA dependent RNA polymerase A subunit [Micropterus salmoides ranavirus]	100	39	8.32E-11	64
k141_117325_flag1_multi4.0000_len436	W4-2	436	AYV88134.2 putative tyrosine kinase [Mandarin fish ranavirus]	100	30	8.70E-11	4
k141_27226_flag1_multi3.0000_len469	W6-1	469	AIG51690.1 major capsid protein, partial [Koi ranavirus]	94.1	34	8.82E-11	5
k141_63299_flag1_multi3.5613_len1119	W5-1	1119	UVF58788.1 MAG: helicase, partial [Halichoeres melanurus ranavirus]	87.8	41	8.92E-11	21
k141_15325_flag1_multi7.1082_len1278	W4-1	1278	UUY86192.1 putative myristylated membrane protein [Largemouth bass virus]	85	40	9.09E-11	21
k141_127255_flag1_multi6.0000_len339	W5-1	339	UVF58793.1 MAG: DNA polymerase [Halichoeres melanurus ranavirus]	100	33	9.83E-11	3
k141_137961_flag1_multi3.3183_len760	W1-1	760	UUY86261.1 hypothetical protein [Largemouth bass virus]	94.7	38	9.90E-11	8
k141_97415_flag0_multi43.9813_len781	W1-1	781	AYV88133.1 putative DNA-directed RNA polymerase II subunit [Mandarin fish ranavirus]	100	30	1.00E-10	301
k141_96034_flag1_multi11.0000_len984	W5-1	984	QIZ30887.1 major capsid protein, partial [Largemouth bass virus]	94.1	34	1.02E-10	33
k141_11708_flag1_multi6.7607_len1796	W6-1	1796	QJE49123.1 putative DNA repair protein RAD2 [Largemouth bass virus]	95	40	1.09E-10	41
k141_69803_flag0_multi77.5781_len506	W4-1	506	AYV88133.1 putative DNA-directed RNA polymerase II subunit [Mandarin fish ranavirus]	100	30	1.14E-10	121
k141_93861_flag1_multi2.0000_len552	W5-1	552	AYV88191.1 hypothetical protein [Mandarin fish ranavirus]	100	30	1.20E-10	8
k141_29612_flag1_multi3.0000_len608	W1-1	608	WEI29006.1 putative 2-cysteine adaptor domain protein [Largemouth bass virus]	62.9	62	1.26E-10	3
k141_123994_flag1_multi35.0000_len858	W1-1	858	UUY86199.1 hypothetical protein [Largemouth bass virus]	100	28	1.27E-10	76
k141_57435_flag1_multi3.2834_len568	W4-1	568	AYV88120.1 putative p31K protein [Mandarin fish ranavirus]	93.9	33	1.35E-10	10
k141_127492_flag0_multi7.9562_len3975	W5-1	3975	UUY86235.1 hypothetical protein [Largemouth bass virus]	94.7	38	1.40E-10	103
k141_48195_flag1_multi1.0000_len557	W6-1	557	UUY86241.1 hypothetical protein [Largemouth bass virus]	100	28	1.40E-10	1
k141_76918_flag1_multi14.1547_len1634	W1-1	1634	AIG51690.1 major capsid protein, partial [Koi ranavirus]	100	38	1.43E-10	58
k141_12396_flag0_multi1.0000_len485	W5-1	485	AYV88179.2 putative DNA dependent RNA polymerase II second largest subunit [Mandarin fish ranavirus]	100	32	1.45E-10	4
k141_76556_flag1_multi4.6014_len427	W4-1	427	AIG51690.1 major capsid protein, partial [Koi ranavirus]	72	50	1.49E-10	1
k141_43246_flag1_multi3.2083_len674	W5-1	674	QJE49101.1 hypothetical protein LMBV_038 [Largemouth bass virus]	88.6	35	1.50E-10	4
k141_154196_flag1_multi4.0000_len652	W5-1	652	QJE49107.1 hypothetical protein LMBV_044 [Largemouth bass virus]	56.3	64	1.51E-10	5
k141_33460_flag1_multi14.0000_len1742	W1-1	1742	WEI29006.1 putative 2-cysteine adaptor domain protein [Largemouth bass virus]	97.2	36	1.52E-10	97
k141_110062_flag1_multi3.9739_len486	W5-1	486	QYU76036.1 helicase-like protein, partial [Koi ranavirus]	100	35	1.56E-10	7
k141_53168_flag1_multi7.0000_len532	W4-1	532	AIG51690.1 major capsid protein, partial [Koi ranavirus]	100	36	1.57E-10	16
k141_38750_flag1_multi2.5582_len906	W5-1	906	QJE49072.1 hypothetical protein LMBV_009 [Largemouth bass virus]	93.9	33	1.62E-10	13.2

k141_20843_flag1_multi4.7920_len516	W1-1	516	UUY86261.1 hypothetical protein [Largemouth bass virus]	100	37	1.69E-10	9
k141_18345_flag1_multi3.0000_len411	W2-1	411	UUY86235.1 hypothetical protein [Largemouth bass virus]	91.9	37	1.79E-10	3
k141_101323_flag0_multi60.7082_len398	W5-1	398	QJE49101.1 hypothetical protein LMBV_038 [Largemouth bass virus]	97	33	1.80E-10	3
k141_166788_flag1_multi1.5876_len512	W5-1	512	QJE49101.1 hypothetical protein LMBV_038 [Largemouth bass virus]	97.1	35	1.81E-10	1
k141_1796_flag1_multi4.0000_len332	W5-1	332	AIG51690.1 major capsid protein, partial [Koi ranavirus]	69.6	56	1.93E-10	3
k141_44673_flag1_multi6.0000_len1273	W5-1	1273	AIG51690.1 major capsid protein, partial [Koi ranavirus]	100	35	2.00E-10	20
k141_59542_flag0_multi14.8758_len2621	W5-1	2621	AYV88191.1 hypothetical protein [Mandarin fish ranavirus]	97	33	2.00E-10	84
k141_106505_flag1_multi1.0000_len492	W2-3	492	AIG51690.1 major capsid protein, partial [Koi ranavirus]	75	48	2.07E-10	0
k141_158443_flag1_multi11.0000_len2521	W5-1	2521	QIZ30887.1 major capsid protein, partial [Largemouth bass virus]	88.2	34	2.18E-10	101
k141_56601_flag1_multi0.9780_len414	W5-1	414	UUY86267.1 ribonucleotide reductase alpha subunit [Largemouth bass virus]	96.8	31	2.24E-10	0
k141_17275_flag1_multi7.3052_len2084	W5-1	2084	AYV88211.1 hypothetical protein [Mandarin fish ranavirus]	90.5	42	2.24E-10	51
k141_3693_flag1_multi1.0000_len771	W6-1	771	ACT85833.1 major capsid protein, partial [Frog virus 3]	90.9	33	2.32E-10	22
k141_77359_flag1_multi5.9970_len811	W4-1	811	XRB52801.1 hypothetical protein LMBV_44 [Largemouth bass virus]	78.9	38	2.44E-10	20
k141_160075_flag1_multi4.7631_len2032	W5-1	2032	QJE49137.1 hypothetical protein LMBV_074 [Largemouth bass virus]	81.8	44	2.50E-10	31
k141_137468_flag1_multi1.0000_len605	W2-3	605	UUY86235.1 hypothetical protein [Largemouth bass virus]	88.9	36	2.59E-10	9
k141_138326_flag1_multi4.9004_len1065	W5-1	1065	UVF58789.1 MAG: NIF/NLI interacting factor [Halichoeres melanurus ranavirus]	100	30	2.84E-10	21
k141_101460_flag1_multi4.0000_len498	W2-2	498	WAK75102.1 hypothetical protein [Mandarin fish ranavirus]	97	33	2.88E-10	4
k141_89416_flag1_multi2.7789_len521	W1-1	521	WAK75097.1 hypothetical protein [Mandarin fish ranavirus]	94.4	36	2.89E-10	2
k141_118087_flag1_multi4.0746_len677	W5-1	677	QJE49072.1 hypothetical protein LMBV_009 [Largemouth bass virus]	93.8	32	2.90E-10	8
k141_87378_flag1_multi1.9954_len575	W5-1	575	UUY86223.1 hypothetical protein [Largemouth bass virus]	100	35	2.93E-10	4
k141_30388_flag1_multi5.0000_len465	W6-1	465	QIZ30887.1 major capsid protein, partial [Largemouth bass virus]	78	41	2.94E-10	8
k141_49005_flag1_multi20.0000_len1067	W5-1	1067	UVF58785.1 MAG: major capsid protein [Halichoeres melanurus ranavirus]	100	39	2.97E-10	66
k141_50718_flag1_multi4.0000_len321	W5-1	321	AYV88167.1 hypothetical protein [Mandarin fish ranavirus]	100	26	3.00E-10	4
k141_38266_flag1_multi17.0000_len1699	W5-1	1699	WHA35528.1 putative replicating factor [Micropterus salmoides ranavirus]	100	32	3.17E-10	89
k141_8292_flag1_multi2.9864_len583	W2-1	583	AYV88169.1 hypothetical protein [Mandarin fish ranavirus]	97	33	3.34E-10	4
k141_136803_flag1_multi5.0000_len832	W1-1	832	XRL22821.1 hypothetical protein [Siniperca chuatsi ranavirus]	88.9	36	3.47E-10	12
k141_115202_flag1_multi19.0000_len3819	W1-1	3819	AYV88143.1 hypothetical protein [Mandarin fish ranavirus]	100	35	3.52E-10	178
k141_74620_flag1_multi4.2239_len476	W5-1	476	UVF58788.1 MAG: helicase, partial [Halichoeres melanurus ranavirus]	91.9	37	3.62E-10	8
k141_159823_flag1_multi3.0000_len957	W5-1	957	QYU76034.1 putative myristylated membrane protein, partial [Koi ranavirus]	95.1	41	4.34E-10	15
k141_43662_flag1_multi5.0000_len308	W4-1	308	AXB27397.1 major capsid protein, partial [Ranavirus sp.]	88.6	35	4.47E-10	5
k141_869_flag1_multi1.7486_len491	W5-1	491	WXI69541.1 hypothetical protein [Largemouth bass virus]	97	33	4.68E-10	3
k141_16898_flag1_multi6.8099_len1372	W5-1	1372	AYV88125.2 hypothetical protein [Mandarin fish ranavirus]	77	61	4.84E-10	30
k141_80605_flag0_multi1.0000_len223	W4-1	223	WXI69513.1 hypothetical protein [Largemouth bass virus]	70.8	72	5.07E-10	204
k141_61315_flag1_multi4.9642_len1258	W1-1	1258	AYV88191.1 hypothetical protein [Mandarin fish ranavirus]	100	33	5.24E-10	16
k141_110331_flag1_multi3.1725_len628	W1-1	628	AYV88179.2 putative DNA dependent RNA polymerase II second largest subunit [Mandarin fish ranavirus]	97.1	35	5.32E-10	6
k141_16031_flag1_multi4.0000_len629	W4-1	629	AYV88120.1 putative p31K protein [Mandarin fish ranavirus]	74.5	47	5.58E-10	7
k141_38047_flag1_multi4.0000_len651	W4-1	651	AYV88211.1 hypothetical protein [Mandarin fish ranavirus]	100	36	5.63E-10	1
k141_122957_flag0_multi3.0110_len414	W5-1	414	WEI29006.1 putative 2-cysteine adaptor domain protein [Largemouth bass virus]	100	33	5.76E-10	5
k141_168723_flag1_multi1.9413_len533	W5-1	533	QJE49137.1 hypothetical protein LMBV_074 [Largemouth bass virus]	100	32	5.81E-10	4

k141_119478_flag0_multi10.7101_len3094	W5-1	3094	WAK75072.1 hypothetical protein [Mandarin fish ranavirus]	100	36	5.83E-10	98.69
k141_26549_flag1_multi4.4757_len1110	W2-1	1110	WEI28966.1 hypothetical protein [Largemouth bass virus]	88.1	42	6.37E-10	23
k141_128060_flag1_multi4.9222_len861	W1-1	861	XRL22790.1 hypothetical protein [Siniperca chuatsi ranavirus]	97.3	37	7.24E-10	14
k141_14651_flag1_multi6.0000_len571	W5-1	571	UUY86199.1 hypothetical protein [Largemouth bass virus]	100	27	7.25E-10	8
k141_114117_flag1_multi6.0000_len447	W5-1	447	AYV88191.1 hypothetical protein [Mandarin fish ranavirus]	91.2	34	7.34E-10	10
k141_98925_flag1_multi3.0000_len707	W5-1	707	UUY86258.1 hypothetical protein [Largemouth bass virus]	82.5	40	8.15E-10	5
k141_41694_flag1_multi4.0000_len1010	W5-1	1010	AYV88191.1 hypothetical protein [Mandarin fish ranavirus]	100	32	8.51E-10	24
k141_62496_flag1_multi38.4805_len2528	W1-1	2528	WHA35533.1 putative DNA dependent RNA polymerase A subunit [Micropterus salmoides ranavirus]	100	34	8.63E-10	332
k141_59986_flag1_multi5.0457_len754	W5-1	754	UUY86257.1 hypothetical protein [Largemouth bass virus]	100	30	9.46E-10	12
k141_91600_flag1_multi4.0000_len309	W4-1	309	QJE49144.1 hypothetical protein LMBV_081 [Largemouth bass virus]	67.3	52	1.71E-09	2
k141_50675_flag1_multi13.7958_len905	W1-1	905	UUY86261.1 hypothetical protein [Largemouth bass virus]	100	36	1.75E-09	36
k141_150797_flag1_multi3.0000_len938	W5-1	938	QJE49104.1 hypothetical protein LMBV_041 [Largemouth bass virus]	97.1	34	2.07E-09	7
k141_15034_flag1_multi8.0000_len1086	W5-1	1086	UUY86261.1 hypothetical protein [Largemouth bass virus]	100	39	2.62E-09	25
