# Supplementary material for: A new lineage of Ranavirus micropterus1 infects ornamental wrasses (Macropharyngodon choati) from the Great Barrier Reef and causes severe disease in captivity
Source: Front Vet Sci. 2026 May 18;13:1829414. doi: 10.3389/fvets.2026.1829414 (PMC13224474; doi:10.3389/fvets.2026.1829414)
Supplement: Supplementary file 2 [file Table_2.PDF]

Table S2. Top 1000 expressed host genes.

W1.1_spleen	W1.2_liver	W1.3_brain	W2.1_spleen	W2.2_liver	W2.3_brain	W3.1_spleen	W3.2_liver	W3.3_brain	W4.1_spleen	W4.2_liver	W4.3_brain	W5.1_spleen	W5.2_liver	W5.3_brain	W6.1_spleen	W6.2_liver	W6.3_brain	Total counts	Protein names	Gene names	Gene Ontology (Biological Process)
396696.4	409326.69	0	394744.67	237728.71	1691.56	2033.97	290103.14	0	690273.55	753442.86	0	208038.05	246538.5	0	355145.83	605680.27	16780.83	4608225.03	Nucleoredoxin (EC 1.8.1.8)	nxn zgc:110449	cell differentiation [GO:0030154]; circulatory system development [GO:0072359]; negative regulation of protein ubiquitination [GO:0031397]; negative regulation of Wnt signaling pathway [GO:0030178]; Wnt signaling pathway [GO:0016055]
188775.06	254248.56	120008.44	444064.98	207158.42	752312.47	1634.61	160837.75	59854.22	397073.85	249766.89	1191591.13	249225.44	86684.59	75011.34	22533.79	74381.69	67234.25	4602397.48	Receptor-type tyrosine-protein phosphatase eta (Protein-tyrosine phosphatase eta) (R-PTP-eta) (EC 3.1.3.48) (HPTP eta) (Protein-tyrosine phosphatase receptor type J) (R-PTP-J) (Supporting-cell antigen)	PTPRJ	negative regulation of epidermal grow factor receptor signaling pathway [GO:0042059]; negative regulation of MAP kinase activity [GO:0043407]; negative regulation of phosphatidylinositol 3-kinase/protein kinase B signal transduction [GO:0051898]; negative regulation of T cell receptor signaling pathway [GO:0050860]; platelet formation [GO:0030220]; platelet-derived growth factor receptor signaling pathway [GO:0048008]; positive regulation of ce adhesion [GO:0045785]; positive regulation of focal adhesion assembly [GO:0051894]

3129.37	2.35	71.93	83626.61	2.93	571.54	1038674.27	1.8	131.8	6129.62	14181.99	51.92	300.37	7.48	197.6	602658.55	15099.23	261.65	1765101.01	Eukaryotic translation initiation factor 2-alpha kinase 3 (EC 2.7.11.1) (PRKR-like endoplasmic reticulum kinase) (Pancreatic eIF2-alpha kinase) (Protein tyrosine kinase EIF2AK3) (EC 2.7.10.2)	Eif2ak3 Pek Perk	angiogenesis [GO:0001525]; bone mineralization [GO:0030282]; calcium-mediated signaling [GO:0019722]; cellular response to amino acid starvation [GO:0034198]; cellular response to cold [GO:0070417]; cellular response to glucose starvation [GO:0042149]; chondrocyte development [GO:0002063]; endocrine pancreas development [GO:0031018]; endoplasmic reticulum organization [GO:0007029]; endoplasmic reticulum unfolded protein response [GO:0030968]; ER overload response [GO:0006983]; ERAD pathway [GO:0036503]; fat cell differentiation [GO:0045444]; insulin-like growth factor receptor signaling pathway [GO:0048009]; intrinsic apoptotic signaling pathway in response to endoplasmic reticulum stress [GO:0070059]; lactation [GO:0007595]; negative regulation of apoptotic process [GO:0043066]; negative regulation of gene expression [GO:0010629]; negative regulation of myelination [GO:0031642];
376417.3	4169.88	2186.3	102710.03	28628.32	52729.12	1580.48	2527.13	16.5	234002.85	20185.64	10985.18	769111.54	2977.48	3184.9	18004.34	5263.68	220.69	1634901.36	Histone H1 [Cleaved into: Oncorhyncin II]	LOC141806056	defense response to bacterium [GO:0042742]; nucleosome assembly [GO:0006334]
79168.92	61476.22	68384.67	26432.59	66918.9	158670.16	10623.24	29755.86	115301.45	45097.35	23062.29	54267.14	126804.59	144889.86	169443.96	49958.84	74016.03	184482.15	1488754.22	Tumor necrosis factor receptor superfamily member 1B (Tumor necrosis factor receptor 2) (TNF-R2) (Tumor necrosis factor receptor type II) (TNF-RII) (TNFR-II) (p75) (p80 TNF-alpha receptor) (CD antigen CD120b) (Etanercept) [Cleaved into: Tumor necrosis factor receptor superfamily member 1b, membrane form;Tumor necrosis factor-binding protein 2 (TBP-2) (TBPII)]	TNFRSF1B TNFBR TNFR2	aortic valve development [GO:0003176]; cellular response to lipopolysaccharide [GO:0071222]; extrinsic apoptotic signaling pathway [GO:0097191]; glial cell-neuron signaling [GO:0150098]; inflammatory response [GO:0006954]; intrinsic apoptotic signaling pathway in response to DNA damage [GO:0008630]; negative regulation of cardiac muscle hypertrophy [GO:0010614]; negative regulation of extracellular matrix constituent secretion [GO:0003332]; negative regulation of neuroinflammatory response [GO:0150079]; positive regulation of apoptotic process involved in morphogenesis [GO:1902339]; positive regulation of membrane protein ectodomain proteolysis [GO:0051044]; positive regulation of myelination [GO:0031643]; positive regulation of oligodendrocyte differentiation [GO:0048714]; pulmonary valve development [GO:0003177]; regulation of cytokine production involved in

1891.59	2.29	16.03	61180.55	0	622.23	733032.4	0	0	3243.7	7843.86	5.97	192.43	0	0	507281.15	11733.32	197.01	1327242.53	Nuclear receptor subfamily 1 group D member 2 (Orphan nuclear hormone receptor BD73) (Rev-erb alpha-related receptor) (RVR) (Rev-erb-beta) (V-erbA-related protein 1-related) (EAR-1R)	NR1D2	cell differentiation [GO:0030154]; circadian behavior [GO:0048512]; energy homeostasis [GO:0097009]; hormone-mediated signaling pathway [GO:0009755]; intracellular receptor signaling pathway [GO:0030522]; lipid homeostasis [GO:0055088]; negative regulation of DNA-templated transcription [GO:0045892]; negative regulation of inflammatory response [GO:0050728]; negative regulation of transcription by RNA polymerase II [GO:0000122]; positive regulation of DNA-templated transcription [GO:0045893]; positive regulation of transcription by RNA polymerase II [GO:0045944]; regulation of circadian rhythm [GO:0042752]; regulation of DNA-templated transcription [GO:0006355]; regulation of inflammatory response [GO:0050727]; regulation of lipid metabolic process [GO:0019216]; regulation of skeletal muscle cell differentiation [GO:2001014]
171973.47	2446.59	1307.72	89095.66	17463.73	22240.65	1560.67	1652.63	45.32	486158.8	11032.74	9872.92	384390.13	1498.7	1446.39	14378.14	3039.5	126.97	1219730.73	General transcription factor II-I repeat domain-containing protein 2 (GTF2I repeat domain-containing protein 2) (Transcription factor GTF2IRD2)	GTF2IRD2	transition between fast and slow fiber [GO:0014883]
52087.46	57349.11	156292.68	30038.46	73490.84	104232.21	643.13	72278.55	87798.38	56910.39	63829.75	180356.45	18780.13	29511.83	55413.17	3564.24	19552.83	55092.9	1117222.51	N-acylglucosamine 2-epimerase (AGE) (EC 5.1.3.8) (GlcNAc 2-epimerase) (N-acetyl-D-glucosamine 2-epimerase) (Renin-binding protein) (RnBP)	RENBP	carbohydrate metabolic process [GO:0005975]; N-acetylglucosamine metabolic process [GO:0006044]; N-acetylmannosamine metabolic process [GO:0006051]; N-acetylneuraminate catabolic process [GO:0019262]; regulation of blood pressure [GO:0008217]

35873.73	60619.86	27748.84	71686.5	55455.05	168778.11	197.37	48075.94	25398.11	42988.84	38832.78	215865.61	70513.59	45005.97	29814.76	9549.62	24950.51	30370.83	1001726.02	Nuclear pore complex protein Nup160 (160 kDa nucleoporin) (Gene trap locus 1-13 protein) (GTL-13) (Nucleoporin Nup160)	Nup160 Gtl1-13 Kiaa0197	mRNA export from nucleus [GO:0006406]; nucleocytoplasmic transport [GO:0006913]; protein transport [GO:0015031]
40071.19	60971.45	11220.57	85612.54	22118.77	98743.48	437.8	27008.14	8344.56	98628	57965.83	101873.15	19260.16	7358.7	6912.99	1945.33	4682.56	7623.48	660778.7	Tuftelin-interacting protein 11 (Septin and tuftelin-interacting protein 1) (STIP-1)	tfip11 stip zgc:86644	spliceosomal complex disassembly [GO:0000390]
25865.79	36614.35	17977.1	57614.89	34291.86	104493.92	169.48	20601.61	9689.2	44098.41	33538.6	149458.42	36155.26	16430.65	17722.88	4376.83	12076.87	11989.58	633165.7	Dynein axonemal heavy chain 12 (Bm259)	Dnah12	cilium movement involved in cell motility [GO:0060294]
137350.44	1514.17	780.7	28905.85	6242.65	20003.95	342.68	697.53	21.39	77241.27	4189.84	6242.6	316725.69	845.83	1281.13	7369.48	1436.98	107.49	611299.67	U3 small nucleolar RNA-associated protein 18 homolog (WD repeat-containing protein 50)	UTP18 WDR50 CDABP0061 CGI-48	ribosomal small subunit biogenesis [GO:0042274]; rRNA processing [GO:0006364]
1264.27	10.62	13.26	15401.15	21.73	343.37	332056.54	2.21	3.28	3310.84	7894.86	42.21	453.83	23.92	31.26	233689.16	5884.87	115.05	600562.43	Transcriptional regulator Myc (c-Myc)	myc	
32606.24	31014.44	59489.18	14453.25	42907.98	61770.06	236.64	38146.19	59246.94	29962.83	24713.07	84550.28	14583.44	17933.87	37634.88	2135.94	11997.6	35774.9	599157.73	Complement C3 [Cleaved into: Complement C3 beta chain;Complement C3 alpha chain;C3a anaphylatoxin;Complement C3b (Complement C3b-alpha' chain)]	c3	complement activation, alternative pathway [GO:0006957]; complement activation, classical pathway [GO:0006958]; defense response to bacterium [GO:0042742]; inflammatory response [GO:0006954]

127332.8	1761.45	1185.15	27242.71	3420.46	8606.8	367.23	503.05	218.79	100439.32	3003.21	6009.27	290847.44	794.61	1225.05	8365.43	1435.83	329.33	583087.93	Polycomb protein eed	eed zgc:112509	digestive tract development [GO:0048565]; exocrine pancreas development [GO:0031017]; heterochromatin formation [GO:0031507]; liver development [GO:0001889]; negative regulation of transcription by RNA polymerase II [GO:0000122]; regulation of glial cell differentiation [GO:0045685]; regulation of neuron differentiation [GO:0045664]
14789.4	15559.83	36916.71	10797.3	35845	60055.25	1060.12	23289.26	62584.49	13598.39	13661	72109.13	27372.61	40053.7	81772.3	3586.89	17696.27	38316.17	569063.82	Arginine-glutamic acid dipeptide repeats protein (Atrophin-2)	Rere Atr2 Kiaa0458	branching morphogenesis of a nerve [GO:0048755]; cerebellar granule cell precursor proliferation [GO:0021930]; cerebellar Purkinje cell layer maturation [GO:0021691]; cerebellum development [GO:0021549]; chromatin remodeling [GO:0006338]; dendrite morphogenesis [GO:0048813]; radial glia guided migration of Purkinje cell [GO:0021942]
25917.17	39463.73	34.72	106781.18	68634.76	743.75	295.62	39118.7	6.39	72041.14	105981.72	7.2	11655.37	15909.43	7.1	22299.39	50553.02	523.15	559973.54	Enkurin domain-containing protein 1	ENKD1	establishment of mitotic spindle orientation [GO:0000132]; motile cilium assembly [GO:0044458]; non-motile cilium assembly [GO:1905515]

35587.75	76927.31	11475.43	8823.94	32617.48	21397.28	9345.36	28196.4	7713.04	6376.19	16803.06	4833.65	50546.9	107044.11	7420.56	6398.97	48864.24	10064.82	490436.49	U5 small nuclear ribonucleoprotein 200 kDa helicase (EC 3.6.4.13) (Activating signal cointegrator 1 complex subunit 3-like 1) (BRR2 homolog) (U5 snRNP-specific 200 kDa protein) (U5-200KD)	SNRNP200 ASCC3L1 BRR2 HELIC2 KIAA0788	cis assembly of pre-catalytic spliceosome [GO:0000354]; mRNA splicing, via spliceosome [GO:0000398]; osteoblast differentiation [GO:0001649]; spliceosome conformational change to release U4 (or U4atac) and U1 (or U11) [GO:0000388]
26356.48	15368.97	43895.18	9511.3	14117.8	32112.95	60632.54	3893.38	12187.89	7906.3	5589.84	65581.83	51632.4	31413.72	26396.14	33568.32	26382.22	21664.5	488211.76	mRNA export factor GLE1 (GLE1 RNA export mediator) (GLE1-like protein) (Nucleoporin GLE1)	GLE1 GLE1L	poly(A)+ mRNA export from nucleus [GO:0016973]; protein transport [GO:0015031]
81.66	166150.38	151.95	223.38	49289.06	152.32	17.43	63383.53	96.75	185.35	17917.86	97.9	117.28	85341.43	146.89	53.64	99474.71	806.5	483688.02	Cell cycle control protein 50A (P4-ATPase flippase complex beta subunit TMEM30A) (Transmembrane protein 30A)	TMEM30A CDC50A	phospholipid translocation [GO:0045332]
1269.06	13.31	18.81	8929.2	18.6	215.62	267315.7	5.81	1.92	2142.51	4604.14	35.44	565.65	30.43	39.52	174972.33	4038.35	102.06	464318.46	Transmembrane protein 268	TMEM268	

17587.51	29300.38	9088.47	35978.08	27206.71	79426.52	63.66	19079.74	9112.17	16930.08	15680.98	101869.59	31085.96	16194.8	9557.01	3464.7	10975.68	11609.55	444211.59	Phospholipid phosphatase 3 (EC 3.1.3.-) (EC 3.1.3.4) (Lipid phosphate phosphohydrolase 3) (PAP2-beta) (Phosphatidate phosphohydrolase type 2b) (Phosphatidic acid phosphatase 2b) (PAP-2b) (PAP2b) (Vascular endothelial growth factor and type I collagen-inducible protein) (VCIP)	PLPP3 LPP3 PPAP2B	Bergmann glial cell differentiation [GO:0060020]; blood vessel development [GO:0001568]; cell-cell adhesion [GO:0098609]; cell-cell adhesion mediated by integrin [GO:0033631]; ceramide metabolic process [GO:0006672]; gastrulation with mouth forming second [GO:0001702]; integrin-mediated signaling pathway [GO:0007229]; phospholipid dephosphorylation [GO:0046839]; phospholipid metabolic process [GO:0006644]; positive regulation of endothelial cell migration [GO:0010595]; positive regulation of endothelial cell-matrix adhesion via fibronectin [GO:1904906]; positive regulation of homotypic cell-cell adhesion [GO:0034112]; positive regulation of intracellular signal transduction [GO:1902533]; positive regulation of transcription by RNA polymerase II [GO:0045944]; protein stabilization [GO:0050821]; regulation of sphingolipid mediated signaling pathway [GO:1902068]; regulation of
37754.23	51475.86	39.19	82775.22	41970.5	442.66	223.63	32131.35	55.86	27345.15	38108.82	24.39	22796.77	30192.78	52.9	23887.61	53947.81	467.22	443691.95	Zinc-binding protein A33		
87831.81	1526.18	690.32	24373.2	13681.36	24203.41	185.11	1046.97	31.63	43637.77	8391.75	3287.88	215223.85	1032.87	1288.89	5322.94	2178.11	76.36	434010.41	Histone-lysine N-methyltransferase SETD5 (EC 2.1.1.359) (EC 2.1.1.367) (SET domain-containing protein 5)	Setd5 Kiaa1757	cognition [GO:0050890]; methylation [GO:0032259]; negative regulation of transcription by RNA polymerase III [GO:0016480]; regulation of chromatin organization [GO:1902275]; regulation of DNA-templated transcription elongation [GO:0032784]; regulation of synapse assembly [GO:0051963]

0	0	184417.91	0	0	225891.12	0	0	0	0	0	0	0	0	11771.17	0	0	0	422080.2	Ras-related protein Rab-39B (EC 3.6.5.2)	RAB39B	autophagy [GO:0006914]; protein transport [GO:0015031]; Rab protein signal transduction [GO:0032482]; regulation of autophagy [GO:0010506]; synapse organization [GO:0050808]; vesicle-mediated transport [GO:0016192]
15935.91	20444.27	21949.87	17198.21	24894.66	31660.63	33853.08	9338.02	6016.79	9414.8	11626.37	23189.42	28810.85	44007.52	11855.57	34071.65	23523.21	9607.92	377398.75	Phospholipid scramblase 1 (PL scramblase 1) (Ca(2+)-dependent phospholipid scramblase 1) (Erythrocyte phospholipid scramblase) (Mg(2+)-dependent nuclease) (EC 3.1.-.-) (MmTRA1b)	PLSCR1	acute-phase response [GO:0006953]; apoptotic process [GO:0006915]; defense response to virus [GO:0051607]; negative regulation of phagocytosis [GO:0050765]; negative regulation of viral genome replication [GO:0045071]; phosphatidylserine biosynthetic process [GO:0006659]; phosphatidylserine exposure on apoptotic cell surface [GO:0070782]; plasma membrane phospholipid scrambling [GO:0017121]; platelet activation [GO:0030168]; positive regulation of chromosome separation [GO:1905820]; positive regulation of DNA topoisomerase (ATP-hydrolyzing) activity [GO:2000373]; positive regulation of gene expression [GO:0010628]; positive regulation of innate immune response [GO:0045089]; positive regulation of transcription by RNA polymerase II [GO:0045944]; regulation of Fc receptor mediated stimulatory signaling pathway [GO:0060368]; regulation of mast cell activation [GO:0033003]; response to [illegible]
69547.43	801.91	604.46	17637.8	3836.56	15592.51	263.47	365.11	3.43	53547.35	2164.57	3361.47	202445.45	411.53	1014.88	4070.98	828.23	24.53	376521.67	SREBP regulating gene protein	spring zgc:110063	intestinal lipid absorption [GO:0098856]; positive regulation of SREBP signaling pathway [GO:2000640]

16.47	39.94	72376.02	5.73	0.97	8777	47.81	0.76	316.57	6.19	3.39	286527.42	4.63	15.72	1392.47	39.53	14.86	569.92	370155.4	CD48 antigen (BCM1 surface antigen) (BLAST-1) (HM48-1) (MRC OX-45 surface antigen) (SLAM family member 2) (SLAMF2) (Signaling lymphocytic activation molecule 2) (sgp-60) (CD antigen CD48)	Cd48 Bcm-1	signal transduction [GO:0007165]; T cell activation [GO:0042110]
11921.65	12066.12	16668.62	8634.84	12641.18	22381.49	97479.05	3790.75	11703.73	6240.73	8163.46	13598.11	25056.77	14957.16	10724.18	52934.21	14403.75	14156.28	357522.08	V-type immunoglobulin domain-containing suppressor of T-cell activation (Platelet receptor Gi24) (V-set domain-containing immunoregulatory receptor) (V-set immunoregulatory receptor)	Vsir Dies1 PD-1H VISTA	BMP signaling pathway [GO:0030509]; negative regulation of CD4-positive, alpha-beta T cell proliferation [GO:2000562]; negative regulation of T cell cytokine production [GO:0002725]; positive regulation of BMP signaling pathway [GO:0030513]; positive regulation of stem cell differentiation [GO:2000738]; stem cell differentiation [GO:0048863]

14550.77	20528.65	10776.81	18338.85	19827.88	61196.6	199.62	16524.88	11156.66	15469.87	17040.83	40623.49	20001.89	17389.16	11852.45	3696.59	8743.15	11036.86	318955.01	Fos-related antigen 2 (FRA-2)	Fosl2 Fra-2 Fra2	alveolar secondary septum development [GO:0061144]; B cell differentiation [GO:0030183]; B cell proliferation [GO:0042100]; bone development [GO:0060348]; bone mineralization [GO:0030282]; cartilage development [GO:0051216]; cell morphogenesis [GO:0000902]; chondrocyte differentiation [GO:0002062]; chondrocyte proliferation [GO:0035988]; collagen biosynthetic process [GO:0032964]; collagen metabolic process [GO:0032963]; fat cell apoptotic process [GO:1904606]; fat cell differentiation [GO:0045444]; fat pad development [GO:0060613]; gene expression [GO:0010467]; glucose homeostasis [GO:0042593]; growth plate cartilage development [GO:0003417]; homeostasis of number of cells [GO:0048872]; homeostasis of number of cells within a tissue [GO:0048873]; inflammatory response [GO:0006954]; inflammatory response to antigenic stimulus [GO:0002437];
7652.26	12549.91	16197.39	10724.51	20960.88	25833.53	21153.09	8345.99	4171.41	6199.58	9466.68	25866.46	19464.79	43806.11	8133.99	20963.54	20592.4	6357.34	288439.86	Ephrin type-A receptor 4 (EC 2.7.10.1) (Tyrosine-protein kinase receptor MPK-3) (Tyrosine-protein kinase receptor SEK-1)	Epha4 Sek Sek1	adherens junction organization [GO:0034332]; adult walking behavior [GO:0007628]; axon guidance [GO:0007411]; cell adhesion [GO:0007155]; cellular response to amyloid-beta [GO:1904646]; cochlea development [GO:0090102]; corticospinal tract morphogenesis [GO:0021957]; ephrin receptor signaling pathway [GO:0048013]; fasciculation of motor neuron axon [GO:0097156]; fasciculation of sensory neuron axon [GO:0097155]; glial cell migration [GO:0008347]; innervation [GO:0060384]; motor neuron axon guidance [GO:0008045]; negative regulation of axon regeneration [GO:0048681]; negative regulation of cell adhesion [GO:0007162]; negative regulation of cell migration [GO:0030336]; negative regulation of cellular response to hypoxia [GO:1900038]; negative regulation of epithelial to mesenchymal transition [GO:0010719]; negative regulation of ERK1 and ERK2 cascade [GO:0070373];

35793.12		15581.68	26238.23	16953	13465.77	24183.97	209.62	37579.85	31498.28	37216.83	7301.47	3181.8	6913.92	10613.06	3105.83	1201.21	11230.85	5588.68	287857.17	Dedicator of cytokinesis protein 2	DOCK2 KIAA0209	actin cytoskeleton organization [GO:0030036]; alpha-beta T cell proliferation [GO:0046633]; cell migration [GO:0016477]; chemotaxis [GO:0006935]; establishment of T cell polarity [GO:0001768]; immunological synapse formation [GO:0001771]; macropinocytosis [GO:0044351]; membrane raft polarization [GO:0001766]; myeloid dendritic cell activation involved in immune response [GO:0002277]; myoblast fusion [GO:0007520]; negative thymic T cell selection [GO:0045060]; positive regulation of phagocytosis [GO:0050766]; positive thymic T cell selection [GO:0045059]; regulation of small GTPase mediated signal transduction [GO:0051056]; small GTPase-mediated signal transduction [GO:0007264]
10250.2		16905.24	14107.92	8973.67	20668.03	12535.47	41635.1	8404.35	3906.19	6576.67	11215.86	12537.05	14729.88	28300.72	6520.07	36666.34	17174.8	4849.71	275957.27	Retinal dehydrogenase 2 (RALDH 2) (RalDH2) (EC 1.2.1.36) (Aldehyde dehydrogenase family 1 member A2) (ALDH1A2) (Retinaldehyde-specific dehydrogenase type 2) (RALDH(II))	ALDH1A2 RALDH2	heart looping [GO:0001947]; heart morphogenesis [GO:0003007]; positive regulation of gene expression [GO:0010628]; protein homotetramerization [GO:0051289]; response to retinoic acid [GO:0032526]; retinoic acid biosynthetic process [GO:0002138]; retinoic acid metabolic process [GO:0042573]; retinol metabolic process [GO:0042572]; spinal cord motor neuron differentiation [GO:0021522]

13723.53	13283.7	15056.64	11760.6	19082.97	21761.15	24165.56	5229.64	6037.08	7133.45	8355.93	17580.39	20399.69	29922.72	7666.65	23507.67	20246.93	9280.85	274195.15	BCL2/adenovirus E1B 19 kDa protein-interacting protein 3-like (Adenovirus E1B19K-binding protein B5) (BCL2/adenovirus E1B 19 kDa protein-interacting protein 3A) (NIP3-like protein X) (NIP3L)	BNIP3L BNIP3A BNIP3H NIX	cellular response to hypoxia [GO:0071456]; defense response to virus [GO:0051607]; mitochondrial outer membrane permeabilization [GO:0097345]; mitochondrial protein catabolic process [GO:0035694]; negative regulation of apoptotic process [GO:0043066]; negative regulation of mitochondrial membrane potential [GO:0010917]; negative regulation of programmed cell death [GO:0043069]; positive regulation of apoptotic process [GO:0043065]; positive regulation of macroautophagy [GO:0016239]; regulation of mitophagy [GO:1901524]; regulation of programmed cell death [GO:0043067]; regulation of protein targeting to mitochondrion [GO:1903214]
62624.26	421.11	300.39	12575.19	1305.33	3125.76	176.84	91.24	1.69	44861.05	1075.02	2708.43	137038.48	225.6	438.41	3742.69	511.78	47.08	271270.35	Tripartite motif-containing protein 16 (EC 2.3.2.27) (E3 ubiquitin-protein ligase TRIM16) (Estrogen-responsive B box protein)	TRIM16 EBBP	positive regulation of DNA-templated transcription [GO:0045893]; positive regulation of interleukin-1 beta production [GO:0032731]; positive regulation of keratinocyte differentiation [GO:0045618]; positive regulation of retinoic acid receptor signaling pathway [GO:0048386]; response to growth hormone [GO:0060416]; response to retinoic acid [GO:0032526]
69201.46	617.2	344.77	14923.83	4055.06	8470.63	129.92	304.53	9.63	30302	2244.1	1904.95	125693.15	319.05	428.81	4232.38	743.33	22.22	263947.02	NADH dehydrogenase [ubiquinone] 1 alpha subcomplex subunit 11 (Complex I-B14.7) (CI-B14.7) (NADH-ubiquinone oxidoreductase subunit B14.7)	NDUFA11	mitochondrial electron transport, NADH to ubiquinone [GO:0006120]

11575.38	12305.18	13632.98	8617.53	16218.03	23703.77	20374.88	6573.8	6006.83	5787.07	8215.14	15966.81	21447.84	27852.47	9203.05	20162.86	15244.21	7974.75	250862.58	Sialidase-1 (EC 3.2.1.18) (Acetylneuraminyl hydrolase) (Lysosomal sialidase) (N-acetyl-alpha-neuraminidase 1)	NEU1	ganglioside catabolic process [GO:0006689]; oligosaccharide catabolic process [GO:0009313]
10635.66	20874.03	16024.13	8788.44	9637.72	43643.84	124.66	22166.21	9541.56	9379.01	6135.69	20480.72	12832.68	8306.28	9383.75	3111.08	14603.78	10353.42	236022.66	Leukocyte antigen CD37 (Tetraspanin-26) (Tspan-26) (CD antigen CD37)	CD37 TSPAN26	defense response to protozoan [GO:0042832]; negative regulation of myeloid dendritic cell activation [GO:0030886]; negative regulation of T cell proliferation [GO:0042130]; positive regulation of immunoglobulin production [GO:0002639]; regulation of defense response to virus [GO:0050688]; regulation of humoral immune response [GO:0002920]; T cell proliferation [GO:0042098]
7.87	72009.1	23.49	6.91	36714.15	125.19	0.22	22718.53	22.36	4.52	14929.32	16.98	25.26	47700.4	43.97	4.67	31537.82	96.2	225986.96	N-myc-interactor (Nmi) (N-myc and STAT interactor)	NMI	cell surface receptor signaling pathway via JAK-STAT [GO:0007259]; innate immune response [GO:0045087]; macrophage activation involved in immune response [GO:0002281]; negative regulation of cell population proliferation [GO:0008285]; negative regulation of innate immune response [GO:0045824]; negative regulation of interferon-alpha production [GO:0032687]; negative regulation of interferon-beta production [GO:0032688]; negative regulation of non-canonical NF-kappaB signal transduction [GO:1901223]; positive regulation of inflammatory response [GO:0050729]; positive regulation of innate immune response [GO:0045089]; positive regulation of non-canonical NF-kappaB signal transduction [GO:1901224]; positive regulation of protein K48-linked ubiquitination [GO:1902524]; protein K48-linked ubiquitination [GO:0070936]; response to virus [GO:0009615]; toll-like receptor 4 signaling pathway [GO:0034142]

1160.03	4.97	0	3460.11	0	94.45	144058.5	6.87	5.49	1147.52	3219.8	7.56	146.8	0	0	64446.61	2339.43	63.46	220161.6	Secretogranin-2 (Chromogranin-C) (Secretogranin II) (SgII) [Cleaved into: Secretoneurin (SN);Manserin]	SCG2 CHGC	angiogenesis [GO:0001525]; eosinophil chemotaxis [GO:0048245]; induction of positive chemotaxis [GO:0050930]; intracellular signal transduction [GO:0035556]; MAPK cascade [GO:0000165]; negative regulation of endothelial cell apoptotic process [GO:2000352]; negative regulation of extrinsic apoptotic signaling pathway [GO:2001237]; positive chemotaxis [GO:0050918]; positive regulation of endothelial cell proliferation [GO:0001938]
561.64	4.64	5.9	9030.59	8.49	172.25	114392.87	3.08	4.81	1165.99	2018.9	22.98	164.12	39.98	5.76	86299.09	2431.43	67.47	216399.99	Exosome complex component RRP43 (Exosome component 8) (Opa-interacting protein 2) (OIP-2) (Ribosomal RNA-processing protein 43) (p9)	EXOSC8 OIP2 RRP43	exonucleolytic trimming to generate mature 3'-end of 5.8S rRNA from tricistronic rRNA transcript (SSU-rRNA, 5.8S rRNA, LSU-rRNA) [GO:0000467]; nuclear mRNA surveillance [GO:0071028]; nuclear polyadenylation-dependent rRNA catabolic process [GO:0071035]; RNA catabolic process [GO:0006401]; RNA processing [GO:0006396]; rRNA catabolic process [GO:0016075]; TRAMP-dependent tRNA surveillance pathway [GO:0071038]; U1 snRNA 3'-end processing [GO:0034473]; U4 snRNA 3'-end processing [GO:0034475]; U5 snRNA 3'-end processing [GO:0034476]

13539.44	12970.11	23134.41	17680.12	19363.8	16096.13	1741.28	9779.45	5228.46	8554.36	9400.64	16160.26	9115.55	15588.93	7413.27	6094.08	13780.17	7206.85	212847.31	Coronin-1C (Coronin-3) (hCRNN4)	CORO1C CRN2 CRNN4	actin filament organization [GO:0007015]; activation of GTPase activity [GO:0090630]; corpus callosum development [GO:0022038]; endosomal transport [GO:0016197]; endosome fission [GO:0140285]; endosome membrane tubulation [GO:0097750]; establishment of protein localization [GO:0045184]; membrane fission [GO:0090148]; negative regulation of epithelial cell migration [GO:0010633]; negative regulation of focal adhesion assembly [GO:0051895]; negative regulation of protein kinase activity by regulation of protein phosphorylation [GO:0044387]; negative regulation of protein phosphorylation [GO:0001933]; negative regulation of substrate adhesion-dependent cell spreading [GO:1900025]; neural crest cell migration [GO:0001755]; phagocytosis [GO:0006909]; positive regulation of lamellipodium morphogenesis [GO:2000394]; regulation of epithelial cell migration [GO:0010632]; regulation of fibroblast migration [GO:0010762];
9772.55	8151.35	10689.8	9027.91	10507.61	17101.31	12387.19	5057.59	6272.47	15052.77	7325.95	26654.33	15956.85	9547.95	16749.11	13300.19	8181.69	10206.38	211943	Bcl-2-like protein 11 (Bcl2-L-11) (Bcl-2-related ovarian death protein) (Bcl2-interacting mediator of cell death)	Bcl2l11 Bim Bod	apoptotic process involved in embryonic digit morphogenesis [GO:1902263]; B cell homeostasis [GO:0001782]; cell-matrix adhesion [GO:0007160]; cellular response to amyloid-beta [GO:1904646]; cellular response to estradiol stimulus [GO:0071392]; cellular response to nerve growth factor stimulus [GO:1990090]; developmental pigmentation [GO:0048066]; ear development [GO:0043583]; extrinsic apoptotic signaling pathway in absence of ligand [GO:0097192]; in utero embryonic development [GO:0001701]; intrinsic apoptotic signaling pathway in response to DNA damage [GO:0008630]; kidney development [GO:0001822]; leukocyte homeostasis [GO:0001776]; lymphocyte apoptotic process [GO:0070227]; lymphocyte homeostasis [GO:0002260]; male gonad development [GO:0008584]; mammary gland development [GO:0030879]; meiosis I [GO:0007127]; myeloid cell homeostasis [GO:0002262];
7759.07	5409.11	8049.52	7637.21	9769.67	28367.27	10359.42	5429.73	5448.85	12750.69	7831.23	9759.1	9746.7	19927.75	11641.88	22755.94	10110.83	11499.89	204253.86	Protein-methionine sulfoxide oxidase mical3b (EC 1.14.13.225) (Molecule interacting with CasL protein 3B) (MICAL-3B)	mical3b	actin filament depolymerization [GO:0030042]; cytoskeleton organization [GO:0007010]; exocytosis [GO:0006887]

294.85	0	0	941.17	0	63.15	164025.9	0	2.69	310.7	1273.17	0	43.75	0	0	34190.78	688.56	30.28	201865	Peroxisomal membrane protein PMP34 (34 kDa peroxisomal membrane protein) (Solute carrier family 25 member 17)	SLC25A17 PMP34	ATP transport [GO:0015867]; fatty acid alpha-oxidation [GO:0001561]; fatty acid beta-oxidation [GO:0006635]; fatty acid transport [GO:0015908]; nucleotide transmembrane transport [GO:1901679]
8713.05	15841.11	5802.24	11300.47	14938.52	28042.64	760.03	16301.94	6131.81	14225.15	16038.28	10094.83	6896.88	23028.77	6159.11	2625.46	6780.56	5227.77	198908.62	Inward rectifier potassium channel 2 (BIK) (Inward rectifier K(+) channel Kir2.1) (IRK-1) (Potassium channel, inwardly rectifying subfamily J member 2)	KCNJ2 IRK1	cardiac muscle cell action potential involved in contraction [GO:0086002]; magnesium ion transport [GO:0015693]; potassium ion import across plasma membrane [GO:1990573]; potassium ion transport [GO:0006813]; protein homotetramerization [GO:0051289]; regulation of heart rate by cardiac conduction [GO:0086091]; regulation of membrane repolarization [GO:0060306]; regulation of monoatomic ion transmembrane transport [GO:0034765]; regulation of skeletal muscle contraction via regulation of action potential [GO:0014861]; relaxation of cardiac muscle [GO:0055119]; relaxation of skeletal muscle [GO:0090076]
21530.71	20165.89	0	27998.52	13656.16	190.94	145.17	19958.58	0	23986.22	27757.05	21.46	10586.49	12820.09	20.83	5611.75	14199.64	54.22	198703.72	Glycylpeptide N-tetradecanoyltransferase 2 (EC 2.3.1.97) (Myristoyl-CoA:protein N-myristoyltransferase 2) (NMT 2) (Peptide N-myristoyltransferase 2) (Type II N-myristoyltransferase)	nmt2	N-terminal peptidyl-glycine N-myristoylation [GO:0018008]; protein localization to membrane [GO:0072657]

1540.93		40847.28	9693.3	184.89	44136.59	15890.91	134.07	16498.81	643.01	677.56	6982.46	5513.39	613.75	35431.42	857.72	578.85	16873.89	757.57	197856.4	Nucleosome-remodeling factor subunit BPTF (Bromodomain and PHD finger-containing transcription factor) (Fetal Alz-50 clone 1 protein) (Fetal Alzheimer antigen)	BPTF FAC1 FALZ	anterior/posterior pattern specification [GO:0009952]; brain development [GO:0007420]; cellular response to nerve growth factor stimulus [GO:1990090]; chromatin remodeling [GO:0006338]; embryonic placenta development [GO:0001892]; endoderm development [GO:0007492]; negative regulation of transcription by RNA polymerase II [GO:0000122]; positive regulation of transcription by RNA polymerase II [GO:0045944]; regulation of DNA-templated transcription [GO:0006355]; regulation of transcription by RNA polymerase II [GO:0006357]
9971.97		9593.77	20376.39	5262.64	7318.68	15460.78	11722.75	1277.06	2410.93	6561.8	6287.73	17240.5	19751.79	24616.62	8939.88	8733.62	8085.96	5728.08	189340.95	Microtubule-associated serine/threonine-protein kinase 1 (EC 2.7.11.1) (Syntrophin-associated serine/threonine-protein kinase)	Mast1 Sast	brain development [GO:0007420]; cytoskeleton organization [GO:0007010]; intracellular signal transduction [GO:0035556]; protein phosphorylation [GO:0006468]

0	53751.07	0	4.15	14273.16	60.48	0	25221.95	0	0.38	9668.01	1.63	1.68	37120.2	0	2.6	47457.36	190.3	187752.97	Potassium voltage-gated channel subfamily KQT member 2 (KQT-like 2) (Potassium channel subunit alpha KvLQT2) (Voltage-gated potassium channel subunit Kv7.2)	Kcnq2 Kqt2	action potential [GO:0001508]; action potential initiation [GO:0099610]; apoptosome assembly [GO:0097314]; brain development [GO:0007420]; cellular response to calcium ion [GO:0071277]; cellular response to xenobiotic stimulus [GO:0071466]; cognition [GO:0050890]; dentate gyrus development [GO:0021542]; determination of adult lifespan [GO:0008340]; endocytosis [GO:0006897]; establishment of cell polarity [GO:0030010]; excitatory chemical synaptic transmission [GO:0098976]; exocytosis [GO:0006887]; exploration behavior [GO:0035640]; gene expression [GO:0010467]; grooming behavior [GO:0007625]; hippocampal pyramidal neuron differentiation [GO:0097432]; hippocampus development [GO:0021766]; inhibitory chemical synaptic transmission [GO:0098977]; learning [GO:0007612]; memory [GO:0007613]; mitochondrial depolarization [GO:0051882];
7321.45	12132.91	6484.89	6929.43	14718.01	12371.48	47105.56	6542.52	2407.2	3906.01	6196.62	5048.71	8256.81	16778.11	2945.47	16623.3	8550.71	2782.57	187101.76	Metabotropic glycine receptor (mGlyR) (G-protein coupled receptor 158)	Gpr158	brain development [GO:0007420]; cognition [GO:0050890]; G protein-coupled receptor signaling pathway [GO:0007186]; positive regulation of neurotransmitter secretion [GO:0001956]; protein localization to plasma membrane [GO:0072659]; regulation of G protein-coupled receptor signaling pathway [GO:0008277]; regulation of synapse organization [GO:0050807]

633.36	7553.21	2289.83	2512.41	20801.75	1685.11	28127.82	11517.87	16.91	716.46	5974.21	33656.37	328.23	29100.63	310.75	20012.93	16379.18	162.28	181779.31	Mismatch repair endonuclease PMS2 (EC 3.1.-.-) (DNA mismatch repair protein PMS2) (PMS1 protein homolog 2)	PMS2 PMSL2	mismatch repair [GO:0006298]; positive regulation of isotype switching to IgA isotypes [GO:0048298]; positive regulation of isotype switching to IgG isotypes [GO:0048304]; response to xenobiotic stimulus [GO:0009410]; somatic hypermutation of immunoglobulin genes [GO:0016446]; somatic recombination of immunoglobulin gene segments [GO:0016447]
3421.58	5833.38	0	26145.31	14145.25	140.99	110.07	14930.12	0	20261.65	30968.03	0	6551.63	10797.79	0	14389.43	32609.39	278.6	180583.22	EF-hand domain-containing protein D2 (Swiprosin-1)	EFHD2 SWS1	
11.06	84687.17	2.89	244.65	10652.1	34.47	0.45	16358.52	0	175.78	5591.79	2	4.79	44267.37	1.29	2.09	17606.8	30.33	179673.55	Transcription initiation factor TFIID subunit 2 (TBP-associated factor 150 kDa) (Transcription initiation factor TFIID 150 kDa subunit) (TAF(II)150) (TAFII-150) (TAFII150)	taf2	transcription initiation at RNA polymerase II promoter [GO:0006367]

239.78	32.73	10204.4	89.78	3.38	4963.39	0.5	2.66	55.26	70.22	8.42	161352.68	513.66	2.99	782.81	15.12	10.48	359.26	178707.52	Peroxisomal membrane protein PEX14 (PTS1 receptor-docking protein) (Peroxin-14) (Peroxisomal membrane anchor protein PEX14)	Pex14	cellular response to reactive oxygen species [GO:0034614]; microtubule anchoring [GO:0034453]; peroxisome transport along microtubule [GO:0036250]; protein import into peroxisome matrix, docking [GO:0016560]; protein import into peroxisome matrix, substrate release [GO:0044721]; protein import into peroxisome matrix, translocation [GO:0016561]; protein-containing complex assembly [GO:0065003]
37440.75	272.84	276.69	9089.22	1469.34	7159.96	162.8	100.87	4.77	35451.69	970.53	1824.03	76019.8	162.81	331.36	1893.29	325.52	17.26	172973.53	26S proteasome regulatory subunit 6A (26S proteasome AAA-ATPase subunit RPT5) (Proteasome 26S subunit ATPase 3) (Spermatogenic cell/sperm-associated Tat-binding protein homolog SATA) (Tat-binding protein 1) (TBP-1)	Psmc3 Tbp1	blastocyst development [GO:0001824]; modulation by host of viral transcription [GO:0043921]; positive regulation of transcription by RNA polymerase II [GO:0045944]; proteasome-mediated ubiquitin-dependent protein catabolic process [GO:0043161]

4540.6	6354.63	8516.53	5380.27	6041.8	8130.98	42587.88	2457.53	2766.09	2597.61	3227.68	7986.18	7138.66	17106.85	3746.37	28541.34	10519.12	4027.36	171667.48	Activity-regulated cytoskeleton-associated protein (hArc) (Activity-regulated gene 3.1 protein homolog) (ARC/ARG3.1) (Arg3.1)	ARC KIAA0278	anterior/posterior pattern specification [GO:0009952]; cell migration [GO:0016477]; cytoskeleton organization [GO:0007010]; dendritic spine morphogenesis [GO:0060997]; endocytosis [GO:0006897]; endoderm development [GO:0007492]; long-term memory [GO:0007616]; long-term synaptic potentiation [GO:0060291]; modulation of chemical synaptic transmission [GO:0050804]; mRNA transport [GO:0051028]; protein homooligomerization [GO:0051260]; regulation of cell morphogenesis [GO:0022604]; regulation of dendritic spine morphogenesis [GO:0061001]; regulation of long-term synaptic depression [GO:1900452]; regulation of long-term synaptic potentiation [GO:1900271]; regulation of neuronal synaptic plasticity [GO:0048168]; regulation of postsynaptic neurotransmitter receptor internalization [GO:0099149]; vesicle-mediated intercellular transport [GO:0110077]
3718.61	8252.91	7471.32	6362.35	11662.97	12002.25	25020.64	5756.09	3430.16	3757.27	5976.02	6554.83	7240.19	17491.49	4844.67	23967.08	10676.89	2868.66	167054.4	Immediate early response gene 2 protein (Protein ETR101)	IER2 ETR101 PIP92	cell motility [GO:0048870]; neuron differentiation [GO:0030182]; positive regulation of transcription by RNA polymerase II [GO:0045944]; response to fibroblast growth factor [GO:0071774]

420.5	486.88	27653.55	276.96	214.12	52737.51	34.76	219.87	622.23	1024.55	832.48	70601.95	270.97	378.38	7959.09	939.82	368.65	771.72	165813.99	Dihydrolipoyllysine-residue succinyltransferase component of 2-oxoglutarate dehydrogenase complex, mitochondrial (EC 2.3.1.61) (2-oxoglutarate dehydrogenase complex component E2) (OGDC-E2) (Dihydrolipoamide succinyltransferase component of 2-oxoglutarate dehydrogenase complex) (E2K)	dlst	2-oxoglutarate metabolic process [GO:0006103]; L-lysine catabolic process to acetyl-CoA via saccharopine [GO:0033512]; succinyl-CoA metabolic process [GO:0006104]; tricarboxylic acid cycle [GO:0006099]
8809.37	8025.54	8619.89	7215.68	8516.51	15337	12111.43	2806.82	3359.36	5147.11	5173.19	12795.92	12954.57	16716.12	5105.12	12050.64	10261.87	4724.47	159730.61	GDNF family receptor alpha-4 (GDNF receptor alpha-4) (GDNFR-alpha-4) (GFR-alpha-4)	GFRA4	glial cell-derived neurotrophic factor receptor signaling pathway [GO:0035860]; nervous system development [GO:0007399]
7847.49	7831.37	11697.75	2971.88	14345.28	19640.92	55.12	6566.38	1120.45	3841.71	5066.01	6263.86	23923.11	18125.07	11921.23	1692.88	8838.52	6600.94	158349.97	Intermembrane lipid transfer protein VPS13B (Cohen syndrome protein 1) (Vacuolar protein sorting-associated protein 13B)	VPS13B CHS1 COH1 KIAA0532	acrosome assembly [GO:0001675]; adipose tissue development [GO:0060612]; central nervous system development [GO:0007417]; dentate gyrus development [GO:0021542]; Golgi organization [GO:0007030]; Golgi reassembly [GO:0090168]; head morphogenesis [GO:0060323]; lipid transport [GO:0006869]; maintenance of lens transparency [GO:0036438]; memory [GO:0007613]; multicellular organism growth [GO:0035264]; muscle organ development [GO:0007517]; nervous system development [GO:0007399]; neuron projection development [GO:0031175]; slow endocytic recycling [GO:0032458]; social behavior [GO:0035176]; vesicle-mediated transport [GO:0016192]

305.09	0	0	4705.98	0	100.2	111199.19	0	0	428.26	1326.06	0	41.4	0	4.35	38751.64	848.25	5.34	157715.76	Centrosomal protein of 44 kDa (Cep44)	cep44	centriole replication [GO:0007099]; centriole-centriole cohesion [GO:0010457]; centrosome cycle [GO:0007098]
0	19628.17	0	11.45	36534.32	230.16	0	16348.73	0	5.5	9679.05	0	0	54505.83	0	0	16074.52	56.32	153074.05	Neurabin-2 (Neurabin-II) (Protein phosphatase 1 regulatory subunit 9B) (Spinophilin)	PPP1R9B PPP1R6	actin filament depolymerization [GO:0030042]; actin filament organization [GO:0007015]; calcium-mediated signaling [GO:0019722]; cell migration [GO:0016477]; cellular response to epidermal growth factor stimulus [GO:0071364]; cellular response to estradiol stimulus [GO:0071392]; cellular response to morphine [GO:0071315]; cellular response to peptide [GO:1901653]; cellular response to xenobiotic stimulus [GO:0071466]; cerebral cortex development [GO:0021987]; dendrite development [GO:0016358]; developmental process involved in reproduction [GO:0003006]; filopodium assembly [GO:0046847]; hippocampus development [GO:0021766]; learning [GO:0007612]; male mating behavior [GO:0060179]; negative regulation of cell growth [GO:0030308]; neuron projection development [GO:0031175]; positive regulation of protein localization to actin cortical patch [illegible]
34732.03	277.79	234.54	6066.06	887.6	2616.96	74.15	65.44	0	19186.18	708.67	1202.11	83986.2	206.58	260.84	1808.71	324.58	16.66	152655.1	Dixin-A (Coiled-coil protein DIX1-A) (Coiled-coil-DIX1-A) (DIX domain-containing protein 1-A)	dixdc1a ccd1 dixdc1	camera-type eye development [GO:0043010]; canonical Wnt signaling pathway [GO:0060070]; forebrain development [GO:0030900]

118.97	0	0	1884.31	0	69.92	100441.18	0	0	469.61	1192.51	0	30.76	0	0	42461.72	1117.19	52.71	147838.88	Collectin-12 (Collectin placenta protein 1) (CL-P1) (Nurse cell scavenger receptor 2)	Colec12 Clp1 Nsr2	cellular response to exogenous dsRNA [GO:0071360]; defense response to bacterium [GO:0042742]; immune response [GO:0006955]; phagocytosis, recognition [GO:0006910]; plasma membrane raft organization [GO:0044857]; toll-like receptor 3 signaling pathway [GO:0034138]
897.99	627.93	311.25	1838.32	629.48	915.97	77748.53	160.9	120.58	1066.78	1730.58	233.43	3737.61	716.48	590.86	54358.18	1603.15	282.19	147570.21	Protein Aster-B (GRAM domain-containing protein 1B)	GRAMD1B KIAA1201 UNQ3032/PRO9834	cellular response to cholesterol [GO:0071397]; cholesterol homeostasis [GO:0042632]; intracellular sterol transport [GO:0032366]
119.26	3.59	19.45	914.61	3.79	62.9	103940.76	0	0	583.66	1204.11	57.37	22.9	4.29	8.68	38359.76	780.53	47.49	146133.15	Mitogen-activated protein kinase-binding protein 1 (JNK-binding protein 1) (JNKBP-1)	MAPKBP1 JNKBP1 KIAA0596	negative regulation of canonical NF-kappaB signal transduction [GO:0043124]; negative regulation of defense response to bacterium [GO:1900425]; negative regulation of interleukin-8 production [GO:0032717]; positive regulation of JNK cascade [GO:0046330]

5264.08	4443.26	4799.07	3707.05	4309.01	10311.73	29.22	71147.62	10018.04	4264.36	5328.3	1263.97	1779.42	9922.26	1606.35	537	3486.8	2162.03	144379.57	Y+L amino acid transporter 1 (Monocyte amino acid permease 2) (MOP-2) (Solute carrier family 7 member 7) (y(+)L-type amino acid transporter 1) (Y+LAT1) (y+LAT-1)	SLC7A7	amino acid transmembrane transport [GO:0003333]; basic amino acid transmembrane transport [GO:1990822]; L-arginine transmembrane transport [GO:1903826]; L-leucine transport [GO:0015820]; regulation of arginine metabolic process [GO:0000821]
9214.37	14775	10673.02	2793.44	16485.84	20125.96	76.61	5237.93	626.52	1936.12	5600.04	6630.22	7871.71	19875.89	6582.05	1673.11	7836.3	4816.21	142830.34	Vasoactive intestinal polypeptide receptor 2 (VIP-R-2) (Pituitary adenylate cyclase-activating polypeptide type III receptor) (PACAP type III receptor) (PACAP-R-3) (PACAP-R3)	Vipr2	adenylate cyclase-activating G protein-coupled receptor signaling pathway [GO:0007189]; adenylate cyclase-modulating G protein-coupled receptor signaling pathway [GO:0007188]; cell surface receptor signaling pathway [GO:0007166]; negative regulation of smooth muscle cell proliferation [GO:0048662]

12507.29	7561.08	6948.51	6006.73	11712.51	10849.59	17209.39	4381.3	3478.31	4906.62	4494.42	5045.73	8355.07	12784.57	2453.95	13646.93	6942	3022.92	142306.92	FACT complex subunit SPT16 (Chromatin-specific transcription elongation factor 140 kDa subunit) (FACT 140 kDa subunit) (FACTp140) (Facilitates chromatin transcription complex subunit SPT16)	Supt16h Fact140 Factp140 Supt16	DNA repair [GO:0006281]; DNA replication [GO:0006260]; nucleosome assembly [GO:0006334]; nucleosome disassembly [GO:0006337]
6423.65	11939.6	15447.46	2031.27	10835.97	14727.01	496.82	11248.19	1376.13	2019.14	5109.7	8328.89	12120.65	16713.2	5993.38	1527.38	11032.12	4100.92	141471.48	Androgen-induced gene 1 protein (AIG-1) (Fatty acid esters of hydroxy fatty acids hydrolase AIG1) (FAHFA hydrolase AIG1) (EC 3.1.-.-)	AIG1 CGI-103	long-chain fatty acid catabolic process [GO:0042758]
36406.51	295.12	117.51	6778.04	923.75	2045.08	77.73	119.07	3.06	22923.94	843.69	1156.38	66292.2	139.14	158.42	1622.45	264.15	30.67	140196.91	SEC14 domain and spectrin repeat-containing protein 1 (Protein Solo)	sestd1 sol solo si:dz106a20.1 si:dz42h5.2	blood vessel morphogenesis [GO:0048514]
3305.5	1545.84	3261.51	2650.96	1737.4	5533.36	51164.88	1072.72	5095.18	1948.31	1546.1	3500.64	8672.51	2265.52	4406.86	33448.2	3117.67	4985.25	139258.41	LIM domain-binding protein 3 (Protein cypher) (Protein oracle) (Z-band alternatively spliced PDZ-motif protein)	Ldb3 Kiaa0613	sarcomere organization [GO:0045214]

13823.23	11780.55	8068.18	7141.56	9390.69	16274.44	136	6258.6	11800.31	12622.51	12220.97	9670.16	2842.68	5292.27	6191.57	150.95	750.93	2342.72	136758.32	Integrin alpha-7 (H36-alpha7) [Cleaved into: Integrin alpha-7 heavy chain;Integrin alpha-7 light chain;Integrin alpha-7 70 kDa form]	Itga7	blood vessel morphogenesis [GO:0048514]; cell adhesion [GO:0007155]; cell adhesion mediated by integrin [GO:0033627]; cell migration [GO:0016477]; cell-cell adhesion [GO:0098609]; endodermal cell differentiation [GO:0035987]; heterotypic cell-cell adhesion [GO:0034113]; integrin-mediated signaling pathway [GO:0007229]; leukocyte migration [GO:0050900]; regulation of cell shape [GO:0008360]; skeletal muscle tissue development [GO:0007519]
30330.28	255.87	141.28	5749.25	1066.42	1491.99	67.93	98.53	3.31	17070.6	668.3	1011.85	74633.7	130.05	185.82	2157.41	363.26	17.71	135443.56	High affinity cGMP-specific 3',5'-cyclic phosphodiesterase 9A (EC 3.1.4.35)	Pde9a	cAMP-mediated signaling [GO:0019933]; cGMP catabolic process [GO:0046069]; cGMP metabolic process [GO:0046068]; negative regulation of neural precursor cell proliferation [GO:2000178]; positive regulation of cardiac muscle hypertrophy [GO:0010613]; positive regulation of long-term synaptic potentiation [GO:1900273]

33142.19	261.41	140.4	6149.95	656.81	1108.94	79.51	57.87	0.96	28794.37	588.37	1218.06	60653.06	104.32	105.58	1607.5	251.42	16.12	134936.84	Sodium/hydrogen exchanger 6 (Na(+)/H(+) exchanger 6) (NHE-6) (Sodium/hydrogen exchanger) (Solute carrier family 9 member 6)	Slc9a6	axon extension [GO:0048675]; brain-derived neurotrophic factor receptor signaling pathway [GO:0031547]; dendrite extension [GO:0097484]; dendritic spine development [GO:0060996]; establishment of cell polarity [GO:0030010]; glial cell activation [GO:0061900]; neuron projection morphogenesis [GO:0048812]; potassium ion transmembrane transport [GO:0071805]; proton transmembrane transport [GO:1902600]; regulation of intracellular pH [GO:0051453]; regulation of neurotrophin TRK receptor signaling pathway [GO:0051386]; regulation of postsynaptic membrane neurotransmitter receptor levels [GO:0099072]; regulation of synaptic vesicle lumen acidification [GO:1901546]; sodium ion import across plasma membrane [GO:0098719]; synapse organization [GO:0050808]
4391.63	5926.03	6850.55	5130.31	8543.07	6922.81	20832.55	3311.11	7023.06	2416.85	4057.15	5375.76	5880.94	12226.27	5637.61	13756.82	7446.41	6995.88	132724.81	Transmembrane protein 101	TMEM101	
48412.44	1406.11	10793.09	9409.36	1799.48	20543.36	2014.44	890.03	2703.78	8917.91	740.17	4745.39	7225.03	1110.9	2454.71	2790.59	1793.34	4539.77	132289.9	Dapper homolog 2 (Frodo 2)	dact2 dpr2 frd2 zgc:152832	mesoderm morphogenesis [GO:0048332]; negative regulation of nodal signaling pathway [GO:1900108]; Wnt signaling pathway [GO:0016055]

102.47	60270.24	22.87	261.93	28905.15	162.83	1.11	13427.36	6.59	12.66	2717.54	0	33.58	20589.13	4.93	5.87	5452.84	40.27	132017.37	Cadherin-related family member 2	Cdhr2	cell-cell adhesion mediated by cadherin [GO:0044331]; epithelial cell differentiation [GO:0030855]; homophilic cell adhesion via plasma membrane adhesion molecules [GO:0007156]; intermicrovillar adhesion [GO:0090675]; negative regulation of cell growth involved in contact inhibition [GO:0060243]; regulation of microvillus length [GO:0032532]
207.99	0	0	2949.51	0	29.04	83448.17	0	0	440.78	1063.2	0	0	0	0	42797.51	935	0	131871.2	Transient receptor potential cation channel subfamily A member 1 (Ankyrin-like with transmembrane domains protein 1) (Transformation-sensitive protein p120) (p120) (Wasabi receptor)	TRPA1 ANKTM1	calcium ion transmembrane import into cytosol [GO:0097553]; calcium ion transmembrane transport [GO:0070588]; cell surface receptor signaling pathway [GO:0007166]; cellular response to caffeine [GO:0071313]; cellular response to carbon dioxide [GO:0071244]; cellular response to cold [GO:0070417]; cellular response to food [GO:0071240]; cellular response to heat [GO:0034605]; cellular response to hydrogen peroxide [GO:0070301]; cellular response to toxic substance [GO:0097237]; detection of chemical stimulus involved in sensory perception of pain [GO:0050968]; detection of mechanical stimulus involved in sensory perception of pain [GO:0050966]; intracellular calcium ion homeostasis [GO:0006874]; monoatomic ion transport [GO:0006811]; positive regulation of insulin secretion involved in cellular response to glucose stimulus [GO:0035774]; positive regulation of monoatomic anion transport [illegible]

10235.64	8950.65	6777.07	7687.92	9749.53	13635	38.77	6950.39	9995.83	10204.12	9931.42	12600.82	3655.04	3600.6	5266.12	536.74	2783.98	9175.49	131775.13	Nuclear receptor subfamily 1 group D member 1 (Rev-erbA-alpha) (V-erbA-related protein 1) (EAR-1)	Nr1d1	cell differentiation [GO:0030154]; cellular response to interleukin-1 [GO:0071347]; cellular response to lipopolysaccharide [GO:0071222]; cellular response to tumor necrosis factor [GO:0071356]; cholesterol homeostasis [GO:0042632]; circadian regulation of gene expression [GO:0032922]; circadian rhythm [GO:0007623]; circadian temperature homeostasis [GO:0060086]; glycogen biosynthetic process [GO:0005978]; hormone-mediated signaling pathway [GO:0009755]; intracellular glucose homeostasis [GO:0001678]; intracellular receptor signaling pathway [GO:0030522]; negative regulation of astrocyte activation [GO:0061889]; negative regulation of canonical NF-kappaB signal transduction [GO:0043124]; negative regulation of cold-induced thermogenesis [GO:0120163]; negative regulation of DNA-templated transcription [GO:0045892]; negative regulation of inflammatory response [GO:0050728];
5795.02	10843.2	3524.82	7636.47	10458.52	20495.68	503.69	9728.28	3053.39	9329.81	11661.69	6552.98	3826.93	11942.73	3347.42	1880.67	4067.8	2725.44	127374.54	TRIO and F-actin-binding protein (Protein Tara) (Trio-associated repeat on actin)	Triobp Kiaa1662 Tara	actin filament bundle assembly [GO:0051017]; auditory receptor cell stereocilium organization [GO:0060088]; cell division [GO:0051301]; positive regulation of substrate adhesion-dependent cell spreading [GO:1900026]; sensory perception of sound [GO:0007605]
32548.95	291.08	99.5	6455.29	844.07	2172.91	88.01	63.02	0	20021.05	674.63	1345.04	60447.15	163.41	144.68	1340.42	248.04	2.89	126950.14	RNA-binding protein PNO1	pno1	ribosome biogenesis [GO:0042254]

26124.58	229.81	152.69	6032.92	612.28	2376.43	76.98	49.52	0	22919.54	515.11	1332.7	58950.21	121.49	140.64	1588.1	182.26	8.29	121413.55	NMDA receptor synaptonuclear signaling and neuronal migration factor (Nasal embryonic luteinizing hormone-releasing hormone factor) (Nasal embryonic LHRH factor)	nsmf nelf nelfb	positive regulation of axon extension [GO:0045773]; positive regulation of neuron migration [GO:2001224]; regulation of neuronal synaptic plasticity [GO:0048168]
9544.12	4177.58	11220.12	2471.54	15183.77	13790.7	62.96	7572.6	739.15	3783.62	7688.33	11624.39	6483.19	9305.14	3074.81	2725.18	9950.04	1655.49	121052.73	Core histone macro-H2A.1 (Histone macroH2A1) (mH2A1) (H2A.y) (H2A/y)	Macroh2a1 H2afy	DNA repair [GO:0006281]; epigenetic regulation of gene expression [GO:0040029]; establishment of protein localization to chromatin [GO:0071169]; heterochromatin formation [GO:0031507]; negative regulation of cell cycle G2/M phase transition [GO:1902750]; negative regulation of gene expression, epigenetic [GO:0045814]; negative regulation of protein localization to chromosome, telomeric region [GO:1904815]; negative regulation of transcription by RNA polymerase II [GO:0000122]; negative regulation of transcription of nucleolar large rRNA by RNA polymerase I [GO:1901837]; nucleosome assembly [GO:0006334]; positive regulation of endodermal cell differentiation [GO:1903226]; positive regulation of keratinocyte differentiation [GO:0045618]; positive regulation of maintenance of mitotic sister chromatid cohesion [GO:0034184]; regulation of lipid [illegible]
205.13	0	0	452.97	0	4.84	68627.87	0	0	1008.64	1870.9	1.89	47.33	0	0	46851.35	1317	29.56	120417.48	E3 ubiquitin-protein ligase KCMF1 (EC 2.3.2.27)	kcmf1 si:ch211-220b11.7	negative regulation of HRI-mediated signaling [GO:0141191]; protein K63-linked ubiquitination [GO:0070534]; synaptic signaling [GO:0099536]

4770.12	5490.42	4608.59	4563.79	8234.69	9001.56	13618.86	3056.34	2711.24	4270.7	4627.02	8376.91	7512.74	12552.8	3217.42	10541.14	7913.88	3583.13	118651.35	Proline-serine-threonine phosphatase-interacting protein 2 (PEST phosphatase-interacting protein 2)	PSTPIP2	
8108.34	13653.02	8409.23	1625.16	17017.82	10801.29	187.43	3690.93	738.92	2950.53	8242.3	10954.42	6095.55	11968.69	2827.85	1501.38	7876	1948.9	118597.76	Phosphatidylinositol 3,4,5-trisphosphate-dependent Rac exchanger 2 protein (P-Rex2) (PtdIns(3,4,5)-dependent Rac exchanger 2) (DEP domain-containing protein 2)	PREX2 DEPDC2	adult locomotory behavior [GO:0008344]; dendrite morphogenesis [GO:0048813]; G protein-coupled receptor signaling pathway [GO:0007186]; negative regulation of TOR signaling [GO:0032007]; phosphatidylinositol 3-kinase/protein kinase B signal transduction [GO:0043491]; regulation of small GTPase mediated signal transduction [GO:0051056]
6050.13	5838.48	8460.75	4815.17	6690.05	11256.99	4873.26	2282.61	3588.76	3364.45	3763.57	10482.81	9284.61	12637.83	4083.92	7468.06	7901.27	4365.77	117208.49	4-galactosyl-N-acetylglucosaminide 3-alpha-L-fucosyltransferase 9 (EC 2.4.1.152) (Fucosyltransferase 9) (Fucosyltransferase IX) (Fuc-TIX) (FucT-IX) (Galactoside 3-L-fucosyltransferase)	Fut9	fucosylation [GO:0036065]; glycosphingolipid biosynthetic process [GO:0006688]; Lewis x epitope biosynthetic process [GO:0106402]; N-glycan fucosylation [GO:0036071]; neuron differentiation [GO:0030182]; neuronal stem cell division [GO:0036445]; oligosaccharide biosynthetic process [GO:0009312]; polysaccharide biosynthetic process [GO:0000271]; positive regulation of neuron projection development [GO:0010976]; protein glycosylation [GO:0006486]; protein N-linked glycosylation [GO:0006487]; protein O-linked glycosylation [GO:0006493]; regulation of leukocyte cell-cell adhesion [GO:1903037]; regulation of leukocyte tethering or rolling [GO:1903236]

3650.58	1245.06	1898.71	2189.73	762.92	1999	45802.35	495.29	3691.59	1306.95	1183.51	1620.9	4422.85	3385.83	2671.77	32723.59	2150.83	2956.43	114157.89	Ecto-ADP-ribosyltransferase 4 (EC 2.4.2.31) (ADP-ribosyltransferase C2 and C3 toxin-like 4) (ARTC4) (Mono(ADP-ribosyl)transferase 4) (NAD(P)(+)--arginine ADP-ribosyltransferase 4) (CD antigen CD297)	Art4	
405.43	150.56	247.47	1566.68	145.05	316.69	51411.74	58.4	215.32	492.77	1320.07	194.58	374.57	201.7	276.3	54973.38	1384.76	287.68	114023.15	Erythroid differentiation-related factor 1	EDRF1 C10orf137	positive regulation of DNA-templated transcription [GO:0045893]
3820.22	8157.78	5187.65	3827.21	11062.63	5824.31	10342.04	4763.61	2616.56	3741.96	6539.3	6009.81	3952.8	12751.12	2536.33	8291.71	9840.78	3878.22	113144.04	Uncharacterized protein K02A2.6	K02A2.6	DNA integration [GO:0015074]
23422.9	326.7	158.46	11149.24	1805.04	6706.04	166.82	412.75	17.25	24410.36	783.59	699.6	39222.64	165.83	214.87	2913.54	416.26	88.48	113080.37	Probable methyltransferase-like protein 25 (EC 2.1.1.-)	METTL25 C12orf26	methylation [GO:0032259]
120.71	0	0	635.19	0	3.06	61055.81	0	0	510.5	1339.18	0	10.62	0	0	46990.29	1205.09	29.55	111900	Nck-associated protein 5-like (NCKAP5-like) (Centrosomal protein of 169 kDa) (Cep169)	NCKAP5L CEP169 KIAA1602 FP1193	microtubule bundle formation [GO:0001578]; microtubule depolymerization [GO:0007019]

5016.76	3745.33	4945.83	4644.77	5017.03	11054.76	14103.28	1670.75	2879.71	3346.87	3132.83	7707.26	7075.63	8968.99	3552.93	13448.12	6136.27	3766.01	110213.13	ATP-binding cassette sub-family C member 8 (Sulfonylurea receptor 1)	Abcc8 Sur Sur1	action potential [GO:0001508]; cellular response to nutrient levels [GO:0031669]; female pregnancy [GO:0007565]; glutamate secretion, neurotransmission [GO:0061535]; intracellular glucose homeostasis [GO:0001678]; memory [GO:0007613]; negative regulation of angiogenesis [GO:0016525]; negative regulation of blood-brain barrier permeability [GO:1905604]; negative regulation of glial cell proliferation [GO:0060253]; negative regulation of insulin secretion [GO:0046676]; negative regulation of low-density lipoprotein particle clearance [GO:0010989]; negative regulation of neuroblast migration [GO:0061855]; negative regulation of neurogenesis [GO:0050768]; neuromuscular process [GO:0050905]; positive regulation of insulin secretion involved in cellular response to glucose stimulus [GO:0035774]; positive regulation of potassium ion transport [GO:0043268]; positive regulation of [illegible]
94.57	1.01	45.72	1356.03	0	40.34	83330.33	0	3.05	265.97	661.03	25.85	14.99	0	3.49	21558.52	461.44	6.57	107868.91	NAD-dependent protein deacylase sirtuin-6 (EC 2.3.1.-) (NAD-dependent protein deacetylase sirtuin-6) (EC 2.3.1.286) (Protein mono-ADP-ribosyltransferase sirtuin-6) (EC 2.4.2.-)	SIRT6	cardiac muscle cell differentiation [GO:0055007]; circadian regulation of gene expression [GO:0032922]; determination of adult lifespan [GO:0008340]; double-strand break repair [GO:0006302]; ketone biosynthetic process [GO:0042181]; negative regulation of gluconeogenesis [GO:0045721]; negative regulation of glycolytic process [GO:0045820]; negative regulation of protein import into nucleus [GO:0042308]; negative regulation of transcription by RNA polymerase II [GO:0000122]; negative regulation of transcription elongation by RNA polymerase II [GO:0034244]; pericentric heterochromatin formation [GO:0031508]; positive regulation of cold-induced thermogenesis [GO:0120162]; positive regulation of double-strand break repair [GO:2000781]; positive regulation of fat cell differentiation [GO:0045600]; positive regulation of insulin secretion [GO:0032024]; positive regulation of [illegible]
8447.03	5803.85	8686.98	4423.87	11347.97	12019.37	959.35	5493.47	1848.64	4409.08	4258.9	9691.62	5670.03	7538.88	2626.69	2544.38	6649.89	2825.71	105245.71	Spermatid perinuclear RNA-binding protein	STRBP	cell differentiation [GO:0030154]; spermatogenesis [GO:0007283]

0	34703.25	0	0	6644.64	0	0	7921.53	0	0	4268.15	0	0	36161.07	0	0	13000.05	92.85	102791.54	Transcription factor IIIB 90 kDa subunit (TFIIIB90) (mTFIIIB90) (B-related factor 1) (BRF-1)	Brf1	regulation of mRNA stability [GO:0043488]; transcription preinitiation complex assembly [GO:0070897]
10857.91	4666.7	9630.43	3295.54	4083.01	7615.27	5479.99	1263.44	3194.5	2315.77	1078.18	3950.57	17475.3	4686.02	5123.19	5215.63	7172.47	4334.02	101437.94	Phospholipid phosphatase-related protein type 1 (Inactive 2-lysophosphatidate phosphatase PLPPR1) (Lipid phosphate phosphatase-related protein type 1)	plppr1 lppr1 zgc:86759	nervous system development [GO:0007399]; phospholipid dephosphorylation [GO:0046839]; phospholipid metabolic process [GO:0006644]; signal transduction [GO:0007165]
5866.19	4926.82	6408.47	4988.08	4874.23	9198.39	7998.51	1998.58	2053.7	2791.3	2842.3	7204.62	7917.24	11923.78	2811.34	7803.72	6802.35	3022	101431.62	E3 SUMO-protein ligase ZBED1 (EC 2.3.2.-) (DNA replication-related element-binding factor) (Putative Ac-like transposable element) (Zinc finger BED domain-containing protein 1) (dREF homolog)	ZBED1 ALTE DREF hDREF KIAA0785 TRAMP	negative regulation by host of viral genome replication [GO:0044828]; positive regulation of transcription by RNA polymerase II [GO:0045944]; protein autosumoylation [GO:1990466]; protein sumoylation [GO:0016925]; regulation of DNA-templated transcription [GO:0006355]; regulation of transcription by RNA polymerase II [GO:0006357]

32.68	45306.35	68.78	32.22	13654.7	210.85	94.08	2971.23	38.41	105.26	3618.5	45.61	71.61	17474.45	182.7	62.1	16770.37	418.62	101158.52	Lysine-specific demethylase 2A (EC 1.14.11.27) (CXXC-type zinc finger protein 8) (F-box and leucine-rich repeat protein 11) (F-box protein FBL7) (F-box protein Lilina) (F-box/LRR-repeat protein 11) (JmjC domain-containing histone demethylation protein 1A) ([Histone-H3]-lysine-36 demethylase 1A)	KDM2A CXXC8 FBL11 FBL7 FBXL11 JHDM1A KIAA1004	chromatin remodeling [GO:0006338]; circadian regulation of gene expression [GO:0032922]; double-strand break repair via nonhomologous end joining [GO:0006303]; negative regulation of transcription by competitive promoter binding [GO:0010944]; regulation of circadian rhythm [GO:0042752]; regulation of transcription by RNA polymerase II [GO:0006357]
20537.3	1478.47	5363.49	10565.53	1812.94	3354.71	311.71	664.72	904.89	19037.65	1684.07	631.16	29707.97	416.03	1066.72	1726.55	687.02	943.47	100894.4	Serine/threonine-protein phosphatase 2A 55 kDa regulatory subunit B beta isoform (PP2A subunit B isoform B55-beta) (PP2A subunit B isoform PR55-beta) (PP2A subunit B isoform R2-beta) (PP2A subunit B isoform beta)	PPP2R2B	

9311.15	7892.76	6912.07	3787.75	7099.19	8627.86	3351.29	3432.7	3602.47	3017.21	2636.88	4939.01	5654.33	10335.42	4389.66	3748.17	6526.81	5624.67	100889.4	Ras-related protein Rab-6A (Rab-6) (EC 3.6.5.2)	RAB6A	intra-Golgi vesicle-mediated transport [GO:0006891]; intracellular protein transport [GO:0006886]; protein localization to Golgi apparatus [GO:0034067]; protein localization to Golgi membrane [GO:1903292]; retrograde transport, endosome to Golgi [GO:0042147]; retrograde vesicle-mediated transport, Golgi to endoplasmic reticulum [GO:0006890]
4986.25	4343.43	5028.94	2008.51	4631.98	4862.9	19959.18	2151.29	3782.05	2096.88	2227.91	3635.19	5848.22	6656.46	3756.2	16325.31	3674.11	4059.44	100034.25	Disks large-associated protein 1 (DAP-1) (Guanylate kinase-associated protein)	dlgap1 si:zc142h2.3	modulation of chemical synaptic transmission [GO:0050804]; signaling [GO:0023052]
4629.07	15241.06	7200.02	756.85	14863.78	7194.74	3226.21	10449.96	633.56	1096.16	3930.98	4437.43	2510.64	8472	2124.21	2415.25	7195.74	1752.49	98130.15	Tax1-binding protein 3 (Glutaminase-interacting protein 3) (Tax interaction protein 1) (TIP-1) (Tax-interacting protein 1)	TAX1BP3 TIP1	negative regulation of cell population proliferation [GO:0008285]; negative regulation of protein localization to cell surface [GO:2000009]; negative regulation of Wnt signaling pathway [GO:0030178]; regulation of Cdc42 protein signal transduction [GO:0032489]; Rho protein signal transduction [GO:0007266]; Wnt signaling pathway [GO:0016055]

6906.53	5443.29	5064.27	5105.93	6415.27	8785.75	73.63	4050.52	4571.53	6190.36	6778.53	16426.41	3248.98	867.65	8514.62	551.74	1749.53	7057.33	97801.87	Chymotrypsin-like elastase family member 2A (EC 3.4.21.71) (Elastase-2A)	CELA2A ELA2A	insulin catabolic process [GO:1901143]; proteolysis [GO:0006508]; regulation of insulin secretion [GO:0050796]; regulation of platelet aggregation [GO:0090330]; response to insulin [GO:0032868]
18032.89	309.23	70.37	6207.36	1874.81	7054.6	72.34	285.18	3.42	10996.11	842.84	284.29	42180.62	185.2	163.89	3260.6	411.35	66.3	92301.4	Transcription factor SOX-10 (cSOX10)	SOX10	cardiac neuron development [GO:0060959]; central nervous system myelination [GO:0022010]; enteric nervous system development [GO:0048484]; morphogenesis of an epithelium [GO:0002009]; negative regulation of transcription by RNA polymerase II [GO:0000122]; neural crest cell migration [GO:0001755]; oligodendrocyte development [GO:0014003]; oligodendrocyte differentiation [GO:0048709]; peripheral nervous system development [GO:0007422]; positive regulation of DNA-templated transcription [GO:0045893]

4892.82	7809.3	0	19934.82	12243.44	137.14	40.91	7146.81	0	4923.07	10160.45	0	2244.91	2352.76	1.03	5149.25	13310.83	126.25	90473.79	Actin-binding LIM protein 3 (abLIM-3) (Actin-binding LIM protein family member 3)	ABLIM3 KIAA0843 HMFN1661	cilium assembly [GO:0060271]; cytoskeleton organization [GO:0007010]; lamellipodium assembly [GO:0030032]; positive regulation of protein targeting to mitochondrion [GO:1903955]; positive regulation of transcription by RNA polymerase II [GO:0045944]; transcription by RNA polymerase II [GO:0006366]
1269.82	16154.95	0	294.18	9139.03	232.98	0	12221.49	0	328.57	4682.61	31.84	945.21	25757.24	138.35	119.44	17024.69	134.82	88475.22	Chaperone protein DnaK (HSP70) (Heat shock 70 kDa protein) (Heat shock protein 70)	dnaK VIBHAR_01134	
4266.3	6238.78	5870.5	1347.17	5240.41	11657.81	2227.76	2588.31	6954.09	2362.23	2809.94	7656.38	3477.2	6816.99	6100.17	1577.27	2827.09	6037.48	86055.88	Opsin-5 (G-protein coupled receptor 136) (G-protein coupled receptor PGR12) (Neuropsin) (Transmembrane protein 13)	OPN5 GPR136 PGR12 TMEM13	cellular response to light stimulus [GO:0071482]; cellular response to UV-A [GO:0071492]; entrainment of circadian clock by photoperiod [GO:0043153]; G protein-coupled receptor signaling pathway [GO:0007186]; hyaloid vascular plexus regression [GO:1990384]; phototransduction [GO:0007602]; phototransduction, UV [GO:0007604]; visual perception [GO:0007601]

2853.94	11671.76	2217.81	408.98	19041.53	6730.28	32.87	3448.56	541.8	646.2	7200.62	1810.01	1614.2	20848.27	1366.77	222.61	4661.16	673.22	85990.59	Protein phosphatase 1 regulatory subunit 37 (Leucine-rich repeat-containing protein 68)	PPP1R37 LRRC68	
6370.35	8303.54	4326.71	2547.16	9859.65	7759.91	1363.37	5205.68	439.38	3433.71	4313.84	3270.95	6878.77	8713.66	1938.73	2715.55	6546.24	1843.31	85830.51	DNA replication licensing factor mcm7-B (EC 3.6.4.12) (CDC47 homolog B) (CDC47-2p) (Minichromosome maintenance protein 7-B) (xMCM7-B)	mcm7-b cdc47-2	DNA replication [GO:0006260]; DNA replication initiation [GO:0006270]; DNA strand elongation involved in DNA replication [GO:0006271]; double-strand break repair via break-induced replication [GO:0000727]; regulation of DNA-templated DNA replication initiation [GO:0030174]
6083.27	8510.62	5.43	14302.26	8745.7	73.02	56.94	7868.84	1.33	7355.98	11257.23	1.21	1839.81	2891.37	0	4352.71	11875.19	67.68	85288.59	Evolutionarily conserved signaling intermediate in Toll pathway, mitochondrial	ecsit zgc:152999	cell surface receptor protein serine/threonine kinase signaling pathway [GO:0007178]; innate immune response [GO:0045087]; regulation of oxidoreductase activity [GO:0051341]; regulation of protein complex stability [GO:0061635]
2833.33	7229.91	6973.58	1958.22	5485.32	8696.91	82.78	4782.64	2857.03	1316.37	2409.54	8118.01	4486.93	10440.84	4918.39	1062.35	4731.36	6330.86	84714.37	Pre-mRNA-splicing factor SPF27 (Protein BCAS2 homolog)	bcas2 zgc:101730	mRNA splicing, via spliceosome [GO:0000398]

1845.27	15348.25	2461.19	2007.69	11564.72	3195.6	2246.57	7978.1	726.22	866.62	3631.29	2842.56	3288.07	9035.35	1667.87	2237.93	11791.03	1331.27	84065.6	CUB and sushi domain-containing protein 1 (CUB and sushi multiple domains protein 1)	CSMD1 KIAA1890 UNQ5952/PRO19863	conditioned place preference [GO:1990708]; female gonad development [GO:0008585]; gene expression [GO:0010467]; glucose homeostasis [GO:0042593]; male gonad development [GO:0008584]; mammary gland branching involved in pregnancy [GO:0060745]; memory [GO:0007613]; oviduct epithelium development [GO:0035846]; startle response [GO:0001964]
4088.92	4959.28	4190.17	3201.6	5290.11	8440.29	9341.55	2036.44	1973.77	2064.15	2767.44	5539.05	5576.66	7218.8	2415.06	7874.88	4577.67	2193.4	83749.24	Gamma-crystallin M3 (Gamma-M3)		lens development in camera-type eye [GO:0002088]; visual perception [GO:0007601]
15147.6	173.24	93.95	3269.81	689.33	1512.66	61.24	38.9	3.43	10976	431.33	760.99	48475.8	119.66	137.79	994.79	171.76	11.63	83069.91	Carbonyl reductase [NADPH] 1 (EC 1.1.1.184) (15-hydroxyprostaglandin dehydrogenase [NADP(+)]) (EC 1.1.1.196, EC 1.1.1.197) (20-beta-hydroxysteroid dehydrogenase) (Alcohol dehydrogenase [NAD(P)+] CBR1) (EC 1.1.1.71) (NADPH-dependent carbonyl reductase 1) (Prostaglandin 9-ketoreductase) (PG-9-KR) (Prostaglandin-F(2) 9-reductase)	CBR1	lipid metabolic process [GO:0006629]; vitamin K metabolic process [GO:0042373]; xenobiotic metabolic process [GO:0006805]

272.42	121.18	154.93	132.41	209.93	133.74	3.59	89.37	50.75	125.24	93.94	99.31	78075.32	1952.84	235.73	168.65	666.07	74.81	82660.23	Signal transducing adapter molecule 2 (STAM-2) (Hrs-binding protein)	Stam2 Hbp	protein transport [GO:0015031]
6324.42	8120.24	4.75	9114.35	5327.9	45.2	49.4	7262.63	0	8977.84	11536.63	3.03	3717.29	4797.11	1.89	4477.28	11861.52	119.18	81740.66	Peroxisomal N(1)-acetyl-spermine/spermidine oxidase (EC 1.5.3.13) (Polyamine oxidase)	Paox Pao	polyamine catabolic process [GO:0006598]; positive regulation of spermidine biosynthetic process [GO:1901307]; putrescine biosynthetic process [GO:0009446]; putrescine catabolic process [GO:0009447]; spermidine catabolic process [GO:0046203]; spermine catabolic process [GO:0046208]
7957.77	21324.2	4.78	9283.75	9495.32	54.93	48.82	1234.54	0	3412.19	7321.14	0	3733.69	6723.97	0	2747.47	8024.95	61.85	81429.37	Stathmin-2 (Superior cervical ganglion-10 protein) (Protein SCG10)	Stmn2 Scg10 Scgn10 Stmb2	cellular response to nerve growth factor stimulus [GO:1990090]; negative regulation of microtubule depolymerization [GO:0007026]; negative regulation of microtubule polymerization [GO:0031115]; negative regulation of neuron projection development [GO:0010977]; positive regulation of microtubule depolymerization [GO:0031117]; positive regulation of neuron projection development [GO:0010976]

59.02	26857.79	71.79	42.63	11476.75	141.32	0	7163.48	63.55	49.96	2611.37	98.68	26.76	22195.16	105.03	116.39	9945.25	154.45	81179.38	Mitogen-activated protein kinase kinase kinase 7-interacting protein 3 homolog	map3k7ip3	positive regulation of canonical NF-kappaB signal transduction [GO:0043123]
953.77	2613.68	5970.3	2245.1	2003.72	2246.79	38.97	2094.78	721.85	1142.22	1849.1	8583.33	1150.84	28142.04	2266.18	7403.59	9618.35	1397.84	80442.45	NF-kappa-B essential modulator (NEMO) (IkB kinase-associated protein 1) (IKKAP1) (Inhibitor of nuclear factor kappa-B kinase subunit gamma) (I-kappa-B kinase subunit gamma) (IKK-gamma) (IKKG) (IkB kinase subunit gamma) (NF-kappa-B essential modifier)	IKBKG NEMO	DNA damage response [GO:0006974]; positive regulation of canonical NF-kappaB signal transduction [GO:0043123]; positive regulation of NF-kappaB transcription factor activity [GO:0051092]; positive regulation of T cell receptor signaling pathway [GO:0050862]; positive regulation of transcription by RNA polymerase II [GO:0045944]
7392.65	5278.38	4309.79	4590.79	3942.37	8956.72	14.82	21384.27	5820.7	4177.29	2575.31	1500.43	1859.49	2774.39	1122.65	309.18	2775.4	1655.93	80440.56	Gamma-aminobutyric acid receptor subunit beta-3 (GABA(A) receptor subunit beta-3) (GABAAR subunit beta-3)	GABRB3	chloride transmembrane transport [GO:1902476]; gamma-aminobutyric acid signaling pathway [GO:0007214]; inhibitory synapse assembly [GO:1904862]

380.73	496.54	260	157.76	346.29	429.9	37.37	364.6	33.63	129.98	629.62	259.72	671.97	50789.28	303.51	3339.23	21302.87	428.18	80361.18	Probable JmjC domain-containing histone demethylation protein 2C (EC 1.14.11.-) (Jumonji domain-containing protein 1C) (Thyroid receptor-interacting protein 8) (TR-interacting protein 8) (TRIP-8)	JMJD1C JHDM2C KIAA1380 TRIP8	blood coagulation [GO:0007596]; regulation of DNA-templated transcription [GO:0006355]; regulation of transcription by RNA polymerase II [GO:0006357]
250.01	6.63	0.96	1073.48	6.64	26.78	41861.69	8.48	36.84	587.12	1591.07	28.91	20.94	9.1	12.61	33050.37	704.7	86.39	79362.72	Sodium/potassium/calcium exchanger 3 (Na(+)/K(+)/Ca(2+)-exchange protein 3) (Solute carrier family 24 member 3)	Slc24a3 Nckx3	bone mineralization [GO:0030282]; calcium ion transmembrane transport [GO:0070588]; intracellular calcium ion homeostasis [GO:0006874]; monoatomic cation transport [GO:0006812]; negative regulation of gene expression [GO:0010629]; positive regulation of gene expression [GO:0010628]; potassium ion transmembrane transport [GO:0071805]; sodium ion transmembrane transport [GO:0035725]
12999.38	104.52	70.43	3846.37	645.74	2289.8	46.14	42.78	1.44	13520.42	392.36	600.84	42183.8	72.79	155.71	1098.63	161.34	6.48	78238.97	Homeobox protein HMX1 (GH6) (Homeobox protein H6)	HMX1 H6	negative regulation of DNA-templated transcription [GO:0045892]; regulation of transcription by RNA polymerase II [GO:0006357]

3852.69	6577.51	5807.96	2090.26	7087.93	5427.64	6544.23	3745.31	2020.65	1435.34	3503.2	3927.22	4672.91	7060.3	2392.58	4865.39	4942.05	2219.64	78172.81	Elongation factor 1-alpha (EF-1-alpha) (42Sp50) (Thesaurin A)		translation [GO:0006412]; translational elongation [GO:0006414]
22565.62	994.12	10777.31	2481.73	579.04	9587.16	255.65	286.82	516.22	4924.79	662.72	3735.16	9607.48	2067.59	3972.27	1959.07	1521.75	1098.28	77592.78	Kelch-like protein 29 (Kelch repeat and BTB domain-containing protein 9)	KLHL29 KBTBD9 KIAA1921	proteasome-mediated ubiquitin-dependent protein catabolic process [GO:0043161]
3266.85	4570.72	5132.26	3065.45	5999.57	5879.78	6677.58	2544.06	1676.63	1856.1	3323.95	5963.51	4181.61	7162.05	2019.21	7419.12	4368.09	2053.46	77160	Centromere-associated protein E (Centromere protein E) (CENP-E) (Kinesin-7) (Kinesin-related protein CENPE)	CENPE	attachment of mitotic spindle microtubules to kinetochore [GO:0051315]; cell division [GO:0051301]; chromosome segregation [GO:0007059]; kinetochore assembly [GO:0051382]; lateral attachment of mitotic spindle microtubules to kinetochore [GO:0099607]; metaphase chromosome alignment [GO:0051310]; microtubule plus-end directed mitotic chromosome migration [GO:0099606]; microtubule-based movement [GO:0007018]; mitotic cell cycle [GO:0000278]; mitotic chromosome movement towards spindle pole [GO:0007079]; mitotic metaphase chromosome alignment [GO:0007080]; mitotic spindle organization [GO:0007052]; positive regulation of protein kinase activity [GO:0045860]; regulation of mitotic metaphase/anaphase transition [GO:0030071]

2803.63	4066.36	4543.09	3241.68	5087.45	6637.22	7356.73	1834.65	1666.16	2028.69	2761.29	5520.77	4934.06	7772.14	2350.14	6318.54	4538.3	1959.21	75420.11	Voltage-gated purine nucleotide uniporter SLC17A9 (Solute carrier family 17 member 9) (Vesicular nucleotide transporter) (VNUT)	Slc17a9	ADP transport [GO:0015866]; ATP export [GO:1904669]; ATP transport [GO:0015867]; guanine nucleotide transmembrane transport [GO:1903790]; lysosomal protein catabolic process [GO:1905146]; purine nucleotide import into lysosome [GO:0141013]
4019.57	3423.64	5280.85	4931.13	3563.36	7081.98	5293.71	1777.75	1509.33	2736.85	2585.57	5610.65	5692.06	6713.06	2111.98	5709.75	4885.61	2204.5	75131.35	Phosphatidylinositol transfer protein beta isoform (PI-TP-beta) (PtdIns transfer protein beta) (PtdInsTP beta) (Phosphatidylinositol-transfer protein 36 kDa isoform) (PI-TP 36 kda isoform)	PITPNB	retrograde vesicle-mediated transport, Golgi to endoplasmic reticulum [GO:0006890]

3677.32	3856.42	6755.25	2007.59	5761.29	5323.75	6020.99	2279.91	3580.44	1520.27	2259.72	4270.8	3925.93	6715.16	3693.63	4854.74	3716.69	4506.97	74726.87	Beta-hexosaminidase subunit beta (EC 3.2.1.52) (Beta-N-acetylhexosaminidase subunit beta) (Hexosaminidase subunit B) (N-acetyl-beta-glucosaminidase subunit beta)	HEXB	carbohydrate metabolic process [GO:0005975]; ganglioside catabolic process [GO:0006689]; glycosaminoglycan metabolic process [GO:0030203]; N-glycan processing [GO:0006491]; single fertilization [GO:0007338]
0	0	0	0	0	74535.57	18.96	0	0	0	0	13.86	14.35	0	0	11.69	4.3	8.39	74607.12	Glycine receptor subunit beta (Glycine receptor 58 kDa subunit)	GLRB	acrosome reaction [GO:0007340]; adult walking behavior [GO:0007628]; chemical synaptic transmission [GO:0007268]; chloride transmembrane transport [GO:1902476]; gamma-aminobutyric acid receptor clustering [GO:0097112]; monoatomic ion transport [GO:0006811]; nervous system development [GO:0007399]; neuropeptide signaling pathway [GO:0007218]; righting reflex [GO:0060013]; startle response [GO:0001964]; synaptic transmission, glycinergic [GO:0060012]; visual perception [GO:0007601]

2834.98		1494.72	3941.11	1998.44	1548.26	10454.62	83.21	714.24	278.12	1625.34	1001.35	2079.76	17726.99	9556.25	11289.75	2315.05	4548.24	932.76	74423.19	Prostaglandin E2 receptor EP1 subtype (PGE receptor EP1 subtype) (PGE2 receptor EP1 subtype) (Prostanoid EP1 receptor)	Ptger1 Ptgerep1	adenylate cyclase-activating dopamine receptor signaling pathway [GO:0007191]; response to lipopolysaccharide [GO:0032496]; response to prostaglandin E [GO:0034695]
2734.36		5809.45	3464.35	3939.37	5545.08	8503.16	39.86	3043.95	2446.97	6502.6	5902.54	4131.57	3799.68	8308.52	3112.06	1015.99	2842.02	2688.23	73829.76	Guanine nucleotide-binding protein G(i) subunit alpha-2 (Adenylate cyclase-inhibiting G alpha protein)	GNAI2	adenylate cyclase-inhibiting G protein-coupled receptor signaling pathway [GO:0007193]; cell division [GO:0051301]; cell population proliferation [GO:0008283]; G protein-coupled acetylcholine receptor signaling pathway [GO:0007213]; G protein-coupled adenosine receptor signaling pathway [GO:0001973]; gamma-aminobutyric acid signaling pathway [GO:0007214]; modulation of chemical synaptic transmission [GO:0050804]; positive regulation of cell population proliferation [GO:0008284]

2523.4	4705.31	3632.85	1370.25	8159.96	6650.74	1202.89	3695.14	717.91	1307.5	2673.08	3177.68	5465.69	9552.57	5021.77	1113.08	6030.28	5881.65	72881.75	NLR family CARD domain-containing protein 3 (CARD15-like protein) (Caterpiller protein 16.2) (CLR16.2) (NACHT, LRR and CARD domains-containing protein 3) (Nucleotide-binding oligomerization domain protein 3)	NLRC3 NOD3	canonical NF-kappaB signal transduction [GO:0007249]; negative regulation of canonical NF-kappaB signal transduction [GO:0043124]; negative regulation of cytokine production involved in inflammatory response [GO:1900016]; negative regulation of epithelial cell proliferation [GO:0050680]; negative regulation of fibroblast proliferation [GO:0048147]; negative regulation of inflammatory response [GO:0050728]; negative regulation of innate immune response [GO:0045824]; negative regulation of interferon-alpha production [GO:0032687]; negative regulation of interferon-beta production [GO:0032688]; negative regulation of interleukin-6 production [GO:0032715]; negative regulation of NF-kappaB transcription factor activity [GO:0032088]; negative regulation of NLRP3 inflammasome complex assembly [GO:1900226]; negative regulation of non-canonical NF-kappaB signal transduction [GO:1901223];
126.63	0	0	876.9	0	35.59	54268.62	1.98	0	431.81	795.54	16.58	36.27	0	13.67	14877.61	682.17	22.08	72185.45	Serine/threonine-protein phosphatase 2A 55 kDa regulatory subunit B delta isoform (PP2A subunit B isoform B55-delta) (PP2A subunit B isoform PR55-delta) (PP2A subunit B isoform R2-delta) (PP2A subunit B isoform delta)	ppp2r2d zgc:76887	cell division [GO:0051301]; exit from mitosis [GO:0010458]; mitotic cell cycle [GO:0000278]; myoblast fusion [GO:0007520]; regulation of chromosome segregation [GO:0051983]

0	0	71924.65	0	0	0	0	0	1.28	0	0	0	0	0	0	0	0	2.87	71928.8	Regulating synaptic membrane exocytosis protein 1 (Rab-3-interacting molecule 1) (RIM 1) (Rab-3-interacting protein 2)	RIMS1 KIAA0340 RAB3IP2 RIM1 Nbla00761	acrosomal vesicle exocytosis [GO:0060478]; calcium-ion regulated exocytosis [GO:0017156]; cell differentiation [GO:0030154]; intracellular protein transport [GO:0006886]; membrane fusion [GO:0061025]; positive regulation of dendrite extension [GO:1903861]; positive regulation of excitatory postsynaptic potential [GO:2000463]; positive regulation of gene expression [GO:0010628]; positive regulation of inhibitory postsynaptic potential [GO:0097151]; protein-containing complex assembly [GO:0065003]; regulated exocytosis [GO:0045055]; regulation of neurotransmitter secretion [GO:0046928]; regulation of synaptic vesicle exocytosis [GO:2000300]; secretion [GO:0046903]; synaptic vesicle docking [GO:0016081]; synaptic vesicle exocytosis [GO:0016079]; synaptic vesicle priming [GO:0016082]; visual perception [GO:0007601]
2.88	24198.55	34.65	4.08	7589.49	165.3	0.81	5361.8	9.77	7.88	4301.09	176.73	3.09	20825.75	19.14	2.99	8889.26	40.93	71634.19	Glucoside xylosyltransferase 1 (EC 2.4.2.42) (Glycosyltransferase 8 domain-containing protein 3)	Gxylt1 Glt8d3 Gm87	O-glycan processing [GO:0016266]
2139.68	3317.83	7423.47	1715.84	3285.85	4810.33	7913.82	1775.14	2027.24	1012.34	1366.5	11972.96	2852.57	5353.27	2359.28	5717.76	3037.23	2141.55	70222.66	Dehydrogenase/reductase SDR family member 11 (17-beta-hydroxysteroid dehydrogenase) (3-beta-hydroxysteroid 3-dehydrogenase) (EC 1.1.1.270) (Estradiol 17-beta-dehydrogenase) (EC 1.1.1.62) (Short-chain dehydrogenase/reductase family 24C member 1)	DHRS11 SDR24C1	estrogen biosynthetic process [GO:0006703]

208.35	0	0	1320.85	1.82	46.75	49937.05	0	0	184.02	258.92	17.15	63.49	0	3.39	17453.35	407.66	11.97	69914.77	Actin-binding protein IPP (Intracisternal A particle-promoted polypeptide) (IPP) (Kelch-like protein 27)	IPP KLHL27	proteasome-mediated ubiquitin-dependent protein catabolic process [GO:0043161]
188.28	0	0	503.66	0	10.77	6695.57	0	0	155.06	480.91	0	0	0	0	59402.27	1081.71	0	68518.23	Retinol dehydrogenase 13 (EC 1.1.1.300) (Short chain dehydrogenase/reductase family 7C member 3)	RDH13 SDR7C3 PSEC0082 UNQ736/PRO1430	eye photoreceptor cell development [GO:0042462]; response to high light intensity [GO:0009644]; retina layer formation [GO:0010842]; retinal metabolic process [GO:0042574]
0	0	9.93	0	0	68374.09	0	0	0	7.68	0	0	13.43	0	22.5	20.61	0	44.82	68493.06	Rho guanine nucleotide exchange factor TIAM1 (T-lymphoma invasion and metastasis-inducing protein 1) (TIAM-1)	Tiam1 Tiam-1	activation of GTPase activity [GO:0090630]; brain-derived neurotrophic factor receptor signaling pathway [GO:0031547]; cell migration [GO:0016477]; cell-matrix adhesion [GO:0007160]; ephrin receptor signaling pathway [GO:0048013]; positive regulation of axonogenesis [GO:0050772]; positive regulation of neuron projection development [GO:0010976]; Rac protein signal transduction [GO:0016601]; regulation of dopaminergic neuron differentiation [GO:1904338]; regulation of GTPase activity [GO:0043087]; regulation of non-canonical Wnt signaling pathway [GO:2000050]; small GTPase-mediated signal transduction [GO:0007264]

1938.54	1524.7	3964.68	1170.56	1713.6	2356.58	15853.6	543.84	3957.78	712.04	965.64	6363.93	2851.42	3112.89	3385.25	10638.8	1895.95	4530.11	67479.91	Poly(rC)-binding protein 2 (Alpha-CP2) (CTBP) (CBP) (Putative heterogeneous nuclear ribonucleoprotein X) (hnRNP X)	Pcbp2 Cbp Hnrnpx Hnrpx	defense response to virus [GO:0051607]; innate immune response [GO:0045087]; negative regulation of defense response to virus [GO:0050687]; positive regulation of transcription by RNA polymerase II [GO:0045944]; proteasome-mediated ubiquitin-dependent protein catabolic process [GO:0043161]; protein maturation [GO:0051604]
0.78	22924.69	0.56	6.23	7871.54	40.49	0.61	7936.12	1.79	1.04	4262.98	3.4	2.41	13048.16	0	3.29	10204.75	60.32	66369.16	Peroxidasin (EC 1.11.2.-) [Cleaved into: PXDN active fragment]	pxdn pxn	angiogenesis [GO:0001525]; basement membrane assembly [GO:0070831]; basement membrane organization [GO:0071711]; cell adhesion [GO:0007155]; hydrogen peroxide catabolic process [GO:0042744]; protein homooligomerization [GO:0051260]; protein homotrimerization [GO:0070207]; response to oxidative stress [GO:0006979]
79.89	10.89	49.67	391.36	19.39	46.04	33754.48	4.89	12.3	455.17	846.85	23.19	55.12	25.53	50.3	29211.51	759.8	62.04	65858.42	cGMP-specific 3',5'-cyclic phosphodiesterase (EC 3.1.4.35) (cGMP-binding cGMP-specific phosphodiesterase) (CGB-PDE)	PDE5A PDE5	cAMP-mediated signaling [GO:0019933]; cGMP catabolic process [GO:0046069]

2154.35		3991.76	7902.9	1874	5872.76	6769.14	1595.18	2093.25	4278.36	3669.91	3284.15	1316.57	3568.75	4916.09	3919.3	2392.27	3626.57	2587.07	65812.38	Retrovirus-related Pol polyprotein from transposon 17.6 [Includes: Protease (EC 3.4.23.-);Reverse transcriptase (EC 2.7.7.49);Endonuclease]	pol	DNA integration [GO:0015074]; proteolysis [GO:0006508]
1077.16		11950.28	1897.85	1067.1	11052.05	1477.86	374.9	3411.62	1076.57	412.5	3003.1	1721.97	951.51	16021.34	1373.94	618.3	6603.82	1694.37	65786.24	Serine/threonine-protein kinase 26 (EC 2.7.11.1) (MST3 and SOK1-related kinase) (Mammalian STE20-like protein kinase 4) (MST-4) (STE20-like kinase MST4) (Serine/threonine-protein kinase MASK)	STK26 MASK MST4	apoptotic process [GO:0006915]; cellular response to oxidative stress [GO:0034599]; cellular response to starvation [GO:0009267]; intracellular signal transduction [GO:0035556]; microvillus assembly [GO:0030033]; negative regulation of cell migration [GO:0030336]; protein autophosphorylation [GO:0046777]; protein phosphorylation [GO:0006468]; regulation of apoptotic process [GO:0042981]
3125.63		1866.83	6909.87	1657.41	1519.44	9441.62	18.94	2373.55	7279.69	3242.63	1200.82	12135.83	1936.58	1375.43	4936.96	156.86	991.18	5098.95	65268.22	Lanosterol synthase (EC 5.4.99.7) (2,3-epoxysqualene--lanosterol cyclase) (Oxidosqualene--lanosterol cyclase) (OSC)	Lss Osc	cholesterol biosynthetic process [GO:0006695]; regulation of protein stability [GO:0031647]; steroid biosynthetic process [GO:0006694]; sterol metabolic process [GO:0016125]; triterpenoid biosynthetic process [GO:0016104]

2648.87	3920.67	5213.47	2382.25	4095.14	5517.28	2918.18	1862.47	2151.03	1477.12	2040.83	5066.63	5098.41	7879.89	2876.73	3611.1	3796.14	2202.71	64758.92	Transcription factor IIIB 50 kDa subunit (B-related factor 2) (BRF-2)	brf2 zgc:100856	cellular response to oxidative stress [GO:0034599]; regulation of transcription by RNA polymerase III [GO:0006359]; transcription preinitiation complex assembly [GO:0070897]
5520.66	1530.39	3785.16	1975.6	1104.63	4613.12	411.68	322.67	6622.32	1347.08	549.98	3147.92	10363.23	3719.16	7446.34	1583.46	3097.35	7451.84	64592.59	Probable E3 ubiquitin-protein ligase HERC3 (EC 2.3.2.26) (HECT domain and RCC1-like domain-containing protein 3) (HECT-type E3 ubiquitin transferase HERC3)	HERC3 KIAA0032	protein ubiquitination [GO:0016567]; ubiquitin-dependent protein catabolic process [GO:0006511]
2743.9	4925.89	3873.15	1806.84	4317.54	5874.08	136.32	6325.96	4994.27	2880.27	3453.41	1961.95	2270.31	9764.57	2927.56	1023.27	2868.44	2217.96	64365.69	Protein phosphatase 1 regulatory subunit 12A (Myosin phosphatase-targeting subunit 1) (Myosin phosphatase target subunit 1) (Protein phosphatase myosin-binding subunit)	ppp1r12a mbs mypt1 si:dkey-28j4.1 zgc:110448	actin filament bundle assembly [GO:0051017]; actin filament bundle distribution [GO:0070650]; axonogenesis [GO:0007409]; convergent extension involved in axis elongation [GO:0060028]; exocrine pancreas development [GO:0031017]; liver development [GO:0001889]; midbrain-hindbrain boundary morphogenesis [GO:0021555]; motor neuron axon guidance [GO:0008045]; neuron projection morphogenesis [GO:0048812]; regulation of BMP signaling pathway [GO:0030510]; regulation of cell adhesion [GO:0030155]; regulation of cell shape [GO:0008360]; signal transduction [GO:0007165]

3418.07		3199.99	3676.95	1323.67	2909.71	3650.35	4392	1255.54	7703.2	999.28	1029.52	4006.94	4478.3	3465.52	7377.12	2939.73	1907.77	6525.3	64258.96	Axin-1 (Axis inhibition protein 1) (Protein Fused)	Axin1 Axin Fu	apoptotic process [GO:0006915]; axial mesoderm development [GO:0048318]; axial mesoderm formation [GO:0048320]; beta-catenin destruction complex assembly [GO:1904885]; canonical Wnt signaling pathway [GO:0060070]; cytoplasmic microtubule organization [GO:0031122]; dorsal/ventral axis specification [GO:0009950]; dorsal/ventral pattern formation [GO:0009953]; epigenetic programming in the zygotic pronuclei [GO:0044725]; head development [GO:0060322]; in utero embryonic development [GO:0001701]; negative regulation of canonical Wnt signaling pathway [GO:0090090]; negative regulation of fat cell differentiation [GO:0045599]; negative regulation of gene expression [GO:0010629]; negative regulation of protein metabolic process [GO:0051248]; negative regulation of transcription elongation by RNA polymerase II [GO:0034244]; negative regulation of Wnt signaling pathway
1228.85		3162.27	4356.19	1495.14	3643.94	3073.75	131.95	1435.64	4847.82	1126.6	1519.68	7420.59	1467.96	8462.5	8912.07	1793.07	3904.64	6225.35	64208.01	Properdin (Complement factor P)	Cfp Pfc	complement activation [GO:0006956]; complement activation, alternative pathway [GO:0006957]; positive regulation of immune response [GO:0050778]; positive regulation of opsonization [GO:1903028]
3194.51		4226.07	6273.25	1445.97	2518.58	3228.35	4545.47	1797.01	1965.61	2066.78	2708.98	6307.6	5815.06	9054.97	2668.96	1989.05	2809.36	1459.92	64075.5	Mesoderm induction early response protein 3 (Mi-er3)	Mier3	

2230.85	2949.14	2926.1	1963.84	3522.18	4632.98	9710.72	1254.94	1328.36	1332.3	1918.27	3485.48	4122.16	8301.82	2148.35	6449.62	4238.28	1463.96	63979.35	Cadherin EGF LAG seven-pass G-type receptor 3	Celsr3	axonal fasciculation [GO:0007413]; cilium assembly [GO:0060271]; dopaminergic neuron axon guidance [GO:0036514]; homophilic cell adhesion via plasma membrane adhesion molecules [GO:0007156]; motor neuron migration [GO:0097475]; regulation of protein localization [GO:0032880]; serotonergic neuron axon guidance [GO:0036515]; Wnt signaling pathway, planar cell polarity pathway [GO:0060071]
3954.89	6305.99	1673.61	1341.41	6225.43	2791.94	240.86	2921.13	3073.75	852.68	2170.93	1608.36	1911.4	13716.71	1353.36	1088.45	10165.42	2539.17	63935.49	Prolyl 3-hydroxylase OGFOD1 (EC 1.14.11.-) (2-oxoglutarate and iron-dependent oxygenase domain-containing protein 1) (uS12 prolyl 3-hydroxylase)	OGFOD1	cell population proliferation [GO:0008283]; protein hydroxylation [GO:0018126]; regulation of translational termination [GO:0006449]; stress granule assembly [GO:0034063]

14541.9	117.89	75.29	2640.72	421.52	645.94	30.39	36.68	1.58	14688.32	302.22	489.72	28719.04	47.7	69.04	966.36	114.49	5.31	63914.11	Rab11 family-interacting protein 3 (FIP3) (FIP3-Rab11) (Rab11-FIP3) (Arfophilin-1) (EF hands-containing Rab-interacting protein) (Eferin) (MU-MB-17.148)	RAB11FIP3 ARFO1 KIAA0665	cell division [GO:0051301]; early endosome to recycling endosome transport [GO:0061502]; endocytic recycling [GO:0032456]; Golgi to plasma membrane protein transport [GO:0043001]; negative regulation of adiponectin secretion [GO:0070164]; positive regulation of cilium assembly [GO:0045724]; positive regulation of mitotic cytokinetic process [GO:1903438]; protein localization to cilium [GO:0061512]; protein localization to cleavage furrow [GO:1905345]; regulation of cilium assembly [GO:1902017]; regulation of cytokinesis [GO:0032465]; regulation of early endosome to recycling endosome transport [GO:1902954]; regulation of endocytic recycling [GO:2001135]; regulation of protein localization to centrosome [GO:1904779]; regulation of vesicle-mediated transport [GO:0060627]; vesicle-mediated transport [GO:0016192]
6.55	0	62785.31	0	3.34	0	0	0	0	0	0	0	0	0	0	0	0	0	62795.2	Trafficking regulator of GLUT4 1 (Dispanin subfamily B member 1) (DSPB1) (Tumor suppressor candidate 5 homolog)	trarg1 tusc5	cellular response to insulin stimulus [GO:0032869]; endosome to plasma membrane protein transport [GO:0099638]; glucose import in response to insulin stimulus [GO:0044381]; protein localization to plasma membrane [GO:0072659]
3415.58	3251.9	2981.53	2596.28	3704.17	5846.35	5785.24	1418.88	1796.84	1676.79	1759.11	3231.85	4855.69	7768.39	1966.06	5625.85	3416.23	1665.11	62761.85	NF-kappa-B inhibitor-interacting Ras-like protein 1 (I-kappa-B-interacting Ras-like protein 1) (Kappa B-Ras protein 1) (KappaB-Ras1)	nkiras1 si:dkey-126a1.3 zgc:92823	negative regulation of canonical NF-kappaB signal transduction [GO:0043124]; Ral protein signal transduction [GO:0032484]

5038.22	5457.24	5249.88	917.67	4199.96	3540.74	124.65	4397.81	749.2	1598.25	3563.36	2818.86	6837.62	9725.51	2647.05	523.84	3468.23	1570.77	62428.86	Enhancer of polycomb homolog 1	Epc1	double-strand break repair via homologous recombination [GO:0000724]; negative regulation of gene expression, epigenetic [GO:0045814]; negative regulation of transcription by RNA polymerase II [GO:0000122]; positive regulation of DNA-templated transcription [GO:0045893]; positive regulation of double-strand break repair via homologous recombination [GO:1905168]; positive regulation of striated muscle cell differentiation [GO:0051155]; positive regulation of transcription by RNA polymerase II [GO:0045944]; regulation of apoptotic process [GO:0042981]; regulation of cell cycle [GO:0051726]; regulation of chromatin organization [GO:1902275]; regulation of double-strand break repair [GO:2000779]; sperm DNA condensation [GO:0035092]; vascular associated smooth muscle cell differentiation [GO:0035886]
13392.29	192.14	150.36	5510.57	2046.72	4520.62	56.78	171.49	7.66	9312.85	1126.37	410.51	24193.77	128.82	127.64	685.53	256.53	17.25	62307.9	Vasopressin V2 receptor (V2R) (AVPR V2) (Antidiuretic hormone receptor) (Renal-type arginine vasopressin receptor)	AVPR2	cellular response to hormone stimulus [GO:0032870]; G protein-coupled receptor signaling pathway [GO:0007186]; positive regulation of gene expression [GO:0010628]; positive regulation of vasoconstriction [GO:0045907]; regulation of systemic arterial blood pressure by vasopressin [GO:0001992]

9075.41	934.86	3620.09	3191.23	3558.09	12790.13	16.72	652.4	123.76	3584.13	1169.73	2043.65	9557.84	1797.56	4094.66	1645.94	3538.84	793	62188.04	N-acyl-phosphatidylethanolamine-hydrolyzing phospholipase D (N-acyl phosphatidylethanolamine phospholipase D) (NAPE-PLD) (NAPE-hydrolyzing phospholipase D) (EC 3.1.4.54)	Napepld	host-mediated regulation of intestinal microbiota composition [GO:0048874]; N-acylethanolamine metabolic process [GO:0070291]; N-acylphosphatidylethanolamine metabolic process [GO:0070292]; negative regulation of eating behavior [GO:1903999]; phospholipid catabolic process [GO:0009395]; phospholipid metabolic process [GO:0006644]; positive regulation of brown fat cell differentiation [GO:0090336]; positive regulation of inflammatory response [GO:0050729]; response to isolation stress [GO:0035900]; temperature homeostasis [GO:0001659]
1867.69	563.1	1643.01	646.98	452.53	2052.55	9.66	98.78	13370.39	400.68	223.14	6100.01	2552.35	862.26	12564.38	442.65	654.72	17193.71	61698.59	Retinal homeobox protein Rx3	rx3	adenohypophysis development [GO:0021984]; camera-type eye morphogenesis [GO:0048593]; cell fate specification [GO:0001708]; embryonic camera-type eye morphogenesis [GO:0048596]; eye development [GO:0001654]; eye field cell fate commitment involved in camera-type eye formation [GO:0060898]; forebrain development [GO:0030900]; hypothalamus development [GO:0021854]; neural crest cell migration [GO:0001755]; positive regulation of transcription by RNA polymerase II [GO:0045944]; regulation of transcription by RNA polymerase II [GO:0006357]; retina development in camera-type eye [GO:0060041]
31965.08	454.18	299.73	9207.66	1875.56	1574.94	68.55	651.9	205.03	4666.14	862.16	477.74	5809.85	602.41	179.48	618.82	1450.45	135.31	61104.99	Transposable element Tcb1 transposase (Transposable element Barney transposase)		DNA integration [GO:0015074]; DNA transposition [GO:0006313]

5900.41	7596.72	3142.38	1264.52	4374.77	7877.94	80.46	2093.33	872.72	1164.62	1565.71	5080.22	5871.21	5159.03	2842.56	513.12	3543.47	2053.66	60996.85	Mitochondrial inner membrane protein OXA1L (Oxidase assembly 1-like protein) (OXA1-like protein)	OXA1L	mitochondrial protein quality control [GO:0141164]; mitochondrial respiratory chain complex I assembly [GO:0032981]; protein insertion into mitochondrial inner membrane from matrix [GO:0032979]; protein tetramerization [GO:0051262]
1422.69	466.68	3286.05	1166.71	1137.7	3666.31	30682.54	533.06	653.9	1108.05	988.64	3109.59	4774.8	1550.93	2614.17	2869.8	336.9	242.55	60611.07	Protein FAM13B (GAP-like protein N61)	FAM13B C5orf5 FAM13B1	regulation of small GTPase mediated signal transduction [GO:0051056]; signal transduction [GO:0007165]
1696.96	1653.11	1549.35	1661.66	2204.51	5331.15	92	28489.81	4239.05	2200.3	1230.81	1504.68	990.91	2336.37	1702.79	195.88	1413.68	2051.59	60544.61	Semaphorin-6A (Semaphorin VIA) (Sema VIA) (Semaphorin-6A-1) (SEMA6A-1)	SEMA6A KIAA1368 SEMAQ	animal organ morphogenesis [GO:0009887]; apoptotic process [GO:0006915]; axon guidance [GO:0007411]; cell surface receptor signaling pathway [GO:0007166]; cellular response to vascular endothelial growth factor stimulus [GO:0035924]; cytoskeleton organization [GO:0007010]; negative regulation of angiogenesis [GO:0016525]; negative regulation of cell adhesion involved in sprouting angiogenesis [GO:0106089]; negative regulation of ERK1 and ERK2 cascade [GO:0070373]; negative regulation of sprouting angiogenesis [GO:1903671]; negative regulation of vascular endothelial growth factor signaling pathway [GO:1900747]; nervous system development [GO:0007399]; neural crest cell migration [GO:0001755]; positive regulation of neuron migration [GO:2001224]; semaphorin-plexin signaling pathway [GO:0071526]

3008.53	4713.48	5.67	18310.24	9936.47	63.79	65.22	5970.57	0	1591.95	2659.04	0	1683.51	2126.27	0	2697.83	7529.08	85.03	60446.68	Pro-opiomelanocortin B (POMC-B) (Corticotropin-lipotropin B) [Cleaved into: NPP 2;Corticotropin (Adrenocorticotropic hormone) (ACTH);Melanocyte-stimulating hormone alpha 2 (Alpha-MSH 2) (Melanotropin alpha 2);Corticotropin-like intermediary peptide 2 (CLIP-2);Lipotropin beta (Beta-LPH);Lipotropin gamma (Gamma-LPH);Melanocyte-stimulating hormone beta 2	pomcb	neuropeptide signaling pathway [GO:0007218]
2661.47	2262.49	1955.15	1573.28	1832.79	3295.3	12516.65	985.11	1855.47	1321	1672.09	3170.2	3890.44	6835.56	2046.48	8508.02	2603.23	1368.81	60353.54	Golgi reassembly-stacking protein 2 (GRS2) (Golgi phosphoprotein 6) (GOLPH6) (Golgi reassembly-stacking protein of 55 kDa) (GRASP55) (p59)	GORASP2 GOLPH6	cell differentiation [GO:0030154]; establishment of protein localization to plasma membrane [GO:0061951]; Golgi organization [GO:0007030]; organelle assembly [GO:0070925]; organelle organization [GO:0006996]; response to endoplasmic reticulum stress [GO:0034976]; response to unfolded protein [GO:0006986]; spermatogenesis [GO:0007283]

2985.52	4125.3	2938.37	1432.39	4644.24	6380.98	510.4	2608.58	650.59	1529.94	1638.24	2797.24	6742.19	8445.29	4014.61	1243.42	4163.56	3434.23	60285.09	Death domain-associated protein 6 (Daxx)	DAXX	androgen receptor signaling pathway [GO:0030521]; cellular response to cadmium ion [GO:0071276]; cellular response to copper ion [GO:0071280]; cellular response to diamide [GO:0072738]; cellular response to heat [GO:0034605]; cellular response to sodium arsenite [GO:1903936]; cellular response to unfolded protein [GO:0034620]; extrinsic apoptotic signaling pathway via death domain receptors [GO:0008625]; JNK cascade [GO:0007254]; negative regulation of DNA-templated transcription [GO:0045892]; negative regulation of gene expression [GO:0010629]; neuron intrinsic apoptotic signaling pathway in response to oxidative stress [GO:0036480]; nucleosome assembly [GO:0006334]; regulation of apoptotic process [GO:0042981]; regulation of protein ubiquitination [GO:0031396]
0	1.22	4563.96	0	0	423.55	0	0	14995.26	0	7.24	14310.01	3.37	0	7428.94	1.49	12.03	18272.6	60019.67	Integrin beta-7 (Gut homing receptor beta subunit)	ITGB7	cell adhesion [GO:0007155]; cell adhesion mediated by integrin [GO:0033627]; cell-cell adhesion [GO:0098609]; cell-matrix adhesion [GO:0007160]; cell-matrix adhesion involved in ameboidal cell migration [GO:0003366]; heterotypic cell-cell adhesion [GO:0034113]; immune response in gut-associated lymphoid tissue [GO:0002387]; integrin-mediated signaling pathway [GO:0007229]; leukocyte migration [GO:0050900]; leukocyte tethering or rolling [GO:0050901]; receptor clustering [GO:0043113]; substrate adhesion-dependent cell spreading [GO:0034446]; T cell migration [GO:0072678]
2649.68	3245.62	4363	2011.49	3780.85	6481.72	3165.04	1514.92	2088.41	1688.65	1749.14	4874.88	4591.94	5897.69	2317.17	3536.67	3542.1	2321.79	59820.76	Peroxisome proliferator-activated receptor gamma coactivator-related protein 1 (PGC-1-related coactivator) (PRC)	PPRC1 KIAA0595	energy homeostasis [GO:0097009]; positive regulation of transcription by RNA polymerase II [GO:0045944]

215.95	0	0	996.07	0	39.68	28681.18	7.27	0	281.89	933.21	4.72	31.28	0	0	27790.46	651.98	11.45	59645.14	Myelin transcription factor 1-like protein (MyT1-L) (MyT1L) (Neural zinc finger factor 1) (NZF-1) (Postmitotic neural gene 1 protein) (Zinc finger protein Png-1)	Myt1l Kiaa1106 Nzf1 Png1	negative regulation of transcription by RNA polymerase II [GO:0000122]; nervous system development [GO:0007399]; neuron development [GO:0048666]; neuron differentiation [GO:0030182]; neuron fate commitment [GO:0048663]; neuron fate specification [GO:0048665]; regulation of transcription by RNA polymerase II [GO:0006357]
4592.57	5146.47	0	8800.46	4993.18	34.08	37.1	2901.84	0	5674.12	7805.09	0	4828.74	2020.16	0	5257.89	7216.02	111.18	59418.9	LINE-1 retrotransposable element ORF2 protein (ORF2p) [Includes: Reverse transcriptase (EC 2.7.7.49);Endonuclease (EC 3.1.21.-)]		DNA recombination [GO:0006310]; nucleic acid metabolic process [GO:0090304]; retrotransposition [GO:0032197]
2120.86	2903.3	3241.72	2927.5	7386.68	2607.53	15.11	2292.67	4356.52	983.87	1961.29	2367.48	2445.26	8772.51	3390.84	797.33	5647.36	4943.01	59160.84	AP-4 complex accessory subunit RUSC1 (New molecule containing SH3 at the carboxy-terminus) (Nesca) (RUN and SH3 domain-containing protein 1)	RUSC1 NESCA	protein polyubiquitination [GO:0000209]
4833.38	7063.27	9045.57	2218.87	2994.28	3763.52	4351.37	1228.6	853.74	20.79	29.61	0	7625.14	10955.05	3182.88	813.56	28	0	59007.63	Translationally-controlled tumor protein homolog (TCTP)	tpt1 tct1 tctp	

51.83	69.48	17391.29	51.8	92.11	2094.47	21.19	52.04	166.65	35.18	36.69	37127.51	80.07	175.47	369.96	49.68	65.02	116.89	58047.33	Dedicator of cytokinesis protein 8	Dock8	cellular response to chemokine [GO:1990869]; dendritic cell migration [GO:0036336]; immunological synapse formation [GO:0001771]; memory T cell proliferation [GO:0061485]; negative regulation of T cell apoptotic process [GO:0070233]; positive regulation of establishment of T cell polarity [GO:1903905]; positive regulation of T cell migration [GO:2000406]; small GTPase-mediated signal transduction [GO:0007264]
2473.33	1678.92	6693.87	1238.74	2060.32	3177.05	2629.35	1314.16	5591.48	963.19	756.67	3214.76	2275.95	4715.59	6538.08	2207.22	3227.67	6441.88	57198.23	Condensin-2 complex subunit H2 (Non-SMC condensin II complex subunit H2)	ncaph2 si:dkey-202b22.2	meiotic chromosome condensation [GO:0010032]; mitotic sister chromatid separation [GO:0051306]
4981.7	9171.96	0	19293.27	10581.94	68.05	44.04	5456.27	9.96	45.18	0	0	463.83	506.43	0	1488.98	4616.78	57.65	56786.04	SLAM family member 9	Slamf9	defense response to bacterium [GO:0042742]; plasmacytoid dendritic cell chemotaxis [GO:0002410]; plasmacytoid dendritic cell differentiation [GO:0002273]
116.84	4068.87	614.26	195.76	4307.29	1832.25	5715.66	1746.17	3166.04	90.17	2220.37	2282.12	94.43	10135.99	2540.94	6352.45	8736.79	2563.38	56779.78	Solute carrier family 25 member 48	slc25a48 zgc:92090	

3439.74	2789.03	3928.81	1446.88	2760.68	3281.12	7414.88	1341.78	1921.6	755.89	1191.75	5106.22	1794.96	4750.52	1871.99	6149.1	3923.87	2740.63	56609.45	Serine/threonine-protein phosphatase 6 catalytic subunit (PP6C) (EC 3.1.3.16) (Protein phosphatase V) (PP-V)	Ppp6c Ppv	G1/S transition of mitotic cell cycle [GO:0000082]; innate immune response [GO:0045087]; negative regulation of cGAS/STING signaling pathway [GO:0160049]
1426.63	1095.51	4667.77	715.66	1258.84	2004.47	12650.91	377.86	2450.57	329.33	604.88	7295.66	2253.35	2464.42	3111.96	7096.7	1814.27	4256.23	55875.02	THAP domain-containing protein 5	THAP5	negative regulation of cell cycle [GO:0045786]
3649.21	4611.2	0	12258.25	6942.32	77.02	31.63	3020.7	0	4269.3	4784.61	0	2377.43	3950.02	0	3107.23	6690.81	88.68	55858.41	DNA repair protein XRCC3 (X-ray repair cross-complementing protein 3)	Xrcc3	double-strand break repair via homologous recombination [GO:0000724]; interstrand cross-link repair [GO:0036297]; positive regulation of mitotic cell cycle spindle assembly checkpoint [GO:0090267]; regulation of centrosome duplication [GO:0010824]; resolution of mitotic recombination intermediates [GO:0071140]; t-circle formation [GO:0090656]; telomere maintenance via recombination [GO:0000722]
2343.32	2510.85	2869.63	1842.51	3652.5	4917.05	5674.7	989.06	1819.81	1315.28	1707.15	3314.39	3874	7336.64	1755.87	5496.14	3131.83	1282.17	55832.9	Thiosulfate:glutathione sulfurtransferase (TST) (EC 2.8.1.3)	TSTD1 KAT	sulfide oxidation, using sulfide:quinone oxidoreductase [GO:0070221]

12.17	3.23	879.56	4.09	15.78	165.34	1.27	5.09	13077.72	0	23.96	4574.75	41.13	3.2	13463.1	59.12	68.89	23361.4	55759.8	LINE-1 retrotransposable element ORF2 protein (ORF2p) (Long interspersed element-1) (L1) (Retrovirus-related Pol polyprotein LINE-1) [Includes: Reverse transcriptase (EC 2.7.7.49);Endonuclease (EC 3.1.21.-)]	Pol Gm17492	DNA recombination [GO:0006310]
711.99	2703.27	4192.81	752.51	5793.72	1794.35	2652.36	3466.47	4835.28	372.13	2148.3	4094.49	1262.84	6468.74	3648.13	1664.65	4346.92	4760.55	55669.51	Protein YIF1B (YIP1-interacting factor homolog B)	yif1b zgc:103562	cilium assembly [GO:0060271]; endoplasmic reticulum to Golgi vesicle-mediated transport [GO:0006888]; protein transport [GO:0015031]; sperm flagellum assembly [GO:0120316]
135.67	0	0	756.68	0	17.94	32700.05	0.88	2.88	233.2	629.36	3.39	21.71	0	0	20095.23	714.18	21.29	55332.46	Junctional adhesion molecule A (JAM-A) (Junctional adhesion molecule 1) (JAM-1) (CD antigen CD321)	F11R JAM1	cell adhesion [GO:0007155]; establishment of endothelial intestinal barrier [GO:0090557]; intestinal absorption [GO:0050892]; regulation of membrane permeability [GO:0090559]

5.63	4.75	20233.55	5.95	0.21	1922.01	70.23	0.09	212.17	3.59	1.02	32134.71	9.32	8.87	217.3	69.93	3.49	51.97	54954.79	Titin (EC 2.7.11.1) (Connectin) (Rhabdomyosarcoma antigen MU-RMS-40.14)	TTN	cardiac muscle cell development [GO:0055013]; cardiac muscle contraction [GO:0060048]; cardiac muscle hypertrophy [GO:0003300]; cardiac muscle tissue morphogenesis [GO:0055008]; cardiac myofibril assembly [GO:0055003]; detection of muscle stretch [GO:0035995]; mitotic chromosome condensation [GO:0007076]; muscle contraction [GO:0006936]; muscle filament sliding [GO:0030049]; positive regulation of gene expression [GO:0010628]; positive regulation of protein secretion [GO:0050714]; protein kinase A signaling [GO:0010737]; response to calcium ion [GO:0051592]; sarcomere organization [GO:0045214]; sarcomerogenesis [GO:0048769]; skeletal muscle contraction [GO:0003009]; skeletal muscle myosin thick filament assembly [GO:0030241]; skeletal muscle thin filament assembly [GO:0030240]; striated muscle contraction [GO:0006941]
0	0	5.81	0	0	54886	0	0	0	0	0	0	0	0	6.71	5.31	3.38	13.24	54920.45	Collagen alpha-1(XIV) chain (Undulin)	COL14A1	cell adhesion [GO:0007155]
2368.05	2316.68	2875.23	1039	2393.25	2726.76	7654.45	1193.76	4275.77	723.41	1197.74	2271.23	2287.74	5751.51	2671.95	5927.16	3576.46	3301.27	54551.42	Arf-GAP with dual PH domain-containing protein 2 (Centaurin-alpha-2) (Cnt-a2)	Adap2 Centa2	heart development [GO:0007507]
87.13	0	4.63	337.13	0	5.63	38597.51	0	0	180.3	570.99	0	29.67	0	0	14208.85	371.59	2.71	54396.14	Large ribosomal subunit protein eL6 (60S ribosomal protein L6) (TAX-responsive enhancer element-binding protein 107) (TAXREB107)	Rpl6	cytoplasmic translation [GO:0002181]; translation at postsynapse [GO:0140242]; translation at presynapse [GO:0140236]

834.29	9229.19	447.35	189.61	10088.04	1394.99	18.78	3956.4	8.47	306.99	4158.76	576.42	889.49	16682.12	401.74	210.47	4883.27	75.78	54352.16	Rho GTPase-activating protein SYDE2 (Synapse defective protein 1 homolog 2) (Protein syd-1 homolog 2)	SYDE2	activation of GTPase activity [GO:0090630]; cell migration [GO:0016477]; regulation of Ras protein signal transduction [GO:0046578]; regulation of small GTPase mediated signal transduction [GO:0051056]; signal transduction [GO:0007165]
1405.74	6107.07	3665.35	607.45	4390.43	4022.17	7494.21	1490.2	250.49	761.98	2074.38	2165.39	2640.26	5677.86	2480.52	4496.44	3185.42	956.26	53871.62	AP-4 complex accessory subunit RUSC2 (Interacting protein of Rab1) (Iporin) (RUN and SH3 domain-containing protein 2)	RUSC2 KIAA0375	
1131.48	924.26	1003.08	966.58	708.26	1522.26	25741.73	406.72	1926.6	550.81	550.23	998.05	1450.33	1416.36	1477.76	10460.7	1104.27	1440.41	53779.89	Small ribosomal subunit protein uS5m (28S ribosomal protein S5, mitochondrial) (MRP-S5) (S5mt)	Mrps5	mitochondrial translation [GO:0032543]
335.32	9724.19	682.22	52.97	14871.63	2583.33	0.17	5094.05	6.26	166.29	2896.69	1036.89	720.03	10464.26	404.76	56.29	4385.37	53.09	53533.81	HEAT repeat-containing protein 6	heatr6 zgc:172359	
3551.64	4133.67	1334.39	928.21	4696.28	1528.61	484.19	1410.01	1285.71	665.75	1126.26	982.32	4808.01	18050.36	1346.63	2485.61	3218.59	1082.06	53118.3	Glutaminyl-peptide cyclotransferase (EC 2.3.2.5) (Glutaminyl cyclase) (QC) (Glutaminyl-tRNA cyclotransferase)	QPCT	peptidyl-pyroglutamic acid biosynthetic process, using glutaminyl-peptide cyclotransferase [GO:0017186]

1872.48	3818.87	3880.15	904.43	9025.46	4442.69	122.46	3105.55	316.47	756.55	2143.91	1712.57	3814.88	7812.79	2122.94	665.85	5021.01	1052.25	52591.31	Ankyrin repeat domain-containing protein 33B	Ankrd33b	
9562.49	128.59	88.84	2837.95	890.23	2100.73	39.86	110.4	1.08	6002.96	436.96	394.64	28496.5	95.86	140.69	844.62	182.44	17.69	52372.53	Histone-lysine N-methyltransferase SMYD3 (EC 2.1.1.354) (SET and MYND domain-containing protein 3) (Zinc finger MYND domain-containing protein 1)	Smyd3 Zmynd1	cellular response to dexamethasone stimulus [GO:0071549]; establishment of protein localization [GO:0045184]; methylation [GO:0032259]; myotube cell development [GO:0014904]; nucleosome assembly [GO:0006334]; positive regulation of transcription by RNA polymerase II [GO:0045944]
1543.17	315.96	1021.96	796.39	342.33	3982.16	14404.25	308.52	391.75	866	900.1	743.96	6733.92	2112.52	2080.02	13784.33	884.72	819.46	52031.52	Protein unc-80 homolog (mUNC-80)	Unc80 Kiaa1843	
29.78	0	3299.68	0	0	35624.84	11.75	0	0	6.71	3.36	64.48	6.81	3.5	12097.88	9.7	0	0	51158.49	CD209 antigen-like protein A (Dendritic cell-specific ICAM-3-grabbing non-integrin) (DC-SIGN) (CD antigen CD209)	Cd209a Cire	endocytosis [GO:0006897]; regulation of T cell proliferation [GO:0042129]

4367.66	2542.63	1161.74	532.95	1579.74	2455.41	27.46	1085.59	242.25	0.7	0	0	11252.26	15907.05	5904.38	905.62	2530.25	596.02	51091.71	Ceramide-1-phosphate transfer protein (CPTP) (Glycolipid transfer protein domain-containing protein 1) (GLTP domain-containing protein 1)	CPTP GLTPD1	ceramide 1-phosphate transport [GO:1902389]; ceramide transport [GO:0035627]; intermembrane lipid transfer [GO:0120009]; negative regulation of autophagy [GO:0010507]; negative regulation of interleukin-1 beta production [GO:0032691]; negative regulation of NLRP3 inflammasome complex assembly [GO:1900226]
4.27	0	6.93	0	0	50277.55	7.47	0	3.54	0	0	9.86	4.87	0	8.14	3.79	4.06	16.02	50346.5	Lumican (Keratan sulfate proteoglycan lumican) (KSPG lumican)	LUM LDC	
1513.77	4278.34	1149.37	3834.22	5405.26	1053.29	5124.8	5243.95	439.36	2013.16	4194.34	1063.89	1680.74	3606.6	615.57	3092.73	4931.09	818.83	50059.31	CCN family member 1 (Cellular communication network factor 1) (Protein CEF-10) (Protein CYR61)	CCN1 CYR61	apoptotic process involved in heart morphogenesis [GO:0003278]; atrial septum morphogenesis [GO:0060413]; atrioventricular valve morphogenesis [GO:0003181]; cell adhesion [GO:0007155]; cell-cell adhesion [GO:0098609]; chondroblast differentiation [GO:0060591]; extracellular matrix organization [GO:0030198]; integrin-mediated signaling pathway [GO:0007229]; intussusceptive angiogenesis [GO:0002041]; negative regulation of apoptotic process [GO:0043066]; osteoblast differentiation [GO:0001649]; positive regulation of apoptotic process [GO:0043065]; positive regulation of BMP signaling pathway [GO:0030513]; positive regulation of bone mineralization [GO:0030501]; positive regulation of cartilage development [GO:0061036]; positive regulation of cell differentiation [GO:0045597]; positive regulation of cell migration [GO:0030335]; positive regulation of cell-substrate adhesion
15431.23	824.19	1175.33	4799.67	1068.08	3721.22	41.79	717.6	328.86	3381.39	1033.33	1448.52	10666.81	1469.56	718.03	562.2	1850.15	348.64	49586.6	Protein MON2 homolog (Protein SF21)	MON2 KIAA1040 SF21	Golgi to endosome transport [GO:0006895]; protein transport [GO:0015031]

72.98	145.45	9423.4	27	553.18	3923.89	156.94	112.44	107.43	41.18	260.26	31807.77	21.47	771.66	482.59	252.62	444.28	113.13	48717.67	Hemicentin-1 (Fibulin-6) (FIBL-6)	HMCN1 FIBL6	actin cytoskeleton organization [GO:0030036]; basement membrane organization [GO:0071711]; cell division [GO:0051301]; heterophilic cell-cell adhesion via plasma membrane cell adhesion molecules [GO:0007157]; homophilic cell adhesion via plasma membrane adhesion molecules [GO:0007156]; response to bacterium [GO:0009617]; visual perception [GO:0007601]
2300.56	2645	1903.21	1947.13	2988.15	3527.53	5317.49	985.63	1229.66	1369.42	1654.34	3199.43	3394.22	5709.05	1368.94	4126.41	3108.99	1368.56	48143.72	Echinoderm microtubule-associated protein-like 6 (EMAP-6) (Echinoderm microtubule-associated protein-like 5-like)	EML6 EML5L	
18.02	9.59	962.01	9.69	14.23	109.56	0	0	16668.25	0	0	3647.06	114.35	43.99	11423.98	3.26	6.87	15070.94	48101.8	Poly(rC)-binding protein 4 (Alpha-CP4)	PCBP4	negative regulation of mRNA splicing, via spliceosome [GO:0048025]; regulation of transcription by RNA polymerase II [GO:0006357]

658.42	289.3	239.53	709.91	564.81	586.8	20572.93	338.37	374.78	432.52	591.03	227.41	2095.12	776.35	494.9	17546.86	921.12	494.31	47914.47	SAP domain-containing ribonucleoprotein (Nuclear protein Hcc-1)	SARNP HCC1	mRNA export from nucleus [GO:0006406]; poly(A)+ mRNA export from nucleus [GO:0016973]; regulation of translation [GO:0006417]
1490.97	2912.93	2907.95	1035.08	3478.34	3061.62	5890.94	1156.63	2075.37	751.8	1345.37	3312.8	2077.45	4139.46	2179.07	3552.13	2201.45	3024.1	46593.46	Phosphatase and actin regulator 2	Phactr2	actin cytoskeleton organization [GO:0030036]
3717.54	2875.16	5346.97	1211.81	3246.28	3350.9	1345.08	1503.81	2675.75	932.58	802.31	2784.84	2436.82	3719.81	3284.2	1362.32	2305.76	3390.52	46292.46	Protein phosphatase 1 regulatory subunit 12A (MBSP) (Myosin phosphatase-targeting subunit 1) (Myosin phosphatase target subunit 1) (Protein phosphatase myosin-binding subunit) (Protein phosphatase subunit 1M) (PP-1M) (Serine/threonine protein phosphatase PP1 smooth muscle regulatory subunit M110)	Ppp1r12a Mbs Mypt1	cellular response to xenobiotic stimulus [GO:0071466]; centrosome cycle [GO:0007098]; mitotic cell cycle [GO:0000278]; negative regulation of catalytic activity [GO:0043086]; neuron projection morphogenesis [GO:0048812]; positive regulation of transcription by RNA polymerase II [GO:0045944]; protein dephosphorylation [GO:0006470]; regulation of cell adhesion [GO:0030155]; regulation of nucleocytoplasmic transport [GO:0046822]; signal transduction [GO:0007165]

1277.07	1129.93	4625.09	863.06	2214.83	4136.39	56.07	564.27	2849.2	814.41	1055.77	2118.6	1235.09	10016.85	2321.86	2282.26	5575.54	3008.16	46144.45	Golgi resident protein GCP60 (Acyl-CoA-binding domain-containing protein 3) (Golgi complex-associated protein 1) (GOCAP1) (Golgi phosphoprotein 1) (GOLPH1) (PBR- and PKA-associated protein 7) (Peripheral benzodiazepine receptor-associated protein PAP7) [Cleaved into: Golgi resident protein GCP60, N-terminally processed]	ACBD3 GCP60 GOCAP1 GOLPH1	steroid biosynthetic process [GO:0006694]
212.29	8518.72	2419.1	458.94	3956.69	2595	43.14	2541.15	3455.38	353.25	1060.48	4102.52	88.83	7066.52	2994.64	113.05	1730.48	4217.74	45927.92	Probable ATP-dependent RNA helicase DDX59 (EC 3.6.4.13) (DEAD box protein 59) (Zinc finger HIT domain-containing protein 5)	Ddx59 Znhit5	
714.49	7813.2	4880.15	213.12	5483.24	6301.05	60.61	2879.85	230.31	220.6	1453.76	563.4	687.62	6712.39	2051.38	178.14	4369.84	837.35	45650.5	Rho GTPase-activating protein 17 (Rho-type GTPase-activating protein 17) (RhoGAP interacting with CIP4 homologs protein 1) (RICH-1)	ARHGAP17 RICH1 MSTP066 MSTP110	negative regulation of small GTPase mediated signal transduction [GO:0051058]; regulation of actin cytoskeleton organization [GO:0032956]; regulation of Rac protein signal transduction [GO:0035020]; signal transduction [GO:0007165]

9868.91	123.19	61.33	2697.08	422.73	1413.78	33.39	71.17	1.71	10852.18	244.9	489.44	18183.79	51.77	69.34	916.96	100.93	16.41	45619.01	Syntaxin-binding protein 4 (Syntaxin 4-interacting protein) (STX4-interacting protein) (Synip)	STXBP4	cellular response to type II interferon [GO:0071346]; DNA damage response [GO:0006974]; insulin receptor signaling pathway [GO:0008286]; positive regulation of cell cycle G1/S phase transition [GO:1902808]; positive regulation of keratinocyte proliferation [GO:0010838]; protein stabilization [GO:0050821]; protein targeting [GO:0006605]; regulation of D-glucose transmembrane transport [GO:0010827]; regulation of insulin secretion involved in cellular response to glucose stimulus [GO:0061178]
6368.83	54.16	122.64	1877.43	106.58	512.1	0	0	5.24	11992.24	181.02	564.81	22971	0	78.99	632.25	109.45	16.95	45593.69	DNA fragmentation factor subunit beta (EC 3.-.-.-) (Caspase-activated deoxyribonuclease) (CAD) (Caspase-activated DNase) (DNA fragmentation factor 40 kDa subunit) (DFF-40)	Dffb Cad	apoptotic chromosome condensation [GO:0030263]; apoptotic DNA fragmentation [GO:0006309]; negative regulation of apoptotic DNA fragmentation [GO:1902511]

313.75	16517.95	106.25	739.23	8229.16	211.79	52.11	5302.59	55.44	209.17	1791.64	169.54	130.05	5325.81	51.75	106.06	5870.7	109	45291.99	Phosphatidylinositol 3,4,5-trisphosphate 3-phosphatase and dual-specificity protein phosphatase PTEN (EC 3.1.3.16) (EC 3.1.3.48) (EC 3.1.3.67) (Inositol polyphosphate 3-phosphatase) (EC 3.1.3.-) (Phosphatase and tensin homolog)	Pten rCG_47874	adult behavior [GO:0030534]; angiogenesis [GO:0001525]; apoptotic process [GO:0006915]; B cell proliferation [GO:0042100]; brain morphogenesis [GO:0048854]; cardiac muscle tissue development [GO:0048738]; cell migration [GO:0016477]; cell motility [GO:0048870]; cell population proliferation [GO:0008283]; cellular response to decreased oxygen levels [GO:0036294]; cellular response to electrical stimulus [GO:0071257]; cellular response to ethanol [GO:0071361]; cellular response to hypoxia [GO:0071456]; cellular response to insulin stimulus [GO:0032869]; cellular response to insulin-like growth factor stimulus [GO:1990314]; cellular response to leptin stimulus [GO:0044320]; cellular response to nerve growth factor stimulus [GO:1990090]; cellular response to sorbitol [GO:0072709]; central nervous system development [illegible]
9697.19	105.81	64.44	2360.68	638.35	1174.66	0	60.46	0.91	8492.83	461.67	372.3	21081.54	56.58	54.42	458.66	111.32	7.13	45198.95	BCL-6 corepressor-like protein 1 (BCoR-L1) (BCoR-like protein 1)	BCORL1	chromatin organization [GO:0006325]; negative regulation of transcription by RNA polymerase II [GO:0000122]
5149.4	2148.85	6319.24	1342.31	2341.38	4333.61	191.5	856.09	3103.51	3675.08	1195.13	4489.2	2823.44	1569.01	1534.33	459.55	580.53	2348.26	44460.42	L-amino-acid oxidase (EC 1.4.3.2) (Antiparasitic protein) (APP) (Serum L-amino-acid oxidase) (SR-LAAO)		amino acid catabolic process [GO:0009063]; defense response to bacterium [GO:0042742]; innate immune response [GO:0045087]

110.97	0	9.83	100.47	0	0	21047.45	0	0	349.7	836.15	5.99	16.75	0	1.35	21261.03	576.68	14.3	44330.67	72 kDa type IV collagenase (EC 3.4.24.24) (72 kDa gelatinase) (Gelatinase A) (Matrix metalloproteinase-2) (MMP-2) (TBE-1) [Cleaved into: PEX]	MMP2 CLG4A	angiogenesis [GO:0001525]; blood vessel maturation [GO:0001955]; bone trabecula formation [GO:0060346]; cell migration [GO:0016477]; cellular response to amino acid stimulus [GO:0071230]; cellular response to estradiol stimulus [GO:0071392]; cellular response to fluid shear stress [GO:0071498]; cellular response to interleukin-1 [GO:0071347]; cellular response to reactive oxygen species [GO:0034614]; cellular response to UV-A [GO:0071492]; collagen catabolic process [GO:0030574]; endodermal cell differentiation [GO:0035987]; ephrin receptor signaling pathway [GO:0048013]; extracellular matrix disassembly [GO:0022617]; extracellular matrix organization [GO:0030198]; face morphogenesis [GO:0060325]; heart development [GO:0007507]; intramembranous ossification [GO:0001957]; luteinization [GO:0001553]; macrophage chemotaxis [GO:0048246]; negative regulation of cell adhesion [GO:0007162]; negative
4670.87	6479.71	4.77	6267.26	3870	43.94	22.72	2718.75	0	3785.2	4785.14	0	1835.12	2549.17	2.86	2214.74	5036.84	39.44	44326.53	Anoctamin-3 (Transmembrane protein 16C)	Ano3 Tmem16c	calcium activated galactosylceramide scrambling [GO:0061591]; calcium activated phosphatidylcholine scrambling [GO:0061590]; detection of mechanical stimulus [GO:0050982]; detection of temperature stimulus [GO:0016048]; establishment of localization in cell [GO:0051649]
718.6	1174.21	4189.44	460.44	1454.41	1922.98	2346.79	583.12	6385.92	354.23	467.88	6122.9	851.23	1526.63	5759.56	1351.83	712.06	7895.52	44277.75	DBF4-type zinc finger-containing protein 2	ZDBF2 KIAA1571	genomic imprinting [GO:0071514]

2431.11	2651.01	2576.53	588.26	6409.98	1819.83	788.19	1468.65	176.5	528.94	5330.85	1786.81	707.51	9899.09	464.11	427.09	5456.64	270.97	43782.07	Peroxisome biogenesis factor 10 (EC 2.3.2.27) (Peroxin-10) (Peroxisomal biogenesis factor 10) (Peroxisome assembly protein 10)	Pex10	cellular response to reactive oxygen species [GO:0034614]; protein import into peroxisome matrix, receptor recycling [GO:0016562]; protein polyubiquitination [GO:0000209]
4607.57	7304.6	3518.23	791.21	7223.24	2501.33	0	32.81	43.11	239.21	325.3	60.17	44.47	76.01	12.01	3415.97	9839.43	3698.3	43732.97	Protocadherin-10	PCDH10 KIAA1400	cell adhesion [GO:0007155]; homophilic cell adhesion via plasma membrane adhesion molecules [GO:0007156]; nervous system development [GO:0007399]
208.38	58.05	3444.8	16.76	49.03	658.82	1697.66	3.19	12063.67	60.53	23.65	4033.93	192.19	64.08	8841.22	1044.63	63.61	11195.07	43719.27	Myosin-7 (Myosin heavy chain 7) (Myosin heavy chain slow isoform) (MyHC-slow) (Myosin heavy chain, cardiac muscle beta isoform) (MyHC-beta)	Myh7	adult heart development [GO:0007512]; ATP metabolic process [GO:0046034]; cardiac muscle contraction [GO:0060048]; cardiac muscle hypertrophy in response to stress [GO:0014898]; muscle filament sliding [GO:0030049]; regulation of heart rate [GO:0002027]; regulation of slow-twitch skeletal muscle fiber contraction [GO:0031449]; regulation of the force of heart contraction [GO:0002026]; regulation of the force of skeletal muscle contraction [GO:0014728]; skeletal muscle contraction [GO:0003009]; transition between fast and slow fiber [GO:0014883]; ventricular cardiac muscle tissue morphogenesis [GO:0055010]

3.66	14077.21	4.2	0	4648.05	54.79	0	4592.95	14.16	0.51	2104.27	9.25	11.11	10947.8	46.24	2.47	6889.04	45.59	43451.3	DnaJ homolog subfamily C member 2	dnajc2 zgc:85671	'de novo' cotranslational protein folding [GO:0051083]; positive regulation of DNA-templated transcription [GO:0045893]; regulation of translational fidelity [GO:0006450]
504.66	5633.15	1474.06	471.23	6413.9	1458.32	2398.44	3713.06	347.35	263.99	2326.48	1148.47	1034.15	8329.21	1036.39	1551.75	3839.05	719.78	42663.44	V-set domain-containing T-cell activation inhibitor 1	Vtcn1	adaptive immune response [GO:0002250]; negative regulation of apoptotic process [GO:0043066]; negative regulation of T cell activation [GO:0050868]; negative regulation of T cell proliferation [GO:0042130]; positive regulation of interleukin-2 production [GO:0032743]; positive regulation of T cell proliferation [GO:0042102]; regulation of cytokine production [GO:0001817]; response to protozoan [GO:0001562]; T cell receptor signaling pathway [GO:0050852]
191.61	4509.55	272.22	91.36	12995.7	663.39	3.58	8350.04	77.75	197.32	3754.7	299.36	440.99	6964.16	133.93	55.54	3127.17	512.47	42640.84	Phosphatidylinositol N-acetylglucosaminyltransferase subunit C (Phosphatidylinositol-glycan biosynthesis class C protein) (PIG-C)	PIGC GPI2	GPI anchor biosynthetic process [GO:0006506]

1294.39	3998.92	3104.03	841.64	4733.15	3043.01	2596.65	1999.95	927.67	697.84	1199.3	3525.11	1245.5	6053.07	1532.64	2543.06	1939.71	1252.31	42527.95	Semaphorin-3F (Sema III/F) (Semaphorin IV) (Sema IV)	SEMA3F	axon extension involved in axon guidance [GO:0048846]; axon guidance [GO:0007411]; branchiomotor neuron axon guidance [GO:0021785]; facial nerve structural organization [GO:0021612]; negative chemotaxis [GO:0050919]; negative regulation of axon extension involved in axon guidance [GO:0048843]; nerve development [GO:0021675]; neural crest cell migration [GO:0001755]; neural crest cell migration involved in autonomic nervous system development [GO:1901166]; positive regulation of cell migration [GO:0030335]; regulation of postsynapse organization [GO:0099175]; semaphorin-plexin signaling pathway [GO:0071526]; sympathetic ganglion development [GO:0061549]; sympathetic neuron projection extension [GO:0097490]; sympathetic neuron projection guidance [GO:0097491]; trigeminal nerve structural organization [GO:0021637]; ventral trunk neural
1703.8	2802.14	4181.27	1156.11	1476.58	5791.83	13.63	3412.41	3749.77	2372.39	1140.13	2194.29	2151.85	2059.8	2651.36	634.24	2523.94	2304.74	42320.28	Xylulose kinase (Xylulokinase) (EC 2.7.1.17)	Xylb	D-xylose metabolic process [GO:0042732]; xylulose metabolic process [GO:0005997]
1493.01	4540.89	1723.82	2476.6	3933.24	1963.93	2146.74	2770.53	471.67	1098.33	1859.49	1632.1	2672.74	4407.37	934.4	2331.54	4665.58	1030.68	42152.66	Ankyrin repeat domain-containing protein 34C	Ankrd34c	
57.74	0	0	340.23	0	6.67	25233.48	0	0	242.42	682.72	1.12	24.56	0	0	15122.52	400.37	24.89	42136.72	UDP-glucuronosyltransferase 2B15 (UDPGT 2B15) (UGT2B15) (EC 2.4.1.17) (HLUG4) (UDP-glucuronosyltransferase 2B8) (UDPGT 2B8) (UDPGTh-3)	UGT2B15 UGT2B8	estrogen metabolic process [GO:0008210]; steroid metabolic process [GO:0008202]; xenobiotic metabolic process [GO:0006805]

3968.45	2667.92	1383.86	1020.76	4157.82	4108.44	41.39	2759.6	184.38	1672.51	1899.71	1263.62	5988.75	5576.3	1556.79	609.79	2718.6	530.03	42108.72	E3 ubiquitin-protein ligase TRAF7 (EC 2.3.2.-) (EC 2.3.2.27) (RING finger and WD repeat-containing protein 1) (RING finger protein 119) (RING-type E3 ubiquitin transferase TRAF7) (TNF receptor-associated factor 7)	TRAF7 RFWD1 RNF119	apoptotic process [GO:0006915]; positive regulation of apoptotic signaling pathway [GO:2001235]; positive regulation of MAPK cascade [GO:0043410]; positive regulation of neuron apoptotic process [GO:0043525]; positive regulation of ubiquitin-dependent protein catabolic process [GO:2000060]; protein K29-linked ubiquitination [GO:0035519]; protein ubiquitination [GO:0016567]; regulation of ERK1 and ERK2 cascade [GO:0070372]
939.61	1997.46	2429.29	702.19	1498.25	6765.95	795.2	1050.98	1112.59	1413.11	1953.83	4406.17	703.92	5357.11	2408.28	2419.11	3947.27	1843.95	41744.27	Voltage-gated potassium channel subunit beta-1 (EC 1.1.1.-) (K(+) channel subunit beta-1) (Kv-beta-1)	KCNAB1 KVB1.1	regulation of potassium ion transmembrane transport [GO:1901379]
4051.49	3621.95	1230	790.15	6187.44	1721.71	1004.68	871.95	561.52	554.42	1949.66	824.01	775.73	683.6	155.96	922.38	15237.21	567.86	41711.72	Diacylglycerol kinase theta (DAG kinase theta) (EC 2.7.1.107) (EC 2.7.1.93) (Diglyceride kinase theta) (DGK-theta)	Dgkq	adenylate cyclase-activating G protein-coupled receptor signaling pathway [GO:0007189]; cellular response to bile acid [GO:1903413]; diacylglycerol metabolic process [GO:0046339]; epidermal growth factor receptor signaling pathway [GO:0007173]; lipid phosphorylation [GO:0046834]; negative regulation of gene expression [GO:0010629]; phosphatidic acid biosynthetic process [GO:0006654]; phospholipase C-activating G protein-coupled receptor signaling pathway [GO:0007200]; positive regulation of gene expression [GO:0010628]; positive regulation of protein kinase C signaling [GO:0090037]; regulation of cholesterol metabolic process [GO:0090181]; regulation of cortisol biosynthetic process [GO:2000064]; regulation of gluconeogenesis [GO:0006111]; regulation of progesterone biosynthetic process [GO:2000182]; regulation of synaptic vesicle endocytosis [GO:1900242]; regulation of TORC1 signaling [GO:1903432]; regulation of [illegible]
2742.98	6429.34	3257.54	830.65	2810.27	4606.71	32.88	1884.59	1601.52	960.23	898.64	1501.43	1674.14	4901.81	2000.44	321.54	2643.28	2378.33	41476.32	Transmembrane protein 134	TMEM134	

121.53	5722.65	526.55	102.96	9895.61	255.5	1841.28	3953.83	45.87	48.86	3527.6	1975.06	80.34	9080.94	86.98	321.85	3474.25	82.14	41143.8	F-box/LRR-repeat protein 3 (F-box and leucine-rich repeat protein 3A) (F-box/LRR-repeat protein 3A)	FBXL3 FBL3A FBXL3A	entrainment of circadian clock by photoperiod [GO:0043153]; protein destabilization [GO:0031648]; protein ubiquitination [GO:0016567]; regulation of circadian rhythm [GO:0042752]; rhythmic process [GO:0048511]; SCF-dependent proteasomal ubiquitin-dependent protein catabolic process [GO:0031146]
12.81	18538.75	249.66	31.24	7413.93	52.02	11.06	1936.08	4.75	29.75	2001.23	13	4.01	7227.71	1.09	10.53	3576.79	19.91	41134.32	TBC1 domain family member 9 (TBC1 domain family member 9A)	TBC1D9 KIAA0882 TBC1D9A	
754.07	65.12	1991.32	325.07	86.08	22531.89	14.4	12.95	18.78	104.88	20.99	88.56	298.02	82.3	14277.5	45.81	40.33	7.04	40765.11	HLA class II histocompatibility antigen, DR alpha chain (MHC class II antigen DRA)	HLA-DRA HLA-DRA1	adaptive immune response [GO:0002250]; antigen processing and presentation of endogenous peptide antigen via MHC class II [GO:0002491]; antigen processing and presentation of exogenous peptide antigen via MHC class II [GO:0019886]; antigen processing and presentation of peptide or polysaccharide antigen via MHC class II [GO:0002504]; cognition [GO:0050890]; immune response [GO:0006955]; myeloid dendritic cell antigen processing and presentation [GO:0002469]; peptide antigen assembly with MHC class II protein complex [GO:0002503]; positive regulation of CD4-positive, alpha-beta T cell activation [GO:2000516]; positive regulation of CD4-positive, CD25-positive, alpha-beta regulatory T cell differentiation [GO:0032831]; positive regulation of immune response [GO:0050778]; positive regulation of memory T cell differentiation [GO:0043382]; positive regulation of T cell activation [GO:0050870]; positive

0.65	1.68	38396.1	1.3	1.05	1158.58	13.32	0	6.55	3.55	0.48	94.65	1.74	29.67	920	11.93	0.18	9.34	40650.77	Death-inducer obliterator 1 (DIO-1) (Death-associated transcription factor 1) (DATF-1)	Dido1 Datf1 Dio1	apoptotic signaling pathway [GO:0097190]; DNA-templated transcription [GO:0006351]
2084.06	2808	2127.48	3011.61	2636.97	4833.17	420.49	1372.65	1342.14	3130.12	2574.73	5236.48	2502.94	1889.17	1474.28	738.15	1216.68	960.42	40359.54	E3 ubiquitin-protein ligase RNF6 (EC 2.3.2.27)	RNF6 SPG2	axon extension [GO:0048675]; negative regulation of axon extension [GO:0030517]; positive regulation of DNA-templated transcription [GO:0045893]; protein K27-linked ubiquitination [GO:0044314]; protein K48-linked ubiquitination [GO:0070936]; protein K6-linked ubiquitination [GO:0085020]; protein ubiquitination [GO:0016567]; regulation of androgen receptor signaling pathway [GO:0060765]; regulation of DNA-templated transcription [GO:0006355]; ubiquitin-dependent protein catabolic process [GO:0006511]
194.4	129.69	103.41	183	114.69	240.57	19803.94	42.95	187.72	154.17	458.68	78.5	262.64	156.77	185.1	17355.78	472.53	152.45	40276.99	Carboxypeptidase E (CPE) (EC 3.4.17.10) (Carboxypeptidase H) (CPH) (Enkephalin convertase) (Prohormone-processing carboxypeptidase)	cpe	peptide metabolic process [GO:0006518]; protein processing [GO:0016485]

9099.12	70.89	34.98	2371.4	214.82	1329.56	57.6	17.35	0	6788.49	140.11	369.25	18846.9	54.07	45.97	673.1	58.8	1.27	40173.68	Charged multivesicular body protein 4c (Chromatin-modifying protein 4c) (CHMP4c)	chmp4c chmp4b zgc:55566	cilium assembly [GO:0060271]; late endosome to vacuole transport via multivesicular body sorting pathway [GO:0032511]; midbody abscission [GO:0061952]; mitotic cytokinesis checkpoint signaling [GO:0044878]; negative regulation of cytokinesis [GO:0032466]; protein transport [GO:0015031]; vesicle budding from membrane [GO:0006900]
172.6	11250.65	2352.45	246.75	3079.19	1199.01	73.21	1748.52	169.34	270.65	1237.51	1506.09	251.44	12906.27	430.88	59.93	2991.95	219	40165.44	Zinc finger protein 512B	ZNF512B KIAA1196	negative regulation of miRNA transcription [GO:1902894]
0	1.16	23214.82	0	0	1129.7	14.17	0	191.78	0.36	0	15341.55	0	0	81.81	31.35	0	41.3	40048	Kinesin-1 heavy chain (Conventional kinesin heavy chain) (Ubiquitous kinesin heavy chain) (UKHC)	Kif5b Khcs Kns1	anterograde axonal protein transport [GO:0099641]; cellular response to type II interferon [GO:0071346]; centrosome localization [GO:0051642]; cytoplasm organization [GO:0007028]; lysosome localization [GO:0032418]; microtubule-based process [GO:0007017]; mitochondrial transport [GO:0006839]; mitochondrion transport along microtubule [GO:0047497]; mitocytosis [GO:0160040]; natural killer cell mediated cytotoxicity [GO:0042267]; plus-end-directed vesicle transport along microtubule [GO:0072383]; positive regulation of potassium ion transport [GO:0043268]; positive regulation of protein localization to plasma membrane [GO:1903078]; positive regulation of synaptic transmission, GABAergic [GO:0032230]; regulation of membrane potential [GO:0042391]; regulation of modification of synapse structure, modulating synaptic transmission [GO:0098987]; stress granule disassembly [GO:0035617]; vesicle [illegible]

3882.32	4883.45	616.72	906.77	3532.67	1221.21	1287.96	4996.12	500.2	844.48	1230.25	2645.02	2208.93	1545.57	601.59	985.72	7401.89	565.52	39856.39	WD repeat domain phosphoinositide-interacting protein 1 (WIPI-1) (Atg18 protein homolog) (WD40 repeat protein interacting with phosphoinositides of 49 kDa) (WIPI 49 kDa)	WIPI1 WIPI49	autophagosome assembly [GO:0000045]; autophagy [GO:0006914]; autophagy of mitochondrion [GO:0000422]; cellular response to starvation [GO:0009267]; glycophagy [GO:0061723]; nucleophagy [GO:0044804]; pexophagy [GO:0000425]; positive regulation of autophagosome assembly [GO:2000786]; protein localization to phagophore assembly site [GO:0034497]; vesicle targeting, trans-Golgi to endosome [GO:0048203]
3937.06	274.5	6265.08	1906.08	896.03	1615.25	568.17	296.87	3900.01	1796.06	511.47	4031.78	1987.42	199.83	3565.16	791.41	807.95	6442.73	39792.86	Cytospin-B (Sperm antigen with calponin homology and coiled-coil domains 1)	Specc1 Cytsb Kiaa4061	associative learning [GO:0008306]; blastocyst development [GO:0001824]
3231.72	3922.62	2837.73	708.72	2359.53	3230.09	396.6	1136.39	376.88	726.2	978.03	1792.48	2979.05	6267.76	2378.48	1665.98	3767.41	1000.61	39756.28	A-kinase anchor protein 9 (AKAP-9) (Protein kinase A-anchoring protein 9) (PRKA9)	Akap9 Kiaa0803	maintenance of centrosome location [GO:0051661]; microtubule nucleation [GO:0007020]; protein-containing complex localization [GO:0031503]; regulation of Golgi organization [GO:1903358]; Sertoli cell development [GO:0060009]; signal transduction [GO:0007165]; spermatogenesis [GO:0007283]

3079.43	2187.24	3379.41	1182.26	2550.94	5226.97	2471.71	1379.19	1247.12	925.53	795.55	2658.72	2302.92	2806.26	1716.62	1976.91	1991.86	1774.09	39652.73	Calpain-3 (EC 3.4.22.54) (Calcium-activated neutral proteinase 3) (CANP 3) (Calpain L3) (Calpain p94) (Muscle-specific calcium-activated neutral protease 3)	Capn3	calcium-dependent self proteolysis [GO:1990092]; cellular response to calcium ion [GO:0071277]; cellular response to salt stress [GO:0071472]; G1 to G0 transition involved in cell differentiation [GO:0070315]; muscle structure development [GO:0061061]; myofibril assembly [GO:0030239]; negative regulation of apoptotic process [GO:0043066]; negative regulation of DNA-templated transcription [GO:0045892]; negative regulation of protein sumoylation [GO:0033234]; negative regulation of skeletal muscle cell differentiation [GO:2001015]; positive regulation of DNA-templated transcription [GO:0045893]; positive regulation of proteolysis [GO:0045862]; positive regulation of release of sequestered calcium ion into cytosol [GO:0051281]; positive regulation of satellite cell activation involved in skeletal muscle regeneration [GO:0014718]; programmed cell death [GO:0012501]; protein catabolic process [GO:0030163]; protein
0	0	618.82	2.48	0	12.92	0	0	13006.49	1.99	0	5226.53	0	4.66	5814.69	0.52	13.08	14609.27	39311.45	Transmembrane protein 198	tmem198	Wnt signaling pathway [GO:0016055]
1.34	73.03	3787.85	0	136.18	1054.48	19.96	91.07	18.96	67.41	144.86	33376.35	44.39	49.91	198.57	38.88	0	151.61	39254.85	Acyl-coenzyme A thioesterase 8 (Acyl-CoA thioesterase 8) (EC 3.1.2.1) (EC 3.1.2.11) (EC 3.1.2.2) (EC 3.1.2.3) (EC 3.1.2.5) (Choloyl-coenzyme A thioesterase) (EC 3.1.2.27) (Peroxisomal acyl-coenzyme A thioester hydrolase 1) (PTE-1) (Peroxisomal long-chain acyl-CoA thioesterase 1)	Acot8 Pte1	acyl-CoA metabolic process [GO:0006637]; dicarboxylic acid catabolic process [GO:0043649]; fatty acid catabolic process [GO:0009062]; negative regulation of glycoprotein biosynthetic process [GO:0010561]; peroxisome fission [GO:0016559]

59.68	7603.33	0	112.34	7942.99	24.68	18.4	2955.25	1.36	417.98	2981.37	0	1.97	11786.64	2.19	153.3	5037.83	21.93	39121.24	LIM and senescent cell antigen-like-containing domain protein 1 (Particularly interesting new Cys-His protein 1) (PINCH-1) (Renal carcinoma antigen NY-REN-48)	LIMS1 PINCH PINCH1	cell-cell adhesion [GO:0098609]; cell-cell junction organization [GO:0045216]; cellular response to transforming growth factor beta stimulus [GO:0071560]; establishment of protein localization [GO:0045184]; negative regulation of DNA-templated transcription [GO:0045892]; positive regulation of canonical NF-kappaB signal transduction [GO:0043123]; positive regulation of cell-substrate adhesion [GO:0010811]; positive regulation of focal adhesion assembly [GO:0051894]; positive regulation of gene expression [GO:0010628]; positive regulation of GTPase activity [GO:0043547]; positive regulation of integrin-mediated signaling pathway [GO:2001046]; positive regulation of substrate adhesion-dependent cell spreading [GO:1900026]; tumor necrosis factor-mediated signaling pathway [GO:0033209]
4143.53	1225.59	3341.62	1347.44	3384.66	3638.64	49.16	1283.94	869.26	4015.87	1698.3	2605.64	2853.12	2920.06	1819.7	682.87	1791.89	1330.49	39001.78	Serine/threonine-protein phosphatase with EF-hands 2 (PPEF-2) (EC 3.1.3.16)	PPEF2	cellular response to hydrogen peroxide [GO:0070301]; detection of stimulus involved in sensory perception [GO:0050906]; negative regulation of apoptotic signaling pathway [GO:2001234]; negative regulation of MAPK cascade [GO:0043409]; protein dephosphorylation [GO:0006470]; visual perception [GO:0007601]
488.9	8391.3	46.69	1201.24	6049.49	60.86	9.69	2154.29	5.03	571.33	3459.7	512.56	233.92	9957.87	32.71	402.42	5255.4	55.44	38888.84	Carnosine N-methyltransferase (EC 2.1.1.22)	CARNMT1 C9orf41	carnosine metabolic process [GO:0035498]; L-histidine catabolic process [GO:0006548]; methylation [GO:0032259]

12.15	1.1	1929.2	1.86	0.81	29.04	1.07	0.95	11686.07	2.9	2.63	3463.97	10.22	7.85	9941.94	4.69	7.49	11645.62	38749.56	CUGBP Elav-like family member 2 (CELF-2) (Bruno-like protein 3) (CUG triplet repeat RNA-binding protein 2) (CUG-BP2) (CUG-BP- and ETR-3-like factor 2) (ELAV-type RNA-binding protein 3) (ETR-3) (RNA-binding protein BRUNOL-3)	celf2 brunol3 cugbp2 etr3	mRNA processing [GO:0006397]
19.03	23.7	13890.74	2.72	3.93	20869.96	79.15	3.33	11.13	0	2.41	3160.6	4.38	109.58	283.57	46.98	16.04	16.63	38543.88	F-actin-uncapping protein LRRC16A (CARMIL homolog) (Capping protein regulator and myosin 1 linker protein 1) (Capping protein, Arp2/3 and myosin-I linker homolog 1) (Capping protein, Arp2/3 and myosin-I linker protein 1) (Leucine-rich repeat-containing protein 16A)	CARMIL1 CARMIL LRRC16 LRRC16A	actin filament network formation [GO:0051639]; actin filament organization [GO:0007015]; barbed-end actin filament uncapping [GO:0051638]; cell migration [GO:0016477]; lamellipodium assembly [GO:0030032]; macropinocytosis [GO:0044351]; negative regulation of barbed-end actin filament capping [GO:2000813]; positive regulation of actin filament polymerization [GO:0030838]; positive regulation of cell migration [GO:0030335]; positive regulation of lamellipodium organization [GO:1902745]; positive regulation of stress fiber assembly [GO:0051496]; positive regulation of substrate adhesion-dependent cell spreading [GO:1900026]; regulation of Arp2/3 complex-mediated actin nucleation [GO:0034315]; ruffle organization [GO:0031529]; urate metabolic process [GO:0046415]

507.3	1660.31	4561.71	733.63	2238.23	16390.68	18.66	617.95	64.25	630.92	1286.28	2979.03	1769.42	1504.01	1840.9	493.03	1153.44	63.76	38513.51	Claudin-5	Cldn5	bicellular tight junction assembly [GO:0070830]; calcium-independent cell-cell adhesion via plasma membrane cell-adhesion molecules [GO:0016338]; cell adhesion [GO:0007155]; cell-cell junction assembly [GO:0007043]; establishment of blood-retinal barrier [GO:1990963]; establishment of endothelial barrier [GO:0061028]; maintenance of blood-brain barrier [GO:0035633]; myelination [GO:0042552]; negative regulation of angiogenesis [GO:0016525]; negative regulation of cell migration [GO:0030336]; negative regulation of complement-dependent cytotoxicity [GO:1903660]; negative regulation of gene expression [GO:0010629]; negative regulation of vascular permeability [GO:0043116]; positive regulation of bicellular tight junction assembly [GO:1903348]; positive regulation of cell population proliferation [GO:0008284]; positive regulation of establishment of endothelial barrier [GO:1903142]; positive regulation of gene expression
0	6157.87	0	0	1004.74	6.86	0	3266.59	0	0	4503.92	0	0	19653.91	0.58	0	3462.74	10.05	38067.26	P2Y purinoceptor 14 (P2Y14) (UDP-glucose receptor)	P2RY14	G protein-coupled receptor signaling pathway [GO:0007186]
1625.76	1438.58	713.23	5685.6	2814.99	1286.48	7.85	1089.1	84	5960.42	7227.37	362.62	1383.13	1286.98	102.42	1542	4994.08	373.23	37977.84	TIMELESS-interacting protein	tipin zgc:91928	cell cycle phase transition [GO:0044770]; cell division [GO:0051301]; DNA replication checkpoint signaling [GO:0000076]; mitotic intra-S DNA damage checkpoint signaling [GO:0031573]; positive regulation of cell population proliferation [GO:0008284]; replication fork arrest [GO:0043111]; replication fork processing [GO:0031297]

1801.28	2386.13	2715.07	1869.61	3813.83	4351.57	63.98	1407.82	340.12	797.87	878.11	1319.44	5028.53	4535.86	2678.46	492.93	1478.93	1724.04	37683.58	Cyclin-dependent kinase 14 (EC 2.7.11.22) (Cell division protein kinase 14) (Serine/threonine-protein kinase PFTAIRE-1)	Cdk14 Kiaa0834 Pftk1	cell division [GO:0051301]; G2/M transition of mitotic cell cycle [GO:0000086]; regulation of canonical Wnt signaling pathway [GO:0060828]; Wnt signaling pathway [GO:0016055]
2254.42	3787.75	263.92	7136.05	6515.18	106.19	3.18	2838.78	18.55	2081.77	3325.06	768.45	1158.84	2517.01	2.19	1339.62	3496.3	45.74	37659	Tropomodulin-3 (Ubiquitous tropomodulin) (U-Tmod)	TMOD3	actin filament organization [GO:0007015]; erythrocyte development [GO:0048821]; mitotic cell cycle phase transition [GO:0044772]; muscle contraction [GO:0006936]; myofibril assembly [GO:0030239]; pointed-end actin filament capping [GO:0051694]; positive regulation of mitotic cell cycle phase transition [GO:1901992]

1096.14	3473.72	995.6	5110.63	5975.21	2068.2	800.33	2430.65	140.4	857.88	1541.47	929.2	1131.35	3620.54	289.19	1724.8	5135.07	326.64	37647.02	LINE-1 retrotransposable element ORF1 protein (L1-ORF1p) (LINE retrotransposable element 1) (LINE1 retrotransposable element 1) (Transposase element L1Md-A101/L1Md-A102/L1Md-A2)	Lire1	retrotransposition [GO:0032197]
465.73	9977.13	287.69	166.1	5773	307.54	6.43	3652.95	175.3	112.24	1706.52	195.12	773.63	8870.22	277.98	134.86	4409.23	355.25	37646.92	NK-tumor recognition protein (NK-TR protein) (Natural-killer cells cyclophilin-related protein) (Peptidyl-prolyl cis-trans isomerase NKTR) (PPIase) (EC 5.2.1.8) (Rotamase)	NKTR	protein folding [GO:0006457]
1635.72	1995.31	2107.26	1504.26	2326.26	3528.53	3170.13	996.69	692.51	1006.43	1189.88	2716.41	2889.18	3979.8	1147.38	3185.89	2462.98	1021.5	37556.12	TBC1 domain family member 14	TBC1D14 KIAA1322	autophagy [GO:0006914]; negative regulation of autophagy [GO:0010507]; recycling endosome to Golgi transport [GO:0071955]; regulation of autophagosome assembly [GO:2000785]

3807.67	692.45	3262.8	717.86	514.92	1579.14	64.82	172.26	5432.55	1343.36	460.34	4416.88	3502.06	722.99	5728.91	370.38	472.87	4260.64	37522.9	Ras association domain-containing protein 1 (Protein 123F2)	Rassf1	DNA damage response [GO:0006974]; positive regulation of protein ubiquitination [GO:0031398]; protein stabilization [GO:0050821]; Ras protein signal transduction [GO:0007265]; regulation of cell cycle [GO:0051726]; regulation of cell cycle G1/S phase transition [GO:1902806]
0	0	1226.54	1.87	0	15.35	0	0	11130.96	0	0	2938.8	3	0.59	10375.69	2.35	1.64	11596.68	37293.47	Dynamin-1 (EC 3.6.5.5) (B-dynamin) (D100) (Dynamin I) (Dynamin, brain)	Dnm1 Dnm	clathrin coat assembly involved in endocytosis [GO:0099049]; endocytosis [GO:0006897]; endosome organization [GO:0007032]; G protein-coupled receptor internalization [GO:0002031]; modulation of chemical synaptic transmission [GO:0050804]; positive regulation of synaptic vesicle endocytosis [GO:1900244]; positive regulation of synaptic vesicle recycling [GO:1903423]; protein homooligomerization [GO:0051260]; protein homotetramerization [GO:0051289]; receptor internalization [GO:0031623]; receptor-mediated endocytosis [GO:0006898]; regulation of synaptic vesicle endocytosis [GO:1900242]; regulation of vesicle size [GO:0097494]; response to amyloid-beta [GO:1904645]; synaptic vesicle budding from presynaptic endocytic zone membrane [GO:0016185]; synaptic vesicle endocytosis [GO:0048488]; vesicle scission [GO:0099050]
2019.34	1390.4	2250.26	915.03	1328.73	2704.64	6591.32	598.02	2599.63	998.99	777.01	1954.16	2082.31	1842.89	2450.84	3927.87	1010.25	1835.97	37277.66	UPF0606 protein KIAA1549L	KIAA1549L C11orf41 C11orf69	

95.98	7099.95	587.33	179.84	4258.91	354.28	260.38	4218.57	650.15	43.2	1478.57	364.23	101.28	9231.26	729.69	571.46	5955.13	978.97	37159.18	Ubiquitin carboxyl-terminal hydrolase 38 (EC 3.4.19.12) (Deubiquitinating enzyme 38) (HP43.8KD) (Ubiquitin thioesterase 38) (Ubiquitin-specific-processing protease 38)	USP38 KIAA1891	negative regulation of innate immune response [GO:0045824]; negative regulation of proteasomal ubiquitin-dependent protein catabolic process [GO:0032435]; protein K33-linked deubiquitination [GO:1990168]; regulation of protein stability [GO:0031647]; ubiquitin-dependent protein catabolic process [GO:0006511]
0	0	913.15	3.19	1.23	17.39	0	2.37	9551.33	1.53	0	3624.34	5.95	4.98	10360.52	0.67	7.01	12652.54	37146.2	Ubiquitin-conjugating enzyme E2 K (EC 2.3.2.23) (E2 ubiquitin-conjugating enzyme K) (Huntingtin-interacting protein 2) (HIP-2) (Ubiquitin carrier protein) (Ubiquitin-conjugating enzyme E2-25 kDa) (Ubiquitin-conjugating enzyme E2(25K)) (Ubiquitin-conjugating enzyme E2-25K) (Ubiquitin-protein ligase)	UBE2K HIP2	protein K48-linked ubiquitination [GO:0070936]; protein polyubiquitination [GO:0000209]
1103.47	1137.4	1383.79	2133.86	2977.26	3354.32	1682.14	966.45	1448.33	1050.27	1032.96	2645.34	2788.21	4712.71	1394.18	2243.2	3049.47	2028.52	37131.88	Nebulette (Actin-binding Z-disk protein)	Nebl Lnebl	

520.52	298.84	137.62	215.13	29.07	48.99	17354.85	22.43	3.9	299.9	735.18	18.12	532.5	46.52	62.76	15396.15	369.28	27.58	36119.34	2',3'-cyclic-nucleotide 3'-phosphodiesterase (CNP) (CNPase) (EC 3.1.4.37)	Cnp Cnp1	adult locomotory behavior [GO:0008344]; axonogenesis [GO:0007409]; cyclic nucleotide catabolic process [GO:0009214]; oligodendrocyte differentiation [GO:0048709]; response to toxic substance [GO:0009636]
2947.68	1974.16	7933.7	469.73	1320.88	1946.76	141.26	1275.85	4160.67	1295.8	1359.63	5258.21	995.98	1436.03	1083.44	590.5	802.92	1112	36105.2	U5 small nuclear ribonucleoprotein 40 kDa protein (U5 snRNP 40 kDa protein) (WD repeat-containing protein 57)	Snrnp40 Wdr57	mRNA processing [GO:0006397]; RNA splicing [GO:0008380]
6860.09	163.5	96.23	2066.72	1460.45	2690.94	27.68	130.9	2.8	5482.81	780.75	310.87	15277.66	70.85	65.81	307.75	201.74	14.17	36011.72	O-phosphoseryl-tRNA(Sec) selenium transferase (EC 2.9.1.2) (Selenocysteine synthase) (Sec synthase) (Selenocysteinyl-tRNA(Sec) synthase) (Sep-tRNA:Sec-tRNA synthase) (SepSecS) (Soluble liver antigen/liver pancreas antigen-like) (UGA suppressor tRNA-associated protein homolog)	sepsecs sla/lpl zgc:55980	conversion of seryl-tRNAsec to selenocys-tRNAsec [GO:0001717]; selenocysteine incorporation [GO:0001514]

1174.74	330.45	2142.86	369.12	686.25	2891.92	8766.11	121.47	3468.02	555.98	444.06	1461.12	1285.05	1185.42	3115.71	4351.34	685.57	2576.02	35611.21	RING finger protein 122	Rnf122	negative regulation of mitochondrial membrane potential [GO:0010917]; positive regulation of apoptotic process [GO:0043065]; proteasome-mediated ubiquitin-dependent protein catabolic process [GO:0043161]; protein autoubiquitination [GO:0051865]
7805	83.29	129.42	1772.43	384.2	636.88	18.79	46.57	70.94	8374.57	185.71	321.62	14995.54	39.78	72.93	451.92	60.9	42.77	35493.26	Dedicator of cytokinesis protein 3 (Modifier of cell adhesion) (Presenilin-binding protein) (PBP)	DOCK3 KIAA0299 MOCA	neurotrophin TRK receptor signaling pathway [GO:0048011]; regulation of small GTPase mediated signal transduction [GO:0051056]; small GTPase-mediated signal transduction [GO:0007264]

2644.06	1939.09	3053.09	811.04	2501.18	5381.2	63.1	1611.81	329.09	1251.86	930.82	1653.26	4600.79	2724.65	2354.86	540.02	1558.55	1465.85	35414.32	E3 ubiquitin-protein ligase rnf213-beta (EC 2.3.2.27) (EC 3.6.4.-) (E3 ubiquitin-lipopolysaccharide ligase rnf213-beta) (EC 2.3.2.-) (Mysterin-B) (Mysterin-beta) (RING finger protein 213-B) (RING finger protein 213-beta)	rnf213b	angiogenesis [GO:0001525]; defense response to bacterium [GO:0042742]; immune system process [GO:0002376]; lipid droplet formation [GO:0140042]; lipid ubiquitination [GO:0120323]; protein K63-linked ubiquitination [GO:0070534]; regulation of lipid metabolic process [GO:0019216]; xenophagy [GO:0098792]
4111.73	1170.75	2668.67	1042.86	1853.41	4283.73	382.68	899.51	413.14	986.38	725.44	1966.9	4017.84	4350.2	2127.92	1332.87	1924.87	1074.37	35333.27	1-acyl-sn-glycerol-3-phosphate acyltransferase gamma (EC 2.3.1.51) (1-acylglycerol-3-phosphate O-acyltransferase 3) (1-AGP acyltransferase 3) (1-AGPAT 3) (Lysophosphatidic acid acyltransferase gamma) (LPAAT-gamma)	Agpat3 Lpaat3	CDP-diacylglycerol biosynthetic process [GO:0016024]
1360.07	3204.8	2763.28	978.12	4293.46	2952.63	47.55	1522.76	1542.21	688.87	1866.86	2215.89	1210.14	3268.43	1451.44	404.82	4390.6	1078.17	35240.1	Transcription factor COE3 (Early B-cell factor 3) (EBF-3) (Olf-1/EBF-like 2) (O/E-2) (OE-2)	Ebf3 Coe3	positive regulation of DNA-templated transcription [GO:0045893]; positive regulation of transcription by RNA polymerase II [GO:0045944]

50.7	0.73	5.32	179.71	1.36	7.6	20334.33	1.07	7.61	101.23	378.94	0	24.97	0.87	2.56	13527.58	309.95	9.41	34943.94	Mothers against decapentaplegic homolog 1 (MAD homolog 1) (Mothers against DPP homolog 1) (SMAD family member 1) (SMAD 1) (Smad1)	smad1 madh1	anatomical structure morphogenesis [GO:0009653]; BMP signaling pathway [GO:0030509]; cell differentiation [GO:0030154]; dorsal/ventral pattern formation [GO:0009953]; embryonic pattern specification [GO:0009880]; myeloid cell development [GO:0061515]; negative regulation of DNA-templated transcription [GO:0045892]; regulation of DNA-templated transcription [GO:0006355]; regulation of transcription by RNA polymerase II [GO:0006357]; SMAD protein signal transduction [GO:0060395]; transforming growth factor beta receptor signaling pathway [GO:0007179]
4943.89	144.95	78.77	2027.64	906.86	3036.99	11.06	268.74	0	5037.02	241.1	205.24	16695.28	48.19	76.96	817.57	221.73	47.02	34809.01	Ubiquitin-conjugating enzyme E2Q-like protein 1 (EC 2.3.2.23) (E2Q-like ubiquitin-conjugating enzyme 1)	Ube2ql1	protein ubiquitination [GO:0016567]
70.19	9.78	18.39	162.01	2.73	14.52	23341.36	15.7	13.93	180.57	340.28	2.35	30.12	0	13.86	10134.84	283.66	16.6	34650.89	NudC domain-containing protein 1	nudcd1 zgc:110705	
8158.78	91.97	3538	397.93	77.08	5351.21	16.88	79.31	99.56	625.68	38.41	12419.92	3007.15	87.87	172.35	169.6	143.02	140.67	34615.39	Sodium/potassium-transporting ATPase subunit alpha-1 (Na(+)/K(+) ATPase alpha-1 subunit) (EC 7.2.2.13) (Sodium pump subunit alpha-1)	atp1a1	intracellular potassium ion homeostasis [GO:0030007]; intracellular sodium ion homeostasis [GO:0006883]; potassium ion import across plasma membrane [GO:1990573]; proton transmembrane transport [GO:1902600]; sodium ion export across plasma membrane [GO:0036376]

105.56	103.1	31.45	247.58	233.16	148.33	2.31	109.39	0	331.42	180.35	18	31715.88	342.24	505.8	278.5	221.52	14.72	34589.31	Zinc finger BED domain-containing protein DAYSLEEPER (Transposase-like protein DAYSLEEPER)	HAT At3g42170 T27B3.40	post-embryonic development [GO:0009791]
1890.73	1705.23	2532.85	320.18	1092.47	3715.74	468.87	383.61	5294.43	442.88	420.12	3304.81	889.49	1503.29	3907.53	401.36	550.28	5707.86	34531.73	Glycerophosphocholine phosphodiesterase GPCPD1 (EC 3.1.4.2) (Glycerophosphodiester phosphodiesterase 5)	GPCPD1 GDE5 KIAA1434	glycerophospholipid catabolic process [GO:0046475]; skeletal muscle tissue development [GO:0007519]
11.93	19.77	34261.39	2.86	10.28	26.31	0.23	18.28	35.08	5.85	6.2	23.62	7.68	31.14	32.46	0.45	6.54	10.96	34511.03	Next to BRCA1 gene 1 protein (Neighbor of BRCA1 gene 1 protein)	NBR1	macroautophagy [GO:0016236]
2360.54	4157.16	1403.05	709.58	3254.32	1888.59	1571.02	1842.17	779.66	704.08	1069.24	1765.09	3256.82	2483.35	1202.86	1477.48	3635.75	937.1	34497.86	PDZ domain-containing protein 7	PDZD7 PDZK7	auditory receptor cell development [GO:0060117]; auditory receptor cell stereocilium organization [GO:0060088]; detection of mechanical stimulus involved in sensory perception of sound [GO:0050910]; establishment of protein localization [GO:0045184]

183.96	171.36	364	472.42	133.41	213.77	15260.02	44.41	336.41	157.34	273.75	559.89	1076.14	735	1176.31	12386.34	510.33	405.98	34460.84	Phosphoribosyl pyrophosphate synthase-associated protein 1 (PRPP synthase-associated protein 1) (39 kDa phosphoribosypyrophosphate synthase-associated protein) (PAP39)	PRPSAP1	5-phosphoribose 1-diphosphate biosynthetic process [GO:0006015]; nucleobase-containing compound metabolic process [GO:0006139]; purine nucleotide biosynthetic process [GO:0006164]
2473.62	1308.27	3737.47	1132.99	1493.86	3489.67	37.68	852.13	4337.15	750.19	473.32	2033.66	621.06	1592.63	3578.92	118.09	994.21	5362.63	34387.55	Neuronal membrane glycoprotein M6-a (M6a)	Gpm6a M6a	neural retina development [GO:0003407]; neuron migration [GO:0001764]; neuron projection morphogenesis [GO:0048812]; positive regulation of filopodium assembly [GO:0051491]; regulation of synapse organization [GO:0050807]; response to bacterium [GO:0009617]; stem cell differentiation [GO:0048863]; synapse assembly [GO:0007416]
2.28	11206.72	19.09	1.29	5583.52	7.77	1.35	1660.81	7.01	11.27	2755.26	49.36	4.46	8872.24	9.02	6.81	4138.2	12.45	34348.91	Protein DENND6A (DENN domain-containing protein 6A)	DENND6A FAM116A	positive regulation of cell-cell adhesion mediated by cadherin [GO:2000049]

173.73	10161.19	203.54	21.46	4796.5	272.35	7.64	2369.11	54.27	68.37	788.18	158.51	93.81	13098.61	168.36	57.3	1414.87	361.06	34268.86	CCN family member 2 (Cellular communication network factor 2) (Connective tissue growth factor) (Connective tissue growth-related protein)	Ccn2 Ctgf	angiogenesis [GO:0001525]; calcium ion transmembrane import into cytosol [GO:0097553]; cartilage condensation [GO:0001502]; cell adhesion [GO:0007155]; cell migration [GO:0016477]; cell-matrix adhesion [GO:0007160]; cellular response to fatty acid [GO:0071398]; cellular response to glucose stimulus [GO:0071333]; cellular response to interleukin-13 [GO:0035963]; chondrocyte differentiation [GO:0002062]; chondrocyte proliferation [GO:0035988]; connective tissue development [GO:0061448]; DNA biosynthetic process [GO:0071897]; extracellular matrix constituent secretion [GO:0070278]; fibroblast growth factor receptor signaling pathway [GO:0008543]; integrin-mediated signaling pathway [GO:0007229]; intracellular signal transduction [GO:0035556]; lung development [GO:0030324]; microtubule depolymerization [GO:0007019]; negative regulation of
2017.88	3542.26	1284.56	716.1	3966.44	3672.21	26.05	2252.16	239.05	905.5	1400.75	828.83	3215.79	4351.08	1346.01	410.63	3101.55	863.44	34140.29	Talin-1	TLN1 TLN	cell-cell adhesion [GO:0098609]
2.27	12195.2	0	0	2560.94	2.46	0	4668.7	0	0	2047.35	0.67	0	5285.45	0	0	7334.44	1.27	34098.75	B-cell receptor CD22 (Sialic acid-binding Ig-like lectin 2) (Siglec-2) (CD antigen CD22)	CD22 SIGLEC2	B cell activation [GO:0042113]; cell adhesion [GO:0007155]; negative regulation of B cell receptor signaling pathway [GO:0050859]; negative regulation of calcium-mediated signaling [GO:0050849]; negative regulation of immunoglobulin production [GO:0002638]; regulation of B cell proliferation [GO:0030888]; regulation of endocytosis [GO:0030100]

0.19	6855.41	0.3	0	1836	8.44	0	1895.11	0	0.8	1575.56	1.35	0.79	16044.74	0	0	5813.89	22.07	34054.65	Neural-cadherin (Cadherin-N) (dN-cadherin)	CadN CG7100	axon extension [GO:0048675]; axon extension involved in axon guidance [GO:0048846]; axon guidance [GO:0007411]; axon target recognition [GO:0007412]; axonal fasciculation [GO:0007413]; calcium-dependent cell-cell adhesion via plasma membrane cell adhesion molecules [GO:0016339]; cell-cell adhesion [GO:0098609]; cell-cell adhesion mediated by cadherin [GO:0044331]; homophilic cell adhesion via plasma membrane adhesion molecules [GO:0007156]; negative regulation of dendrite morphogenesis [GO:0050774]; ommatidial rotation [GO:0016318]; R7 cell development [GO:0045467]; R8 cell development [GO:0045463]; regulation of axon extension involved in axon guidance [GO:0048841]; regulation of dendrite morphogenesis [GO:0048814]; retinal ganglion cell axon guidance [GO:0031290]
2670.59	3700.98	1249.79	862.57	3155.24	1331.79	380.18	1468.16	1717.65	809.45	1111.99	1540.5	2548.87	4014.34	1287.61	867.08	3498.95	1637.26	33853	Gamma-adducin (Adducin-like protein 70) (Protein kinase C-binding protein 35H)	Add3	barbed-end actin filament capping [GO:0051016]; positive regulation of cytoskeleton organization [GO:0051495]; positive regulation of vasoconstriction [GO:0045907]; response to xenobiotic stimulus [GO:0009410]
884.91	627.48	750.05	812.19	725.51	1049.65	11540.8	438.31	207.92	433.91	470.09	477.44	2645.91	1446.33	784.23	8903.97	940.18	677.63	33816.51	G protein-coupled receptor kinase 3 (EC 2.7.11.15) (Beta-adrenergic receptor kinase 2) (Beta-ARK-2)	GRK3 ADRBK2	desensitization of G protein-coupled receptor signaling pathway [GO:0002029]; G protein-coupled receptor signaling pathway [GO:0007186]

6077.93	69.56	999.29	4091.7	275.68	11714.63	15.17	65.41	478.94	3383.77	162.38	2137.47	712.06	84.5	336.07	355.2	583.26	2258.21	33801.23	Folliculin (BHD skin lesion fibrofolliculoma protein) (Birt-Hogg-Dube syndrome protein)	FLCN BHD	cell proliferation involved in kidney development [GO:0072111]; cell-cell junction assembly [GO:0007043]; cellular response to amino acid starvation [GO:0034198]; cellular response to starvation [GO:0009267]; energy homeostasis [GO:0097009]; epithelial cell proliferation [GO:0050673]; ERK1 and ERK2 cascade [GO:0070371]; hemopoiesis [GO:0030097]; in utero embryonic development [GO:0001701]; intracellular signal transduction [GO:0035556]; intrinsic apoptotic signaling pathway [GO:0097193]; lysosome localization [GO:0032418]; negative regulation of brown fat cell differentiation [GO:1903444]; negative regulation of cell proliferation involved in kidney development [GO:1901723]; negative regulation of cold-induced thermogenesis [GO:0120163]; negative regulation of epithelial cell proliferation [GO:0050680]; negative regulation of ERK1 and ERK2 cascade [GO:0070373]; [illegible]
2406.84	1219.7	4227.53	796.65	1070.82	2530.51	674.34	467.77	2948.32	781.52	540.23	2122.44	2318.72	1784.27	3602.14	991.38	1183.35	4114.72	33781.25	Relaxin-3 (Insulin-like peptide INSL7) (Insulin-like peptide 7) (Prorelaxin R3) [Cleaved into: Relaxin-3 B chain;Relaxin-3 A chain]	Rln3 Insl7	
6.85	6203.02	4.3	10.22	6440.38	36.1	0.36	2817.35	0.94	7.59	2887.97	1.74	2.84	11069.18	2.6	2.04	4002.84	24.67	33520.99	Sterile alpha motif domain-containing protein 9 (SAM domain-containing protein 9)	SAMD9 C7orf5 DRIF1 KIAA2004 OEF1	endosomal vesicle fusion [GO:0034058]; innate immune response [GO:0045087]

2128.17	1633.07	1811.26	880.91	1621.41	2616.16	4640.96	1074.58	552.78	940.79	825.89	1407.67	2150.58	1666.33	702.47	5431.84	2517.08	841.71	33443.66	Conserved oligomeric Golgi complex subunit 3 (COG complex subunit 3) (Component of oligomeric Golgi complex 3)	Cog3	endoplasmic reticulum to Golgi vesicle-mediated transport [GO:0006888]; intra-Golgi vesicle-mediated transport [GO:0006891]; intracellular protein transport [GO:0006886]; retrograde vesicle-mediated transport, Golgi to endoplasmic reticulum [GO:0006890]
1252.03	824.68	475.52	495.45	954.1	962.57	12427.29	361.18	307.47	311.26	436.4	188.67	1738.17	2422.72	522.43	8235.32	1056.87	413.62	33385.75	WD repeat and FYVE domain-containing protein 1 (WD40- and FYVE domain-containing protein 1)	Wdfy1 Kiaa1435	positive regulation of toll-like receptor 3 signaling pathway [GO:0034141]; positive regulation of toll-like receptor 4 signaling pathway [GO:0034145]
0	8742.07	7.51	0.96	7267.66	41.92	0.7	2791.43	0	0.54	2693.61	32.39	0.9	4716.85	0	0	6696.33	20.66	33013.53	Nuclear body protein SP140 (Lymphoid-restricted homolog of Sp100) (LYSp100) (Nuclear autoantigen Sp-140) (Speckled 140 kDa)	SP140 LYSP100	defense response [GO:0006952]; regulation of transcription by RNA polymerase II [GO:0006357]

548.58	979.17	2355.16	76.86	575.43	1140.94	245.45	435.81	16283.59	322.39	268.43	4914.35	208.39	251.71	1285	132.51	192.15	2581.43	32797.35	Polyadenylate-binding protein 2 (PABP-2) (Poly(A)-binding protein 2) (Nuclear poly(A)-binding protein 1) (Poly(A)-binding protein II) (PABII) (Polyadenylate-binding nuclear protein 1)	Pabpn1 Pab2 Pabp2	cellular response to lipopolysaccharide [GO:0071222]; MAPK cascade [GO:0000165]; mRNA processing [GO:0006397]; positive regulation of polynucleotide adenylyltransferase activity [GO:1904247]; regulation of mRNA 3'-end processing [GO:0031440]
114.89	620.7	1357.34	17.14	431.82	714.44	4.28	218.38	9273.26	54.76	214.03	2870.62	51.19	1299.66	7448.19	205.75	784.84	7066.98	32748.27	Serine/threonine-protein kinase 10 (EC 2.7.11.1)	stk10 zgc:63495	intracellular signal transduction [GO:0035556]; protein autophosphorylation [GO:0046777]; regulation of lymphocyte migration [GO:2000401]
1677.35	1543.87	195.65	63.64	189.06	309.96	7080.32	308.37	2360.37	1237.12	810.24	2136.47	3451.02	9341.51	1652.64	34.47	154.95	83.14	32630.15	TBC1 domain family member 17	TBC1D17	autophagy [GO:0006914]; protein transport [GO:0015031]; retrograde transport, endosome to Golgi [GO:0042147]
1217.93	5928.39	608	443.71	4155.53	1760.65	808.29	2253.43	1071.04	359.86	1366.16	1278.95	1403.73	2858.83	1542.09	730.95	3217.1	1562.68	32567.32	Amphiphysin	AMPH	synaptic vesicle endocytosis [GO:0048488]

105.29	139.54	143.27	49.66	167.82	212.44	17283.63	60.19	111.43	130.47	309.07	72.45	110.74	167.52	172.34	12862.86	324.54	142.7	32565.96	Protein dispatched homolog 1 (Protein chameleon)	disp1 con	adenohypophysis development [GO:0021984]; animal organ morphogenesis [GO:0009887]; anterior/posterior pattern specification [GO:0009952]; blood circulation [GO:0008015]; embryonic cranial skeleton morphogenesis [GO:0048701]; inner ear development [GO:0048839]; muscle cell fate specification [GO:0042694]; retinal ganglion cell axon guidance [GO:0031290]; skeletal muscle tissue development [GO:0007519]; smoothened signaling pathway [GO:0007224]
1183.48	792.36	1003.68	792.71	663.85	1665.87	9544.96	268.65	1022.27	517.2	388.8	944.89	2080.27	1065.42	1096.55	7479.42	774.32	1181.75	32466.45	Neuronal growth regulator 1 (IgLON family member 4)	NEGR1 IGLON4 UNQ2433/PRO 4993	brain development [GO:0007420]; cell-cell adhesion [GO:0098609]; cholesterol homeostasis [GO:0042632]; fat cell differentiation [GO:0045444]; feeding behavior [GO:0007631]; lipid droplet formation [GO:0140042]; locomotory behavior [GO:0007626]; neuron projection morphogenesis [GO:0048812]; positive regulation of calcium-mediated signaling [GO:0050850]; positive regulation of neuron projection development [GO:0010976]; positive regulation of saliva secretion [GO:0046878]; regulation of synapse assembly [GO:0051963]; skeletal muscle organ development [GO:0060538]; social behavior [GO:0035176]

3994.56		1985.28	2068.19	2071.72	1330.87	2566.8	402.02	4282.18	4489.01	3056.09	878.95	564.36	920.97	1131.36	388.98	337	1167.97	645.21	32281.52	Period circadian protein homolog 2 (cPER2) (Circadian clock protein PERIOD 2)	PER2	circadian regulation of gene expression [GO:0032922]; circadian rhythm [GO:0007623]; entrainment of circadian clock by photoperiod [GO:0043153]; negative regulation of transcription by RNA polymerase II [GO:0000122]
2374.31		1822.14	3728.44	672.64	1679.08	2487.12	1271.84	862.98	2879.39	772.25	953.88	2119.25	1405.84	2016.28	1528.44	1154.89	2253.55	2229.41	32211.73	Growth arrest-specific protein 6 (GAS-6) (AXL receptor tyrosine kinase ligand)	GAS6 AXLLG	activation of protein kinase B activity [GO:0032148]; animal organ regeneration [GO:0031100]; apoptotic cell clearance [GO:0043277]; B cell chemotaxis [GO:0035754]; blood coagulation [GO:0007596]; calcium ion transmembrane transport [GO:0070588]; cell surface receptor signaling pathway [GO:0007166]; cell-substrate adhesion [GO:0031589]; cellular response to glucose stimulus [GO:0071333]; cellular response to growth factor stimulus [GO:0071363]; cellular response to interferon-alpha [GO:0035457]; cellular response to starvation [GO:0009267]; cellular response to vitamin K [GO:0071307]; cellular response to xenobiotic stimulus [GO:0071466]; dendritic cell differentiation [GO:0097028]; enzyme-linked receptor protein signaling pathway [GO:0007167]; extracellular matrix assembly [GO:0085029]; fibroblast apoptotic process [GO:0044346]; fusion of virus membrane with host plasma membrane

787.26		3792.89	1091	383.62	4084.01	1592.34	93.08	2215.07	574.66	300.42	1374.2	946.74	1818.3	7062.78	1367.26	335.82	3101.56	1196	32117.01	Junction-mediating and -regulatory protein	jmy zgc:77377	'de novo' actin filament nucleation [GO:0070060]; actin polymerization-dependent cell motility [GO:0070358]; Arp2/3 complex-mediated actin nucleation [GO:0034314]; cellular response to starvation [GO:0009267]; DNA repair [GO:0006281]; intrinsic apoptotic signaling pathway by p53 class mediator [GO:0072332]; positive regulation of apoptotic process [GO:0043065]
1109.44		586.01	10536.25	309.57	500.02	8145.07	27.9	177.18	232.8	346.35	238.54	4592.41	2250.87	355.73	1616.63	370.96	402.3	269.65	32067.68	Arf-GAP with GTPase, ANK repeat and PH domain-containing protein 1 (AGAP-1) (Centaurin-gamma-2) (Cnt-g2) (GTP-binding and GTPase-activating protein 1) (GGAP1)	AGAP1 CENTG2 KIAA1099	protein transport [GO:0015031]; regulation of modification of postsynaptic structure [GO:0099159]

0	2.04	7722.95	5.65	0	738.36	0	0	38.46	0	0	23385.57	0	1.23	35.16	0	0	43.64	31973.06	Glutathione-specific gamma-glutamylcyclotransferase 1 (Gamma-GCG 1) (EC 4.3.2.7) (Cation transport regulator-like protein 1)	chac1		apoptotic process [GO:0006915]; glutathione catabolic process [GO:0006751]; negative regulation of Notch signaling pathway [GO:0045746]; negative regulation of protein processing [GO:0010955]; neurogenesis [GO:0022008]; Notch signaling pathway [GO:0007219]; response to unfolded protein [GO:0006986]
1424.56	1361.49	2921.47	495.75	1485.47	1912.75	78.11	1019.79	5016.59	920.75	821.13	2508.97	1986.69	2406.33	2196.5	518.17	1572.46	3277.94	31924.92	Galectin-3 (Gal-3) (35 kDa lectin) (Carbohydrate-binding protein 35) (CBP 35) (Galactose-specific lectin 3) (IgE-binding protein) (L-34 galactoside-binding lectin) (Laminin-binding protein) (Lectin L-29) (Mac-2 antigen)	Lgals3		cell differentiation [GO:0030154]; extracellular matrix organization [GO:0030198]; innate immune response [GO:0045087]; mRNA processing [GO:0006397]; negative regulation of endocytosis [GO:0045806]; negative regulation of immunological synapse formation [GO:2000521]; negative regulation of T cell activation via T cell receptor contact with antigen bound to MHC molecule on antigen presenting cell [GO:2001189]; negative regulation of T cell receptor signaling pathway [GO:0050860]; RNA splicing [GO:0008380]; skeletal system development [GO:0001501]

1454.91	1814.38	2575.82	703.4	2158.05	3560.02	110.53	1267.1	647.89	694.06	860.52	2322.49	2722.74	3275.58	2889.2	347.58	1621.98	2855.9	31882.15	Transmembrane 9 superfamily member 2	TM9SF2	ceramide metabolic process [GO:0006672]; glycosphingolipid biosynthetic process [GO:0006688]; protein localization to membrane [GO:0072657]; regulation of heparan sulfate proteoglycan biosynthetic process [GO:0010908]
1625.71	1752.78	1589.1	1195.34	1703.72	2900.73	2974.44	693.79	764.53	947.23	1005.83	2015.77	2453.42	3059.36	1048.09	3453.49	1723.46	912.6	31819.39	Retinoschisin (X-linked juvenile retinoschisis protein homolog)	xlrs1	cell adhesion [GO:0007155]; visual perception [GO:0007601]
3.82	2048.62	27.06	11.35	4102.14	70.24	251.48	1707.29	10.2	8.06	3026.84	21.33	6.43	16917.82	8.05	109.97	3231.29	30.11	31592.1	Insulin-like growth factor 2 mRNA-binding protein 2 (IGF2 mRNA-binding protein 2) (IMP-2) (Hepatocellular carcinoma autoantigen p62) (IGF-II mRNA-binding protein 2) (VICKZ family member 2)	IGF2BP2 IMP2 VICKZ2	anatomical structure morphogenesis [GO:0009653]; cold-induced thermogenesis [GO:0106106]; CRD-mediated mRNA stabilization [GO:0070934]; energy homeostasis [GO:0097009]; mRNA transport [GO:0051028]; negative regulation of translation [GO:0017148]; nervous system development [GO:0007399]; regulation of cytokine production [GO:0001817]; RNA stabilization [GO:0043489]

24.48	0	54.34	0.73	0	0.78	16039.35	0.25	12.85	68.43	321.74	31.05	10.77	9.37	429.71	14263.18	238.71	11.85	31517.59	Caspase-6 (CASP-6) (EC 3.4.22.59) [Cleaved into: Caspase-6 subunit p18;Caspase-6 subunit p11]	CASP6	activation of innate immune response [GO:0002218]; apoptotic chromosome condensation [GO:0030263]; apoptotic DNA fragmentation [GO:0006309]; apoptotic nuclear changes [GO:0030262]; apoptotic process [GO:0006915]; hepatocyte apoptotic process [GO:0097284]; intrinsic apoptotic signaling pathway by p53 class mediator [GO:0072332]; lens fiber cell differentiation [GO:0070306]; positive regulation of apoptotic process [GO:0043065]; positive regulation of necroptotic process [GO:0060545]; positive regulation of neuron apoptotic process [GO:0043525]; protein autoprocessing [GO:0016540]; proteolysis [GO:0006508]; pyroptotic inflammatory response [GO:0070269]
1327.04	1479.81	1778.32	1387.06	1751.62	2174.67	5046.65	635.84	896.98	662.94	882.59	1387.96	2009.2	3052.21	888.1	3733.33	1470.81	772.87	31338	Inhibin beta B chain (Activin beta-B chain)	INHBB	activin receptor signaling pathway [GO:0032924]; animal organ development [GO:0048513]; cellular response to insulin stimulus [GO:0032869]; cellular response to starvation [GO:0009267]; fat cell differentiation [GO:0045444]; negative regulation of follicle-stimulating hormone secretion [GO:0046882]; negative regulation of hepatocyte growth factor production [GO:0032686]; negative regulation of insulin secretion [GO:0046676]; positive regulation of follicle-stimulating hormone secretion [GO:0046881]; positive regulation of ovulation [GO:0060279]; response to wounding [GO:0009611]

0.46	0	31219.36	0	0	0	23.79	0	3.98	0	1.16	2.71	0	0	3.41	32.82	0	2.23	31289.92	5-aminolevulinate synthase, non-specific, mitochondrial (ALAS-H) (EC 2.3.1.37) (5-aminolevulinic acid synthase 1) (Delta-ALA synthase 1) (Delta-aminolevulinate synthase 1)	alas1	erythrocyte development [GO:0048821]; hemoglobin biosynthetic process [GO:0042541]; protoporphyrinogen IX biosynthetic process [GO:0006782]; response to bile acid [GO:1903412]
1667.3	3349.8	1562.52	241.23	2156.62	1369.34	16.07	1714.33	3411.66	541.86	1137.66	1570.33	1501.08	3271.61	1887.39	331.22	2438.72	3098.89	31267.63	Protein DOP1A	DOP1A DOPEY1 KIAA1117	Golgi to endosome transport [GO:0006895]; protein transport [GO:0015031]
1686.86	1720.26	1446.57	582.88	2838.65	4615.52	22.43	1488.25	148.83	1032.88	945.94	1270.97	3812.43	3654.7	2295.05	421.16	2197.57	1036.16	31217.11	E3 ubiquitin-protein ligase UBR4 (EC 2.3.2.27) (600 kDa retinoblastoma protein-associated factor) (p600) (N-recognin-4) (Retinoblastoma-associated factor of 600 kDa) (RBAF600)	UBR4 KIAA0462 KIAA1307 RBAF600	cytoplasm protein quality control [GO:0140455]; cytoplasm protein quality control by the ubiquitin-proteasome system [GO:0071629]; endosome organization [GO:0007032]; negative regulation of fatty acid biosynthetic process [GO:0045717]; negative regulation of HRI-mediated signaling [GO:0141191]; positive regulation of autophagy [GO:0010508]; proteasome-mediated ubiquitin-dependent protein catabolic process [GO:0043161]; protein branched polyubiquitination [GO:0141198]; protein K11-linked ubiquitination [GO:0070979]; protein K27-linked ubiquitination [GO:0044314]; protein K48-linked ubiquitination [GO:0070936]; protein stabilization [GO:0050821]; response to oxidative stress [GO:0006979]; ubiquitin-dependent protein catabolic process [GO:0006511]; ubiquitin-dependent protein catabolic process via the N-end rule pathway [GO:0071596]

3346.56	3059.99	2104.71	727.43	3179.92	2801.09	36.12	1519.06	582.81	839.97	993.67	1541.45	2796.85	3281.8	1162.92	262.63	1906.91	1065.11	31209	Nucleoprotein TPR (Megator) (NPC-associated intranuclear protein) (Translocated promoter region protein)	TPR	cell division [GO:0051301]; cellular response to heat [GO:0034605]; cellular response to interferon-alpha [GO:0035457]; mitotic spindle assembly checkpoint signaling [GO:0007094]; mRNA export from nucleus [GO:0006406]; mRNA export from nucleus in response to heat stress [GO:0031990]; negative regulation of RNA export from nucleus [GO:0046832]; negative regulation of transcription by RNA polymerase II [GO:0000122]; negative regulation of translational initiation [GO:0045947]; nuclear pore organization [GO:0006999]; nucleocytoplasmic transport [GO:0006913]; positive regulation of heterochromatin formation [GO:0031453]; positive regulation of intracellular protein transport [GO:0090316]; positive regulation of mitotic cell cycle spindle assembly checkpoint [GO:0090267]; positive regulation of protein export from nucleus [GO:0046827]; positive regulation of protein import into nucleus
1722.08	890.34	731.11	791.29	1250.83	2551.68	8938.06	642.75	408.08	1078.63	923.37	1061.23	1821.17	1043.78	608.11	5016.66	1128.09	550.75	31158.01	Gamma-aminobutyric acid receptor subunit pi (GABA(A) receptor subunit pi) (GABAAR subunit pi)	Gabrp	
1187.18	1612.2	5116.35	844.01	1335.04	4710.22	55.31	421.89	195.05	1356.34	1192.06	8107.07	709.1	1364.03	545.4	772.35	1275.01	253.3	31051.91	von Willebrand factor D and EGF domain-containing protein	VWDE	

1024.98	1108.5	476.04	594.4	1010.44	1921.45	11909.32	465.75	388.75	511.09	625.8	452.17	916.47	1194.66	574.78	6924.01	556.14	266.65	30921.4	cAMP-dependent protein kinase catalytic subunit beta (PKA C-beta) (EC 2.7.11.11)	PRKACB	adenylate cyclase-modulating G protein-coupled receptor signaling pathway [GO:0007188]; high-density lipoprotein particle assembly [GO:0034380]; negative regulation of smoothened signaling pathway [GO:0045879]; negative regulation of TORC1 signaling [GO:1904262]; neural tube closure [GO:0001843]; protein phosphorylation [GO:0006468]; regulation of protein processing [GO:0070613]; renal water homeostasis [GO:0003091]; signal transduction [GO:0007165]; vascular endothelial cell response to laminar fluid shear stress [GO:0097700]
14.67	2.52	0	207.37	0	21.59	18869.62	0	0	172.7	542.79	42	0	0	5.74	10413.93	392.77	18.91	30704.61	Solute carrier family 12 member 2 (Bumetanide-sensitive sodium-(potassium)-chloride cotransporter 2) (BSC2) (Na-K-Cl cotransporter 1) (NKCC1)	slc12a2 nkcc1	ammonium transmembrane transport [GO:0072488]; cell volume homeostasis [GO:0006884]; chloride ion homeostasis [GO:0055064]; chloride transmembrane transport [GO:1902476]; ear development [GO:0043583]; inner ear morphogenesis [GO:0042472]; potassium ion homeostasis [GO:0055075]; potassium ion import across plasma membrane [GO:1990573]; sodium ion homeostasis [GO:0055078]; sodium ion transmembrane transport [GO:0035725]; swim bladder inflation [GO:0048798]

1940.34		1787.56	6628.43	326.17	1506.66	2329.3	177.09	391.78	5256.29	973.14	455.47	4333.39	519.08	95.97	1072.51	383.38	434.79	1833.11	30444.46	Rho GTPase-activating protein 7 (Deleted in liver cancer 1 protein) (DLC-1) (HP protein) (Rho-type GTPase-activating protein 7) (START domain-containing protein 12) (StARD12) (StAR-related lipid transfer protein 12)	DLC1 ARHGAP7 KIAA1723 STARD12	actin cytoskeleton organization [GO:0030036]; focal adhesion assembly [GO:0048041]; forebrain development [GO:0030900]; heart morphogenesis [GO:0003007]; hindbrain morphogenesis [GO:0021575]; intracellular signal transduction [GO:0035556]; negative regulation of cell migration [GO:0030336]; negative regulation of cell population proliferation [GO:0008285]; negative regulation of focal adhesion assembly [GO:0051895]; negative regulation of Rho protein signal transduction [GO:0035024]; negative regulation of stress fiber assembly [GO:0051497]; neural tube closure [GO:0001843]; positive regulation of execution phase of apoptosis [GO:1900119]; regulation of Rho protein signal transduction [GO:0035023]; regulation of small GTPase mediated signal transduction [GO:0051056]
646.92		1369.43	361.3	1186.05	4076.55	3476.17	1315.8	1176.03	870.09	303.51	601.19	1201.78	2439.14	4390.81	1838.69	1656.97	2170.54	1269.71	30350.68	Hexokinase-1 (EC 2.7.1.1) (Brain form hexokinase) (Hexokinase type I) (HK I) (Hexokinase-A)	HK1	canonical glycolysis [GO:0061621]; carbohydrate phosphorylation [GO:0046835]; establishment of protein localization to mitochondrion [GO:0072655]; fructose 6-phosphate metabolic process [GO:0006002]; GDP-mannose biosynthetic process [GO:0009298]; GDP-mannose biosynthetic process from mannose [GO:0061728]; glucose 6-phosphate metabolic process [GO:0051156]; glucose metabolic process [GO:0006006]; glycolytic process [GO:0006096]; inflammatory response [GO:0006954]; innate immune response [GO:0045087]; intracellular glucose homeostasis [GO:0001678]; maintenance of protein location in mitochondrion [GO:0072656]; mannose metabolic process [GO:0006013]; positive regulation of cytokine production involved in immune response [GO:0002720]; positive regulation of interleukin-1 beta production [GO:0032731]
3802.45		369.01	5908.25	1070.41	400.42	1502.04	436.46	136.75	3535.83	681.5	151.86	1558.66	2409.69	858.82	2293.7	642.8	954.39	3626.03	30339.07	Cytochrome P450 20A1 (EC 1.14.-.-)	CYP20A1 UNQ667/PRO1301	
3099.03		583.99	373.05	1793.3	2044.26	2349.8	389.88	1797.55	793.35	1204.77	544	996.76	6301.83	2092.81	2041.08	587.16	1166.36	2102.77	30261.75	Uncharacterized protein 045L	FV3-045L	

1930.76	2598.79	2839.02	821.83	1746.6	5029.99	65.46	629.44	1183.57	723.72	501.59	3688.73	1344.79	2331.55	1168.01	534.66	1537.38	1469.03	30144.92	Serine/threonine-protein phosphatase PP1-beta catalytic subunit (PP-1B) (EC 3.1.3.16)	ppp1cb TEgg049h05.1	cell division [GO:0051301]; glycogen metabolic process [GO:0005977]; regulation of cell adhesion [GO:0030155]
320.56	520.69	918.56	130.37	653.46	325.55	102.13	636.7	5.78	241.69	937.15	20003.77	737.71	2814.29	35.72	404.82	1255.62	40.39	30084.96	DENN domain-containing protein 5B (Rab6IP1-like protein)	dennd5b si:ch211-11c20.2 zgc:77218	
1133.38	1597.39	3091.19	344.34	1730.36	2021.96	5660.31	800.1	1018.94	348.09	628.68	2171.29	959.33	1969.52	1297.71	3041.49	1093.49	1078.55	29986.12	Protein FAM193A (Protein IT14)	FAM193A C4orf8 RES4-22	
0	0	29937.28	0	0	0	0	0	0	0	0.59	2.77	0	1.22	3.47	0	5.14	3.68	29954.15	Rieske domain-containing protein	Rfesd	
1194.78	1714.34	2027.06	561.31	1213.39	1393.5	1834.36	617.01	3133.98	401.63	584.44	2286.89	1416.16	2441.89	3318.9	1642.52	1183.42	2980.58	29946.16	Protein jagged-1 (Jagged1) (hJ1) (CD antigen CD339)	JAG1 JAGL1	angiogenesis [GO:0001525]; aorta morphogenesis [GO:0035909]; aortic valve morphogenesis [GO:0003180]; blood vessel remodeling [GO:0001974]; cardiac neural crest cell development involved in outflow tract morphogenesis [GO:0061309]; cardiac right ventricle morphogenesis [GO:0003215]; cardiac septum morphogenesis [GO:0060411]; cell fate determination [GO:0001709]; ciliary body morphogenesis [GO:0061073]; distal tubule development [GO:0072017]; endocardial cushion cell development [GO:0061444]; endothelial cell differentiation [GO:0045446]; hemopoiesis [GO:0030097]; inhibition of neuroepithelial cell differentiation [GO:0002085]; inner ear auditory receptor cell differentiation [GO:0042491]; keratinocyte differentiation [GO:0030216]; loop of Henle development [GO:0072070]; morphogenesis of an epithelial sheet [GO:0002011]; myoblast differentiation [GO:0045445]; negative regulation of [illegible]
145.27	5687.75	603.69	429.25	3467.46	160.08	3.08	2390.63	3560.96	224.57	1182.85	2170.22	61.86	4033.34	1764.38	56.32	1449.64	2522.62	29913.97	Cerebellar degeneration-related protein 2	CDR2 RCJMB04_19l3	

7225.13	69.49	33.13	1357.44	129.64	354.83	24.85	11.45	0.83	6161.85	104.55	275.1	13492.52	26.24	29.09	403.77	54.09	6.28	29760.28	Transcription initiation factor TFIID subunit 4 (RNA polymerase II TBP-associated factor subunit C) (TBP-associated factor 4) (Transcription initiation factor TFIID 130 kDa subunit) (TAF(II)130) (TAFII-130) (TAFII130) (Transcription initiation factor TFIID 135 kDa subunit) (TAF(II)135) (TAFII-135) (TAFII135)	TAF4 TAF2C TAF2C1 TAF4A TAFII130 TAFII135	DNA-templated transcription initiation [GO:0006352]; mRNA transcription by RNA polymerase II [GO:0042789]; ovarian follicle development [GO:0001541]; positive regulation of DNA-templated transcription [GO:0045893]; positive regulation of transcription initiation by RNA polymerase II [GO:0060261]; regulation of DNA repair [GO:0006282]; regulation of transcription by RNA polymerase II [GO:0006357]; RNA polymerase II preinitiation complex assembly [GO:0051123]; transcription by RNA polymerase II [GO:0006366]; transcription initiation at RNA polymerase II promoter [GO:0006367]
0	0	0	0	0	29716.85	5.83	0	11.18	0	0	0	0	4.42	0	8.93	6.49	6.36	29760.06	Procollagen-lysine,2-oxoglutarate 5-dioxygenase 1 (EC 1.14.11.4) (Lysyl hydroxylase 1) (LH1)	Plod1 Plod	cellular response to hormone stimulus [GO:0032870]; collagen fibril organization [GO:0030199]; epidermis development [GO:0008544]; peptidyl-lysine hydroxylation [GO:0017185]; response to hypoxia [GO:0001666]

5211.44	43.22	57.09	1099.62	215.41	996.1	14.82	22.21	5.59	3902.45	161.7	216.51	17279.49	41.74	66.02	304.49	57.01	2.09	29697	E3 ubiquitin-protein ligase pellino homolog 1 (Pellino-1) (EC 2.3.2.27) (Pellino-related intracellular-signaling molecule) (RING-type E3 ubiquitin transferase pellino homolog 1)	PELI1 PRISM	DNA repair [GO:0006281]; negative regulation of necroptotic process [GO:0060546]; negative regulation of T cell proliferation [GO:0042130]; negative regulation of TORC1 signaling [GO:1904262]; positive regulation of B cell proliferation [GO:0030890]; positive regulation of canonical NF-kappaB signal transduction [GO:0043123]; positive regulation of cytokine production [GO:0001819]; positive regulation of double-strand break repair via homologous recombination [GO:1905168]; positive regulation of protein ubiquitination [GO:0031398]; positive regulation of toll-like receptor 3 signaling pathway [GO:0034141]; positive regulation of toll-like receptor 4 signaling pathway [GO:0034145]; proteasome-mediated ubiquitin-dependent protein catabolic process [GO:0043161]; protein K48-linked ubiquitination [GO:0070936]; protein K63-linked ubiquitination [GO:0070534]; regulation of necroptotic process [GO:0060544]; regulation of Toll [illegible]
33.39	6268.79	355.37	28.84	5752.32	648.4	248.23	3251.16	59.92	29.17	2514.06	192.81	4.13	7263.67	282.7	87.74	2544.57	116.69	29681.96	Transmembrane channel-like protein 6	Tmc6 Ever1	intracellular zinc ion homeostasis [GO:0006882]; protein stabilization [GO:0050821]
2231.65	1524.98	3171.14	759.42	1847.53	5302.43	120.08	373.26	758.6	585.61	596.36	1771.3	3066.88	1865.23	2061.55	508.87	1166.25	1957.75	29668.89	E3 ubiquitin-protein ligase RNF114 (EC 2.3.2.27) (RING finger protein 114) (RING-type E3 ubiquitin transferase RNF114) (Zinc finger protein 313)	Rnf114 Zfp313 Znf313	cell differentiation [GO:0030154]; protein polyubiquitination [GO:0000209]; spermatogenesis [GO:0007283]; ubiquitin-dependent protein catabolic process [GO:0006511]

0.95	11730.72	25.96	1.83	1304.78	25.46	1.21	2881.45	8.26	0.51	1585.32	113.1	7.98	2855.33	8.09	0.67	9046.78	29.61	29628.01	Solute carrier family 25 member 47-A (Hepatocellular carcinoma down-regulated mitochondrial carrier homolog A)	slc25a47a hdmcpa zgc:136752	
503.17	1078.66	7006.96	212.08	874.69	2424.7	1008.01	583.6	2253.99	161.08	387.25	4798.87	704.63	972.24	2309.94	698.02	964.54	2640.91	29583.34	Phosphofurin acidic cluster sorting protein 1 (PACS-1)	PACS1 KIAA1175	lymphocyte homeostasis [GO:0002260]; protein localization to Golgi apparatus [GO:0034067]; protein localization to plasma membrane [GO:0072659]
1402.11	1504.15	965.8	883.44	1688.08	2929.19	6279.43	603.8	399.84	666.08	595.07	1058.98	2026.61	1056.17	975.46	4660.26	929.04	885.55	29509.06	F-box only protein 50 (NCC receptor protein 1) (NCCRP-1) (Non-specific cytotoxic cell receptor protein 1)	nccrp1 si:dkey-85K8.6 wu:fa93e02 wu:fb12f07 zgc:122987	ERAD pathway [GO:0036503]; glycoprotein catabolic process [GO:0006516]; SCF-dependent proteasomal ubiquitin-dependent protein catabolic process [GO:0031146]

1443.78	5244.74	385.36	682.53	2515.04	878.34	2139.33	2472.37	383.1	380.51	808.67	767.56	2084.98	1772.94	457.66	1619.75	4716.75	555.45	29308.86	Cadherin EGF LAG seven-pass G-type receptor 1 (Cadherin family member 9) (Flamingo homolog 2) (hFmi2)	CELSR1 CDHF9 FMI2	apical protein localization [GO:0045176]; cell-cell adhesion [GO:0098609]; central nervous system development [GO:0007417]; establishment of body hair planar orientation [GO:0048105]; establishment of planar polarity [GO:0001736]; establishment of planar polarity of embryonic epithelium [GO:0042249]; homophilic cell adhesion via plasma membrane adhesion molecules [GO:0007156]; lateral sprouting involved in lung morphogenesis [GO:0060490]; neural tube closure [GO:0001843]; neuron migration [GO:0001764]; orthogonal dichotomous subdivision of terminal units involved in lung branching morphogenesis [GO:0060488]; planar dichotomous subdivision of terminal units involved in lung branching morphogenesis [GO:0060489]; protein localization involved in establishment of planar polarity [GO:0090251]; regulation of actin cytoskeleton organization [GO:0032956]; Rho protein
1375.69	1646.97	2387.02	1047.21	1605.49	1984.63	1972.19	801.09	1390.36	744.24	1011.59	2053.54	1993.87	2744.45	1371.74	1640.22	1787.91	1693.05	29251.26	Receptor-type tyrosine-protein phosphatase zeta (R-PTP-zeta) (EC 3.1.3.48) (3F8 chondroitin sulfate proteoglycan) (3H1 keratan sulfate proteoglycan) (Phosphacan)	Ptprz1 Ptprz Ptpz	axonal fasciculation [GO:0007413]; axonogenesis [GO:0007409]; hematopoietic progenitor cell differentiation [GO:0002244]; hippocampus development [GO:0021766]; learning or memory [GO:0007611]; negative regulation of cell population proliferation [GO:0008285]; negative regulation of cell-substrate adhesion [GO:0010812]; negative regulation of dendrite development [GO:2000171]; negative regulation of neuron apoptotic process [GO:0043524]; neuron development [GO:0048666]; neuron projection development [GO:0031175]; oligodendrocyte differentiation [GO:0048709]; peptidyl-tyrosine dephosphorylation [GO:0035335]; positive regulation of cell migration [GO:0030335]; positive regulation of dendrite development [GO:1900006]; positive regulation of fibroblast proliferation [GO:0048146]; positive regulation of neuron migration [GO:2001224]; positive regulation of

5599.53	100.98	105.96	3410.42	470.8	2730.63	23.24	196.86	0	5682.47	151.39	160.51	9012.62	55.52	47	1179.17	196.66	69.64	29193.4	Lipopolysaccharide-induced tumor necrosis factor-alpha factor homolog (LPS-induced TNF-alpha factor homolog) (Small integral membrane protein of lysosome/late endosome)	LITAF SIMPLE	cytokine production [GO:0001816]; regulation of cytokine production [GO:0001817]; sexual reproduction [GO:0019953]
1421.54	783.93	1737.98	567.52	2361.02	1994.14	44.65	565.58	1835.83	605.1	280.35	955.33	4697.97	4046.09	3826.83	285.61	808.03	2221.17	29038.67	Leukocyte receptor cluster member 8 homolog	leng8 zgc:158262	
2078.22	1886.66	1641.51	651.39	1641.44	2278.36	1594.28	761.82	2267.95	751.39	774.65	1746.19	2036.7	1848.24	2065.23	1149.33	1292.72	2529.93	28996.01	snRNA-activating protein complex subunit 4 (SNAPc subunit 4) (snRNA-activating protein complex 190 kDa subunit) (SNAPc 190 kDa subunit)	Snapc4	snRNA transcription by RNA polymerase II [GO:0042795]; snRNA transcription by RNA polymerase III [GO:0042796]
4.12	0	739.06	0.86	0.47	0	0	0	7188.83	0	0.3	2692.5	9.66	0.63	7689.85	0.84	2.65	10634.01	28963.78	Leucine-rich repeat transmembrane neuronal protein 4	LRRTM4 UNQ3075/PRO9907	

1356.72	1472.7	1936.46	433.75	1098.17	1539.33	3301.83	641.75	2590.63	367.52	451.78	1334.58	1604.42	1973.55	2536.81	2292.53	1093.34	2812.62	28838.49	BAR/IMD domain-containing adapter protein 2 (Brain-specific angiogenesis inhibitor 1-associated protein 2) (BAI-associated protein 2) (BAI1-associated protein 2) (Insulin receptor substrate protein of 53 kDa) (IRSp53) (Insulin receptor substrate p53) (Insulin receptor tyrosine kinase substrate protein p53)	Baiap2	actin crosslink formation [GO:0051764]; actin filament bundle assembly [GO:0051017]; cellular response to epidermal growth factor stimulus [GO:0071364]; cellular response to L-glutamate [GO:1905232]; dendrite development [GO:0016358]; modulation of chemical synaptic transmission [GO:0050804]; neuron differentiation [GO:0030182]; plasma membrane organization [GO:0007009]; positive regulation of actin filament polymerization [GO:0030838]; positive regulation of dendritic spine morphogenesis [GO:0061003]; positive regulation of excitatory postsynaptic potential [GO:2000463]; positive regulation of neuron projection development [GO:0010976]; protein localization to synapse [GO:0035418]; regulation of actin cytoskeleton organization [GO:0032956]; regulation of cell shape [GO:0008360]; regulation of modification of postsynaptic actin cytoskeleton [GO:1905274]; regulation of postsynapse organization
6.67	9528.29	60.91	9.63	7430.47	80.67	1.38	2658.59	7.28	8.95	999.22	20.6	7.65	6409.84	10.32	101.92	1471.44	14.29	28828.12	tRNA (guanosine(18)-2'-O)-methyltransferase TARBP1 (EC 2.1.1.34) (TAR RNA-binding protein 1) (TAR RNA-binding protein of 185 kDa) (TRP-185)	TARBP1 TRM3 TRP185	regulation of transcription by RNA polymerase II [GO:0006357]; tRNA methylation [GO:0030488]
493.22	3623.18	1197.98	316.49	5203.59	928.8	628.08	2046.42	792.65	153.28	1382.33	1718.6	1091.92	5100.07	670.77	467.88	2126.61	782.31	28724.18	Protein NLRC3	Nlrc3	canonical NF-kappaB signal transduction [GO:0007249]; negative regulation of canonical NF-kappaB signal transduction [GO:0043124]; negative regulation of cytokine production involved in inflammatory response [GO:1900016]; negative regulation of epithelial cell proliferation [GO:0050680]; negative regulation of fibroblast proliferation [GO:0048147]; negative regulation of inflammatory response [GO:0050728]; negative regulation of innate immune response [GO:0045824]; negative regulation of interferon-alpha production [GO:0032687]; negative regulation of interferon-beta production [GO:0032688]; negative regulation of interleukin-1 beta production [GO:0032691]; negative regulation of interleukin-12 production [GO:0032695]; negative regulation of interleukin-6 production [GO:0032715]; negative regulation of NLRP3 inflammasome complex assembly [GO:1900226]; negative regulation of

2719.29	3581.3	2135.89	544	2999.55	1379.91	450.58	1330.3	724.44	909.03	1262.39	999.69	2242.84	2667.4	1466.96	484.16	1896.77	868.41	28662.91	Acid sphingomyelinase-like phosphodiesterase 3b (ASM-like phosphodiesterase 3b) (EC 3.1.4.-)	SMPDL3B ASML3B ASMLPD		inflammatory response [GO:0006954]; innate immune response [GO:0045087]; membrane lipid catabolic process [GO:0046466]; negative regulation of inflammatory response [GO:0050728]; negative regulation of toll-like receptor signaling pathway [GO:0034122]; sphingomyelin catabolic process [GO:0006685]
54.01	6.55	0	216.68	1.2	5.39	15152.56	0.98	3.97	103.41	303.51	2.7	6.11	2599.26	1.17	8608.53	1565.55	24.34	28655.92	RNA-directed RNA polymerase (EC 2.7.7.48) (p91)			
4698.02	60.83	64.43	2123.54	329.59	1758.5	15.26	74.94	2.3	6358.26	171.14	239.46	11933.26	37.81	51.12	513.47	76.61	16.74	28525.28	Regucalcin (RC) (Gluconolactonase) (GNL) (EC 3.1.1.17) (Senescence marker protein 30) (SMP-30) (xSMP-30)	rgn		intracellular calcium ion homeostasis [GO:0006874]; L-ascorbic acid biosynthetic process [GO:0019853]; positive regulation of ATP-dependent activity [GO:0032781]; regulation of calcium-mediated signaling [GO:0050848]

3034.41	924.66	2461.37	920.48	792.45	2530.15	672.47	324.17	1988.36	888.3	401.09	3078.99	2600.97	1133.53	1894.71	972.11	765.16	3072.21	28455.59	Importin-5 (Imp5) (Importin subunit beta-3) (Karyopherin beta-3) (Ran-binding protein 5) (RanBP5)	Ipo5 Kpnb3 Ranbp5	cellular response to amino acid stimulus [GO:0071230]; cytoplasmic pattern recognition receptor signaling pathway [GO:0002753]; NLS-bearing protein import into nucleus [GO:0006607]; positive regulation of protein import into nucleus [GO:0042307]; ribosomal protein import into nucleus [GO:0006610]	
688.45	674.92	6059.19	634.41	1410.11	1619.12	3893.12	430.78	1400.35	434.13	578.6	1372.36	1302.71	1442.53	1537.67	2524.23	754.06	1662.74	28419.48	PDZ domain-containing protein 4 (PDZ domain-containing RING finger protein 4-like protein)	PDZD4 KIAA1444 PDZK4 PDZRN4L		
5153.46	128.12	29.02	1114.69	916.87	1218.72	4.58	108.97	0	2638.25	567.65	188.4	15640.38	93.55	91.32	307.55	155	9.67	28366.2	Replication factor C subunit 5 (Activator 1 36 kDa subunit) (A1 36 kDa subunit) (Activator 1 subunit 5) (Replication factor C 36 kDa subunit) (RF-C 36 kDa subunit) (RFC36)	Rfc5	DNA-templated DNA replication [GO:0006261]; positive regulation of DNA-directed DNA polymerase activity [GO:1900264]	

1732.34	1937.45	2758.15	838.86	2126.33	2954.85	1390.74	823.56	719.4	582.88	715.03	2692.82	1902.21	2405.63	1202.96	1188.86	1232.45	967.33	28171.85	Colorectal mutant cancer protein (Protein MCC)	MCC	establishment of protein localization [GO:0045184]; negative regulation of canonical Wnt signaling pathway [GO:0090090]; negative regulation of epithelial cell migration [GO:0010633]; negative regulation of epithelial cell proliferation [GO:0050680]; signal transduction [GO:0007165]; Wnt signaling pathway [GO:0016055]
13.1	7889.98	4.19	0	5944.83	39.88	3.87	2466.17	2.18	1.78	864.29	0	3.56	8430.57	1.67	2.36	2467.94	6.58	28142.95	Cell adhesion molecule DSCAM (Down syndrome cell adhesion molecule homolog)	DSCAM	axon guidance [GO:0007411]; camera-type eye photoreceptor cell differentiation [GO:0060219]; dendrite morphogenesis [GO:0048813]; dendrite self-avoidance [GO:0070593]; dendritic spine development [GO:0060996]; homophilic cell adhesion via plasma membrane adhesion molecules [GO:0007156]; locomotory behavior [GO:0007626]; negative regulation of cell adhesion [GO:0007162]; positive regulation of axon extension involved in axon guidance [GO:0048842]; positive regulation of phosphorylation [GO:0042327]; post-embryonic retina morphogenesis in camera-type eye [GO:0060060]; retina layer formation [GO:0010842]; social behavior [GO:0035176]; synapse assembly [GO:0007416]; synaptic transmission, glutamatergic [GO:0035249]

4388.6	1255.98	634.12	1277.14	1821.16	2942.17	10.97	460.66	79.9	3301.83	941.42	1329.27	7666.53	312.06	928.81	308.17	331.91	151.98	28142.68	Teneurin-4 (Ten-4) (Protein Odd Oz/ten-m homolog 4) (Tenascin-M4) (Ten-m4) (Teneurin transmembrane protein 4)	tenm4 odz4 tnm4	axon guidance [GO:0007411]; central nervous system myelin formation [GO:0032289]; homophilic cell adhesion via plasma membrane adhesion molecules [GO:0007156]; neuron development [GO:0048666]; positive regulation of gastrulation [GO:2000543]; positive regulation of myelination [GO:0031643]; positive regulation of neuron projection development [GO:0010976]; positive regulation of oligodendrocyte differentiation [GO:0048714]; regulation of myelination [GO:0031641]; signal transduction [GO:0007165]
37.71	56.43	0	22.73	68.72	0	1.4	28.46	33.2	3.93	358.34	25.67	0	16544.49	34.18	2371.61	8353.24	72.64	28012.75	Zinc fingers and homeoboxes protein 3 (Triple homeobox protein 1) (Zinc finger and homeodomain protein 3)	Zhx3 Kiaa0395 Tix1	cell differentiation [GO:0030154]; negative regulation of DNA-templated transcription [GO:0045892]; negative regulation of transcription by RNA polymerase II [GO:0000122]; positive regulation of osteoblast differentiation [GO:0045669]

108.22	4.01	32.69	135.22	3.88	54.81	11749.63	2.37	11.48	113.28	509.19	19.89	66.64	4.83	47.61	14674.02	335.97	30.27	27904.01	Sodium/potassium-transporting ATPase subunit beta-1-interacting protein 1 (Na(+)/K(+)-transporting ATPase subunit beta-1-interacting protein 1) (Protein FAM77C)	Nkain1 Fam77c	
1160.74	140.86	4440	161.78	113.25	449.18	1305.49	28.7	3958.42	203.96	49.37	1177.01	1339.04	422.32	6310.67	809	265.62	5544.76	27880.17	Tetraspanin-9 (Tspan-9) (Tetraspan NET-5)	TSPAN9 NET5	
532.07	3290.34	2316.16	431.8	3851.15	2781.41	58.48	1936.44	527.77	445.12	930.74	1243.18	691.29	5014.05	780.81	201.28	1403.04	1398.33	27833.46	Ras-related protein R-Ras2 (EC 3.6.5.2) (Ras-like protein TC21) (Teratocarcinoma oncogene)	RRAS2 TC21	osteoblast differentiation [GO:0001649]; positive regulation of Schwann cell migration [GO:1900149]; Ras protein signal transduction [GO:0007265]; Schwann cell migration [GO:0036135]

0	7949.88	4.64	5	4293.76	19.29	1.32	2235.9	1.85	0.26	1063.87	21	0	7913.1	0.7	4.38	4276.68	11.95	27803.58	Epidermal growth factor receptor kinase substrate 8-like protein 2 (EPS8-like protein 2) (Epidermal growth factor receptor pathway substrate 8-related protein 2) (EPS8-related protein 2)	Eps8l2 Eps8r2	positive regulation of ruffle assembly [GO:1900029]; Rac protein signal transduction [GO:0016601]; regulation of Rho protein signal transduction [GO:0035023]; Rho protein signal transduction [GO:0007266]; sensory perception of sound [GO:0007605]
4030.71	828.94	1645.94	1261.95	2382.09	2347.05	146.93	1018.94	314.77	2563.5	1488.79	803.15	3370.05	2057	552.29	574.15	1852.79	545.52	27784.56	Inositol monophosphatase 1 (IMP 1) (IMPase 1) (EC 3.1.3.25) (D-galactose 1-phosphate phosphatase) (EC 3.1.3.94) (Inositol-1(or 4)-monophosphatase 1) (Lithium-sensitive myo-inositol monophosphatase A1)	IMPA1 IMPA	inositol biosynthetic process [GO:0006021]; inositol metabolic process [GO:0006020]; phosphatidylinositol phosphate biosynthetic process [GO:0046854]; signal transduction [GO:0007165]
2475.05	1776.98	372.18	1181.15	3060.23	1664.54	127.11	1792.81	273.84	1951.79	2191.12	1173.05	1565.04	2621.8	422.22	661.15	3826.96	478.52	27615.54	VPS10 domain-containing receptor SorCS1 (hSorCS)	SORCS1 SORCS	neuropeptide signaling pathway [GO:0007218]; post-Golgi vesicle-mediated transport [GO:0006892]

403.01	10.1	307.79	37.29	31.98	83.55	11.13	9.49	7374.64	20.8	15.36	3548.31	39.69	21.58	7927.75	3.46	9.07	7744.64	27599.64	Lysosome-associated membrane glycoprotein 1 (LAMP-1) (Lysosome-associated membrane protein 1) (CD107 antigen-like family member A) (CD antigen CD107a)	LAMP1	establishment of protein localization to organelle [GO:0072594]; Golgi to lysosome transport [GO:0090160]; granzyme-mediated programmed cell death signaling pathway [GO:0140507]; lysosomal lumen acidification [GO:0007042]; positive regulation of natural killer cell degranulation [GO:0043323]; positive regulation of natural killer cell mediated cytotoxicity [GO:0045954]; protein stabilization [GO:0050821]; regulation of organelle transport along microtubule [GO:1902513]
5905.71	537.7	1954.3	1620.48	1043.98	5669.41	29.87	483.79	548.44	1895.87	434.19	1167.4	3015.12	1087.49	755.98	306.43	765.11	375.72	27596.99	Ceramide kinase (hCERK) (EC 2.7.1.138) (Acylsphingosine kinase) (Lipid kinase 4) (LK4)	CERK KIAA1646	ceramide metabolic process [GO:0006672]; glycosphingolipid biosynthetic process [GO:0006688]
284.08	6998.31	306.96	109.45	5041.67	332.9	86.61	3990.81	147.85	112.82	1270.73	253.3	507.54	3633.67	268.44	185.91	3729.38	218.56	27478.99	Apoptosis-stimulating of p53 protein 2 (Bcl2-binding protein) (Bbp) (Renal carcinoma antigen NY-REN-51) (Tumor suppressor p53-binding protein 2) (53BP2) (p53-binding protein 2) (p53BP2)	TP53BP2 ASPP2 BBP	intrinsic apoptotic signaling pathway by p53 class mediator [GO:0072332]; negative regulation of cell cycle [GO:0045786]; positive regulation of execution phase of apoptosis [GO:1900119]; signal transduction [GO:0007165]
578.98	792.07	1827.12	309.84	990.92	3016.24	3222.98	409.85	2969.47	410.86	512.04	1600.27	1081.52	903.1	2388.11	2316.26	848.56	3258.03	27436.22	Anoctamin-10 (Transmembrane protein 16K)	ano10 tmem16k zgc:114140	chloride transmembrane transport [GO:1902476]

2070.43	1614.88	2360.09	767.48	1554.88	1986.58	2486.42	719.22	809.35	768.16	755.31	1373.08	2488.75	2372.16	1365.12	1814.12	1207.41	864.99	27378.43	S-adenosylmethionine synthase isoform type-1 (AdoMet synthase 1) (EC 2.5.1.6) (Methionine adenosyltransferase 1) (MAT 1)	MAT1A	methionine catabolic process [GO:0009087]; one-carbon metabolic process [GO:0006730]; S-adenosylmethionine biosynthetic process [GO:0006556]
1343.55	1488.06	1885.42	784.51	1827.31	2065.04	2255.76	795.97	1753.02	600.49	1021.52	2026.5	1121.91	2474.67	1156.5	1570.75	1556.84	1530.99	27258.81	Secretogranin-3 (Secretogranin III) (SgIII)	scg3 si:ch211-12a1.3 zgc:73236	protein localization to secretory granule [GO:0033366]
1087.65	1573.28	541.91	1022.17	1856.85	5221.21	417.25	1755.75	847.25	1789.64	1990.05	529.2	1038.76	3716.09	1608.49	587.37	831.21	824.04	27238.17	Triple functional domain protein (EC 2.7.11.1)	Trio	negative regulation of fat cell differentiation [GO:0045599]; neuron projection morphogenesis [GO:0048812]; postsynaptic modulation of chemical synaptic transmission [GO:0099170]

0	0	1016.07	0	0.97	0	0	0.33	7941.45	0	1.07	2305.76	0	0	6940.99	0.57	6.29	8949.98	27163.48	Catenin delta-2 (Delta-catenin) (GT24) (Neural plakophilin-related ARM-repeat protein) (NPRAP) (Neurojungin)	CTNND2 NPRAP	cell adhesion [GO:0007155]; cell-cell adhesion [GO:0098609]; dendritic spine morphogenesis [GO:0060997]; regulation of canonical Wnt signaling pathway [GO:0060828]; signal transduction [GO:0007165]; synapse organization [GO:0050808]; Wnt signaling pathway [GO:0016055]
1299.99	1860.93	7169.47	2023.25	2103.72	250.99	457.15	1423.04	33.26	451.8	595.34	3944.2	798.8	1831.25	46.97	1164.64	1434.92	65.24	26954.96	Intraflagellar transport protein 20 homolog	ift20 zgc:103674	cell differentiation [GO:0030154]; cilium assembly [GO:0060271]; intraciliary transport [GO:0042073]; protein localization to cilium [GO:0061512]; regulation of platelet-derived growth factor receptor-alpha signaling pathway [GO:2000583]; spermatogenesis [GO:0007283]

1972.64	1625.59	2228.95	588.65	2022.63	1959.48	884.27	673.64	2063.88	924.55	816.5	1670.25	1334.01	2028.45	1709.28	886.29	1283.14	2261.77	26933.97	Mediator of RNA polymerase II transcription subunit 7 (Cofactor required for Sp1 transcriptional activation subunit 9) (CRSP complex subunit 9) (Mediator complex subunit 7)	med7 crsp9	regulation of transcription by RNA polymerase II [GO:0006357]
21.06	5291.32	224.63	22.26	4674.44	160.88	1.13	5352.09	124.61	50.53	1902.6	148.65	14	4636.67	88.91	46.38	3914.59	207.35	26882.1	CCR4-NOT transcription complex subunit 10	CNOT10	mRNA catabolic process [GO:0006402]; negative regulation of translation [GO:0017148]
930.98	3424.5	1590.56	297.61	3293.05	2049.37	661.6	1851.7	999.62	295.67	740.02	1649.91	1115.41	3249.52	973.67	570.36	1575.42	1585.29	26854.26	IQ motif and SEC7 domain-containing protein 3 (Potential synaptic guanine nucleotide exchange factor for Arf) (SynArfGEF-Po)	Iqsec3 Sag	actin cytoskeleton organization [GO:0030036]; activation of GTPase activity [GO:0090630]; postsynapse organization [GO:0099173]; regulation of ARF protein signal transduction [GO:0032012]; regulation of small GTPase mediated signal transduction [GO:0051056]

48.98	0	0	264.05	0	1.41	14037.13	0	0	156.61	444.87	0	6	0	1.81	11604.28	285.78	2.09	26853.01	Lamin-B1	LMNB1 LMN2 LMNB	heterochromatin formation [GO:0031507]; nuclear envelope organization [GO:0006998]; nuclear migration [GO:0007097]; nuclear pore localization [GO:0051664]; protein localization to nuclear envelope [GO:0090435]
2807.16	2493.12	2162.38	519.19	1753.37	2221.05	154.95	1080.73	323.54	1047.33	562.51	1051.29	3305.89	3476.28	1116.58	364.64	1567.48	834.97	26842.46	Sodium/iodide cotransporter (Na(+)/I(-) cotransporter) (Sodium-iodide symporter) (Na(+)/I(-) symporter) (Solute carrier family 5 member 5)	Slc5a5 Nis	thyroid hormone generation [GO:0006590]
1521.93	2010.67	622.43	3483.68	1836.79	82.56	2451.82	746.51	37.61	1849.27	2210.56	1199.37	903.21	1421.55	26.92	2889.08	3458.77	47.64	26800.37	Protocadherin-19	Pcdh19 Kiaa1313	homophilic cell adhesion via plasma membrane adhesion molecules [GO:0007156]

7.67	19.88	1533.12	11.97	24.73	85.93	28.36	14.52	6389.11	9.99	23.93	2554.08	180.93	69.16	6373.29	38.54	39.66	9272.26	26677.13	ER degradation-enhancing alpha-mannosidase-like protein 2	Edem2	carbohydrate metabolic process [GO:0005975]; endoplasmic reticulum mannose trimming [GO:1904380]; ERAD pathway [GO:0036503]; mannoprotein catabolic process [GO:0006058]; positive regulation of retrograde protein transport, ER to cytosol [GO:1904154]; response to unfolded protein [GO:0006986]
427.55	609.24	1765.98	105.65	333.33	1118.3	150.6	168.37	5966.37	138.6	148.14	2886.27	520.87	816.62	5677.13	199.29	282.87	5341.39	26656.57	SH3 domain and tetratricopeptide repeat-containing protein 1	SH3TC1	
1763.63	1075.18	963.13	816.88	1195.89	1480.74	4759.04	484.87	1745.54	686.43	626.86	1350.9	1578.34	1486.93	1296.3	3273.96	825.84	1239.68	26650.14	Alanine--glyoxylate aminotransferase (AGT) (EC 2.6.1.44) (Serine--pyruvate aminotransferase, mitochondrial) (SPT) (EC 2.6.1.51)	AGXT AGT1	glycine biosynthetic process, by transamination of glyoxylate [GO:0019265]; glyoxylate metabolic process [GO:0046487]

35.74	0	0	209.61	0	7.65	12744.23	0	2.19	117.18	350.71	0	8.68	0.56	0	12846.09	300.8	8.58	26632.02	Lysophosphatidic acid receptor 1-A (LPA receptor 1-A) (LPA-1-A) (Lysophosphatidic acid receptor LPA1 homolog 1) (xLPA1-1)	lpar1-a lpa1r lpa1r1	adenylate cyclase-activating G protein-coupled receptor signaling pathway [GO:0007189]; adenylate cyclase-inhibiting G protein-coupled receptor signaling pathway [GO:0007193]; negative regulation of neuron projection development [GO:0010977]; neurogenesis [GO:0022008]; positive regulation of MAPK cascade [GO:0043410]; positive regulation of Rho protein signal transduction [GO:0035025]; positive regulation of stress fiber assembly [GO:0051496]; regulation of cell shape [GO:0008360]; regulation of metabolic process [GO:0019222]
0.8	2235.48	184.6	1.5	318.87	35.59	0.73	657.6	64.57	1.68	1933.75	123.3	0.93	2352.4	1997.09	1.18	4877.41	11788.89	26576.37	Serine/threonine-protein kinase 17A (EC 2.7.11.1) (DAP kinase-related apoptosis-inducing protein kinase 1)	STK17A DRAK1	apoptotic process [GO:0006915]; intracellular signal transduction [GO:0035556]; positive regulation of apoptotic process [GO:0043065]; positive regulation of fibroblast apoptotic process [GO:2000271]; protein phosphorylation [GO:0006468]; regulation of reactive oxygen species metabolic process [GO:2000377]

1250.54	1902.76	1234.51	883.92	2039.32	1940.8	3924.89	475.1	412.17	412.08	714.12	938.29	1680.25	2796.69	620.51	3057.13	1477.73	668.86	26429.67	Relaxin receptor 1 (Leucine-rich repeat-containing G-protein coupled receptor 7) (Relaxin family peptide receptor 1)	RXFP1 LGR7	adenylate cyclase-activating G protein-coupled receptor signaling pathway [GO:0007189]; extracellular matrix organization [GO:0030198]; hormone-mediated signaling pathway [GO:0009755]; lung connective tissue development [GO:0060427]; myofibroblast differentiation [GO:0036446]; nipple morphogenesis [GO:0060658]; parturition [GO:0007567]
1792.63	1678.57	2420.02	550.59	1367.72	2726.02	2594.78	784.93	293.46	618.25	615.3	1152.14	1741.64	2121.48	1235.3	2673.1	1513.61	527.95	26407.49	Single-strand DNA endonuclease ASTE1 (EC 3.1.-.-) (Protein asteroid homolog 1)	aste1a si:dkey-11p23.5	
473.77	1201.05	1702.64	127.94	729.28	936.44	99.44	413.51	10688.78	153.42	250.9	3610.14	156.26	310.94	729.77	231.84	372.56	4088.42	26277.1	Chromodomain-helicase-DNA-binding protein 3 (CHD-3) (EC 3.6.4.-) (ATP-dependent helicase CHD3) (Mi-2 autoantigen 240 kDa protein) (Mi2-alpha) (Zinc finger helicase) (hZFH)	CHD3	centrosome cycle [GO:0007098]; chromatin remodeling [GO:0006338]; negative regulation of DNA-templated transcription [GO:0045892]; negative regulation of transcription by RNA polymerase II [GO:0000122]; positive regulation of DNA-templated transcription [GO:0045893]; regulation of cell fate specification [GO:0042659]; regulation of DNA-templated transcription [GO:0006355]; regulation of stem cell differentiation [GO:2000736]; spindle organization [GO:0007051]

0	0	0	181.27	0	0	12649.19	0	0	42.76	447.78	0	0	0	0	12689.02	193.75	0	26203.77	Trans-1,2-dihydrobenzene-1,2-diol dehydrogenase (EC 1.3.1.20) (D-xylose 1-dehydrogenase) (D-xylose-NADP dehydrogenase) (EC 1.1.1.179) (Dimeric dihydrodiol dehydrogenase)	dhdh ch211-203b17.3 zgc:101723	
1339.46	2576.42	2148.29	572.73	2586.29	3150.74	87.73	1183.48	1038.56	615.29	691.81	1890.5	1288.35	2518.82	1253.03	311.67	1401.33	1398.29	26052.79	Mitogen-activated protein kinase kinase kinase kinase 3 (EC 2.7.11.1) (Germinal center kinase-related protein kinase) (GLK) (MAPK/ERK kinase kinase kinase 3) (MEK kinase kinase 3) (MEKKK 3)	Map4k3	intracellular signal transduction [GO:0035556]; protein phosphorylation [GO:0006468]; response to tumor necrosis factor [GO:0034612]; response to UV [GO:0009411]
364.25	597.68	2518.2	75.2	401.68	769.35	8.66	234.84	5603.32	145.67	312.24	2610.33	224.9	401.56	5011.73	57.69	183.39	6481.63	26002.32	Sodium-dependent phosphate transporter 2 (Solute carrier family 20 member 2)	slc20a2 TEgg117l18.1	phosphate ion transport [GO:0006817]
0	0	25850.12	0	0	0	10.12	0	0	0	0	0	0	0	2.42	3.75	0	2.38	25868.79	RNA polymerase II subunit A C-terminal domain phosphatase SSU72 (CTD phosphatase SSU72) (EC 3.1.3.16)	ssu72 zgc:73143	mRNA processing [GO:0006397]; termination of RNA polymerase II transcription [GO:0006369]

2.93	3.22	1754.94	0	5.98	18.86	2.51	0	6972.69	7.73	9.23	2554.79	3.3	0	6208	10.22	8.31	8274.44	25837.15	Serologically defined colon cancer antigen 8 (Antigen NY-CO-8) (Centrosomal colon cancer autoantigen protein) (hCCCAP)	SDCCAG8 CCCAP NPHP10 HSPC085	cell projection organization [GO:0030030]; centrosome cycle [GO:0007098]; establishment of cell polarity [GO:0030010]; microtubule organizing center organization [GO:0031023]; neuron migration [GO:0001764]; regulation of cilium assembly [GO:1902017]; tube formation [GO:0035148]
1092.04	6289.01	753.99	770.83	2463.93	2093.79	172.37	1357.37	293.69	401.3	848.54	747.46	716.11	4064.5	612.79	385.97	2359.27	338.07	25761.03	Testis-expressed protein 264 (Putative secreted protein Zsig11)	TEX264 ZSIG11 UNQ337/PRO536	protein-DNA covalent cross-linking repair [GO:0106300]; reticulophagy [GO:0061709]
967.24	724.39	3499.21	715.45	1164.84	1722.67	1703.67	509.53	987.68	348.95	388.33	5059.83	881.54	1658.61	1108.72	1634.39	1093.84	1560.57	25729.46	Transmembrane protein 200A	tmem200a si:ch211-45m15.5	
3944.71	1646.9	846.63	1410.45	1801.37	465.54	358.79	948.38	265.41	2981.01	837.53	499.24	4593.98	2223.34	503.61	284.04	1363.68	675.31	25649.92	E3 ubiquitin-protein ligase RNF180 (EC 2.3.2.27) (RING finger protein 180) (RING-type E3 ubiquitin transferase RNF180)	RNF180	adult behavior [GO:0030534]; norepinephrine metabolic process [GO:0042415]; positive regulation of proteasomal ubiquitin-dependent protein catabolic process [GO:0032436]; positive regulation of protein ubiquitination [GO:0031398]; protein polyubiquitination [GO:0000209]; serotonin metabolic process [GO:0042428]

2987.92	1250.02	3065.49	807.71	1391.34	2717.53	219.28	505.69	1386.41	753.3	512.97	1455.27	1810.39	1553.52	1793.52	566.63	1046.89	1626.67	25450.55	Transposable element Tcb2 transposase		DNA integration [GO:0015074]; DNA transposition [GO:0006313]
290.53	183.94	870.58	109.46	404.12	467.01	107.06	179.84	4254.35	169.26	130.23	1734.5	558.17	394.76	8068.35	136.44	253.31	7030.33	25342.24	Semaphorin-7A (CDw108) (JMH blood group antigen) (John-Milton-Hargen human blood group Ag) (Semaphorin-K1) (Sema K1) (Semaphorin-L) (Sema L) (CD antigen CD108)	SEMA7A CD108 SEMAL	axon extension [GO:0048675]; axon guidance [GO:0007411]; immune response [GO:0006955]; inflammatory response [GO:0006954]; integrin-mediated signaling pathway [GO:0007229]; negative chemotaxis [GO:0050919]; neural crest cell migration [GO:0001755]; olfactory lobe development [GO:0021988]; osteoblast differentiation [GO:0001649]; positive regulation of axon extension [GO:0045773]; positive regulation of cell migration [GO:0030335]; positive regulation of ERK1 and ERK2 cascade [GO:0070374]; positive regulation of macrophage cytokine production [GO:0060907]; regulation of inflammatory response [GO:0050727]; regulation of synapse maturation [GO:0090128]; semaphorin-plexin signaling pathway [GO:0071526]
1038.14	1346.37	1371.13	694.76	1703.54	1785.47	4088.43	754.69	957.18	652.93	622.44	1209.28	1127.31	2181.69	889.45	2808.6	957.33	1142.22	25330.96	E3 ubiquitin-protein ligase znrf2 (EC 2.3.2.27) (RING-type E3 ubiquitin transferase znrf2) (Zinc/RING finger protein 2)	znrf2 zgc:152865	proteasome-mediated ubiquitin-dependent protein catabolic process [GO:0043161]; protein K48-linked ubiquitination [GO:0070936]

1355	1755.73	2097.79	958.35	1418.56	1670.98	2544.98	726.79	467.56	538.53	832.05	1265.7	1715.06	3167.07	724.01	1982.03	1575.81	509.24	25305.24	Single-stranded DNA-binding protein 3 (Sequence-specific single-stranded-DNA-binding protein)	SSBP3 SSDP SSDP1	head morphogenesis [GO:0060323]; hematopoietic progenitor cell differentiation [GO:0002244]; mesendoderm development [GO:0048382]; midbrain-hindbrain boundary initiation [GO:0021547]; positive regulation of anterior head development [GO:2000744]; positive regulation of cell population proliferation [GO:0008284]; positive regulation of transcription by RNA polymerase II [GO:0045944]; prechordal plate formation [GO:0021501]; protein-containing complex assembly [GO:0065003]
2515.95	1671.14	1649.92	622.73	2519.69	3401.63	1174.51	1080.2	482.52	558.59	617.14	1376.46	1956.77	1447.77	1138.43	656.71	1665.14	739.55	25274.85	Transcription factor SOX-4	Sox4 Sox-4	ascending aorta morphogenesis [GO:0035910]; atrial septum primum morphogenesis [GO:0003289]; cardiac right ventricle morphogenesis [GO:0003215]; cellular response to glucose stimulus [GO:0071333]; endocrine pancreas development [GO:0031018]; gene expression [GO:0010467]; glial cell development [GO:0021782]; glial cell proliferation [GO:0014009]; glucose homeostasis [GO:0042593]; heart development [GO:0007507]; hematopoietic stem cell homeostasis [GO:0061484]; kidney morphogenesis [GO:0060993]; mesenchyme development [GO:0060485]; mitral valve morphogenesis [GO:0003183]; negative regulation of myoblast differentiation [GO:0045662]; negative regulation of transcription by RNA polymerase II [GO:0000122]; nervous system development [GO:0007399]; neuroepithelial cell differentiation [GO:0060563]; noradrenergic neuron differentiation [GO:0003357]; positive [illegible]
1093.25	2836.3	1284.11	278.31	2925.69	1918.88	1556.21	1604.64	1299.3	482.04	1238.7	1236.25	586.65	2560.59	1415.35	586.69	1209.35	1023.84	25136.15	Myosin-binding protein C, fast-type (Fast MyBP-C) (C-protein, skeletal muscle fast isoform)	MYBPC2 MYBPCF	cell adhesion [GO:0007155]; sarcomere organization [GO:0045214]

1755.36	1685.83	2939.65	495.58	1278.38	2528.05	2363.06	483	547.05	456.85	486.21	1366.46	1645.63	1736.26	704.6	2348.68	1499.48	733.99	25054.12	Tyrosine-protein phosphatase non-receptor type 11 (EC 3.1.3.48) (SH-PTP2) (cSH-PTP2)	PTPN11	cellular response to epidermal growth factor stimulus [GO:0071364]; ERBB signaling pathway [GO:0038127]; negative regulation of chondrocyte differentiation [GO:0032331]; positive regulation of ERK1 and ERK2 cascade [GO:0070374]; positive regulation of ossification [GO:0045778]
284.56	81.78	74.83	394.66	95.49	165.63	15228.26	68.17	62.86	159.33	511.93	29.54	50.26	127.53	35.56	7327.29	231.48	47.89	24977.05	Transmembrane protein 43 (Protein LUMA)	Tmem43	innate immune response [GO:0045087]; nuclear membrane organization [GO:0071763]
3291.27	1214.22	4483.49	781.38	1298.79	1581.49	46.92	558.44	1255.27	842.41	470.12	1788.24	2508.87	927.66	1316.45	302.71	709.27	1524.45	24901.45	Leucine-rich repeat and calponin homology domain-containing protein 4 (Leucine-rich repeat neuronal protein 4) (Leucine-rich neuronal protein)	LRCH4 LRN LRRN1 LRRN4	membrane raft assembly [GO:0001765]; nervous system development [GO:0007399]; positive regulation of toll-like receptor signaling pathway [GO:0034123]
59.98	1.35	0	366.33	0	6.37	15344.79	0	2.04	131.32	250.76	3.79	2.8	0	0	8459.54	227.35	6.85	24863.27	Glucoside xylosyltransferase 2 (EC 2.4.2.42) (Glycosyltransferase 8 domain-containing protein 4)	GXYLT2 GLT8D4	O-glycan processing [GO:0016266]

111.9	6548.73	4.8	74.55	5592.95	9.03	2.14	3430.01	2.84	24.25	1002.48	0	58	5424.37	7.87	6.42	2492.12	26.27	24818.73	DNA polymerase eta (EC 2.7.7.7) (RAD30 homolog A) (Xeroderma pigmentosum variant type protein homolog)	Polh Rad30a Xpv	cellular response to UV-C [GO:0071494]; DNA replication [GO:0006260]; DNA synthesis involved in DNA repair [GO:0000731]; postreplication repair [GO:0006301]; pyrimidine dimer repair [GO:0006290]
279.65	290.94	519.5	131.13	263.16	403.7	11554.07	115.6	453.24	142.05	437.04	272.48	380.06	317.37	453.95	7860.44	375.21	414.02	24663.61	Aftiphilin	Aftph Afth	intracellular transport [GO:0046907]; protein transport [GO:0015031]
482.78	703.68	1377.04	289.94	620.73	1546.43	147.33	291.35	3936.25	265.98	476.42	1740.76	800.34	1153.51	5344.45	322.61	493.09	4434.96	24427.65	Ran-binding protein 3 (RanBP3)	RANBP3 QccE-19613	protein export from nucleus [GO:0006611]
132.88	667.95	1639.03	78.26	662.43	2062.61	100.72	248.04	4050.53	81.65	232.77	1981.76	43.29	1358.38	4488.36	491.5	827.22	5031.24	24178.62	Copper-transporting ATPase 1 (EC 7.2.2.8) (Copper pump 1) (Menkes disease-associated protein)	ATP7A MC1 MNK	ATP metabolic process [GO:0046034]; blood vessel development [GO:0001568]; blood vessel remodeling [GO:0001974]; cartilage development [GO:0051216]; catecholamine metabolic process [GO:0006584]; central nervous system neuron development [GO:0021954]; cerebellar Purkinje cell differentiation [GO:0021702]; collagen fibril organization [GO:0030199]; copper ion export [GO:0060003]; copper ion import [GO:0015677]; copper ion transport [GO:0006825]; dendrite morphogenesis [GO:0048813]; detoxification of copper ion [GO:0010273]; dopamine metabolic process [GO:0042417]; elastic fiber assembly [GO:0048251]; epinephrine metabolic process [GO:0042414]; establishment of localization in cell [GO:0051649]; extracellular matrix organization [GO:0030198]; glycoprotein biosynthetic process [GO:0009101]; hair follicle morphogenesis [GO:0031069]; intracellular copper ion homeostasis [illegible]

497.09	510.14	367.7	366.12	561.55	1004.88	8585	139.14	272.37	308.62	274.29	521.13	1447.76	657.4	467.31	7166.6	620.85	337.04	24104.99	Protein KRI1 homolog	kri1 kri1l	definitive hemopoiesis [GO:0060216]; endonucleolytic cleavage in ITS1 to separate SSU-rRNA from 5.8S rRNA and LSU-rRNA from tricistronic rRNA transcript (SSU-rRNA, 5.8S rRNA, LSU-rRNA) [GO:0000447]; ribosome biogenesis [GO:0042254]
900.03	467.8	3054.06	539.23	678.11	1579.08	1684.24	282.02	1194.47	384.97	285.6	6545.11	989.3	1227.36	1009.95	1286.01	802.75	1180.97	24091.06	Sphingosine 1-phosphate receptor 3 (S1P receptor 3) (S1P3) (Sphingosine 1-phosphate receptor Edg-3) (S1P receptor Edg-3)	s1pr3 edg3	
1167.04	924.44	1624.39	365.67	750.84	1499.13	4388.97	353.37	1899.9	367.01	425.27	1063.31	994.84	1580.47	1653.82	2309.22	844.15	1853.12	24064.96	Opsin-5 (G-protein coupled receptor 136) (G-protein coupled receptor PGR12) (Neuropsin)	Opn5 Gpr136 Pgr12	cellular response to light stimulus [GO:0071482]; cellular response to UV-A [GO:0071492]; entrainment of circadian clock by photoperiod [GO:0043153]; hyaloid vascular plexus regression [GO:1990384]; phototransduction [GO:0007602]; phototransduction, UV [GO:0007604]; visual perception [GO:0007601]

458.58	2057.03	1964.82	361.79	2356.19	2959.04	6.24	724.73	1183.98	260.91	867.97	2003.71	737.78	3689.08	1550.62	251.81	1329.02	1301.28	24064.58	Neural cell adhesion molecule L1 (N-CAM-L1) (NCAM-L1) (L1-CAM)	l1cam	axon guidance [GO:0007411]; brain development [GO:0007420]; positive regulation of axon extension [GO:0045773]
1005.78	618.29	1324.6	285.01	333.35	1706.31	6855.9	234.21	563.38	257.05	255.6	1179.56	1396.31	696.19	1191.71	4320.54	631.98	1178.84	24034.61	General transcription factor IIE subunit 1 (Transcription initiation factor IIE subunit alpha) (TFIIE-alpha)	GTF2E1	transcription initiation at RNA polymerase II promoter [GO:0006367]
472.6	166.26	378.92	86.43	112.28	250.56	102.75	73.31	99.01	111.85	51.03	280.48	72.72	85.11	233.01	1510.08	4931.89	14976.48	23994.77	Lipase member H (EC 3.1.1.-)	liph zgc:91985	lipid catabolic process [GO:0016042]
1832.59	123.3	4609.23	383.07	140.86	7051.52	5.53	77.43	1196.89	399.69	89.66	700.66	820.89	534.31	4152.9	39.07	251.53	1542.91	23952.04	Chromosome-associated kinesin KIF4 (Chromokinesin) (Chromosome-associated kinesin KLP1)	kif4 kif4a klp1	microtubule-based movement [GO:0007018]; mitotic spindle organization [GO:0007052]; spindle elongation [GO:0051231]
56.97	3.14	3.37	530.08	13.44	11.73	12432.87	5.27	3.52	120.89	294.88	0	13.13	49.35	12.09	10143.57	210.39	7.36	23912.05	Zinc finger protein 181	ZNF181	regulation of transcription by RNA polymerase II [GO:0006357]

49.54	61.98	3272.92	128.94	20.03	1033.43	10493.86	11.97	84.91	117.95	221.47	2911.38	51.95	70.5	212.15	4686.82	217.02	243.93	23890.75	Histone-lysine N-methyltransferase SETD7 (EC 2.1.1.364) (SET domain-containing protein 7)	setd7 zgc:92330	heart morphogenesis [GO:0003007]; heterochromatin organization [GO:0070828]; peptidyl-lysine dimethylation [GO:0018027]; peptidyl-lysine monomethylation [GO:0018026]; positive regulation of DNA-templated transcription [GO:0045893]
1865.79	1144.15	2413.54	612.78	1961.59	3078.63	24.12	935.89	596.89	647.3	640.97	1193.33	3117.32	2062.19	1377.43	249.96	1064.82	883.51	23870.21	G-protein coupled receptor 39	GPR39	G protein-coupled receptor signaling pathway [GO:0007186]
851.02	2576.39	1651.54	173.67	2518.96	1881.8	85.36	1984.21	224.57	689.15	1522.28	1558.35	1349.25	2959.64	1355.1	151.44	1215.22	1059.82	23807.77	Xin actin-binding repeat-containing protein 2 (Beta-xin) (Cardiomyopathy-associated protein 3) (L-NAME-induced actin cytoskeletal protein)	Xirp2 Cmya3 Xin2	actin filament organization [GO:0007015]; cardiac muscle tissue morphogenesis [GO:0055008]; cell-cell junction organization [GO:0045216]; positive regulation of protein localization [GO:1903829]; regulation of actin filament organization [GO:0110053]; ventricular septum development [GO:0003281]

1362.03	1788.6	2041.3	510.88	1753.09	1676.04	1923.15	895.76	755.6	385.09	725.2	1129.85	1773.42	1872.76	1548.79	1117.06	1283.05	1125.71	23667.38	Puratrophin-1 (Pleckstrin homology domain-containing family G member 4) (PH domain-containing family G member 4) (Purkinje cell atrophy-associated protein 1)	PLEKHG4 PRTPHN1	axon guidance [GO:0007411]; regulation of small GTPase mediated signal transduction [GO:0051056]
1407.27	2909.99	1102.78	557.41	1826.83	995.29	2589.75	839.79	666.61	362.99	666.35	922.4	1848.59	1171.73	877.02	1815.28	2241.48	767.53	23569.09	Apoptotic chromatin condensation inducer in the nucleus (Acinus)	Acin1 Acinus	apoptotic chromosome condensation [GO:0030263]; apoptotic process [GO:0006915]; erythrocyte differentiation [GO:0030218]; mRNA processing [GO:0006397]; negative regulation of mRNA splicing, via spliceosome [GO:0048025]; positive regulation of apoptotic process [GO:0043065]; positive regulation of monocyte differentiation [GO:0045657]; RNA splicing [GO:0008380]
4.15	1.87	439.01	1.9	0	14.4	0	0	6365.11	1.73	1.64	2231.18	4.7	0	5886.39	3.15	7.5	8601.94	23564.67	Glycine N-methyltransferase (EC 2.1.1.20) (Folate-binding protein)	Gnmt Fbp-cII	glycine metabolic process [GO:0006544]; glycogen metabolic process [GO:0005977]; methionine metabolic process [GO:0006555]; methylation [GO:0032259]; one-carbon metabolic process [GO:0006730]; protein homotetramerization [GO:0051289]; regulation of gluconeogenesis [GO:0006111]; S-adenosylhomocysteine metabolic process [GO:0046498]; S-adenosylmethionine metabolic process [GO:0046500]; sarcosine metabolic process [GO:1901052]

1184.72	1618.16	1743.41	336.29	1766.74	1626.57	1627.58	1060.06	965.23	328.09	611.02	1172.39	2102.96	2045.47	1367.93	1386.1	1223.1	1396.33	23562.15	Large ribosomal subunit protein mL39 (39S ribosomal protein L39, mitochondrial) (L39mt) (MRP-L39) (39S ribosomal protein L5, mitochondrial) (L5mt) (MRP-L5)	MRPL39 C21orf92 MRPL5 RPML5 MSTP003 PRED22	mitochondrial translation [GO:0032543]
857.13	4109.91	443.71	1163.19	2726.57	536.9	1002.06	2342.36	158.42	577.73	1306.61	425.58	1110.83	1484.34	254.72	1037.74	3759.97	247.59	23545.36	Regulating synaptic membrane exocytosis protein 2 (Rab-3-interacting molecule 2) (RIM 2) (Rab-3-interacting protein 2)	Rims2 Rab3ip2 Rim2	adenylate cyclase-modulating G protein-coupled receptor signaling pathway [GO:0007188]; calcium ion-regulated exocytosis of neurotransmitter [GO:0048791]; calcium-ion regulated exocytosis [GO:0017156]; cell differentiation [GO:0030154]; establishment of localization in cell [GO:0051649]; insulin secretion [GO:0030073]; intracellular protein transport [GO:0006886]; neurotransmitter secretion [GO:0007269]; positive regulation of excitatory postsynaptic potential [GO:2000463]; positive regulation of gene expression [GO:0010628]; positive regulation of inhibitory postsynaptic potential [GO:0097151]; regulation of calcium-dependent activation of synaptic vesicle fusion [GO:0150037]; regulation of exocytosis [GO:0017157]; regulation of membrane potential [GO:0042391]; spontaneous neurotransmitter secretion [GO:0061669]; synaptic vesicle docking [GO:0016081]; synaptic vesicle priming
717.47	2751.41	187.4	150.93	2041.83	229.38	11.74	2593.06	334.62	274.13	1521.4	373.05	1332.93	5631.61	311.96	128.15	4497.02	320.07	23408.16	RNA-binding protein FXR1 (FMR1 autosomal homolog 1)	fxr1	animal organ development [GO:0048513]; dentate gyrus development [GO:0021542]; heart development [GO:0007507]; membraneless organelle assembly [GO:0140694]; mRNA destabilization [GO:0061157]; mRNA transport [GO:0051028]; muscle cell development [GO:0055001]; negative regulation of inflammatory response [GO:0050728]; negative regulation of long-term synaptic potentiation [GO:1900272]; negative regulation of translation [GO:0017148]; negative regulation of tumor necrosis factor production [GO:0032720]; positive regulation of long-term neuronal synaptic plasticity [GO:0048170]; positive regulation of translation [GO:0045727]; post-transcriptional regulation of gene expression [GO:0010608]; regulation of mRNA stability [GO:0043488]; regulation of neurogenesis [GO:0050767]; regulation of synaptic transmission, glutamatergic [GO:0051966]; regulation of translation

716.16	631.98	1341.46	512.65	515.08	943.21	3194.24	219.41	1570.29	287.1	299.92	2560.61	1857.37	1517.35	1319.54	3067.13	973.2	1847.25	23373.95	F-box and WD repeat domain-containing 11-B	fbxw11b	protein ubiquitination [GO:0016567]; sprouting angiogenesis [GO:0002040]; Wnt signaling pathway [GO:0016055]
1826.14	1213.23	1500.59	504.87	1034.5	1396.61	3130.81	531.65	943.56	639.75	633.28	935.35	1172.63	1958.57	969.37	2863.09	1443.82	644.43	23342.25	Ubiquitin-like modifier-activating enzyme 1 (EC 6.2.1.45) (Ubiquitin-activating enzyme E1)	UBA1 UBE1	DNA damage response [GO:0006974]; protein ubiquitination [GO:0016567]; ubiquitin-dependent protein catabolic process [GO:0006511]
777.53	1190.11	2742.81	472.1	2163.59	1379.8	3347.73	870.83	481.56	287.96	867.53	771.86	1107.51	2353.4	710.58	2008.71	1219.46	574.02	23327.09	Semaphorin-4G	Sema4g	cell differentiation [GO:0030154]; nervous system development [GO:0007399]; semaphorin-plexin signaling pathway [GO:0071526]
1.08	6234.39	12.24	2.31	5809.41	30.39	0	2346.01	0	0.76	809.21	10.6	2.18	6875.15	0	0.32	1161.86	13.29	23309.2	Choline dehydrogenase, mitochondrial (CDH) (CHD) (EC 1.1.99.1)	Chdh	glycine betaine biosynthetic process from choline [GO:0019285]

2057.56	1156.76	1194.66	602.98	1750.79	948.91	1100.68	387.44	2188.12	552.63	568.13	954.09	2051.61	1425.01	2400.5	848.59	884.28	2227.08	23299.82	Replication factor C subunit 3 (Activator 1 38 kDa subunit) (A1 38 kDa subunit) (Activator 1 subunit 3) (Replication factor C 38 kDa subunit) (RF-C 38 kDa subunit) (RFC38)	RFC3	DNA repair [GO:0006281]; DNA-templated DNA replication [GO:0006261]; positive regulation of DNA-directed DNA polymerase activity [GO:1900264]
1632.64	1016.04	1614.85	475.92	1085.88	1608.67	668.34	475.08	2641.98	497.98	413.19	1773.65	1125.29	1453.91	2234.66	675.94	791.88	3109.84	23295.74	Alpha-mannosidase 2 (EC 3.2.1.114) (Golgi alpha-mannosidase II) (AMan II) (Man II) (Mannosidase alpha class 2A member 1) (Mannosyl-oligosaccharide 1,3-1,6-alpha-mannosidase)	Man2a1 Mana2	in utero embryonic development [GO:0001701]; liver development [GO:0001889]; lung alveolus development [GO:0048286]; mannose metabolic process [GO:0006013]; mitochondrion organization [GO:0007005]; N-glycan processing [GO:0006491]; positive regulation of neurogenesis [GO:0050769]; protein glycosylation [GO:0006486]; respiratory gaseous exchange by respiratory system [GO:0007585]; retina morphogenesis in camera-type eye [GO:0060042]; vacuole organization [GO:0007033]

635.28	1994.95	1355.75	442.73	2628.41	3204.45	8.53	951.91	1560.33	292.94	498.04	1357.84	438.78	3466.16	1633.94	81.6	714.85	2024.29	23290.78	E3 ubiquitin/ISG15 ligase TRIM25 (EC 6.3.2.n3) (Estrogen-responsive finger protein) (RING finger protein 147) (RING-type E3 ubiquitin transferase) (EC 2.3.2.27) (RING-type E3 ubiquitin transferase TRIM25) (Tripartite motif-containing protein 25) (Ubiquitin/ISG15-conjugating enzyme TRIM25) (Zinc finger protein 147)	TRIM25 EFP RNF147 ZNF147	antiviral innate immune response [GO:0140374]; cellular response to leukemia inhibitory factor [GO:1990830]; cytoplasmic pattern recognition receptor signaling pathway [GO:0002753]; ERAD pathway [GO:0036503]; host-mediated suppression of symbiont invasion [GO:0046597]; innate immune response [GO:0045087]; positive regulation of canonical NF-kappaB signal transduction [GO:0043123]; positive regulation of DNA-binding transcription factor activity [GO:0051091]; positive regulation of NF-kappaB transcription factor activity [GO:0051092]; protein K48-linked ubiquitination [GO:0070936]; protein monoubiquitination [GO:0006513]; regulation of protein localization [GO:0032880]; regulation of viral entry into host cell [GO:0046596]; response to estrogen [GO:0043627]; response to oxidative stress [GO:0006979]; response to vitamin D [GO:0033280]; suppression of viral release by host [illegible]
1342.62	1797.27	3111.7	369.85	1490.26	1664.13	208.53	854.62	1463.24	411.98	592.77	1527.06	2109.11	1940.92	1089.1	411.97	1082.7	1813.4	23281.23	Sodium channel protein type 3 subunit alpha (Sodium channel protein brain III subunit alpha) (Sodium channel protein type III subunit alpha) (Voltage-gated sodium channel subtype III) (Voltage-gated sodium channel subunit alpha Nav1.3)	SCN3A KIAA1356 NAC3	behavioral response to pain [GO:0048266]; cardiac muscle cell action potential involved in contraction [GO:0086002]; membrane depolarization during action potential [GO:0086010]; sodium ion transmembrane transport [GO:0035725]; sodium ion transport [GO:0006814]

1440.95		1533.18	1034.89	550.08	1274.97	1679.66	2633.04	667.58	1282.55	480.13	561.57	1142.71	1419.94	1483.91	1211.57	2085.93	1173.63	1601.73	23258.02	Calmodulin-binding transcription activator 1	Camta1 Kiaa0833	neuromuscular process controlling balance [GO:0050885]; positive regulation of calcineurin-NFAT signaling cascade [GO:0070886]; positive regulation of transcription by RNA polymerase II [GO:0045944]
1309.98		2859.83	523.95	433.91	2881.46	1001.14	14.45	1826.42	73.24	328.24	600.66	330.31	2802.05	4693.01	882.47	289.26	1825.67	569.2	23245.25	RelA-associated inhibitor (Inhibitor of ASPP protein) (Protein iASPP) (NFkB-interacting protein 1) (PPP1R13B-like protein)	PPP1R13L IASPP NKIP1 PPP1R13BL RAI	apoptotic process [GO:0006915]; cardiac muscle contraction [GO:0060048]; cardiac right ventricle morphogenesis [GO:0003215]; embryonic camera-type eye development [GO:0031076]; hair cycle [GO:0042633]; multicellular organism growth [GO:0035264]; multicellular organismal-level homeostasis [GO:0048871]; negative regulation of inflammatory response [GO:0050728]; negative regulation of transcription by RNA polymerase II [GO:0000122]; positive regulation of cell differentiation [GO:0045597]; post-embryonic development [GO:0009791]; regulation of transcription by RNA polymerase II [GO:0006357]; ventricular cardiac muscle tissue development [GO:0003229]

676.5	3225.23	1355.6	732.09	2351.09	1467.51	487.91	1572.88	451.8	400.96	1604.91	2878.85	1260.42	1115.65	504.68	355.47	2229.47	569.2	23240.22	Inward rectifier potassium channel 2 (Inward rectifier K(+) channel Kir2.1) (IRK-1) (RBL-IRK1) (Potassium channel, inwardly rectifying subfamily J member 2)	Kcnj2 Irk1	cardiac muscle cell action potential [GO:0086001]; cardiac muscle cell action potential involved in contraction [GO:0086002]; cellular response to mechanical stimulus [GO:0071260]; magnesium ion transport [GO:0015693]; membrane repolarization during action potential [GO:0086011]; membrane repolarization during cardiac muscle cell action potential [GO:0086013]; positive regulation of potassium ion transmembrane transport [GO:1901381]; potassium ion import across plasma membrane [GO:1990573]; potassium ion transmembrane transport [GO:0071805]; potassium ion transport [GO:0006813]; protein homotetramerization [GO:0051289]; regulation of cardiac muscle cell contraction [GO:0086004]; regulation of heart rate by cardiac conduction [GO:0086091]; regulation of membrane repolarization [GO:0060306]; regulation of monoatomic ion transmembrane transport [GO:0034765]; regulation of
104.36	4234.91	307.69	281.03	3633.68	265.84	6.62	3074.88	628.08	174.45	1203.72	624.87	92.25	3756.68	631.96	76.28	2970.99	996.31	23064.6	TBC1 domain family member 25	Tbc1d25	autophagy [GO:0006914]; regulation of autophagosome maturation [GO:1901096]
245.01	397.14	1673.25	563.73	540.34	123.22	3.82	375.44	5453.35	391.57	529.98	2401.37	247.12	377.96	3820.67	160.53	543.04	5195.38	23042.92	MICAL-like protein 2 (Junctional Rab13-binding protein) (Molecule interacting with CasL-like 2) (MICAL-L2)	MICALL2 JRAB	actin cytoskeleton organization [GO:0030036]; actin filament polymerization [GO:0030041]; bicellular tight junction assembly [GO:0070830]; endocytic recycling [GO:0032456]; endosome membrane tubulation [GO:0097750]; neuron projection development [GO:0031175]; positive regulation of protein targeting to mitochondrion [GO:1903955]; Rab protein signal transduction [GO:0032482]; substrate adhesion-dependent cell spreading [GO:0034446]

1082.16	1959.09	39.27	1677.95	981.2	13.54	127.45	913.07	3.87	2412.01	3360.78	1.8	1538.08	1919.6	8.84	2041.16	4866.78	96.24	23042.89	Tumor protein p63-regulated gene 1-like protein (Mossy fiber terminal-associated vertebrate-specific presynaptic protein) (Protein FAM79A)	TPRG1L FAM79A MOVER	calcineurin-NFAT signaling cascade [GO:0033173]; negative regulation of synaptic transmission [GO:0050805]; regulation of synaptic transmission, glutamatergic [GO:0051966]; synaptic vesicle docking [GO:0016081]
1165.41	1647.55	1282.92	921.55	2538.87	1446.15	1790.55	920.42	674.87	537.51	1214.79	1028.81	1153.48	2486.17	650.37	1225.04	1632.19	706.02	23022.67	Unconventional myosin-If (Myosin-Ie)	MYO1F	actin filament organization [GO:0007015]; endocytosis [GO:0006897]
1093.83	1000.67	1785.8	329.55	1673.61	2380.22	19.86	830.85	1647.82	445.51	557.13	1364.76	1552.17	2473.53	2493.41	150.48	566.7	2434.52	22800.42	Intersectin-1	itsn1	endocytosis [GO:0006897]; exocytosis [GO:0006887]; intracellular signal transduction [GO:0035556]; positive regulation of Rho protein signal transduction [GO:0035025]; protein transport [GO:0015031]

1238.09	1551.78	1413.12	667.65	1664.41	1926.53	1919.89	525.19	1038.17	356.64	508.71	1586.01	1673.24	2138.28	963.52	1401.39	1135.64	1089.53	22797.79	Rap1 GTPase-GDP dissociation stimulator 1-A (Rap1gds1-A protein) (RalB-binding protein A) (XsmgGDS-A) (smgGDS-A)	rap1gds1-a	positive regulation of GTPase activity [GO:0043547]; small GTPase-mediated signal transduction [GO:0007264]
573.73	109.66	1289.02	174.42	168.41	1171.32	7171.7	105.82	602.92	254.01	300.19	684.11	735.6	1268.97	760.22	6252.63	561.57	542.16	22726.46	Inner nuclear membrane protein Man1 (LEM domain-containing protein 3)	Lemd3 Man1	angiogenesis [GO:0001525]; blood vessel endothelial cell migration involved in intussusceptive angiogenesis [GO:0002044]; nucleus organization [GO:0006997]; regulation of cell cycle [GO:0051726]; regulation of extracellular matrix organization [GO:1903053]; skeletal muscle cell differentiation [GO:0035914]
1221.56	1657.89	1003.15	574.76	2055.25	2099.66	39.8	1426.41	123.45	635.54	981.51	898.89	3546.76	2654.69	1231.83	303.75	1517.39	638.04	22610.33	Protein phosphatase 1A (EC 3.1.3.16) (Protein phosphatase 2C isoform alpha) (PP2C-alpha) (Protein phosphatase IA)	PPM1A PPPM1A	cellular response to transforming growth factor beta stimulus [GO:0071560]; dephosphorylation [GO:0016311]; N-terminal protein myristoylation [GO:0006499]; negative regulation of BMP signaling pathway [GO:0030514]; negative regulation of canonical NF-kappaB signal transduction [GO:0043124]; negative regulation of non-canonical NF-kappaB signal transduction [GO:1901223]; negative regulation of transcription by RNA polymerase II [GO:0000122]; negative regulation of transforming growth factor beta receptor signaling pathway [GO:0030512]; positive regulation of canonical NF-kappaB signal transduction [GO:0043123]; positive regulation of canonical Wnt signaling pathway [GO:0090263]; positive regulation of DNA-templated transcription [GO:0045893]; positive regulation of protein export from nucleus [GO:0046827]; protein dephosphorylation [GO:0006470]; protein export from nucleus [illegible]

215.54	165.17	1755.94	65.75	114.78	768	258.93	19.48	5729.87	68.71	57.46	1734.46	491.57	459.35	5386.94	287.37	419.57	4475.94	22474.83	tRNA (34-2'-O)-methyltransferase regulator WDR6 (WD repeat-containing protein 6)	WDR6	G1 to G0 transition [GO:0070314]; negative regulation of autophagy [GO:0010507]; negative regulation of cell population proliferation [GO:0008285]; tRNA methylation [GO:0030488]; wobble position ribose methylation [GO:0002130]
1257.64	1801.07	1737.28	300.65	1378.33	2721.52	446.92	679.09	1150	923.37	606.71	1661.05	1192.79	1804.94	1805.84	401.49	988.36	1606.19	22463.24	Ankyrin-2 (ANK-2) (Ankyrin-B) (Brain ankyrin)	Ank2 AnkB	atrial cardiac muscle cell action potential [GO:0086014]; atrial cardiac muscle cell to AV node cell communication [GO:0086066]; cardiac muscle contraction [GO:0060048]; endocytosis [GO:0006897]; intracellular calcium ion homeostasis [GO:0006874]; positive regulation of calcium ion transport [GO:0051928]; positive regulation of gene expression [GO:0010628]; positive regulation of potassium ion import across plasma membrane [GO:1903288]; protein localization [GO:0008104]; protein localization to cell surface [GO:0034394]; protein localization to endoplasmic reticulum [GO:0070972]; protein localization to M-band [GO:0036309]; protein localization to plasma membrane [GO:0072659]; protein localization to T-tubule [GO:0036371]; protein stabilization [GO:0050821]; protein transport [GO:0015031]; regulation of atrial cardiac muscle cell action potential [GO:0098910]; regulation of AV node cell
1981.57	1883.35	1363.31	308.97	1293.14	1526.78	428.71	838.06	104.83	566.34	928.53	787.23	2299.65	3305.47	1117.6	1261.99	2071.57	375.88	22442.98	Palmitoyltransferase ZDHHC5-A (EC 2.3.1.225) (Zinc finger DHHC domain-containing protein 5-A) (DHHC-5A)	zdhhc5a	positive regulation of pattern recognition receptor signaling pathway [GO:0062208]

2395.24	1547.28	1892.76	553.62	1699.68	2730.31	179.49	802.08	456.38	595.79	665.25	1013.36	2332.12	1965.03	1021.67	374.06	1318.65	850.98	22393.75	Alpha-2-macroglobulin-like protein 1 (C3 and PZP-like alpha-2-macroglobulin domain-containing protein 9)	A2ML1 CPAMD9	regulation of endopeptidase activity [GO:0052548]
0	0	25.41	0	0	0	0	1.02	6825.49	0	0	1854.17	0	0	7372.85	0	2.43	6281.48	22362.85	Growth hormone-regulated TBC protein 1-A	grtp1a grtp1	
4440.43	55.2	39.11	723.49	261.41	355.9	9.8	24.37	0	2774.14	159.35	137.77	12904.27	39.18	35.67	299.41	47.68	3.5	22310.68	Oxysterol-binding protein-related protein 10 (ORP-10) (OSBP-related protein 10)	Osbpl10 Orp10	lipid metabolic process [GO:0006629]; lipid transport [GO:0006869]
1217.59	2080.5	1403.08	532.68	2038.35	2095.55	28.79	1051.66	283.55	924.67	1057.93	1100.86	2450.3	2753.92	1045.93	185.61	1440.5	583.5	22274.97	Opioid growth factor receptor (OGFr) (Zeta-type opioid receptor)	Ogfr	
3541.04	857.19	1184.37	897.42	1440.17	2763.67	223.93	999.63	1625.44	1293.4	694.35	1258.88	1059.8	1256.19	542.11	221.44	1410.58	1003.3	22272.91	A disintegrin and metalloproteinase with thrombospondin motifs 6 (ADAM-TS 6) (ADAM-TS6) (ADAMTS-6) (EC 3.4.24.-)	ADAMTS6	aorta development [GO:0035904]; cardiac septum development [GO:0003279]; coronary vasculature development [GO:0060976]; extracellular matrix organization [GO:0030198]; kidney development [GO:0001822]; proteolysis [GO:0006508]

313.72	1439.13	2105.24	222.46	1155.4	1292.95	115.22	807.72	2553.62	172.07	373.69	1695.64	427.04	2642.97	2622.87	321.53	727.48	3056.96	22045.71	Transposon Tf2-9 polyprotein (Retrotransposable element Tf2 155 kDa protein)	Tf2-9 SPBC9B6.02c	DNA integration [GO:0015074]; DNA recombination [GO:0006310]; proteolysis [GO:0006508]
629.12	852.42	1945.81	239.41	945.39	1474.65	32.2	531.37	2671.43	310.07	333.06	1955	677.02	1348.21	3406.1	212.89	967.95	3494.1	22026.2	Son of sevenless homolog 1 (SOS-1)	SOS1	axon guidance [GO:0007411]; B cell homeostasis [GO:0001782]; B cell receptor signaling pathway [GO:0050853]; blood vessel morphogenesis [GO:0048514]; cardiac atrium morphogenesis [GO:0003209]; cytokine-mediated signaling pathway [GO:0019221]; epidermal growth factor receptor signaling pathway [GO:0007173]; eyelid development in camera-type eye [GO:0061029]; Fc-epsilon receptor signaling pathway [GO:0038095]; fibroblast growth factor receptor signaling pathway [GO:0008543]; hair follicle development [GO:0001942]; heart trabecula morphogenesis [GO:0061384]; insulin receptor signaling pathway [GO:0008286]; insulin-like growth factor receptor signaling pathway [GO:0048009]; leukocyte migration [GO:0050900]; midbrain morphogenesis [GO:1904693]; multicellular organism growth [GO:0035264]; myelination [GO:0042552]; neurotrophin TRK receptor signaling pathway
643.65	882.82	531	542.9	1404.56	1415.98	4857.54	504.09	991.21	647.55	627.94	1363.42	1076.96	1639.6	662.76	1462.07	1454.45	1314.66	22023.16	MAP kinase-activated protein kinase 5 (MAPK-activated protein kinase 5) (MAPKAP kinase 5) (MAPKAPK-5) (EC 2.7.11.1)	Mapkapk5	negative regulation of TOR signaling [GO:0032007]; positive regulation of dendritic spine development [GO:0060999]; positive regulation of telomere maintenance [GO:0032206]; positive regulation of transcription by RNA polymerase II [GO:0045944]; protein autophosphorylation [GO:0046777]; Ras protein signal transduction [GO:0007265]; regulation of translation [GO:0006417]; stress-induced premature senescence [GO:0090400]

68.08	0	2.09	304.64	0	10.15	14395.45	0	0	156.3	420.96	0	0	0	0	6469.82	131.88	0	21959.37	Trimeric intracellular cation channel type A (TRIC-A) (TRICA) (Transmembrane protein 38A)	tmem38a zgc:77831	regulation of release of sequestered calcium ion into cytosol [GO:0051279]
3208.36	330.41	1141.02	953.32	239.78	1866.54	2901.42	165.58	66.61	1046.72	466.45	445.23	2371.62	411.01	1730.06	3858.23	675.89	58.14	21936.39	Transposable element Tc1 transposase	tc1a tca T07D3.8	DNA integration [GO:0015074]; DNA transposition [GO:0006313]
1050.73	709.72	1497.08	271.19	651.89	580.42	1108.07	294.76	4114.53	276.05	187.93	1363.32	1010.79	1257.24	2691.36	724.12	542.41	3587.92	21919.53	Apolipoprotein F (Apo-F) (Liver regeneration-related protein LRRG151)	Apof Ba1-666	cholesterol efflux [GO:0033344]; cholesterol metabolic process [GO:0008203]; triglyceride metabolic process [GO:0006641]
424.94	225.96	3626	216.54	160.08	2674.71	3.63	111.14	2927.77	253.66	125.67	1323.4	1360.66	560.18	2910.03	238.79	425.1	4265.9	21834.16	E3 ubiquitin-protein ligase RING2 (EC 2.3.2.27) (RING finger protein 1B) (RING1b) (RING finger protein 2) (RING-type E3 ubiquitin transferase RING2)	rnf2 ring1b	cardiac conduction system development [GO:0003161]; central nervous system development [GO:0007417]; embryonic cranial skeleton morphogenesis [GO:0048701]; enteric nervous system development [GO:0048484]; epigenetic regulation of gene expression [GO:0040029]; negative regulation of intrinsic apoptotic signaling pathway by p53 class mediator [GO:1902254]; negative regulation of transcription by RNA polymerase II [GO:0000122]; pectoral fin development [GO:0033339]; protein ubiquitination [GO:0016567]; regulation of cardiac myofibril assembly [GO:1905304]; regulation of chondrocyte differentiation [GO:0032330]; regulation of neural crest cell differentiation [GO:1905292]; regulation of neural crest cell fate specification [GO:1905295]; retina morphogenesis in camera-type eye [GO:0060042]

8145.76	522.97	273.52	1658.55	234.78	384.69	14.23	215.88	103.17	3070.7	610.94	318.89	3778.48	505.67	183.83	331.05	1120.33	249.47	21722.91	Spindlin-1 (Spindlin1)	SPIN1 SPIN	gamete generation [GO:0007276]; meiotic cell cycle [GO:0051321]; positive regulation of DNA-templated transcription [GO:0045893]; positive regulation of Wnt signaling pathway [GO:0030177]; rRNA transcription [GO:0009303]; Wnt signaling pathway [GO:0016055]
795.52	3154.72	623.02	476.01	1760.94	856.31	1951.02	1088.04	779.54	293.99	608.89	709.89	1578.76	1493.41	961.08	1315.36	2419.96	840.73	21707.19	TBC1 domain family member 4 (Akt substrate of 160 kDa) (AS160)	TBC1D4 AS160 KIAA0603	cellular response to insulin stimulus [GO:0032869]; negative regulation of vesicle fusion [GO:0031339]; vesicle-mediated transport [GO:0016192]
547.32	504.13	1393.26	153.71	376.27	533.45	291.98	174.06	4022.63	137.89	219.04	2085	506.34	518.36	3721.64	449.06	312.1	5700.32	21646.56	Carboxypeptidase O (EC 3.4.17.-)	cpo	proteolysis [GO:0006508]
1346.58	864.91	1731.73	527.37	625.38	1650.52	2726.01	543.83	1201.34	437.65	384.61	1883.44	749.62	801.28	903.21	2276.23	923.3	2035.11	21612.12	Epoxide hydrolase 4 (EC 3.3.-.-) (Abhydrolase domain-containing protein 7) (Epoxide hydrolase-related protein)	Ephx4 Abhd7 Ephxrp	

873.11	841.61	1347.45	235.94	404.93	851.94	5348.72	485.93	803.84	82.13	139.02	446.19	1361.59	700.2	938.79	4799.93	760.75	1183.78	21605.85	Storkhead-box protein 2	STOX2 QmoA-11510	embryo development ending in birth or egg hatching [GO:0009792]; maternal placenta development [GO:0001893]; regulation of transcription by RNA polymerase II [GO:0006357]
894.46	515.93	1393.17	468.95	460.85	783.37	3596.3	212.33	1453.88	415.72	425.44	1936.18	1218.15	1625.42	1437.97	2935.25	746.87	1007.79	21528.03	Glutaredoxin 1	grxC1 grx RP204	cell redox homeostasis [GO:0045454]
887.38	938.2	1158.24	961.47	1188.53	2152.38	0	5.65	127.4	1247.84	1117.36	2990.97	1543.91	1866.76	883.27	1792.88	1544.75	1084.07	21491.06	Spliceosome RNA helicase DDX39B (EC 3.6.4.13) (56 kDa U2AF65-associated protein) (ATP-dependent RNA helicase p47) (DEAD box protein UAP56) (HLA-B-associated transcript 1 protein)	DDX39B BAT1 UAP56	mRNA export from nucleus [GO:0006406]; mRNA splicing, via spliceosome [GO:0000398]; RNA export from nucleus [GO:0006405]; RNA splicing [GO:0008380]; spliceosomal complex assembly [GO:0000245]

2037.13	1116.22	2548.76	612.69	898.79	1476.29	321.62	287.88	824.07	509.65	326.72	2738.57	2836.08	1413.88	1508.42	508.9	635.48	812.86	21414.01	DNA-directed RNA polymerase I subunit RPA34 (A34.5) (Antisense to ERCC-1 protein) (ASE-1) (CD3-epsilon-associated protein) (CD3E-associated protein) (DNA-directed RNA polymerase I subunit G) (RNA polymerase I-associated factor PAF49)	POLR1G ASE1 CAST CD3EAP PAF49	cell surface receptor protein tyrosine kinase signaling pathway [GO:0007169]; rRNA transcription [GO:0009303]; transcription initiation at RNA polymerase I promoter [GO:0006361]
145.56	11.84	3122.18	27.59	8.68	44.63	0.96	2.99	5976.62	19.53	12.87	1669.35	223.66	47.06	4067.56	12.56	36.85	5971.14	21401.63	Putative E3 ubiquitin-protein ligase UBR7 (EC 2.3.2.27) (N-recognin-7) (RING-type E3 ubiquitin transferase UBR7)	Ubr7	protein ubiquitination [GO:0016567]
1132.27	847.75	1621.95	294.08	566.23	1033.88	2570.31	284.86	1680.76	369.68	245	1043.87	1542.92	1337.07	2093.62	2100.86	667.08	1954.24	21386.43	Polymerase delta-interacting protein 2 (38 kDa DNA polymerase delta interaction protein) (p38)	POLDIP2 PDIP38 POLD4 HSPC017	error-free translesion synthesis [GO:0070987]; mitochondrion organization [GO:0007005]; mitotic spindle assembly [GO:0090307]; negative regulation of macroautophagy [GO:0016242]; positive regulation of focal adhesion assembly [GO:0051894]; positive regulation of mitotic cell cycle [GO:0045931]; positive regulation of mitotic cytokinesis [GO:1903490]; vascular associated smooth muscle cell proliferation [GO:1990874]

701.6	2628.43	1804.18	311.72	1941.73	1326.06	21.37	1566.11	837.76	355.46	994.14	1024.84	834.61	2944.71	752.77	148.95	2003.85	1128.98	21327.27	Zyxin	ZYX	cell adhesion [GO:0007155]; integrin-mediated signaling pathway [GO:0007229]; transforming growth factor beta receptor signaling pathway [GO:0007179]
1464.53	1643.82	3143.06	173.57	701.81	522.06	15.38	598.77	1576.68	531.37	467.84	2120.7	1095.49	1514.25	1198.71	426.24	1508.96	2601.13	21304.37	Tissue factor (TF) (Coagulation factor III) (CD antigen CD142)	F3 Cf-3 Cf3	blood coagulation [GO:0007596]
656.23	834.73	695.39	139.87	1178.81	1314.01	4509.63	567.67	1031.44	247.44	391.7	1001.79	1164.94	1252.29	1816.54	2277.44	555.57	1643.46	21278.95	DNA (cytosine-5)-methyltransferase 3C (Dnmt3c) (EC 2.1.1.37)	Dnmt3c Gm14490	cell differentiation [GO:0030154]; gene silencing by piRNA-directed DNA methylation [GO:0141176]; homologous chromosome pairing at meiosis [GO:0007129]; male meiosis I [GO:0007141]; methylation [GO:0032259]; spermatogenesis [GO:0007283]; transposable element silencing by heterochromatin formation [GO:0141005]; transposable element silencing by piRNA-mediated DNA methylation [GO:0141196]; transposable element silencing by piRNA-mediated heterochromatin formation [GO:0141006]

783	1717.77	1347.34	1286.27	1559.1	2443.75	26.73	943.49	692.49	1317.68	1413.46	715.37	1109.61	2380.36	907.83	341.86	1265.4	1007.29	21258.8	Cadherin EGF LAG seven-pass G-type receptor 3 (Cadherin family member 11) (Epidermal growth factor-like protein 1) (EGF-like protein 1) (Flamingo homolog 1) (hFmi1) (Multiple epidermal growth factor-like domains protein 2) (Multiple EGF-like domains protein 2)	CELSR3 CDHF11 EGFL1 FMI1 KIAA0812 MEGF2	cell-cell adhesion [GO:0098609]; dopaminergic neuron axon guidance [GO:0036514]; G protein-coupled receptor signaling pathway [GO:0007186]; homophilic cell adhesion via plasma membrane adhesion molecules [GO:0007156]; serotonergic neuron axon guidance [GO:0036515]; Wnt signaling pathway, planar cell polarity pathway [GO:0060071]
2089.55	1042.35	1176.7	883.28	1051.84	1715.71	2840.76	384.31	466.32	558.03	460.87	1018.26	1599.93	1683.56	548.66	2098.47	1043.12	567.4	21229.12	T-box transcription factor TBX1 (T-box protein 1)	tbx1 zgc:136724	cardiac muscle cell differentiation [GO:0055007]; cartilage morphogenesis [GO:0060536]; cell fate specification [GO:0001708]; chordate pharyngeal muscle development [GO:0043282]; ear development [GO:0043583]; ear morphogenesis [GO:0042471]; embryonic heart tube morphogenesis [GO:0003143]; embryonic viscerocranium morphogenesis [GO:0048703]; heart development [GO:0007507]; heart looping [GO:0001947]; neural crest cell development [GO:0014032]; neural crest cell migration [GO:0001755]; parathyroid gland development [GO:0060017]; pharyngeal system development [GO:0060037]; positive regulation of DNA-templated transcription [GO:0045893]; positive regulation of secondary heart field cardioblast proliferation [GO:0072513]; regulation of transcription by RNA polymerase II [GO:0006357]; semicircular canal morphogenesis [GO:0048752]; soft palate development [illegible]

56.55	2607.81	164.82	32.19	2708.3	458.22	1.39	2541.74	1.73	35.52	1039.22	82.1	63.29	5988.07	102.57	141.39	5070.66	62.09	21157.66	5'-3' exonuclease PLD3 (EC 3.1.16.1) (Choline phosphatase 3) (Phosphatidylcholine-hydrolyzing phospholipase D3) (Phospholipase D3) (PLD 3)	pld3	immune system process [GO:0002376]; inflammatory response [GO:0006954]; myotube differentiation [GO:0014902]; regulation of cytokine production involved in inflammatory response [GO:1900015]
1360.68	1271.11	1286.44	309.63	1694.03	2053.03	1200.96	808.11	1013.01	512.24	600.55	1134.2	1539.21	1812.99	1568.18	753.3	935.58	1290.76	21144.01	Probable E3 ubiquitin-protein ligase HECTD4 (EC 2.3.2.26) (HECT domain-containing protein 4) (HECT-type E3 ubiquitin transferase HECTD4)	HECTD4 C12orf51 KIAA0614	glucose homeostasis [GO:0042593]; glucose metabolic process [GO:0006006]; protein ubiquitination [GO:0016567]
38.37	1662.84	73.92	80.6	5430.48	433.19	0	2016.12	0	48.99	5181.19	465.97	0	4030.65	148.79	196.11	1286.35	0	21093.57	MAP7 domain-containing protein 1	Map7d1 Mtap7d1	microtubule cytoskeleton organization [GO:0000226]
4171.69	52.44	35.02	913.85	386.32	1152.34	0	62.94	1.31	2306.32	215.07	144.57	11222.37	46.73	70.63	145.16	78	3.43	21008.19	WD repeat-containing protein 13	WDR13	

790.69	1513.66	3346.76	420.81	869.9	5907.76	153.33	441.65	303.11	389.17	594.51	2579.03	596.54	753.33	936.99	349.37	699.97	349.57	20996.15	Netrin-G2 (Laminet-2)	Ntng2 Lmnt2	axonogenesis [GO:0007409]; modulation of chemical synaptic transmission [GO:0050804]; postsynaptic specialization assembly [GO:0098698]; regulation of neuron migration [GO:2001222]; regulation of neuron projection arborization [GO:0150011]; regulation of neuron projection development [GO:0010975]; regulation of presynapse assembly [GO:1905606]; synaptic membrane adhesion [GO:0099560]
483.9	1650.93	297	257	1608.28	423.61	1335.14	344.6	403.46	135.44	431.86	269.65	644.13	7720.62	307.19	2269.36	2097.85	293.29	20973.31	Protein FAM110B	fam110b zgc:114139	
278.07	1559.14	239.98	763.12	2034.63	1375.04	19.55	1249.83	427.61	110.55	286.17	326.26	480.12	6459.5	778.86	294.61	3396.31	877.82	20957.17	Sorting nexin-2	SNX2	early endosome to Golgi transport [GO:0034498]; intracellular protein transport [GO:0006886]; lamellipodium morphogenesis [GO:0072673]; retrograde transport, endosome to Golgi [GO:0042147]

234.85	3677.8	2517.78	147.3	1570.95	823.5	5.06	1427.86	1068.79	88.23	626.54	670.12	221.1	4785.34	393.3	44.86	1721.27	926.5	20951.15	Mitochondrial tRNA methylthiotransferase CDK5RAP1 (EC 2.8.4.3) (CDK5 activator-binding protein C42) (CDK5 regulatory subunit-associated protein 1) (mt-tRNA-2-methylthio-N6-dimethylallyladenosine synthase) (mt-tRNA-N6-(dimethylallyl)adenosine(37) methylthiotransferase)	Cdk5rap1	mitochondrial tRNA modification [GO:0070900]; negative regulation of cyclin-dependent protein serine/threonine kinase activity [GO:0045736]; positive regulation of mitochondrial translation [GO:0070131]; positive regulation of translational fidelity [GO:0045903]; regulation of cyclin-dependent protein serine/threonine kinase activity [GO:0000079]; RNA modification [GO:0009451]
1144.8	1273.93	1570.37	478.27	1562.3	3680.46	687.99	307.97	130.58	616.14	666.09	1346.44	1635.53	1788.26	1507.18	622.21	878	1025.59	20922.11	Paired amphipathic helix protein Sin3b (Histone deacetylase complex subunit Sin3b) (Transcriptional corepressor Sin3b)	Sin3b Kiaa0700	cardiac muscle tissue development [GO:0048738]; negative regulation of cell cycle [GO:0045786]; negative regulation of cell migration [GO:0030336]; negative regulation of DNA-templated transcription [GO:0045892]; negative regulation of transcription by RNA polymerase II [GO:0000122]; skeletal muscle tissue development [GO:0007519]

133.41	3314.19	73.9	166.67	3636.77	32.73	34.24	2162.37	13.46	53.6	1274.99	78.41	45.26	7861.29	7.88	188.61	1703.51	28.04	20809.33	LON peptidase N-terminal domain and RING finger protein 2 (Neuroblastoma apoptosis-related protease) (RING finger protein 192)	LONRF2 RNF192	motor behavior [GO:0061744]; negative regulation of neuron apoptotic process [GO:0043524]; neuromuscular process [GO:0050905]; neuron apoptotic process [GO:0051402]; neuron projection development [GO:0031175]; protein quality control for misfolded or incompletely synthesized proteins [GO:0006515]; spinal cord motor neuron differentiation [GO:0021522]
123.74	136.81	186.1	158.25	122.46	122.83	12023.56	56.53	210.35	139.12	398.34	377.9	317.63	195.52	306.27	5176.45	265.15	439.79	20756.8	F-box/LRR-repeat protein 19 (F-box and leucine-rich repeat protein 19)	Fbxl19	proteasome-mediated ubiquitin-dependent protein catabolic process [GO:0043161]
1105.55	1582.33	789.55	468.47	1714.56	2784.28	1203.14	979.03	774.51	397.74	602.04	1448.86	1633.34	1570.93	1367.24	667.8	734.86	818.89	20643.12	Caspase-8 (CASP-8) (EC 3.4.22.61) [Cleaved into: Caspase-8 subunit p18;Caspase-8 subunit p10]	Casp8	angiogenesis [GO:0001525]; apoptotic process [GO:0006915]; apoptotic signaling pathway [GO:0097190]; cardiac muscle tissue development [GO:0048738]; execution phase of apoptosis [GO:0097194]; extrinsic apoptotic signaling pathway [GO:0097191]; extrinsic apoptotic signaling pathway via death domain receptors [GO:0008625]; heart development [GO:0007507]; hepatocyte apoptotic process [GO:0097284]; macrophage differentiation [GO:0030225]; necroptotic process [GO:0070266]; negative regulation of necroptotic process [GO:0060546]; neural tube formation [GO:0001841]; positive regulation of apoptotic process [GO:0043065]; positive regulation of execution phase of apoptosis [GO:1900119]; positive regulation of extrinsic apoptotic signaling pathway [GO:2001238]; protein processing [GO:0016485]; proteolysis involved in protein catabolic process [illegible]

501.89	388.8	3770.06	134.64	486.81	1396.82	327.14	187.73	3055.41	259.66	228.43	1940.47	600.64	1020.98	2859.2	295.39	326.88	2859.76	20640.71	Serine palmitoyltransferase 2 (EC 2.3.1.50) (Long chain base biosynthesis protein 2) (LCB 2) (Long chain base biosynthesis protein 2a) (LCB2a) (Serine-palmitoyl-CoA transferase 2) (SPT 2)	Sptlc2 Lcb2	adipose tissue development [GO:0060612]; ceramide biosynthetic process [GO:0046513]; lipophagy [GO:0061724]; positive regulation of lipophagy [GO:1904504]; sphinganine biosynthetic process [GO:0046511]; sphingolipid biosynthetic process [GO:0030148]; sphingomyelin biosynthetic process [GO:0006686]; sphingosine biosynthetic process [GO:0046512]
1161.58	973.86	4345.19	229.41	740.31	610.56	45.43	607.59	2194.65	441.19	521.52	986.35	352.02	2687.12	1562.06	121.76	638.92	2417.58	20637.1	Cadherin-2 (Neural cadherin) (N-cadherin) (Parachute) (ZNCAD)	cdh2 pac	adherens junction organization [GO:0034332]; auditory receptor cell stereocilium organization [GO:0060088]; axial mesoderm structural organization [GO:0048331]; axon guidance [GO:0007411]; axonal fasciculation [GO:0007413]; brain development [GO:0007420]; calcium-dependent cell-cell adhesion via plasma membrane cell adhesion molecules [GO:0016339]; cardioblast differentiation [GO:0010002]; cartilage condensation [GO:0001502]; cell migration [GO:0016477]; cell migration in hindbrain [GO:0021535]; cell migration involved in somitogenic axis elongation [GO:0090248]; cell morphogenesis [GO:0000902]; cell-cell adhesion [GO:0098609]; cell-cell adhesion mediated by cadherin [GO:0044331]; cell-cell adhesion via plasma-membrane adhesion molecules [GO:0098742]; cell-cell junction assembly [GO:0007043]; cilium assembly [GO:0060271]; commissural neuron axon guidance [GO:0071679];

1034.86	604.32	7773.22	328.95	168.5	1856.67	5.54	73.77	75.49	364.3	198.01	1923.98	2605.66	659.34	698.18	1135.63	883.05	238.88	20628.35	Protein kinase C-binding protein NELL1 (NEL-like protein 1)	Nell1	cell differentiation [GO:0030154]; negative regulation of osteoblast proliferation [GO:0033689]; negative regulation of protein catabolic process [GO:0042177]; positive regulation of bone mineralization [GO:0030501]; positive regulation of osteoblast differentiation [GO:0045669]; regulation of gene expression [GO:0010468]	
78.2	482.02	300.03	280.99	187.05	949.85	9746.59	70.88	56.58	149.56	284.11	205.34	18.43	160.89	837.21	6354.93	395.02	15.56	20573.24	Uridine-cytidine kinase 2-B (UCK 2-B) (EC 2.7.1.48) (Cytidine monophosphokinase 2-B) (Uridine monophosphokinase 2-B)	uck2b si:ch211-284b7.4 zgc:56174	CTP salvage [GO:0044211]; UMP salvage [GO:0044206]	
0.76	1.72	14207.02	0.96	0	313.91	0.23	0.21	83.71	1.27	0.51	5879.5	3.27	1.05	36.13	2.1	0.49	2.44	20535.28	Protein piccolo (Aczonin)	PCLO ACZ	cytoskeleton organization [GO:0007010]; presynaptic active zone assembly [GO:1904071]; protein localization to synapse [GO:0035418]	

5103.26	71.12	953.6	2155.88	114.3	4077.33	7.07	40.06	264.66	2142.35	58.97	1157.44	2502.06	67.74	373.67	122.51	206.84	1097.14	20516	Ubiquitin-like protein 4A	ubl4a	post-translational protein targeting to endoplasmic reticulum membrane [GO:0006620]; tail-anchored membrane protein insertion into ER membrane [GO:0071816]
2688.7	828.13	1424.66	527.18	1692.24	2084.4	75.2	847.73	717.34	597.74	733.22	1017.73	2216.58	1335.26	1223.4	179.3	1256.61	1064.98	20510.4	Plexin-C1 (Virus-encoded semaphorin protein receptor) (CD antigen CD232)	PLXNC1 VESPR	cell adhesion [GO:0007155]; negative regulation of cell adhesion [GO:0007162]; positive regulation of axonogenesis [GO:0050772]; regulation of cell migration [GO:0030334]; regulation of cell shape [GO:0008360]; regulation of synapse pruning [GO:1905806]; semaphorin-plexin signaling pathway [GO:0071526]; synapse assembly [GO:0007416]
3330.86	578.11	199.76	713.44	763.58	268.67	10.2	235.54	6.39	620.61	248.39	176.44	10032.85	1336.33	960.87	414.54	379.46	162.31	20438.35	Protein phosphatase 1 regulatory subunit 16A (Myosin phosphatase-targeting subunit 3)	PPP1R16A MYPT3	
349.68	910.09	483.21	32.27	902.26	1590.2	0	926.32	3896.89	259.2	395.39	1011.41	242.99	1207.87	2618.51	255.3	1239.87	4110.03	20431.49	Signal peptidase complex subunit 3 (Microsomal signal peptidase 22/23 kDa subunit) (SPC22/23) (SPase 22/23 kDa subunit)	SPCS3 SPC22	signal peptide processing [GO:0006465]; viral protein processing [GO:0019082]

1200.72	1285.73	1505.36	418.42	1640.93	1901.3	16.14	1330.42	303.7	454.28	518.44	1189.56	2013.69	1918.24	1690.04	203.39	1099.21	1571.53	20261.1	Guanine nucleotide exchange factor VAV3 (VAV-3)	Vav3	angiogenesis [GO:0001525]; B cell receptor signaling pathway [GO:0050853]; cell migration [GO:0016477]; cell projection assembly [GO:0030031]; DNA damage response [GO:0006974]; integrin-mediated signaling pathway [GO:0007229]; lamellipodium assembly [GO:0030032]; neutrophil chemotaxis [GO:0030593]; positive regulation of B cell proliferation [GO:0030890]; positive regulation of cell adhesion [GO:0045785]; positive regulation of phosphatidylinositol 3-kinase/protein kinase B signal transduction [GO:0051897]; regulation of cell size [GO:0008361]; response to xenobiotic stimulus [GO:0009410]; small GTPase-mediated signal transduction [GO:0007264]; vesicle fusion [GO:0006906]
492.72	397.21	657.74	148.39	332.03	458.85	449.21	191.18	4871.76	194.35	145.97	1481.67	513.18	413.96	3622.35	533.05	242.7	4927.19	20073.51	Pleckstrin homology domain-containing family A member 5 (PH domain-containing family A member 5) (Phosphoinositol 3-phosphate-binding protein 2) (PEPP-2)	PLEKHA5 KIAA1686 PEPP2	reproductive system development [GO:0061458]
1030.17	1095.57	2102.02	329.72	1096.87	1607.52	17.78	543.49	1470.58	393.11	491.27	1714.53	1588.05	2034.67	1504.38	264.59	1014.53	1754.08	20052.93	Neuropilin-1a (znrp1)	nrp1a np-1 nrp1	angiogenesis [GO:0001525]; angiogenesis involved in wound healing [GO:0060055]; axon extension [GO:0048675]; axon guidance [GO:0007411]; axonal fasciculation [GO:0007413]; cartilage development [GO:0051216]; cell activation [GO:0001775]; forebrain development [GO:0030900]; larval heart development [GO:0007508]; motor neuron axon guidance [GO:0008045]; neural crest cell migration [GO:0001755]; olfactory bulb axon guidance [GO:0071678]; positive regulation of cell migration involved in sprouting angiogenesis [GO:0090050]; positive regulation of endothelial cell migration [GO:0010595]; positive regulation of filopodium assembly [GO:0051491]; regulation of angiogenesis [GO:0045765]; regulation of axonogenesis [GO:0050770]; regulation of photoreceptor cell axon guidance [GO:2000289]; regulation of retinal ganglion cell axon guidance [GO:0090259]; regulation of vascular [illegible]

1074.64	1025.85	331.78	25.42	94.38	0	27.71	1229.38	4392.15	1385.67	1242.61	302.71	317.22	3127.22	1361.19	67.02	741.45	3306.02	20052.42	Mitochondrial transcription rescue factor 1	Mtres1	regulation of mitochondrial transcription [GO:1903108]; rescue of stalled ribosome [GO:0072344]
970.63	1262.74	2163.66	307.24	1463.2	1457.97	460.3	557.81	1369.06	371.37	455.42	1287.63	1036.36	1617.25	1533.09	541.27	1098.13	2098.51	20051.64	Coiled-coil domain-containing protein 120	CCDC120 JM11	microtubule anchoring at centrosome [GO:0034454]; protein localization [GO:0008104]
38.04	0	10.15	114.23	0	14.12	11506.1	0	0	75.26	214.38	4.76	10.7	0	734.16	7108.47	179.16	3.24	20012.77	Pre-mRNA-processing factor 19 (EC 2.3.2.27) (PRP19/PSO4 homolog) (RING-type E3 ubiquitin transferase PRP19)	PRPF19	DNA damage checkpoint signaling [GO:0000077]; double-strand break repair via nonhomologous end joining [GO:0006303]; inner cell mass cell proliferation [GO:0001833]; lipid biosynthetic process [GO:0008610]; mRNA splicing, via spliceosome [GO:0000398]; positive regulation of mRNA splicing, via spliceosome [GO:0048026]; proteasomal protein catabolic process [GO:0010498]; protein K63-linked ubiquitination [GO:0070534]; protein localization [GO:0008104]; spliceosomal complex assembly [GO:0000245]; spliceosomal tri-snRNP complex assembly [GO:0000244]

337.17	1230.38	655.59	313.1	2271.71	1443.54	1932.72	786.6	1110.98	341.22	580.44	960.36	1070.37	3011.28	1405.07	780.47	741.18	1020.76	19992.94	Gag-Pol polyprotein [Cleaved into: Matrix protein p10 (MA);p20;Capsid protein p25 (CA);Nucleocapsid protein p14 (NC-pol);Protease p15 (PR) (EC 3.4.23.-);Reverse transcriptase/ribonuclease H p90 (RT) (EC 2.7.7.49) (EC 2.7.7.7) (EC 3.1.26.4);Integrase p46 (IN) (EC 2.7.7.-) (EC 3.1.-.-)]	gag-pol	DNA integration [GO:0015074]; DNA recombination [GO:0006310]; proteolysis [GO:0006508]
4.72	1.05	548.85	1.33	3.04	11.07	1.7	3.98	6877.84	1.53	2.94	1905.53	0.53	0.6	4603.94	5.81	5.77	5976.05	19956.28	Elongation factor 2b (EF-2) (EC 3.6.5.-)	eef2b zgc:63584	cellular response to xenobiotic stimulus [GO:0071466]; chordate embryonic development [GO:0043009]; translational elongation [GO:0006414]
43.09	332.44	56.29	33.28	1129.34	68.54	2.18	808.59	53.09	10.98	371.54	41.78	52.42	16129.38	60.56	16.92	658.75	58.42	19927.59	Vacuolar protein sorting-associated protein 37A (hVps37A) (ESCRT-I complex subunit VPS37A) (Hepatocellular carcinoma-related protein 1)	VPS37A HCRP1	macroautophagy [GO:0016236]; membrane fission [GO:0090148]; multivesicular body assembly [GO:0036258]; protein targeting to membrane [GO:0006612]; protein targeting to vacuole [GO:0006623]; protein transport to vacuole involved in ubiquitin-dependent protein catabolic process via the multivesicular body sorting pathway [GO:0043328]; ubiquitin-dependent protein catabolic process via the multivesicular body sorting pathway [GO:0043162]; viral budding via host ESCRT complex [GO:0039702]

294.89	2339.63	168.27	835.15	2223.83	2870.4	54.79	816.47	103.02	10.13	333.38	37.56	3463.71	1883.82	1095.73	560.18	2548.68	272.77	19912.41	Aminopeptidase N (AP-N) (bAPN) (EC 3.4.11.2) (Alanyl aminopeptidase) (Aminopeptidase M) (AP-M) (Microsomal aminopeptidase) (CD antigen CD13)	ANPEP APN	angiogenesis [GO:0001525]; cell differentiation [GO:0030154]; peptide catabolic process [GO:0043171]; proteolysis [GO:0006508]
2907.78	927.25	1375.68	259.26	882.66	1252.95	24.07	1346.83	95.95	1047.65	1176.51	590.92	4264.57	1703.8	677.49	90.89	888.45	383.42	19896.13	Putative fibroblast growth factor 1 (FGF-1) (Acidic fibroblast growth factor) (aFGF) (Heparin-binding growth factor 1) (HBGF-1)	fgf1 fgf-1 zgc:73249	angiogenesis [GO:0001525]; branch elongation involved in ureteric bud branching [GO:0060681]; fibroblast growth factor receptor signaling pathway [GO:0008543]; hemopoiesis [GO:0030097]; mesonephric epithelium development [GO:0072163]; neurogenesis [GO:0022008]; positive regulation of angiogenesis [GO:0045766]; positive regulation of cell division [GO:0051781]; positive regulation of cell migration [GO:0030335]; positive regulation of cell population proliferation [GO:0008284]; positive regulation of cholesterol biosynthetic process [GO:0045542]; positive regulation of intracellular signal transduction [GO:1902533]; positive regulation of MAPK cascade [GO:0043410]; positive regulation of transcription by RNA polymerase II [GO:0045944]; regulation of cell migration [GO:0030334]
130.2	43.02	93.7	615.89	26	122.88	10700.27	18.23	65.05	97.78	222.02	73.39	97.48	75.68	58.29	7181.53	196.4	70.55	19888.36	Myosin-binding protein H (MyBP-H) (86 kDa protein) (H-protein)	MYBPH	cell adhesion [GO:0007155]; sarcomere organization [GO:0045214]
17	4340.35	6.21	26.95	3629.28	34.79	0.85	3401.48	11.15	2.6	905.73	4.78	4.52	4290.07	4.13	5.79	3154.84	21.32	19861.84	DISP complex protein LRCH3 (Leucine-rich repeat and calponin homology domain-containing protein 3)	Lrch3	septin cytoskeleton organization [GO:0032185]

149.16	4604.46	234.64	504.06	4214.92	835.13	21.04	1451.32	93.05	64.13	1089.15	425.84	84.89	3662.7	121.46	121.54	2015.17	114.51	19807.17	Embryonal Fyn-associated substrate (hEFS) (Cas scaffolding protein family member 3)	EFS CASS3	cell adhesion [GO:0007155]; cell migration [GO:0016477]; cell surface receptor protein tyrosine kinase signaling pathway [GO:0007169]; intracellular signal transduction [GO:0035556]
1264.68	1591.59	1235.75	375.23	2058.08	2465.09	23.98	1005.1	475.39	503.79	817.47	943.29	1026.73	2468.77	825.96	194.96	1468.36	1060.48	19804.7	Translation initiation factor IF-2, mitochondrial (IF-2(Mt)) (IF-2Mt) (IF2(mt))	Mtif2	mitochondrial translational initiation [GO:0070124]; ribosome disassembly [GO:0032790]
2541.4	745	2239.4	638.23	756.02	2143.81	1077.15	255.29	993.86	877.68	236.96	1522.3	1342.47	1000.1	1055.19	732.09	647.73	998.73	19803.41	Acyl-CoA:lysophosphatidylglycerol acyltransferase 1 (2-acylglycerophosphocholine O-acyltransferase) (EC 2.3.1.62) (Acyl-CoA:monoacylglycerol acyltransferase LPGAT1) (EC 2.3.1.22) (Lysophospholipid acyltransferase 7) (LPLAT7) (EC 2.3.1.-) (Stearoyl-CoA:1-lyso-2-acyl-PE acyltransferase)	LPGAT1 FAM34A KIAA0205	phosphatidylethanolamine acyl-chain remodeling [GO:0036152]; phosphatidylglycerol acyl-chain remodeling [GO:0036148]; phosphatidylinositol acyl-chain remodeling [GO:0036149]; phospholipid biosynthetic process [GO:0008654]; positive regulation of fatty acid biosynthetic process [GO:0045723]; triglyceride biosynthetic process [GO:0019432]

903.11	866.29	1304.29	385.39	554.46	1090.13	2445.71	329.73	1574.07	627.48	415.21	1083.17	1522.96	849.62	1476.2	2260.87	549.95	1559.92	19798.56	Formin-like protein 2 (Formin homology 2 domain-containing protein 2)	FMNL2 FHOD2 KIAA1902	cell migration [GO:0016477]; cortical actin cytoskeleton organization [GO:0030866]; cytoskeleton organization [GO:0007010]; regulation of cell morphogenesis [GO:0022604]; regulation of cell shape [GO:0008360]
665.16	735.84	609.05	699.4	2922.44	3075.14	100.63	808.2	80.52	358.49	507.94	366.08	2729.52	3414.83	1255.39	185.56	1002.87	262.5	19779.56	SLIT and NTRK-like protein 4	Slitrk4	axonogenesis [GO:0007409]; behavioral fear response [GO:0001662]; cell population proliferation [GO:0008283]; long-term synaptic potentiation [GO:0060291]; memory [GO:0007613]; positive regulation of synapse assembly [GO:0051965]; smoothened signaling pathway [GO:0007224]

34.47	0	0	202.82	0	4.82	11000.4	0	0	90.9	252.05	0	7.27	0	2.02	7945.8	210.73	6.14	19757.42	Anoctamin-1 (Transmembrane protein 16A)	Ano1 Tmem16a	cellular response to heat [GO:0034605]; chloride transmembrane transport [GO:1902476]; chloride transport [GO:0006821]; detection of temperature stimulus involved in sensory perception of pain [GO:0050965]; establishment of localization in cell [GO:0051649]; glial cell projection elongation [GO:0106091]; mucus secretion [GO:0070254]; phospholipase C-activating G protein-coupled receptor signaling pathway [GO:0007200]; positive regulation of insulin secretion involved in cellular response to glucose stimulus [GO:0035774]; regulation of membrane potential [GO:0042391]; trachea development [GO:0060438]
1225.55	864.02	1844.21	814.85	726.63	2018.74	1133.09	448.79	898.65	1150.88	721.58	1950	1297.65	842.62	898.78	1096.94	829.93	992.68	19755.59	Sphingosine-1-phosphate phosphatase 1 (SPPase1) (Spp1) (EC 3.1.3.-) (Sphingosine-1-phosphatase 1)	Sgpp1	ER to Golgi ceramide transport [GO:0035621]; extrinsic apoptotic signaling pathway [GO:0097191]; intrinsic apoptotic signaling pathway [GO:0097193]; phospholipid dephosphorylation [GO:0046839]; regulation of epidermis development [GO:0045682]; regulation of keratinocyte differentiation [GO:0045616]; sphinganine-1-phosphate metabolic process [GO:0006668]; sphingolipid metabolic process [GO:0006665]; sphingosine metabolic process [GO:0006670]
416.62	357.22	1612.83	145.13	333.35	731.76	613.4	140.16	4282.34	136.48	124.3	1499.17	410.19	484.38	2977.45	560.11	346.72	4527.71	19699.32	Regulator of G-protein signaling 3 (RGS3) (SRB-RGS)	Rgs3	negative regulation of signal transduction [GO:0009968]

523.27	626.96	727.34	373.44	767.1	1317.11	6477.3	281.9	274.2	272.37	375.34	526.72	970.67	640.29	727.5	3931.12	443.63	439.56	19695.82	PHD finger protein 20 (Hepatocellular carcinoma-associated antigen 58 homolog)	Phf20 Hca58	chromatin organization [GO:0006325]; positive regulation of DNA-templated transcription [GO:0045893]
770.58	729.95	1120.06	256.09	745.72	1075.94	2576.7	322.28	2917.72	414.52	357.23	1055.84	751.68	823.61	1872.34	1247.49	516.39	2121.18	19675.32	Acetyl-CoA carboxylase (ACC) (EC 6.4.1.2) [Includes: Biotin carboxylase (EC 6.3.4.14)]	ACAC	epigenetic regulation of gene expression [GO:0040029]; fatty acid biosynthetic process [GO:0006633]; malonyl-CoA biosynthetic process [GO:2001295]; positive regulation of DNA-templated transcription [GO:0045893]; positive regulation of gene expression [GO:0010628]; regulation of biological quality [GO:0065008]; response to carbohydrate [GO:0009743]; response to fatty acid [GO:0070542]; response to thyroid hormone [GO:0097066]; transport [GO:0006810]
6.83	1.59	6.94	53.8	0	4.24	12544.12	0	0	60.16	278.29	0	3.31	0	0	6579.44	132.93	0	19671.65	Protein O-mannose kinase (POMK) (EC 2.7.1.183) (Protein kinase-like protein SgK196) (Sugen kinase 196)	pomk sgk196 zgc:101572	carbohydrate phosphorylation [GO:0046835]; muscle structure development [GO:0061061]; protein O-linked glycosylation [GO:0006493]; swimming [GO:0036268]

883.55	603.78	831.36	230.92	339.49	893.05	6083.85	212.75	1145.92	306.48	259.55	596.97	895.7	719.62	1079.83	3056.24	446.28	1070.51	19655.85	DDB1- and CUL4-associated factor 7 (WD repeat-containing protein 68) (WD repeat-containing protein An11 homolog)	DCAF7 HAN11 WDR68	protein ubiquitination [GO:0016567]
2629.58	964.4	2906.23	467.65	500.59	1362.47	19.51	622.95	1687.59	395.18	269.75	1135.71	1318.42	1331.12	1592.66	256.84	645.89	1529.15	19635.69	Centriolar coiled-coil protein of 110 kDa (Centrosomal protein of 110 kDa) (CP110) (Cep110)	CCP110 CEP110 CP110 KIAA0419	centriole replication [GO:0007099]; centrosome duplication [GO:0051298]; ciliary basal body organization [GO:0032053]; negative regulation of cilium assembly [GO:1902018]; positive regulation of cilium assembly [GO:0045724]; regulation of cytokinesis [GO:0032465]
1190.91	1919.95	1085.71	277.34	593.49	2824.92	207.68	451.56	1884.7	437.98	425.43	1016.35	810.19	1557.22	1880.36	210.6	742.89	2112.1	19629.38	Chloride anion exchanger (Down-regulated in adenoma) (Protein DRA) (Solute carrier family 26 member 3)	Slc26a3 Dra	cellular response to cAMP [GO:0071320]; intracellular pH elevation [GO:0051454]; membrane hyperpolarization [GO:0060081]; sperm capacitation [GO:0048240]

696.45		442.37	893.91	262.24	455.97	780.75	5516.84	229.04	619.23	263.46	328.07	550.51	872.04	544.56	739.83	5226.24	469.09	694.79	19585.39	Protein RCC2 homolog	rcc2 zgc:77115	cell division [GO:0051301]; chromosome passenger complex localization to kinetochore [GO:0072356]; neural crest cell migration [GO:0001755]; positive regulation of attachment of spindle microtubules to kinetochore [GO:0051987]
481.76		425.03	3239.56	127.75	397.74	846.25	4630.8	216.25	1763.89	204.97	231.89	759.94	214.65	229.5	1609.23	2098.79	277.89	1821.42	19577.31	Ribonucleoside-diphosphate reductase large subunit (EC 1.17.4.1) (Ribonucleoside-diphosphate reductase subunit M1) (Ribonucleotide reductase large subunit) (Ribonucleotide reductase protein R1 class I)	rrm1	deoxyribonucleotide biosynthetic process [GO:0009263]

476.81	111.3	3488.18	212.75	196.35	815.85	1721.79	55	1733.8	234.37	145.18	4132.9	799.76	253.63	1144.32	1850.37	265.59	1922.84	19560.79	Transcription initiation factor TFIID subunit 5 (Transcription initiation factor TFIID 100 kDa subunit) (TAF(II)100) (TAFII-100) (TAFII100)	TAF5 TAF2D	DNA-templated transcription initiation [GO:0006352]; mRNA transcription by RNA polymerase II [GO:0042789]; positive regulation of DNA-templated transcription [GO:0045893]; positive regulation of transcription initiation by RNA polymerase II [GO:0060261]; regulation of DNA repair [GO:0006282]; regulation of transcription by RNA polymerase II [GO:0006357]; RNA polymerase II preinitiation complex assembly [GO:0051123]; transcription initiation at RNA polymerase II promoter [GO:0006367]
775.89	820.87	655.54	402.67	675.26	1221.53	4998.09	301.18	648.32	238.52	299.63	473.97	1373.73	922.37	1039.74	3351.59	565.45	795.83	19560.18	Protein GREB1	Greb1 Kiaa0575	
827.29	1296.95	460.88	2612.76	1591.35	462.33	1025.77	998.6	305.17	1296.56	1699.98	493.65	1408.74	1482.8	351.87	1230.66	1650.61	362.4	19558.37	Rho-related GTP-binding protein Rho6 (Rho family GTPase 1) (Rnd1)	RND1 RHO6	actin filament organization [GO:0007015]; negative regulation of cell adhesion [GO:0007162]; neuron remodeling [GO:0016322]; regulation of actin cytoskeleton organization [GO:0032956]; signal transduction [GO:0007165]; small GTPase-mediated signal transduction [GO:0007264]

975.48		810.77	1065.9	494.45	865.81	1704.7	2302.37	377.71	824.26	408.95	540.85	1356.45	1438.64	2434.98	744.95	1561.57	884.93	760.39	19553.16	Ubiquitin-conjugating enzyme E2 G1 (EC 2.3.2.23) (E2 ubiquitin-conjugating enzyme G1) (E217K) (UBC7) (Ubiquitin carrier protein G1) (Ubiquitin-protein ligase G1) [Cleaved into: Ubiquitin-conjugating enzyme E2 G1, N-terminally processed]	UBE2G1 UBE2G	proteasome-mediated ubiquitin-dependent protein catabolic process [GO:0043161]; protein K48-linked ubiquitination [GO:0070936]; protein K63-linked ubiquitination [GO:0070534]; protein polyubiquitination [GO:0000209]; ubiquitin-dependent protein catabolic process [GO:0006511]
414.46		3624.78	570.53	166.71	3669.52	844.55	673.4	2562.86	468.23	119.78	661.05	1237.05	419.7	1306.74	821.67	394.6	829.52	757.5	19542.65	Insulin receptor substrate 2-B (IRS-2-B)	irs2-b	insulin receptor signaling pathway [GO:0008286]
2725.04		659.36	2579.36	523.2	471.26	4093.54	218.79	268.64	235.02	836.66	236.15	1049.23	3370.32	284.99	920.99	262.28	408.53	356.62	19499.98	Histone-lysine N-methyltransferase EZH1 (EC 2.1.1.356) (Enhancer of zeste homolog 1)	EZH1	heterochromatin formation [GO:0031507]; methylation [GO:0032259]
843.17		1306.95	1017.36	702.76	1153.7	1769.33	5067.2	1192.99	2419.22	7.89	44.96	178.54	3.48	30.07	31.54	1864.73	1167.68	673.89	19475.46	6-phosphogluconate dehydrogenase, decarboxylating (EC 1.1.1.44)	Pgd	D-gluconate metabolic process [GO:0019521]; NADP metabolic process [GO:0006739]; pentose biosynthetic process [GO:0019322]; pentose-phosphate shunt [GO:0006098]; pentose-phosphate shunt, oxidative branch [GO:0009051]

38.31		58.97	1662.62	54.38	35.57	253.73	165.65	15.71	5998.94	15.06	21.98	1757.8	32.52	369.5	3521.91	206.43	147.9	5080.25	19437.23	Lysine-specific histone demethylase 1A (EC 1.14.99.66) (BRAF35-HDAC complex protein BHC110) (Flavin-containing amine oxidase domain-containing protein 2) ([histone H3]-dimethyl-L-lysine(4) FAD-dependent demethylase 1A)	KDM1A AOF2 KDM1 KIAA0601 LSD1	cellular response to cAMP [GO:0071320]; cellular response to gamma radiation [GO:0071480]; cellular response to UV [GO:0034644]; cerebral cortex development [GO:0021987]; DNA repair-dependent chromatin remodeling [GO:0140861]; epigenetic regulation of gene expression [GO:0040029]; guanine metabolic process [GO:0046098]; muscle cell development [GO:0055001]; negative regulation of DNA damage response, signal transduction by p53 class mediator [GO:0043518]; negative regulation of intrinsic apoptotic signaling pathway by p53 class mediator [GO:1902254]; negative regulation of intrinsic apoptotic signaling pathway in response to DNA damage by p53 class mediator [GO:1902166]; negative regulation of transcription by RNA polymerase II [GO:0000122]; negative regulation of transcription initiation-coupled chromatin remodeling [GO:0160217]; neuron maturation [GO:0042551]; positive regulation of cell
1521		2202.29	97	2227.65	1425.52	875.98	36.06	1015.3	6.16	1575.67	2027.73	32.62	713.18	1687.28	440.09	1314.94	2186.32	42.64	19427.43	Focal adhesion kinase 1 (FADK 1) (EC 2.7.10.2) (Focal adhesion kinase-related nonkinase) (FRNK) (p41/p43FRNK) (Protein-tyrosine kinase 2) (p125FAK) (pp125FAK)	PTK2 FAK FAK1	actin filament organization [GO:0007015]; angiogenesis involved in wound healing [GO:0060055]; epidermal growth factor receptor signaling pathway [GO:0007173]; negative regulation of anoikis [GO:2000811]; negative regulation of cell-substrate adhesion [GO:0010812]; negative regulation of protein autophosphorylation [GO:0031953]; positive regulation of cell migration [GO:0030335]; positive regulation of cell population proliferation [GO:0008284]; positive regulation of focal adhesion assembly [GO:0051894]; positive regulation of protein binding [GO:0032092]; positive regulation of protein tyrosine kinase activity [GO:0061098]; positive regulation of substrate-dependent cell migration, cell attachment to substrate [GO:1904237]; protein autophosphorylation [GO:0046777]; radial glia-guided pyramidal neuron migration [GO:0140650]; regulation of cell adhesion [GO:0030155]; response

1389.05	688.22	2870.84	381.98	460.35	2186.53	601.59	150.13	3030.87	339.12	139.24	1539.38	907.1	470.1	1256.18	400.6	448.06	2166.2	19425.54	Glutamate decarboxylase 1 (EC 4.1.1.15) (67 kDa glutamic acid decarboxylase) (GAD-67) (Glutamate decarboxylase 67 kDa isoform)	GAD1 GAD67	gamma-aminobutyric acid biosynthetic process [GO:0009449]; glutamate catabolic process [GO:0006538]; locomotory exploration behavior [GO:0035641]; social behavior [GO:0035176]
1366.27	1059.17	1086.89	354.73	1230.64	1285.01	394.3	1498.74	1341.51	434.51	884.87	860.77	935.6	2313.65	1157.12	425.81	1399.39	1285.81	19314.79	Protein Niban 2 (Meg-3) (Niban-like protein 1) (Protein FAM129B)	Niban2 Fam129b	gonadotropin secretion [GO:0032274]; negative regulation of apoptotic process [GO:0043066]; negative regulation of cell population proliferation [GO:0008285]; negative regulation of DNA biosynthetic process [GO:2000279]; negative regulation of DNA-templated transcription [GO:0045892]; positive regulation of DNA-templated transcription [GO:0045893]

3401.55	758.34	1003.09	832.7	692.58	1314.28	693.46	246.55	669.06	814.02	334.2	804.5	3591.51	1041.71	1007.18	707.84	707	656.43	19276	DNA topoisomerase 3-alpha (EC 5.6.2.1) (DNA topoisomerase III alpha)	TOP3A TOP3	chromosome separation [GO:0051304]; DNA recombination [GO:0006310]; DNA repair [GO:0006281]; DNA topological change [GO:0006265]; double-strand break repair via homologous recombination [GO:0000724]; meiotic cell cycle [GO:0051321]; mitochondrial DNA metabolic process [GO:0032042]; resolution of DNA recombination intermediates [GO:0071139]
51.4	256.22	4.5	89.22	194.15	1.89	0.8	50.06	0	119.13	888.04	36.44	16.39	4759.15	1.32	9593.89	3162.78	42.88	19268.26	Interleukin-17 receptor E-like protein (IL-17 receptor E-like) (IL-17RE-like)	IL17REL	
4302.52	638.36	1331.94	737.88	717.85	1554.44	37.67	227.14	1186.98	1120.99	274.91	749.28	2307.81	971.97	1358.07	204.74	457.45	1068.3	19248.3	F-BAR and double SH3 domains protein 2 (Carom) (Protein nervous wreck 1) (NWK1) (SH3 multiple domains protein 3)	FCHSD2 KIAA0769 SH3MD3	clathrin-dependent endocytosis [GO:0072583]; membrane organization [GO:0061024]; neuromuscular synaptic transmission [GO:0007274]; positive regulation of actin filament polymerization [GO:0030838]; positive regulation of Arp2/3 complex-mediated actin nucleation [GO:2000601]; protein transport [GO:0015031]; regulation of actin filament polymerization [GO:0030833]

1476.53	4520.22	173.51	174.34	2140.05	310.04	0.86	1403.73	23.47	223.27	474.43	109.29	225.22	4842.15	60.75	70.91	2935.79	75.41	19239.97	EH domain-containing protein 4 (PAST homolog 2) (mPAST2)	Ehd4 Past2	cell migration involved in sprouting angiogenesis [GO:0002042]; cellular response to growth factor stimulus [GO:0071363]; endocytic recycling [GO:0032456]; pinocytosis [GO:0006907]; protein homooligomerization [GO:0051260]; regulation of endocytosis [GO:0030100]
426.27	252.99	350.33	232.22	256.05	526.49	6550.43	134.4	266.5	197.99	319.91	230.04	494.58	329.09	329.82	7621.82	413.87	299.98	19232.78	Huntingtin-interacting protein 1-related protein (HIP1-related protein)	Hip1r	clathrin coat assembly [GO:0048268]; digestive system development [GO:0055123]; intrinsic apoptotic signaling pathway [GO:0097193]; membrane organization [GO:0061024]; negative regulation of actin filament polymerization [GO:0030837]; negative regulation of apoptotic process [GO:0043066]; negative regulation of Arp2/3 complex-mediated actin nucleation [GO:0034316]; positive regulation of clathrin coat assembly [GO:1905445]; positive regulation of clathrin-dependent endocytosis [GO:2000370]; positive regulation of epidermal growth factor receptor signaling pathway [GO:0045742]; positive regulation of mitochondrial outer membrane permeabilization involved in apoptotic signaling pathway [GO:1901030]; positive regulation of platelet-derived growth factor receptor-beta signaling pathway [GO:2000588]; postsynapse organization [GO:0099173]; protein stabilization [GO:0050821]; receptor-mediated [illegible]

100.65	5224.48	512.22	272.72	3094.58	152.34	2.38	1847.08	153.94	116.38	1078.5	201.92	135.35	3776.1	247.14	24.63	2032.2	260.15	19232.76	Frizzled-7-A (Fz-7-A) (Xfz7-A)	fzd7-a fz7-a	canonical Wnt signaling pathway [GO:0060070]; convergent extension involved in gastrulation [GO:0060027]; dorsal/ventral axis specification [GO:0009950]; mesoderm formation [GO:0001707]; non-canonical Wnt signaling pathway [GO:0035567]; positive regulation of transcription by RNA polymerase II [GO:0045944]; protein localization [GO:0008104]; tissue morphogenesis [GO:0048729]; Wnt signaling pathway [GO:0016055]
1785.1	788.21	1039.87	522.61	501.43	1148.15	2328.98	227	495.7	684.53	259.68	1064.87	1438	731.87	678.08	3310.55	1355.79	823.32	19183.74	Solute carrier family 2, facilitated glucose transporter member 5 (Fructose transporter) (Glucose transporter type 5, small intestine) (GLUT-5)	Slc2a5 Glut5	cellular response to fructose stimulus [GO:0071332]; D-glucose import [GO:0046323]; D-glucose transmembrane transport [GO:1904659]; dehydroascorbic acid transport [GO:0070837]; fructose import across plasma membrane [GO:1990539]; fructose transmembrane transport [GO:0015755]; metanephric proximal tubule development [GO:0072237]; regulation of systemic arterial blood pressure mediated by a chemical signal [GO:0003044]; response to fructose [GO:0009750]
1237.16	1242.05	1408.54	470.91	1207.85	1628.32	1499.76	680.97	807.28	373.54	467.91	1087.18	1043.35	1940	763.43	1349.7	1090.95	825.28	19124.18	Fibulin-2 (FIBL-2)	FBLN2	positive regulation of cell-substrate adhesion [GO:0010811]

2084.51	1064.26	1441.67	964.79	1485.92	1575.98	1936.2	598.84	330.34	557.27	616.77	654.07	1202.74	1335.07	428.8	1346.46	1074.18	356.44	19054.31	CXXC-type zinc finger protein 4	cxxc4	Wnt signaling pathway [GO:0016055]; zygotic specification of dorsal/ventral axis [GO:0007352]
2218.33	750.24	2039.35	530.98	1405.65	1627.48	29.66	443.32	826.85	1027.7	647.5	1585.51	1155.31	568.04	927.58	285.2	2139.01	833.78	19041.49	tRNA N(3)-cytidine methyltransferase METTL6 (EC 2.1.1.-) (Methyltransferase-like protein 6) (hMETTL6)	METTL6	tRNA methylation [GO:0030488]; tRNA modification [GO:0006400]
89.22	16.21	11.63	376.33	19.8	26.72	10394.01	5.85	3.28	127.38	234.26	14.15	275.7	22.37	24.31	7159.69	185.38	4.59	18990.88	Carboxyl-terminal PDZ ligand of neuronal nitric oxide synthase protein (C-terminal PDZ ligand of neuronal nitric oxide synthase protein) (Nitric oxide synthase 1 adaptor protein)	Nos1ap Capon Kiaa0464	postsynaptic actin cytoskeleton organization [GO:0098974]; regulation of heart rate by chemical signal [GO:0003062]; regulation of ventricular cardiac muscle cell membrane repolarization [GO:0060307]
812.79	349.02	1720.27	355.1	2543.76	5801.21	6.26	446.89	230.15	285.25	300.38	417.95	1212.17	670.44	488.85	316.65	1358.94	1631.4	18947.48	Transient receptor potential cation channel subfamily M member 5	trpm5	

2041.72	1329.56	583.28	331.4	1528.44	629.48	393.24	895.3	237.5	429.58	594.26	302.32	2638.38	3100.99	776.15	538.51	2066.37	496.82	18913.3	Multiple C2 and transmembrane domain-containing protein 1	Mctp1	negative regulation of cell migration [GO:0030336]; negative regulation of endocytosis [GO:0045806]; negative regulation of response to oxidative stress [GO:1902883]
568.21	519.72	1099.66	263.06	477.47	1066.13	1755.65	149.15	2538.32	182.05	261.05	1327.47	1268.32	858.35	2273.33	980.7	475.63	2814.66	18878.93	Receptor-type tyrosine-protein phosphatase C (EC 3.1.3.48) (Leukocyte common antigen) (L-CA) (T200) (CD antigen CD45)	PTPRC CD45	alpha-beta T cell proliferation [GO:0046633]; B cell differentiation [GO:0030183]; B cell proliferation [GO:0042100]; B cell receptor signaling pathway [GO:0050853]; bone marrow development [GO:0048539]; cell cycle phase transition [GO:0044770]; cell surface receptor signaling pathway [GO:0007166]; defense response to virus [GO:0051607]; dephosphorylation [GO:0016311]; DN2 thymocyte differentiation [GO:1904155]; extrinsic apoptotic signaling pathway [GO:0097191]; gamma-delta T cell differentiation [GO:0042492]; hematopoietic progenitor cell differentiation [GO:0002244]; heterotypic cell-cell adhesion [GO:0034113]; leukocyte cell-cell adhesion [GO:0007159]; MAPK cascade [GO:0000165]; natural killer cell differentiation [GO:0001779]; negative regulation of cell adhesion involved in substrate-bound cell migration [GO:0006933]; negative regulation of cytokine-mediated signaling pathway

1068.08	868.1	1013.05	420.5	931.34	1423.66	2946.74	387.12	1288.78	368.62	275.86	969.91	1386.85	941.42	1252.07	1201.38	623	1461.72	18828.2	Mediator of RNA polymerase II transcription subunit 13-like (Mediator complex subunit 13-like) (Thyroid hormone receptor-associated protein 2) (Thyroid hormone receptor-associated protein complex 240 kDa component-like)	MED13L KIAA1025 PROSIT240 THRAP2 TRAP240L	positive regulation of transcription by RNA polymerase II [GO:0045944]
0	0	0	0	0	0	12322.49	0.79	0	42.32	0	4.57	0	0	0	6211.07	210.93	16.38	18808.55	RNA-directed DNA polymerase homolog (EC 2.7.7.48) (Reverse transcriptase homolog)		
1758.11	1430.91	1279.68	545.76	1809.46	2999.89	69.91	805.17	109.12	656.93	741.36	851.07	1513.01	1468.19	660.74	263.78	1270.86	548.44	18782.39	Protein capicua homolog	Cic Kiaa0306	brain development [GO:0007420]; learning [GO:0007612]; lung alveolus development [GO:0048286]; memory [GO:0007613]; negative regulation of DNA-templated transcription [GO:0045892]; negative regulation of transcription by RNA polymerase II [GO:0000122]; social behavior [GO:0035176]; transcription by RNA polymerase II [GO:0006366]

1.79	0.23	925.89	0.88	1.31	13.1	9.98	0	4361.11	0.71	1.48	1805.01	5.66	3.89	5140.83	4.37	4.17	6499.36	18779.77	Intraflagellar transport protein 140 homolog (WD and tetratricopeptide repeats protein 2)	Ift140 WDTC2	cilium assembly [GO:0060271]; determination of left/right symmetry [GO:0007368]; embryonic brain development [GO:1990403]; embryonic camera-type eye development [GO:0031076]; embryonic cranial skeleton morphogenesis [GO:0048701]; embryonic digit morphogenesis [GO:0042733]; heart development [GO:0007507]; intraciliary retrograde transport [GO:0035721]; intraciliary transport [GO:0042073]; limb morphogenesis [GO:0035108]; neural tube patterning [GO:0021532]; non-motile cilium assembly [GO:1905515]; photoreceptor cell outer segment organization [GO:0035845]; protein localization to cilium [GO:0061512]; regulation of cilium assembly [GO:1902017]; regulation of smoothened signaling pathway [GO:0008589]
591.86	1207.81	543.34	957.59	1217.9	486.51	274.52	1985.53	361.11	864.03	1405.61	1202.88	1724.32	3430.18	579.01	365.55	1224.49	329.29	18751.53	Transmembrane protein 222	TMEM222 C1orf160	
660.5	1745.94	903.56	234.42	1775.86	1484.97	603.33	1165.74	79.17	307.57	929.06	828.95	565.31	3205.6	723.97	871.21	2422.42	219.77	18727.35	Transcription initiation factor TFIID subunit 8 (Protein taube nuss-like) (TBP-associated factor 8)	taf8 tbnl	cell differentiation [GO:0030154]; transcription initiation at RNA polymerase II promoter [GO:0006367]
930.98	404.4	977.49	420.1	2236.51	1198.86	534.1	440.9	621.42	440.61	274.3	569.61	2241.06	3203.77	1438.31	528.28	1177.58	1084.76	18723.04	WD repeat-containing protein 19 (Intraflagellar transport 144 homolog)	Wdr19 Ift144 Kiaa1638	cell morphogenesis [GO:0000902]; cilium assembly [GO:0060271]; digestive system development [GO:0055123]; ear morphogenesis [GO:0042471]; embryonic camera-type eye development [GO:0031076]; embryonic cranial skeleton morphogenesis [GO:0048701]; embryonic limb morphogenesis [GO:0030326]; gonad development [GO:0008406]; in utero embryonic development [GO:0001701]; intraciliary retrograde transport [GO:0035721]; myotome development [GO:0061055]; nervous system process [GO:0050877]; protein localization to ciliary membrane [GO:1903441]; protein localization to cilium [GO:0061512]; protein-containing complex assembly [GO:0065003]; receptor clustering [GO:0043113]; smoothened signaling pathway [GO:0007224]; smoothened signaling pathway involved in dorsal/ventral neural tube patterning [GO:0060831]

2.06	10.8	1962.67	3	0.71	14.55	0	0.56	6318.07	3.84	1.36	1768.5	12.01	8.45	2928.96	2.51	3.3	5673.53	18714.88	BMP/retinoic acid-inducible neural-specific protein 3	Brinp3 Fam5c	cellular response to retinoic acid [GO:0071300]; central nervous system neuron differentiation [GO:0021953]; exploration behavior [GO:0035640]; multicellular organism growth [GO:0035264]; negative regulation of mitotic cell cycle [GO:0045930]; nervous system development [GO:0007399]; positive regulation of neuron differentiation [GO:0045666]; social behavior [GO:0035176]
1389.16	1214.37	1160.67	579.14	1338.59	1657.74	2143.33	325.72	411.79	328.33	492.31	1211.4	1320.86	1574.16	570.25	1474.35	970.9	524.17	18687.24	Armadillo repeat-containing protein 6	Armc6	hematopoietic progenitor cell differentiation [GO:0002244]
25.19	0	0	456.77	2.68	30.5	8220.72	4.14	0	45.38	115.48	0	108.38	17.14	0	9355.4	263.85	5.33	18650.96	Iduronate 2-sulfatase (EC 3.1.6.13) (Alpha-L-iduronate sulfate sulfatase) (Idursulfase) [Cleaved into: Iduronate 2-sulfatase 42 kDa chain;Iduronate 2-sulfatase 14 kDa chain]	IDS SIDS	dermatan sulfate proteoglycan catabolic process [GO:0030209]; glycosaminoglycan catabolic process [GO:0006027]; heparan sulfate proteoglycan catabolic process [GO:0030200]
0.66	0.74	2139.82	0.41	0.68	424.6	6.59	0	31.43	0.46	0	15925.83	0.77	0	64.55	12.04	4.13	18.77	18631.48	Nuclear prelamin A recognition factor	narf zgc:92186	

31.97	84.68	2721.11	45.14	43.97	3448.11	65.16	20.16	38.12	120.21	118.13	11301.23	16.22	52.65	76.44	87.32	105.15	234.65	18610.42	Transcription factor HIVEP3 (Human immunodeficiency virus type I enhancer-binding protein 3 homolog) (KB-binding and recognition component) (Kappa-B and V(D)J recombination signal sequences-binding protein) (Kappa-binding protein 1) (KBP-1) (Recombinant component) (Schnurri-3) (Zinc finger protein ZAS3)	Hivep3 KBP1 Kiaa1555 Krc Rc shn3 Zas3	positive regulation of DNA-templated transcription [GO:0045893]; skeletal muscle cell differentiation [GO:0035914]
511.87	1807.12	847.6	324.31	1669.33	625.41	1549.98	762.91	552.58	232.1	488.94	707.85	587.59	3577.02	747.47	1590.49	1193.24	822.61	18598.42	Fatty acid desaturase 6 (EC 1.14.19.-)	Fads6	fatty acid biosynthetic process [GO:0006633]
1053.66	771.75	1652.69	251.75	716.37	1356.94	432.81	438.77	2031.47	528.31	466.87	1340.97	865.87	1326.15	2327.54	312.67	639.83	2081.71	18596.13	Serine/threonine-protein kinase SMG1 (smg-1) (EC 2.7.11.1) (Nonsense-mediated mRNA decay-associated PI3K-related kinase SMG1)	smg1 atx	nuclear-transcribed mRNA catabolic process, nonsense-mediated decay [GO:0000184]
513.79	2510	1124.29	557.84	1632.17	886.47	820.35	1423.12	317.17	287.74	749.3	1421.62	775.82	1787.29	407.43	856.17	2152.29	363.79	18586.65	GPI ethanolamine phosphate transferase 3, catalytic subunit (EC 2.-.-.-) (Phosphatidylinositol-glycan biosynthesis class O protein) (PIG-O)	Pigo	GPI anchor biosynthetic process [GO:0006506]

1412.67	690.66	1437.93	369	918.57	1049.97	213.5	341.58	1906.23	411.65	244.66	1094.47	1560.5	1149.69	1663.3	603.56	739.46	2755.16	18562.56	SAM domain-containing protein SAMSN-1 (SAM domain, SH3 domain and nuclear localization signals protein 1) (SH3 protein expressed in lymphocytes 2) (SH3-lymphocyte protein 2) (SLy2)	Samsn1	negative regulation of adaptive immune response [GO:0002820]; negative regulation of B cell activation [GO:0050869]; negative regulation of peptidyl-tyrosine phosphorylation [GO:0050732]
681.96	731.76	1454.9	416.23	847.98	1206.84	3700.26	239.46	938.35	218.11	330.05	823.04	992.35	1025.67	1178.44	2150.93	569.32	1051.73	18557.38	AN1-type zinc finger protein 2B (Arsenite-inducible RNA-associated protein-like protein) (AIRAP-like protein)	ZFAND2B AIRAPL	proteasome-mediated ubiquitin-dependent protein catabolic process [GO:0043161]; protein targeting to ER [GO:0045047]; regulation of insulin-like growth factor receptor signaling pathway [GO:0043567]; SRP-dependent cotranslational protein targeting to membrane, translocation [GO:0006616]

1053.95	1152.9	927.7	452.05	1278.33	1629.5	3225.84	532.26	617.95	346.37	425.34	766.3	1503	1225.24	903.31	1464.1	408.76	632.14	18545.04	E3 ubiquitin-protein ligase MSL2 (EC 2.3.2.27) (Male-specific lethal-2 homolog) (MSL-2)	MSL2	DNA damage response [GO:0006974]; epigenetic regulation of gene expression [GO:0040029]; positive regulation of DNA-templated transcription [GO:0045893]; protein monoubiquitination [GO:0006513]; protein ubiquitination [GO:0016567]
1400.84	849.81	1557.29	276.68	741.43	1328.06	1867.31	332.21	1498.92	311.65	256.34	949.45	1029.77	1104.06	1492.06	1367.96	606.65	1574.3	18544.79	Probable RNA-directed DNA polymerase from transposon BS (EC 2.7.7.49) (Reverse transcriptase)	RTase	DNA transposition [GO:0006313]
21.58	24.65	488.59	10.86	12.4	5.61	44.28	1.72	6146.97	4.81	1.28	2004.95	60.09	22.86	3900.76	93.92	11.82	5674.73	18531.88	Alkaline phosphatase, tissue-nonspecific isozyme (AP-TNAP) (TNS-ALP) (TNSALP) (EC 3.1.3.1) (Alkaline phosphatase liver/bone/kidney isozyme) (Phosphoamidase) (Phosphocreatine phosphatase) (EC 3.9.1.1)	ALPL	biomineral tissue development [GO:0031214]; bone mineralization [GO:0030282]; calcium ion homeostasis [GO:0055074]; cellular homeostasis [GO:0019725]; cementum mineralization [GO:0071529]; developmental process involved in reproduction [GO:0003006]; endochondral ossification [GO:0001958]; futile creatine cycle [GO:0140651]; inhibition of non-skeletal tissue mineralization [GO:0140928]; osteoblast differentiation [GO:0001649]; phosphate ion homeostasis [GO:0055062]; positive regulation of cold-induced thermogenesis [GO:0120162]; pyridoxal phosphate metabolic process [GO:0042822]; response to antibiotic [GO:0046677]; response to glucocorticoid [GO:0051384]; response to insulin [GO:0032868]; response to lipopolysaccharide [GO:0032496]; response to macrophage colony-stimulating factor [GO:0036005]; response to sodium phosphate [illegible]

847.39	662.74	1000.88	292.07	854.99	689.77	398.59	287.32	2332.9	231.52	228.95	1159.5	1249.73	1608.73	2564.63	681.5	729.73	2709.49	18530.43	Retrovirus-related Pol polyprotein from type-2 retrotransposable element R2DM (Retrovirus-related Pol polyprotein from type II retrotransposable element R2DM) [Includes: Protease (EC 3.4.23.-);Reverse transcriptase (EC 2.7.7.49);Endonuclease]	pol	proteolysis [GO:0006508]
106.13	12.73	16.55	310.04	13.68	40.93	9070.8	0	137.2	145.01	145.82	97.29	1051.29	215.47	809.83	5790.69	227.69	331.96	18523.11	Endophilin-A2 (Endophilin-2) (SH3 domain protein 2B) (SH3 domain-containing GRB2-like protein 1) (SH3p8)	Sh3gl1 Sh3p8	endocytosis [GO:0006897]; modulation of excitatory postsynaptic potential [GO:0098815]; positive regulation of synaptic vesicle endocytosis [GO:1900244]; regulation of synaptic vesicle endocytosis [GO:1900242]

1793.63	648.42	1836.97	436.03	578.48	980.08	1066.66	249.02	2057.31	516.11	304.89	812.48	1016.05	1545.7	1545.5	812.78	509.92	1776.82	18486.85	Hypoxanthine-guanine phosphoribosyltransferase (HGPRT) (HGPRTase) (EC 2.4.2.8)	HPRT1 HPRT	GMP catabolic process [GO:0046038]; guanine salvage [GO:0006178]; hypoxanthine metabolic process [GO:0046100]; hypoxanthine salvage [GO:0043103]; IMP metabolic process [GO:0046040]; IMP salvage [GO:0032264]; positive regulation of dopamine metabolic process [GO:0045964]; purine nucleotide biosynthetic process [GO:0006164]; purine ribonucleoside salvage [GO:0006166]
970.98	1050.22	1366.67	331.29	1102.1	1491.97	1129.23	1730.19	715.66	427.5	683.37	596.63	1128.97	1797.09	1197.62	968.89	826.84	936.23	18451.45	Aldo-keto reductase family 1 member D1 (EC 1.3.1.3) (3-oxo-5-beta-steroid 4-dehydrogenase) (Delta(4)-3-ketosteroid 5-beta-reductase) (Delta(4)-3-oxosteroid 5-beta-reductase)	AKR1D1 SRD5B1	androgen metabolic process [GO:0008209]; bile acid biosynthetic process [GO:0006699]; bile acid catabolic process [GO:0030573]; C21-steroid hormone metabolic process [GO:0008207]; cholesterol catabolic process [GO:0006707]; digestion [GO:0007586]
722.8	789.84	654.6	172	774.77	1087.69	628.03	145.62	2532.41	214.7	141.1	1427.91	583.92	543.36	3560.05	378.69	282.07	3790.43	18429.99	YLP motif-containing protein 1 (Nuclear protein ZAP3) (ZAP113)	YLPM1 C14orf170 ZAP3	regulation of telomere maintenance [GO:0032204]

497.43		1661.39	621.76	297	1523.27	1492.63	1529.85	684.28	1060.01	169.49	348.63	2033.06	786.79	1402.76	890.06	918.68	1160.47	1311.37	18388.93	Diacylglycerol kinase epsilon (DAG kinase epsilon) (EC 2.7.1.107) (Diglyceride kinase epsilon) (DGK-epsilon)	DGKE DAGK5	diacylglycerol metabolic process [GO:0046339]; glycerolipid metabolic process [GO:0046486]; intracellular signal transduction [GO:0035556]; lipid phosphorylation [GO:0046834]; modulation of chemical synaptic transmission [GO:0050804]; phosphatidic acid biosynthetic process [GO:0006654]; phosphatidylinositol biosynthetic process [GO:0006661]; phospholipase C-activating G protein-coupled receptor signaling pathway [GO:0007200]; platelet activation [GO:0030168]
4170.23		1518.99	2025.16	446.87	2374.22	821.26	7.93	527.92	143.65	620.69	937.02	139.63	417.01	2225.2	89.96	290.74	1420.92	120.63	18298.03	Platelet endothelial cell adhesion molecule (PECAM-1) (CD antigen CD31)	Pecam1 Pecam Pecam-1	angiogenesis [GO:0001525]; bicellular tight junction assembly [GO:0070830]; cell adhesion [GO:0007155]; cell surface receptor signaling pathway [GO:0007166]; cellular response to mechanical stimulus [GO:0071260]; detection of mechanical stimulus [GO:0050982]; diapedesis [GO:0050904]; endothelial cell migration [GO:0043542]; endothelial cell morphogenesis [GO:0001886]; endothelial cell-matrix adhesion [GO:0090673]; establishment of endothelial barrier [GO:0061028]; glomerular endothelium development [GO:0072011]; homophilic cell adhesion via plasma membrane adhesion molecules [GO:0007156]; leukocyte cell-cell adhesion [GO:0007159]; monocyte extravasation [GO:0035696]; neutrophil extravasation [GO:0072672]; phagocytosis [GO:0006909]; positive regulation of cell migration [GO:0030335]; positive regulation of MAPK cascade [GO:0043410]; positive regulation of phosphatidylinositol 3-

667.12	713.86	2365.84	301.31	577.49	899.77	1706.31	253.82	1598.41	149.5	263.49	1463.66	1002.71	1108.19	1549.74	1432.46	558.01	1680.28	18291.97	Dual specificity protein phosphatase 26 (EC 3.1.3.16) (EC 3.1.3.48)	DUSP26	negative regulation of ERK1 and ERK2 cascade [GO:0070373]; negative regulation of transcription by RNA polymerase II [GO:0000122]; positive regulation of cell adhesion [GO:0045785]
149.41	3239.44	221.99	133.21	2624.67	341.38	11.34	2750.67	309.04	115.18	1126.64	242.49	190.7	1928.44	444.93	79.92	3709.81	661.21	18280.47	Melanopsin-A (Opsin-4A)	opn4a	phototransduction [GO:0007602]; visual perception [GO:0007601]
70.48	3.82	8.98	122.94	3.49	31.8	9865.24	1.38	5.39	77.72	333.73	5.25	28.88	3.33	13.22	7462.85	202.96	15.28	18256.74	RIMS-binding protein 2 (RIM-BP2)	RIMBP2 RBP2	neuromuscular synaptic transmission [GO:0007274]
2944.61	767.42	855.13	511.73	590.2	1348.82	374.3	373.88	543.06	671.27	282.11	645.03	667.96	3521.14	1006.05	605.78	1578.99	960.89	18248.37	Clusterin (51.5 kDa protein) [Cleaved into: Clusterin beta chain;Clusterin alpha chain]	CLU T64	positive regulation of apoptotic process [GO:0043065]; positive regulation of proteasomal ubiquitin-dependent protein catabolic process [GO:0032436]; positive regulation of receptor-mediated endocytosis [GO:0048260]; protein stabilization [GO:0050821]; regulation of cell population proliferation [GO:0042127]

1588.63	648.63	1559.06	443.68	666.35	884.16	153.27	380.47	2524.33	451.45	235.03	953.27	1550.72	1237.85	1791.83	329.64	616.95	2226.22	18241.54	Polycystin-2-like protein 1 (Polycystin-2L1) (Polycystic kidney disease 2-like 1 protein) (Polycystin-2 homolog)	Pkd2l1 Trpp3	cellular response to acidic pH [GO:0071468]; cellular response to pH [GO:0071467]; detection of chemical stimulus involved in sensory perception of sour taste [GO:0001581]; detection of chemical stimulus involved in sensory perception of taste [GO:0050912]; inorganic cation transmembrane transport [GO:0098662]; monoatomic cation transport [GO:0006812]; protein homotetramerization [GO:0051289]; protein tetramerization [GO:0051262]; response to water [GO:0009415]; smoothened signaling pathway [GO:0007224]
1045.61	916.21	1285.56	347.07	1049.18	1229.76	2093.99	482.35	1189.47	386.36	341.23	870.22	1088.58	1038.37	1200.98	1493.6	716.32	1420.98	18195.84	E3 ubiquitin-protein ligase TRIP12 (EC 2.3.2.26) (HECT-type E3 ubiquitin transferase TRIP12) (Thyroid receptor-interacting protein 12) (TR-interacting protein 12) (TRIP-12)	trip12 si:ch211-272f3.4	DNA damage response [GO:0006974]; DNA repair [GO:0006281]; DNA repair-dependent chromatin remodeling [GO:0140861]; heterochromatin boundary formation [GO:0033696]; proteasome-mediated ubiquitin-dependent protein catabolic process [GO:0043161]; protein polyubiquitination [GO:0000209]; regulation of embryonic development [GO:0045995]; ubiquitin-dependent protein catabolic process [GO:0006511]

0	22.36	4.4	4.51	67.21	1.19	4.09	1.46	4.61	0	18.43	0	0	14899	1.75	3.23	3144.65	15.36	18192.25	Neurosecretory protein VGF [Cleaved into: Neuroendocrine regulatory peptide-1 (NERP-1);Neuroendocrine regulatory peptide-2 (NERP-2);VGF-derived peptide TLQP-21;VGF-derived peptide TLQP-62;Antimicrobial peptide VGF[554-577]]	VGF	carbohydrate homeostasis [GO:0033500]; defense response to bacterium [GO:0042742]; generation of precursor metabolites and energy [GO:0006091]; glucose homeostasis [GO:0042593]; insulin secretion [GO:0030073]; ovarian follicle development [GO:0001541]; regulation of synaptic plasticity [GO:0048167]; response to cAMP [GO:0051591]; response to cold [GO:0009409]; response to dietary excess [GO:0002021]; response to insulin [GO:0032868]; sexual reproduction [GO:0019953]
1199.93	198.05	5381.95	308.55	238.62	1879.92	340.2	95.92	830.23	406.19	149.71	1062.92	958.22	247.05	1801.3	323.18	352.95	2396.1	18170.99	Low-density lipoprotein receptor-related protein 1B (LRP-1B) (Low-density lipoprotein receptor-related protein-deleted in tumor) (LRP-DIT)	LRP1B LRPDIT	protein transport [GO:0015031]; receptor-mediated endocytosis [GO:0006898]
33.34	2.25	321.47	5.86	2.81	230.63	0.33	3.05	4402.09	2.05	1.69	1695.81	52.52	2.75	4880.95	6.71	9.64	6516.9	18170.85	Tetratricopeptide repeat protein 7B (TPR repeat protein 7B) (Tetratricopeptide repeat protein 7-like-1) (TPR repeat protein 7-like-1)	TTC7B TTC7L1	phosphatidylinositol phosphate biosynthetic process [GO:0046854]; protein localization to plasma membrane [GO:0072659]

16.89	0	0	484.16	0	7.09	4259.7	0	1.1	89.14	182.15	0	8.38	0	7.5	12783.15	308.4	18.07	18165.73	E3 SUMO-protein ligase RanBP2 (EC 2.3.2.-) (358 kDa nucleoporin) (Nuclear pore complex protein Nup358) (Nucleoporin Nup358) (Ran-binding protein 2) (RanBP2) (p270)	RANBP2	intracellular transport [GO:0046907]; mRNA transport [GO:0051028]; protein folding [GO:0006457]; protein sumoylation [GO:0016925]; protein transport [GO:0015031]
969.19	2784.88	2674.24	772.84	1130.1	814.37	70.52	600.95	409.48	411.77	303.96	445.02	574.37	4423.39	724.93	234.05	391.63	417.43	18153.12	Leucine-rich repeat-containing protein 58	lrrc58	
248.98	96.26	1019.13	271.17	165.26	403.22	6957.28	58.45	464.86	145.56	286.36	530.54	268.49	263.01	373.86	5597.85	380.19	596.52	18126.99	Ryanodine receptor 1 (RYR-1) (RyR1) (Skeletal muscle calcium release channel) (Skeletal muscle ryanodine receptor) (Skeletal muscle-type ryanodine receptor) (Type 1 ryanodine receptor)	RYR1	calcium ion transmembrane transport [GO:0070588]; cellular response to caffeine [GO:0071313]; cellular response to calcium ion [GO:0071277]; intracellular calcium ion homeostasis [GO:0006874]; muscle contraction [GO:0006936]; ossification involved in bone maturation [GO:0043931]; outflow tract morphogenesis [GO:0003151]; protein homotetramerization [GO:0051289]; release of sequestered calcium ion into cytosol [GO:0051209]; release of sequestered calcium ion into cytosol by sarcoplasmic reticulum [GO:0014808]; skeletal muscle fiber development [GO:0048741]; skin development [GO:0043588]; striated muscle contraction [GO:0006941]
124.34	172.78	1412.92	122.99	136.7	162.21	218.13	62.98	4395.77	79.82	81.35	1506.47	465.4	422.34	3573.08	188.91	160.84	4839.94	18126.97	MOB kinase activator 1A (Mob1 alpha) (Mob1A) (Mob1 homolog 1B) (Mps one binder kinase activator-like 1B)	MOB1A C2orf6 MOB4B MOBK1B MOBKL1B	hippo signaling [GO:0035329]

2312.22	801.28	1116.54	501.47	609.98	986.41	2064.9	230.41	164.8	499.69	317.7	421.06	3801.41	866.47	772.5	1653.39	602.45	379.55	18102.23	Collectin-12 (Collectin placenta protein 1) (CL-P1)	COLEC12 CLP1	phagocytosis, recognition [GO:0006910]
59.74	44.11	1140.97	93.15	45.1	223.65	4961.04	17.99	2398.18	53.95	152.98	538.07	15.55	239.88	1258.69	4285.65	493.69	2069.05	18091.44	Oxysterol-binding protein-related protein 3 (ORP-3) (OSBP-related protein 3)	OSBPL3 KIAA0704 ORP3 OSBP3	bile acid biosynthetic process [GO:0006699]
281.56	5266.68	458.07	372.76	2429.57	1406.92	2.01	986.2	52.45	130.08	438.64	432.74	252.01	3768.93	429.75	49.32	1248.34	71.5	18077.53	V-type proton ATPase subunit H (V-ATPase subunit H) (V-ATPase 50/57 kDa subunits) (Vacuolar proton pump subunit H) (Vacuolar proton pump subunit SFD)	ATP6V1H	endocytosis [GO:0006897]; pH reduction [GO:0045851]; proton transmembrane transport [GO:1902600]
638.15	522.44	2854.63	357.28	658.16	1000.63	848.62	311.84	1071.55	242.79	279.04	485.46	1045.13	2496.93	1336.95	833.05	1996.61	1090.91	18070.17	cGMP-dependent protein kinase 2 (cGK 2) (cGK2) (EC 2.7.11.12) (cGMP-dependent protein kinase II) (cGKII)	PRKG2 PRKGR2	negative regulation of chloride transport [GO:2001226]; positive regulation of chondrocyte differentiation [GO:0032332]; positive regulation of protein localization [GO:1903829]; protein localization to plasma membrane [GO:0072659]; protein phosphorylation [GO:0006468]; signal transduction [GO:0007165]; tetrahydrobiopterin metabolic process [GO:0046146]

1.52	2852.04	3	1.96	2065.27	2.79	0	1640.75	7.53	0	527.35	32.77	0.87	9100.19	58.52	0.68	1639.64	96.01	18030.89	Microtubule-associated protein tau (Neurofibrillary tangle protein) (Paired helical filament-tau) (PHF-tau)	MAPT TAU	microtubule cytoskeleton organization [GO:0000226]; neuron projection development [GO:0031175]
680.51	625.56	730.05	295.61	717.29	881.24	1471.77	270.38	2045.91	273.39	402.54	768.43	827.19	1907.69	1787.02	1048.37	993.48	2284.95	18011.38	Interferon alpha/beta receptor 1a (Membrane-associated type I interferon receptor) (mIFNAR1) (Type I interferon receptor 1a)	ifnar1a	type I interferon-mediated signaling pathway [GO:0060337]
553.08	1149.54	1124.04	526.69	1825.63	1871.79	927.98	764.45	997.59	892.28	501.89	764.46	748.74	1452.74	1221.82	780.8	718.35	1183.03	18004.9	Cytochrome P450 2D6 (EC 1.14.14.-) (CYPIID6) (Cholesterol 25-hydroxylase) (Cytochrome P450-DB1) (Debrisoquine 4-hydroxylase)	CYP2D6 CYP2DL1	alkaloid catabolic process [GO:0009822]; alkaloid metabolic process [GO:0009820]; arachidonate metabolic process [GO:0019369]; cholesterol metabolic process [GO:0008203]; coumarin metabolic process [GO:0009804]; estrogen metabolic process [GO:0008210]; isoquinoline alkaloid metabolic process [GO:0033076]; long-chain fatty acid biosynthetic process [GO:0042759]; monoterpenoid metabolic process [GO:0016098]; negative regulation of organofluorine metabolic process [GO:0090350]; oxidative demethylation [GO:0070989]; retinol metabolic process [GO:0042572]; steroid metabolic process [GO:0008202]; xenobiotic catabolic process [GO:0042178]; xenobiotic metabolic process [GO:0006805]

921.95		1286.09	1587.11	385.48	1209.63	1172.39	67.22	616.93	1500.73	330.47	411.13	1130.05	992.09	1773.71	1604.29	228.94	908.15	1868.43	17994.79	High affinity cationic amino acid transporter 1 (CAT-1) (CAT1) (Ecotropic retroviral leukemia receptor homolog) (Ecotropic retrovirus receptor homolog) (Solute carrier family 7 member 1) (System Y+ basic amino acid transporter)	SLC7A1 ATRC1 ERR REC1L	amino acid import across plasma membrane [GO:0089718]; amino acid transport [GO:0006865]; L-amino acid transport [GO:0015807]; L-arginine import across plasma membrane [GO:0097638]; L-arginine transmembrane transport [GO:1903826]; L-histidine import across plasma membrane [GO:1903810]; L-ornithine transmembrane transport [GO:1903352]; lysine transport [GO:0015819]; ornithine transport [GO:0015822]; positive regulation of T cell proliferation [GO:0042102]; transport across blood-brain barrier [GO:0150104]
711.09		646.69	1988.13	201.72	562.6	947.57	376.87	313.41	2210.1	306.42	234.48	1244.85	1175.57	1066.09	2227.46	472.11	513.82	2761.29	17960.27	Sodium-dependent neutral amino acid transporter SLC6A17 (Sodium-dependent neurotransmitter transporter NTT4) (Solute carrier family 6 member 17)	Slc6a17 Ntt4	alanine transport [GO:0032328]; brain development [GO:0007420]; glycine transport [GO:0015816]; L-leucine transport [GO:0015820]; neurotransmitter transport [GO:0006836]; neutral amino acid transport [GO:0015804]; proline transport [GO:0015824]; protein catabolic process [GO:0030163]; sodium ion transport [GO:0006814]

2291.54		1039.19	1365.83	875.19	839.95	1629.98	1303.41	269.47	685.37	326.96	118.36	429.34	1996.39	787.04	676.72	1244.69	1116.87	957.69	17953.99	Pituitary adenylate cyclase-activating polypeptide type I receptor (PACAP type I receptor) (PACAP-R-1) (PACAP-R1)	Adcyap1r1	adenylate cyclase-activating G protein-coupled receptor signaling pathway [GO:0007189]; cell differentiation [GO:0030154]; cell surface receptor signaling pathway [GO:0007166]; G protein-coupled receptor signaling pathway [GO:0007186]; spermatogenesis [GO:0007283]
171.04		4001.63	5.25	296.01	2277.67	21.27	1.8	1271.84	14.52	130.25	1347.84	19.54	153.99	4193	10.47	247.84	3718.36	58.03	17940.35	WD repeat-containing protein 74	WDR74	blastocyst formation [GO:0001825]; ribosomal large subunit biogenesis [GO:0042273]; RNA metabolic process [GO:0016070]; rRNA processing [GO:0006364]

74.51	0	18.78	237.77	1.87	4.09	11955.43	0.74	4.35	93.46	311.35	3.87	21.65	0	1.85	4925.46	242.24	33.14	17930.56	Fumarate hydratase, mitochondrial (Fumarase) (EC 4.2.1.2)	fh zgc:66253	DNA damage response [GO:0006974]; DNA repair [GO:0006281]; fumarate metabolic process [GO:0006106]; malate metabolic process [GO:0006108]; positive regulation of double-strand break repair via nonhomologous end joining [GO:2001034]; regulation of arginine metabolic process [GO:0000821]; tricarboxylic acid cycle [GO:0006099]; urea cycle [GO:0000050]
1274.94	3061.73	57.75	301.43	1354.33	83.81	5.17	3837.89	12.4	381.55	1102.09	82.37	775.6	3441.29	40.08	68.67	2001.21	39.61	17921.92	Drebrin-like protein	DBNL	adaptive immune response [GO:0002250]; endocytosis [GO:0006897]; neuron projection morphogenesis [GO:0048812]; podosome assembly [GO:0071800]; positive regulation of axon extension [GO:0045773]; positive regulation of dendritic spine morphogenesis [GO:0061003]; postsynaptic actin cytoskeleton organization [GO:0098974]; regulation of actin filament polymerization [GO:0030833]; synapse assembly [GO:0007416]
414.44	345.15	1450.12	143.2	347.8	1013	229.43	120.45	3405.35	140.55	142.51	1734.9	955.44	432.44	3032.49	168.78	210.73	3617.37	17904.15	Vitamin K-dependent protein S	PROS1 PROS	blood coagulation [GO:0007596]; fibrinolysis [GO:0042730]

6.35	6272.76	9.16	1.93	2365.4	8.49	0.64	2065.9	10.93	1.28	1489.68	7.08	6.79	3405.94	12.84	1.68	2209.14	21.9	17897.89	SH2 domain-containing protein 3C (Cas/HEF1-associated signal transducer) (SH2 domain-containing Eph receptor-binding protein 1)	Sh2d3c Chat Shep1	signal transduction [GO:0007165]; small GTPase-mediated signal transduction [GO:0007264]
6737.68	107.43	1499.54	1006.92	89.95	3803.38	14.17	55.46	351.95	1436.09	66.94	428.23	732.85	424.52	238.84	49.1	308.17	517.71	17868.93	Heparan-sulfate 6-O-sulfotransferase 1-B (HS 6-OST-1B) (EC 2.8.2.-)	hs6st1b	
901.99	1446.99	999.37	379.88	2085.4	1925.25	14.79	1125.86	68.18	357.76	616.88	509.85	2323.57	2416.61	1016.13	139.37	1119.39	419.19	17866.46	Interphotoreceptor matrix proteoglycan 2 (Interphotoreceptor matrix proteoglycan of 200 kDa) (IPM 200) (Sialoprotein associated with cones and rods proteoglycan) (Spacrcan)	IMPG2 IPM200	extracellular matrix organization [GO:0030198]; protein localization [GO:0008104]; retina morphogenesis in camera-type eye [GO:0060042]; visual perception [GO:0007601]

632.84	1117.7	393.78	622.73	1254.21	3085.62	46.83	1154.82	765.41	658.19	1034.03	329.31	730.65	2628.71	1418.49	397.88	627.81	950.02	17849.03	Probable ATP-dependent RNA helicase DDX5 (EC 3.6.4.13) (DEAD box protein 5) (RNA helicase p68)	DDX5 G17P1 HELR HLR1	alternative mRNA splicing, via spliceosome [GO:0000380]; androgen receptor signaling pathway [GO:0030521]; BMP signaling pathway [GO:0030509]; epithelial to mesenchymal transition [GO:0001837]; estrogen receptor signaling pathway [GO:0030520]; intrinsic apoptotic signaling pathway by p53 class mediator [GO:0072332]; miRNA transcription [GO:0061614]; mRNA splicing, via spliceosome [GO:0000398]; mRNA transcription [GO:0009299]; myoblast differentiation [GO:0045445]; negative regulation of transcription by RNA polymerase II [GO:0000122]; nuclear-transcribed mRNA catabolic process [GO:0000956]; positive regulation of DNA damage response, signal transduction by p53 class mediator [GO:0043517]; primary miRNA processing [GO:0031053]; regulation of alternative mRNA splicing, via spliceosome [GO:0000381]; regulation of androgen receptor signaling pathway [GO:0060765]; regulation of osteoblast
23.98	0	1366.81	93.24	2.23	72.94	7834.62	4.14	824.71	41.18	175.91	267.29	10.08	4.14	357.45	5806.39	141.1	781.95	17808.16	RILP-like protein 2 (Rab-interacting lysosomal-like protein 2)	rilpl2 si:ch211-275j6.7 zgc:162589	cilium assembly [GO:0060271]; epithelial cell morphogenesis [GO:0003382]; protein transport from ciliary membrane to plasma membrane [GO:1903445]

1015.72	1205.2	1060.35	334.65	1050.03	890.97	364.07	470.99	1788.23	307.88	408.78	1058.35	938.02	1493.9	1829.93	483.24	840.27	2255.99	17796.57	Histone acetyltransferase KAT6A (EC 2.3.1.48) (MOZ, YBF2/SAS3, SAS2 and TIP60 protein 3) (MYST-3) (Monocytic leukemia zinc finger protein) (Runt-related transcription factor-binding protein 2) (Zinc finger protein 220)	KAT6A MOZ MYST3 RUNXBP2 ZNF220	cellular senescence [GO:0090398]; chromosome organization [GO:0051276]; myeloid cell differentiation [GO:0030099]; negative regulation of DNA-templated transcription [GO:0045892]; nucleosome assembly [GO:0006334]; positive regulation of DNA-templated transcription [GO:0045893]; positive regulation of gene expression [GO:0010628]; protein acetylation [GO:0006473]; regulation of developmental process [GO:0050793]; regulation of DNA-templated transcription [GO:0006355]; regulation of hemopoiesis [GO:1903706]; regulation of signal transduction by p53 class mediator [GO:1901796]; regulation of transcription by RNA polymerase II [GO:0006357]
1264.73	1681.95	1677.2	97.62	548.97	465.98	36.52	517.31	2930.88	714.72	588.1	1280.56	141.13	546.95	657.18	265.35	592.81	3760.88	17768.84	Myosin regulatory light chain 2, ventricular/cardiac muscle isoform (MLC-2) (MLC-2v) (Cardiac myosin light chain 2) (Myosin light chain 2, slow skeletal/ventricular muscle isoform) (MLC-2s/v) (Ventricular myosin light chain 2)	MYL2 MLC2	cardiac muscle contraction [GO:0060048]; cardiac myofibril assembly [GO:0055003]; heart contraction [GO:0060047]; heart development [GO:0007507]; muscle cell fate specification [GO:0042694]; negative regulation of cell growth [GO:0030308]; positive regulation of the force of heart contraction [GO:0098735]; post-embryonic development [GO:0009791]; regulation of striated muscle contraction [GO:0006942]; regulation of the force of heart contraction [GO:0002026]; ventricular cardiac muscle tissue morphogenesis [GO:0055010]

2662.62		659.31	973.08	568.75	590.29	1517.12	426.77	309.98	1010.18	897.19	354.59	811.67	2391.02	776.84	1173.87	502.26	684.48	1448.08	17758.1	Laminin subunit alpha-3 (Epiligrin 170 kDa subunit) (E170) (Epiligrin subunit alpha) (Kalinin subunit alpha) (Laminin-5 subunit alpha) (Laminin-6 subunit alpha) (Laminin-7 subunit alpha) (Nicein subunit alpha)	LAMA3 LAMNA	cell-cell adhesion [GO:0098609]; endodermal cell differentiation [GO:0035987]; epidermis development [GO:0008544]; hemidesmosome assembly [GO:0031581]; regulation of cell adhesion [GO:0030155]; regulation of cell migration [GO:0030334]; regulation of embryonic development [GO:0045995]
563.24		1410.22	1071.97	188.45	1224.23	1332.8	1026.9	492.67	1603.41	311.99	438.76	1447.88	716.4	1202.11	1518.06	611.09	785.55	1776.81	17722.54	Intermembrane lipid transfer protein VPS13C (Vacuolar protein sorting-associated protein 13C)	VPS13C KIAA1421	Golgi to endosome transport [GO:0006895]; lipid transport [GO:0006869]; mitochondrion organization [GO:0007005]; negative regulation of type 2 mitophagy [GO:1905090]; protein retention in Golgi apparatus [GO:0045053]; protein targeting to vacuole [GO:0006623]; response to insulin [GO:0032868]

779.41		759.1	1219.42	277.73	888.61	1203.85	1373.78	412.47	1378.57	288.23	307.77	1077.76	849.67	986.08	1409.3	1716.22	660.72	2128.83	17717.52	Nuclear mitotic apparatus protein 1 (Nuclear matrix protein-22) (NMP-22) (Nuclear mitotic apparatus protein) (NuMA protein) (SP-H antigen)	NUMA1 NMP22 NUMA	anastral spindle assembly [GO:0055048]; astral microtubule organization [GO:0030953]; cell division [GO:0051301]; establishment of mitotic spindle orientation [GO:0000132]; meiotic cell cycle [GO:0051321]; microtubule bundle formation [GO:0001578]; nucleus organization [GO:0006997]; positive regulation of BMP signaling pathway [GO:0030513]; positive regulation of chromosome segregation [GO:0051984]; positive regulation of chromosome separation [GO:1905820]; positive regulation of hair follicle development [GO:0051798]; positive regulation of intracellular transport [GO:0032388]; positive regulation of keratinocyte differentiation [GO:0045618]; positive regulation of microtubule polymerization [GO:0031116]; positive regulation of mitotic spindle elongation [GO:1902846]; positive regulation of protein localization to cell cortex [GO:1904778]; positive regulation of protein localization to spindle pole body
2346.59		933.9	2424.17	518.45	768.09	1314.42	34.51	368.85	1243.44	633.28	354.05	1693.19	1785.62	591.19	796.46	227.49	442.17	1233.39	17709.26	Annexin A1 (Annexin I) (Annexin-1) (Calpactin II) (Calpactin-2) (Chromobindin-9) (Lipocortin I) (Phospholipase A2 inhibitory protein) (p35) [Cleaved into: Annexin Ac2-26]	ANXA1 ANX1 LPC1	actin cytoskeleton organization [GO:0030036]; adaptive immune response [GO:0002250]; alpha-beta T cell differentiation [GO:0046632]; arachidonate secretion [GO:0050482]; cell surface receptor signaling pathway [GO:0007166]; cellular response to glucocorticoid stimulus [GO:0071385]; cellular response to vascular endothelial growth factor stimulus [GO:0035924]; G protein-coupled receptor signaling pathway, coupled to cyclic nucleotide second messenger [GO:0007187]; granulocyte chemotaxis [GO:0071621]; inflammatory response [GO:0006954]; innate immune response [GO:0045087]; keratinocyte differentiation [GO:0030216]; monocyte chemotaxis [GO:0002548]; myoblast migration involved in skeletal muscle regeneration [GO:0014839]; negative regulation of apoptotic process [GO:0043066]; negative regulation of exocytosis [GO:0045920]; negative regulation of interleukin-8 production [GO:0032717]; negative regulation of T-
742.79		573.47	1639.54	384.37	916.96	1635.6	6.87	1263.77	711.93	567.31	702.5	1679.73	783.07	1806.7	433.81	119.27	3207.43	499.19	17674.31	Centrosomal protein of 89 kDa (Cep89) (Coiled-coil domain-containing protein 123)	cep89 ccdc123 zgc:63648	chemical synaptic transmission [GO:0007268]; cilium assembly [GO:0060271]; mitochondrion organization [GO:0007005]

22.61	2102.34	80.27	1.78	801.54	76.75	0	4076.29	0	32.59	864.17	470.12	57.07	1331.06	16.68	156.32	7452.01	104.78	17646.38	Doublesex- and mab-3-related transcription factor A2 (Doublesex- and mab-3-related transcription factor 5)	dmrta2 dmrt5	germ cell development [GO:0007281]; sex differentiation [GO:0007548]
904.26	864.93	629.32	357.36	614.5	925.55	3825.99	184.06	386.13	296.94	243.3	390.15	1208.13	1009.09	542.52	4112.91	599.96	546.56	17641.66	Protocadherin gamma-B5 (PCDH-gamma-B5)	PCDHGB5	cell adhesion [GO:0007155]; homophilic cell adhesion via plasma membrane adhesion molecules [GO:0007156]; nervous system development [GO:0007399]
711.41	978.32	1132.94	337.15	1104.12	918.26	3101.84	401.81	784.34	275.16	383.91	651.24	793.44	1551.54	1054.49	1573.61	920.02	956.46	17630.06	Tomoregulin-2 (TR-2) (Transmembrane protein with EGF-like and two follistatin-like domains)	TMEFF2	cell differentiation [GO:0030154]; negative regulation of cell migration [GO:0030336]; negative regulation of integrin biosynthetic process [GO:0045720]; negative regulation of stress fiber assembly [GO:0051497]; wound healing, spreading of cells [GO:0044319]

1108.37	769.75	721.8	456.8	1608.17	974.39	12.08	1247.38	156.77	656.6	902.45	947.09	2600.23	1959.07	1430.31	88.92	1044	932.27	17616.45	DCN1-like protein 2 (DCNL2) (DCUN1 domain-containing protein 2) (Defective in cullin neddylation protein 1-like protein 2)	DCUN1D2 C13orf17 DCUN1L2	positive regulation of protein neddylation [GO:2000436]; protein neddylation [GO:0045116]; regulation of protein neddylation [GO:2000434]
774.85	420.91	1262.8	411.81	599.21	1573.09	490.98	204.53	1299.98	374.65	251.41	1258.81	1341.6	810.93	1385.49	1064.53	1118.57	2949.77	17593.92	Cholesterol 25-hydroxylase-like protein (EC 1.14.99.38)	ch25h zgc:101688	B cell chemotaxis [GO:0035754]; cholesterol metabolic process [GO:0008203]; defense response to virus [GO:0051607]; sterol biosynthetic process [GO:0016126]
63.94	5836.07	181.05	470.77	2423.03	1741.25	5.43	1700.82	6.05	117.62	227.77	665.49	38.32	2540.06	10.37	66.58	1381.8	60.53	17536.95	mRNA-decapping enzyme 1B (EC 3.6.1.62)	DCP1B	deadenylation-dependent decapping of nuclear-transcribed mRNA [GO:0000290]; deadenylation-independent decapping of nuclear-transcribed mRNA [GO:0031087]; nuclear-transcribed mRNA catabolic process, nonsense-mediated decay [GO:0000184]

0	0	578.93	15.59	107.77	5742.61	13.25	28.61	48.3	20.98	7.37	328.31	0	82.39	10339.37	61.96	76.36	0	17451.8	Thrombospondin-3a (Thbs3a)	thbs3a thbs3 tsp3 zgc:103461	cell adhesion [GO:0007155]; mesenchymal to epithelial transition [GO:0060231]; somite development [GO:0061053]
0	123.27	0	9.03	4036.8	14.34	2.45	2436.46	0	10.06	7539.26	0	0	2689.3	0	10.63	484.86	3.45	17359.91	Apelin receptor A (Angiotensin II receptor-like 1a) (Angiotensin receptor-like 1a) (G-protein coupled receptor APJ A)	aplnra agtrl1 agtrl1a	angioblast cell migration from lateral mesoderm to midline [GO:0035479]; blood vessel development [GO:0001568]; cardiac muscle tissue development [GO:0048738]; cell migration [GO:0016477]; cell migration involved in gastrulation [GO:0042074]; coronary vasculature development [GO:0060976]; determination of heart left/right asymmetry [GO:0061371]; determination of left/right symmetry [GO:0007368]; determination of liver left/right asymmetry [GO:0071910]; endodermal cell differentiation [GO:0035987]; epiboly involved in gastrulation with mouth forming second [GO:0055113]; G protein-coupled receptor signaling pathway [GO:0007186]; gastrulation with mouth forming second [GO:0001702]; heart development [GO:0007507]; Kupffer's vesicle development [GO:0070121]; lymph vessel development [GO:0001945]; negative regulation of cAMP-mediated signaling [GO:0043951]; positive regulation of
1721.51	1281.1	514.05	947.94	3244.38	760.55	343.32	420.06	62.06	557.37	990.66	210.47	1618.59	3091.89	161.18	260.68	1120.96	41.45	17348.22	Signal transducer and activator of transcription 4	STAT4	cell surface receptor signaling pathway via JAK-STAT [GO:0007259]; cytokine-mediated signaling pathway [GO:0019221]; defense response [GO:0006952]; interleukin-12-mediated signaling pathway [GO:0035722]; positive regulation of transcription by RNA polymerase II [GO:0045944]; regulation of cell population proliferation [GO:0042127]; regulation of transcription by RNA polymerase II [GO:0006357]; response to interleukin-6 [GO:0070741]; response to peptide hormone [GO:0043434]; T-helper 1 cell differentiation [GO:0045063]

575.25	310.17	1018.71	214.46	254.28	747.45	1629.05	168.12	2772.26	199.02	157.67	1323.54	800.06	705.59	2241.69	1038.95	327.24	2843.31	17326.82	Inositol hexakisphosphate and diphosphoinositol-pentakisphosphate kinase 2 (EC 2.7.4.24) (Diphosphoinositol pentakisphosphate kinase 2) (Histidine acid phosphatase domain-containing protein 1) (InsP6 and PP-IP5 kinase 2) (VIP1 homolog 2) (mmVIP2)	Ppip5k2 Hisppd1 Kiaa0433 Vip2	inositol metabolic process [GO:0006020]; sensory perception of sound [GO:0007605]
1126.22	490.59	1531.03	343.83	434.54	816.59	1525.64	170.44	1827.16	381.3	252.52	1068.65	1183.36	625.69	1751.95	1102.11	423.25	2241.77	17296.64	Exosome complex component RRP42 (Exosome component 7) (Ribosomal RNA-processing protein 42) (p8)	EXOSC7 KIAA0116 RRP42	exonucleolytic trimming to generate mature 3'-end of 5.8S rRNA from tricistronic rRNA transcript (SSU-rRNA, 5.8S rRNA, LSU-rRNA) [GO:0000467]; nuclear mRNA surveillance [GO:0071028]; nuclear polyadenylation-dependent rRNA catabolic process [GO:0071035]; RNA catabolic process [GO:0006401]; RNA processing [GO:0006396]; rRNA catabolic process [GO:0016075]; rRNA processing [GO:0006364]; TRAMP-dependent tRNA surveillance pathway [GO:0071038]; U1 snRNA 3'-end processing [GO:0034473]; U4 snRNA 3'-end processing [GO:0034475]; U5 snRNA 3'-end processing [GO:0034476]
1121.03	2388.9	951.53	283.06	2299.46	872.44	377.93	1165.56	236.72	281.74	1060.63	386.84	418.35	3590.56	162.07	297.85	1188.86	205.66	17289.19	Ankyrin repeat and SOCS box protein 16 (ASB-16)	ASB16	intracellular signal transduction [GO:0035556]; protein ubiquitination [GO:0016567]

952.94	1066.63	871.71	545.71	1297.76	1186.35	1709.94	579.99	786.84	458.18	530.34	1075.86	1002.84	1633.75	710.83	1152.03	910.7	798.96	17271.36	Histone H4 transcription factor (Histone nuclear factor P) (HiNF-P) (MBD2-interacting zinc finger protein) (Methyl-CpG-binding protein 2-interacting zinc finger protein)	Hinfp Mizf	cell cycle G1/S phase transition [GO:0044843]; DNA damage checkpoint signaling [GO:0000077]; DNA repair [GO:0006281]; DNA-templated transcription [GO:0006351]; establishment of protein localization [GO:0045184]; G1/S transition of mitotic cell cycle [GO:0000082]; in utero embryonic development [GO:0001701]; myoblast differentiation [GO:0045445]; negative regulation of DNA-templated transcription [GO:0045892]; negative regulation of gene expression [GO:0010629]; negative regulation of transcription by RNA polymerase II [GO:0000122]; positive regulation of DNA-templated transcription [GO:0045893]; positive regulation of gene expression [GO:0010628]; regulation of DNA-templated transcription [GO:0006355]
1247.4	1320.68	6.16	2626.28	1125.17	16.14	9.07	931.6	21.42	3034.44	3266.41	7.97	596.02	708.21	9.78	720.64	1585.13	23.64	17256.16	Myosin light chain 1, skeletal muscle isoform (LC-1) (LC1) (Myosin light chain alkali 1) (Myosin light chain A1)		
0.78	0.45	1157.52	2.3	0	5.46	1.4	0	4808.59	0	1.5	1813	2.29	1.62	3853.97	2.41	5.94	5598.56	17255.79	Zinc finger CCHC domain-containing protein 8 (TRAMP-like complex RNA-binding factor ZCCHC8)	ZCCHC8	mRNA 3'-end processing [GO:0031124]; RNA splicing [GO:0008380]

76.12	66.41	267.42	407.08	45.7	412.25	8189.23	17.25	32.3	121.5	252.85	89.99	70.59	274.9	129.08	6473.02	285.97	25.19	17236.85	Transmembrane protein 182	Tmem182	muscle organ development [GO:0007517]; myotube cell development involved in skeletal muscle regeneration [GO:0014906]; myotube differentiation involved in skeletal muscle regeneration [GO:0014908]; negative regulation of myoblast differentiation [GO:0045662]; negative regulation of myoblast fusion [GO:1901740]
458.64	1378.54	713.44	229.49	2539.36	1231.32	381.14	1360.55	141.42	268.28	799.23	385.58	1009.39	3116.87	589.05	515	1596.04	523.23	17236.57	Teashirt homolog 1 (Teashirt-like zinc finger protein)	tshz1 sdccag33 tsrt	regulation of transcription by RNA polymerase II [GO:0006357]
5118.57	536.92	705	1059.61	595.18	982.65	847.61	244.93	155.73	883.27	317.66	291.52	2166.07	1293.74	282.99	848.5	732.9	164.92	17227.77	Inositol hexakisphosphate kinase 1 (InsP6 kinase 1) (EC 2.7.4.21) (Inositol hexaphosphate kinase 1)	Ip6k1 Ihpk1	inositol metabolic process [GO:0006020]; inositol phosphate biosynthetic process [GO:0032958]; negative regulation of cold-induced thermogenesis [GO:0120163]; phosphatidylinositol phosphate biosynthetic process [GO:0046854]

1837.35		1005.42	1353.97	442.83	1141.39	1195.18	401.88	480.25	999.95	547.14	428.96	843.42	1346.94	1319.56	1159.26	461.78	934.84	1289.99	17190.11	Protein-lysine N-methyltransferase SMYD4 (EC 2.1.1.-) (SET and MYND domain-containing protein 4)	smyd4	determination of heart left/right asymmetry [GO:0061371]; heart development [GO:0007507]; heart looping [GO:0001947]; heart morphogenesis [GO:0003007]; methylation [GO:0032259]
2339.65		655.56	571.27	817.78	2049.1	1827.14	13.62	843.26	40.24	499.86	331.59	273.51	2909.47	1869.22	765.45	237.6	915.46	94.47	17054.25	Bone morphogenetic protein receptor type-1A (BMP type-1A receptor) (BMPR-1A) (EC 2.7.11.30) (Activin receptor-like kinase 3) (ALK-3) (Bone morphogenetic protein 4 receptor) (CD antigen CD292)	Bmpr1a	angiogenesis [GO:0001525]; anterior/posterior pattern specification [GO:0009952]; atrioventricular node cell development [GO:0060928]; atrioventricular valve development [GO:0003171]; BMP signaling pathway [GO:0030509]; cardiac conduction system development [GO:0003161]; cardiac right ventricle morphogenesis [GO:0003215]; cartilage development [GO:0051216]; cell differentiation [GO:0030154]; cellular response to BMP stimulus [GO:0071773]; cellular response to growth factor stimulus [GO:0071363]; central nervous system neuron differentiation [GO:0021953]; chondrocyte differentiation [GO:0002062]; developmental growth [GO:0048589]; dorsal aorta morphogenesis [GO:0035912]; dorsal/ventral axis specification [GO:0009950]; dorsal/ventral pattern formation [GO:0009953]; ectoderm development [GO:0007398]; embryonic digit morphogenesis [GO:0042733]; [illegible]

560.91	527.06	1926.11	202.44	539.34	1340.27	1723.3	197.23	1394.13	211.93	211.02	1204.79	1044.1	512.38	1566.43	1628.59	537.65	1703.27	17030.95	m7GpppN-mRNA hydrolase (EC 3.6.1.62) (Nucleoside diphosphate-linked moiety X motif 20) (Nudix motif 20) (mRNA-decapping enzyme 2) (hDpc)	DCP2 NUDT20	deadenylation-dependent decapping of nuclear-transcribed mRNA [GO:0000290]; histone mRNA catabolic process [GO:0071044]; mRNA catabolic process [GO:0006402]; negative regulation of telomere maintenance via telomerase [GO:0032211]; nuclear-transcribed mRNA catabolic process, deadenylation-dependent decay [GO:0000288]; nuclear-transcribed mRNA catabolic process, nonsense-mediated decay [GO:0000184]; regulation of mRNA stability [GO:0043488]; regulation of telomerase RNA localization to Cajal body [GO:1904872]
1791.59	355.24	1531.69	537.4	596.8	792.92	780.71	118.17	2116.85	609.89	194.36	973.46	1256.62	305.24	1646.89	834.7	596.05	1975.85	17014.43	Polysialoglycoprotein (PSGP) (Apopolysialoglycoprotein) (apoPSGP)		
401.63	530.87	1923.38	156.07	497.11	1162.87	70.89	237.31	1974.09	227.45	319.96	1777.71	360.09	497.89	2655.35	132.65	270.44	3808.02	17003.78	Zinc finger and BTB domain-containing protein 39	ZBTB39 KIAA0352	negative regulation of transcription by RNA polymerase II [GO:0000122]; regulation of cytokine production [GO:0001817]; regulation of immune system process [GO:0002682]

897.1	843.31	1268.1	540.51	981.32	1397.39	693.57	276.38	911.51	429.41	602.78	1272.13	1207.95	2413.49	817.01	809.48	886.41	754.26	17002.11	CMP-N-acetylneuraminate-beta-galactosamide-alpha-2,3-sialyltransferase 1 (Alpha 2,3-ST 1) (Beta-galactoside alpha-2,3-sialyltransferase 1) (EC 2.4.3.4) (Gal-NAc6S) (Gal-beta-1,3-GalNAc-alpha-2,3-sialyltransferase) (Monosialoganglioside sialyltransferase) (EC 2.4.3.2) (ST3Gal I) (ST3GalI) (ST3GalA.1) (ST3O) (Sialyltransferase 4A) (SIAT4-A)	ST3GAL1 SIAT4A	ganglioside biosynthetic process via lactosylceramide [GO:0010706]; N-acetylneuraminate metabolic process [GO:0006054]; protein glycosylation [GO:0006486]; protein N-linked glycosylation [GO:0006487]; sialylation [GO:0097503]
1117.66	449.13	1383.65	327.86	642.94	1191.55	303.45	333.75	2121.06	398.4	287.14	1262.39	977.82	911.82	1739.12	409.46	630.99	2512.05	17000.24	Neuronal acetylcholine receptor subunit beta-2 (GF-beta-2)	chrnb2	acetylcholine receptor signaling pathway [GO:0095500]; behavioral response to nicotine [GO:0035095]; cognition [GO:0050890]; memory [GO:0007613]; nervous system process [GO:0050877]; response to hypoxia [GO:0001666]; signal transduction [GO:0007165]; synaptic transmission, cholinergic [GO:0007271]; visual learning [GO:0008542]
626.54	850.79	1102.21	228.85	828.5	761.76	3610.77	388.22	1253	179.35	390.72	1200.57	569.45	1209.61	816.09	1827.79	457.36	663.81	16965.39	Small integral membrane protein 7	smim7	
1014.04	294.64	813.62	444.45	380.45	1281.75	3645.59	210.83	519.59	656.29	284.37	799.65	907.65	479.01	923.06	3015.78	603.18	682.45	16956.4	Butyrophilin subfamily 1 member A1 (BT)	BTN1A1 BTN	regulation of cytokine production [GO:0001817]; T cell receptor signaling pathway [GO:0050852]

8.99	4817.37	0	20.8	3042.57	19.83	0	2064.82	1.18	32.32	1213.41	0	3.49	4203.89	0	11.62	1494.88	2.87	16938.04	Guanine nucleotide exchange factor DBS (DBL's big sister) (MCF2-transforming sequence-like protein) (OST oncogene)	Mcf2l Ost	intracellular signal transduction [GO:0035556]; positive regulation of transcription by RNA polymerase II [GO:0045944]; Rho protein signal transduction [GO:0007266]
5708.67	469.89	1833.26	1041.27	483.74	348.84	21.75	266.13	753.06	1284.11	245.4	658.33	751.96	238.7	335.76	96.63	512.21	1887.72	16937.43	Nuclear protein MDM1	mdm1 si:ch211-266a5.6	negative regulation of centriole replication [GO:0046600]
0	0	542.39	2.21	0	70.28	2.24	0	1945.17	0	0	673.3	0	0	5675.89	0.96	2.49	8010.94	16925.87	Sialin (H(+)/nitrate cotransporter) (H(+)/sialic acid cotransporter) (AST) (Solute carrier family 17 (Anion/sugar transporter), member 5) (Vesicular excitatory amino acid transporter) (VEAT)	Slc17a5	neurotransmitter loading into synaptic vesicle [GO:0098700]; response to bacterium [GO:0009617]; sialic acid transport [GO:0015739]
2073.2	1060.97	2098.92	606.64	1028.2	1174.9	773.56	327.76	833.19	460.01	289.14	828	990.45	1039.44	972.83	709.62	528.13	1113.67	16908.63	Transcription elongation factor A protein 3 (Transcription elongation factor S-II protein 3) (Transcription elongation factor TFIIS.h)	TCEA3 TFIISH	regulation of transcription by RNA polymerase II [GO:0006357]; transcription elongation by RNA polymerase II [GO:0006368]

363.31	254.62	951.5	289.19	241.73	500.18	5425.78	134.34	398.53	196.25	284.3	477.45	401.2	359.61	555.62	5167.21	327.67	574.77	16903.26	Bridging integrator 3	Bin3	actin cortical patch localization [GO:0051666]; cell division [GO:0051301]; endocytosis [GO:0006897]; myoblast migration involved in skeletal muscle regeneration [GO:0014839]; plasma membrane tubulation [GO:0097320]; protein localization [GO:0008104]; regulation of lamellipodium assembly [GO:0010591]; skeletal muscle fiber development [GO:0048741]; skeletal muscle tissue regeneration [GO:0043403]; unidimensional cell growth [GO:0009826]
165.43	1075.04	480.09	149.52	3458.44	164.34	16.66	505.86	237.54	95.27	415.8	240.75	260.61	5194.45	152.48	296.33	3735.88	255.6	16900.09	Rhodopsin kinase GRK1 (RK) (EC 2.7.11.14) (G protein-coupled receptor kinase 1)	GRK1 RHOK	G protein-coupled opsin signaling pathway [GO:0016056]; protein autophosphorylation [GO:0046777]; regulation of G protein-coupled receptor signaling pathway [GO:0008277]; regulation of opsin-mediated signaling pathway [GO:0022400]; regulation of signal transduction [GO:0009966]; visual perception [GO:0007601]
825.41	938.82	1526.42	402.89	855.06	898.22	37.92	465.06	1601.64	636.25	426.28	883	981.74	1727.6	1759.76	269.4	750.9	1886.88	16873.25	Cubilin (Intrinsic factor-cobalamin receptor)	CUBN IFCR	cholesterol metabolic process [GO:0008203]; protein transport [GO:0015031]

498.12	7.12	0.65	2155.65	0	38.6	4550.98	0	0	142.69	153.9	1.96	0	0	0	9025.17	223.92	14.51	16813.27	Contactin-associated protein-like 4 (Cell recognition molecule Caspr4)	CNTNAP4 CASPR4 KIAA1763	cell adhesion [GO:0007155]; regulation of grooming behavior [GO:2000821]; regulation of synaptic transmission, dopaminergic [GO:0032225]; regulation of synaptic transmission, GABAergic [GO:0032228]
18.97	32.34	624.09	22.6	83.71	193.46	33.56	18.98	4953.97	15.72	41.28	1407.98	9.13	164.1	4247	78.31	117.7	4698.94	16761.84	Reelin (EC 3.4.21.-) (Reeler protein)	Reln Rl	associative learning [GO:0008306]; axon guidance [GO:0007411]; brain development [GO:0007420]; cell adhesion [GO:0007155]; cell migration [GO:0016477]; cell morphogenesis [GO:0000902]; central nervous system development [GO:0007417]; cerebral cortex development [GO:0021987]; cerebral cortex tangential migration [GO:0021800]; dendrite development [GO:0016358]; forebrain development [GO:0030900]; glial cell differentiation [GO:0010001]; hippocampus development [GO:0021766]; interneuron migration [GO:1904936]; lateral motor column neuron migration [GO:0097477]; layer formation in cerebral cortex [GO:0021819]; learning [GO:0007612]; locomotory behavior [GO:0007626]; long-term memory [GO:0007616]; long-term synaptic potentiation [GO:0060291]; modulation of chemical synaptic transmission [GO:0050804]; motor neuron migration [GO:0097475]; neuron migration [GO:0001764]; NMDA glutamate

585.37	815.33	205.14	264.35	877.7	139.49	6321.86	340.69	151.98	182.49	407.91	139.06	551.88	1088.62	187.46	3707.97	578.41	188.58	16734.29	LETM1 domain-containing protein LETM2, mitochondrial (LETM1 and EF-hand domain-containing protein 2) (Leucine zipper-EF-hand-containing transmembrane protein 1-like)	letm2 TEgg018g03.1	
0	0	631.24	0	0	8750.21	8.24	0	0	12.8	6.23	0	0	0	7290.7	8.66	9.97	0	16718.05	Cyclic AMP-responsive element-binding protein 3-like protein 3-B (cAMP-responsive element-binding protein 3-like protein 3-B) [Cleaved into: Processed cyclic AMP-responsive element-binding protein 3-like protein 3-B]	creb3l3b creb3l3 si:dkey-110c1.2	regulation of transcription by RNA polymerase II [GO:0006357]

48.16	24.62	1.33	37.97	31.72	0.97	21.19	26.25	1.24	3.05	321.37	11.5	0	7772.41	0	1437.58	6897.77	72.72	16709.85	Nesprin-2 (KASH domain-containing protein 2) (KASH2) (Nuclear envelope spectrin repeat protein 2) (Nucleus and actin connecting element protein) (Protein NUANCE) (Synaptic nuclear envelope protein 2) (Syne-2)	SYNE2 KIAA1011 NUA	centrosome localization [GO:0051642]; nuclear migration [GO:0007097]; nuclear migration along microfilament [GO:0031022]; nucleokinesis involved in cell motility in cerebral cortex radial glia guided migration [GO:0021817]; positive regulation of cell migration [GO:0030335]; regulation of cilium assembly [GO:1902017]
612.42	887.29	1079.5	2652.33	1716.94	1212.78	88.56	1197.71	511.43	508.4	783.79	215.22	909.44	698.74	1192.78	541.69	1303.91	545.91	16658.84	Inactive serine protease PAMR1 (Peptidase domain-containing protein associated with muscle regeneration 1) (Regeneration-associated muscle protease homolog)	PAMR1 RAMP	
1526.27	126.11	1856.24	178.43	786.16	3095.8	13.28	78.59	164.99	649.88	385.18	2513.57	1173.38	2333.08	343.4	199.12	967.77	235.17	16626.42	Kinesin heavy chain isoform 5A (EC 5.6.1.3) (Kinesin heavy chain neuron-specific 1) (Neuronal kinesin heavy chain) (NKHC)	Kif5a Kiaa4086 Kif5 Nkhc1	anterograde axonal protein transport [GO:0099641]; anterograde dendritic transport of neurotransmitter receptor complex [GO:0098971]; vesicle-mediated transport [GO:0016192]

786.55	553.34	3248.86	262.52	347.01	4427.44	326.47	179.52	186.2	330.9	239.59	1492.22	1491.49	377.25	1080.31	403.02	425.2	467.6	16625.49	Interferon regulatory factor 4 (IRF-4) (Lymphocyte-specific interferon regulatory factor) (LSIRF) (NF-EM5) (PU.1 interaction partner) (Transcriptional activator PIP)	Irf4 Spip	chromatin remodeling [GO:0006338]; defense response to protozoan [GO:0042832]; myeloid dendritic cell differentiation [GO:0043011]; negative regulation of toll-like receptor signaling pathway [GO:0034122]; positive regulation of cold-induced thermogenesis [GO:0120162]; positive regulation of DNA-templated transcription [GO:0045893]; positive regulation of interleukin-10 production [GO:0032733]; positive regulation of interleukin-13 production [GO:0032736]; positive regulation of interleukin-2 production [GO:0032743]; positive regulation of interleukin-4 production [GO:0032753]; positive regulation of transcription by RNA polymerase II [GO:0045944]; T-helper 17 cell lineage commitment [GO:0072540]
26.29	0.69	263.88	113.23	1.34	498.38	9126.62	1.49	24.37	49.92	188.3	305.36	8.51	0.84	126.96	5675.3	179.12	19.44	16610.04	Malate dehydrogenase, cytoplasmic (EC 1.1.1.37) (Cytosolic malate dehydrogenase)	MDH1 RCJMB04_2g5	gluconeogenesis [GO:0006094]; malate metabolic process [GO:0006108]; tricarboxylic acid cycle [GO:0006099]
684.06	737.89	1676.84	336.06	859.43	1621.28	1263.82	318.51	854.97	322.96	408.77	1789.88	739.7	1498.47	816.66	1280.2	788.24	589.84	16587.58	LanC-like protein 2 (Testis-specific adriamycin sensitivity protein)	LANCL2 GPR69B TASP	carbohydrate metabolic process [GO:0005975]; negative regulation of DNA-templated transcription [GO:0045892]; peptide modification [GO:0031179]; positive regulation of abscisic acid-activated signaling pathway [GO:0009789]

891.95	851.5	1689.18	139.4	1042.53	172.49	153.97	746.29	2176.81	71.66	449.28	625.43	313.78	3389.03	1522.16	94.88	779.98	1458.07	16568.39	Prosaposin receptor GPR37 (Endothelin B receptor-like protein 1) (ETBR-LP-1) (G-protein coupled receptor 37) (Parkin-associated endothelin receptor-like receptor) (PAELR)	GPR37	adenylate cyclase-inhibiting G protein-coupled receptor signaling pathway [GO:0007193]; cellular response to reactive oxygen species [GO:0034614]; dendrite development [GO:0016358]; dopamine biosynthetic process [GO:0042416]; G protein-coupled receptor signaling pathway [GO:0007186]; locomotion involved in locomotory behavior [GO:0031987]; neuropeptide signaling pathway [GO:0007218]; positive regulation of dopamine metabolic process [GO:0045964]; positive regulation of MAPK cascade [GO:0043410]
0	1.63	651.44	20.23	0	44.74	24.26	0.67	4934.81	0	18.04	3316.27	0	0	2264.58	0	16.45	5260.8	16553.92	Pseudouridylate synthase TRUB1 (EC 5.4.99.-) (TruB pseudouridine synthase homolog 1) (tRNA pseudouridine 55 synthase TRUB1) (Psi55 synthase TRUB1) (EC 5.4.99.25)	TRUB1 PUS4	mRNA processing [GO:0006397]; mRNA pseudouridine synthesis [GO:1990481]; positive regulation of pre-miRNA processing [GO:2000633]; tRNA modification [GO:0006400]

172.22	276.99	2463.49	300.92	142.38	3297.87	21.08	82.32	22.86	316.14	290.96	8107.02	135.72	180.15	46.38	193.12	291.56	207.91	16549.09	Sterol regulatory element-binding protein 2 (SREBP-2) (Sterol regulatory element-binding transcription factor 2) [Cleaved into: Processed sterol regulatory element-binding protein 2 (Transcription factor SREBF2)]	srebf2 SREBP2 zgc:158371	cholesterol metabolic process [GO:0008203]; liver development [GO:0001889]; positive regulation of cholesterol storage [GO:0010886]; positive regulation of transcription by RNA polymerase II [GO:0045944]; regulation of Notch signaling pathway [GO:0008593]
65.37	4730.53	62.41	17.72	5433.15	40.77	0	3749.26	51.23	6.33	206.12	32.15	52.59	900.75	71.08	5.05	1008.21	92.78	16525.5	Ryanodine receptor 2 (RYR-2) (RyR2) (Cardiac muscle ryanodine receptor) (Cardiac muscle ryanodine receptor-calcium release channel) (Cardiac muscle-type ryanodine receptor) (Type 2 ryanodine receptor)	RYR2	calcium ion transport [GO:0006816]; calcium-mediated signaling [GO:0019722]; cellular response to caffeine [GO:0071313]; embryonic heart tube morphogenesis [GO:0003143]; intracellular calcium ion homeostasis [GO:0006874]; release of sequestered calcium ion into cytosol by sarcoplasmic reticulum [GO:0014808]; striated muscle contraction [GO:0006941]
1237.36	1129.37	342.03	862.24	2562.25	1493.44	56.39	906.51	157.06	605.76	444.16	492.94	936.92	2331.28	394.53	157.75	2083.49	321.88	16515.36	Anosmin-1 (Adhesion molecule-like X-linked) (Kallmann syndrome protein)	ANOS1 ADMLX KAL KAL1 KALIG1	axon guidance [GO:0007411]; cell adhesion [GO:0007155]; chemotaxis [GO:0006935]; neuron differentiation [GO:0030182]

240.41	274.32	2085.48	98.94	283.64	968.77	590.71	157	2609.5	102.96	178.26	1436.05	228.84	336.11	2245.06	546.63	307.7	3814.35	16504.73	Ubiquitin carboxyl-terminal hydrolase MINDY-1 (EC 3.4.19.12) (Deubiquitinating enzyme MINDY-1) (Protein FAM63A)	mindy1 fam63a si:ch211-210h11.5	proteolysis [GO:0006508]
885.39	76.54	1702.22	271.26	128.54	5285.04	10.93	76.88	1031.58	228.23	59.36	593.08	518.63	382.9	3937.29	34.53	144.83	1137.47	16504.7	Microtubule-actin cross-linking factor 1, isoforms 6/7 (Uncharacterized protein KIAA0754)	MACF1 KIAA0754	
1074.29	1029.34	704.85	355.83	623.3	908.58	3218.44	437.6	743.09	327.3	302.82	474.27	1317.68	1020.02	654.32	1752.59	765.43	792.81	16502.56	Cytochrome c oxidase assembly protein COX18, mitochondrial	Cox18 Oxa1l2	mitochondrial cytochrome c oxidase assembly [GO:0033617]; protein insertion into mitochondrial inner membrane from matrix [GO:0032979]; protein insertion into mitochondrial membrane [GO:0051204]; respiratory chain complex IV assembly [GO:0008535]

157.34	59.12	267.08	141.13	34.28	477.82	7261.8	20.19	25.32	180.59	312.42	322.04	182.04	31.78	120.05	6615.83	190.78	96.51	16496.12	Glycerophosphodiester phosphodiesterase 1 (Glycerophosphoinositol glycerophosphodiesterase GDE1) (EC 3.1.4.44) (Lysophospholipase D GDE1) (EC 3.1.4.-) (Membrane-interacting protein of RGS16)	Gde1 Mir16	ethanolamine metabolic process [GO:0006580]; G protein-coupled receptor signaling pathway [GO:0007186]; N-acylethanolamine metabolic process [GO:0070291]; phospholipid metabolic process [GO:0006644]
775.94	1522.77	56.44	2764.22	2045.15	43.9	142.51	1222.27	57.4	1116.95	1661.92	90.24	879.28	1392.24	57.09	1032.92	1517.62	110.05	16488.91	ADP-ribosylation factor-like protein 5A	ARL5A	intracellular protein transport [GO:0006886]; protein localization to Golgi membrane [GO:1903292]; vesicle-mediated transport [GO:0016192]
146.38	521.18	1126.6	134.97	540.6	537.33	45.73	189.67	2669.44	90.85	184.67	1506.91	131.54	642.31	3625.05	92.85	531.97	3744.46	16462.51	X-linked retinitis pigmentosa GTPase regulator (mRpgr)	Rpgr	cellular response to light stimulus [GO:0071482]; eye photoreceptor cell development [GO:0042462]; intraciliary transport [GO:0042073]; positive regulation of autophagy [GO:0010508]; protein localization to non-motile cilium [GO:0097499]; retina morphogenesis in camera-type eye [GO:0060042]; visual perception [GO:0007601]

84.9	5757.17	1.01	113.54	2928.36	9.43	0	1038.68	0	17.77	477.64	0	35.7	2900	0	26.81	3050.06	7.15	16448.22	WD repeat-containing protein 47 (Neuronal enriched MAP-interacting protein) (Nemitin)	WDR47 KIAA0893	adult locomotory behavior [GO:0008344]; anterior commissure morphogenesis [GO:0021960]; autophagy [GO:0006914]; cerebral cortex radial glia-guided migration [GO:0021801]; corpus callosum development [GO:0022038]; detection of hot stimulus involved in thermoception [GO:0120168]; microtubule cytoskeleton organization [GO:0000226]; motor behavior [GO:0061744]; negative regulation of microtubule depolymerization [GO:0007026]; neural precursor cell proliferation [GO:0061351]; neuronal stem cell population maintenance [GO:0097150]
11.94	9.8	227.43	3.16	7.59	20.1	1.26	3.36	5542.81	4.51	6.39	1279.13	15.02	19.28	3550.31	5.89	19.38	5708.43	16435.79	A-type potassium channel modulatory protein DPP6 (DPPX) (Dipeptidyl aminopeptidase-like protein 6) (Dipeptidyl aminopeptidase-related protein) (Dipeptidyl peptidase 6) (Dipeptidyl peptidase IV-like protein) (Dipeptidyl peptidase VI) (DPP VI)	Dpp6 Dpp-6	establishment of localization in cell [GO:0051649]; neuronal action potential [GO:0019228]; positive regulation of potassium ion transmembrane transport [GO:1901381]; potassium ion transmembrane transport [GO:0071805]; protein localization to plasma membrane [GO:0072659]; proteolysis [GO:0006508]; regulation of membrane potential [GO:0042391]; regulation of postsynaptic membrane potential [GO:0060078]; regulation of potassium ion transmembrane transport [GO:1901379]; regulation of potassium ion transport [GO:0043266]

941.75	480.36	987.47	358.01	449.44	1080.12	3055.99	257	809.1	399.06	246.93	662.22	1538.63	662.93	1048.16	1830.54	561.91	1046.41	16416.03	Microtubule-associated protein RP/EB family member 1 (APC-binding protein EB1) (End-binding protein 1) (EB1)	Mapre1	attachment of mitotic spindle microtubules to kinetochore [GO:0051315]; cell division [GO:0051301]; cell migration [GO:0016477]; establishment of mitotic spindle orientation [GO:0000132]; microtubule bundle formation [GO:0001578]; microtubule polymerization [GO:0046785]; negative regulation of microtubule polymerization [GO:0031115]; non-motile cilium assembly [GO:1905515]; positive regulation of microtubule polymerization [GO:0031116]; protein localization to astral microtubule [GO:1902888]; protein localization to centrosome [GO:0071539]; protein localization to microtubule [GO:0035372]; protein localization to mitotic spindle [GO:1902480]; regulation of microtubule polymerization or depolymerization [GO:0031110]; spindle assembly [GO:0051225]
4241.36	665.69	555.62	1079.12	1642.93	652.45	26.46	559.27	66.03	1563.96	507.19	612.26	1464.45	709.91	258.49	434.04	1212.13	160.79	16412.15	ADP/ATP translocase 2 (ADP,ATP carrier protein 2) (Adenine nucleotide translocator 2) (ANT 2) (Solute carrier family 25 member 5) [Cleaved into: ADP/ATP translocase 2, N-terminally processed]	Slc25a5 Ant2	adaptive thermogenesis [GO:1990845]; adenine nucleotide transport [GO:0051503]; B cell differentiation [GO:0030183]; cellular response to leukemia inhibitory factor [GO:1990830]; chromosome segregation [GO:0007059]; erythrocyte differentiation [GO:0030218]; mitochondrial ADP transmembrane transport [GO:0140021]; mitochondrial ATP transmembrane transport [GO:1990544]; negative regulation of mitochondrial outer membrane permeabilization involved in apoptotic signaling pathway [GO:1901029]; positive regulation of cell population proliferation [GO:0008284]; positive regulation of mitophagy [GO:1901526]; regulation of mitochondrial membrane permeability [GO:0046902]
0.46	0.48	566.67	2.31	0.46	3.49	32.02	0.52	5277.4	1.01	2.23	1659.87	0	2.64	3836.35	27.04	3.81	4979.36	16396.12	Large ribosomal subunit protein uL2m (39S ribosomal protein L2, mitochondrial) (L2mt) (MRP-L2)	MRPL2 CGI-22	mitochondrial translation [GO:0032543]

1328.07	1038.18	1616.39	126.26	288.44	104.61	1145.3	537.29	553.03	818.4	517.19	2168.85	709.76	2713.67	373.48	955.54	895.74	433.93	16324.13	Matrix metalloproteinase-24 (MMP-24) (EC 3.4.24.-) (Membrane-type matrix metalloproteinase 5) (MT-MMP 5) (MTMMP5) (Membrane-type-5 matrix metalloproteinase) (MT5-MMP) (MT5MMP) [Cleaved into: Processed matrix metalloproteinase-24]	Mmp24 Mt5mmp	cell-cell adhesion mediated by cadherin [GO:0044331]; cell-cell adhesion via plasma-membrane adhesion molecules [GO:0098742]; collagen catabolic process [GO:0030574]; detection of temperature stimulus involved in sensory perception of pain [GO:0050965]; extracellular matrix organization [GO:0030198]; glial cell differentiation [GO:0010001]; neuronal stem cell population maintenance [GO:0097150]; proteolysis [GO:0006508]
885.56	839.51	682.64	533.51	895.72	947.6	2828.42	378.95	549.23	410.88	503.52	873.92	993.42	1037.16	553.13	2151.53	762.5	474.7	16301.9	Histone-lysine N-methyltransferase KMT5C (Lysine N-methyltransferase 5C) (Lysine-specific methyltransferase 5C) (Suppressor of variegation 4-20 homolog 2) (Su(var)4-20 homolog 2) (Suv4-20h2) ([histone H4]-N-methyl-L-lysine20 N-methyltransferase KMT5B) (EC 2.1.1.362) ([histone H4]-lysine20 N-methyltransferase KMT5B) (EC 2.1.1.361)	KMT5C SUV420H2 PP7130	DNA repair [GO:0006281]; methylation [GO:0032259]; positive regulation of double-strand break repair via nonhomologous end joining [GO:2001034]; positive regulation of isotype switching [GO:0045830]

4697.76	3280.32	2016.59	129.77	345.2	563.43	11.44	139.73	37.22	1155.76	993.18	1318.41	507.59	560.09	396.63	56.64	72.23	9.06	16291.05	Endoplasmic reticulum-Golgi intermediate compartment protein 1 (ER-Golgi intermediate compartment 32 kDa protein) (ERGIC-32)	ergic1 ergic32 zgc:114085	endoplasmic reticulum to Golgi vesicle-mediated transport [GO:0006888]; retrograde vesicle-mediated transport, Golgi to endoplasmic reticulum [GO:0006890]
546.38	606.87	821.56	332.2	650.73	792.85	2386.68	281.9	1160.17	203.72	252.87	1665.46	859.51	965.77	1041.48	1691.01	670.7	1353.56	16283.42	E3 ubiquitin-protein ligase ZNRF1 (EC 2.3.2.27) (RING-type E3 ubiquitin transferase ZNRF1) (Zinc/RING finger protein 1)	znrf1	proteasome-mediated ubiquitin-dependent protein catabolic process [GO:0043161]; protein K48-linked ubiquitination [GO:0070936]
0	0	0	7.02	2.3	0	1.97	3.6	14.17	0	14.57	4.65	0	14030.55	53.41	2.02	2113.59	23.61	16271.46	Mitochondrial glutamate carrier 1 (GC-1) (Glutamate/H(+) symporter 1) (Solute carrier family 25 member 22)	SLC25A22 GC1	aspartate transmembrane transport [GO:0015810]; L-glutamate transmembrane transport [GO:0015813]; malate-aspartate shuttle [GO:0043490]; regulation of insulin secretion [GO:0050796]

472.45	1183.51	1106.94	275.9	2285.01	1192.93	203.37	903.22	517.15	241.37	524.78	713.49	1063.92	2356.73	937.68	302.31	991.27	995.35	16267.38	Juxtaposed with another zinc finger protein 1 (TAK1-interacting protein 27) (Zinc finger protein 802)	JAZF1 TIP27 ZNF802	lipid metabolic process [GO:0006629]; negative regulation of transcription by RNA polymerase II [GO:0000122]
27.72	0	6030.03	27.21	0	1408.88	0	2.19	27.31	14.69	3.51	8627.06	0	0	68.13	12.14	2.62	5.16	16256.65	5-hydroxytryptamine receptor 2A (5-HT-2) (5-HT-2A) (Serotonin receptor 2A)	HTR2A HTR2	artery smooth muscle contraction [GO:0014824]; behavioral response to cocaine [GO:0048148]; chemical synaptic transmission [GO:0007268]; detection of mechanical stimulus involved in sensory perception of pain [GO:0050966]; detection of temperature stimulus involved in sensory perception of pain [GO:0050965]; G protein-coupled receptor signaling pathway, coupled to cyclic nucleotide second messenger [GO:0007187]; G protein-coupled serotonin receptor signaling pathway [GO:0098664]; glycolytic process [GO:0006096]; intracellular calcium ion homeostasis [GO:0006874]; memory [GO:0007613]; negative regulation of potassium ion transport [GO:0043267]; negative regulation of synaptic transmission, glutamatergic [GO:0051967]; phospholipase C-activating serotonin receptor signaling pathway [GO:0007208]; positive regulation of cell population proliferation [GO:0008284]; positive
1733.59	1099.27	935.95	1143.97	1058.14	1807.51	407.43	997.35	1218.31	1224.12	654.87	541.85	606.3	689.69	399.35	514.58	711.17	508.39	16251.84	Bifunctional glutamate/proline--tRNA ligase (Bifunctional aminoacyl-tRNA synthetase) (Glutamyl-prolyl-tRNA synthetase) [Includes: Glutamate--tRNA ligase (EC 6.1.1.17) (Glutamyl-tRNA synthetase) (GluRS);Proline--tRNA ligase (EC 6.1.1.15) (Prolyl-tRNA synthetase) (ProRS)]	EPRS1 EPRS QPRS	cellular response to insulin stimulus [GO:0032869]; cellular response to type II interferon [GO:0071346]; glutamyl-tRNA aminoacylation [GO:0006424]; negative regulation of translation [GO:0017148]; prolyl-tRNA aminoacylation [GO:0006433]; regulation of long-chain fatty acid import into cell [GO:0140212]

2.09	0.88	2305.76	0.78	0.43	492.49	67.55	0	48.65	4.48	0.85	13121.68	0.97	0.58	98.26	53.04	2.49	49.83	16250.81	Carnitine O-palmitoyltransferase 1, liver isoform (CPT1-L) (EC 2.3.1.21) (Carnitine O-palmitoyltransferase I, liver isoform) (CPT I) (CPTI-L) (Carnitine palmitoyltransferase 1A) (Succinyltransferase CPT1A) (EC 2.3.1.-)	CPT1A CPT1	aflatoxin metabolic process [GO:0046222]; carnitine metabolic process [GO:0009437]; carnitine shuttle [GO:0006853]; cellular response to fatty acid [GO:0071398]; eating behavior [GO:0042755]; epithelial cell differentiation [GO:0030855]; fatty acid beta-oxidation [GO:0006635]; fatty acid metabolic process [GO:0006631]; glucose metabolic process [GO:0006006]; liver regeneration [GO:0097421]; long-chain fatty acid metabolic process [GO:0001676]; positive regulation of fatty acid beta-oxidation [GO:0032000]; positive regulation of innate immune response [GO:0045089]; regulation of insulin secretion [GO:0050796]; regulation of lipid storage [GO:0010883]; response to alkaloid [GO:0043279]; response to ethanol [GO:0045471]; response to hypoxia [GO:0001666]; response to nutrient [GO:0007584]; response to tetrachloromethane [GO:1904772]; response to xenobiotic stimulus [GO:0009410]; triglyceride metabolic [illegible]
1842.85	522.68	1590.23	483.96	1185.2	2550.83	7.12	367.77	236.3	516.49	928.16	1142.47	589.02	1393.53	433.18	159.09	1670.48	628.07	16247.43	Sodium/hydrogen exchanger 7 (Na(+)/H(+) exchanger 7) (NHE-7) (Solute carrier family 9 member 7)	SLC9A7 NHE7	monoatomic ion transport [GO:0006811]; potassium ion transmembrane transport [GO:0071805]; regulation of Golgi lumen acidification [GO:1905526]; regulation of intracellular pH [GO:0051453]; regulation of pH [GO:0006885]; sodium ion import across plasma membrane [GO:0098719]
423.72	29.18	553.16	123.48	64.61	539.9	653.51	86.58	164.13	329.55	70.3	230.22	1484.8	1667.34	3614.85	1431.96	1601.8	3097.92	16167.01	Tetratricopeptide repeat protein 24 (TPR repeat protein 24)	TTC24	

615.64	343.14	1420.5	241.36	557.27	853.45	1062.01	211.81	2259.38	323.31	219.07	1172.17	608.88	686.28	2332.43	578.02	306.7	2350.09	16141.51	Myocardin	Myocd Mycd	cardiac muscle cell apoptotic process [GO:0010659]; cardiac muscle cell differentiation [GO:0055007]; cardiac muscle cell myoblast differentiation [GO:0060379]; cardiac vascular smooth muscle cell differentiation [GO:0060947]; cardiac ventricle development [GO:0003231]; cell growth involved in cardiac muscle cell development [GO:0061049]; cellular component maintenance [GO:0043954]; cellular response to angiotensin [GO:1904385]; cellular response to growth factor stimulus [GO:0071363]; cellular response to hypoxia [GO:0071456]; cellular response to transforming growth factor beta stimulus [GO:0071560]; chromatin organization [GO:0006325]; digestive tract development [GO:0048565]; ductus arteriosus closure [GO:0097070]; heart development [GO:0007507]; hepatic stellate cell activation [GO:0035733]; lung alveolus development [GO:0048286]; negative regulation of amyloid-beta clearance
952.24	319.62	826.04	142.69	200.16	501.8	265.45	70.66	2835.43	171.73	67.63	1297.27	1502.52	316.62	2765.37	282.17	149.72	3469.27	16136.39	Microtubule-associated protein 4 (MAP-4)	MAP4	cell division [GO:0051301]; cilium disassembly [GO:0061523]; establishment of spindle orientation [GO:0051294]; microtubule cytoskeleton organization [GO:0000226]; microtubule polymerization [GO:0046785]; microtubule sliding [GO:0051012]; mitotic spindle organization [GO:0007052]; negative regulation of non-motile cilium assembly [GO:1902856]; neuron projection development [GO:0031175]
587.35	1219.97	1384.96	336.77	1070.85	971.77	1335.03	390.15	740.96	171.12	349.19	1666.7	711.82	1697.28	880.86	887.55	838.31	893.48	16134.12	RNA-binding motif, single-stranded-interacting protein 2 (Suppressor of CDC2 with RNA-binding motif 3)	RBMS2 SCR3	RNA processing [GO:0006396]

359.61	498.34	1204.71	346.48	350.49	521.07	1911.97	60.14	2213.08	152.97	179.31	1985.05	693.15	873.22	1049.25	1620.07	481.57	1630.27	16130.75	rRNA N(6)-adenosine-methyltransferase METTL5 (EC 2.1.1.-) (Methyltransferase-like protein 5)	mettl5	positive regulation of translation [GO:0045727]; rRNA methylation [GO:0031167]; stem cell differentiation [GO:0048863]
248.41	644.01	3335.37	203.24	302.27	3664.19	10.66	143.23	175.86	473.58	518.5	4105.03	137.67	368.17	556.97	498.75	362.65	381.81	16130.37	Sulfotransferase 1C1 (ST1C1) (EC 2.8.2.-) (Phenol sulfotransferase)	Sult1c1 Sult1a2	3'-phosphoadenosine 5'-phosphosulfate metabolic process [GO:0050427]; sulfur compound metabolic process [GO:0006790]; xenobiotic metabolic process [GO:0006805]
111.56	87.84	890.59	31.8	33.95	105.57	16.65	18.91	4337.82	29.93	26.18	1361.8	139.13	95.97	4121.42	32.62	51.24	4631.58	16124.56	Zinc finger FYVE domain-containing protein 16 (Endofin) (Endosomal-associated FYVE domain protein)	Zfyve16 Kiaa0305	

445.1	474.37	641.82	238.73	539.47	734.79	3777.64	249.26	1475.42	284.16	350.89	754.81	477.69	638.32	924.96	2263.18	440.58	1400.95	16112.14	26S proteasome non-ATPase regulatory subunit 3 (26S proteasome regulatory subunit RPN3) (26S proteasome regulatory subunit S3) (Proteasome subunit p58) (Transplantation antigen P91A) (Tum-P91A antigen)	Psmd3 P91a Tstap91a	regulation of protein catabolic process [GO:0042176]
178.26	162.52	550.13	64.47	196.73	252.51	1418.79	121.75	3871.69	74.98	113.38	1367.33	131.09	286.05	3339.54	520.85	135.41	3322.12	16107.6	Sodium/potassium-transporting ATPase subunit alpha-2 (Na(+)/K(+) ATPase alpha-2 subunit) (EC 7.2.2.13) (Sodium pump subunit alpha-2)	ATP1A2 KIAA0778	adult locomotory behavior [GO:0008344]; amygdala development [GO:0021764]; ATP metabolic process [GO:0046034]; behavioral fear response [GO:0001662]; cardiac muscle contraction [GO:0060048]; cell communication by electrical coupling involved in cardiac conduction [GO:0086064]; cellular response to mechanical stimulus [GO:0071260]; cellular response to steroid hormone stimulus [GO:0071383]; intracellular potassium ion homeostasis [GO:0030007]; intracellular sodium ion homeostasis [GO:0006883]; L-ascorbic acid metabolic process [GO:0019852]; locomotion [GO:0040011]; locomotory exploration behavior [GO:0035641]; membrane depolarization during cardiac muscle cell action potential [GO:0086012]; membrane repolarization [GO:0086009]; monoatomic cation transmembrane transport [GO:0098655]; negative regulation of calcium ion transmembrane transport

55.45	0	10.41	184.71	0	6.21	8322.97	0	2.82	92.22	287.22	3.85	12	0	4.11	6888.33	227.26	0	16097.56	Very-long-chain (3R)-3-hydroxyacyl-CoA dehydratase (EC 4.2.1.134) (3-hydroxyacyl-CoA dehydratase) (HACD) (Protein-tyrosine phosphatase-like A domain-containing protein 1)	hacd3 ptplad1 si:ch211-117i10.7 zgc:63632	fatty acid elongation [GO:0030497]; sphingolipid biosynthetic process [GO:0030148]; very long-chain fatty acid biosynthetic process [GO:0042761]
1094.14	507.55	1189.95	282.55	522.99	969.46	82.71	229.71	1814.97	326.73	161.07	1125.7	774.56	1136.23	2126.83	315.84	659.19	2759.52	16079.7	26S proteasome non-ATPase regulatory subunit 4 (26S proteasome regulatory subunit RPN10) (26S proteasome regulatory subunit S5A) (Multiubiquitin chain-binding protein)	Psmd4 Mcb1	
893.9	1073.65	1404.49	320.64	899.54	1904.15	135.24	392.84	110.5	436.87	411.18	880	2679.33	1655.41	1134.15	353.13	760.35	600.71	16046.08	Neutrophil cytosolic factor 1 (NCF-1)	Ncf1	cellular defense response [GO:0006968]; cellular response to glucose stimulus [GO:0071333]; cellular response to testosterone stimulus [GO:0071394]; defense response to bacterium [GO:0042742]; defense response to fungus [GO:0050832]; defense response to Gram-positive bacterium [GO:0050830]; epithelial cell proliferation [GO:0050673]; hydrogen peroxide biosynthetic process [GO:0050665]; inflammatory response [GO:0006954]; leukocyte mediated cytotoxicity [GO:0001909]; leukotriene metabolic process [GO:0006691]; neutrophil-mediated killing of fungus [GO:0070947]; neutrophil-mediated killing of gram-positive bacterium [GO:0070946]; protein targeting to membrane [GO:0006612]; reactive oxygen species biosynthetic process [GO:1903409]; regulation of respiratory burst involved in inflammatory response [GO:0060264]; respiratory burst [GO:0045730]; respiratory burst involved in defense response [illegible]

1716.93	608.58	1295.44	497.06	1224.26	2308.41	10.1	609.35	301.02	446.43	280.4	583.88	2021.27	1726.58	982.79	294.23	667.87	445.33	16019.93	Kinesin-like protein KIF1C	Kif1c	microtubule-based movement [GO:0007018]; retrograde vesicle-mediated transport, Golgi to endoplasmic reticulum [GO:0006890]
894.21	630.49	1314.85	233.47	647.49	748.98	175.49	339.32	2497.41	300.58	306.9	1214.74	665.04	876.34	2110.19	318.95	385.91	2347.72	16008.08	ATP-dependent RNA helicase DHX58 (EC 3.6.4.13) (ATP-dependent helicase LGP2) (Protein D11Lgp2 homolog) (RIG-I-like receptor 3) (RLR-3) (RIG-I-like receptor LGP2) (RLR)	DHX58 D11LGP2E LGP2	antiviral innate immune response [GO:0140374]; cytoplasmic pattern recognition receptor signaling pathway [GO:0002753]; negative regulation of innate immune response [GO:0045824]; negative regulation of MDA-5 signaling pathway [GO:0039534]; negative regulation of RIG-I signaling pathway [GO:0039536]; negative regulation of type I interferon production [GO:0032480]; positive regulation of MDA-5 signaling pathway [GO:1900245]; positive regulation of RIG-I signaling pathway [GO:1900246]; positive regulation of type I interferon production [GO:0032481]; regulation of innate immune response [GO:0045088]; response to bacterium [GO:0009617]; response to virus [GO:0009615]
1175.11	796.22	826.72	456.91	832.89	1407.01	2860.51	407.43	358.74	467.99	371.32	509.42	1237.43	880.75	549.47	1757.12	600.98	504.28	16000.3	Diablo IAP-binding mitochondrial protein (Diablo homolog, mitochondrial) (Direct IAP-binding protein with low pI) (Second mitochondria-derived activator of caspase) (Smac) [Cleaved into: Diablo IAP-binding mitochondrial protein, cleaved form]	Diablo Smac	intrinsic apoptotic signaling pathway [GO:0097193]; intrinsic apoptotic signaling pathway in response to oxidative stress [GO:0008631]; neuron apoptotic process [GO:0051402]; positive regulation of apoptotic process [GO:0043065]

1005.03	1430.14	2818.84	203.33	707.88	1655.32	444.18	177.34	421.32	435.64	334.95	908.34	649.05	1544.48	827.81	265.45	466.81	1652	15947.91	E3 SUMO-protein ligase PIAS2 (EC 2.3.2.-) (Androgen receptor-interacting protein 3) (ARIP3) (DAB2-interacting protein) (DIP) (E3 SUMO-protein transferase PIAS2) (Msx-interacting zinc finger protein) (Miz1) (PIAS-NY protein) (Protein inhibitor of activated STAT x) (Protein inhibitor of activated STAT2)	PIAS2 PIASX	DNA-templated transcription [GO:0006351]; negative regulation of androgen receptor signaling pathway [GO:0060766]; negative regulation of DNA-binding transcription factor activity [GO:0043433]; protein sumoylation [GO:0016925]; regulation of transcription by RNA polymerase II [GO:0006357]
1435.76	96.28	1674.58	275.94	436.91	1799.74	13.45	69.74	1193.77	670.27	260.18	2301.87	1370.83	342.25	1495.18	283.86	448.28	1760.61	15929.5	Protein ABHD18 (Alpha/beta hydrolase domain-containing protein 18) (Abhydrolase domain-containing protein 18)	Abhd18	
18.82	0	4.86	227.78	0	0	10580.49	0	0	79.05	181.35	0	0	0	0	4742.11	84.03	0	15918.49	FRAS1-related extracellular matrix protein 2 (ECM3 homolog)	FREM2	anatomical structure morphogenesis [GO:0009653]; cell adhesion [GO:0007155]; cell communication [GO:0007154]; embryonic digit morphogenesis [GO:0042733]; eye development [GO:0001654]; heart development [GO:0007507]; inner ear development [GO:0048839]; kidney development [GO:0001822]; morphogenesis of an epithelium [GO:0002009]

2903.86	31.79	18.6	764.08	216.16	525.88	11.84	18.16	0.33	2876.04	149.74	124.87	7919.32	24.04	25.55	213.4	46.38	0.75	15870.79	Ankyrin repeat and SAM domain-containing protein 1A (Odin)	ANKS1A ANKS1 KIAA0229 ODIN	ephrin receptor signaling pathway [GO:0048013]; neuron remodeling [GO:0016322]; substrate-dependent cell migration [GO:0006929]
0	0	4330.24	0	0	0	142.25	0	3858.35	0	6.1	1053.14	0	0	1971.12	83.26	13.99	4386.85	15845.3	Gap junction beta-1 protein (Connexin-32) (Cx32)	GJB1 CXN-32	cell-cell signaling [GO:0007267]; purine ribonucleotide transport [GO:0015868]
3604.03	44.26	28.02	833.73	214.06	375.83	28.26	16.47	0	2910.67	132.2	131.37	7169.17	20.95	17.15	200.51	41.78	4.63	15773.09	Cadherin EGF LAG seven-pass G-type receptor 2 (Cadherin family member 10) (Epidermal growth factor-like protein 2) (EGF-like protein 2) (Flamingo homolog 3) (Multiple epidermal growth factor-like domains protein 3) (Multiple EGF-like domains protein 3)	CELSR2 CDHF10 EGFL2 KIAA0279 MEGF3	cell-cell adhesion [GO:0098609]; cerebrospinal fluid secretion [GO:0033326]; cilium assembly [GO:0060271]; cilium movement [GO:0003341]; dendrite morphogenesis [GO:0048813]; G protein-coupled receptor signaling pathway [GO:0007186]; homophilic cell adhesion via plasma membrane adhesion molecules [GO:0007156]; motor neuron migration [GO:0097475]; neural plate anterior/posterior regionalization [GO:0021999]; regulation of cell-cell adhesion [GO:0022407]; regulation of DNA-templated transcription [GO:0006355]; regulation of protein localization [GO:0032880]; ventricular system development [GO:0021591]; Wnt signaling pathway [GO:0016055]; Wnt signaling pathway, planar cell polarity pathway [GO:0060071]

1387	670.4	881.96	446.87	698.38	2720.88	1190.86	251.41	215.74	814	374.67	884.45	2039.62	740.96	734.47	760.96	633.07	298.38	15744.08	Rapamycin-insensitive companion of mTOR (AVO3 homolog) (hAVO3)	RICTOR KIAA1999	actin cytoskeleton organization [GO:0030036]; cellular response to nutrient levels [GO:0031669]; cytoskeleton organization [GO:0007010]; embryo development ending in birth or egg hatching [GO:0009792]; lipid biosynthetic process [GO:0008610]; negative regulation of apoptotic process [GO:0043066]; positive regulation of actin filament polymerization [GO:0030838]; positive regulation of cell growth [GO:0030307]; positive regulation of cell migration [GO:0030335]; positive regulation of endothelial cell proliferation [GO:0001938]; positive regulation of phosphatidylinositol 3-kinase/protein kinase B signal transduction [GO:0051897]; positive regulation of TOR signaling [GO:0032008]; regulation of actin cytoskeleton organization [GO:0032956]; regulation of establishment of cell polarity [GO:2000114]; regulation of gene expression [GO:0010468]; regulation of
702.48	1110.15	1240.99	291.85	1406.51	3072.01	430.54	397.17	229.58	350.13	391.17	748.03	680.48	1485.41	1241.37	537.68	1140.94	270.88	15727.37	Palmitoyltransferase ZDHHC17 (EC 2.3.1.225) (Acyltransferase ZDHHC17) (EC 2.3.1.-) (DHHC domain-containing cysteine-rich protein 17) (Zinc finger DHHC domain-containing protein 17)	zdhhc17 dhhc17	axonogenesis [GO:0007409]; habituation [GO:0046959]; regulation of ERK1 and ERK2 cascade [GO:0070372]; regulation of neurotrophin TRK receptor signaling pathway [GO:0051386]
862.84	1386.7	1090.84	672.01	1193.78	1165.53	782.97	810.89	494.63	388.79	513.69	671.47	1213.18	1087.59	813.44	657.19	1232.98	686.59	15725.11	Myophilin		actin filament organization [GO:0007015]
32.62	20.2	3876.7	7.9	13.86	17.61	0.78	2.41	3965.97	7.52	10.39	1151.69	20.22	9.61	2981.54	2.75	11.65	3566.29	15699.71	GTP-binding protein RAD (RAD1) (Ras associated with diabetes)	RRAD RAD	small GTPase-mediated signal transduction [GO:0007264]

529.88	274.08	1970.43	161.91	352.58	1609.33	51.07	127.43	2423.51	138.84	134.36	1787.97	493.47	464.21	2231.27	97.54	156.83	2683.4	15688.11	Voltage-gated inwardly rectifying potassium channel KCNH6 (Ether-a-go-go-related gene potassium channel 2) (ERG-2) (Eag-related protein 2) (Ether-a-go-go-related protein 2) (hERG-2) (hERG2) (Potassium voltage-gated channel subfamily H member 6) (Voltage-gated potassium channel subunit Kv11.2)	KCNH6 ERG2	membrane repolarization during cardiac muscle cell action potential [GO:0086013]; potassium ion transmembrane transport [GO:0071805]; regulation of heart rate by cardiac conduction [GO:0086091]; regulation of ventricular cardiac muscle cell membrane repolarization [GO:0060307]
1.9	1.08	825.4	0	1.64	14.8	0	1.3	3816.26	2.92	2.33	1357.14	181.34	14.9	4390.13	2.05	8.57	5048.5	15670.26	Voltage-dependent T-type calcium channel subunit alpha-1H (Voltage-gated calcium channel subunit alpha Cav3.2)	Cacna1h	aldosterone biosynthetic process [GO:0032342]; calcium ion import [GO:0070509]; calcium ion transport [GO:0006816]; cellular response to hormone stimulus [GO:0032870]; cellular response to potassium ion [GO:0035865]; cortisol biosynthetic process [GO:0034651]; inorganic cation transmembrane transport [GO:0098662]; membrane depolarization during action potential [GO:0086010]; positive regulation of acrosome reaction [GO:2000344]; positive regulation of calcium ion-dependent exocytosis [GO:0045956]; positive regulation of cardiac muscle cell contraction [GO:0106134]; regulation of membrane potential [GO:0042391]
5.1	0.4	762.63	5.3	0.36	7.64	16.43	0.22	4859.12	1.11	1.86	1183.94	0	0	3981.39	30.5	9.96	4803.07	15669.03	Enhancer of mRNA-decapping protein 4	edc4 si:dkey-16n13.6	deadenylation-independent decapping of nuclear-transcribed mRNA [GO:0031087]

378.99	554.4	2296.41	198.4	640.32	1116.87	3.22	365.94	1200.44	158.74	200.26	1120.34	656.51	1116.44	2568.4	171.92	498.04	2370.13	15615.77	Cholesterol 7-desaturase nvd (EC 1.14.19.21) (Protein neverland) (Nvd protein) (nvd-Dr)	nvd zgc:92275	cholesterol metabolic process [GO:0008203]
755.74	885.17	1598.58	398.07	1194.18	2484.43	82.47	192.35	44.82	400.96	545.83	1745.17	1835.27	1528.13	510.25	382.18	913.71	104.3	15601.61	Myosin light chain kinase family member 4 (EC 2.7.11.1) (Sugen kinase 85) (SgK085)	MYLK4 SGK085	signal transduction [GO:0007165]
798.06	846.57	8288.92	147.71	365.09	413.51	40.23	102.99	2238.83	166.9	217.76	1663.39	33.47	6.27	61.67	0	0	207.92	15599.29	Large ribosomal subunit protein uL10 (60S acidic ribosomal protein P0) (60S ribosomal protein L10E)	RPLP0	cytoplasmic translation [GO:0002181]; ribosomal large subunit assembly [GO:0000027]
5.19	6953.38	21.84	2.61	978.16	4.31	0	2820.16	17.73	9.66	1421.13	0	0.77	2470.38	24.14	6.55	821.61	29.31	15586.93	Plasma kallikrein (EC 3.4.21.34) (Fletcher factor) (Kininogenin) (Plasma prekallikrein) [Cleaved into: Plasma kallikrein heavy chain;Plasma kallikrein light chain]	Klkb1 Klk3 Pk	blood coagulation [GO:0007596]; fibrinolysis [GO:0042730]; inflammatory response [GO:0006954]; liver regeneration [GO:0097421]; plasminogen activation [GO:0031639]; positive regulation of fibrinolysis [GO:0051919]

236.08	112.53	74.39	90.43	100.76	344.34	9796.29	41.47	187.35	115.28	108.2	122.79	326.42	219.13	240.53	3205	117.63	134.99	15573.61	E3 ubiquitin-protein ligase CHIP (EC 2.3.2.27) (Carboxy terminus of Hsp70-interacting protein) (RING-type E3 ubiquitin transferase CHIP) (STIP1 homology and U box-containing protein 1)	Stub1 Chip	cellular response to heat [GO:0034605]; cellular response to hypoxia [GO:0071456]; cellular response to misfolded protein [GO:0071218]; chaperone-mediated autophagy [GO:0061684]; DNA repair [GO:0006281]; endoplasmic reticulum unfolded protein response [GO:0030968]; ERAD pathway [GO:0036503]; MAPK cascade [GO:0000165]; negative regulation of apoptotic process [GO:0043066]; negative regulation of cardiac muscle hypertrophy [GO:0010614]; negative regulation of peroxisome proliferator activated receptor signaling pathway [GO:0035359]; negative regulation of smooth muscle cell apoptotic process [GO:0034392]; negative regulation of transforming growth factor beta receptor signaling pathway [GO:0030512]; negative regulation of vascular associated smooth muscle contraction [GO:1904694]; positive regulation of chaperone-mediated protein complex assembly
2391.37	245.29	3213.48	361.32	141.72	1059.15	33.82	101.68	789.76	636.37	85.86	844.12	1373.86	287.6	1837.63	253.18	265.42	1633.11	15554.74	1-phosphatidylinositol 4,5-bisphosphate phosphodiesterase beta-1 (EC 3.1.4.11) (PLC-154) (Phosphoinositide phospholipase C-beta-1) (Phospholipase C-beta-1) (PLC-beta-1)	PLCB1	G protein-coupled receptor signaling pathway [GO:0007186]; interleukin-1-mediated signaling pathway [GO:0070498]; interleukin-12-mediated signaling pathway [GO:0035722]; interleukin-15-mediated signaling pathway [GO:0035723]; lipid catabolic process [GO:0016042]; memory [GO:0007613]; phosphatidylinositol metabolic process [GO:0046488]; phosphatidylinositol-mediated signaling [GO:0048015]; positive regulation of JNK cascade [GO:0046330]; release of sequestered calcium ion into cytosol [GO:0051209]

9.49	3832.7	43.4	27.11	2761.36	37.72	0	1762.19	21.05	6.1	615.23	16.88	15.29	4318.16	26.02	2.88	2009.08	39.66	15544.32	DNA topoisomerase 2-binding protein 1-A (Cut5 protein) (DNA topoisomerase II-binding protein 1-A) (TopBP1-A) (XtopBP)	topbp1-A cut5	chromosome organization [GO:0051276]; DNA amplification [GO:0006277]; DNA damage response [GO:0006974]; DNA replication checkpoint signaling [GO:0000076]; DNA replication initiation [GO:0006270]; DNA replication preinitiation complex assembly [GO:0071163]; double-strand break repair via alternative nonhomologous end joining [GO:0097681]; double-strand break repair via homologous recombination [GO:0000724]; GINS complex assembly [GO:0071165]; mitotic DNA replication checkpoint signaling [GO:0033314]; mitotic G2 DNA damage checkpoint signaling [GO:0007095]; positive regulation of chromatin binding [GO:0035563]; positive regulation of peptidyl-serine phosphorylation [GO:0033138]; positive regulation of protein kinase activity [GO:0045860]; protein localization to chromatin [GO:0071168]; regulation of DNA-templated DNA replication initiation [GO:0030174]
180.38	749.72	1200.26	194.3	1322.22	900.22	110.97	320.39	1188.98	8.18	218.02	744.33	56.1	3144.78	1624.25	846.06	1626.09	1105.8	15541.05	DCN1-like protein 5 (DCNL5) (DCUN1 domain-containing protein 5) (Defective in cullin neddylation protein 1-like protein 5) (Squamous cell carcinoma-related oncogene 5)	DCUN1D5 SCCRO5	DNA damage response [GO:0006974]; positive regulation of protein neddylation [GO:2000436]; protein neddylation [GO:0045116]; regulation of cell growth [GO:0001558]; regulation of protein neddylation [GO:2000434]
1550.75	721.08	904.8	297.22	903.1	939.91	77.02	445.01	1599.42	438.46	328.25	917.11	949.41	1157.2	1312.25	279.1	690.34	2028.98	15539.41	NHS-like protein 2	NHSL2	cell differentiation [GO:0030154]

1147.35	835.48	644.48	225.05	1588.82	1046.38	711	1193.7	295.56	370.32	687.34	522.78	2073.13	1641.58	487.22	494.95	1076.5	465.02	15506.66	Transcription factor SOX-5	SOX5	asymmetric neuroblast division [GO:0055059]; cartilage condensation [GO:0001502]; cartilage development [GO:0051216]; cell fate commitment [GO:0045165]; cellular response to transforming growth factor beta stimulus [GO:0071560]; chondrocyte differentiation [GO:0002062]; positive regulation of cartilage development [GO:0061036]; positive regulation of chondrocyte differentiation [GO:0032332]; positive regulation of mesenchymal stem cell differentiation [GO:2000741]; regulation of transcription by RNA polymerase II [GO:0006357]; transcription by RNA polymerase II [GO:0006366]
308.64	501.6	1021.07	66.64	599.41	1351.76	822.38	256.38	1568.16	112.88	341.99	1529.56	333.35	961.57	2647.56	509.45	341.34	2228.08	15501.82	Methylcytosine dioxygenase TET2 (EC 1.14.11.80)	TET2 KIAA1546 Nbla00191	chromosomal 5-methylcytosine DNA demethylation, oxidation pathway [GO:0141167]; leukocyte differentiation [GO:0002521]; myeloid cell differentiation [GO:0030099]; positive regulation of gene expression via chromosomal CpG island demethylation [GO:0044029]; positive regulation of transcription by RNA polymerase II [GO:0045944]; protein O-linked glycosylation [GO:0006493]
26.8	3.98	897.21	15.89	11.68	1.87	32.54	1.72	4813.25	12.84	7.46	1546.11	85.71	11.58	2481.51	35.49	26.91	5487.6	15500.15	FGFR1 oncogene partner 2 homolog	fgfr1op2 si:ch211-203h15.2 zgc:64126	response to wounding [GO:0009611]
832.99	1500.97	1262.19	421.12	1322.18	1654.58	319.4	606.06	506.06	361.56	448.2	882.62	1108.99	1461.1	831.1	446.89	800.65	732.62	15499.28	Echinoderm microtubule-associated protein-like 5 (EMAP-5)	Eml5	

2.6		3965.33	54.55	28.05	3073.24	39.06	473.21	1069.5	14.71	7.42	487	1.27	0.85	4032.19	1.59	385.28	1828.92	18.73	15483.5	ATP-sensitive inward rectifier potassium channel 8 (Inward rectifier K(+) channel Kir6.1) (Potassium channel, inwardly rectifying subfamily J member 8) (uKATP-1)	KCNJ8	adaptive immune response [GO:0002250]; apoptotic process [GO:0006915]; atrioventricular node cell differentiation [GO:0060922]; calcium ion transmembrane transport [GO:0070588]; CAMKK-AMPK signaling cascade [GO:0061762]; coronary vasculature development [GO:0060976]; defense response to virus [GO:0051607]; determination of adult lifespan [GO:0008340]; establishment of cell polarity [GO:0030010]; fat cell differentiation [GO:0045444]; fatty acid transport [GO:0015908]; fibroblast proliferation [GO:0048144]; gene expression [GO:0010467]; glutamate secretion, neurotransmission [GO:0061535]; heart morphogenesis [GO:0003007]; inorganic cation transmembrane transport [GO:0098662]; kidney development [GO:0001822]; membrane repolarization during ventricular cardiac muscle cell action potential [GO:0098915]; microglial cell activation [GO:0001774]; neuromuscular process
287.66		233.19	931.2	295.74	220.17	533.38	3941.58	112.55	1256.6	164.09	163.84	733.13	611.88	335.22	1491.18	2001.25	308.73	1789.75	15411.14	Phospholipase B-like 1 (EC 3.1.1.-) (LAMA-like protein 1) (Lamina ancestor homolog 1) (Phospholipase B domain-containing protein 1) [Cleaved into: Phospholipase B-like 1 chain A;Phospholipase B-like 1 chain B;Phospholipase B-like 1 chain C]	Plbd1	phospholipid catabolic process [GO:0009395]

0	30.62	0	9.66	16.86	0	1.56	4.59	1.46	0	28.66	0	9	11573.21	0	2.35	3705.83	20.74	15404.54	Cystathionine gamma-lyase (EC 4.4.1.1) (Cysteine-protein sulfhydrase) (Gamma-cystathionase)	CTH	cysteine biosynthetic process [GO:0019344]; cysteine biosynthetic process via cystathionine [GO:0019343]; hydrogen sulfide biosynthetic process [GO:0070814]; lipid metabolic process [GO:0006629]; negative regulation of apoptotic process [GO:0043066]; positive regulation of canonical NF-kappaB signal transduction [GO:0043123]; positive regulation of NF-kappaB transcription factor activity [GO:0051092]; protein sulfhydration [GO:0044524]; protein-pyridoxal-5-phosphate linkage via peptidyl-N6-pyridoxal phosphate-L-lysine [GO:0018272]; transsulfuration [GO:0019346]
1608.07	343.69	2280.94	366.82	501.86	1933.73	239.46	175.35	686.05	474.33	265.56	1664.32	1821.92	288.63	848.25	363.48	476.74	1023.87	15363.07	DENN domain-containing protein 2C	DENND2C	
10.33	1.44	2185.46	0.91	0.62	602.44	2.07	2.64	2227.82	2.7	2.25	6548.05	29.91	2.12	1397.67	3.84	1.72	2310.5	15332.49	Alanine--tRNA ligase, mitochondrial (EC 6.1.1.7) (Alanyl-tRNA synthetase) (AlaRS) (Protein lactyltransferase AARS2) (EC 6.-.-.-)	Aars2 Aarsl Gm89 Kiaa1270	mitochondrial alanyl-tRNA aminoacylation [GO:0070143]; negative regulation of cGAS/STING signaling pathway [GO:0160049]
389.29	530.04	688.13	563.25	1418.36	2131.86	1099.13	666.24	930.49	288.9	270.55	445.79	1330.98	1533.24	987.31	682.63	638.59	697.01	15291.79	Serine/threonine-protein kinase RIO1 (EC 2.7.11.1) (EC 3.6.1.-) (RIO kinase 1)	RIOK1 RIO1	maturation of SSU-rRNA [GO:0030490]; positive regulation of rRNA processing [GO:2000234]; ribosomal small subunit biogenesis [GO:0042274]
761.1	605.87	1651.27	354.03	707.7	1426.47	542.24	217.3	1621.6	291.14	216.96	1223.77	712.38	608.91	1517.87	512.39	393.16	1845.45	15209.61	Actin-2 (EC 3.6.4.-)		

904.36	450.04	344.13	243.7	548.37	414.99	3279.13	267.14	95.45	205.97	295.97	194.36	1575.02	1272.65	364.09	3601.1	856.54	287.37	15200.38	Bridge-like lipid transfer protein family member 3B (Syntaxin-6 Habc-interacting protein of 164 kDa) (UHRF1-binding protein 1-like)	Bltp3b Kiaa0701 Ship164 Uhrf1bp1l	early endosome to Golgi transport [GO:0034498]; intermembrane lipid transfer [GO:0120009]
4477.76	199.04	1010.57	652.02	178.29	1286.38	6.94	76.96	55.6	489.72	108.07	531.54	3297.28	614.42	1086.77	295.93	604.28	227.38	15198.95	Mitotic interactor and substrate of PLK1 (Mitotic spindle positioning protein)	MISP C19orf21	cell division [GO:0051301]; cell migration [GO:0016477]; establishment of centrosome localization [GO:0051660]; establishment of mitotic spindle orientation [GO:0000132]; mitotic spindle assembly [GO:0090307]; organelle localization [GO:0051640]; regulation of protein localization to cell cortex [GO:1904776]
0	0	8.68	256.69	0	9.04	8209.88	3.36	0	83.56	207.97	7.55	0	0	0	6237.82	120.23	6.85	15151.63	Fibroblast growth factor receptor substrate 2 (FGFR substrate 2) (FGFR-signaling adaptor SNT) (Suc1-associated neurotrophic factor target 1) (SNT-1)	FRS2	anterior/posterior axis specification, embryo [GO:0008595]; cell surface receptor protein tyrosine phosphatase signaling pathway [GO:0007185]; fibroblast growth factor receptor signaling pathway [GO:0008543]; forebrain development [GO:0030900]; G protein-coupled receptor signaling pathway [GO:0007186]; gastrulation with mouth forming second [GO:0001702]; lens fiber cell development [GO:0070307]; lens placode formation involved in camera-type eye formation [GO:0046619]; negative regulation of cardiac muscle cell differentiation [GO:2000726]; neuroblast proliferation [GO:0007405]; organ induction [GO:0001759]; positive regulation of MAPK cascade [GO:0043410]; prostate epithelial cord arborization involved in prostate glandular acinus morphogenesis [GO:0060527]; regulation of apoptotic process [GO:0042981]; regulation of epithelial cell proliferation [GO:0050678]; regulation of ERK1 and

5795.12	327.24	514.71	469.78	160.45	199.2	7.49	106.27	109.13	630.57	101.02	199.36	4024.84	754.26	396.03	376.04	810.09	164.18	15145.78	Slit homolog 2 protein (Slit-2) [Cleaved into: Slit homolog 2 protein N-product;Slit homolog 2 protein C-product]	Slit2	aortic valve morphogenesis [GO:0003180]; axon guidance [GO:0007411]; axonogenesis [GO:0007409]; cell-cell adhesion [GO:0098609]; dorsal/ventral axon guidance [GO:0033563]; in utero embryonic development [GO:0001701]; kidney development [GO:0001822]; mammary duct terminal end bud growth [GO:0060763]; mammary gland duct morphogenesis [GO:0060603]; metanephros development [GO:0001656]; negative chemotaxis [GO:0050919]; negative regulation of axon extension [GO:0030517]; negative regulation of cell population proliferation [GO:0008285]; negative regulation of gene expression [GO:0010629]; neuron projection morphogenesis [GO:0048812]; olfactory bulb development [GO:0021772]; pulmonary valve morphogenesis [GO:0003184]; retinal ganglion cell axon guidance [GO:0031290]; telencephalon cell migration [GO:0022029]; ventricular septum morphogenesis
41.89	35.63	712.5	4.11	29.83	174.44	2.54	8.57	5352.69	18.22	4.91	1858.89	41.67	14.86	2794.29	13.72	22.65	3940.57	15071.98	Protein O-linked-mannose beta-1,2-N-acetylglucosaminyltransferase 1 (POMGnT1) (EC 2.4.1.-)	POMGNT1	O-glycan processing [GO:0016266]; protein O-linked glycosylation [GO:0006493]
637.86	1624.01	1162.67	236.07	1104.71	944.28	521.45	411.99	987.81	256.06	409.19	718.16	440.04	1879.28	1033.85	608.06	924.23	1155.68	15055.4	PDZ domain-containing protein 8	Pdzd8	cytoskeleton organization [GO:0007010]; lipid transport [GO:0006869]; mitochondrial calcium ion homeostasis [GO:0051560]; mitochondrion-endoplasmic reticulum membrane tethering [GO:1990456]; regulation of cell morphogenesis [GO:0022604]

0	0	542.84	0	0	0	0	0	5703.39	0	0	992.03	0	0	3021.25	0	0	4776.86	15036.37	Potassium channel subfamily K member 3 (Acid-sensitive potassium channel protein TASK-1) (TWIK-related acid-sensitive K(+) channel 1) (Two pore potassium channel KT3.1) (Two pore K(+) channel KT3.1)	Kcnk3 Task Task1	cellular response to acidic pH [GO:0071468]; cellular response to hypoxia [GO:0071456]; cellular response to zinc ion [GO:0071294]; cochlea development [GO:0090102]; detection of hypoxic conditions in blood by carotid body chemoreceptor signaling [GO:0003029]; monoatomic ion transmembrane transport [GO:0034220]; negative regulation of cytosolic calcium ion concentration [GO:0051481]; potassium ion transmembrane transport [GO:0071805]; potassium ion transport [GO:0006813]; regulation of action potential firing rate [GO:0099605]; regulation of resting membrane potential [GO:0060075]; response to xenobiotic stimulus [GO:0009410]
756.88	666.27	381.8	289.87	601.47	905.59	3531.43	415.92	541.53	295.99	268.62	352.21	1155.1	1057.51	597.02	1892.88	676.79	561.43	14948.31	TNF receptor-associated factor 3 (EC 2.3.2.27) (CD40 receptor-associated factor 1) (CRAF1) (CD40-binding protein) (CD40BP) (RING-type E3 ubiquitin transferase TRAF3)	Traf3 Cap-1 Craf1 Trafamn	apoptotic process [GO:0006915]; innate immune response [GO:0045087]; negative regulation of NF-kappaB transcription factor activity [GO:0032088]; positive regulation of type I interferon production [GO:0032481]; regulation of apoptotic process [GO:0042981]; regulation of cytokine production [GO:0001817]; regulation of defense response to virus [GO:0050688]; regulation of interferon-beta production [GO:0032648]; regulation of proteolysis [GO:0030162]; Toll signaling pathway [GO:0008063]; toll-like receptor 4 signaling pathway [GO:0034142]; toll-like receptor signaling pathway [GO:0002224]; tumor necrosis factor-mediated signaling pathway [GO:0033209]
1726.88	983.04	1182.26	752.9	775.37	1595.04	192.75	263.19	2079.29	1173.19	679.13	353.89	714.04	641.34	609.52	242.82	396.42	582.07	14943.14	Lecithin retinol acyltransferase (EC 2.3.1.135) (Phosphatidylcholine--retinol O-acyltransferase)	LRAT	retinol metabolic process [GO:0042572]; visual perception [GO:0007601]; vitamin A metabolic process [GO:0006776]

572.53		1558.85	1026.8	233.44	803.85	1044.61	1448.54	599.7	734.98	173.81	325.09	1212.49	785.45	904.24	877.74	1097.93	597.94	938.61	14936.6	Rho GTPase-activating protein 6 (Rho-type GTPase-activating protein 6) (Rho-type GTPase-activating protein RhoGAPX-1)	ARHGAP6 RHOGAP6	actin filament organization [GO:0007015]; actin filament polymerization [GO:0030041]; focal adhesion assembly [GO:0048041]; negative regulation of focal adhesion assembly [GO:0051895]; negative regulation of stress fiber assembly [GO:0051497]; positive regulation of intracellular signal transduction [GO:1902533]; positive regulation of phospholipase C/protein kinase C signal transduction [GO:0141214]; regulation of small GTPase mediated signal transduction [GO:0051056]; Rho protein signal transduction [GO:0007266]
1641.58		689.9	431.83	693.43	1155.49	1503.63	85.07	309.03	510.48	834.07	354.8	490.63	1165.84	2373.58	550.94	514.04	814.32	816.11	14934.77	Tyrosine-protein phosphatase non-receptor type 23 (EC 3.1.3.48)	Ptpn23 Kiaa1471	cilium assembly [GO:0060271]; early endosome to late endosome transport [GO:0045022]; endocytic recycling [GO:0032456]; negative regulation of epithelial cell migration [GO:0010633]; positive regulation of adherens junction organization [GO:1903393]; positive regulation of early endosome to late endosome transport [GO:2000643]; positive regulation of homophilic cell adhesion [GO:1903387]; positive regulation of Wnt protein secretion [GO:0061357]; protein transport [GO:0015031]; ubiquitin-dependent protein catabolic process via the multivesicular body sorting pathway [GO:0043162]

340.86	1279.02	1076	345.9	1491.66	960.34	1594.51	652.81	430.47	181.66	481.56	1260.03	609.45	1475.02	612.24	911.01	677.73	548.76	14929.03	Replication protein A 32 kDa subunit-B (RP-A p32) (Replication factor A protein 2) (RF-A protein 2) (Replication protein A 34 kDa subunit) (RP-A p34)	rpa2-b repa2 rpa32 rpa34	base-excision repair [GO:0006284]; DNA replication [GO:0006260]; DNA replication checkpoint signaling [GO:0000076]; double-strand break repair via homologous recombination [GO:0000724]; mismatch repair [GO:0006298]; nucleotide-excision repair [GO:0006289]; protein localization to chromosome [GO:0034502]; regulation of DNA damage checkpoint [GO:2000001]; telomere maintenance [GO:0000723]
1818.58	859.05	1322.84	373.7	1094.03	1670.55	260.56	391.39	91.23	321.37	396.55	642.22	1148.55	1914.44	878.19	466.5	941.93	310.08	14901.76	Carboxy-terminal domain RNA polymerase II polypeptide A small phosphatase 2 (EC 3.1.3.16) (Nuclear LIM interactor-interacting factor 2) (NLI-interacting factor 2) (Protein OS-4) (Small C-terminal domain phosphatase 2) (Small CTD phosphatase 2) (SCP2)	CTDSP2 NIF2 OS4 SCP2	negative regulation of G1/S transition of mitotic cell cycle [GO:2000134]; protein dephosphorylation [GO:0006470]
21.23	1.04	0	273.87	0	6.1	7641.98	0.75	0	64.18	189.91	1.43	12.88	1.26	0	6511.14	155.27	5.28	14886.32	Pre-mRNA-splicing factor 18 (PRP18 homolog)	prpf18 si:ch211-220f12.3	generation of catalytic spliceosome for second transesterification step [GO:0000350]
3676.79	591.66	1172.03	305.6	288.85	3343.24	23.2	156.18	239.5	383.57	224.04	750.73	2029.27	273.18	833.94	110.93	127.45	347.32	14877.48	Complement C1q tumor necrosis factor-related protein 6	C1QTNF6 CTRP6 UNQ581/PRO1151	

1152.57		632.81	1098.38	609.66	567.06	1590.27	1054.29	260.3	394.85	357.81	233.27	765.96	2068.93	809.52	737.82	1196.07	661.41	674.85	14865.83	T-cell surface glycoprotein CD4 (T-cell surface antigen T4/Leu-3) (CD antigen CD4)	CD4	adaptive immune response [GO:0002250]; calcium-mediated signaling [GO:0019722]; cell adhesion [GO:0007155]; cell surface receptor protein tyrosine kinase signaling pathway [GO:0007169]; cell surface receptor signaling pathway [GO:0007166]; cellular response to granulocyte macrophage colony-stimulating factor stimulus [GO:0097011]; cellular response to ionomycin [GO:1904637]; defense response to Gram-negative bacterium [GO:0050829]; enzyme-linked receptor protein signaling pathway [GO:0007167]; helper T cell enhancement of adaptive immune response [GO:0035397]; immune response [GO:0006955]; interleukin-15-mediated signaling pathway [GO:0035723]; macrophage differentiation [GO:0030225]; maintenance of protein location in cell [GO:0032507]; positive regulation of calcium ion transport into cytosol [GO:0010524]; positive regulation of
956.27		1107.59	1011.92	200.08	1252.97	1680.08	128.02	728.75	290.51	304.79	602.5	1057.3	870.15	2326.31	590.14	188.26	1000.61	541.66	14837.91	Erythropoietin receptor (EPO-R)	EPOR	brain development [GO:0007420]; cytokine-mediated signaling pathway [GO:0019221]; decidualization [GO:0046697]; heart development [GO:0007507]; positive regulation of cell population proliferation [GO:0008284]

1584.41	861.04	799.65	504.04	970.66	1001.85	499.35	501.32	278.5	652.15	439.68	677.51	2059.17	992.29	388.69	606.65	1406.49	600.13	14823.58	Fibroblast growth factor 11 (FGF-11) (Fibroblast growth factor homologous factor 3) (FHF-3)	FGF11 FHF3	cell-cell signaling [GO:0007267]; nervous system development [GO:0007399]; neurogenesis [GO:0022008]; signal transduction [GO:0007165]
446.7	1365.77	369.62	98.92	1721.15	331.75	14.19	673.19	1092.98	50.81	435.19	489.61	426.91	3505.82	1250.93	320.5	819.29	1408.81	14822.14	LIM domain-binding protein 1 (LDB-1) (Carboxyl-terminal LIM domain-binding protein 2) (CLIM-2) (LIM domain-binding factor CLIM2) (cLdb1) (Neural Src-interacting protein) (Nuclear LIM interactor)	LDB1 CLIM2 NSIP	anterior/posterior axis specification [GO:0009948]; negative regulation of erythrocyte differentiation [GO:0045647]; negative regulation of transcription by RNA polymerase II [GO:0000122]; nervous system development [GO:0007399]; neuron differentiation [GO:0030182]; positive regulation of transcription by RNA polymerase II [GO:0045944]; Wnt signaling pathway [GO:0016055]
1562.57	768.6	630.08	362.45	629.93	1183.02	1772	295.24	224.52	382.39	200.72	461.25	2333.4	787.31	850.41	1331.33	603.41	436.49	14815.12	Charged multivesicular body protein 2a (Chromatin-modifying protein 2a) (CHMP2a)	chmp2a TNeu078g14.1	exit from mitosis [GO:0010458]; nuclear membrane reassembly [GO:0031468]; protein transport [GO:0015031]; vacuolar transport [GO:0007034]

838.95	1484.52	944.43	232.73	1220.83	963.12	797.45	857.72	597.15	205.06	433.83	694.42	1068.9	1316.66	730.08	608.81	1151.23	631.07	14776.96	Large ribosomal subunit protein uL15m (39S ribosomal protein L15, mitochondrial) (L15mt) (MRP-L15)	mrpl15	translation [GO:0006412]
31.69	0	2.55	232.97	0	10.1	7273.01	0	0	61.74	194.32	0.74	3.84	0	0	6748.63	173.55	10.6	14743.74	Ankycorbin (Ankyrin repeat and coiled-coil structure-containing protein) (Retinoic acid-induced protein 14)	Rai14	cell differentiation [GO:0030154]; spermatogenesis [GO:0007283]
416	732.97	1761.57	323.91	540.05	477.1	1313.73	304.1	986.55	262.47	357.55	1993.65	587.75	1150.67	1058.92	793.66	609.35	1070.5	14740.5	Peptidyl-prolyl cis-trans isomerase FKBP14 (PPIase FKBP14) (EC 5.2.1.8) (22 kDa FK506-binding protein) (22 kDa FKBP) (FKBP-22) (FK506-binding protein 14) (FKBP-14) (Rotamase)	FKBP14 FKBP22 UNQ322/PRO381	
533.94	385.01	345.69	76.81	265.77	589.26	176.36	135.95	38.38	221.46	122.59	164.39	524.47	459.79	188.94	3006.47	7277.63	219.2	14732.11	Protein jagged-2 (Jagged2)	Jag2	auditory receptor cell fate commitment [GO:0009912]; cell fate determination [GO:0001709]; epithelial cell apoptotic process involved in palatal shelf morphogenesis [GO:1990134]; gamma-delta T cell differentiation [GO:0042492]; in utero embryonic development [GO:0001701]; morphogenesis of embryonic epithelium [GO:0016331]; Notch signaling pathway [GO:0007219]; odontogenesis of dentin-containing tooth [GO:0042475]; positive regulation of Notch signaling pathway [GO:0045747]; regulation of cell adhesion [GO:0030155]; regulation of cell population proliferation [GO:0042127]; respiratory system process [GO:0003016]; sensory perception of sound [GO:0007605]; skeletal system development [GO:0001501]; spermatogenesis [GO:0007283]; thymic T cell selection [GO:0045061]

1681.17	19.44	672.03	465.42	34.33	1612.5	13.65	14.65	3162.31	643.93	13.77	813.08	393.99	28.88	1947.05	26.22	33.6	3119.3	14695.32	Little elongation complex subunit 2 (Interactor of little elongator complex ELL subunit 2) (NMDA receptor-regulated protein 2)	ICE2 BRCC1 NARG2 UNQ3101/PRO10100	positive regulation of transcription by RNA polymerase III [GO:0045945]; snRNA transcription by RNA polymerase II [GO:0042795]; snRNA transcription by RNA polymerase III [GO:0042796]
251.07	4033.86	334.79	429.06	3467.49	95.66	2.33	2570.92	7.59	148.54	657.47	546.71	54.75	814.71	12.59	31.7	1184.18	14.15	14657.57	GON-4-like protein (GON-4 homolog)	GON4L GON4 KIAA1606	regulation of DNA-templated transcription [GO:0006355]
476.81	143.6	1460.28	241.86	206.79	706.45	5229.76	132.97	206.46	230.42	163.57	422.7	663.48	235.77	362.42	3040.98	393.62	339.04	14656.98	Palmitoyltransferase ZDHHC16A (EC 2.3.1.225) (Zinc finger DHHC domain-containing protein 16A) (DHHC-16A)	zdhhc16a zdhhc16	commitment of multipotent stem cells to neuronal lineage in forebrain [GO:0021898]; DNA damage response [GO:0006974]; eye development [GO:0001654]; fibroblast growth factor receptor signaling pathway involved in forebrain neuron fate commitment [GO:0021899]; heart development [GO:0007507]; protein palmitoylation [GO:0018345]; telencephalon development [GO:0021537]

484.41	1141.63	1122.63	251.76	1005.86	828.32	1526.96	434.38	828.52	207.34	316.29	772.83	864.16	1147.2	892.59	1314.01	605.65	911.34	14655.88	Actin-related protein 3 (Actin-like protein 3)	actr3 arp3	Arp2/3 complex-mediated actin nucleation [GO:0034314]; positive regulation of transcription by RNA polymerase II [GO:0045944]
406.43	779.94	478.99	192.89	628.78	620.49	2966.66	276.64	861.29	122.58	265.78	849.8	600.88	1671.69	889.98	1655.09	631.69	755.34	14654.94	Protein FAM210B, mitochondrial	FAM210B C20orf108 PSEC0265	cellular response to estradiol stimulus [GO:0071392]; erythrocyte maturation [GO:0043249]; inflammatory response [GO:0006954]; positive regulation of erythrocyte differentiation [GO:0045648]; reactive oxygen species metabolic process [GO:0072593]; skin development [GO:0043588]; spleen development [GO:0048536]
532.32	322.74	1566.64	211.51	365.59	645.84	835.04	129.59	2436.59	180.47	146.26	1165.26	652.68	600.62	1535.14	828.28	361.47	2126.45	14642.49	Clathrin light chain B (Lcb)	CLTB CLTLB	clathrin-dependent endocytosis [GO:0072583]; intracellular protein transport [GO:0006886]

774.89	942.41	1122.15	184.57	702.07	900.88	335.61	279.85	1513.87	172.56	270.02	1050.71	644.89	1653.14	1382.6	358.17	591.18	1751.4	14630.97	Protein lyl-1 (Class A basic helix-loop-helix protein 18) (bHLHa18) (Lymphoblastic leukemia-derived sequence 1)	LYL1 BHLHA18	B cell differentiation [GO:0030183]; blood vessel maturation [GO:0001955]; definitive hemopoiesis [GO:0060216]; positive regulation of DNA-templated transcription [GO:0045893]; regulation of DNA-templated transcription [GO:0006355]; regulation of transcription by RNA polymerase II [GO:0006357]
508.68	412.09	680.59	237.69	411.14	737.56	3377.51	165.55	1067.03	199.24	253.77	518.42	436.88	704.62	845.8	2760.16	375.21	936.31	14628.25	RNA-binding protein 25 (Arg/Glu/Asp-rich protein of 120 kDa) (RED120) (Protein S164) (RNA-binding motif protein 25) (RNA-binding region-containing protein 7)	RBM25 RNPC7	mRNA processing [GO:0006397]; regulation of alternative mRNA splicing, via spliceosome [GO:0000381]; regulation of apoptotic process [GO:0042981]; RNA splicing [GO:0008380]
0	7.8	1864.4	0	0.96	2.61	0.35	0.74	4058.52	1.18	1.31	880.08	1.53	0.77	3479.17	14.35	2.93	4305.46	14622.16	Endophilin-B2 (SH3 domain-containing GRB2-like protein B2)	SH3GLB2 KIAA1848 PP578	membrane organization [GO:0061024]
1224.02	856.06	1224.55	360.25	816.8	1301.47	56.01	393.08	1443.43	395.12	262.73	988.23	992.86	938.97	1291.26	154.26	507.92	1411.55	14618.57	Serine/threonine-protein kinase tousled-like 2 (EC 2.7.11.1) (PKU-alpha) (Tousled-like kinase 2)	tlk2 zgc:136697	chromosome segregation [GO:0007059]; intracellular signal transduction [GO:0035556]

5491.07	127.53	1726.74	666.94	101.88	3870.48	5.83	52.32	344.18	892.94	50.01	333.01	211.53	99.57	49.79	40.26	242.88	260.95	14567.91	Membrane-bound transcription factor site-2 protease (EC 3.4.24.85) (Endopeptidase S2P) (Sterol regulatory element-binding proteins intramembrane protease) (SREBPs intramembrane protease)	MBTPS2 S2P	ATF6-mediated unfolded protein response [GO:0036500]; bone maturation [GO:0070977]; cholesterol metabolic process [GO:0008203]; endoplasmic reticulum unfolded protein response [GO:0030968]; membrane protein intracellular domain proteolysis [GO:0031293]; mitotic G2 DNA damage checkpoint signaling [GO:0007095]; positive regulation of cholesterol biosynthetic process [GO:0045542]; positive regulation of transcription by RNA polymerase II [GO:0045944]; protein maturation [GO:0051604]; regulation of cholesterol biosynthetic process [GO:0045540]; regulation of response to endoplasmic reticulum stress [GO:1905897]; response to endoplasmic reticulum stress [GO:0034976]
34.53	3.99	771.43	4.7	9.71	137.96	3.11	9.45	4415.88	3.85	2.86	1650.34	35.51	6.9	3251.09	2.91	4.09	4207.55	14555.86	Semaphorin-4B (Semaphorin-C) (Sema C)	Sema4b Kiaa1745 Semac SemC	cell differentiation [GO:0030154]; nervous system development [GO:0007399]
0.52	4.05	1858.19	0.15	5.9	2.21	98.99	1	4197.62	1.31	3.8	1236.62	1.46	7.53	3802.15	54.62	5.99	3266.47	14548.58	Low-density lipoprotein receptor-related protein 1 (LRP-1) (Alpha-2-macroglobulin receptor) (A2MR)	LRP1	endocytosis [GO:0006897]

332.81	314.81	1744.34	178.12	305.31	1214.32	16.51	282.17	1856.2	100.17	145.33	1018.95	609.5	562.2	3312.33	63.53	448.39	2032.64	14537.63	Chemokine-like protein TAFA-5	TAFA5 FAM19A5	G protein-coupled receptor signaling pathway [GO:0007186]; negative regulation of vascular associated smooth muscle cell proliferation [GO:1904706]; negative regulation of vascular wound healing [GO:0061044]
2.7	2.38	5303.63	2.72	7.32	1041.27	0.37	4.05	23.59	4.5	5.25	7965.58	2.14	4.64	78.27	21.56	26.74	7.94	14504.65	Pre-mRNA-processing-splicing factor 8 (220 kDa U5 snRNP-specific protein) (PRP8 homolog) (Splicing factor Prp8) (p220)	PRPF8 PRPC8	cellular response to lipopolysaccharide [GO:0071222]; cellular response to tumor necrosis factor [GO:0071356]; mRNA processing [GO:0006397]; mRNA splicing, via spliceosome [GO:0000398]; RNA splicing [GO:0008380]; RNA splicing, via transesterification reactions [GO:0000375]; spliceosomal tri-snRNP complex assembly [GO:0000244]
124.2	733.48	209.63	172.76	1494.82	1425.63	3.34	1415.57	105.34	215.04	926.72	840.48	1165.11	2275.63	792.21	149.82	1583.39	866.78	14499.95	THAP domain-containing protein 11	thap11 zgc:65871	regulation of transcription by RNA polymerase II [GO:0006357]

309.75	848.31	590.59	634.17	1536.71	738.07	976.73	818.56	574.19	366.67	709.02	964.83	89.23	586.41	52.63	1593.6	2226.65	882.84	14498.96	Heat shock cognate 71 kDa protein (EC 3.6.4.10) (Heat shock 70 kDa protein 8)	HSPA8 HSC70	chaperone cofactor-dependent protein refolding [GO:0051085]; clathrin coat disassembly [GO:0072318]; mRNA processing [GO:0006397]; negative regulation of DNA-templated transcription [GO:0045892]; protein refolding [GO:0042026]; protein targeting to lysosome involved in chaperone-mediated autophagy [GO:0061740]; RNA splicing [GO:0008380]; synaptic vesicle uncoating [GO:0016191]
1203.97	326.5	2425.92	379.81	1514.1	1105.54	118.17	450.14	279.79	707.33	671.91	1286.29	711.96	310.47	666.33	318.23	1627.54	383.95	14487.95	Guanine nucleotide exchange factor VAV2 (VAV-2)	Vav2	angiogenesis [GO:0001525]; cell migration [GO:0016477]; cell projection assembly [GO:0030031]; lamellipodium assembly [GO:0030032]; positive regulation of phosphatidylinositol 3-kinase/protein kinase B signal transduction [GO:0051897]; regulation of cell size [GO:0008361]; small GTPase-mediated signal transduction [GO:0007264]

155.06	532.82	887.78	134.21	372.56	776.62	21.41	224.7	2246.02	114.77	220.78	1044.84	76.18	969.16	2614.88	262.32	667.85	3161.64	14483.6	Probable E3 ubiquitin-protein ligase HERC1 (EC 2.3.2.26) (HECT domain and RCC1-like domain-containing protein 1) (HECT-type E3 ubiquitin transferase HERC1) (p532) (p619)	HERC1	autophagy [GO:0006914]; bone mineralization [GO:0030282]; bone remodeling [GO:0046849]; cerebellar Purkinje cell differentiation [GO:0021702]; corpus callosum development [GO:0022038]; gene expression [GO:0010467]; negative regulation of autophagy [GO:0010507]; neuromuscular process controlling balance [GO:0050885]; neuron projection development [GO:0031175]; protein ubiquitination [GO:0016567]
1071.93	765.15	942.39	427.04	1070.64	626.14	7.57	344.59	2030.94	321.43	275.65	1028.48	349.07	562.08	1713.18	234.35	943.76	1732.4	14446.79	Inhibitor of nuclear factor kappa-B kinase subunit alpha (I kappa-B kinase alpha) (IKK-A) (IKK-alpha) (IkBKA) (IkappaB kinase) (EC 2.7.11.10) (Conserved helix-loop-helix ubiquitous kinase) (I-kappa-B kinase 1) (IKK1) (Nuclear factor NF-kappa-B inhibitor kinase alpha) (NFKBIKA)	chuk ikk1 zgc:56539	cellular response to tumor necrosis factor [GO:0071356]; central nervous system development [GO:0007417]; epiboly involved in gastrulation with mouth forming second [GO:0055113]; negative regulation of apoptotic process [GO:0043066]; negative regulation of canonical NF-kappaB signal transduction [GO:0043124]; positive regulation of canonical NF-kappaB signal transduction [GO:0043123]; positive regulation of transcription by RNA polymerase II [GO:0045944]; somite specification [GO:0001757]; tumor necrosis factor-mediated signaling pathway [GO:0033209]
20.66	21.15	1011.79	36.13	7.46	22.46	4639.47	18.69	2064.52	23.81	140.23	531.71	11.72	38.68	1194.01	2942.57	105.14	1599.68	14429.88	RNA-binding protein NOB1 (EC 3.1.-.-)	NOB1	maturation of SSU-rRNA [GO:0030490]

814.08		1373.7	821.81	265.84	908.47	1296.52	110.39	523.29	274.03	297.96	582.66	710.65	929.14	2055.58	780.62	515.02	1339.37	824.05	14423.18	Collagen alpha-1(XXVII) chain B	col27a1b col27a1	bone mineralization [GO:0030282]; notochord morphogenesis [GO:0048570]; skeletal system development [GO:0001501]
959.85		763.37	795.87	351.2	638.38	1255.18	2344.39	418.02	480.71	364.62	322.01	598.94	913.54	783.45	630.69	1790.71	422.5	568.9	14402.33	TBC1 domain family member 1	TBC1D1 KIAA1108	
250.24		758.99	245.61	599.92	1774.51	1628.47	10.97	627.5	1097.26	1565.95	1078.97	531.41	508.71	1297.28	968.54	197.8	622.3	637.44	14401.87	Anoctamin-7 (New gene expressed in prostate homolog) (Transmembrane protein 16G)	Ano7 Ngep Tmem16g	calcium activated galactosylceramide scrambling [GO:0061591]; calcium activated phosphatidylcholine scrambling [GO:0061590]; calcium activated phosphatidylserine scrambling [GO:0061589]; chloride transport [GO:0006821]; establishment of localization in cell [GO:0051649]

586.62	749.4	719.17	257.81	685.87	981.74	3249.47	286.18	522.02	282.72	273.43	620.11	608.99	616.52	606.16	2182.97	440.25	719.01	14388.44	Delta-1-pyrroline-5-carboxylate synthase (P5CS) (Aldehyde dehydrogenase family 18 member A1) [Includes: Glutamate 5-kinase (GK) (EC 2.7.2.11) (Gamma-glutamyl kinase);Gamma-glutamyl phosphate reductase (GPR) (EC 1.2.1.41) (Glutamate-5-semialdehyde dehydrogenase) (Glutamyl-gamma-semialdehyde dehydrogenase)]	ALDH18A1	citrulline biosynthetic process [GO:0019240]; glutamate metabolic process [GO:0006536]; L-proline biosynthetic process [GO:0055129]; ornithine biosynthetic process [GO:0006592]; proline biosynthetic process [GO:0006561]
986.04	1190.91	567.77	225.33	1114.33	666.8	208.84	251.59	186.84	223.72	738.12	429.4	2638.26	1757.34	587.27	1057.4	1180.02	367.37	14377.35	Very-long-chain 3-oxoacyl-CoA reductase-A (EC 1.1.1.330) (17-beta-hydroxysteroid dehydrogenase 12-A) (17-beta-HSD 12-A) (zf3.1) (zfHSD17B12A) (3-ketoacyl-CoA reductase) (KAR) (Estradiol 17-beta-dehydrogenase 12-A) (EC 1.1.1.62)	hsd17b12a zgc:55589	estrogen biosynthetic process [GO:0006703]; fatty acid biosynthetic process [GO:0006633]
1299.37	1722.96	1468.92	256.75	940.87	1019.7	46.75	779.45	848.26	406.31	505.57	1497.79	559.75	921.86	278.17	155.24	1364.96	304.64	14377.32	PI-PLC X domain-containing protein 1	PLCXD1	lipid metabolic process [GO:0006629]

447.15	754.9	133.24	830.32	344.37	728.11	4979.7	675.28	209.43	921.23	533.8	388.06	366.5	259.99	155.33	2236.61	276.67	136.19	14376.88	Mucosa-associated lymphoid tissue lymphoma translocation protein 1 (EC 3.4.22.-) (MALT lymphoma-associated translocation) (Paracaspase)	MALT1 MLT	B cell activation [GO:0042113]; B-1 B cell differentiation [GO:0001923]; defense response [GO:0006952]; innate immune response [GO:0045087]; lipopolysaccharide-mediated signaling pathway [GO:0031663]; negative regulation of apoptotic process [GO:0043066]; nuclear export [GO:0051168]; positive regulation of canonical NF-kappaB signal transduction [GO:0043123]; positive regulation of interleukin-1 beta production [GO:0032731]; positive regulation of interleukin-2 production [GO:0032743]; positive regulation of protein ubiquitination [GO:0031398]; positive regulation of T cell cytokine production [GO:0002726]; positive regulation of T-helper 17 cell differentiation [GO:2000321]; proteolysis [GO:0006508]; proteolysis involved in protein catabolic process [GO:0051603]; regulation of apoptotic process [GO:0042981]; regulation of T cell receptor signaling pathway [GO:0050856]; response to fungus
3084.74	238.11	179.55	775.68	267.21	253.17	6.55	115.24	30.7	870.93	201.01	109.49	6516.93	617.62	334.04	245.8	406.76	116.25	14369.78	Neural cell adhesion molecule L1-like protein (Close homolog of L1) [Cleaved into: Processed neural cell adhesion molecule L1-like protein]	CHL1 CALL	adult locomotory behavior [GO:0008344]; axon guidance [GO:0007411]; cell adhesion [GO:0007155]; cognition [GO:0050890]; exploration behavior [GO:0035640]; negative regulation of neuron apoptotic process [GO:0043524]; neuron migration [GO:0001764]; signal transduction [GO:0007165]

990.64	794.69	1150.78	289.8	822.28	1014.21	162.91	538.66	1016.54	326.35	392.62	866.4	608.7	1336.05	1278.71	225.76	850.35	1696.83	14362.28	Spliceosome associated factor 3, U4/U6 recycling protein (Squamous cell carcinoma antigen recognized by T-cells 3) (SART-3)	sart3 egy si:ch211-191d15.4 wu:fc51h03	exocrine pancreas development [GO:0031017]; hematopoietic stem cell homeostasis [GO:0061484]; lymphocyte differentiation [GO:0030098]; mRNA splicing, via spliceosome [GO:0000398]; nucleosome assembly [GO:0006334]; regulation of hematopoietic stem cell proliferation [GO:1902033]; regulation of intrinsic apoptotic signaling pathway by p53 class mediator [GO:1902253]; spliceosomal complex assembly [GO:0000245]; spliceosomal snRNP assembly [GO:0000387]; thymus development [GO:0048538]; transcription elongation-coupled chromatin remodeling [GO:0140673]
1289.95	637.21	2053.31	465.17	791.78	2029.69	166.78	248.88	2196.44	448.63	254.13	1241.1	469.99	137.22	715.73	269.68	90.33	834.87	14340.89	Dihydrolipoyl dehydrogenase, mitochondrial (EC 1.8.1.4) (Dihydrolipoamide dehydrogenase)	DLD	2-oxoglutarate metabolic process [GO:0006103]; branched-chain amino acid catabolic process [GO:0009083]; gastrulation [GO:0007369]; mitochondrial electron transport, NADH to ubiquinone [GO:0006120]; proteolysis [GO:0006508]; pyruvate metabolic process [GO:0006090]; regulation of membrane potential [GO:0042391]; sperm capacitation [GO:0048240]

29.59	43.08	531.63	5.44	56.05	38.67	1.76	27.6	3647.75	3.67	12.58	1399.3	9.6	9.87	3984.53	2.09	28.53	4497.64	14329.38	Delta-like protein 4 (Drosophila Delta homolog 4) (Delta4)	DLL4 UNQ1895/PRO4341	angiogenesis [GO:0001525]; aortic valve morphogenesis [GO:0003180]; blood vessel lumenization [GO:0072554]; blood vessel remodeling [GO:0001974]; branching involved in blood vessel morphogenesis [GO:0001569]; cardiac atrium morphogenesis [GO:0003209]; cardiac ventricle morphogenesis [GO:0003208]; cellular response to fibroblast growth factor stimulus [GO:0044344]; cellular response to vascular endothelial growth factor stimulus [GO:0035924]; dorsal aorta morphogenesis [GO:0035912]; negative regulation of blood vessel endothelial cell proliferation involved in sprouting angiogenesis [GO:1903588]; negative regulation of cell migration involved in sprouting angiogenesis [GO:0090051]; negative regulation of cell population proliferation [GO:0008285]; negative regulation of endothelial cell migration [GO:0010596]; negative regulation of gene expression [GO:0010629]; negative regulation of Notch signaling pathway [GO:0045746]; negative regulation of
698.79	550.29	777.69	340.95	499.36	719.54	3868.65	317.37	365.46	194.55	230.79	452.43	672.47	723.57	496.42	2465.39	426.99	496.36	14297.07	GDNF family receptor alpha-2 (GDNF receptor alpha-2) (GDNFR-alpha-2) (GFR-alpha-2)	GFRA2	glial cell-derived neurotrophic factor receptor signaling pathway [GO:0035860]; nervous system development [GO:0007399]
260.39	634.98	2882.95	494.4	806.84	543.82	467.52	433.94	1207.43	196.06	350.22	1751.13	235.71	752	867.12	671.34	554.05	1104.17	14214.07	Snaclec 7 (C-type lectin-like 7)		

722.48	1675.32	857.86	201.92	1447.97	890.53	687.51	947.97	605.32	216.66	363.09	549.34	920.5	1125.22	785.37	530.74	944.81	734.99	14207.6	Serine/threonine-protein kinase PAK 5 (EC 2.7.11.1) (p21-activated kinase 5) (PAK-5) (p21-activated kinase 7) (PAK-7)	Pak5 Pak7	apoptotic process [GO:0006915]; cytoskeleton organization [GO:0007010]; learning [GO:0007612]; locomotory behavior [GO:0007626]; memory [GO:0007613]; negative regulation of extrinsic apoptotic signaling pathway [GO:2001237]
504.98	415.09	449.96	316.29	420.93	485.24	4039.39	152.32	271.79	202.71	305.24	360.87	1114.1	366.18	535.58	3294.59	421.38	526.02	14182.66	Sulfhydryl oxidase 2 (EC 1.8.3.2) (Neuroblastoma-derived sulfhydryl oxidase) (Quiescin Q6-like protein 1)	QSOX2 QSCN6L1 SOXN	protein folding [GO:0006457]
497.31	772.83	1120.09	260.82	852.37	871.14	1843.4	363.64	1024.16	233.25	324.42	923.78	712.39	997.39	930.07	763.39	597.8	1089.28	14177.53	Zinc finger protein 366 (Dendritic cell-specific transcript protein) (DC-SCRIPT)	ZNF366	negative regulation of intracellular estrogen receptor signaling pathway [GO:0033147]; negative regulation of transcription by RNA polymerase II [GO:0000122]; response to estrogen [GO:0043627]

722.29		1075.75	1198.23	321.16	634.4	1470.12	98.02	337.26	974.54	318.83	245.95	906.13	797.65	1538.75	1120.17	319.65	708.39	1381.59	14168.88	Indoleamine 2,3-dioxygenase 2 (IDO-2) (EC 1.13.11.-) (Indoleamine 2,3-dioxygenase-like protein 1) (Indoleamine-pyrrole 2,3-dioxygenase-like protein 1)	IDO2 INDOL1	'de novo' NAD biosynthetic process from L-tryptophan [GO:0034354]; immune system process [GO:0002376]; L-tryptophan catabolic process to kynurenine [GO:0019441]
386.93		493.45	1240.7	178.31	566.14	984.14	4.28	306.97	1675.16	212.25	205.6	949.24	619.53	1168.45	2480.43	262.37	449.61	1958.11	14141.67	Propionyl-CoA carboxylase beta chain, mitochondrial (PCCase subunit beta) (EC 6.4.1.3) (Propanoyl-CoA:carbon dioxide ligase subunit beta)	Pccb	
82.82		3430.48	0	176.49	3225.35	16.76	0	774.17	0	76.06	1045.15	5.9	43.63	3715.19	0	222.62	1306.81	6.06	14127.49	Pleckstrin homology-like domain family B member 1 (Protein LL5-alpha)	Phldb1 Ll5	positive regulation of basement membrane assembly involved in embryonic body morphogenesis [GO:1904261]; regulation of epithelial to mesenchymal transition [GO:0010717]; regulation of gastrulation [GO:0010470]; regulation of microtubule cytoskeleton organization [GO:0070507]

874.97	608.69	2381.27	418.33	738.99	1472.05	645.42	281.92	804.66	371.78	280.11	1169.27	763.51	658.93	684.11	653.79	500.61	807.41	14115.82	Y+L amino acid transporter 2 (Solute carrier family 7 member 6) (zfSlc7a6) (y(+)L-type amino acid transporter 2) (Y+LAT2) (y+LAT-2)	slc7a6	amino acid transmembrane transport [GO:0003333]; glycine betaine transport [GO:0031460]; L-arginine transmembrane transport [GO:1903826]; neutral amino acid transport [GO:0015804]; nitric oxide biosynthetic process [GO:0006809]
36.07	0	14.2	113.2	9.73	6.82	7989.25	6.3	9.66	81.96	206.97	0	37.37	1.18	10.03	5378.08	171.21	20.62	14092.65	Stonustoxin subunit beta (SNTX subunit beta) (DELTA-synanceitoxin-Sh1b) (DELTA-SYTX-Sh1b) (Trachynilysin subunit beta) (TLY subunit beta)		killing of cells of another organism [GO:0031640]
1019.24	874.67	1162.62	391.24	901.45	1399.99	530.24	487.49	761.68	354.49	356.96	918.68	506.96	1294.43	861.01	646.39	776.84	847.05	14091.43	Zinc finger protein AEBP2 (Adipocyte enhancer-binding protein 2 homolog) (AE-binding protein 2 homolog)	aebp2 si:dkey-158p11.2 zgc:63755	chromatin organization [GO:0006325]; regulation of transcription by RNA polymerase II [GO:0006357]

9.14	1.14	437.34	5.1	4.55	19.46	0	5.27	2968.55	4.55	2.38	874.52	4.25	17.35	5096.22	1.61	0.7	4624.6	14076.73	Unconventional myosin-Ic (Myosin I beta) (MMI-beta) (MMIb)	MYO1C RCJMB04_6o17	actin filament organization [GO:0007015]; actin filament-based movement [GO:0030048]; endocytosis [GO:0006897]
440.04	186.07	5628.69	81.34	76.56	472.66	3.76	60.62	984.65	111.5	34.31	3222.34	83.74	292.68	827.16	15.53	49.9	1418.78	13990.33	A-kinase anchor protein 13 (AKAP-13) (AKAP-Lbc) (Breast cancer nuclear receptor-binding auxiliary protein) (Guanine nucleotide exchange factor Lbc) (Human thyroid-anchoring protein 31) (Lymphoid blast crisis oncogene) (LBC oncogene) (Non-oncogenic Rho GTPase-specific GTP exchange factor) (Protein kinase A-anchoring protein 13) (PRKA13) (p47)	AKAP13 BRX HT31 LBC	adrenergic receptor signaling pathway [GO:0071875]; bone development [GO:0060348]; cardiac muscle cell differentiation [GO:0055007]; G protein-coupled receptor signaling pathway [GO:0007186]; heart development [GO:0007507]; positive regulation of canonical NF-kappaB signal transduction [GO:0043123]; positive regulation of Rho protein signal transduction [GO:0035025]; regulation of Rho protein signal transduction [GO:0035023]; regulation of sarcomere organization [GO:0060297]; regulation of small GTPase mediated signal transduction [GO:0051056]
675.58	951.65	476.41	147.08	774.64	457.35	865.02	359.38	602.22	94.71	301.78	329.66	1529.27	2206.12	921.99	1340.14	1135.78	777.15	13945.93	Calmodulin-lysine N-methyltransferase (CLNMT) (CaM KMT) (EC 2.1.1.60)	Camkmt Clnmt	methylation [GO:0032259]; mitochondrion organization [GO:0007005]
2048.89	867.85	238.06	293.73	1568.2	244.25	110.77	416.57	46.45	355.56	293.89	122.62	2623.26	2196.98	287.08	719.74	1311.5	199.87	13945.27	Casein kinase I isoform gamma-2 (CKI-gamma 2) (EC 2.7.11.1)	Csnk1g2 Ck1g2	Wnt signaling pathway [GO:0016055]
416.61	616.97	464.09	219.46	550.35	833.49	3618.05	495.5	533.99	172.78	151.03	427.85	840.83	813.32	410.05	1838.49	669.98	853.37	13926.21	Uncharacterized oxidoreductase YtbE (EC 1.-.-.-)	ytbE BSU29050	

1500.77	113.75	2537.09	146.62	110.38	2186.96	4.03	21.14	58.18	303.57	23.34	2673.44	3314.78	52.36	553.71	79.28	17.58	200.87	13897.85	Alpha-soluble NSF attachment protein (SNAP-alpha) (N-ethylmaleimide-sensitive factor attachment protein alpha)	NAPA SNAPA	apical protein localization [GO:0045176]; brain development [GO:0007420]; intra-Golgi vesicle-mediated transport [GO:0006891]; intracellular protein transport [GO:0006886]; membrane fusion [GO:0061025]; neuron differentiation [GO:0030182]; regulation of synaptic vesicle priming [GO:0010807]; SNARE complex disassembly [GO:0035494]; synaptic transmission, glutamatergic [GO:0035249]; synaptic vesicle priming [GO:0016082]
13.62	18.08	3139.91	36.02	33.68	569.69	1011.8	31.35	245.56	37.47	90.75	5902.85	29	22.15	188.27	2332.07	62.45	99.88	13864.6	Ubiquitin thioesterase otulin (EC 3.4.19.12) (Deubiquitinating enzyme otulin) (OTU domain-containing deubiquitinase with linear linkage specificity) (Ubiquitin thioesterase Gumby)	Otulin Fam105b Gum	innate immune response [GO:0045087]; negative regulation of inflammatory response [GO:0050728]; negative regulation of NF-kappaB transcription factor activity [GO:0032088]; nucleotide-binding oligomerization domain containing 2 signaling pathway [GO:0070431]; protein linear deubiquitination [GO:1990108]; proteolysis [GO:0006508]; regulation of canonical Wnt signaling pathway [GO:0060828]; regulation of tumor necrosis factor-mediated signaling pathway [GO:0010803]; sprouting angiogenesis [GO:0002040]; Wnt signaling pathway [GO:0016055]

1025.04	952.55	895.93	336.2	1032.65	984.01	205.85	530.93	816.37	386.66	437.9	760.08	826.86	1364.06	1112.93	324.31	794.81	1051.64	13838.78	ATP-binding cassette sub-family G member 4 (EC 7.6.2.-)	ABCG4 WHITE2	cellular response to high density lipoprotein particle stimulus [GO:0071403]; cellular response to leukemia inhibitory factor [GO:1990830]; cholesterol efflux [GO:0033344]; cholesterol homeostasis [GO:0042632]; positive regulation of cholesterol biosynthetic process [GO:0045542]; positive regulation of cholesterol efflux [GO:0010875]; regulation of DNA-templated transcription [GO:0006355]; transmembrane transport [GO:0055085]
881.85	632.73	3950.44	399.25	999.38	531.59	6.51	196.09	1011.53	371.41	262.54	896.14	2266.21	313.61	303.81	138.41	314.06	361.38	13836.94	Mitochondrial glutathione transporter SLC25A40 (Solute carrier family 25 member 40)	slc25a40 zgc:92520	import into the mitochondrion [GO:0170036]
30.03	4092.85	190.74	29.09	3064.11	288.34	4.08	1589.7	13.7	20.1	495.66	104.47	23.09	2295.45	92.55	60.82	1359.26	80.16	13834.2	DCC-interacting protein 13-alpha (Dip13-alpha) (Adapter protein containing PH domain, PTB domain and leucine zipper motif 1)	Appl1 Dip13a Kiaa1428	adiponectin-activated signaling pathway [GO:0033211]; cellular response to hepatocyte growth factor stimulus [GO:0035729]; insulin receptor signaling pathway [GO:0008286]; maintenance of synapse structure [GO:0099558]; negative regulation of Fc-gamma receptor signaling pathway involved in phagocytosis [GO:1905450]; positive regulation of cytokine production involved in inflammatory response [GO:1900017]; positive regulation of D-glucose import [GO:0046326]; positive regulation of macropinocytosis [GO:1905303]; positive regulation of melanin biosynthetic process [GO:0048023]; protein import into nucleus [GO:0006606]; regulation of fibroblast migration [GO:0010762]; regulation of G1/S transition of mitotic cell cycle [GO:2000045]; regulation of innate immune response [GO:0045088]; regulation of protein localization to plasma membrane [GO:1903076]; regulation of toll-like receptor 4 [illegible]

497.11	974.72	979.99	215.67	1023.89	933.91	234.19	374.39	1120.43	155.48	326.67	1040.28	417.64	1865.12	1428.29	342.61	621.45	1270.66	13822.5	Phosphomevalonate kinase (PMKase) (EC 2.7.4.2)	PMVK	cholesterol biosynthetic process [GO:0006695]; isopentenyl diphosphate biosynthetic process, mevalonate pathway [GO:0019287]; response to cholesterol [GO:0070723]
511.07	428.16	1618.87	303.3	812.78	998.61	43.96	206.73	1021.4	187.41	484.27	2872.91	530.47	602.02	888.69	260.7	955.5	1077.78	13804.63	Low-density lipoprotein receptor (LDL receptor)	Ldlr	amyloid-beta clearance [GO:0097242]; amyloid-beta clearance by cellular catabolic process [GO:0150094]; artery morphogenesis [GO:0048844]; cellular response to fatty acid [GO:0071398]; cellular response to low-density lipoprotein particle stimulus [GO:0071404]; cholesterol homeostasis [GO:0042632]; cholesterol import [GO:0070508]; cholesterol metabolic process [GO:0008203]; cholesterol transport [GO:0030301]; endocytosis [GO:0006897]; establishment of localization in cell [GO:0051649]; high-density lipoprotein particle clearance [GO:0034384]; intestinal cholesterol absorption [GO:0030299]; lipid metabolic process [GO:0006629]; lipoprotein catabolic process [GO:0042159]; lipoprotein metabolic process [GO:0042157]; long-term memory [GO:0007616]; low-density lipoprotein particle clearance [GO:0034383]; negative regulation of amyloid fibril formation [GO:1905907]; negative regulation of astrocyte [illegible]

679.1		589.93	791.14	271.3	738.52	971.65	1662.3	245.2	939.17	260.13	220.31	797.47	892.08	604.19	1073.55	1263.39	429.46	1354.04	13782.93	Transcription factor AP-2 gamma (AP2-gamma) (Activating enhancer-binding protein 2 gamma) (Transcription factor ERF-1)	TFAP2C	cell-cell signaling [GO:0007267]; cerebral cortex development [GO:0021987]; dichotomous subdivision of terminal units involved in mammary gland duct morphogenesis [GO:0060598]; epithelial cell proliferation involved in mammary gland duct elongation [GO:0060750]; forebrain neuron fate commitment [GO:0021877]; germ-line stem cell population maintenance [GO:0030718]; hair follicle development [GO:0001942]; inner cell mass cell differentiation [GO:0001826]; keratinocyte development [GO:0003334]; male gonad development [GO:0008584]; morula formation [GO:0140001]; negative regulation of gene expression, epigenetic [GO:0045814]; positive regulation of transcription by RNA polymerase II [GO:0045944]; regulation of cell population proliferation [GO:0042127]; regulation of epidermis development [GO:0045682]; regulation of transcription by RNA polymerase II [GO:0006357]; sebaceous gland [illegible]
876.25		602.77	806.99	320.49	568.33	1259.67	1805.66	347.76	288.56	331.29	316.33	495.69	1412.97	791.79	673.64	1878.14	535.26	465.14	13776.73	Glutamate receptor 3 (GluR-3) (AMPA-selective glutamate receptor 3) (GluR-C) (GluR-K3) (Glutamate receptor ionotropic, AMPA 3)	Gria3 GluA3 Glur3	long-term synaptic potentiation [GO:0060291]; modulation of chemical synaptic transmission [GO:0050804]; protein heterotetramerization [GO:0051290]; protein homotetramerization [GO:0051289]; regulation of receptor recycling [GO:0001919]; response to fungicide [GO:0060992]; response to lithium ion [GO:0010226]; synaptic transmission, glutamatergic [GO:0035249]

737.93	550.26	675.28	254.58	624.19	987.86	2187.33	248.59	809.4	198.04	211.13	563.78	1063.71	847.57	988.37	1378.08	446.51	1000.63	13773.24	Tyrosine-protein kinase Blk (EC 2.7.10.2) (B lymphocyte kinase) (p55-Blk)	BLK	B cell receptor signaling pathway [GO:0050853]; cell differentiation [GO:0030154]; cell surface receptor protein tyrosine kinase signaling pathway [GO:0007169]; intracellular signal transduction [GO:0035556]; peptidyl-tyrosine phosphorylation [GO:0018108]; positive regulation of insulin secretion [GO:0032024]
192.77	2286.72	100.94	98.59	1733.51	14.51	0	791.62	12.11	62.27	868.87	47.43	498.27	4193.86	148.33	392.71	2056.41	267.28	13766.2	NACHT, LRR and PYD domains-containing protein 12 (Monarch-1) (PYRIN-containing APAF1-like protein 7) (Regulated by nitric oxide)	NLRP12 NALP12 PYPAF7 RNO	cellular response to cytokine stimulus [GO:0071345]; dendritic cell migration [GO:0036336]; ERK1 and ERK2 cascade [GO:0070371]; negative regulation of canonical NF-kappaB signal transduction [GO:0043124]; negative regulation of cytokine production [GO:0001818]; negative regulation of ERK1 and ERK2 cascade [GO:0070373]; negative regulation of inflammatory response [GO:0050728]; negative regulation of interleukin-1 production [GO:0032692]; negative regulation of interleukin-6 production [GO:0032715]; negative regulation of non-canonical NF-kappaB signal transduction [GO:1901223]; negative regulation of signal transduction [GO:0009968]; negative regulation of Toll signaling pathway [GO:0045751]; positive regulation of inflammatory response [GO:0050729]; positive regulation of interleukin-1 beta production [GO:0032731]; positive regulation of MHC class I biosynthetic process [GO:0045345]; positive regulation of non-
2233.51	370.67	661.11	872.28	831.12	830.74	31.55	235.07	434.25	1006.49	209.9	2641.46	1040.53	206.98	321.98	211.17	590.1	1036	13764.91	Guanine nucleotide-binding protein G(s) subunit alpha (Adenylate cyclase-stimulating G alpha protein)	gnas	adenylate cyclase-activating dopamine receptor signaling pathway [GO:0007191]; sensory perception of chemical stimulus [GO:0007606]

3835.83	26.49	15.63	749	66.24	282.82	39.14	4.42	0	2965.2	43.1	119.17	5198.09	15.46	46.49	325.86	25.32	0.73	13758.99	COMM domain-containing protein 5 (Hypertension-related calcium-regulated gene protein) (HCaRG)	COMMD5 HT002	
782.84	984.42	1362.22	290.44	943.9	1225.78	501.31	321.96	707.39	234.87	289.97	1051.91	977.52	1243.08	800.24	464.24	812.78	763.91	13758.78	Fibrinogen alpha chain [Cleaved into: Fibrinopeptide A;Fibrinogen alpha chain]	FGA	blood coagulation, common pathway [GO:0072377]; fibrinolysis [GO:0042730]; platelet aggregation [GO:0070527]; positive regulation of heterotypic cell-cell adhesion [GO:0034116]; protein polymerization [GO:0051258]
517.72	1099.29	593.8	278.76	701.57	225.45	1.16	528.29	177.53	308.93	287.8	296.16	198.21	6980.26	240.38	48.61	954.76	319.29	13757.97	Zinc finger protein Pegasus (Ikaros family zinc finger protein 5)	ikzf5 zgc:92405	determination of intestine left/right asymmetry [GO:0071908]; determination of left/right asymmetry in lateral mesoderm [GO:0003140]; determination of left/right symmetry [GO:0007368]; determination of pancreatic left/right asymmetry [GO:0035469]; regulation of transcription by RNA polymerase II [GO:0006357]
127.69	1372.53	201.75	395.68	1202.88	125.56	496.69	603.07	164.11	94.74	381.36	241.76	195.79	4536.9	242	758.03	2248.99	358.89	13748.42	Myeloid-associated differentiation marker homolog	myadm	

136.02	31.29	83.76	284.26	28.67	132.05	8444.35	5.59	21.04	96.12	137.9	57.54	608.31	36.1	48.54	3366.89	180.09	37.21	13735.73	5'-AMP-activated protein kinase subunit gamma-2 (AMPK gamma2) (AMPK subunit gamma-2) (H91620p)	PRKAG2	ATP biosynthetic process [GO:0006754]; cellular response to glucose starvation [GO:0042149]; cellular response to nutrient levels [GO:0031669]; fatty acid biosynthetic process [GO:0006633]; glycogen metabolic process [GO:0005977]; intracellular signal transduction [GO:0035556]; positive regulation of gluconeogenesis [GO:0045722]; regulation of carbon utilization [GO:0043609]; regulation of cell cycle [GO:0051726]; regulation of D-glucose import [GO:0046324]; regulation of fatty acid metabolic process [GO:0019217]; regulation of fatty acid oxidation [GO:0046320]; regulation of glycolytic process [GO:0006110]; sterol biosynthetic process [GO:0016126]
232.49	88.5	2870.56	73.12	41.02	1364.55	12.1	9.34	2889.79	72.13	75.84	1319.96	312.38	341.76	1404.8	132.74	517.49	1921.86	13680.43	Occludin	Ocln	bicellular tight junction assembly [GO:0070830]; cell-cell junction organization [GO:0045216]; cellular response to tumor necrosis factor [GO:0071356]; negative regulation of gene expression [GO:0010629]; positive regulation of blood-brain barrier permeability [GO:1905605]; positive regulation of D-glucose import [GO:0046326]; positive regulation of gene expression [GO:0010628]; positive regulation of lamellipodium assembly [GO:0010592]; positive regulation of microtubule polymerization [GO:0031116]; positive regulation of wound healing [GO:0090303]; regulation of D-glucose transmembrane transport [GO:0010827]; response to ethanol [GO:0045471]; response to Gram-positive bacterium [GO:0140459]; response to interleukin-18 [GO:0070673]; tight junction organization [GO:0120193]
998.48	921.05	932.87	267.79	827.77	950.3	670.23	555.47	891.38	320.88	331.75	713.01	800.86	1245.55	910.64	518.49	728.49	1068.24	13653.25	Protein PRRC2A (HLA-B-associated transcript 2) (Large proline-rich protein BAT2) (Proline-rich and coiled-coil-containing protein 2A) (Protein G2)	PRRC2A BAT2 G2	cell differentiation [GO:0030154]

1132	619.92	1507.86	472.83	1100.16	1719.02	4.97	425.93	166.35	513.07	441.08	1212.74	1340.77	1287.14	613.43	210.23	532.01	310.4	13609.91	G protein-coupled receptor 137Ba	gpr137ba	autophagy [GO:0006914]; bone remodeling [GO:0046849]; negative regulation of bone resorption [GO:0045779]; negative regulation of osteoclast differentiation [GO:0045671]; positive regulation of protein localization to lysosome [GO:0150032]; positive regulation of TORC1 signaling [GO:1904263]; regulation of autophagy [GO:0010506]; regulation of GTPase activity [GO:0043087]
1059.86	599.22	551.52	470.85	750.83	909.77	1361.94	615.54	385.72	446.76	452.51	469.01	985.68	1189.2	560.76	1186.5	1109.64	500.4	13605.71	Host cell factor 2 (HCF-2) (C2 factor)	Hcfc2	antiviral innate immune response [GO:0140374]; immune response involved in response to exogenous dsRNA [GO:1902615]; negative regulation of transcription by RNA polymerase II [GO:0000122]; regulation of toll-like receptor 3 signaling pathway [GO:0034139]; regulation of transcription by RNA polymerase II [GO:0006357]

884.89		619.48	808.63	206.65	1438.65	1768.29	6.58	344.47	355.2	371.62	638.64	884.42	638.56	1019.88	202.94	461.17	2041.98	912	13604.05	Alpha-internexin (Alpha-Inx) (66 kDa neurofilament protein) (NF-66) (Neurofilament-66) (Neurofilament 5)	INA NEF5	cell differentiation [GO:0030154]; cellular response to leukemia inhibitory factor [GO:1990830]; intermediate filament organization [GO:0045109]; neurofilament cytoskeleton organization [GO:0060052]; postsynaptic modulation of chemical synaptic transmission [GO:0099170]; substantia nigra development [GO:0021762]
985.87		974.38	865.16	270.48	722.55	982.92	824.76	376.08	742.04	244.1	272.62	830.18	896.88	1319.15	858.44	884.13	662.01	884.57	13596.32	Insulin-like growth factor 1 (Insulin-like growth factor I) (IGF-I) (Somatomedin)	igf1 igf-1	cell population proliferation [GO:0008283]; insulin-like growth factor receptor signaling pathway [GO:0048009]; negative regulation of apoptotic process [GO:0043066]; negative regulation of release of cytochrome c from mitochondria [GO:0090201]; positive regulation of cell population proliferation [GO:0008284]; positive regulation of phosphatidylinositol 3-kinase/protein kinase B signal transduction [GO:0051897]

234.22	570.68	2655.79	105.79	967.41	1246.64	925.94	202.94	666.99	70.28	494.96	928.8	222.64	1203.47	1110.89	727.51	747.39	513.9	13596.24	Gamma-aminobutyric acid receptor subunit rho-3 (GABA(A) receptor subunit rho-3) (GABAAR subunit rho-3) (GABA(C) receptor)	GABRR3	chemical synaptic transmission [GO:0007268]; chloride transmembrane transport [GO:1902476]; gamma-aminobutyric acid signaling pathway [GO:0007214]
746.96	763.57	1108.96	192.46	742.91	1127.31	907.51	356.71	669.07	349.7	333.23	939.59	824.01	1022.58	1178.87	596.08	577.58	1149.07	13586.17	Protein sidekick-2	SDK2 KIAA1514	camera-type eye photoreceptor cell differentiation [GO:0060219]; homophilic cell adhesion via plasma membrane adhesion molecules [GO:0007156]; retina layer formation [GO:0010842]; synapse assembly [GO:0007416]

991.45	746.72	850.09	548.08	616.69	2916.7	543.8	264.82	193.64	573.97	242.66	1772.54	1255.35	420.23	565.22	434.4	394.03	230.66	13561.05	SAGA-associated factor 29 (rSGF29) (Coiled-coil domain-containing protein 101) (SAGA complex-associated factor 29)	Sgf29 Ccdc101	negative regulation of transcription by RNA polymerase II [GO:0000122]; regulation of cell cycle [GO:0051726]; regulation of cell division [GO:0051302]; regulation of DNA repair [GO:0006282]; regulation of DNA-templated transcription [GO:0006355]; regulation of embryonic development [GO:0045995]; regulation of transcription by RNA polymerase II [GO:0006357]; response to endoplasmic reticulum stress [GO:0034976]; transcription initiation-coupled chromatin remodeling [GO:0045815]
449.87	612.57	816.55	206.85	632.77	1436.32	215.82	682.33	97.95	417	477.17	1171.57	1398.97	1730.1	1018.87	622.83	899.8	645.14	13532.48	Zinc finger protein 518A	ZNF518A KIAA0335 ZNF518	chromatin organization [GO:0006325]; regulation of transcription by RNA polymerase II [GO:0006357]
42.31	4920.04	146.06	163.22	2621.49	48.31	12.48	1428.32	5.13	47.32	533.6	64.28	18.74	2305.3	33.02	29.14	1091.37	19.51	13529.64	Proto-oncogene tyrosine-protein kinase Src (EC 2.7.10.2) (Proto-oncogene c-Src) (pp60c-src) (p60-Src)	src	cell adhesion [GO:0007155]; cell differentiation [GO:0030154]; epidermal growth factor receptor signaling pathway [GO:0007173]; negative regulation of extrinsic apoptotic signaling pathway [GO:2001237]; negative regulation of intrinsic apoptotic signaling pathway [GO:2001243]; progesterone receptor signaling pathway [GO:0050847]; regulation of vascular permeability [GO:0043114]; response to yeast [GO:0001878]

5.59	13.07	7118.66	16.91	12.62	60.72	47.74	7.68	1777.37	10.26	11.54	1011.76	38.46	22.4	1384.62	90.04	24.81	1862.87	13517.12	DnaJ homolog subfamily B member 6	dnajb6	chaperone-mediated protein folding [GO:0061077]
21.98	297.5	523.12	31.28	289.27	457.18	26.28	94.38	2326.08	21.78	126.23	1042.25	7.13	769.5	3366.21	185.32	364.29	3521.48	13471.26	Protein virilizer homolog	Virma Kiaa1429	mRNA processing [GO:0006397]; RNA splicing [GO:0008380]
604.78	615.89	969.8	254.18	501.31	552.36	2027.62	250.22	836.64	195.28	262.3	1179.41	761.71	855.05	737.05	1445.02	522.38	850.82	13421.82	Ankyrin repeat domain-containing protein 40	ANKRD40	
5.85	47.4	259.97	6.41	12.89	16.44	285.28	2.38	3840.05	5.63	9.53	1339.23	3.42	55.22	3815.05	53.52	33.16	3620.04	13411.47	Nucleus accumbens-associated protein 1 (NAC-1) (BTB/POZ domain-containing protein 14B)	Nacc1 Btbd14b Nac1	negative regulation of DNA-templated transcription [GO:0045892]; positive regulation of cell population proliferation [GO:0008284]
470.58	1103.63	1287.21	395.04	948.86	1012.83	41.72	738.66	479.77	386.38	637.3	2151.13	526.25	1066.95	871.49	102	557.75	626.07	13403.62	Protection of telomeres protein 1 (cPot1) (POT1-like telomere end-binding protein)	POT1	regulation of telomere maintenance via telomerase [GO:0032210]; telomere capping [GO:0016233]; telomere maintenance via telomerase [GO:0007004]

823.76	887.25	510.12	374.7	756.95	989.97	1760.06	377.16	552.43	306.05	375.17	539.98	1019.98	915.37	554.37	1311.63	706.88	641.36	13403.19	Hairy/enhancer-of-split related with YRPW motif protein 2 (Protein gridlock)	hey2 grl zgc:136746	artery development [GO:0060840]; artery morphogenesis [GO:0048844]; blood vessel development [GO:0001568]; blood vessel morphogenesis [GO:0048514]; cardiac muscle cell proliferation [GO:0060038]; circulatory system development [GO:0072359]; dorsal aorta morphogenesis [GO:0035912]; heart development [GO:0007507]; negative regulation of DNA-templated transcription [GO:0045892]; Notch signaling pathway [GO:0007219]; Notch signaling pathway involved in arterial endothelial cell fate commitment [GO:0060853]; regulation of neurogenesis [GO:0050767]; vasculogenesis [GO:0001570]
1398.97	759.47	765.18	298.95	683.35	1135.1	371.31	279.46	722.22	414.59	490.73	810.23	847.11	1479.81	1003.6	380.46	636.26	895.04	13371.84	Leucyl-cystinyl aminopeptidase (Cystinyl aminopeptidase) (EC 3.4.11.3) (Oxytocinase) (OTase)	Lnpep	negative regulation of cold-induced thermogenesis [GO:0120163]; protein catabolic process [GO:0030163]; proteolysis [GO:0006508]
1099.78	737.94	1237.39	390.36	673.77	1171.34	366.19	237.63	921.76	330.96	293.85	743.7	1117.35	892.47	1149.3	311.54	555.25	1134.71	13365.29	Transmembrane protein 266 (hTMEM266) (HV1-related protein 1) (HsHVRP1)	TMEM266 C15orf27 HVRP1	

699.37	542.95	878.44	246.07	707.72	1319.46	901.49	306.6	1041.62	303.07	290.85	818.73	746.05	817.63	1179.92	674.23	535.62	1322.38	13332.2	Anaphase-promoting complex subunit 1 (APC1) (Cyclosome subunit 1) (Mitotic checkpoint regulator) (Testis-specific gene 24 protein)	ANAPC1 TSG24	anaphase-promoting complex-dependent catabolic process [GO:0031145]; cell division [GO:0051301]; metaphase/anaphase transition of mitotic cell cycle [GO:0007091]; protein branched polyubiquitination [GO:0141198]; protein K11-linked ubiquitination [GO:0070979]; protein K48-linked ubiquitination [GO:0070936]; regulation of meiotic cell cycle [GO:0051445]; regulation of mitotic cell cycle [GO:0007346]
737.71	562.1	1566.08	301.94	470.86	821.13	1626.95	290.14	577.14	245.83	201.12	546.63	425.21	1039.82	674.25	1915.16	574.99	739.17	13316.23	Vacuolar protein sorting-associated protein 33B	vps33b	intracellular protein transport [GO:0006886]; intrahepatic bile duct development [GO:0035622]; vesicle-mediated transport [GO:0016192]
1069.59	736.67	836.51	423.8	934.17	787.03	568.05	227.22	125.55	260.01	240.33	616.67	1836.4	1908.29	511	1010.77	895.72	320.95	13308.73	Elongation factor 1-delta (EF-1-delta) (Antigen NY-CO-4)	EEF1D EF1D	cellular response to heat [GO:0034605]; cellular response to ionizing radiation [GO:0071479]; cytoplasmic translational elongation [GO:0002182]; positive regulation of transcription by RNA polymerase II [GO:0045944]; translational elongation [GO:0006414]

5.89	15.44	323.12	1.21	5.7	4.84	3.03	10.45	4399.66	0	4.18	1241.47	3.27	10.94	3274.48	3.88	22.53	3978	13308.09	Transcriptional repressor NF-X1 (EC 2.3.2.-) (Nuclear transcription factor, X box-binding protein 1)	NFX1 NFX2	inflammatory response [GO:0006954]; negative regulation of MHC class II biosynthetic process [GO:0045347]; negative regulation of transcription by RNA polymerase II [GO:0000122]; protein autoubiquitination [GO:0051865]; protein ubiquitination [GO:0016567]; transcription by RNA polymerase II [GO:0006366]
64.63	1.15	11.22	241.14	7.12	17.32	7472.62	1.25	38.89	79.67	183.36	5.5	164.49	6.84	41.79	4791.46	127.12	45.42	13300.99	Cerebral cavernous malformations protein 2 homolog (Malcavernin) (Valentine)	ccm2 vtn	anterior/posterior axis specification [GO:0009948]; cardiac jelly development [GO:1905072]; cell-cell junction assembly [GO:0007043]; heart contraction [GO:0060047]; heart development [GO:0007507]; vasculogenesis [GO:0001570]

313.14	2029.74	535.32	150.94	1459.82	663.74	106.45	769.08	760.26	121.81	505.66	483.87	478.25	1840.74	952.39	164.86	867.22	1094.31	13297.6	SWI/SNF-related matrix-associated actin-dependent regulator of chromatin subfamily A member 2 (SAMRCA2) (EC 3.6.4.-) (BRG1-associated factor 190B) (BAF190B) (Probable global transcription activator SNF2L2) (Protein brahma homolog) (SNF2-alpha)	Smarca2 Baf190b Brm Snf2a Snf2l2	aortic smooth muscle cell differentiation [GO:0035887]; cell population proliferation [GO:0008283]; chromatin organization [GO:0006325]; chromatin remodeling [GO:0006338]; fibroblast proliferation [GO:0048144]; hematopoietic stem cell homeostasis [GO:0061484]; negative regulation of cell differentiation [GO:0045596]; negative regulation of cell population proliferation [GO:0008285]; negative regulation of fibroblast proliferation [GO:0048147]; nervous system development [GO:0007399]; nucleosome assembly [GO:0006334]; positive regulation of cell differentiation [GO:0045597]; positive regulation of cell population proliferation [GO:0008284]; positive regulation of double-strand break repair [GO:2000781]; positive regulation of myoblast differentiation [GO:0045663]; positive regulation of stem cell population maintenance [GO:1902459]; positive regulation of T cell differentiation [GO:0045582]; regulation of G0 to G1 transition
635.08	497.91	753.34	348.46	360.55	981.34	2493.7	249.35	509.17	305.58	177.86	782.74	700.33	470.52	731.63	1900.31	362.27	1024.32	13284.46	Methionine-R-sulfoxide reductase B3, mitochondrial (MsrB3) (EC 1.8.4.12) (EC 1.8.4.14)	Msrb3	protein repair [GO:0030091]; response to oxidative stress [GO:0006979]
582.32	484.86	884.36	153.69	442.6	763.73	696.04	235.91	1800.14	218.6	209.21	803.87	738.3	679.95	1616.54	463.86	394.55	2109.11	13277.64	Endoribonuclease Dicer (EC 3.1.26.3)	dicer1	apoptotic DNA fragmentation [GO:0006309]; cranial skeletal system development [GO:1904888]; global gene silencing by mRNA cleavage [GO:0098795]; heterochromatin formation [GO:0031507]; melanocyte differentiation [GO:0030318]; miRNA processing [GO:0035196]; miRNA-mediated gene silencing by mRNA destabilization [GO:0035279]; miRNA-mediated post-transcriptional gene silencing [GO:0035195]; neural crest cell development [GO:0014032]; pre-miRNA processing [GO:0031054]; regulation of neurogenesis [GO:0050767]; siRNA processing [GO:0030422]; ventricular system development [GO:0021591]
492.23	1050.08	466.84	208.71	1474.54	990.93	259.18	1520.67	105.91	514.24	757.67	324.81	915.19	2105.12	220.78	344.69	1392.9	115.77	13260.26	PHD finger protein 11	PHF11	

1113.49	692.31	953.22	251.57	569.91	824.89	184.21	307.54	1617.61	325.27	245.62	733.8	659.43	913.59	1407.45	324.64	511.74	1614.92	13251.21	Protein mono-ADP-ribosyltransferase PARP9 (EC 2.4.2.-) (ADP-ribosyltransferase diphtheria toxin-like 9) (ARTD9) (B aggressive lymphoma protein homolog) (Poly [ADP-ribose] polymerase 9) (PARP-9)	Parp9 Bal	defense response to virus [GO:0051607]; DNA damage checkpoint signaling [GO:0000077]; double-strand break repair [GO:0006302]; innate immune response [GO:0045087]; negative regulation of gene expression [GO:0010629]; negative regulation of transcription by RNA polymerase II [GO:0000122]; positive regulation of defense response to virus by host [GO:0002230]; positive regulation of DNA-templated transcription [GO:0045893]; positive regulation of protein localization to nucleus [GO:1900182]; positive regulation of type II interferon-mediated signaling pathway [GO:0060335]; post-transcriptional regulation of gene expression [GO:0010608]; regulation of response to type II interferon [GO:0060330]
1104.91	781.55	1136.83	299.97	647.52	938.64	886.11	410.39	799.03	333.13	319.41	609.08	911.97	911.01	841.27	762.37	622.25	919.33	13234.77	La-related protein 4 (La ribonucleoprotein domain family member 4)	LARP4 PP13296	cytoskeleton organization [GO:0007010]; positive regulation of translation [GO:0045727]; post-transcriptional regulation of gene expression [GO:0010608]; regulation of cell morphogenesis [GO:0022604]; translation [GO:0006412]
462.28	905.43	1565.24	151.34	1230.59	1511.01	69.17	923.57	211.77	225.44	506.77	769.07	748.41	1999.09	562.56	142.02	476.81	757.91	13218.48	Bromodomain adjacent to zinc finger domain protein 2B (hWALp4)	BAZ2B KIAA1476	chromatin remodeling [GO:0006338]; regulation of transcription by RNA polymerase II [GO:0006357]

508.15		1080.31	958.84	511.7	1399.77	922.88	853.62	529.77	376.45	301.95	635.63	720.61	548.56	1343.07	358.16	746.67	895.14	520.62	13211.9	Neuroepithelial cell-transforming gene 1 protein (Rho guanine nucleotide exchange factor 8)	Net1 Arhgef8	cellular response to hydrogen peroxide [GO:0070301]; cellular response to ionizing radiation [GO:0071479]; intracellular signal transduction [GO:0035556]; myoblast migration [GO:0051451]; positive regulation of apoptotic process [GO:0043065]; positive regulation of GTPase activity [GO:0043547]; positive regulation of substrate adhesion-dependent cell spreading [GO:1900026]
400.54		503.15	857.12	299.32	642.42	1090.96	2004.15	247.05	563.85	212.98	277.9	863.91	821.14	847.85	707.5	1528.8	467.81	858.6	13195.05	Thyroid hormone receptor beta (TR-beta) (TRb) (Nuclear receptor subfamily 1 group A member 2) (Thyroid hormone receptor beta-1) (TRbeta1)	thrb nr1a2 trb si:ch211-264a6.2	camera-type eye development [GO:0043010]; cell differentiation [GO:0030154]; inner ear development [GO:0048839]; negative regulation of transcription by RNA polymerase II [GO:0000122]; positive regulation of transcription by RNA polymerase II [GO:0045944]; regulation of DNA-templated transcription [GO:0006355]; retinal cone cell development [GO:0046549]; retinal cone cell differentiation [GO:0042670]; retinal cone cell fate determination [GO:0042671]; retinoic acid receptor signaling pathway [GO:0048384]; thyroid hormone receptor signaling pathway [GO:0002154]

0	0	3396.42	0	0.49	0.61	0.42	0	3127.32	0.33	0	1152.71	1.87	0	2244.51	0.44	0.46	3252.91	13178.49	Sphingosine-1-phosphate phosphatase 2 (SPPase2) (Spp2) (hSPP2) (EC 3.1.3.-) (Sphingosine-1-phosphatase 2)	SGPP2	phospholipid dephosphorylation [GO:0046839]; regulation of type B pancreatic cell proliferation [GO:0061469]; sphingolipid catabolic process [GO:0030149]; sphingosine metabolic process [GO:0006670]
980.54	946.75	1349.29	118.44	1430.81	2340.92	40.26	443.53	10.72	112.94	385.12	1461.92	550.8	1903.37	652.38	125.66	231.71	90.21	13175.37	Myocardial zonula adherens protein (GRINL1A upstream protein) (Gup)	MYZAP MYOZAP	intracellular signal transduction [GO:0035556]
153.18	205.15	755.83	40.55	130.21	340.37	173.71	50.44	3149.63	57.08	55.59	1066.4	229.8	348.05	2562.47	189.58	161.25	3499.93	13169.22	Protein PRRC2B (HLA-B-associated transcript 2-like 1) (Proline-rich coiled-coil protein 2B)	PRRC2B BAT2L BAT2L1 KIAA0515	cell differentiation [GO:0030154]
1185.68	577.82	735.98	351.1	866.23	1488.66	109.38	538.19	222.83	453.98	388.04	610.21	2491.24	1014.39	748.15	253.35	694.39	420	13149.62	Troponin I, fast skeletal muscle (Troponin I, fast-twitch isoform)	TNNI2	cardiac muscle contraction [GO:0060048]; skeletal muscle contraction [GO:0003009]

478.47		923.17	679.03	381.47	966.89	740.08	15.14	351.09	476.23	165.19	185.5	1046.91	875.51	2549.2	1132.9	347.56	711.05	1101.1	13126.49	Serine/threonine-protein kinase Nek1 (EC 2.7.11.1) (Never in mitosis A-related kinase 1) (NimA-related protein kinase 1) (Renal carcinoma antigen NY-REN-55)	NEK1 KIAA1901	cell division [GO:0051301]; cilium assembly [GO:0060271]; protein phosphorylation [GO:0006468]
843.98		171.28	1539.41	197.41	251.65	1490.55	47.44	47.4	268.92	236.55	97.94	2758.29	2038.7	741.25	845.54	605.44	465.23	460.25	13107.23	Alpha-1,3-mannosyl-glycoprotein 4-beta-N-acetylglucosaminyltransferase C (EC 2.4.1.145) (N-glycosyl-oligosaccharide-glycoprotein N-acetylglucosaminyltransferase IVc) (GnT-IVc) (N-acetylglucosaminyltransferase IVc) (UDP-N-acetylglucosamine: alpha-1,3-D-mannoside beta-1,4-N-acetylglucosaminyltransferase IVc)	mgat4c zgc:101663	protein N-linked glycosylation [GO:0006487]

1447.74	480.59	649.85	532.8	468.86	1012.05	32.5	298.76	44.05	456.14	267.42	440.7	2900.98	1511.86	1079.99	489.79	829.36	156.75	13100.19	Cathepsin S (EC 3.4.22.27)	CTSS	adaptive immune response [GO:0002250]; antigen processing and presentation [GO:0019882]; antigen processing and presentation of exogenous peptide antigen via MHC class II [GO:0019886]; antigen processing and presentation of peptide antigen [GO:0048002]; basement membrane disassembly [GO:0034769]; cellular response to thyroid hormone stimulus [GO:0097067]; collagen catabolic process [GO:0030574]; extracellular matrix disassembly [GO:0022617]; immune response [GO:0006955]; positive regulation of cation channel activity [GO:2001259]; protein processing [GO:0016485]; proteolysis [GO:0006508]; proteolysis involved in protein catabolic process [GO:0051603]; regulation of antigen processing and presentation [GO:0002577]; response to acidic pH [GO:0010447]; toll-like receptor signaling pathway [GO:0002224]
145.8	276.41	425.75	41.57	333.5	435.76	16.38	171.02	3804.37	39.73	157.09	1171.96	167.09	673.84	1235.06	187.48	305.53	3509.43	13097.77	Clathrin coat assembly protein AP180 (91 kDa synaptosomal-associated protein) (Clathrin coat-associated protein AP180) (Phosphoprotein F1-20)	SNAP91 KIAA0656	clathrin coat assembly [GO:0048268]; clathrin-dependent endocytosis [GO:0072583]; protein transport [GO:0015031]; regulation of clathrin-dependent endocytosis [GO:2000369]; vesicle budding from membrane [GO:0006900]

845.08	708.79	738	339.58	638.14	1155.55	1839.87	396.29	615.65	308.69	260.34	582.65	749.89	887.56	551.18	1110.77	583.55	776.33	13087.91	RalA-binding protein 1 (RalBP1) (Cytocentrin) (Dinitrophenyl S-glutathione ATPase) (DNP-SG ATPase) (EC 7.6.2.2, EC 7.6.2.3) (Ral-interacting protein 1)	Ralbp1	cell division [GO:0051301]; doxorubicin transport [GO:1900753]; endocytosis [GO:0006897]; negative regulation of smooth muscle cell proliferation [GO:0048662]; positive regulation of mitochondrial fission [GO:0090141]; positive regulation of oxidative stress-induced neuron intrinsic apoptotic signaling pathway [GO:1903378]; positive regulation of protein phosphorylation [GO:0001934]; receptor-mediated endocytosis [GO:0006898]; regulation of Cdc42 protein signal transduction [GO:0032489]; regulation of postsynaptic membrane neurotransmitter receptor levels [GO:0099072]; small GTPase-mediated signal transduction [GO:0007264]; transmembrane transport [GO:0055085]; xenobiotic detoxification by transmembrane export across the plasma membrane [GO:1990961]
1026.79	670.71	959.46	458.7	779.87	965.21	1301.26	350.28	629.42	265.3	418.85	611.11	872.3	1108.62	621.4	776.23	665.19	569.79	13050.49	Integral membrane protein 2C [Cleaved into: CT-BRI3]	Itm2c	negative regulation of amyloid precursor protein biosynthetic process [GO:0042985]; negative regulation of neuron projection development [GO:0010977]; neuron differentiation [GO:0030182]; positive regulation of extrinsic apoptotic signaling pathway [GO:2001238]
1.96	2548.83	0	0	3872.9	28.08	68.61	1483.55	6.52	1.5	826.19	2.03	6.79	3274.64	15.63	19.16	868.69	9.38	13034.46	Transmembrane protein 87A (Elkin1)	tmem87a TGas047b06.1	cellular response to mechanical stimulus [GO:0071260]; detection of mechanical stimulus involved in sensory perception of touch [GO:0050976]

0		1926.9	0	20.81	431.29	0	0	996.52	0	0	2368	8.23	0	2966.54	0	0	4315.19	0	13033.48	TLC domain-containing protein 3A (Protein CT120) (Protein FAM57A)	TLCD3A FAM57A	lipid homeostasis [GO:0055088]
166.39		62.96	3410.95	23.89	96.38	143.65	32.76	18.47	3252.56	42.32	31.45	772.91	187.27	66.72	2096.42	47.49	67.3	2509.3	13029.19	Pigment epithelium-derived factor (PEDF) (Serpin F1)	SERPINF1 PEDF	negative regulation of angiogenesis [GO:0016525]; positive regulation of neurogenesis [GO:0050769]
609.57		975.48	629.93	362.51	635.97	766.5	223.9	312.08	1131.72	610.39	437.24	1240.68	1180.25	988.2	928.31	336.79	422.18	1228.02	13019.72	Spermatogenesis-associated protein 7 homolog	Spata7	microtubule cytoskeleton organization [GO:0000226]; photoreceptor cell maintenance [GO:0045494]; protein localization to photoreceptor connecting cilium [GO:1903621]; protein localization to photoreceptor outer segment [GO:1903546]; visual perception [GO:0007601]

1544.74	534.84	600.17	431.28	783.53	378.36	118.73	632.34	754.84	585.5	522.07	408.08	727.7	1254.53	934.36	294.69	1187.44	1316.81	13010.01	Signal transducer and activator of transcription 1-alpha/beta (Transcription factor ISGF-3 components p91/p84)	STAT1	blood circulation [GO:0008015]; cell surface receptor signaling pathway via JAK-STAT [GO:0007259]; cell surface receptor signaling pathway via STAT [GO:0097696]; cellular response to insulin stimulus [GO:0032869]; cellular response to interferon-beta [GO:0035458]; cellular response to type II interferon [GO:0071346]; defense response [GO:0006952]; defense response to virus [GO:0051607]; interleukin-27-mediated signaling pathway [GO:0070106]; interleukin-7-mediated signaling pathway [GO:0038111]; interleukin-9-mediated signaling pathway [GO:0038113]; metanephric mesenchymal cell differentiation [GO:0072162]; metanephric mesenchymal cell proliferation involved in metanephros development [GO:0072136]; negative regulation by virus of viral protein levels in host cell [GO:0046725]; negative regulation of angiogenesis [GO:0016525]; negative regulation of canonical NF-kappaB signal
975.88	674.91	297.04	281.87	769.44	922.69	1899.67	309.06	551.2	314.23	303.74	719.49	1116.75	703.02	599.5	1173.31	750.71	617.53	12980.04	Polycomb group RING finger protein 3 (RING finger protein 3A)	PCGF3 RNF3 RNF3A	random inactivation of X chromosome [GO:0060816]; regulation of transcription by RNA polymerase II [GO:0006357]
570.82	849.96	247.56	974.97	890.63	2564.7	0	609.41	198.13	310.47	337.33	2971.34	1039.55	423.85	260.2	65.73	263.35	399.97	12977.97	Terminal uridylyltransferase 7 (TUTase 7) (EC 2.7.7.52) (Zinc finger CCHC domain-containing protein 6)	TUT7 HS2 KIAA1711 ZCCHC6	miRNA metabolic process [GO:0010586]; oocyte maturation [GO:0001556]; polyuridylation-dependent mRNA catabolic process [GO:1990074]; pre-miRNA processing [GO:0031054]; RNA 3'-end processing [GO:0031123]; transposable element silencing by mRNA destabilization [GO:0141008]

372.89	1528.23	527.95	285.29	2514.89	400.91	21.61	739.03	304.76	313.38	509.94	1300.45	189.79	1115.79	327.22	346.53	1717.08	460.61	12976.35	Protein Red (Cytokine IK) (IK factor)	Ik	mitotic cell cycle [GO:0000278]; mitotic spindle assembly checkpoint signaling [GO:0007094]; mRNA splicing, via spliceosome [GO:0000398]; protein localization to kinetochore [GO:0034501]
635.71	572.65	406.65	309.9	550.67	821.95	2743.2	267.35	433.47	226.41	210.14	453.92	909.5	988.68	521.79	1765.6	539.42	618.57	12975.58	Cyclic AMP receptor-like protein A	crlA DDB_G0280983	adenylate cyclase-activating G protein-coupled receptor signaling pathway [GO:0007189]; negative regulation of cell growth [GO:0030308]; signal transduction [GO:0007165]; sporulation resulting in formation of a cellular spore [GO:0030435]

664.99	259.34	1060.04	194.08	243.46	775.34	203.72	98.87	2024.3	199.42	127.12	1024.3	750.24	680.54	1733.3	290.34	387.19	2257.63	12974.22	Solute carrier family 4 member 11 (Sodium borate cotransporter 1) (NaBC1)	SLC4A11 BTR1	bicarbonate transport [GO:0015701]; borate transport [GO:0046713]; cellular hypotonic response [GO:0071476]; cellular response to oxidative stress [GO:0034599]; fluid transport [GO:0042044]; intracellular monoatomic cation homeostasis [GO:0030003]; monoatomic anion transport [GO:0006820]; monoatomic ion homeostasis [GO:0050801]; proton transmembrane transport [GO:1902600]; regulation of mesenchymal stem cell differentiation [GO:2000739]; regulation of mitochondrial membrane potential [GO:0051881]; sodium ion transport [GO:0006814]; transmembrane transport [GO:0055085]
499.03	681.45	1187.2	104.94	670.86	932.83	58.92	323	1496.4	205.34	247.42	1048.78	609.91	953.42	1653.64	143.58	401.8	1731.59	12950.11	Histone-lysine N-methyltransferase 2C (Lysine N-methyltransferase 2C) (EC 2.1.1.364) (Homologous to ALR protein) (Myeloid/lymphoid or mixed-lineage leukemia protein 3)	KMT2C HALR KIAA1506 MLL3	methylation [GO:0032259]; positive regulation of transcription by RNA polymerase II [GO:0045944]

312.69	221.49	351.74	217.4	198.21	324.66	4792.1	103.42	540.22	163.48	156.31	414.01	448.65	276.08	544.51	2921.96	240.07	696.03	12923.03	Chromodomain-helicase-DNA-binding protein 2 (CHD-2) (EC 3.6.4.-) (ATP-dependent helicase CHD2)	CHD2	DNA damage response [GO:0006974]; gene expression [GO:0010467]; hematopoietic stem cell differentiation [GO:0060218]; muscle organ development [GO:0007517]; nucleosome organization [GO:0034728]
643.26	590.49	1952.84	326.61	586.1	1027.51	233.29	421.97	1006.33	368.9	445.8	483.81	580.47	1006.11	1253.73	299.05	362.59	1330.04	12918.9	3'-5' exoribonuclease HELZ2 (EC 3.1.13.1) (ATP-dependent RNA helicase PRIC285) (EC 3.6.4.13) (Helicase with zinc finger 2, transcriptional coactivator) (Helicase with zinc finger domain 2) (PPAR-alpha-interacting complex protein 285) (PPAR-gamma DNA-binding domain-interacting protein 1) (PDIP1) (PPAR-gamma DBD-interacting protein 1) [illegible]	HELZ2 KIAA1769 PRIC285	positive regulation of transcription by RNA polymerase II [GO:0045944]; regulatory ncRNA-mediated post-transcriptional gene silencing [GO:0035194]

0	0	12906.72	0	0	0	2.51	0	0	0	0	0	0	0	0	0	0	3.59	12912.82	Cadherin-18 (Cadherin-14)	CDH18 CDH14	adherens junction organization [GO:0034332]; calcium-dependent cell-cell adhesion via plasma membrane cell adhesion molecules [GO:0016339]; cell migration [GO:0016477]; cell morphogenesis [GO:0000902]; cell-cell adhesion mediated by cadherin [GO:0044331]; cell-cell junction assembly [GO:0007043]; homophilic cell adhesion via plasma membrane adhesion molecules [GO:0007156]
966.57	641.19	2234.35	183.52	238.13	1455.21	3.84	157.21	795.88	194.75	193.33	491.5	2558.65	716.3	481.63	189.18	283.91	1125.92	12911.07	Carboxypeptidase B2 (EC 3.4.17.20) (Carboxypeptidase R) (CPR) (Carboxypeptidase U) (CPU) (Thrombin-activable fibrinolysis inhibitor) (TAFI)	Cpb2	blood coagulation [GO:0007596]; cellular response to glucose stimulus [GO:0071333]; fibrinolysis [GO:0042730]; liver regeneration [GO:0097421]; negative regulation of fibrinolysis [GO:0051918]; negative regulation of hepatocyte proliferation [GO:2000346]; negative regulation of plasminogen activation [GO:0010757]; positive regulation of extracellular matrix constituent secretion [GO:0003331]; protein catabolic process [GO:0030163]; proteolysis [GO:0006508]; response to xenobiotic stimulus [GO:0009410]
563.95	661.19	554.15	312.4	682.71	1096.28	2457.7	202.99	340.57	192.95	232.21	630.75	946.61	1035.91	456.17	1469.33	636.45	427.22	12899.54	Cytosolic non-specific dipeptidase (EC 3.4.13.18) (CNDP dipeptidase 2) (Threonyl dipeptidase)	CNDP2	proteolysis [GO:0006508]

27.52	29.53	2600.65	14.06	9.03	359.73	1.1	8.08	299.11	13.41	8.26	8307.49	35.8	9.86	483.44	17.67	28.57	616.73	12870.04	Phospholipase DDHD1 (EC 3.1.1.111) (EC 3.1.1.32) (DDHD domain-containing protein 1) (Phosphatidic acid-preferring phospholipase A1 homolog) (PA-PLA1) (EC 3.1.1.118) (Phospholipid sn-1 acylhydrolase)	DDHD1 KIAA1705	phosphatidylinositol metabolic process [GO:0046488]; positive regulation of mitochondrial fission [GO:0090141]
816.21	1465.53	455.66	408.53	1028.81	672.9	420.58	626.99	380.3	261.26	319.44	575.91	1568.22	908.71	535.63	564.25	1313.05	547.88	12869.86	Excitatory amino acid transporter 5 (Retinal glutamate transporter) (Solute carrier family 1 member 7)	SLC1A7 EAAT5	chloride transmembrane transport [GO:1902476]; dicarboxylic acid transport [GO:0006835]; L-glutamate transmembrane transport [GO:0015813]; monoatomic ion transport [GO:0006811]; neurotransmitter transport [GO:0006836]; neurotransmitter uptake [GO:0001504]
1910.24	464.04	406.95	817.53	304.01	1417.47	245.39	108.1	404.2	486.39	158.5	308.65	2536.98	1158.28	811.35	426.66	450.69	426.12	12841.55	ATP synthase peripheral stalk subunit OSCP, mitochondrial (ATP synthase subunit O) (Oligomycin sensitivity conferral protein) (OSCP)	ATP5PO ATP5O	proton motive force-driven mitochondrial ATP synthesis [GO:0042776]

503.27	901.89	578.78	263.02	969.9	645.33	595.04	385.73	713.21	232.08	329.42	487.45	471.08	2001.59	806.2	1137.16	691.97	1126.62	12839.74	Transcription factor SOX-6	SOX6	brain development [GO:0007420]; cartilage condensation [GO:0001502]; cartilage development [GO:0051216]; cell fate commitment [GO:0045165]; chondrocyte differentiation [GO:0002062]; positive regulation of chondrocyte differentiation [GO:0032332]; spinal cord oligodendrocyte cell differentiation [GO:0021529]
1606.55	482.17	1266.95	449.64	417.26	2108.22	284.52	224.8	291.26	587.05	263.96	509.84	1554.78	809.23	560.94	389.18	637.85	362.59	12806.79	Inactive tyrosine-protein kinase PRAG1 (PEAK1-related kinase-activating pseudokinase 1) (Pragmin) (Sugen kinase 223) (SgK223)	PRAG1 SGK223	cell migration [GO:0016477]; negative regulation of neuron projection development [GO:0010977]; positive regulation of Rho protein signal transduction [GO:0035025]; regulation of cell motility [GO:2000145]; regulation of cell shape [GO:0008360]; regulation of Notch signaling pathway [GO:0008593]

916.94	944.76	890.07	374.42	1101.26	1499.58	359.38	489.98	186.85	351.75	522.41	604.73	1178.36	1071.5	572.94	460.16	962.84	311.95	12799.88	Cytochrome P450 7B1 (24-hydroxycholesterol 7-alpha-hydroxylase) (EC 1.14.14.26) (25/26-hydroxycholesterol 7-alpha-hydroxylase) (EC 1.14.14.29) (3-hydroxysteroid 7-alpha hydroxylase) (Oxysterol 7-alpha-hydroxylase)	CYP7B1	B cell chemotaxis [GO:0035754]; bile acid biosynthetic process [GO:0006699]; cholesterol homeostasis [GO:0042632]; cholesterol metabolic process [GO:0008203]; epithelial cell proliferation [GO:0050673]; estrogen receptor signaling pathway [GO:0030520]; negative regulation of intracellular estrogen receptor signaling pathway [GO:0033147]; positive regulation of epithelial cell proliferation [GO:0050679]; prostate gland epithelium morphogenesis [GO:0060740]; steroid biosynthetic process [GO:0006694]; sterol metabolic process [GO:0016125]
416.25	148.51	203.9	342.62	157.39	362.94	5511.12	46.41	270.45	209.19	115.94	188.66	468.69	223.16	275.65	3269.87	234.68	338.47	12783.9	Large ribosomal subunit protein mL65 (28S ribosomal protein S30, mitochondrial) (MRP-S30) (S30mt)	MRPS30	translation [GO:0006412]
1110.36	458.46	132.31	370.14	1215.64	1303.9	903.88	265.07	46.29	680.72	385.71	578.61	1679.86	1217.97	670.62	702.52	796.24	258.53	12776.83	Spermatogenesis-associated protein 2	spata2	programmed cell death [GO:0012501]; protein K63-linked deubiquitination [GO:0070536]; protein linear deubiquitination [GO:1990108]; regulation of inflammatory response [GO:0050727]; regulation of necroptotic process [GO:0060544]; regulation of tumor necrosis factor-mediated signaling pathway [GO:0010803]

933.92	368.87	2404.46	191.26	622.7	760.86	991.1	246.74	673.38	299.17	322.2	1076.66	470.12	264.02	923.87	709.86	284.16	1220.32	12763.67	Formin-binding protein 1 (Formin-binding protein 17) (hFBP17)	FNBP1 FBP17 KIAA0554	endocytosis [GO:0006897]; signal transduction [GO:0007165]
2963.77	424.32	1196.38	599.72	262.72	1105.86	6.85	216.74	112.73	569.88	244.89	753.17	2113.03	809.37	260.28	131.2	803.4	160.68	12734.99	Septin-9 (MLL septin-like fusion protein MSF-A) (MLL septin-like fusion protein) (Ovarian/Breast septin) (Ov/Br septin) (Septin D1)	SEPTIN9 KIAA0991 MSF SEPT9	actin cytoskeleton organization [GO:0030036]; cytoskeleton-dependent cytokinesis [GO:0061640]; positive regulation of non-motile cilium assembly [GO:1902857]; protein localization [GO:0008104]; septin cytoskeleton organization [GO:0032185]
2623.58	1026.5	527.82	429.68	639.21	668.28	225.67	1128.44	76.19	516.44	723.14	638.95	970.04	763.46	421.93	135.25	1042.79	117.1	12674.47	5'-3' exoribonuclease 2 (EC 3.1.13.-) (Protein Dhm1)	Xrn2 Dhm1	DNA recombination [GO:0006310]; DNA repair [GO:0006281]; hippocampus development [GO:0021766]; microtubule-based process [GO:0007017]; mRNA processing [GO:0006397]; neuron differentiation [GO:0030182]; retina development in camera-type eye [GO:0060041]; RNA metabolic process [GO:0016070]; spermatogenesis [GO:0007283]; termination of RNA polymerase II transcription [GO:0006369]

1410.76	437.95	550.04	371.4	333.4	883.74	138.3	176.34	673.76	282.35	124.63	544.93	3632.61	696.11	847.82	336.74	371.26	856.56	12668.7	Protein wntless homolog (Integral membrane protein GPR177)	WLS GPR177 RCJMB04_5c7	intracellular protein transport [GO:0006886]; positive regulation of Wnt protein secretion [GO:0061357]; positive regulation of Wnt signaling pathway [GO:0030177]; Wnt protein secretion [GO:0061355]; Wnt signaling pathway [GO:0016055]
854.67	212	766.79	487.91	190.01	921.54	1562.68	69.74	569.56	508.27	127.2	749.56	3125.17	186.55	496.71	1222.27	125.12	489.09	12664.84	Interferon gamma receptor 1 (Interferon gamma receptor 1-1)	ifngr1 ifngr1-1	
1240.3	382.8	650.42	676.6	467	1072.31	229.53	570.35	28.72	557.8	529.23	1464.67	2055.67	883.8	372.05	421.34	892.13	168.42	12663.14	Mitogen-activated protein kinase 1 (MAP kinase 1) (MAPK 1) (EC 2.7.11.24) (ERT1) (Extracellular signal-regulated kinase 2) (ERK-2) (MAP kinase isoform p42) (p42-MAPK) (Mitogen-activated protein kinase 2) (MAP kinase 2) (MAPK 2)	Mapk1 Erk2 Mapk Prkm1	androgen receptor signaling pathway [GO:0030521]; animal organ morphogenesis [GO:0009887]; apoptotic process [GO:0006915]; B cell receptor signaling pathway [GO:0050853]; Bergmann glial cell differentiation [GO:0060020]; cardiac neural crest cell development involved in heart development [GO:0061308]; caveolin-mediated endocytosis [GO:0072584]; cellular response to amino acid starvation [GO:0034198]; cellular response to tumor necrosis factor [GO:0071356]; cytosine metabolic process [GO:0019858]; DNA damage response [GO:0006974]; DNA-templated transcription [GO:0006351]; epidermal growth factor receptor signaling pathway [GO:0007173]; ERBB2-ERBB3 signaling pathway [GO:0038133]; ERK1 and ERK2 cascade [GO:0070371]; face development [GO:0060324]; heart development [GO:0007507]; insulin receptor signaling pathway [GO:0008286]; insulin-like growth factor receptor signaling pathway [illegible]

761.96	706.15	701	322.85	710.38	1211.09	1452.59	319.14	332.23	212.56	269.06	1026.25	668.34	782.18	524.49	1583.44	494.8	554.96	12633.47	Collagen alpha-1(VIII) chain (Endothelial collagen) [Cleaved into: Vastatin]	COL8A1 C3orf7	angiogenesis [GO:0001525]; camera-type eye morphogenesis [GO:0048593]; cell adhesion [GO:0007155]; endodermal cell differentiation [GO:0035987]; endothelial cell proliferation [GO:0001935]; positive regulation of cell-substrate adhesion [GO:0010811]
808.88	455.51	1209.57	239.11	410.95	726.3	941.56	178.69	1157.74	169.02	153.51	830.03	819.86	616.87	790.78	1014.68	412.54	1689.85	12625.45	Probable N-acetyltransferase 14 (EC 2.3.1.-)	nat14 zgc:153234	
719.17	373.11	2246.36	278.73	557.4	766.32	3.11	688.17	819.62	457.53	598.07	2382.58	534.92	145.45	846.25	38.6	172.5	987.57	12615.46	Polymeric immunoglobulin receptor (PIgR) (Poly-Ig receptor) [Cleaved into: Secretory component]	PIGR	immunoglobulin transcytosis in epithelial cells mediated by polymeric immunoglobulin receptor [GO:0002415]

819.98	607.55	970.99	187.17	532.91	1072.77	1072.9	270.5	775.6	232.96	217	593.84	626.69	723.83	1455.79	755.14	526.91	1161.35	12603.88	Myelin-associated glycoprotein (Siglec-4a)	MAG GMA	axon regeneration [GO:0031103]; cell adhesion [GO:0007155]; cell-cell adhesion via plasma-membrane adhesion molecules [GO:0098742]; cellular response to mechanical stimulus [GO:0071260]; central nervous system myelin formation [GO:0032289]; negative regulation of axon extension [GO:0030517]; negative regulation of neuron apoptotic process [GO:0043524]; negative regulation of neuron differentiation [GO:0045665]; negative regulation of neuron projection development [GO:0010977]; positive regulation of astrocyte differentiation [GO:0048711]; positive regulation of myelination [GO:0031643]; substantia nigra development [GO:0021762]; transmission of nerve impulse [GO:0019226]
1008.8	557.92	856.82	304.67	447.07	934.86	1475.18	228.44	740.65	288.57	234.65	637.23	834.24	599.45	842.17	1333.12	444.69	832.89	12601.42	Prominin-1-A (Prominin-like protein 1)	prom1a proml1	
4191.03	0	7.13	610.34	78.27	114.82	7.24	0	0	2274.54	51.35	91.28	4954.13	6.38	0	188.63	7.1	0	12582.24	Galectin-3-binding protein B (Lectin galactoside-binding soluble 3-binding protein B)	lgals3bpb zgc:77059	cell adhesion [GO:0007155]
658.87	755.87	1006.49	246.66	571.09	1162.24	788.89	263.65	1023.55	208.36	236.23	1169.08	728.55	819.84	1003.66	443.12	483.31	1009.36	12578.82	Metal transporter CNNM1 (Ancient conserved domain-containing protein 1) (Cyclin-M1)	CNNM1 ACDP1	magnesium ion homeostasis [GO:0010960]; monoatomic ion transport [GO:0006811]
449.75	534.07	536.43	193.07	525.57	454.13	1685.07	287.99	1136.64	164.87	189.45	587.92	488.92	651.59	1549.38	945.67	346.48	1817.51	12544.51	Actinia tenebrosa protease inhibitors (Carboxypeptidase inhibitor SmCI-like) [Cleaved into: ATPI-I;ATPI-II]		

111.2	23.74	18.15	84.14	32.45	336.65	18.78	1.21	4.19	47.97	59.24	115.07	7.72	6480.89	216.46	1584.34	3349.2	28.37	12519.77	Serine/threonine-protein kinase 38-like (EC 2.7.11.1) (NDR2 protein kinase) (Nuclear Dbf2-related kinase 2)	STK38L KIAA0965 NDR2	intracellular signal transduction [GO:0035556]; negative regulation of autophagy [GO:0010507]; protein phosphorylation [GO:0006468]; regulation of cellular component organization [GO:0051128]
1609	535.03	1277.5	276.46	678.39	687.63	311.72	255.5	1171.34	492.94	235.61	725.27	472.19	659.27	1140.01	342.73	597.62	1035.34	12503.55	Large neutral amino acids transporter small subunit 3 (L-type amino acid transporter 3) (Prostate cancer overexpressed gene 1 protein) (Solute carrier family 43 member 1)	SLC43A1 LAT3 PB39 POV1	amino acid transport [GO:0006865]; isoleucine transport [GO:0015818]; L-leucine transport [GO:0015820]; L-valine transmembrane transport [GO:1903785]; negative regulation of amino acid transport [GO:0051956]; negative regulation of L-leucine import across plasma membrane [GO:1905533]; neutral amino acid transport [GO:0015804]
1230.63	277.12	2466.18	354.16	216.73	1242.63	114.33	76.23	1794.85	297.32	54.17	617.02	1170.93	93.37	961.75	240.09	338.85	936.17	12482.53	Probable E3 ubiquitin-protein ligase DTX3 (EC 2.3.2.27) (Protein deltex-3) (Deltex3) (mDTX3) (RING-type E3 ubiquitin transferase DTX3)	Dtx3	Notch signaling pathway [GO:0007219]; protein ubiquitination [GO:0016567]

4.18	5.86	413.85	1.23	2.54	3.06	9.51	3.38	4147.48	2.17	0.44	1066.34	108.2	13.52	2655.79	18.92	8.76	4016.97	12482.2	Zinc finger SWIM domain-containing protein 8	ZSWIM8	positive regulation of miRNA catabolic process [GO:2000627]; proteasome-mediated ubiquitin-dependent protein catabolic process [GO:0043161]; protein ubiquitination [GO:0016567]
1678.88	880.6	880.28	323.14	989.4	1372.73	155.5	670.75	105.85	414.99	447.15	883.4	896.77	1050.44	436.96	184.98	853.36	253.02	12478.2	Pericentriolar material 1 protein (PCM-1)	PCM1	cilium assembly [GO:0060271]; intraciliary transport involved in cilium assembly [GO:0035735]; microtubule anchoring at centrosome [GO:0034454]; non-motile cilium assembly [GO:1905515]; positive regulation of intracellular protein transport [GO:0090316]; protein localization to centrosome [GO:0071539]

174.54	0	2092.92	18.23	0.19	67.51	4.38	0.92	3229.18	54.88	2.78	757.12	49.04	0	2203.51	3.19	3.6	3801.85	12463.84	Suppressor of hairless protein homolog (X-Su(H))	rbpj rbpsuh suh	Notch signaling pathway [GO:0007219]; positive regulation of transcription by RNA polymerase II [GO:0045944]; positive regulation of transcription of Notch receptor target [GO:0007221]
52.51	2724.1	307.77	87.24	2131.45	323.1	121.36	1097.72	143.24	35.78	717.29	283.1	52.09	2573.96	145.83	177.2	1328.01	150.33	12452.08	X-linked retinitis pigmentosa GTPase regulator-interacting protein 1 (RPGR-interacting protein 1)	RPGRIP1	non-motile cilium assembly [GO:1905515]; visual perception [GO:0007601]
578.81	613.78	667.34	457.65	644.71	817.14	1534.47	235.54	535.48	224.01	250.35	726.13	901.09	982.49	732	1198.76	540.23	810.06	12450.04	Unconventional myosin-VI (Unconventional myosin-6)	MYO6	actin filament organization [GO:0007015]; actin filament-based movement [GO:0030048]; DNA damage response, signal transduction by p53 class mediator [GO:0030330]; endocytosis [GO:0006897]; inner ear auditory receptor cell differentiation [GO:0042491]; inner ear morphogenesis [GO:0042472]; intracellular protein transport [GO:0006886]; protein localization [GO:0008104]; regulation of secretion [GO:0051046]; sensory perception of sound [GO:0007605]
420.43	551.61	1303.94	345.19	386.74	2483.99	164.83	166.27	569.81	224.98	136.44	1071.66	1392.41	568.73	1064.39	328.24	497.23	738.23	12415.12	Tropomodulin-4 (Skeletal muscle tropomodulin) (Sk-Tmod)	Tmod4	pointed-end actin filament capping [GO:0051694]

644.14	793.04	434.14	308.9	646.14	1344.13	1905.1	235.62	252.38	262.98	286.05	956.09	1031.04	522.47	412.5	1621.63	487.74	265.25	12409.34	Leucine-rich repeat and fibronectin type III domain-containing protein 1-like protein	lrfn1l zgc:172282	
417.35	983.81	547.72	323	789.41	1341	1365.34	218.41	227.66	156.78	232	912.96	1005.76	1044.66	557.7	1223.85	656.78	403.2	12407.39	Inositol hexakisphosphate kinase 2 (InsP6 kinase 2) (EC 2.7.4.-) (P(i)-uptake stimulator) (PiUS)	IP6K2 IHPK2	cellular response to flavonoid [GO:1905396]; inositol phosphate biosynthetic process [GO:0032958]; inositol phosphate metabolic process [GO:0043647]; negative regulation of cell growth [GO:0030308]; phosphatidylinositol phosphate biosynthetic process [GO:0046854]; positive regulation of apoptotic process [GO:0043065]; protein stabilization [GO:0050821]
50.45	2447.61	225.93	37.3	1365.7	138.71	22.37	1070.57	175.92	36.38	611.09	210.24	28.51	3862.16	200.62	40.74	1610.21	268.68	12403.19	Dol-P-Man:Man(5)GlcNAc(2)-PP-Dol alpha-1,3-mannosyltransferase (EC 2.4.1.258) (Asparagine-linked glycosylation protein 3 homolog) (Dol-P-Man-dependent alpha(1-3)-mannosyltransferase) (Dolichyl-P-Man:Man(5)GlcNAc(2)-PP-dolichyl mannosyltransferase) (Dolichyl-phosphate-mannose--glycolipid alpha-mannosyltransferase)	Alg3	dolichol-linked oligosaccharide biosynthetic process [GO:0006488]; protein N-linked glycosylation [GO:0006487]

199.12	260.22	490.58	110.35	263.49	457.57	4867.86	102.29	466.49	183.74	186.39	411.14	310.5	241.3	503.6	2487.17	280.65	573.48	12395.94	Protein mono-ADP-ribosyltransferase PARP11 (EC 2.4.2.-) (ADP-ribosyltransferase diphtheria toxin-like 11) (ARTD11) (Poly [ADP-ribose] polymerase 11) (PARP-11)	Parp11	cell differentiation [GO:0030154]; mRNA transport [GO:0051028]; nuclear envelope organization [GO:0006998]; protein auto-ADP-ribosylation [GO:0070213]; protein transport [GO:0015031]; spermatogenesis [GO:0007283]
978.78	805.61	1232.94	215.02	726.88	1010.03	20.13	290.33	915.93	274.22	274.97	806.16	694.09	1064.39	1174.45	141.57	492.02	1265.63	12383.15	Mesoderm induction early response protein 1 (Early response 1) (Er1) (Mi-er1)	Mier1 Kiaa1610	chromatin remodeling [GO:0006338]; regulation of DNA-templated transcription [GO:0006355]
949	301.66	732.21	124.99	108.32	301.67	2114.96	111.39	410.97	88.08	115.28	342.78	709.46	2589.48	517.21	1401.22	1193.47	255.66	12367.81	DBIRD complex subunit ZNF326 (Zinc finger protein 326) (Zinc finger protein interacting with mRNPs)	ZNF326 ZIRD	mRNA processing [GO:0006397]; regulation of DNA-templated transcription elongation [GO:0032784]; regulation of RNA splicing [GO:0043484]; RNA splicing [GO:0008380]

759.43	980.76	1282.41	827.96	938.55	1613.28	0	18.23	26.04	496.42	401.26	1224.22	1228.82	1775.3	737.61	4.11	21.58	27.97	12363.95	Serine/threonine-protein phosphatase PP1-beta (EC 3.1.3.16) (Glc seven-like phosphatase 2)	gsp-2 CBG00598	cell division [GO:0051301]; chromatin organization [GO:0006325]; meiotic cell cycle [GO:0051321]
29.64	0	0.93	170.96	1.78	3.04	6201.13	0	0	41.5	175.56	0	18.88	2.44	0	5607.4	105.29	1.1	12359.65	GRB2-associated-binding protein 1 (GRB2-associated binder 1) (Growth factor receptor bound protein 2-associated protein 1)	GAB1	signal transduction [GO:0007165]
0	0.61	4714.48	0	1.8	12.24	8.06	0	2349.14	0	0	746.59	0	6.09	1472.11	6.41	2.59	3036.54	12356.66	Glutamate receptor ionotropic, NMDA 3B (GluN3B) (N-methyl-D-aspartate receptor subtype 3B) (NMDAR3B) (NR3B)	GRIN3B	ionotropic glutamate receptor signaling pathway [GO:0035235]; modulation of chemical synaptic transmission [GO:0050804]; monoatomic cation transmembrane transport [GO:0098655]; protein insertion into membrane [GO:0051205]; regulation of calcium ion transport [GO:0051924]; regulation of synaptic plasticity [GO:0048167]; synaptic transmission, glutamatergic [GO:0035249]

480.52	410.92	831.21	130.95	409.7	622.46	2668.56	181.27	689.55	215.49	205.8	635.51	547.46	513.32	961.79	1545.7	330.08	966.47	12346.76	Macrophage mannose receptor 1 (MMR) (C-type lectin domain family 13 member D) (C-type lectin domain family 13 member D-like) (Human mannose receptor) (hMR) (Macrophage mannose receptor 1-like protein 1) (CD antigen CD206)	MRC1 CLEC13D CLEC13DL MRC1L1	cellular response to interleukin-4 [GO:0071353]; cellular response to lipopolysaccharide [GO:0071222]; cellular response to type II interferon [GO:0071346]; receptor-mediated endocytosis [GO:0006898]
1022.83	741.78	1019.27	298.08	677.49	1278.53	94.66	528.08	525.62	377.95	203.7	1110.91	1156.57	1139.52	700.47	171.05	578.23	706.44	12331.18	SEC14-like protein 2 (Alpha-tocopherol-associated protein) (TAP) (bTAP)	SEC14L2	
412.13	829.39	2113.89	153.53	793.47	2418.82	29.88	320.13	223.94	184.15	340.53	997.14	221.67	905.82	718.49	179.01	867.64	616.34	12325.97	Membrane-associated guanylate kinase, WW and PDZ domain-containing protein 1 (Atrophin-1-interacting protein 3) (AIP-3) (BAI1-associated protein 1) (BAP-1) (Membrane-associated guanylate kinase inverted 1) (MAGI-1) (Trinucleotide repeat-containing gene 19 protein) (WW domain-containing protein 3) (WWP3)	MAGI1 AIP3 BAIAP1 BAP1 TNRC19	cell adhesion [GO:0007155]; cell surface receptor signaling pathway [GO:0007166]; endothelial cell morphogenesis [GO:0001886]; positive regulation of cell-cell adhesion [GO:0022409]; protein-containing complex assembly [GO:0065003]; signal transduction [GO:0007165]
627.6	555.65	2831.4	219.14	555.28	1289.45	186.14	188.18	418.53	257.12	194.42	1783.11	438.63	911.24	618.87	404.72	398.42	443.87	12321.77	Zinc finger protein 276 (Zfp-276) (Zinc finger protein 477)	ZNF276 CENP-Z ZFP276 ZNF477	regulation of transcription by RNA polymerase II [GO:0006357]

468.02	775.27	789.82	142.3	751.14	703.62	595.26	304.04	1301.39	158.64	278.54	752.39	368.35	1066.67	1340.54	553.84	435.04	1535.54	12320.41	Progranulin (PGRN) (Acrogranin) (Epithelin precursor) (Glycoprotein of 88 Kda) (GP88) (Glycoprotein 88) (Granulin precursor) (PC cell-derived growth factor) (PCDGF) (Proepithelin) (PEPI) [Cleaved into: Paragranulin;Granulin-1 (Granulin G);Granulin-2 (Granulin F);Granulin-3 (Epithelin-2) (Granulin B);Granulin-4 (Epithelin-1) (Granulin	GRN	astrocyte activation involved in immune response [GO:0002265]; blastocyst hatching [GO:0001835]; embryo implantation [GO:0007566]; epithelial cell proliferation [GO:0050673]; locomotory exploration behavior [GO:0035641]; lysosomal lumen acidification [GO:0007042]; lysosomal protein catabolic process [GO:1905146]; lysosomal transport [GO:0007041]; lysosome organization [GO:0007040]; maintenance of synapse structure [GO:0099558]; microglial cell activation involved in immune response [GO:0002282]; negative regulation of microglial cell activation [GO:1903979]; negative regulation of neuron apoptotic process [GO:0043524]; negative regulation of neutrophil activation [GO:1902564]; negative regulation of respiratory burst involved in inflammatory response [GO:0060266]; positive regulation of angiogenesis [GO:0045766]; positive regulation of aspartic-type peptidase activity [GO:1905247]; positive regulation of
450.49	608.48	1561.63	243.11	766.55	916.92	172.83	526.65	778.16	195.2	284.47	1363.09	790.01	807.95	801.02	199.56	548.54	1301.01	12315.67	Cysteine-rich and transmembrane domain-containing protein 1	CYSTM1	
711.7	878.06	496.83	255.56	447.38	866.28	1730.09	323.26	556.45	220.61	271.18	482.7	1025.53	930.11	564.65	1192.48	688.91	652.1	12293.88	Capping protein, Arp2/3 and myosin-I linker protein 2 (Capping protein regulator and myosin 1 linker 2) (F-actin-uncapping protein RLTPR) (Leucine-rich repeat-containing protein 16C) (RGD, leucine-rich repeat, tropomodulin and proline-rich-containing protein)	CARMIL2 LRRC16C RLTPR	actin filament network formation [GO:0051639]; cell migration [GO:0016477]; establishment or maintenance of cell polarity [GO:0007163]; establishment or maintenance of monopolar cell polarity [GO:0061339]; negative regulation of barbed-end actin filament capping [GO:2000813]; positive regulation of cell migration [GO:0030335]; positive regulation of extracellular matrix disassembly [GO:0090091]; positive regulation of lamellipodium assembly [GO:0010592]; positive regulation of lamellipodium organization [GO:1902745]; positive regulation of ruffle assembly [GO:1900029]; regulation of Arp2/3 complex-mediated actin nucleation [GO:0034315]; wound healing, spreading of cells [GO:0044319]

450.45	558.66	1055.8	220.21	394.33	1333.64	1427.19	181.71	688.17	238.1	189.09	986.3	613.48	591	681.77	1228.47	453.13	1000.17	12291.67	Exocyst complex component 4 (Exocyst complex component Sec8)	EXOC4 KIAA1699 SEC8 SEC8L1	chemical synaptic transmission [GO:0007268]; exocytosis [GO:0006887]; Golgi to plasma membrane transport [GO:0006893]; membrane fission [GO:0090148]; mitotic cytokinesis [GO:0000281]; paraxial mesoderm formation [GO:0048341]; protein transmembrane transport [GO:0071806]; regulation of macroautophagy [GO:0016241]; vesicle docking involved in exocytosis [GO:0006904]; vesicle tethering involved in exocytosis [GO:0090522]
0	0	12285.47	0	0	0	0	0	0	0	0	3.4	0	0	0	0	0	0	12288.87	DELTA-sagatoxin-Srs1a (DELTA-SATX-Srs1a) (Cytolysin Src-1) (Src I)		cytolysis in another organism [GO:0051715]; monoatomic cation transport [GO:0006812]; pore complex assembly [GO:0046931]
3.08	1.16	423.13	0	1.67	15.72	0	0	4284.95	3.98	5.8	1060.26	0	0.71	2879.11	3.78	4.46	3591.77	12279.58	Uncharacterized protein 058R	FV3-058R	

2708.84	59.3	68.32	135.97	108.89	21.48	0	13.3	0.99	70.9	9.08	61.18	6412.11	426.38	175.46	1855.66	135.83	12.11	12275.8	Survival motor neuron protein 1	smn1 smn	axon arborization [GO:0140060]; axon development [GO:0061564]; axon extension [GO:0048675]; axonogenesis [GO:0007409]; dendrite development [GO:0016358]; motor neuron axon guidance [GO:0008045]; mRNA splicing, via spliceosome [GO:0000398]; neuromast regeneration [GO:0070657]; neuromuscular junction development [GO:0007528]; neuron cellular homeostasis [GO:0070050]; peripheral nervous system neuron axonogenesis [GO:0048936]; positive regulation of oxidative phosphorylation [GO:1903862]; protein complex oligomerization [GO:0051259]; regulation of axon extension involved in axon guidance [GO:0048841]; regulation of neuromuscular junction development [GO:1904396]; spliceosomal snRNP assembly [GO:0000387]; synaptic assembly at neuromuscular junction [GO:0051124]; tissue regeneration [GO:0042246]
18.02	9.92	604.32	3.05	3.07	149.71	0	11.81	3674.43	7.92	11.36	993.03	6.78	31.18	3739.59	20.98	2.84	2978.87	12266.88	Speriolin-like protein (Spermatogenesis and centriole-associated protein 1-like protein)	SPATC1L C21orf56	actin polymerization or depolymerization [GO:0008154]; positive regulation of cAMP/PKA signal transduction [GO:0141163]; spermatogenesis [GO:0007283]
254.28	285.37	535.14	374.94	276.62	176.8	1093.42	115.5	1361.14	194.17	285.27	635.13	250.17	1226.94	1447.63	1059.82	870.91	1814.91	12258.16	Ubiquitin carboxyl-terminal hydrolase 21 (EC 3.4.19.12) (Deubiquitinating enzyme 21) (Ubiquitin thioesterase 21) (Ubiquitin-specific-processing protease 21)	Usp21	neuron projection development [GO:0031175]; protein deubiquitination [GO:0016579]; proteolysis [GO:0006508]; transcription initiation-coupled chromatin remodeling [GO:0045815]

327.8	270.4	1030.31	101.09	278.99	643.64	12.86	145.02	1959.07	165.7	166.66	1113.11	306.92	512.58	2430.45	46	217.8	2527.14	12255.54	Thrombospondin-1 (Glycoprotein G)	THBS1 TSP-1 TSP1	behavioral response to pain [GO:0048266]; cell adhesion [GO:0007155]; negative regulation of angiogenesis [GO:0016525]; response to endoplasmic reticulum stress [GO:0034976]; response to unfolded protein [GO:0006986]
759.21	751.83	759.89	311.48	782.19	1006.8	665.49	461.92	824.17	281.32	375.36	796.75	913.51	820.35	796.64	718.56	551.74	673.39	12250.6	BTB/POZ domain-containing protein 6-A	btbd6a zgc:172197	neurogenesis [GO:0022008]; regulation of neurogenesis [GO:0050767]
1361.53	1610.88	359.14	361.45	2225.08	575.25	132.97	687.49	67.21	276.02	416.87	187.42	726.3	2106.08	225.98	182.54	613.89	127.11	12243.21	Integrator complex subunit 9 (Int9)	Ints9 D14Ertd231e	negative regulation of transforming growth factor beta receptor signaling pathway [GO:0030512]; RNA polymerase II transcription initiation surveillance [GO:0160240]; snRNA 3'-end processing [GO:0034472]

647.98	1428.01	366.4	247.87	1227.71	936.71	2.02	1405.27	24.79	324.02	577.71	258.4	1204.74	1484.67	308.92	68.81	1511.21	212.82	12238.06	FAD synthase (EC 2.7.7.2) (FAD pyrophosphorylase) (FMN adenylyltransferase) (Flavin adenine dinucleotide synthase) [Includes: Molybdenum cofactor biosynthesis protein-like region;FAD synthase region]	flad1 zgc:91843	FAD biosynthetic process [GO:0006747]
681.66	434.47	638.14	254.27	275.07	562.19	1722.49	86.24	1179.16	159.43	139.19	592.93	1017.3	393.88	1309.21	1242.56	262.78	1284.25	12235.22	Selenocysteine insertion sequence-binding protein 2 (SECIS-binding protein 2)	SECISBP2 SBP2	forebrain neuron development [GO:0021884]; mRNA stabilization [GO:0048255]; negative regulation of nuclear-transcribed mRNA catabolic process, nonsense-mediated decay [GO:2000623]; RNA catabolic process [GO:0006401]; selenocysteine incorporation [GO:0001514]; striatum development [GO:0021756]

9.94	23.86	615.36	9.55	7.34	110.67	25.03	6.36	2336.46	4.32	30.59	576.51	0	28.35	3875.73	37.92	14.31	4515.39	12227.69	Immunoglobulin-binding protein 1 (B-cell signal transduction molecule alpha 4) (Protein alpha-4) (CD79a-binding protein 1) (Protein phosphatase 2/4/6 regulatory subunit) (Renal carcinoma antigen NY-REN-16)	IGBP1 IBP1	B cell activation [GO:0042113]; intracellular signal transduction [GO:0035556]; negative regulation of stress-activated MAPK cascade [GO:0032873]; negative regulation of transcription by RNA polymerase II [GO:0000122]; regulation of dephosphorylation [GO:0035303]; regulation of microtubule-based movement [GO:0060632]; response to interleukin-1 [GO:0070555]; response to tumor necrosis factor [GO:0034612]; signal transduction [GO:0007165]
1447.06	964.78	1117.45	341.24	1016.61	828.04	13.04	350.32	727.76	297.86	318.46	659.5	698.69	1153.67	732.15	82.38	533.64	942.45	12225.1	Interleukin-1 receptor accessory protein (IL-1 receptor accessory protein) (IL-1RAcP) (EC 3.2.2.6)	Il1rap	cytokine-mediated signaling pathway [GO:0019221]; inflammatory response [GO:0006954]; interleukin-1-mediated signaling pathway [GO:0070498]; interleukin-33-mediated signaling pathway [GO:0038172]; positive regulation of interleukin-13 production [GO:0032736]; positive regulation of interleukin-4 production [GO:0032753]; positive regulation of interleukin-5 production [GO:0032754]; positive regulation of interleukin-6 production [GO:0032755]; positive regulation of synapse assembly [GO:0051965]; regulation of postsynaptic density assembly [GO:0099151]; regulation of presynapse assembly [GO:1905606]; synaptic membrane adhesion [GO:0099560]; trans-synaptic signaling by trans-synaptic complex [GO:0099545]

2048.85	240.46	376.7	839.74	245.24	472.17	2.38	98.35	45.72	590.7	163.87	416.33	5010.74	502.74	337.72	242.27	458.17	132.76	12224.91	Zinc transporter ZIP1 (Solute carrier family 39 member 1) (Zinc-iron-regulated transporter-like) (Zrt- and Irt-like protein 1) (ZIP-1) (hZIP1)	SLC39A1 IRT1 ZIP1 ZIRTL CGI-08 CGI-71	embryonic cranial skeleton morphogenesis [GO:0048701]; in utero embryonic development [GO:0001701]; limb development [GO:0060173]; monoatomic cation transport [GO:0006812]; zinc ion transmembrane transport [GO:0071577]
1784.39	686.19	666.3	262.84	759.54	865.88	1777.64	292.82	399.68	174.72	259.92	429.94	497.67	918.07	346.8	890.61	656.33	551.15	12220.49	Cytokine receptor-like factor 3 (Clf-3 protein)	crlf3 clf-3 zgc:110212	
714.08	460.03	1391.8	278.83	505.7	1845.88	347.83	236.07	686.53	301.13	185.81	832.8	1147.02	483.65	1079.7	451.52	246.55	1017.28	12212.21	Kinesin-like protein KIF16B (Sorting nexin-23)	KIF16B C20orf23 KIAA1590 SNX23	early endosome to late endosome transport [GO:0045022]; endoderm development [GO:0007492]; epidermal growth factor receptor signaling pathway [GO:0007173]; fibroblast growth factor receptor signaling pathway [GO:0008543]; formation of primary germ layer [GO:0001704]; Golgi to endosome transport [GO:0006895]; receptor catabolic process [GO:0032801]; regulation of receptor recycling [GO:0001919]; vesicle transport along microtubule [GO:0047496]
857.78	759.7	513.41	296.97	574.03	705.29	1794.73	317.25	517.47	233.71	271.16	443.34	1026.8	977.22	555.13	1073.58	696.05	590.82	12204.44	Glycogen phosphorylase, muscle form (EC 2.4.1.1) (Myophosphorylase)	Pygm	glycogen catabolic process [GO:0005980]

2.58	0.84	968.51	4.66	1.91	165.27	2.29	1.21	3273.39	2.58	3.64	1405.61	7.44	2.89	2476.99	7.77	8.8	3854.77	12191.15	Sorting nexin-7	Snx7	positive regulation of autophagosome assembly [GO:2000786]; protein transport [GO:0015031]
188.86	425.07	1778.3	582.46	642.54	775.95	1417.08	420.96	372.6	213.37	378.38	1120.2	382.77	474.59	668.91	1111.07	518.21	708.05	12179.37	Vitronectin (VN) (Glycoprotein 66) (S-protein) (Serum-spreading factor)	VTN	cell adhesion mediated by integrin [GO:0033627]; cell-matrix adhesion [GO:0007160]; immune response [GO:0006955]
460.61	439.44	1047.02	133.93	331.18	617.78	753.7	177.22	1597.35	132.06	153.19	896.27	654.25	578.65	1369.62	658.71	344.51	1828.4	12173.89	Coiled-coil domain-containing protein 50	CCDC50 RCJMB04_2n23	
0	0	2032.02	0	0	350.89	0	0	38.39	0	0	9596.84	0	0	126.05	0	0	19.99	12164.18	Saxitoxin and tetrodotoxin-binding protein 1	psbp1 pstbp1	response to toxic substance [GO:0009636]
1861.43	79.94	412.56	596.79	193.8	565.75	411.78	50.46	182.59	2029.71	164.47	247.4	2586.84	236.73	255.09	438.45	422.25	1419.18	12155.22	28S rRNA (cytosine-C(5))-methyltransferase (EC 2.1.1.-) (Nucleolar protein 1) (Nucleolar protein 2 homolog) (Proliferating-cell nucleolar antigen p120) (Proliferation-associated nucleolar protein p120)	Nop2 Nol1	blastocyst formation [GO:0001825]; regulation of signal transduction by p53 class mediator [GO:1901796]; ribosomal large subunit assembly [GO:0000027]; ribosomal large subunit biogenesis [GO:0042273]; rRNA processing [GO:0006364]

1738.25	640.92	449.32	314.02	443.91	431.17	2570.9	233.82	136.13	253.84	423.77	401.38	198.84	1384.23	305.93	1730.96	332.99	143.01	12133.39	Ribosomal protein eL22-like 1 (60S ribosomal protein L22-like 1) (Large ribosomal subunit protein eL22-like 1)	rpl22l1	translation [GO:0006412]
532.73	717.29	407.77	326.18	785.72	1933.09	47.62	649.45	715.34	548.8	753.18	389.53	533.78	1544.71	972.87	234.99	381.54	654.74	12129.33	Ataxin-7-like protein 1 (Ataxin-7-like protein 4)	ATXN7L1 ATXN7L4 KIAA1218	
747.72	979.49	1191.3	215.28	866.93	933.02	8.45	567.01	904.67	262.12	327.29	650.49	719.07	1128.31	813.34	110.31	620.58	1080.43	12125.81	Beta-1,4-mannosyl-glycoprotein 4-beta-N-acetylglucosaminyltransferase (EC 2.4.1.144) (N-glycosyl-oligosaccharide-glycoprotein N-acetylglucosaminyltransferase III) (GNT-III) (GlcNAc-T III) (N-acetylglucosaminyltransferase III)	Mgat3 Gnt3	amyloid-beta metabolic process [GO:0050435]; cellular response to oxidative stress [GO:0034599]; cognition [GO:0050890]; lysosomal protein catabolic process [GO:1905146]; N-acetylglucosamine metabolic process [GO:0006044]; negative regulation of lysosomal protein catabolic process [GO:1905166]; positive regulation of protein localization to early endosome [GO:1902966]; protein localization [GO:0008104]; protein localization to early endosome [GO:1902946]; protein N-linked glycosylation [GO:0006487]; regulation of cell migration [GO:0030334]
324.09	554.1	510.93	651.6	530.39	1712.41	6.21	547.65	851.54	383.12	335.23	2449.16	712.45	446.86	777.53	86.23	237.24	988.96	12105.7	Transcription factor TFIIIB component B" homolog (Transcription factor IIIB 150) (TFIIIB150) (Transcription factor-like nuclear regulator)	BDP1 KIAA1241 KIAA1689 TFNR	RNA polymerase III preinitiation complex assembly [GO:0070898]

537.88	732.74	2081.43	315.9	673.51	1221.51	809.78	336.47	493.39	160.83	232.51	624.98	670.74	580.13	900.3	628.14	507.95	593.26	12101.45	Probable ATP-dependent RNA helicase DDX5 (EC 3.6.4.13) (DEAD box protein 5)	DDX5 QtsA-17658	alternative mRNA splicing, via spliceosome [GO:0000380]; androgen receptor signaling pathway [GO:0030521]; epithelial to mesenchymal transition [GO:0001837]; estrogen receptor signaling pathway [GO:0030520]; intrinsic apoptotic signaling pathway by p53 class mediator [GO:0072332]; miRNA transcription [GO:0061614]; myoblast differentiation [GO:0045445]; negative regulation of transcription by RNA polymerase II [GO:0000122]; nuclear-transcribed mRNA catabolic process [GO:0000956]; positive regulation of DNA damage response, signal transduction by p53 class mediator [GO:0043517]; regulation of alternative mRNA splicing, via spliceosome [GO:0000381]; regulation of androgen receptor signaling pathway [GO:0060765]; regulation of osteoblast differentiation [GO:0045667]; regulation of skeletal muscle cell differentiation [GO:2001014]; regulation of transcription by RNA polymerase II
638.9	893.25	695.73	573.85	592.09	1437.33	6.83	1860.95	876.29	635.59	618.62	281.04	328.06	1394.88	332.55	89.44	450.36	394.54	12100.3	Protein yippee-like 5	YPEL5 CGI-127	cell population proliferation [GO:0008283]
360.55	3.61	2.6	1204.82	0	23.27	3509.03	0	0	0	0	0	0	0	0	6761.83	211.14	16.81	12093.66	BAR/IMD domain-containing adapter protein 2-like 1 (Brain-specific angiogenesis inhibitor 1-associated protein 2-like protein 1) (BAI1-associated protein 2-like protein 1)	Baiap2l1	actin crosslink formation [GO:0051764]; actin filament bundle assembly [GO:0051017]; plasma membrane organization [GO:0007009]; positive regulation of actin filament polymerization [GO:0030838]; regulation of actin cytoskeleton organization [GO:0032956]

647.09	714.45	354.94	232.46	1178.84	573.06	454.86	277.06	286.65	266.45	564.31	638.47	880.85	2811.35	404.01	447.41	971.04	386.76	12090.06	Microspherule protein 1 (58 kDa microspherule protein) (Cell cycle-regulated factor p78) (INO80 complex subunit J) (MCRS2)	MCRS1 INO80Q MSP58	chromatin remodeling [GO:0006338]; DNA recombination [GO:0006310]; DNA repair [GO:0006281]; negative regulation of telomere maintenance via telomere lengthening [GO:1904357]; positive regulation of DNA repair [GO:0045739]; positive regulation of DNA-templated transcription [GO:0045893]; positive regulation of protein localization to nucleolus [GO:1904751]; positive regulation of telomere maintenance in response to DNA damage [GO:1904507]; positive regulation of transcription by RNA polymerase II [GO:0045944]; protein modification process [GO:0036211]; regulation of cell cycle [GO:0051726]; regulation of chromosome organization [GO:0033044]; regulation of DNA repair [GO:0006282]; regulation of DNA replication [GO:0006275]; regulation of DNA strand elongation [GO:0060382]; regulation of embryonic development [GO:0045995]; telomere maintenance [GO:0000723]
488.31	529.71	393.03	196.18	350.93	434.78	3607.42	139.21	604.56	152.47	140.66	397.56	621.8	719.03	482.5	2002.95	226.82	599.34	12087.26	Cyclin-dependent kinase-like 1 (EC 2.7.11.22)	cdkl1 zgc:101002	
0	3702.89	815.54	5.36	1721.85	319.94	11.75	1530.6	191.29	3.84	200.18	566.94	0	2276.61	371.87	9.88	180.49	173.42	12082.45	CXXC motif containing zinc binding protein (UPF0587 protein C1orf123 homolog)	czib	
1159.68	523.77	977.73	360.31	615.01	1477.42	1152.17	255.88	477.11	371.63	227.72	513.45	871.42	715.02	516.43	856.61	456.36	554.02	12081.74	Tyrosine-protein phosphatase non-receptor type 13 (EC 3.1.3.48) (Fas-associated protein-tyrosine phosphatase 1) (FAP-1) (PTP-BAS) (Protein-tyrosine phosphatase 1E) (PTP-E1) (hPTPE1) (Protein-tyrosine phosphatase PTPL1)	PTPN13 PNP1 PTP1E PTPL1	cellular response to toxic substance [GO:0097237]; negative regulation of excitatory synapse assembly [GO:1904890]; negative regulation of protein phosphorylation [GO:0001933]; peptidyl-tyrosine dephosphorylation [GO:0035335]; protein dephosphorylation [GO:0006470]; regulation of phosphatidylinositol 3-kinase/protein kinase B signal transduction [GO:0051896]

464.44	431.91	885.03	172.64	425.1	231.31	398.13	183.1	1965.88	123.69	135.64	816.55	536.59	959.25	1516.54	488.2	354.82	1974.08	12062.9	Liprin-alpha-4 (Protein tyrosine phosphatase receptor type f polypeptide-interacting protein alpha-4) (PTPRF-interacting protein alpha-4)	PPFIA4 KIAA0897	synapse organization [GO:0050808]
605.37	609.01	852.86	200.37	647.77	772.63	294.59	379.39	1414.48	242.41	262.64	814.27	576.16	947.28	1263.56	279.16	422.9	1475.14	12059.99	NEDD4-binding protein 1 (N4BP1) (EC 3.1.-.-)	n4bp1 si:ch211-215n5.3 zgc:66437	innate immune response [GO:0045087]; negative regulation of cytokine production [GO:0001818]; negative regulation of proteasomal ubiquitin-dependent protein catabolic process [GO:0032435]; negative regulation of protein ubiquitination [GO:0031397]; negative regulation of viral genome replication [GO:0045071]; regulation of innate immune response [GO:0045088]
554.59	1468.57	1131	385.38	1591.04	992.12	102.45	313.88	345.69	274.64	430.12	601.19	617.71	942.26	530.12	279.57	979.23	518.74	12058.3	Friend leukemia integration 1 transcription factor (Proto-oncogene Fli-1)	FLI1	cell differentiation [GO:0030154]; regulation of transcription by RNA polymerase II [GO:0006357]

880.43	519.05	1199.13	148.53	538.28	752.94	629.26	334.79	1238.57	222.47	186.22	740.26	643.61	664.36	1041.7	654.74	391.29	1262.1	12047.73	Glia-derived nexin (GDN) (Peptidase inhibitor 7) (PI-7) (Protease nexin 1) (PN-1) (Protease nexin I) (Serpin E2)	Serpine2 Pi7 Pn1	blood coagulation [GO:0007596]; cell differentiation [GO:0030154]; cerebellar granular layer morphogenesis [GO:0021683]; dense core granule biogenesis [GO:0061110]; detection of mechanical stimulus involved in sensory perception [GO:0050974]; embryo implantation [GO:0007566]; innervation [GO:0060384]; long-term synaptic potentiation [GO:0060291]; mating plug formation [GO:0042628]; negative regulation of blood coagulation [GO:0030195]; negative regulation of cell growth [GO:0030308]; negative regulation of cell population proliferation [GO:0008285]; negative regulation of phosphatidylinositol 3-kinase/protein kinase B signal transduction [GO:0051898]; negative regulation of plasminogen activation [GO:0010757]; negative regulation of platelet activation [GO:0010544]; negative regulation of platelet aggregation [GO:0090331]; negative regulation of protein catabolic process [illegible]
74.65	1610.26	190.16	87.17	2612.81	891.91	6.6	782.3	9.76	13.63	419.22	131.15	43.08	2748.82	462.83	276.17	1644.74	35.39	12040.65	Voltage-dependent calcium channel subunit alpha-2/delta-4 (Voltage-gated calcium channel subunit alpha-2/delta-4) [Cleaved into: Voltage-dependent calcium channel subunit alpha-2-4;Voltage-dependent calcium channel subunit delta-4]	Cacna2d4	

3538.86	311.59	927.1	780.49	812.86	933.72	263.21	282.97	513.04	1433.77	350.15	314.21	424.86	75.34	103.44	163.32	405.26	403.29	12037.48	Integrin alpha-11	Itga11	cell adhesion [GO:0007155]; fibroblast migration [GO:0010761]; integrin-mediated signaling pathway [GO:0007229]; substrate-dependent cell migration [GO:0006929]
2095.2	210.93	622.75	572.84	241.24	1671.39	112.79	125.05	327.4	703.35	127.85	615.79	1630.7	918.49	608.5	418.01	574.31	452.6	12029.19	Zinc finger SWIM domain-containing protein 6	Zswim6 Kiaa1577	neuron projection morphogenesis [GO:0048812]; regulation of neuron migration [GO:2001222]; striatal medium spiny neuron differentiation [GO:0021773]
1905.41	244.1	1321.57	793.01	496.02	800.87	33.25	116.74	288.72	720.38	203.95	2123.31	1067.51	275.43	410.42	366.95	402.54	457.95	12028.13	Probable RNA polymerase II nuclear localization protein SLC7A6OS (Solute carrier family 7 member 6 opposite strand transcript homolog)	slc7a6os zgc:103493	central nervous system development [GO:0007417]; developmental process [GO:0032502]; midbrain-hindbrain boundary structural organization [GO:0021552]; protein transport [GO:0015031]; somite development [GO:0061053]

101.31	2076	382.08	219.44	2006.71	563.03	134.54	1345.2	143.47	139.78	593.95	271.81	87.12	2311.54	246.8	105.42	973.11	326.42	12027.73	Complement component C8 beta chain (Complement component 8 subunit beta)	c8b	complement activation, alternative pathway [GO:0006957]; complement activation, classical pathway [GO:0006958]; killing of cells of another organism [GO:0031640]
555.23	320.61	1788.29	145.8	377.77	571.55	498.63	143.2	2024.56	140.81	96.84	710.69	317.17	362.99	1560.6	380.59	201.48	1828.58	12025.39	ADP-ribosylhydrolase ARH1 (EC 3.2.2.19) (ADP-ribose-L-arginine cleaving enzyme) ([Protein ADP-ribosylarginine] hydrolase) (ADP-ribosylarginine hydrolase) (hARH1)	ADPRH ARH1	protein de-ADP-ribosylation [GO:0051725]; protein modification process [GO:0036211]
229.15	153.2	456.19	194.15	224.11	1794.22	3524.32	91.96	153.55	108.75	141.26	1035.76	430.82	289.01	515.95	2020.98	390.06	255.66	12009.1	Golgi apparatus membrane protein TVP23 homolog B	Tvp23b Fam18b Fam18b1	

794.04	368.13	998.98	126.02	251.06	563.58	168.77	86.09	1718.02	177.84	98.67	1005.44	635.72	445.95	2008.04	193.86	178.97	2186.57	12005.75	E3 ubiquitin-protein ligase COP1 (EC 2.3.2.27) (Constitutive photomorphogenesis protein 1 homolog) (mCOP1) (RING finger and WD repeat domain protein 2) (RING-type E3 ubiquitin transferase RFWD2)	Cop1 Rfwd2 RNF200	positive regulation of proteasomal ubiquitin-dependent protein catabolic process [GO:0032436]; proteasome-mediated ubiquitin-dependent protein catabolic process [GO:0043161]; protein ubiquitination [GO:0016567]; response to ionizing radiation [GO:0010212]
563.68	1619.2	458.06	206.48	1965.85	744.37	205.66	888.61	276.53	226.62	475.44	385.54	382.32	1735.64	387.58	189.71	975.78	318.09	12005.16	Coiled-coil domain-containing protein 142	CCDC142 PSEC0243	
2509.81	340.46	235.26	556.54	394.49	586.68	412	396.55	42.73	2157.36	525.37	450.57	2027.58	472.94	121.05	286.07	458.85	22.93	11997.24	L-fucose dehydrogenase (EC 1.1.1.122) (17-beta-hydroxysteroid dehydrogenase DHRS10) (Dehydrogenase/reductase SDR family member 10) (Retinal short-chain dehydrogenase/reductase retSDR3)	HSD17B14 DHRS10 SDR3	L-fucose catabolic process [GO:0042355]; steroid catabolic process [GO:0006706]

46.26	0.36	6.23	58.8	0	3.66	7526.55	0.26	1.1	76.74	188.58	1	14.65	0	0.63	3919.51	123.06	6.45	11973.84	Epidermal growth factor receptor (EC 2.7.10.1)	EGFR		cellular response to epidermal growth factor stimulus [GO:0071364]; cellular response to estradiol stimulus [GO:0071392]; epidermal growth factor receptor signaling pathway [GO:0007173]; learning or memory [GO:0007611]; negative regulation of apoptotic process [GO:0043066]; neuron differentiation [GO:0030182]; positive regulation of cell growth [GO:0030307]; positive regulation of epithelial cell proliferation [GO:0050679]; positive regulation of ERK1 and ERK2 cascade [GO:0070374]; positive regulation of MAPK cascade [GO:0043410]; positive regulation of protein localization to early endosome [GO:1902966]; positive regulation of protein phosphorylation [GO:0001934]; positive regulation of transcription by RNA polymerase II [GO:0045944]
986.03	616.64	1146.14	20.94	581.04	122.56	1764.39	308.07	731.21	35.45	251.23	367.64	540.33	1074.33	915.23	1394.47	490.52	624	11970.22	Trinucleotide repeat-containing gene 6C protein	TNRC6C KIAA1582		miRNA-mediated gene silencing by inhibition of translation [GO:0035278]; miRNA-mediated post-transcriptional gene silencing [GO:0035195]; positive regulation of nuclear-transcribed mRNA catabolic process, deadenylation-dependent decay [GO:1900153]; positive regulation of nuclear-transcribed mRNA poly(A) tail shortening [GO:0060213]

919.91	997.21	772.93	193.81	605.45	935.76	15.95	364.01	87.78	300.74	355.7	407.09	1906.13	1634.19	834.36	182.81	848.03	608.32	11970.18	BBSome complex member BBS1 (Bardet-Biedl syndrome 1 protein homolog)	Bbs1	adult behavior [GO:0030534]; brain morphogenesis [GO:0048854]; cartilage development [GO:0051216]; cerebral cortex development [GO:0021987]; cilium assembly [GO:0060271]; dendrite development [GO:0016358]; fat cell differentiation [GO:0045444]; fertilization [GO:0009566]; Golgi to plasma membrane protein transport [GO:0043001]; hippocampus development [GO:0021766]; hormone metabolic process [GO:0042445]; intraciliary retrograde transport [GO:0035721]; lipid metabolic process [GO:0006629]; microtubule cytoskeleton organization [GO:0000226]; neural precursor cell proliferation [GO:0061351]; neuron migration [GO:0001764]; non-motile cilium assembly [GO:1905515]; olfactory behavior [GO:0042048]; photoreceptor cell maintenance [GO:0045494]; photoreceptor cell morphogenesis [GO:0008594]; protein localization [GO:0008104]; protein localization to cilium [GO:0061512];
1	14.15	360.38	0.91	68.84	15.13	1.45	34.25	4142.2	1.57	22.98	1089.35	2.05	107.47	3165.23	6.06	6.34	2923.88	11963.24	DmX-like protein 1 (X-like 1 protein)	DMXL1 XL1	vacuolar acidification [GO:0007035]
22.09	29.94	1427.46	64.03	56.91	482.6	3.23	22.13	3871.66	14.86	33.12	1275.88	21.58	56.15	2044.34	27.47	35.13	2468.17	11956.75	CD209 antigen-like protein D (DC-SIGN-related protein 3) (DC-SIGNR3) (CD antigen CD209)	Cd209d	defense response to bacterium [GO:0042742]; endocytosis [GO:0006897]; positive regulation of cytokine production [GO:0001819]

505.39	534.93	379.8	216.96	938.08	1364.89	155.88	736.76	118.61	315.58	447.21	626.82	1683.94	1614.3	735.74	254.73	842.91	468.73	11941.26	WASH complex subunit 4 (Strumpellin and WASH-interacting protein) (SWIP) (WASH complex subunit SWIP)	WASHC4 KIAA1033	cognition [GO:0050890]; endolysosomal toll-like receptor signaling pathway [GO:0140894]; endosomal transport [GO:0016197]; endosome organization [GO:0007032]; neuromuscular process [GO:0050905]; nuclear envelope budding [GO:0140591]; protein transport [GO:0015031]; regulation of Arp2/3 complex-mediated actin nucleation [GO:0034315]; regulation of protein complex stability [GO:0061635]
792.24	1106.58	555.68	326.91	987.5	523.1	11.1	683.12	51.75	439.19	358.83	303.53	2657.23	1568.8	427.77	141.22	832.92	171.99	11939.46	Thioredoxin domain-containing protein 5 (EC 1.8.4.-) (EC 5.3.4.1) (Endoplasmic reticulum resident protein 46) (ER protein 46) (ERp46) (Plasma cell-specific thioredoxin-related protein) (PC-TRP) (Thioredoxin-like protein p46)	Txndc5 Tlp46	

656.51	1647.1	69.52	750.04	596.42	1516.08	0	1333.83	0	1471.54	1418.24	0	573.6	1559.9	133.6	29.08	159.14	8.48	11923.08	Tetratricopeptide repeat protein 5 (TPR repeat protein 5) (Stress-responsive activator of p300) (Protein Strap)	TTC5	cellular response to starvation [GO:0009267]; DNA damage response [GO:0006974]; DNA repair [GO:0006281]; positive regulation of mRNA catabolic process [GO:0061014]; positive regulation of transcription by RNA polymerase II [GO:0045944]
333.28	743.23	1240.99	133.49	702.42	1033.59	145.71	196.35	1391.01	122.99	184.69	1116.66	383.48	494.66	1646.63	166.9	317.38	1568.23	11921.69	Cell death regulator Aven	Aven	apoptotic process [GO:0006915]; negative regulation of apoptotic process [GO:0043066]
58.74	0.79	3.4	421.1	0	5.56	6481.65	0	0	68.9	118.51	0	7.34	0	4.08	4653.53	91.35	4.01	11918.96	Kelch domain-containing protein 1	Klhdc1	protein ubiquitination [GO:0016567]; ubiquitin-dependent protein catabolic process via the C-end degron rule pathway [GO:0140627]
1346.55	397.97	649.51	380.62	410.83	452.62	1798.01	218.93	243.81	440.5	312.28	542.82	775.94	572.79	316.69	2009.15	683.9	363.5	11916.42	Metallophosphoesterase 1 (EC 3.1.-.-) (Post-GPI attachment to proteins factor 5)	mppe1 pgap5 zgc:112219	endoplasmic reticulum to Golgi vesicle-mediated transport [GO:0006888]; GPI anchor biosynthetic process [GO:0006506]

762.08	596.55	800.41	286.14	502.56	814.05	892.93	229.7	983.79	268.38	201.14	631.35	796.53	852.68	947.38	687.35	475.51	1177.26	11905.79	Bromodomain-containing protein 1 (BR140-like protein) (Bromodomain and PHD finger-containing protein 2)	BRD1 BRL BRPF2	chromatin remodeling [GO:0006338]; erythrocyte maturation [GO:0043249]; positive regulation of erythrocyte differentiation [GO:0045648]; regulation of developmental process [GO:0050793]; regulation of DNA-templated transcription [GO:0006355]; regulation of hemopoiesis [GO:1903706]; regulation of transcription by RNA polymerase II [GO:0006357]; response to electrical stimulus [GO:0051602]; response to immobilization stress [GO:0035902]
648.6	372.01	1526.19	133.09	342.25	2040.49	271.85	180.72	788.85	188.43	162.88	921.06	777.6	305.32	1098.46	288.25	301.12	1557.66	11904.83	3-hydroxyisobutyryl-CoA hydrolase, mitochondrial (EC 3.1.2.4) (3-hydroxyisobutyryl-coenzyme A hydrolase) (HIB-CoA hydrolase) (HIBYL-CoA-H)	hibch zgc:110824	valine catabolic process [GO:0006574]
5.6	2804.36	110.7	4.73	2192.37	130.44	35.99	488.2	0	5.29	788.52	114.91	0	2813.45	22.96	16.46	2358.31	11.01	11903.3	Lysyl oxidase homolog 2A (EC 1.4.3.13) (Lysyl oxidase-like protein 2A)	loxl2a	collagen fibril organization [GO:0030199]; endothelial cell migration [GO:0043542]; endothelial cell proliferation [GO:0001935]; epithelial to mesenchymal transition [GO:0001837]; heterochromatin organization [GO:0070828]; negative regulation of DNA-templated transcription [GO:0045892]; negative regulation of stem cell population maintenance [GO:1902455]; negative regulation of transcription by RNA polymerase II [GO:0000122]; peptidyl-lysine oxidation [GO:0018057]; positive regulation of chondrocyte differentiation [GO:0032332]; positive regulation of epithelial to mesenchymal transition [GO:0010718]; response to hypoxia [GO:0001666]; sprouting angiogenesis [GO:0002040]

400.77	924.02	1356.51	125.51	899.69	1001.35	155.1	347.64	569.69	133.14	225.39	797.07	607.42	1171.92	1309.38	189.4	560.79	1117.68	11892.47	Dipeptidyl peptidase 9 (EC 3.4.14.5)	dpp9 zgc:152900	proteolysis [GO:0006508]; regulation of inflammatory response [GO:0050727]
578.24	897.43	406.61	749	753.53	1665.85	257.26	938.5	443.04	877.82	953.84	684.51	386.27	975.43	342.8	328.29	327.74	323.66	11889.82	Methionine aminopeptidase 1D, mitochondrial (MAP 1D) (MetAP 1D) (EC 3.4.11.18) (Methionyl aminopeptidase type 1D, mitochondrial) (Peptidase M 1D)	metap1d map1d zgc:110461	proteolysis [GO:0006508]
158.19	126.14	1177.22	98.14	89.16	184.17	721.35	32.61	2256.24	61.37	51.88	1000.05	74.03	155.74	2101.71	585.28	105.6	2901.86	11880.74	Homeobox protein Nkx-6.1 (Homeobox protein NK-6 homolog A)	Nkx6-1 Nkx6.1 Nkx6a	cell differentiation [GO:0030154]; cellular response to cytokine stimulus [GO:0071345]; cellular response to peptide hormone stimulus [GO:0071375]; central nervous system neuron differentiation [GO:0021953]; endocrine pancreas development [GO:0031018]; negative regulation of oligodendrocyte differentiation [GO:0048715]; negative regulation of transcription by RNA polymerase II [GO:0000122]; neurogenesis [GO:0022008]; neuron differentiation [GO:0030182]; oligodendrocyte differentiation [GO:0048709]; pancreatic A cell differentiation [GO:0003310]; positive regulation of insulin secretion [GO:0032024]; positive regulation of neuron differentiation [GO:0045666]; positive regulation of oligodendrocyte differentiation [GO:0048714]; regulation of axon extension [GO:0030516]; regulation of neuron migration [GO:2001222]; regulation of transcription by RNA polymerase II [GO:0006357]; [illegible]

0.25	1.54	102.64	4.64	1.32	16.59	1.63	3.43	4184.27	3.6	3.13	1277.17	4.92	7.09	2989.03	9.37	20.54	3236.38	11867.54	Cytosolic carboxypeptidase 1 (EC 3.4.17.-) (EC 3.4.17.24) (ATP/GTP-binding protein 1) (Protein deglutamylase CCP1)	agtpbp1 ccp1	C-terminal protein deglutamylation [GO:0035609]; cerebellar Purkinje cell differentiation [GO:0021702]; chordate embryonic development [GO:0043009]; eye photoreceptor cell differentiation [GO:0001754]; mitochondrion organization [GO:0007005]; neuromuscular process [GO:0050905]; olfactory bulb development [GO:0021772]; protein side chain deglutamylation [GO:0035610]; proteolysis [GO:0006508]; T cell differentiation in thymus [GO:0033077]
400.02	184.49	499.04	221.41	221.43	370.29	4193.09	107.15	286.97	132.74	155.92	167.53	364.4	188.55	319.69	3537.37	218.88	296.06	11865.03	Nck-associated protein 1 (NAP 1)	nckap1 si:dkey-234n3.1 wu:fd05c01	cell migration [GO:0016477]; cell morphogenesis [GO:0000902]; cell projection assembly [GO:0030031]; cortical actin cytoskeleton organization [GO:0030866]; neuron projection morphogenesis [GO:0048812]
