# Supplementary material for: A new lineage of Ranavirus micropterus1 infects ornamental wrasses (Macropharyngodon choati) from the Great Barrier Reef and causes severe disease in captivity
Source: Front Vet Sci. 2026 May 18;13:1829414. doi: 10.3389/fvets.2026.1829414 (PMC13224474; doi:10.3389/fvets.2026.1829414)
Supplement: Supplementary file 3 [file Table_3.PDF]

Table S3. Read depth and contigs among sample types.

Sample ID	Species	Tissue	Sample	Total reads	Contigs	N50
W1.1	Macropharyngodon choati	Spleen	W1-1	57,984,236	135331	1515
W1.2	Macropharyngodon choati	Liver	W1-2	55,237,154	146945	1443
W1.3	Macropharyngodon choati	Brain	W1-3	56,657,276	252810	1289
W2.1	Macropharyngodon choati	Spleen	W2-1	56,922,386	111341	1086
W2.2	Macropharyngodon choati	Liver	W2-2	52,630,940	136072	1393
W2.3	Macropharyngodon choati	Brain	W2-3	58,123,578	185322	1438
W3.1	Macropharyngodon choati	Spleen	W3-1	55,036,118	35305	2245
W3.2	Macropharyngodon choati	Liver	W3-2	51,281,314	125489	1236
W3.3	Macropharyngodon choati	Brain	W3-3	53,389,004	241874	1814
W4.1	Macropharyngodon choati	Spleen	W4-1	56,002,672	104651	1025
W4.2	Macropharyngodon choati	Liver	W4-2	52,781,266	117339	1119
W4.3	Macropharyngodon choati	Brain	W4-3	56,828,926	241328	1147
W5.1	Macropharyngodon choati	Spleen	W5-1	56,506,838	164824	1581
W5.2	Macropharyngodon choati	Liver	W5-2	52,832,582	164139	1407
W5.3	Macropharyngodon choati	Brain	W5-3	51,554,294	266697	1756
W6.1	Macropharyngodon choati	Spleen	W6-1	49,127,882	115055	1264
W6.2	Macropharyngodon choati	Liver	W6-2	49,481,968	151548	1429
W6.3	Macropharyngodon choati	Brain	W6-3	57,554,352	250964	1963
