# Supplementary material for: A new lineage of Ranavirus micropterus1 infects ornamental wrasses (Macropharyngodon choati) from the Great Barrier Reef and causes severe disease in captivity
Source: Front Vet Sci. 2026 May 18;13:1829414. doi: 10.3389/fvets.2026.1829414 (PMC13224474; doi:10.3389/fvets.2026.1829414)
Supplement: Supplementary file 4 [file Table_4.PDF]

Table S4. Sample names and well numbers. Related to Figure S2.

Number	Sample type
1	Neat P0
2	Neat P0
3	Neat P0
4	Neat P0
5	Neat P0
6	Neat P0
7	1in10 P0
8	1in10 P0
9	1in10 P0
10	1in10 P0
11	1in10 P0
12	1in10 P0
13	Empty
14	Passage P1
15	Passage P1
16	Passage P1
17	Passage P1
18	Barramundi spleen (negative control)
19	Barramundi spleen (negative control)
20	Barramundi spleen (negative control)
21	Wrasse spleen
22	Wrasse spleen
23	Wrasse spleen
24	Barramundi spleen (negative control)
25	Barramundi liver (negative control)
26	Barramundi spleen (negative control)
27	Barramundi liver (negative control)
28	Wrasse spleen
29	Wrasse spleen
30	Wrasse spleen
31	Wrasse liver
32	Wrasse liver
33	Wrasse liver
34	Wrasse liver
35	Wrasse brain tissue
36	Wrasse brain tissue
37	Wrasse brain tissue
38	Wrasse brain tissue
