# Supplementary figures and images for: A new lineage of Ranavirus micropterus1 infects ornamental wrasses (Macropharyngodon choati) from the Great Barrier Reef and causes severe disease in captivity
Source: Front Vet Sci. 2026 May 18;13:1829414. doi: 10.3389/fvets.2026.1829414 (PMC13224474; doi:10.3389/fvets.2026.1829414)

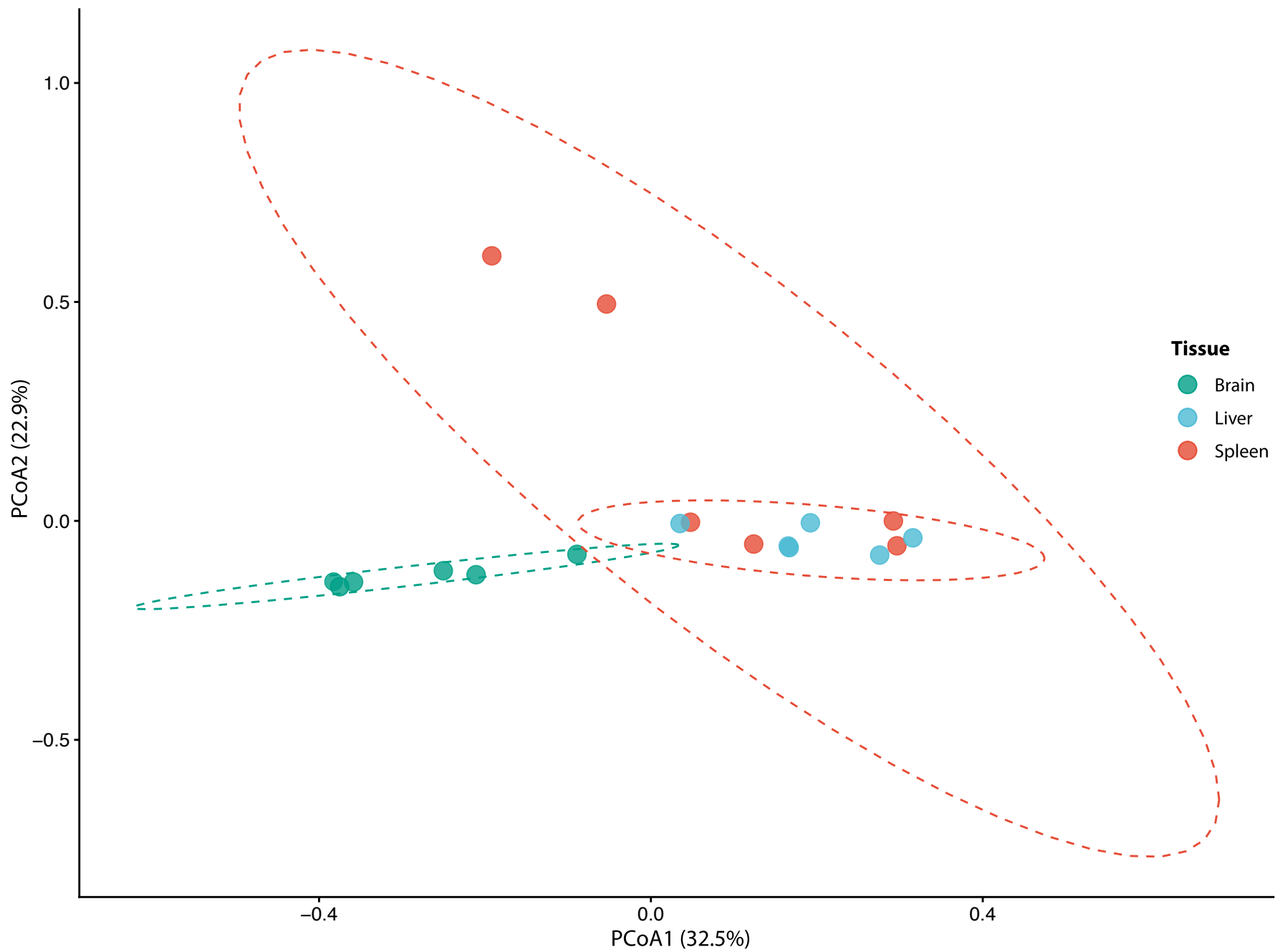

Figure S1. Principal coordinate analysis of host transcriptomes by tissue sample.

Supplement: Supplementary file 5 [file Image_1.PDF]
