# Supplementary material for: A new lineage of Ranavirus micropterus1 infects ornamental wrasses (Macropharyngodon choati) from the Great Barrier Reef and causes severe disease in captivity
Source: Front Vet Sci. 2026 May 18;13:1829414. doi: 10.3389/fvets.2026.1829414 (PMC13224474; doi:10.3389/fvets.2026.1829414)
Supplement: Supplementary file 6 [file Image_2.PDF]

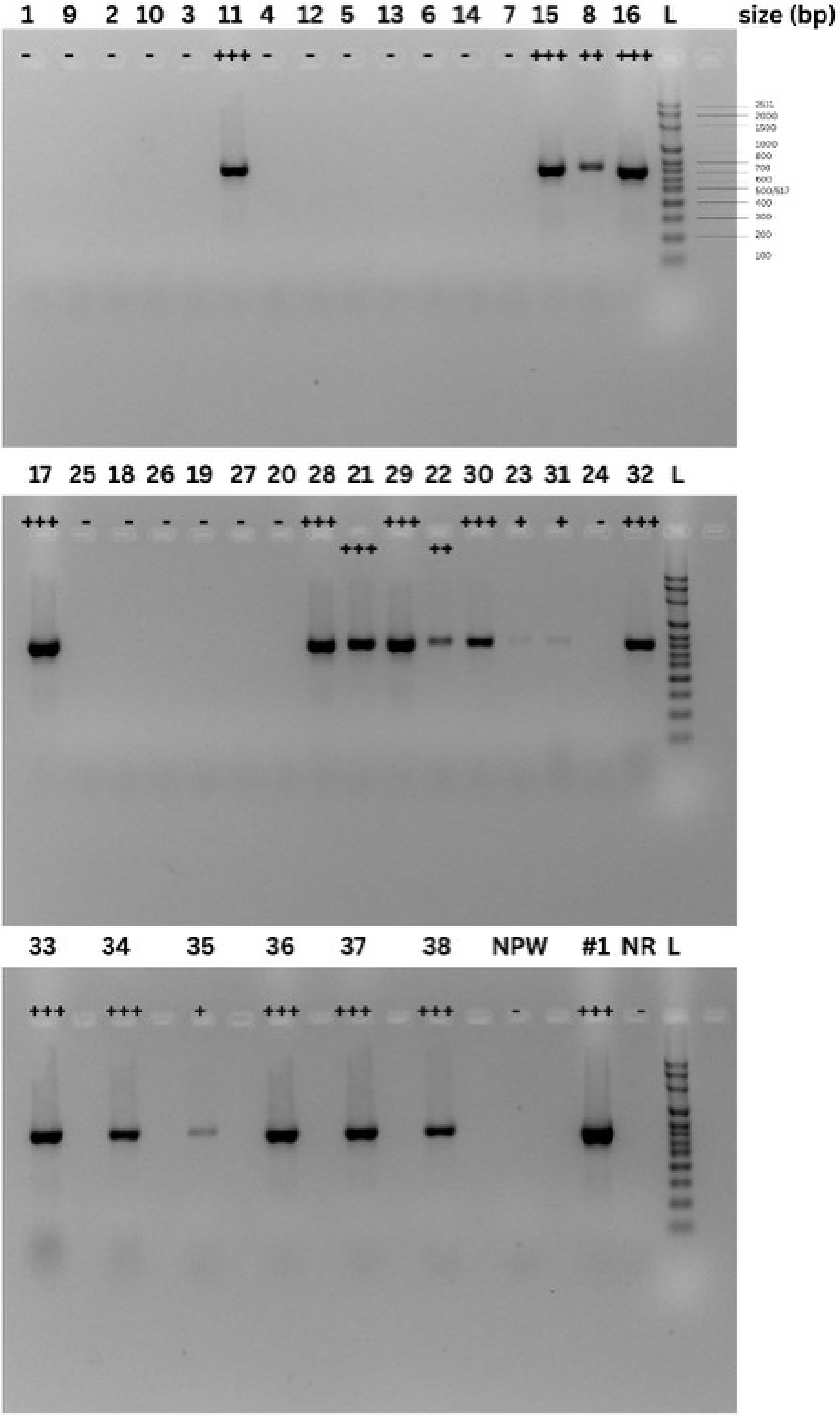

Figure S2. PCR detection of McRV on agarose gel.
Band numbers correspond to sample IDs listed in Table S4.
